# Relevance of the uMap Collaborative Platform as Support for Choropleth Mapping of a Traffic-Light Statistical Signal Atlas of All-Cause Mortality During the First French Lockdown: Geospatial Analysis

**DOI:** 10.2196/82855

**Published:** 2026-08-27

**Authors:** Anne Quesnel-Barbet, Thierry Pages, Julien Soula, Gilles Maignant, Arnaud Hansske

**Affiliations:** 1 Public Health - Regional House of Clinical Research (MRRC), Lille University Hospital (CHU de Lille), 6 rue du Pr Laguesse, CS 70001, Lille cedex, 59037, France, 33 0658066808; 2 Faculty of Medicine, Laboratory of RETINES, University of Côte d'Azur (UCA), Nice Cedex 2, France; 3 TITSOFT Inc, Montpellier, France; 4 ULR 2694 - METRICS: Évaluation des technologies de santé et des pratiques médicales, University of Lille, CHU Lille, F-59000 Lille, France; 5 Research Group on Law, Economics, and Management (GREDEG), National Centre for Scientific Research (CNRS), University Côte d’Azur (UCA), Nice, France; 6 DSI/CIO/CMIO, Catholic Hospitals of Lille (GHICL), Lille, France

**Keywords:** geomatics atlas, uMap online map creator, collaborative platform, traffic light semantic, choropleth mapping, geographic information systems, GIS, information services, information science, health information systems, mortality

## Abstract

**Background:**

The growing need for and interest in geomatics in the medical sector, as well as the pandemic crisis, led us to create a France-wide geomatics project aimed at producing several atlases of all-cause mortality at the municipal and submunicipal district levels via uMap France, a free and open-source collaborative map-sharing platform. In 2020, we decided to circumvent the obstacle of accessing detailed COVID-19 data by adopting a mortality-based approach to map the consequences of the crisis.

**Objective:**

The aim of the uMap study was to provide a webmapping platform with original visualization and knowledge as well as decision-making aids that complement existing information and are relevant to public and health care professionals. Our main hypotheses were as follows: (1) The medical sector could develop a private uMap platform dedicated to health; (2) interest in a municipal mortality atlas for France linked to the pandemic crisis will increase, even if it is produced after the pandemic; and (3) sharing the atlases with the uMap community will enhance their appeal and inspire the creation of similar atlases, owing to the new “experimental choropleth layer” recently developed by the uMap team.

**Methods:**

This approach focused on 3 main parts—data management (data collection, cleansing, and scheduling) and geomatic engineering through a 2-step geomatic action plan to create atlases of the first lockdown period in France displayed on the uMap platform. A logarithmically transformed variable allowed us to obtain an immediate statistical signal of excess mortality or submortality via the Traffic-Light Atlas.

**Results:**

The uMap Traffic-Light Atlas display provides instant statistical signals at a glance, owing to the semantic interplay of colors. The atlas’s double legends make it easy to compare specific regions (northeast, northwest, southeast, and southwest) with all of France. The atlas revealed excess mortality in 41.7% (14,503/34,833) of the municipalities. Of the municipalities, 35% (12,198/34,833) were in the green class (close to average to 2 times the average), 5% (1724/34,833) were in the orange class (2‐4 times the average), and 1.7% (581/34,833) were in the red class (4‐11 times higher than average).

**Conclusions:**

We innovated, enriched, and reinforced the value of uMap for visual rendering by instantiating colored choropleth map atlases and double legends and showed its relevance to the health care sector. We focused on the Traffic-Light Atlas, which is the most relevant aspect because of the instant message it conveys and its interpretability for all audiences. The uMap community can share our all-cause mortality atlases. A second version of the atlas encompassing 4 periods in 2020 and containing a minor error will be updated using either the “experimental choropleth layer” feature recently developed by the uMap team or, if this feature proves insufficient, the geomatic optimization process via the R project.

## Introduction

### Background

The growing need for and interest in geomatics in the medical sector, particularly in hospitals, have led us to strengthen our interdisciplinary collaborations on joint geomatics research projects over the past 2 decades [1-3] (Multimedia Appendix 1). Geomatics is, in a way, the combination of computer science and geography, providing digital mapping via easy-to-use software through the use of more complex software environments such as geographic information systems (GIS), programming languages, scripting languages, and geoservers to produce online maps [3]. Mapping can be an individual project or a collaborative project, owing to platforms with or without the ability to share cartographic productions. More information is provided in Multimedia Appendix 1 and Multimedia Appendix 2.

The COVID-19 pandemic led us to create an all-cause mortality atlas in France (version 1—the period of the first French lockdown) with maps that can be viewed at a very detailed level for municipalities and submunicipal districts (Marseille, Lyon, and Paris) [1,4-7]. This atlas offers a retrospective view of the period under study during the 2020 crisis. The inability to access data on reported SARS-CoV-2 cases with detailed geographical origins at the municipal level in 2020 for the purpose of mapping coronavirus cases at a fine mesh explains our choice to circumvent this obstacle. We therefore decided to use the (open) database of French mortality data from 2020 provided by the French National Institute of Statistics and Economic Studies (INSEE; Multimedia Appendix 3), which has several advantages: (1) The data are broken down at the municipal level (fine grain); (2) access is open (open data); and (3) we can avoid using young coronavirus databases (at the time of our data collection process) that have significant biases, such as “coding errors for deaths due to coronavirus” [8]. However, our monitoring of the evolution of the COVID-19 pandemic has shown, on the one hand, that epidemiological data on the incidence of coronavirus have become unavailable at the municipal level and the few coronavirus cluster maps available at the municipal level have eventually disappeared from the French media. On the other hand, in our neighboring country, Belgium, the incidence of coronavirus per 100,000 inhabitants was reported at the municipal level [9]. Many French studies and accompanying maps are also based on highly aggregated open coronavirus data [10].

Our monitoring of technologies, IT developments, and data sources led us, at the beginning of our mortality atlas project, to focus on 2 geomatic environments. Here, we present a study of geomatics based on uMap France (OpenStreetMap [OSM] server), a free and open-source software (FOSS) [11]. E-atlases based on a developed environment are now available online [7]. Upon querying the OSM server [12,13], we found no “color-coded thematic maps (also known as choropleth maps) with associated legends” created, shared, or posted online by uMap France community users. Examples of inventory maps and analysis maps (the former allowing users to move, explore, identify, place, locate, intervene, allocate, and manage and the latter allowing users to understand, evaluate, and analyze), as well as 2 other projects—uMap Framacarte (Framasoft server) and the uMap of public service (government server)—are displayed on the new uMap project home page created by Yohan Boniface [14].

The main geomatic methodological approaches for obtaining results were divided into 2 stages: step 1: geomatics without webmapping and step 2: geomatics with webmapping [15]. Our atlas maps (instances) were the main results of our study. Among the 3 e-atlases, each created from a statistical variable, the thematic “Traffic-Light Atlas” created from the “Khi2LnFr” variable displays 1 of 3 colors—red, orange, or green—if the municipality exclusively had an excess mortality statistical signal. For the submortality values, a pale yellow color represented values outside the Traffic-Light semantics. The Traffic-Light Atlas appears even more relevant, as cartographic information is instantly visible and easily interpreted by the human eye. We qualified this Traffic-Light Atlas as the best messenger.

### Aims and Objectives

We had 3 main goals for this geomatics project for mortality atlases.

The first was to provide an original and relevant decision-making aid in the “uMap - FOSS online geomatic and mapping platform” [11] (on a French scale), which allowed us to produce and disseminate our mortality atlas for which the statistical and geographical information was processed and analyzed then mapped on a fine territorial grid (municipalities and submunicipal districts). This high level of detail in the processing of information provided new insights compared with studies in which information was aggregated at coarser departmental and regional administrative levels, leading to visualization and interpretation biases (ie, a mesh that is too aggregated can hide or render invisible or imperceptible other information remaining to be discovered) [16]. The departmental level is an administrative division between the municipality and the region. It has an elected council and a prefecture, which stands for the state. A regional level consists of an administrative division, with its own regional government.

The second goal for our version 1 atlas was to process information on mortality in 2020 (compared with 2018 and 2019) based on the period of the first French lockdown.

The third goal was to facilitate the comprehension of thematic information mapped on the scale of France and its overseas departments and regions (DROMs)—administrative divisions in France—for a wide audience of users (the general public, health care professionals, and the uMap community) of our atlases in uMap. To this end, we constructed statistical indicators of excess mortality, which are displayed when the mouse pointer is hovered over the maps in the atlas. These excess mortality indicators (per 10,000 inhabitants at the municipal level) are also displayed in the map tool tips of the atlas. Users can connect to the uMap environment for each atlas instance associated with a statistical variable in 4 layers to subdivide France into 4 geographical areas: northeast, northwest, southeast, and southwest.

### Hypotheses

The first hypothesis was that the evaluation of geomatic environments in the health sector provides new perspectives for medical disciplines in terms of information and communications technology (ICT) knowledge, ICT development, and the use of geomatic tools for the health sector. If this hypothesis is confirmed, this research will create a new knowledge base for developing a local uMap server dedicated to the health sector.

The second hypothesis posited that user interest in a municipal mortality atlas related to the pandemic crisis will increase, even if it is put into production after the pandemic, owing to the general public’s interest in examining whether there was excess mortality in their municipality during the crisis (during or outside the lockdown period, versions 1 and 2 of the atlas), made possible by the fine mesh of the atlas maps, the instant visualization of excess mortality by the Traffic-Light Atlas, and the sharing of maps via the uMap France platform and the Grouping of the Institut Catholique de Lille Hospitals (GHICL) website [6,7,17,18]. If this hypothesis is confirmed, the uMap Traffic-Light Atlas will become more appealing due to its semantics (by ending map interpretation errors in excess mortality or submortality).

The third hypothesis posited that uMap is a remarkably interesting alternative to existing geomatics methods and platforms for collaborative online mapping. First, uMap offers attractive features. The most important of these is its minimal programming requirements. Second, the uMap team allows users to request help or new features as needed. Finally, we demonstrated the feasibility of implementing choropleth maps (based on GeoJSON) with associated double legends (based on image format) through this study and by contacting the uMap team. If this hypothesis is confirmed, the uMap team will be able to provide support and new features concerning the facilitation of creating and associating a legend (image file) with a choropleth map (GeoJSON file). In the short to medium term, we hoped that sharing these atlases with the uMap community will enhance their appeal through their visual and semantic quality and inspire the creation of other similar atlases, owing to the newly and recently developed experimental choropleth layer.

After the introduction and background, we describe the methods and processes involved in developing atlas maps for the uMap environment, followed by the results focused on the Traffic-Light Atlas and the ease of use (platform ergonomics) and cartography (information visualization and interpretation) of uMap France. We then present discussions on the geomatic process (uMap software package) and on the advantages and disadvantages in terms of design and use. The article then describes how the evaluation of the uMap environment and related tasks can be assessed in terms of performance, attractiveness, and profitability and what new developments, geomatic instantiations, and other improvements to the environment can be envisaged. We end with our conclusions.

Multimedia Appendix 2 provides further information on the state of the art in geomatics, including a history of digital cartography and an introduction to geomatics in health care.

## Methods

### Materials

#### Summary

**Figure 1.**
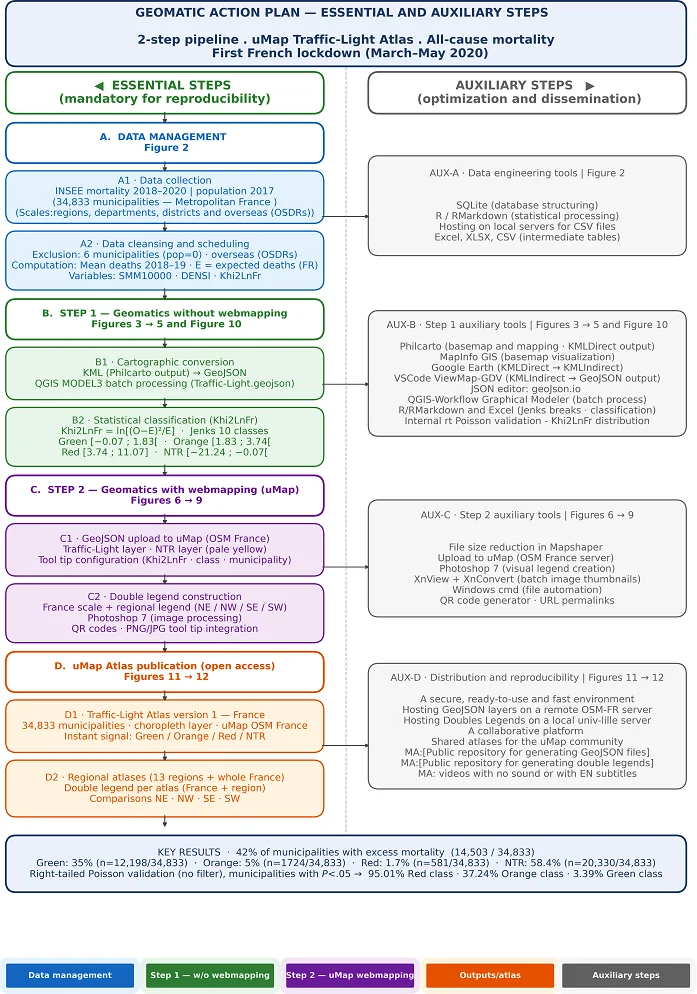
Data pipeline and software architecture showing the essential and auxiliary steps of the Geomatics Action Plan for creating atlases, including the mortality atlas for uMap. The explicit division between essential and auxiliary tools and between Philcarto and QGIS ensures that the workflow is both reproducible at its core and extensible for researchers who wish to add optimization layers. See Figure 2A for the synthetic architecture overview and Figure 2B for technical details of data transformation. GIS: geographic information system; INSEE: French National Institute of Statistics and Economic Studies; KML: Keyhole Markup Language; NTR: nothing to report; OSM: OpenStreetMap; rt: right-tailed; w/o: without.

**Figure 2.**
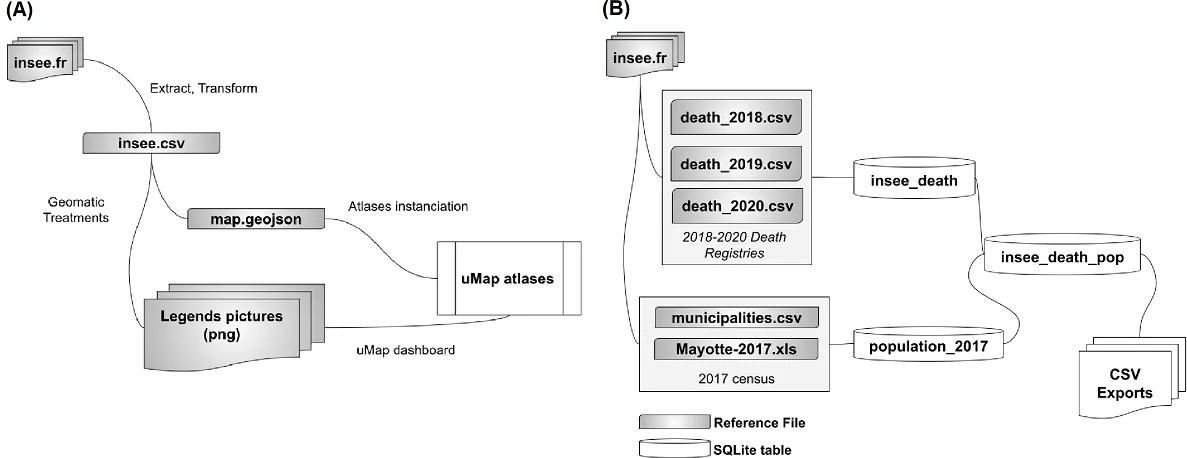
(A) Synthetic architecture and (B) data transformation diagram.

This section describes the key material, data sources, software, formats, and standards used in this geomatics research project (see Table 1); for more details, see Multimedia Appendix 3, Multimedia Appendix 4, and Multimedia Appendix 5. We combined French open data sources with a hybrid software ecosystem (open source and proprietary) for spatial analysis of mortality data. The technical architecture enabled seamless integration between free software and specialized proprietary solutions, perfecting the mortality and geomatic data-processing pipeline (see Figures 1 and 2).

**Table 1.** Summary of the key materials, software, data sources, formats, and standards.

Category	Description	Type	Formats	References
Primary data sources				
Statistical data^a^	Municipal mortality 2018‐2020; 2017 census nomenclature	Open data	CSV, XLSX	Multimedia Appendices 3 and 4 and [4,5]
Geographic, geolocated data	Administrative boundaries, COG^b^, and ADMIN EXPRESS^c^ 2020-IGN raw vectorial basemap; ODbL^d^	Open data	SHP^e^, MID^f^-MIF^g^ (proprietary), GPKG^h^ (open)	[19]
Other geographic, geolocated data	Departmental and regional maps; locations of ICU^i^^,^^j^ and emergency services	Open data	GeoJSON	[20-24]
Software environment				
Database processing	INSEE^k^ data aggregation via SQLite	Open source	SQLite	[25]
Database processing: statistical analysis	R project (v4.2.2); RStudio; Libraries: plyr and openxlsx; RMarkdown	Open-source; GNU GPL^l^^,^^m^ free; public domain	scripts, R	[26-30]
CAD^n^: GIS^o^ workflows	QGIS v3.18.1-Zürich [31]; GDAL^p^ 3.1.4; GEOS v3.8.1^q^; CRS^r^	Open source	QGS, EPSG-7030, EPSG-4326, WGS^s^-84	[32,33]
CAD: cartography, editing, simplification of geographic data	Philcarto v2021.d. and its Eclat utility (shapefile processing); Mapshaper (GeoJSON); Geojson.io online editor	Free, open source	Maps, Macro.PMC SHP, AI^t^, EMF^u^, KML^v^, GeoJSON	[34-37]
CAD: image processing (free)	XnView and XnConvert (batch processing); IrfanView (cropping)	Free	Image files SQL	[38-40]
CAD: proprietary software	Photoshop action ATN scripts; MapInfo GIS; Paint and Paint.Net (VBA^w^); SpreadsheetLight; other CAD software	Proprietary	Images, maps, GIS, ATN	[41-45]
CAD: uMap Platform, a Django project	Collaborative webmapping (uMap) v1.2.1 (WTFPL, FOSS^x^, OSM^y^-fr); creation, visualization, and export	Open source	KML, GeoJSON, uMap	[11-13,46-50]
CAD: virtual globe	Google Earth Pro v7.3.6: visualization and KML export	Free	KML	[49]
Code management	Visual Studio Code; extension: view map; Git Bash^z^	Open source	Source code	[45,51,52]
Standards and formats				
OGC^aa^, ISO^ab^, AFNOR^ac^, CEN^ad^, and W3C^ae^ standards	KML (EPSG:4326 only); GPX^af^	Standards	KML, GPX, EPSG-4326	[53-58]
IOGP^ag^ projection	WGS-84 CRS, EPSG:4326	Standards	EPSG-4326, KML prj	[59]
File formats^ah^	Shapefile (Esri); GeoJSON (RFC^ai^ 7946); MODEL3 (QGIS); ATN (Adobe); PMC macros (Philcarto)	Various: Proprietary. Open. Free.	SHP, ATN, GeoJSON, MODEL3 macro.PMC	[34,41,60-62]
Other CAD software				
Drawing and online maps	Testing of GIMP^aj^, Inkscape (Python scripts), and Mapbox^ak^	Various	Images, DWG online maps	[63-67]

a Data on municipal deaths covering March 1, 2020, through May 15, 2020, (inclusive of the first lockdown) sourced from the French National Institute of Statistics and Economic Studies (INSEE) in May 2020.

b COG: official geographical code.

c ADMIN EXPRESS: French administrative boundary dataset (updated March 25, 2020) produced by the French National Institute of Geographic and Forest Information (IGN).

d ODbL: open database license.

e SHP: shapefile file format.

f MID: MapInfo data file format.

g MIF: MapInfo interchange file format.

h GPKG: GeoPackage file format.

i ICU: intensive care unit.

j Data source: data.gouv.fr.

k INSEE: French National Institute of Statistics and Economic Studies.

l GNU GPL: GNU General Public License.

m Packages licensed under GPL and Massachusetts Institute of Technology (MIT) licenses.

n CAD: computer-aided design.

o GIS: geographic information system.

p GDAL: Geospatial Data Abstraction Library.

q GEOS: a C/C++ library for computational geometry.

r CRS: coordinate reference system.

s WGS: World Geodetic System.

t AI: Adobe Illustrator.

u EMF: Enhanced Metafile Format.

v KML: Keyhole Markup Language.

w VBA: Visual Basic for Applications.

x FOSS: free and open-source software.

y OSM: OpenStreetMap.

z BASH: Bourne-Again Shell.

aa OGC: Open Geospatial Consortium.

ab ISO: International Organization for Standardization.

ac AFNOR: French Association for Standardization; France’s member body of ISO.

ad CEN: European Committee for Standardization.

ae W3C: World Wide Web Consortium.

af GPX: GPS eXchange format.

ag IOGP: International Association of Oil & Gas Producers.

ah Conversion utilities concern Mapshaper and ATN formats.

ai RFC: request for comments.

aj GIMP: GNU Image Manipulation Program.

ak Monitoring through GIMP, Inkscape, and Mapbox for drawing, online maps, and workflow optimization.

#### Rationale for Primary Tool Selection

Philcarto is French statistical data cartographic software specifically designed by a geographer. It implements Jenks natural breaks optimization directly on statistical tabular data, applying validated cartographic semiology rules (color palettes, class breaks) and natively producing Keyhole Markup Language (KML)–Direct output for subsequent use in other geomatic environments. Philcarto is the only tool in our workflow that simultaneously performs (1) statistical classification of Khi2LnFr, (2) Traffic-Light color encoding, and (3) KML export in a single, validated operation. It has been used in French academic geomatics workflows for more than 15 years.

QGIS is used exclusively for the spatial engineering step that Philcarto cannot perform: joining the Philcarto-classified KML attribute table to the French National Institute of Geographic and Forest Information (IGN) ADMIN-EXPRESS municipal geometry shapefile and converting the result to GeoJSON via the QGIS Model3 Graphical Modeler for reproducible batch processing.

This sequential, nonredundant use of both tools reflects their respective strengths: Philcarto for expert cartographic classification and QGIS for spatial data engineering and format interoperability. This division of labor is consistent with the multisoftware public health surveillance workflows described in the literature [68].

In addition, we used a number of other tools for specific functions. Google Earth was used to visually verify the data generated by KML-Direct, which was then exported in an indirect KML format (transformed) in the auxiliary optimization step. In Visual Studio (VS) Code, KML-Indirect files were manually inspected and converted to the GeoJSON format using the “View Map GDV 2.4” extension so that they can be read and processed by QGIS, also in the auxiliary optimization step. Mapshaper was used for GeoJSON file size reduction to meet the uMap server upload constraints during the auxiliary optimization step.

**Figure 3.**
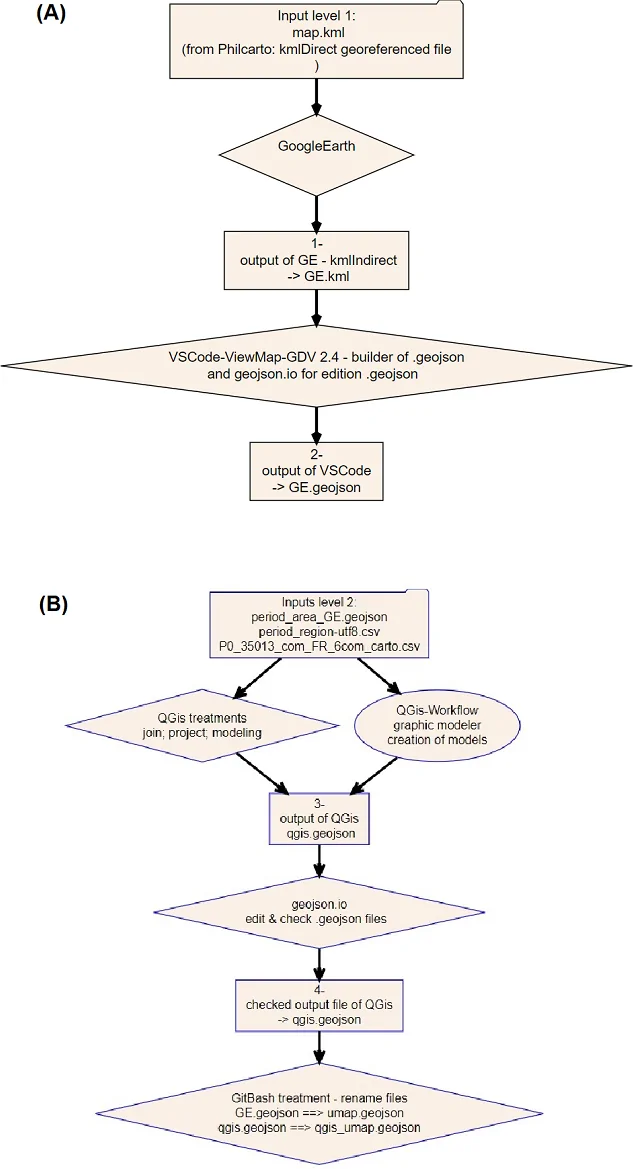
Data pipeline of the geomatic processing software system during step 1: geomatics without webmapping for (A) level 1 input and (B) level 2 inputs. There were 3 types of information: rectangle: input-output result; rhombus or diamond: software processing; and ellipse: QGIS workflow modeler. See Multimedia Appendix 6 [69] and Multimedia Appendix 7 [70]. VS: Visual Studio.

**Figure 4.**
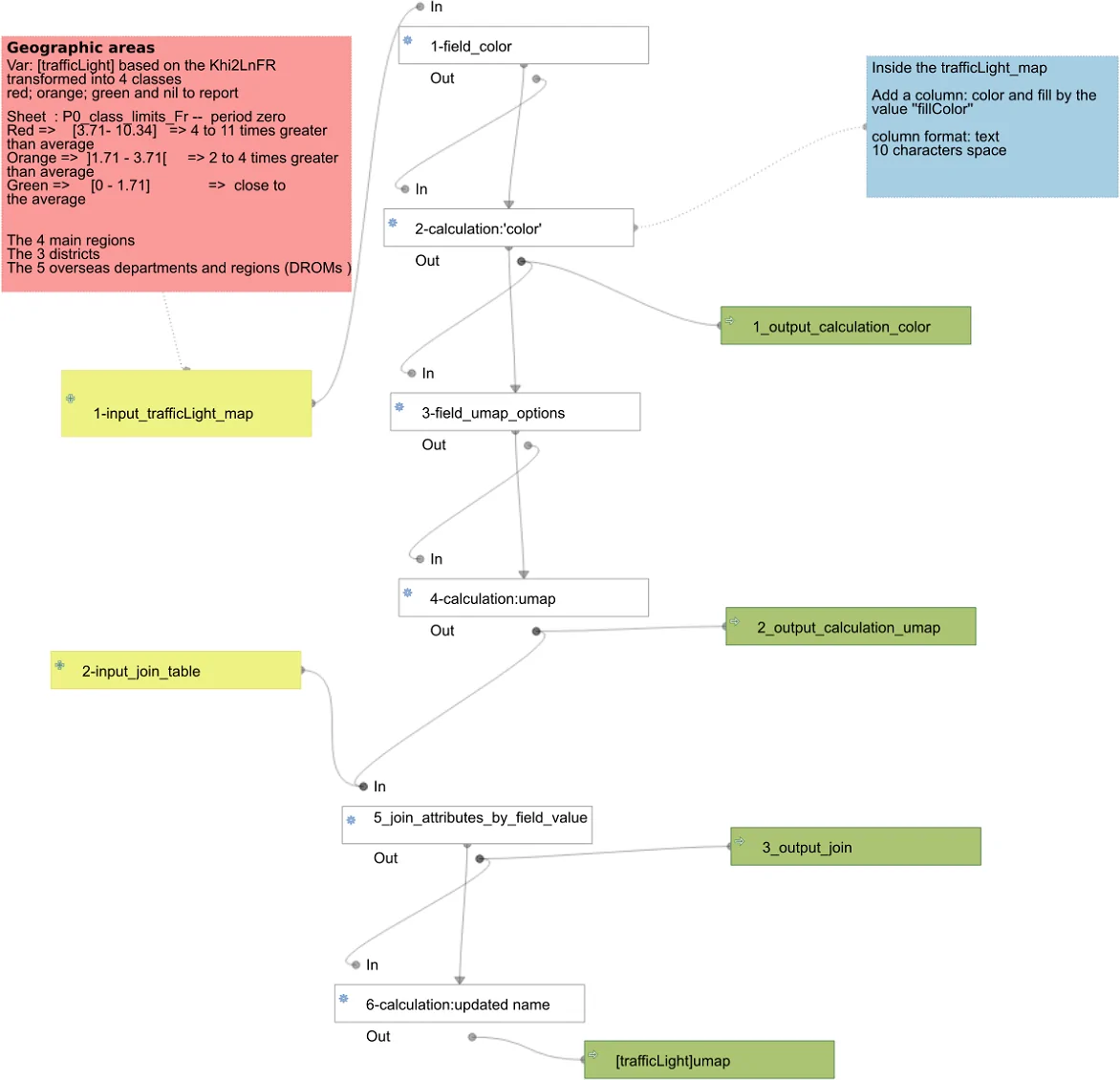
Workflow QGIS (MODEL3) for “part 1: obtaining the Traffic-Light.geojson map for uMap” in step 1: geomatics without webmapping. See Multimedia Appendix 8.

**Figure 5.**
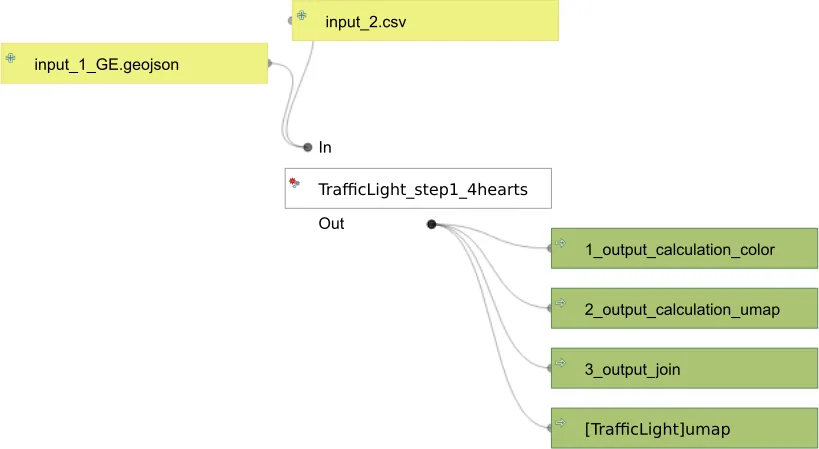
Workflow QGIS (MODEL3) for “part 2: batch processing–Traffic-Light.geojson for uMap” in step 1: geomatics without webmapping. See Multimedia Appendix 8.

R, RMarkdown, and Excel were used for all statistical computations (SMM_10000, DENSI, Khi2LnFr) and to produce the ready-to-use input files for Philcarto. R scripts and RMarkdown programs are available in the public repository. SQLite was used for structured storage and querying of intermediate mortality tables during data preparation. See also Multimedia Appendix 5 and Figures 3–5.

#### Overview of Methodologies

This methodological approach consisted of 3 main parts: data architecture, step 1: geomatics without webmapping, and step 2: geomatics with webmapping.

The first goal was to present the software architecture, which includes a description of the data processing stages (Figures 1 and 2), including the data transformation diagram (Figure 2B). In this study, data management was an essential process carried out in different software environments involving data collection, cleansing, and ordering processes [71]; specialized concepts; processes such as geomatic data processing pipelines; databases; and the extract, transform, and load (ETL) process. We schematized our methodological approaches to enable better monitoring of data pipelines in step 1: geomatics without webmapping or step 2: geomatics with webmapping.

For step 1: geomatics without webmapping, the data pipeline diagram for transforming KML into a GeoJSON file is shown in Figure 3, and the schematics for processing automation in QGIS are shown in Figures 4 and 5.

**Figure 6.**
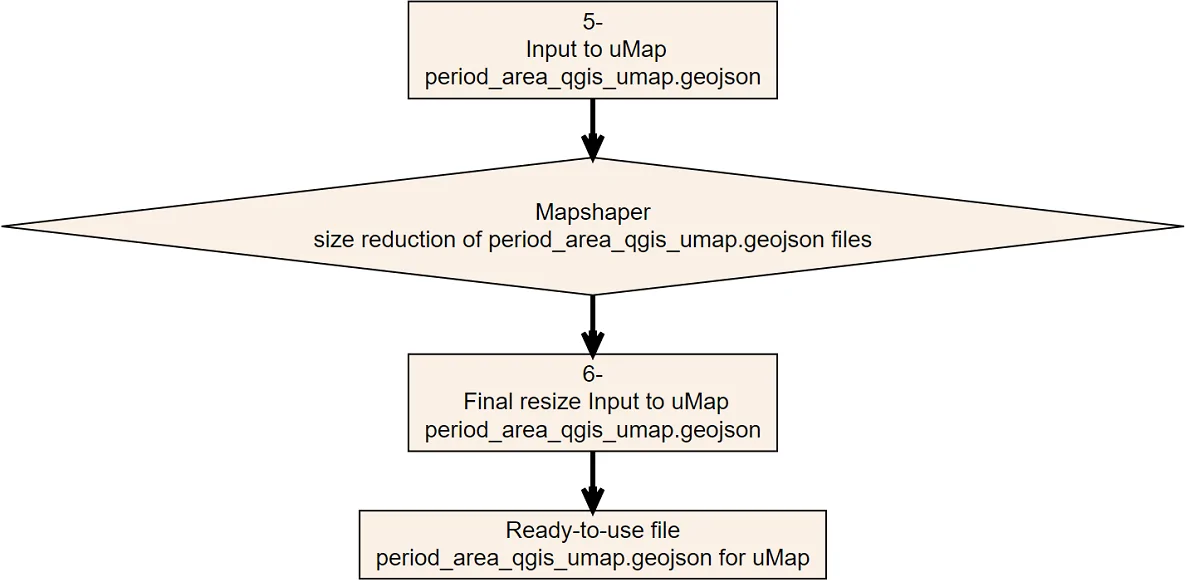
Data pipeline for the geomatics processing software system in step 2: geomatics with webmapping. There were 2 types of information: rectangle: input-output result; diamond: processing. See Multimedia Appendix 9.

**Figure 7.**
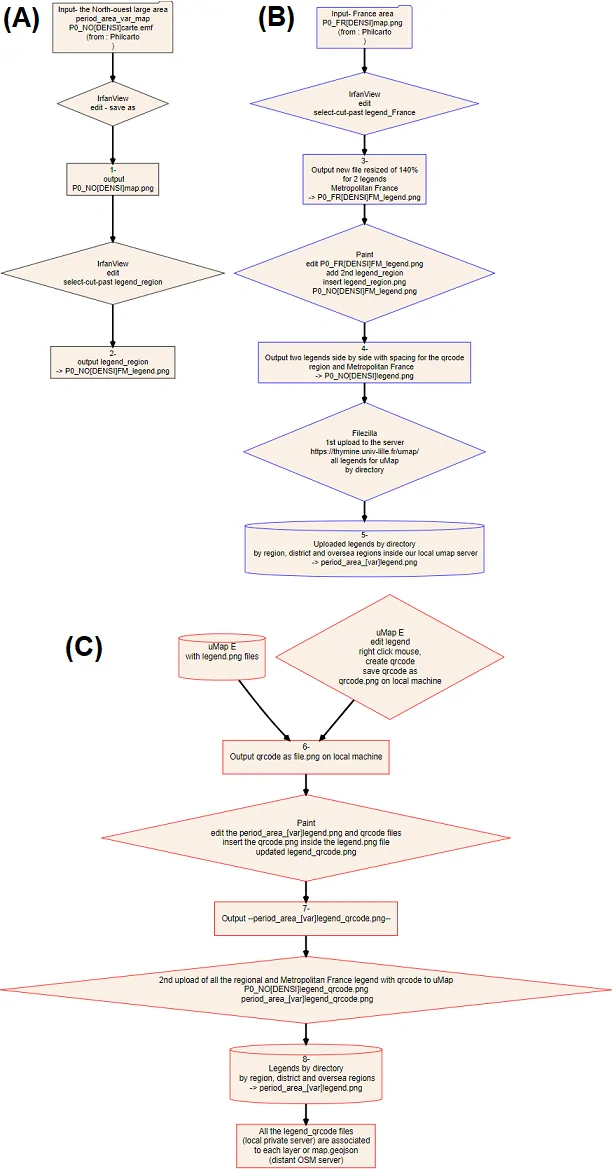
Double caption geomatic data processing pipeline in France and regions in step 2: uMap geomatics with webmapping, including the 3 blocks of procedure stages 1‐8: (A) stages 1‐2, (B) stages 3‐5, and (C) stages 6‐8. There were 3 types of information: rectangle: input-output; cylinder: database; diamond: software processing. See Multimedia Appendix 10[72].

**Figure 8.**
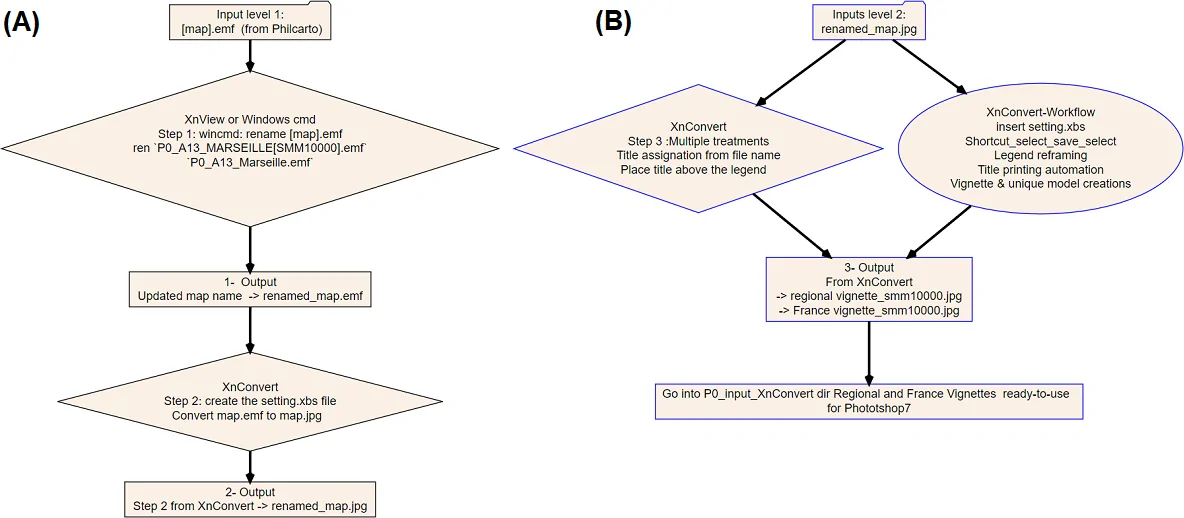
The 2 blocks of procedure stages 1‐3: (A) first workflow: Win cmd and XnViewMP and (B) second workflow: XnConvert_thumbnail processing for Photoshop7 in step 2: geomatics with webmapping. There were 3 types of information: rectangle: input-output; ellipse: workflow process; diamond: software processing. See Multimedia Appendix 11.

**Figure 9.**
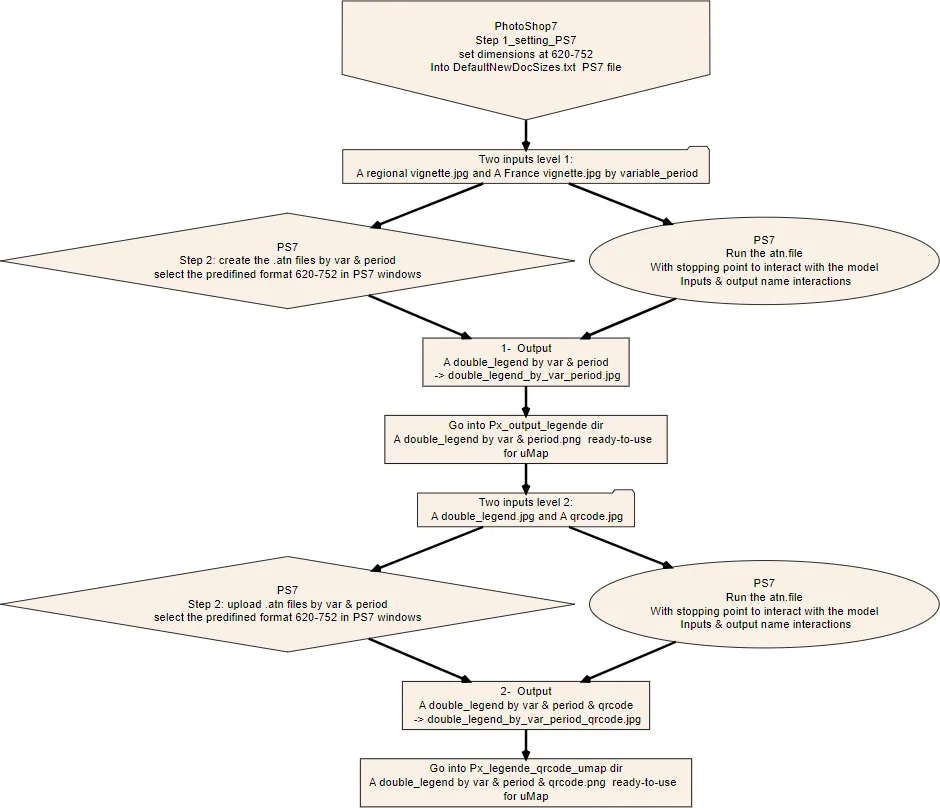
Photoshop7 workflow for legends with and without QR codes in step 2: geomatics with webmapping. There were 3 types of information: rectangle: input-output; ellipse: workflow process; diamond: software processing. See Multimedia Appendix 12. PS: Photoshop.

For step 2: geomatics with webmapping, the resizing processing schematic in Mapshaper is shown in Figure 6. The double caption processing diagram in the computer-aided design (CAD) software and uMap is shown in Figure 7, and the thumbnail processing diagram in the CAD software is shown in Figure 8. Finally, Figure 9 presents the Photoshop processing automation diagram.

### Methodologies

#### The Software Architecture: Geomatic Action Plan—Essential and Auxiliary Steps

See Figure 1 for the software architecture of the mortality atlas for uMap.

#### INSEE Death Data Engineering

All the data sources (Table 1) shared a common identifier: the 5-character INSEE municipality code (eg, 2A014). SQL was used to analyze and process the data in SQLite [25]. All the processing is automated except for one process that stays manual. A simplified data transformation diagram is shown in Figure 2.

The CSV data were imported into SQLite and combined to produce an “insee_death” table. An analysis of the fields (variables) indicated that days and months of birth were not systematically filled in. The age of each individual was calculated, except for those for whom the specific day or month of birth was not documented. The data were then aggregated by the INSEE municipal code, after which the following metrics were calculated: average age (excluding date of birth not provided), 2020 mortality, average mortality over the 2 years 2018 and 2019, and difference in mortality. The final stage in the process, as delineated in Figure 2, was the association of the death data with the reference 2017 municipal population. The database thus created, appointed “insee_death_pop,” was exported in CSV format. The reference structure of the data corresponding to the mortality variables is shown in Table 2 (see Multimedia Appendix 3 and Multimedia Appendix 4).

**Table 2.** Structure of the death database (source: French National Institute of Statistics and Economic Studies [INSEE]).

Variable	Description	Illustrative example
Group 1		
ID_mun	Municipality ID	26362
ID_dep	Departmental ID	26
ID_reg	Regional ID	121
Mun	Municipality name	Valence
Mun_pop_2017	Municipality population number—2017	63,714
Group 2		
Death_2018	Number of deaths in 2018	301
Death_2019	Number of deaths in 2019	295
Avg_death_2018‐2019	Average number of deaths between 2018 and 2019	298
Death_2020	Number of deaths in 2020	352
Mortality_diff	Difference of deaths between Death_2020 and Avg_death_2018‐2019	54
Avg_age	Average age at death	79
Group 3		
SMM_10000	Excess mortality or submortality per 10,000 inhabitants	8

#### INSEE Data Engineering in R and Excel for Philcarto

Several data preprocessing and statistical calculation stages were performed in R, Excel, and Philcarto. In the preprocessing stage (Table 3, line sections [1], [6], [11], [16], and [21]), the output file “insee_death_pop.csv” from Figure 2 needed additional preprocessing stages in R and Excel. These stages primarily entailed the incorporation of statistical variables and the addition and renaming of fields. The enriched death file (Table 3) could then be used in Philcarto and within the processing chain to instantiate atlases in uMap. Compared with the data presented in Table 2, the output file included modified, renamed, created, and reordered variables. The addition of suffixes to variable names enabled direct typing by Philcarto [34,35]. As illustrated in Table 3, the suffix _R_ appointed a report variable, whereas _N_ indicated a nominal variable. After the implementation of the R project program, the resulting XLSX output file had 24 variables.

**Table 3.** French National Institute of Statistics and Economic Studies (INSEE) variables transformed after processing in R and Excel.

Line	Variable names per line^a^
Group 1^b^	
[1]	ID; ID_1; ID_department; ID_municipality; municipality;
[6]	Population; Muni_population; Death_2018; Death_2019; Avg_death_2018_2019;
[11]	Death_2020; Excess mortality; Avg_age; SM_10000; SMM_10000_R_^c^;
[16]	status; Period; ID_2020_region; ID_2020_department; Department_2020;
[21]	Region_2020; Period_N_^d^; statut_N_^d^; Excess_mortality_R_^c^;
Group 2^e^	
a [25]	Death_2020_Theo_Fr; (Obs-Theo) ^2/Theo_Fr; Death_2020_Theo_region; (Obs-Theo) ^2/Theo_region;
a [29]	Khi2LnFr;
Group 3^f^	
b [25]	Death_2020_Theo_Fr; (Obs-Theo) ^2/Theo_Fr; DENSI; Khi2LnFr;
b [29]	Death_2020_Theo_region; (Obs-Theo) ^2/Theo_region;
Group 4^g^	
[31]	ID_region; Khi2LnFr; 10_classes; Class; Traffic-Light;
[36]	Class_1; Excess_mortality_Traffic-Light; Excess_mortality_Traffic-Light_umap;

a We wrote the khi-square variables “Khi2 and Khi2LnFr”.

b Group 1, lines [1] to [21]: 24 variables transformed from R.

c _R_: suffix for group 1 variables.

d _N_: suffix for group 1 variables.

e Group 2, lines a [25] to a [29]: variable numbers 25 to 29 transformed from Excel.

f Group 3, lines b [25] to b [29]: variable numbers 25 to 30 transformed for Philcarto.

g Group 4, lines [31] to [36]: variable numbers 31 to 38 transformed for the Traffic-Light map, workflows, and tool tips.

In the statistical calculation stage (Table 3, sections a [25] and a [29]), Excel spreadsheets were supplemented with additional statistical variables, namely “raw khi-square” and khi-square with the logarithmic transformation “Khi2LnFr.” The methods for calculating these variables are described in the *Computations of the Atlas Variables* section. As outlined in Table 3 (line sections b [25] and b [29]), specific variables may have undergone renaming to align with the Philcarto file header standard. For example, the ratio variable “(Obs - Theo) ^2/Theo_Fr,” previously designated “raw khi-square,” was then referred to as “DENSI.”

At the Traffic-Light classification stage (Table 3, line sections [31] and [36]), a new series of Excel processes was used to classify the values of the variable “Khi2LnFr” to generate the fourth variable of the Traffic-Light Atlas associated with excess mortality in 3 colors and to create subnational maps in Philcarto via the variable “Excess Mortality_Traffic-Light_umap.” At this stage, the input file “insee.xlsx” was enriched with information variables such as “Excess Mortality_Traffic-Light_umap,” which was directly recognized by the suffix “umap.” This variable was used to interactively display the municipal tool tips (on hover) of the uMap atlas instantiations.

At the Philcarto software processing stage (Table 3, line sections [31] and [36]), the following files were presented as inputs to the software: spreadsheet-insee.xlsx (France as a whole; 34,833 municipalities online) and vectorial basemap.shp (georeferenced shapefile for the region or France as a whole) [34,35]. As an output, we obtained a first cartography associated with the 4 atlas variables, designated “SMM_10000,” “DENSI,” “Khi2LnFr,” and “Traffic-Light.” The Traffic-Light Atlas variable was named “Excess Mortality_Traffic-Light.” The available output formats encompassed Adobe Illustrator (AI), Philcarto macro language (PMC), Enhanced Windows Metafile (EMF), and KML (see Table 1). The KML format was georeferenced with the World Geodetic System (WGS) 1984–EPSG_4326 [53-56,59]. The KML format eased interoperability and started the processing chain in other software environments, with the aim of obtaining a ready-to-use map in GeoJSON format for the “uMapOSM.fr” remote server (Figures 1–3). Choropleth maps, also referred to as color-coded thematic maps, were systematically accompanied by a discretized legend that uses the Jenks method (see the *Jenks Discretization* section) [73,74].

#### 2020 Basemap Data Engineering

Basemaps at the scales of the DROMs; large regions (grouped areas); and submunicipal districts of Lyon, Paris, and Marseille were created via IGN files (Table 1). The georeferencing process was executed in accordance with the WGS84 GPS standard [59], which culminated in the generation of a KML output map for use within CAD software (Table 1; Figures 1–3). The shapefile (SHP) files of France’s municipalities were divided into 4 major subnational municipality regions via the Éclats tool [35] due to limitations in vector file size. The basemap was augmented with one or more administrative boundary layers of greater thickness. The quantity of GeoJSON layers given by uMap was related to the number of choropleth maps displayed for each atlas. Finally, the Traffic-Light Atlas had a total of 12 GeoJSON layers, which included 4 large regions, 3 submunicipal districts, and 5 DROMs. For further information on map background engineering, please refer to the authors’ previous work [35,75].

#### Geomatic Data Engineering

To ensure comprehensibility, geomatic data-processing pipelines and workflows are presented in schematic form.

##### GeoJSON Creation Stage (Step 1: Geomatics Without Webmapping)

The “direct KML” file produced by Philcarto is an inaugural file subjected to geomatic processing chains (workflow) via a data pipeline (see the *Data Pipelines of the 2-Step Geomatic Action Plan* section). In a multitude of software packages, the execution of transformations on the “direct KML” results in the generation of a final output file in GeoJSON format (Figure 3). The workflow in QGIS was based on 2 input files, “GE_vscode.geojson*”* and “spreadsheet.csv,” and on the workflow model generated via the “modeler designer” tool, which generates files in MODEL3 format. These models ease batch processing. The output files were obtained from QGIS in the form of maps and enriched data in GeoJSON format and were ready for use in uMap (Figures 4 and 5). For example, the Excess Mortality_Traffic-Light_umap variable was automatically transformed to display structured tool tips when the mouse is moved over the Traffic-Light Atlas.

##### Legends Creation Stage (Step 2: Geomatics With Webmapping)

**Figure 10.**
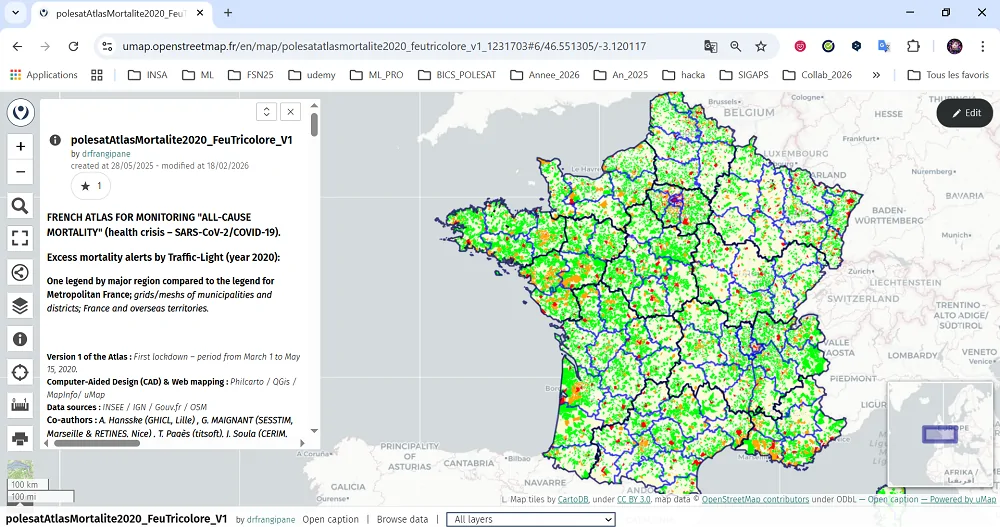
The uMap atlas generated by Traffic-Light, France (see [6]).

**Figure 11.**
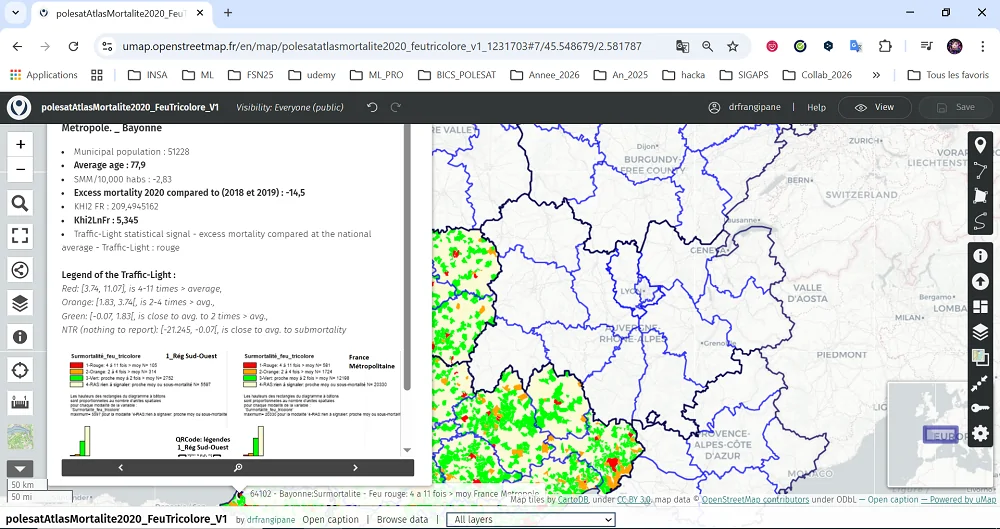
The uMap atlas generated by Traffic-Light, with an enlarged view of the southwestern region of Bayonne town featuring tool tips (see [6,78]).

The GeoJSON map, which was obtained from processing the KML format (step 1: geomatics without webmapping; Figures 3–6), was uploaded as an input file to the French uMap OSM-fr remote server. After its implementation, the GeoJSON map was managed via the uMap “layer window” manager and “dashboard” (Figures 10 and 11). The legend associated with each GeoJSON layer was displayed via the legend creation and formatting data pipeline shown in Figure 7. The workflow for creating caption thumbnails is illustrated in Figure 8, whereas the workflow for formatting captions (automated in Photoshop using “script. ATN”) is shown in Figure 9. Legend files in the PNG or JPG format were uploaded to a remote server at the University of Lille, as these formats cannot be hosted on the uMap OSM-fr server, which is reserved exclusively for GeoJSON files [76,77] (Figure 11).

### Methodologies for Step 1: Geomatics Without Webmapping

#### Choice of Scales and Meshes

A nationwide French analysis was conducted for the mortality atlases under consideration, including DROMs, within a fine territorial grid of municipalities or submunicipal districts in Lyon, Marseille, and Paris. This approach supplemented existing studies that primarily used information from coarser grids [16].

#### Computations of the Atlas Variables

Statistical calculations were performed with death data from INSEE to create 4 atlases (see Figures 1 and 2 as well as *The Software Architecture: Geomatic Action Plan—Essential and Auxiliary Steps* section) based on the following 4 variables: (1) “SMM_10000” variable relating to excess mortality and submortality, (2) “DENSI” variable relating to a raw Khi-Square (Raw Khi2), (3) “Khi2LnFr” variable relating to “Khi-Square Ln Fr”, and (4) “Traffic-Light” variable.

##### “SMM_10000” Variable Relating to Excess Mortality and Submortality

The formula used to calculate this variable is as follows:


$$SMM\_10000=\left[\frac{Death_{2020}-Death_{2018_{2019}}}{Municipal_{Pop}}\right]*10,000$$

This variable is calculated via municipal death data from all causes from 2018 through 2020. The numerator is the difference between the number of deaths in 2020 and the average number of deaths in 2018 and 2019 divided by the municipal population in 2017. This ratio is then multiplied by 10,000. The resulting variable shows the excess mortality or submortality rate per 10,000 inhabitants.

##### “DENSI” Variable Relating to a Raw Khi-Square (Raw Khi2)

The formulas used to calculate this variable are as follows:


$$Death_{2020theoriticalFR}=National\_Death\_Frequency*Municipal\_Pop$$

$$\begin{array}{rl} \text{National\_Death\_frequency} & =\frac{\sum {\text{Death}}_{2020}}{\sum {\text{Municipal}}_{Pop}}\\ =\frac{151,162}{64,639,133}=0.00233855 \end{array}$$

$$DENSI={\left[\frac{{\left(Obs-Theo\right)}^{2}}{Theo}\right]}_{FR}=\frac{{\left(Death\_2020-Death_{2020theoricalFR}\right)}^{2}}{Death_{2020theoricalFR}}$$

The density of deaths was calculated as the ratio of the number of national deaths to the total French municipal population (metropolitan or excluding the DROMs [~64 million]). This ratio was then brought down to the municipal population, thereby allowing for comparisons between observed and theoretical deaths. Consequently, we emphasized excess mortality or submortality, which are more relevant and significant in terms of probability than the national average is.

As a sensitivity check, we assumed a Poisson model for municipal deaths with *E_i_* parameter (based on the 2018‐2019 baseline). Under this assumption, the DENSI corresponded to the classic Pearson khi-square statistic calculated for each municipality based on observed and expected deaths and normalized by the French national reference. DENSI was formally defined as the sum of the squares of the differences between observed and expected deaths for each municipality divided by expected deaths.

##### “Khi2LnFr” Variable Relating to a “Khi-Square Ln Fr”

The formula used to calculate this variable is as follows:


$$X^{2}=Khi2LnFr=Ln(DENSI)$$

The “DENSI” variable is transformed using a logarithmic scale to show excess mortality or submortality compared with the national average.

For each municipality, the life expectancy at death was calculated based on the 2018‐2019 average over the same period. The DENSI variable was the standard term for Pearson khi-square, and Khi2LnFr was its log-transformed version used for cartographic discretization. This discretization method compressed extreme values while preserving the order of municipalities. This transformation facilitated cartographic classification into ordinal classes used for choropleth (Traffic-Light) map representation.

##### “Traffic-Light” Variable

The excess mortality values of the Khi2LnFr variable were grouped into 3 color classes (green, orange, and red) via the Jenks discretization method in Philcarto. This Traffic-Light–inspired semantic approach provides humans with easily understandable statistical information. Municipalities in the red Traffic-Light class were almost systematically above the upper 95% Poisson bound, whereas pale yellow municipalities fell within or below this bound, supporting the consistency of our classification with a simple count-based model.

### Jenks Discretization

The map legend was created via statistical discretization via the Jenks method (the natural threshold method). This method maximizes the variance between classes and minimizes the variation within classes, making each group of geographical units more homogeneous than other methods. Computer calculations can be time-consuming, but the results are optimal. Maps are objective if >5 classes exist. This method is better suited to multimodal variables with a large enough individual to create thresholds. It was developed by GF Jenks, an American cartographer and geographer, in the 1960s [79,80].

### 10-Class Classification

#### Overview

The Jenks method was used to create a map of the whole country for each variable, with a legend of 10 classes. The boundary values of the 10 classes were stored in a repository and used to create thematic maps of the same variable on a subnational scale, including 4 geographical areas (northeast, northwest, southeast, and southwest), administrative regions, submunicipal districts, and DROMs.

#### Example of a 10-Class Frame of Reference

##### Khi2LnFr and Traffic-Light Variables

Table 4 presents the variables Khi2LnFr and Traffic-Light. The first variable had 10 classes, with the classes corresponding to excess mortality compared with the national average being associated with the 3 colors of the Traffic-Light variable. A change from green to red indicated an increase in excess mortality.

**Table 4.** 10-class classification scheme based on Jenks discretization (variable: Khi2LnFr).

Class number	Khi2LnFr interval	Municipalities, n (%)^a^	Class color	Poisson *P*<.05, n (%)^b^^,^^c^	Interpretation^d^
Traffic-Light colors					
10	[+3.74 to +11.07]	581 (1.7)	Red^e^	552 (95.01)	High statistical signal
9	[+1.83 to +3.74[	1724 (5)	Orange^f^	642 (37.24)	Moderate statistical signal
8	[+0.75 to +1.83[	4629 (35)^g^	Green^n^	157 (3.39)	Low statistical signal
7	[–0.07 to +0.75[	7569 (35)^g^	Green^i^	0 (0)	Near-average, minimal signal
Outside Traffic-Light colors					
6	[–0.84 to –0.07[	8409 (58.3)^j^	Pale yellow^k^	—^l^	NTR^m^: close to average to submortality
5	[–1.71 to –0.84[	7070 (58.3)^j^	Pale yellow ^k^	—	NTR: submortality
4	[–3.06 to –1.71[	3466 (58.3)^j^	Pale yellow^k^	—	NTR: submortality
3	[–5.22 to –3.06[	976 (58.3)^j^	Pale yellow^k^	—	NTR: submortality
2	[–8.78 to –5.22[	340 (58.3)^j^	Pale yellow^k^	—	NTR: submortality
1	[–21.24 to –8.78[	69 (58.3)^j^	Pale yellow^k^	—	NTR: submortality

a Municipalities identified in France as a whole: n=34,833 (6 depopulated municipalities with population=0 were excluded from the Poisson calculations).

b For each municipality without a filter, a 1-sided exact Poisson test (right-tailed) was computed: *P*=P(X≥O_i_|λ=E_i_); Mathematical definition for class k: %=100×|{i ∈ class_k_: *P*<.05}|/N_k_, where N_k_=total number of municipalities in class k (denominator=ALL N_k_, regardless of E)*. P*=scipy.stats.poisson.sf (Oᵢ−1, Eᵢ), strict inequality *P*<.05. *P*=P(X≥Oᵢ | λ=Eᵢ)=1−F(Oᵢ−1; Eᵢ), where F is the cumulative distribution function (CDF) of Poisson. The value represents the percentage of municipalities within each Traffic-Light class whose observed excess mortality was statistically incompatible with the random Poisson variation around the 2018‐2019 national baseline at the 5% significance level. For example, 95.01% (552/581) of the red class municipalities individually reached statistical significance at the 5% level, confirming internal coherence of the upper Jenks boundary (Khi2LnFr=+3.74); in other words, a low *P* value (eg, <.05) suggests significant excess mortality in that municipality (see Multimedia Appendix 13).

c No filter on Eᵢ (Eᵢ=expected number of deaths).

d Probability that the observed excess mortality is incompatible with random Poisson variation around the national baseline (2018‐2019); gradient from 3.39% (green) to 95.01% (red) confirmed the internal coherence of the Jenks-based Traffic-Light classification.

e Highest excess mortality signal (see the *Ordinal Interpretation of the Classification* section).

f Moderate excess mortality signal (see the *Ordinal Interpretation of the Classification* section).

g Class numbers 7 and 8 were combined for the percentage of municipalities: n=12,198.

h Low excess mortality signal (see the *Ordinal Interpretation of the Classification* section).

i Near-average to minimal excess mortality signal (see the *Ordinal Interpretation of the Classification* section).

j Class numbers 1 through 6 were combined for the percentage of municipalities: n=20,330.

k No Traffic-Light response; near-average to submortality (see the *Ordinal Interpretation of the Classification* section).

l Not applicable.

m NTR: nothing to report.

##### Internal Coherence of the Classification

The Jenks natural breaks algorithm was applied to the distribution of Khi2LnFr across 34,833 French municipalities, identifying 10 natural classes subsequently merged into 3 Traffic-Light signal bands and 1 “nothing to report (NTR)” category (Table 4). As an internal calibration step of the geomatics pipeline—consistent with the scope of this medical informatics article—a 1-sided exact Poisson test (right-tailed) was computed for each municipality “without a filter” on expected death *E*_*i*_: *P*=P(X≥O_i_|λ=E_i_). The results confirmed a clear, class-dependent gradient of statistical significance: Only 3.39% of green class municipalities reached *P*<.05, compared with 37.24% for orange and 95.01% for red (Table 4). This gradient validated the internal coherence of the classification.

##### Ordinal Interpretation of the Classification

Excess mortality classes (green, orange, red) were defined by intervals based on Khi2LnFr, so they were ordinal bands derived from the national distribution using the Jenks method, with “interpretations” (Table 4) such as “green class: near-average, minimal signal or low statistical signal,” “orange class: moderate statistical signal,” and “red class: high statistical signal” or by using this second example of “verbal labels” such as “~2–11× above average,” indicating approximate orders of magnitude of the observed/expected (O/E) ratio without exact multiplicative values due to the use of logarithms; the rationale is presented in Multimedia Appendix 13. Consequently, the primary information conveyed by this second verbal label—“~2‐11× above average” classification—lies in the relative ranking of units along the excess mortality gradient rather than in the precise numerical value of the O/E ratio. This ordinal approach was intentionally designed to emphasize comparative positioning and spatial contrasts within the national context while limiting overinterpretation of absolute magnitudes that are sensitive to stochastic variability and model assumptions.

### Data Pipelines of the 2-Step Geomatic Action Plan

To determine the “environment of the atlas” in uMap, a 2-step geomatic action plan was used. In step 1: geomatics without webmapping, maps were constructed in GeoJSON format outside of uMap (Figures 3–5). In step 2: geomatics with webmapping, legends were created with uMap (Figures 6–9).

#### Step 1: Geomatics Without Webmapping

##### Data Pipeline Description

A variety of software and processing methods were used to generate instances of the Traffic-Light Atlas in uMap. To obtain files in KML format and subsequently in GeoJSON format ready for uMap, we processed the information through a software suite producing a data pipeline [32]. The data pipeline was complex. The schematic in Figure 3 shows the tasks organized sequentially in different software programs, with the main packages being CAD, GIS, Globe, source code editors, and spreadsheets (see Table 1).

###### KML to GeoJSON for the uMap Data Pipeline Diagram

As demonstrated in Figure 3, the maps in the KML file format were transformed to obtain the final GeoJSON format. The KML map, therefore, underwent a series of processes in a pipeline of software environments (Google Earth, VS Code, and QGIS; the GeoJSON file editor) until the final “map.geojson” file was obtained at the conclusion of step 1 (see Figures 1 and 2).

In Figure 3, a distinct shape is shown for each type of information: rectangle: input‒output result; rhombus or diamond: software processing; and ellipse: QGIS workflow modeler.

The pipeline was sometimes partially automated and sometimes fully automated, as in QGIS workflows (Figures 4 and 5) [32]. The task processing chain was executed within the same software environment. In this particular instance, the “modeler designer” was used [62] to develop a model that automated an entire geomatic processing chain. The model began with an “input” and culminated in the generation of a GeoJSON file (for uMap). Two files were created via the modeler designer (MODEL3) to create a map of France: “file: processing chain” and “file: batch processing” (see Figures 1 and 2).

###### QGIS Workflow Diagrams

In the QGIS, the workflow was divided into 2 parts, as shown in Figures 4 and 5, allowing processing of the direct KML file to obtain the GeoJSON file for uMap.

### Step 2: Geomatics With Webmapping in uMap

After following step 1: geomatics without webmapping, the final “map.geojson” file was ready for use as an “input file” in step 2 [12,13].

The GeoJSON file obtained in step 1 may not be imported in its original state because of size constraints imposed by the OSM server. In this case, we used the Mapshaper tool to reduce the vector shape definition [36,81] and, consequently, the file size.

#### Reduced GeoJSON Resolution Workflow for uMap Imports

In Figure 6, a distinct shape is associated with each of the 2 types of information: diamond: processing and rectangle: input‒output result. See Multimedia Appendix 9.

#### uMap Platform Features

Once the GeoJSON file had been imported into the remote OSM server of uMap, to address the complexity of the processing in step 2, the data pipeline was diagrammed (Figure 7; see Figures 1 and 2 for a reminder).

##### In the uMap Environment

The uMap dashboard environment is distinguished by a relative paucity of lines of code for control. The map display is configured through the dashboard user interface. Upon initiation of the URL in the browser, uMap continues to load each file about the major regions, submunicipal districts, and DROMs in succession. The final display of the Traffic-Light Atlas can be viewed at the scale of the entire French territory. A map setting template was created using the first dashboard settings for the Traffic-Light Atlas (eg, map display parameters such as titles, fonts, and legends). This template automates the integration and display of atlases and their maps in GeoJSON format in uMap according to a variable, which saves time. The open data layers provide administrative and sanitary boundary lines.

##### Different Servers for Hosting Maps and Legends

These data were downloaded in GeoJSON format to the remote OSM server of uMap France and managed via the uMap layer manager (see Table 1 and Multimedia Appendix 5). Hosting the legends associated with each GeoJSON map of France’s major regions, submunicipal districts, and DROMs required access to a new server because the JPEG and PNG legends cannot be uploaded to the same OSM remote server as the Atlas GeoJSON files. The uMap atlas was secure from creation; it was simply set to secure. Four OSM administrators manage the OSM remote server security, and 3 servers are hosted by OVH Cloud France.

These numbers may have changed since the growth of uMap in 2023 with its new uMap public service platform and government server. The map data in the GeoJSON files are not encrypted on the server. The servers are not compliant with the General Data Protection Regulation (GDPR) [82]. The minor weaknesses of the uMap environment described in the literature [11,83] are not concerns.

### Geomatic Data Processing Pipeline Description

To address the complexity of geomatic processing for atlas embellishment in uMap, we schematized the data pipeline to create and associate a double legend for each GeoJSON map.

#### Double Caption Data Pipeline Diagrams

Figure 7 includes 3 blocks of procedures numbered 1 through 8. We started with a Philcarto file in EMF format. At the end of the chain, a double map legend was shown with a QR code in PNG format. These legends are on a remote server for univ-lille.fr and are associated with each GeoJSON map on uMap OSM-fr.

#### Thumbnail Workflow Diagrams—Preparation of Legends for Step 2: uMap Geomatics With Webmapping

For more details about the 2 blocks of procedure stages 1-3, see Figure 8.

#### Workflow Diagram in Photoshop—Double Legends With and Without QR Codes—Step 2: Geomatics With Webmapping

For more details about the Photoshop7 workflow legends with and without QR codes, see Figure 9.

### Ethical Considerations

This study did not use any studies with human participants or animals performed by any of the authors. Since the mortality data from the INSEE are public, we were exempt from any request for authorization from an ethics committee. Please refer to the terms of use for INSEE data in [84] and the copyright for INSEE data in [85]. The first part of the copyright notice for the INSEE data says “The publications and data made available on this website are accessible and downloadable free of charge. Unless otherwise specified, they may be reused for commercial purposes, without a license and without the payment of royalties other than those collected by copyright collection and distribution societies governed by Title II of Book III of the Intellectual Property Code. However, reuse is contingent upon maintaining the integrity of the information and data and providing precise citations of sources…” [69].

## Results

### Overview

This section focuses on the results of the Traffic-Light Atlas. First, we examined the results of the Traffic-Light map in Philcarto during step 1: geomatics without webmapping. We then examined the results of the Traffic-Light Atlas in uMap during step 2: geomatics with webmapping.

In step 1, the initial Philcarto KML map file was processed with VS Code, Google Earth, QGIS, Geojson.io, and Mapshaper, and the spreadsheet data were enriched. In step 2, the atlas was enriched by 2 processing chains prior to obtaining the results of the atlas with its double legends in the uMap environment.

Since the map’s visual aspects remained unchanged throughout step 1, it was unnecessary to display it in intermediate environments until the final GeoJSON file was obtained.

### Step 1: Geomatics Without Webmapping—Traffic-Light Choropleth Map Under Philcarto

**Figure 12.**
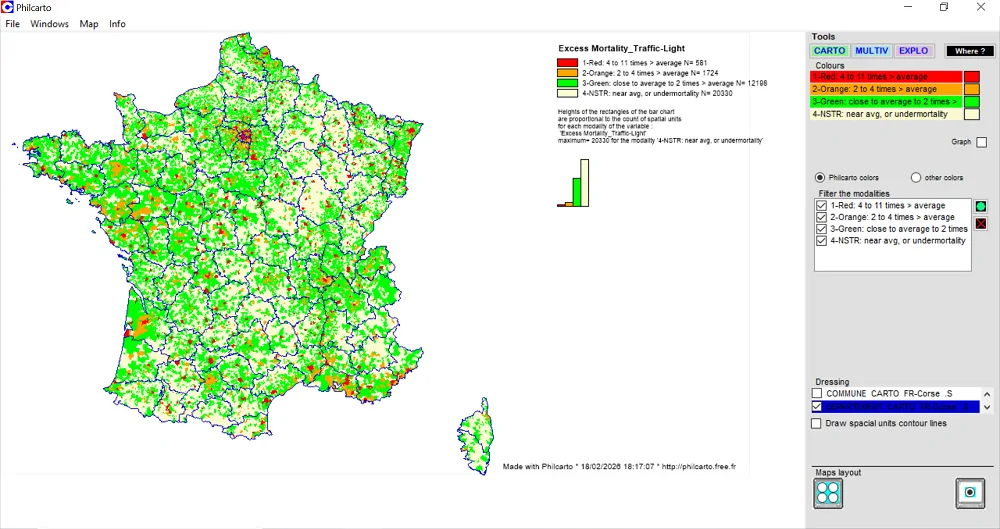
Map from Philcarto: excess mortality based on the Traffic-Light color system and submortality outside the Traffic-Light system (pale yellow); KML-Direct output format.

The cartographic results in the Philcarto environment enabled us to obtain the first exported map in KML format (Figures 1 and 2). This Philcarto environment consisted of a 2-part window: the map result and its single legend on the left and the dashboard for setting map creation on the right (Figure 12).

#### Interpreting the Traffic-Light Choropleth Map

Figure 12 shows a choropleth map accompanied by its corresponding single legend for the Traffic-Light variable. The map was augmented with a departmental boundary and presented on a French scale. The first lockdown linked to the COVID-19 crisis (March 1, 2020, through May 15, 2020) was covered.

Based on the Khi2LnFr variable, our statistical indicators were transformed into classes in the legend titled “Excess Mortality Traffic-Light.” The variable “Traffic-Light” represented the increasing excess mortality distributed across the 3 color classes. A fourth pale yellow color class was added to denote values that fall outside the Traffic-Light semantic range. The values in question were proximate to the mortality average or less than the average, thus indicating submortality. A total of 34,833 geographical units were incorporated into the map, excluding 6 municipalities whose population was determined to be zero. At the time of the study, the total population of France was 63,639,133. In total, 20,330 municipalities (20,330/34,833, 58.3%) were classified as outside the 3 Traffic-Light colors, and 14,503 municipalities (14,503/34,833, 41.7%) were assigned one of the Traffic-Light colors corresponding to excess mortality.

#### Details of the Traffic-Light Classification of the 14,503 Municipalities

A total of 12,198 municipalities (12,198/34,833, 35%) belonged to the green class, in the range from “close to the average to 2 times above the average.”

A total of 1724 towns (1724/34,833, 5%) belonged to the orange class, in the range “2-4 times above the average.”

A total of 581 towns (581/34,833, 1.7%) belonged to the red class, in the range “4–11 times above the average,” which represented an extremely significant excess mortality rate.

An assessment based on a simple Poisson model confirmed the consistency of the red class with the expected 95% quantile exceedance, validating the robustness of the classification.

Below the classes in the Excess mortality_Traffic-Light legend in Figure 12 is a bar chart proportional to the number of spatial units (34,833) included in each modality of the variable. From left to right, the heights of the rectangles are 581 (1.7%), 1724 (5%), 12,198 (35%), and 20,330 (58.3%).

The map aims to signal to the observer immediately (visually) and simplify interpretation for the public via familiar semantics.

### Step 2: Geomatics With Webmapping—Traffic-Light Atlas Under uMap

The results obtained in step 1 were then used and incorporated into the processing chain in step 2. This section presents the uMap functionalities, the Traffic-Light Atlas results for all of France, and a zoom-in on the southwest region with a tool tip pointing to Bayonne.

#### Ergonomics and Usability of the uMap

The uMap showcase includes examples of our atlases, which cover all of France and include DROMs. Three URLs provide access to these atlases [6,17,18]. There were as many double legends as there are imported GeoJSON layers. Atlas access and display times were relatively short: Regarding average page load times, the time measured for the first display (including layer toggles) was 1.5 seconds, and the full page load time was 5 seconds. However, loading into uMap was slower because of the display of the complete atlas, which loads GeoJSON layers at the municipality level for the major regions, submunicipal districts, and DROMs, representing 34,833 municipalities. Once the atlas was loaded, the navigation experience in the browser was smooth and without slowdowns. The dashboard allowed one to select and configure the desired display features, including tool tips.

From the uMap user interface, several functions and icons were accessible: zoom in and zoom out; search for place names; take a full-screen view; center the map on a location; measure distances; and select the desired “tile layer” basemap from those provided by the OSM, IGN, or National Aeronautics and Space Administration. The user guides for uMap are available at [6,14,50,82,83,86-88]. Printing atlases requires 2 stages: accessing the data sharing functionality and retrieving exported map data in GPX and KML formats (for printing purposes, see also other GIS or web services such as MyOSMatic [89]). Finally, uMap is an interoperable platform for exporting and printing maps on the basis of the API [11].

#### Traffic-Light Atlas OSM World Basemap Based

The uMap environment for the uMap atlas generated by Traffic-Light, France (Figure 10) consists of a 3-part window.

The first part combines the visual aspect of the France atlas with several tool buttons on the left of the screen. The main tool button manages and displays layers (GeoJSON maps) and searches for data via the “Browse data” link.

The second part of the screen, on the right, contains the legend tab showing double legends for each layer.

The third part of the user interface displays a banner with the map title, the designer’s name, and information about the ownership of the uMap environment [87].

Figure 10 shows the Traffic-Light Atlas from the first French lockdown. It includes departmental and regional outlines, as well as labels for the GeoJSON layers associated with the 4 subnational areas: northeast, northwest, southeast, and southwest. The atlas uses colors on the map to display excess mortality. Double legends allow comparisons between subnational and national distributions. Figure 11 provides additional information about the uMap atlas generated by Traffic-Light, by featuring a tool tip focusing on an enlarged view of the southwestern region of Bayonne town.

Two processing streams were generated for the uMap atlas generated by Traffic-Light, France (Figure 10). The first stream reduced the file size via Mapshaper to import the GeoJSON files to the remote OSM server uMap, whereas the second added a double legend (via a geomatic pipeline) to improve the visualization and statistical interpretation of the atlas.

The following URLs direct the reader to the atlases of the “SMM_10000” variable [17] and the “DENSI” variable [18].

## Discussion

### Principal Findings

In this study, we demonstrated that the uMap webmapping platform can display choropleth maps accompanied by their double legends. Version 1 of the resulting mortality atlases applies to the period of the first lockdown in 2020. Owing to the Traffic-Light variable, we have shown that color semantics applied at the scale of France allow users to assess at a glance where excess mortality is highest in municipalities. This could encourage 2 things among uMap users (professionals and the public), which would therefore confirm our hypotheses. First, they may be motivated to explore our retrospective mortality atlases further to determine whether their municipality experienced excess mortality during the crisis (we present 2 concrete and hypothetical examples illustrating the practical utility of our atlases designed for a regional health agency). Second, our atlases have the potential to motivate uMap users to generate new thematic choropleth atlases in local or remote uMap servers.

In the *Discussion* section, we review the 2-step geomatic action plan, comprising step 1 (without webmapping) and step 2 (with webmapping). Step 1 involved the execution of the data engineering workflow, which encompassed activities such as data collection, cleansing, and planning (or scheduling tasks) related to death data. The subsequent step 2 involved processing chains and optimization for the creation of double legends.

### INSEE Death Data Engineering

We made 3 selections from the death datasets of INSEE.

Our first reference was the 2017 census population, which comprises data from aggregate data from 2015 through 2019. The explanation is as follows: Communal censuses are conducted annually on one-fifth of the municipalities with fewer than 10,000 inhabitants and on a yearly basis via a survey of a sample of 8% of the population in municipalities with more than 10,000 inhabitants [90]. The reference population for a given year N includes data from years N–2 to N+2. Finally, the INSEE reports an error margin of less than 0.02% for the survey method.

Second, the municipal population was chosen for the study. The explanation is as follows: There are 2 types of population censuses: (1) “municipal population” and (2) “population counted separately,” including second homes and students [91]. These censuses allowed data from one year to the next to be compared without double counting.

Third, we approximated the average age of death when there were missing values. The explanation is as follows: Some days or months after birth were missing (10,435/1,477,067, 0.7%). This average age could be refined by defining the birth month and day (the 6th month of the year and the 15th day of the month). Our approximation was acceptable for this study given the low percentage of incomplete data.

### Database Management Methods in the RStudio Environment

The recent “Cartography” package for R, renamed “mapsf” [73], has great potential because it makes it possible to reproduce step 1 from the input to the output files in GeoJSON format. If the initial step 1 can be replicated exclusively in the RStudio environment, there are many benefits, such as program corrections and one-click process execution. RMarkdown functions as an integrated module in RStudio, enabling users to follow the logic of at least part of the step 1 processing chain. Multimedia Appendix 4 contains a program for obtaining a ready-to-use file for Philcarto. See also Multimedia Appendix 2, Figures 1 and 2, and Table 2.

### uMap Webmapping Environment

We chose an open-source software package that is powerful, easy to use, and compatible with other tools because of the interoperability of the software’s input and output file formats. uMap downloads data in UMAP (uMap file format), GeoJSON (OSM proprietary), and KML (Google proprietary, standardized by the Open Geospatial Consortium [OGC] in 2008) formats [11-13,18,54]. It is evident that uMap is an ethical environment because the reuse of GeoJSON data is contingent upon the activation of sharing rights [83]. The uMap application is predicated on the Django framework [14,92].

The uMap application is distinguished by its simplicity and intuitiveness. Our experience has not revealed any significant issues associated with the platform [11]. uMap features a dashboard that facilitates the management and display of available layers and resources, including world map-type maps [82,87,88,93,94]. uMap is equipped with a number of features, including a slideshow [88], an icon for sharing with 8 export options [59,95], and dynamic updating of the GeoJSON file option [96]. Additionally, the uMap package provides a Python and JavaScript-based API [14] that facilitates the local installation of the uMap server, a feature that is particularly advantageous for a local server dedicated exclusively to health care.

The uMap France remote server hosts a global collection of shared maps with a variety of themes and use cases (eg, sports routes, itineraries with hotspot symbolism). A query conducted 4 years ago revealed the absence of shared choropleth maps when both “health” and “mortality” were entered into the uMap France server [97].

A dynamic community of developers and geomatics specialists maintains the software package. Since May 2023, this community has been reinforced by funding from data.gouv.fr [22] for the development of “uMap of public service,” a government platform [12,13,15,46,50,98,99]. Over the past 2 years, uMap has incorporated several new functionalities, including the following: (1) the ability to tag maps for quick retrieval on the server via a tag list, (2) the ability to create choropleth maps with a basic legend, and (3) a collaborative mapping environment.

### 2-Step Geomatic Action Plan for the uMap Environment

#### Overview

Maps in the humanities and social sciences are the result of a process that is sometimes complex and involves a considerable amount of software (or system) and file formats with limited automation. This issue was identified in our data processing activities. The time required to generate atlases in uMap is significantly impacted by our complex, noninterconnected, geomatic data-processing pipelines. However, even in the absence of interconnection between systems, software programs themselves generate file formats that enable interoperability when they are transitioning between software environments [99].

#### Critical Analysis of the 2-Step Geomatic Action Plan: With and Without Webmapping

Despite the problems highlighted, the results obtained through the implementation of the 2-step geomatic action plan demonstrate the efficacy of our approach, which uses licensed open-source software, free software, and licensed open data, as well as environments and functionalities tailored to our needs. These elements have enabled the full automation of certain tasks. The 4 weaknesses of the 2-step geomatic action plan are as follows:

1. Old versions of proprietary software hinder task automation. Our old Adobe Photoshop 7.0 is an example [41].
2. The data pipeline is not automatically interconnected due to a lack of geomatic functionality [99].
3. The dashboard currently lacks the ability to manage and create double legends for the GeoJSON layer: that is, a map devoid of a legend. We have requested this feature from the uMap team [100].
4. A major disadvantage is that many GeoJSON maps must be produced to instantiate the atlases (by variable).

#### Design of the uMap

##### Design Benefits

uMap is licensed under a permissive free software license, meaning that it is free of redistribution and modification [48]. We are not responsible for the security of our GeoJSON files on the remote server, which is managed by the OSM. This approach is essential for atlas instantiation. We created the Traffic-Light Atlas, which is considered the best way to signal excess mortality in municipalities and districts, outperforming other atlases such as the “SMM_10000,” “DENSI,” and “Khi2LnFr” variables. The atlas of the SMM_10000 variable functions as an indicator of submortality or excess mortality per 10,000 inhabitants, highlighting disparities in smaller towns that constitute most French territories.

##### Design Limitations

The geomatic processing chains necessary to obtain ready-to-use GeoJSON layers are cumbersome, complex, and not currently reproducible in a single environment. The main problem is a pipeline system of noninterconnected data, which requires many tools and workflows (only two are fully automated). We call attention to sections of Figures 3 and 4.

At present, there are no new features that facilitate the linking of legend image files to GeoJSON files.

The image format double legends must now be hosted on an additional remote server, as uMap no longer allows them to be uploaded to the OSM remote server.

### Uses of the uMap Collaborative Platform

#### Benefits of Using

uMap, a geomatic environment for collaborative map sharing, has been diverted as a medium for our choropleth maps and double legends representing the mortality atlases [11]. The nationwide Traffic-Light Atlas displayed in uMap has been deemed superior to the regional display (the online second environment [7]), facilitating instant interpretation of excess mortality statistical signals. The use of a municipal grid enhances the appeal of the atlas, allowing users to analyze specific municipalities. Preliminary observations suggest that no technical difficulties have been encountered when displaying the atlases through different web browsers. Building a parameter template via map cloning has been successful.

#### Main Limitations of Using

It is not possible to group atlases within a single uMap URL. New uMap features and updates since 2023 have caused errors in accessing our atlases. This issue was reported on OSM and GitHub [11-13] and was rapidly resolved.

### Step 2: Workflow Optimization for Double Legends

#### Strengths

We optimized workflows for GeoJSON layer creation in QGIS step 1 and double-legend creation and management in step 2. This section discusses the optimization of the double legends and the limitations encountered.

The Adobe Photoshop actions file format (ATN) script streamlined geomatic processing for double legends in Adobe Photoshop7 through macro recordings. This semiautomated process accelerated captioning and avoided manual errors. Captions were satisfactory, with or without QR codes. Ideally, newer Photoshop versions or nonproprietary free software would be used.

#### Photoshop Limitations

Batch processing can further optimize ATN scripts but is currently not possible in Photoshop7 because of persistent bugs. Incomplete macro recording with mouse or human-machine interaction limits the use of current ATN files. Editing ATN scripts in XML via JavaScript utilities on a Windows XP virtual machine may resolve this issue [101].

### Proliferation of Geomatics Tools, Languages, and Tips

Rapid software evolution in IT and geomatics demands continuous organic (dynamic) monitoring throughout the project [63]. All CAD software used in geomatics, as well as webmapping environments and related platforms, requires substantial investment supported by numerous user guides, instructional videos [75,102], dedicated geomatics forums, and IT expert exchanges. These systems integrate programming languages such as Python, scripting languages such as Scheme [64], and macro languages such as Visual Basic for Applications and Philcarto. We used basic functions in geomatic environments and specific tools or plugins. QGIS automation used the modeler designer tool and its associated MODEL3 format for workflows.

To optimize repetitive tasks and interconnect systems in a geomatic data processing pipeline [99], geomaticians should continually invest in languages such as Python, Scheme, and JavaScript as well as various tools.

### Traffic-Light Atlas

#### Traffic-Light Atlas Based on Semantic Relevance and Scientific Openness

Maps can be hard to read without knowing the cartographic or graphical semiology background. Our atlas targets a wide audience of users (the public, health care, political decision-makers, and the uMap community) seeking relevant, more comprehensive information that can be communicated to everyone. Intuitive comprehension is paramount for capturing and maintaining users’ attention. The Traffic-Light Atlas is particularly clear, offering a comprehensive overview of the current situation for each municipality.

#### Methods Based on the Statistical Signal Levels of the Traffic-Light Atlas

Although this is not an epidemiological article, this geomatics research project indirectly prompts an examination of the factors contributing to the observed variation in mortality rates across municipalities and districts, with some exhibiting high rates of excess mortality and others demonstrating low rates of submortality (reminder: the excess mortality in 2020 was compared with that in 2018 and 2019). The objective of our research was to provide these atlases at a highly detailed scale, ranging from the municipality to the submunicipal district and encompassing the entirety of France, including the DROMs. This approach enables each inhabitant of a municipality or district to assess its position in relation to its region, department, or national level.

#### Indirect Epidemiological Approach Based on Municipal Mortality Atlases

Two epidemiological aspects emerged indirectly from our municipal mortality atlases for 2020. The INSEE data used are for municipalities, except Marseille, Lyon, and Paris, which use district data.

The first aspect is that the available and less biased municipal-level statistics on all-cause mortality (see Multimedia Appendix 2 on cartographic information bias) supplement pandemic-era aggregated data studies [16,103-107]. One additional limitation should be highlighted: singular cases of small municipal populations that distort mortality data.

The second aspect is the source of the death data and our approach, which is similar to that of France 3 TV. It consists of a comparative analysis of mortality rates with those of previous years and the municipal grid. According to France 3, “Even if the cause of death is not mentioned in the INSEE data, an excess of mortality, compared with the two previous years, can be an indication of the impact of the coronavirus, whose information has been aggregated at the departmental level. This is how we were able to measure, for example, the impact of the deadly heatwave in France in the summer of 2003” [106].

#### Utility—Concrete Hypothetical Use Cases

##### Use Case 1

Use case 1 consists of retrospective audit triggering. A French regional health agency using our atlas would identify municipalities flagged in the orange or red class (Khi2LnFr>1.83; ie, O/E mortality ratio≥2) (see Multimedia Appendix 13). This signal would serve as a first-tier screening criterion to prioritize retrospective audits of death certificates in those municipalities—for example, to assess whether excess deaths reflect underdiagnosis of COVID-19, delayed care for chronic conditions (eg, cardiovascular emergencies), or outbreaks in long-term care facilities. This approach is consistent with published frameworks for excess mortality surveillance at the subnational level [108].

##### Use Case 2

Use case 2 consists of hospital surge capacity planning and learning. In a postcrisis quality analysis, a hospital network could correlate the geographic distribution of red class municipalities with Poisson *P*<.05 and its 2020 admission data, identifying spatial mismatches between areas of peak excess mortality and available intensive care unit capacity (see Table 4 and Multimedia Appendix 13). This retrospective triangulation could inform prospective surge capacity planning for future health crises.

##### Limitations—Nonspecificity of the All-Cause Mortality Signal

A fundamental epidemiological limitation of the Traffic-Light Atlas is the nonspecificity of the all-cause mortality signal. Although the use of all-cause mortality data circumvents the well-documented underascertainment issues associated with COVID-19–specific death counts during the early pandemic period, it also means that the excess mortality signal is not attributable solely to direct COVID-19 deaths. During the March 2020–May 2020 lockdown, several concurrent mechanisms may have contributed to or mitigated excess mortality: (1) delayed or foregone care for acute non-COVID-19 conditions (eg, myocardial infarction, stroke), as documented in multiple European studies; (2) reductions in road traffic and occupational accidents; and (3) reduced transmission of seasonal influenza. The Khi2LnFr signal therefore captures the net balance of these competing effects and should be interpreted as “a statistical signal” of municipal-level mortality deviation from the 2018‐2019 baseline, not as a direct COVID-19 death count. Future work could integrate complementary data sources—notably cause-specific mortality from the French National Institute of Health and Medical Research (Inserm) Center for Epidemiology on Medical Causes of Death (CépiDc) when available at the municipal level, as well as hospital admission data from the “Program for Medicalization of Information Systems”—to disentangle these contributions and enhance the epidemiological interpretability of the atlas [109].

### Perspectives

We must keep in mind that the reproducibility of the research was our guiding principle, which also applied to optimizing, implementing, and developing atlases in uMaps [71,110].

#### 2020 Atlas Project Covering 4 Periods

In addition to the initial version of the Traffic-Light Atlas presented in this study, we produced a second version covering 4 periods in 2020 (website published in November 2024 [7]). Owing to a minor statistical error in version 2 [7], our goal was to revise this version while reducing the software used in the geomatics data processing pipeline. This online version 2 of the atlas links 4 French policy measures to the 4 periods of 2020: from January 1 to February 28 inclusive (excluding the COVID-19 crisis), from March 1 to May 15 inclusive (first lockdown), from May 16 to October 28 inclusive (excluding lockdown), and from October 29 to December 31 inclusive (second French lockdown, less strict). The division into 4 periods facilitated a better analysis of the phenomenon (ie, the Traffic-Light Atlases for each period aligned better or more precisely with the policies implemented during the respective periods).

We began the first statistical exploration of new death data from INSEE on July 23, 2021. We noted that the methods used to collect deaths differed between our current version and the second version, which covered 4 periods. The statistics for version 2 showed that an “excess mortality statistical signal” affected 40% (13,944/34,833) of the municipalities. (Reminder: In version 1, the excess mortality statistical signal concerned 41.7% [14,503/34,833]).

Once the update to version 3 is complete, we will be able to compare the results of the atlases with those of health policies implemented nationwide. This will highlight the impact of effective policies and the more nuanced impact of others. We should cross-reference data on excess mortality or submortality with municipalities’ sociodemographic data. Comparing France with a neighboring country such as Belgium might be useful, but there are methodological obstacles to consider. The 4 periods of France and Belgium are out of sync [111], and it is unclear whether 2018 and 2019 are reference years.

Notwithstanding the disparities between Belgium and France regarding the covered time periods, it can be asserted that the methodology used in this study is applicable to any geographical area for which data on mortality (all causes combined) are available.

#### Geomatic Optimization and Reproducibility Based on the R Project

With and without webmapping, geomatic processing chains, such as the production of GeoJSON layers for the uMap Traffic-Light Atlas with “double legends and tool tips,” must be optimized. We also plan to improve how different environments work together. To do this, we could use (1) an ETL pipeline architecture consisting of proprietary software under different types of licenses and (2) a programming language such as Python, where libraries exist for most purposes [99,112]. In the short to medium term, the goal is to revise the atlases. As planned, we may switch to the R project to improve the reproducibility of the “geomatic data processing pipeline in step 1,” thereby eliminating the need for Excel, Philcarto, Google Earth, VS Code, and QGIS.

Digital advances and developers’ interest in geomatics mean that this challenge can be met by streamlining the geomatic processing chain and developing new mapping packages (in accordance with graphic semiology standards). These developers invite us to use the same software environment and R project to process all the data and generate maps; they encourage us to support reproducible cartography [74,110]. The “Cartography” package, now renamed “mapsf,” is more user-friendly, lighter, and more robust [73,74]. Our study’s methodological stages (data collection and cleansing-scheduling processes, analysis, and cartographic representation) could soon be followed and conducted in the same software environment to produce atlases, thus avoiding task dispersion and complications. In other words, this approach serves to unify the workflow and map reproducibility process. The R project could be considered an optimal choice for the geomatic data processing pipeline in step 1 without webmapping.

Reproducible cartography poses a challenge with respect to sensitive, regulated, or inaccessible health data. We cannot make health data available for cartographic reproduction, even for a designated health care community. This barrier is even stronger for nonhealth care communities. Nevertheless, new positive perspectives are emerging as “think tanks” made up of health care professionals [113] working to improve the reusability of health care data, particularly hospital data, for public health and epidemiological research.

#### uMap: Conceptual Evolution and Targeted Geomatic Development

##### Conceptual Evolution Through Sharing Our Atlas

Since uMap is a collaborative map-sharing platform, we decided to share our atlases and provide additional meaning. Users can now share our atlas URLs, download the source files in various formats (GeoJSON, GPX, KML, and CSV), share all the properties and data, and embed atlases in an iframe (for distribution and personalized comments) [11,88]. This initiative aimed to receive feedback from the uMap community and determine how many people have viewed the atlases. At this juncture, an evaluation has yet to be conducted to ascertain whether the use of our atlases within the uMap environment results in enhanced decision-making by users, whether they be professionals or others. In fact, regarding basic usage analysis, we know that we cannot obtain statistical evaluations from the “uMap OSM France server environment.” However, it may be possible to obtain them via the “uMap Framacarte - remote Framasoft server” or “a local uMap server dedicated to health and associated with a Matomo script” [114].

In summary, the use of Traffic-Light semantics should reinforce the appeal of the Traffic-Light Atlas. All-cause mortality atlases at the municipal level, fed by an open INSEE data source, can be included in this collaborative map-sharing concept without ethical constraints.

##### uMap France: Evolving and Progressing

We met with the creator of uMap in May 2023 and sent him our preprint article as soon as it was published in the summer of 2024. Moreover, we suggested some improvements to the uMap project’s GitHub site and reported an access error to our atlases [12,13,102]. Our geomatic study, the implementation of the atlases in uMap, and discussions with the uMap team ultimately led to a new feature developed by uMap called the “experimental choropleth layer,” which partially addresses our need to “create choropleth maps with associated double legends” [115]. It is accessible from the dashboard. We now need to evaluate this feature and provide our beta tester feedback.

In summary, a new “experimental choropleth layer” is available in the uMap dashboard. This requires testing; if it proves effective, this could eliminate the geomatic processing of “steps 1 and 2” considered in the R project. We have partially confirmed hypothesis 3: uMap is growing rapidly, owing to funding from the “uMap of public service” platform, a government server.

##### Toward a Conceptual Evolution: uMap Platform Dedicated to Health

The open-source nature of uMap allows the development of a dedicated health platform. However, health data are subject to a different regulatory framework than INSEE data. Therefore, it appears probable that a health platform would be reserved, in whole or in part, for private use. The new “uMap of public service” platform is reserved exclusively for civil servants.

In summary, the development of a private “uMap-Health” platform would be legitimate within the restrictive framework for health data. Based on the example of the Pixacare medical photo library, we could explore the development and diversification of the use of the “uMap-Health” platform with a new “collaborative health map library” feature [116].

### Conclusions

#### Value

We have demonstrated the value of the FOSS uMap [11], a collaborative and interoperable mapping platform that is experiencing rapid growth. By instantiating all-cause mortality atlases at the municipal level (covering the first lockdown in France), we demonstrated the potential of uMap by creating a new type of map in uMap: the choropleth map and double legends, which did not exist at the beginning of our atlas project. Among the atlases developed, we presented the main one in this study: the Traffic-Light Atlas. Owing to its strengths in terms of displaying and visualizing results, our France-wide atlas offers enormous potential in terms of attractiveness and usability (ergonomics) and in terms of understanding and semantic interpretation of cartographic and statistical results. This atlas is relevant because the instant message it conveys is easily interpretable by all audiences, particularly for the general public. In this context, existing maps related to COVID-19 have been supplemented by all-cause mortality Traffic-Light Atlas creation. In keeping with the concept of the uMap platform, our mortality atlases have been published within uMap and made available to users for sharing. The databases use open data from INSEE [84] and are based on a license not subject to the GDPR. To achieve the desired results, the uMap platform does not require any additional IT development or management of the remote uMap server (handled by OSM-fr).

#### Utility for Professionals

This municipality-level interactive uMap atlas is designed to support both operational public health decision-making and retrospective quality analyses (Multimedia Appendix 2 and Multimedia Appendix 13).

First, local public health teams and municipal crisis management units can use the interface for situational awareness by continuously monitoring spatial and temporal variations in mortality across municipalities, identifying emerging mortality hotspots, and promptly targeting public health communications, resource allocation, and preventive interventions (eg, action protocols during heat or pandemic waves and reinforced medical screening during epidemics).

Second, quality assurance, epidemiology, and health information teams can use atlases to plan and prioritize retrospective audits by systematically flagging municipalities, time periods (version 1 or version 2), and excess deaths relative to comparable areas and atypical trends. These signals then guide focused chart reviews, audit investigations of clinical care pathways, assessment of local health system capacity, and verification of data completeness and coding accuracy in mortality reporting systems.

By providing fine-grained, geocoded mortality intelligence at the municipal level—a resolution not systematically available in routine departmental or regional dashboards—this application complements existing surveillance systems rather than replacing them and supports evidence-based public health action at the local level where resource allocation and outbreak response decisions are made (Multimedia Appendix 2).

When available, basic usage analytics characterize how different stakeholder groups engage with the tool uMap atlases in real-world practice.

For future use of uMap, it would first be legitimate, within the restrictive framework of health data, to create a private uMap platform dedicated to health [116]. Second, as part of an epidemiological approach, our mortality atlases are useful in the retrospective context of the pandemic. To improve our understanding of the municipal landscape, following the detection of excess mortality or submortality statistical signals, we could consider combining our results with sociodemographic data from municipalities. Third, a second version of the Traffic-Light Atlas, encompassing 4 periods in 2020, will be updated using either the “experimental choropleth layer” feature recently developed by the uMap team or, if this feature proves insufficient, the geomatic optimization process via the R project. Finally, for real-time crisis management monitoring, the uMap collaborative platform could be relevant because of its new “choropleth layer” feature and its overall active development.

## Supplementary material

10.2196/82855 Multimedia Appendix 1 The POLESAT Health Geomatics Project and its latest tool: the uMap Mortality Atlases.

10.2196/82855 Multimedia Appendix 2 State of the art in geomatics.

10.2196/82855 Multimedia Appendix 3 Gross death dataset as well as the database accompanied by a script and the corresponding CSV output file associated with Figure 2.

10.2196/82855 Multimedia Appendix 4 Ready-to-use mortality data for the R Project program as an example of our second version of the atlas published by the Grouping of the Institut Catholique de Lille Hospitals (GHICL) covering 4 study periods: an RMarkdown “summary program example” in HTML format that processes the associated input files “mortality_France.csv” and “mortality_France_districts.csv” and generates ready-to-use files in XLSX and TXT formats for creating a KML map via Philcarto computer-aided design (CAD) software.

10.2196/82855 Multimedia Appendix 5 Detailed section describing the materials.

10.2196/82855 Multimedia Appendix 6 The public repository for generating the GeoJSON files in Figure 3A is available at [69].

10.2196/82855 Multimedia Appendix 7 The public repository for generating the GeoJSON files in Figure 3B is available at [70].

10.2196/82855 Multimedia Appendix 8 The public repository for generating the GeoJSON files in Figures 4 and 5.

10.2196/82855 Multimedia Appendix 9 The public repository for generating the GeoJSON files in Figure 6.

10.2196/82855 Multimedia Appendix 10 The public repository for generating the Double Legend files in Figure 7 is found at [72].

10.2196/82855 Multimedia Appendix 11 The public repository for generating the Double Legend files in Figure 8.

10.2196/82855 Multimedia Appendix 12 The public repository for generating the Double Legend files in Figure 9.

10.2196/82855 Multimedia Appendix 13 Detailed analysis of the articulation between DENSI and Khi2Lnfr, titled “From Densi_i Statistics to Verbal Label Categories.” The Excel file contains details of the 1-sided exact Poisson distribution (right-tailed) calculations for 34,833 municipalities.

## Abbreviations

CAD: computer-aided design
CépiDc: Center for Epidemiology on Medical Causes of Death
DROMs: overseas departments and regions of France
EMF: Enhanced Windows Metafile
ETL: extract, transform, load
FOSS: free and open-source software
GDPR: General Data Protection Regulation
GHICL: Grouping of the Institut Catholique de Lille Hospitals
GIS: geographic information system
GNU GPL: GNU General Public License
GPX: GPS eXchange file format
ICT: information and communications technology
IGN: French National Institute of Geographic and Forest Information
INSEE: French National Institute of Statistics and Economic Studies
Inserm: French National Institute of Health and Medical Research
KML: Keyhole Markup Language
O/E: observed/expected
OGC: Open Geospatial Consortium
OSM: OpenStreetMap
PMC: Philcarto macro language
SHP: shapefile
SVG: scalable vector graphics file format
UMAP: uMap file format
VS: Visual Studio
WGS: World Geodetic System
