# Supplementary material for: Relevance of the uMap Collaborative Platform as Support for Choropleth Mapping of a Traffic-Light Statistical Signal Atlas of All-Cause Mortality During the First French Lockdown: Geospatial Analysis
Source: JMIR Med Inform. 2026 Aug 27;14:e82855. doi: 10.2196/82855 (PMC13543287; doi:10.2196/82855)
Supplement: Supplementary file 1 [file medinform-v14-e82855-s001.zip › MA-SF_ThePOLESATgeomaticsProject/ThePOLESATgeomaticsProject.docx]

##### Supplementary_File - the POLESAT Health Geomatics Project and its Latest Tool: the uMap Mortality Atlases

The acronym POLESAT is an abbreviation for the term "Sanitary hubs", which is the capitalized form of the acronym used to denote the overarching project. The project is an innovative e-geo-platform that is intended to evolve over time and is aimed at both the general public and professionals [1, 2]. The POLESAT system currently consists of four modules. First, an e-atlas was developed for the purpose of visualizing hospital care supply and demand [3]. Second, a medicalized geographical support module has been designed for patient orientation and programmed medical choice [4]. Third, a prospective health planning tool with variable scenario simulation geometry is available in two versions. The public version is entitled "e-PoleSat-demo", and the private version is entitled "e-PoleSat-metier" [1, 2]. The fourth module of the POLESAT project focuses on the construction of all-cause mortality atlases at the municipal level: atlases of detailed territorial information in the uMap environment.

**The fourth module: uMap mortality atlases**

All-cause mortality atlases are available in the uMap environment, hosted on the umap.openstreetmap.fr server [5 , 6, 7], as are available in the French Catholic Hospitals of Lille (GHICL) domain on the ghicl.net server. The evolution of these two environments has been a gradual and parallel process, developed over time by the team of developers and geomatics specialists.

The undertaking of tasks in two disparate environments, each with its own distinct geomatics and development requirements, eased a process of comparative analysis and reflection on the technical approaches employed and the graphic and cartographic renderings obtained.

Comparisons were made between database processing, development and geomatics tasks with remote server management and geomatics tasks without remote server management [8]. These comparisons involved two types of "workflows", one based on a "software chain" and the other on "a single software environment".

- The initial workflow generates two categories of data pipelines [9 , 10] through the utilization of geomatic software, such as computer-aided design (CAD) and geographic information systems (GISs), accompanied by a source code editor, and an integrated development environment (IDE) for the R-project [11] (a programming language employed for statistical calculations and graphical outputs), spreadsheets, etc.
- The second workflow is characterized by its internal nature within the GIS QGIS software environment, thereby integrating specialized task automation tools [12].

Finally, the extant literature enumerates a number of positive comparisons and reflections on uMap, including the following: (1) data management in the context of collaborative mapping; (2) data confidentiality in the context of activist mapping; (3) contributor management in the context of "crowd mapping" [13]; and (4) open-source philosophy in the context of teaching collaborative mapping online [8 , 14].

This led us to undertake a comprehensive evaluation of the relative merits and drawbacks of the two working environments, culminating in the formulation of recommendations for future iterations.

**NB**: The uMap France platform, based on the OSM-Fr server, displays atlases of all-cause mortality and is the only environment presented in this article.

**Abbreviations**

**CAD:** computer-aided design

**GHICL:** French Catholic Hospitals of Lille

**GIS:** geographic information system

**IDE:** integrated development environment

**POLESAT:** Sanitary hubs

**References**

1.	Quesnel-Barbet A, Soula J, Sauleau E-A, Bazile P, Dufossez F, Albert F, et al. PoleSat, health planning modeling for decision support. Application to a reorganization of vascular catheterization provision, at the geographic level of "territorial hospital grouping. ISTE openscience. Oct 2022;Special issue: Hospital systems management and engineering(5):21. doi: 10.21494/ISTE.OP.2022.0883.

2.	Quesnel-Barbet A, Soula J, Sauleau E-A, Bazile P, Dufossez F, Maignant G, et al. PoleSat, health planning modeling for decision support. Application to a reorganization of vascular catheterization provision, at the geographic level of "territorial hospital grouping ISTE openscience. Janv 2022;Special issue: Hospital systems management and engineering(5):27. doi: 10.21494/ISTE.OP.2022.0774.

3.	Quesnel A, Soula J, Wyndels K, Beuscart R. POLESAT: e-Atlas of Health, Planning and Sustainable Development. Biomedical engineering and research. 2009;30(4):201-4. doi: 10.1016/j.irbm.2009.05.005.

4.	Quesnel-Barbet A, Soula J, Dufossez F, Sharma N, Ruhela A, Beuscart R. POLESAT, an innovative e-health geomatic platform in decision-making based on: Geographical approach, medical knowledge visualization and geographic information system and web-mapping architecture. IRBM. 2013;34(4-5):267-73. doi: 10.1016/j.irbm.2013.06.001.

5.	OpenStreetMap. Review 2018 OSM France services. 2018 [Accessed 2025-05-26]; Available from: <https://www.openstreetmap.fr/revue-2018-les-services-osm-france/>.

6.	Quesnel-Barbet A, Pagès T, Soula J, Maignant G, Hansske A. uMap atlas - Excess mortality alerts in 2020 expressed by the TrafficLight variable. French atlas monitoring all-cause mortality (health crisis - SARS COV-2). First French lockdown period: March 1 to May 15, 2020 inclusive. umap.openstreetmap.fr; 2023 [Accessed 2025-05-28]; Available from: <https://umap.openstreetmap.fr/en/map/polesatatlasmortalite2020_feutricolore_v1_1231703#6/46.572/4.944>.

7.	Anne Quesnel-Barbet, Thierry Pagès, Julien Soula, Gilles Maignant, Arnaud Hansske. 2020 Mortality Atlas: French Atlas for monitoring all-cause mortality - Reference mortality: years 2018-2019. Four periods for the second version of the atlas - The first lockdown period for the first version of the atlas - Two environments (GHICL e-atlas & uMap e-atlas). Lille, France: GHICL.net & uMap; 2024 [Accessed 2025-06-05]; 2nd & 1st versions:[Available from: <https://www.ghicl.fr/rubrique-177.html>

8.	Shahamati S, Baumann Y, Caquard S, Denieul-Pinsky L, Shaw E. uMap: A Free, Open-Source Alternative to Google My Maps. CartographicPerspectivesorg [Internet]. 2022; (99):[6–18 pp.]. Available from: <https://cartographicperspectives.org/index.php/journal/article/view/1729/2073>.

9.	ibm.com. What is a data pipeline? . 2023 [cited 2023 Accessed 2025-05-26]; Available from: <https://www.ibm.com/topics/data-pipeline>.

10.	Talend Corp. What is a data pipeline and how does it work? Definition of a data pipeline. 2022 [Accessed 2025-05-26]; Available from: <https://www.talend.com/fr/resources/what-is-a-data-pipeline/#:~:text=Un%20pipeline%20de%20donn%C3%A9es%20englobe,donn%C3%A9es%20pr%C3%AAtes%20%C3%A0%20%C3%AAtre%20exploit%C3%A9es>.

11.	R Core Team, Chambers J, Dalgaard P, Gentleman R, Hornik K, Ihaka R, et al. The R Project. The Comprehensive R Archive Network : R for Windows. v 4.3.1 ed. Global community: cran.r-project; 1997. p. GNU GPL v2.

12.	QGIS-Development-Team. QGIS is A Free, Open Source, Cross Platform (lin/win/mac) Geographic Information System (GIS) In: github.com/qgis/QGIS, editor. v 3.18.1-Zürich ed. Global community: Qgis.org; 2021. p. CC BY-SA 3.0; GPL-2.0.

13.	Wikipedia. Crowdmapping. Wikipedia.org; 2017 [updated page: 2024-09-12 - Accessed 2025-05-26]; Available from: <https://en.wikipedia.org/wiki/Crowdmapping>.

14.	UQAM, Côté-Lapointe S. Create a map with uMap. 2023-10-24 [cited 2023 Accessed 2025-05-27]; Available from: <https://recherchenumerique.uqam.ca/scenarios/creer-une-carte-avec-umap/>.
