# Supplementary material for: Relevance of the uMap Collaborative Platform as Support for Choropleth Mapping of a Traffic-Light Statistical Signal Atlas of All-Cause Mortality During the First French Lockdown: Geospatial Analysis
Source: JMIR Med Inform. 2026 Aug 27;14:e82855. doi: 10.2196/82855 (PMC13543287; doi:10.2196/82855)
Supplement: Supplementary file 2 [file medinform-v14-e82855-s002.zip › MA-SF_StateOfTheArtInGeomatics/MA-SF_stateOfTheArtInGeomatics.docx]

##### Supplementary_File – State of the Art in Geomatics

##### Detailed History of Digital Cartography

Geography is an integral part of our modern digital world, and maps are the main tools used. A map is a model of partial representation of the geographic space under analysis; many different types of maps are produced, with varying degrees of respect for scientific reasoning and graphic semiology standards [1-3]. Currently, maps are most often produced via computer-aided design (CAD) software or from software environments with multiple functionalities for spatial representation and analysis, such as geographic information systems (GISs) and shared geographic information platforms; all these environments stand for the world of geomatics.

Geomatics is a contraction of both words "geography," and "computer science." and is associated with the world of digital cartography and spatial analysis. According to Henri PORNON, this discipline addresses the management of spatially referenced data and involves the sciences and technologies associated with their acquisition, storage, processing, and dissemination. A distinction is made between "geographic data", which are representative of an object in the territory, and "localized data", which are representative of an object positioned in the territory [4]. By contracting geographic data and localized data, we obtained geolocalized data for the positioned objects.

In other words, geomatics are a group of IT methods and tools designed to collect, organize, analyze, and represent—in the sense of modeling—geolocalized data. It eases the acquisition, structuring, integration, and analysis of geolocated information prior to the publication of results in the form of maps. Geomatics is generally associated with GIS as the main digital tool for implementing the concepts and methods of geography [4].

Geomatics do not exist without geolocalized data; geolocalized data have been evolving since the 1970s, with the production, distribution, and use of public geolocalized data [4]. The recent concept of findable, accessible, interoperable, and reusable (FAIR) data [5, 6] is defined as the ability of IT systems (with little or no human intervention) to manage information in compliance with the four rules of FAIR. The quality of the data produced is also under scientific scrutiny in the collaborative OpenStreetMap (OSM) project: a free editable map of the world [7, 8].

The development of rigorous data management has given rise to a multitude of concepts, which in turn have had a significant impact on geolocalized data by inheritance. Below, we refer to examples of these concepts: open data—free databases [9, 10], crowdsourcing and crowd mapping—participative production and mapping [11, 12]; open-source—free and open computer code with authorized modification and redistribution; open-source model—provision of software and “open and free” code as opposed to proprietary code [13]; participatory Web 2.0 or contributory internet—shared information and geographic information platform; and collaborative platform—information commons and commons [14].

Location-based information and free geomatics [7] are affected by heritage through the "four rules of FAIR data" of free computing [5, 6]. These include interoperability, which guarantees (due to open standards) the exchange of files between users equipped with different hardware or software [15]. There are two types of interoperability: syntactic interoperability, which allows two systems to communicate with one another, and interdomain interoperability, which allows organizations to cooperate and exchange information with one another. Interoperability thus offers proprietary producers the opportunity to turn to these concepts, gradually leading to the emergence of production chains and pipelines that are completely free to use [7, 16].

Three categories of open standards accompany interoperability [17]. **(1)** Standards set by software publishers and users (eg, the shapefile file format (SHP) from ©ESRI, a GIS publisher, or the drawing file format **(**DWG**)** from ©AutoCAD, a drawing software publisher). **(2)** Formal standards are defined according to a strict protocol by standard bodies such as the open geospatial consortium (OGC), 1994 [5], which represents a worldwide resource community for geospatial information and standards. Other representative bodies include the French national organization for standardization (AFNOR) [18], the international organization for standardization (ISO) [19], the European Committee for Standardization (ECS) and Etalab [20] of the French interministerial digital department (DINUM), whose aim is to improve public service and public action through data; and **(3)** French government standards created to adapt uses to the French national territory. Thus, the French National Council for Geolocated Information (CNIG) was renamed (CNIG for geographic information becomes CNIG for geolocalized or geolocated information) to cover the topography of territories and, more recently, their location—coordinates and supports public and private players in meeting the evolving challenges of standardization, innovation, production, distribution and sharing of geolocated information. Finally, standard bodies such as the OGC and the CNIG comply with European policies, directives and regulations, such as the directive, infrastructure for spatial information in Europe (INSPIRE) and the European fair and innovative data (DataAct) [21].

The following are the major projects and standards of significant importance and impact for geomatics:

1. The standards of the international oil and gas producers association (IOGP), formerly the European petroleum survey group (EPSG) 1985 for geodesy maintenance and free sharing of the EPSG geodetic parameter dataset [22-24].
2. Web standards include the World Wide Web Consortium (W3C) for developers and by heritage for geomatics developers [10, 17, 25].
3. National projects are based on very large-scale building information modeling standards (BIM and GeoBIM) [26, 27].

The defence geospatial information working group (DGIWG) has been managing geospatial information and ensuring interoperability since 1983 by creating the standards, implementation guidelines and procedures necessary to enable the provision, exchange and use of standardized geospatial information [28].

##### Introductory of geomatics in healthcare

As seen in the summary of the history of digital cartography, geomatics has received help from continuous digital transformation. Maps are at the center of attention in many fields of business and research. Open-source geomatics is accelerating this trend, meaning that cartography is no longer the sole preservation of geolocation information specialists. Many professionals, such as IT specialists, data scientists, and open-source project contributors, are now involved in geomatics [7], and scientists (whether or not they specialize in location-based information) have embraced maps as attractive, useful, strategic, and valuable objects that support knowledge, spatial memory and reflection. They have also embraced maps as digital objects to be produced, automated and distributed.

Geomatics in healthcare has become increasingly important in recent decades [29]. Current societal developments, marked by the COVID-19 pandemic, have boosted the use of digital maps in healthcare. At the height of the crisis, millions of internet users and other readers followed maps. This societal awareness of health risks, accentuated by the COVID-19 pandemic, calls for a map to become an essential part of our society and public health.

New map showcases—based on geomatic technologies—are emerging in the form of online public, private or mixed dashboards, replacing more complex geographic information system (GIS) environments [30, 31]. Static or dynamic atlases are integrated into these environments, with geographical scales ranging from global to local, using geolocalized health data that are more or less aggregated according to the authorized territorial mesh for spatiotemporal analysis, which can lead to known cartographic information biases. For more details, see [32-35].

###### **Overview of mortality atlases**

Generally, the epidemiological statistics and mortality atlases they produce are essential tools for understanding mortality patterns and trends in a given region, zone or country and for comparing the mortality rates of the studied areas [36].

In France, for example, the French National Institute of Statistics and Economic Studies (INSEE) systematically publishes an interpretation of the country's mortality data, as well as several mortality atlases at the subnational (regional or departmental) level. Overall, these atlases show mortality rates by cause of death, age, and sex. These methods provide a better understanding of geographical variations and help individuals identify the most vulnerable population groups [37]. In addition, France public health (FPH), including the “Géodes” platform [38], is an organization in charge of continuous monitoring of the population's state of health and its evolution [39]. The “Sentinelles” network monitors the health of nineteen pathologies (infectious, seasonal, etc.) [40].

In June 2020, the France 3 media highlighted the fact that neither the FPH nor the French regional health agency (ARS) published epidemiological data on a municipality-by-municipality basis. In fact, daily reports are limited to deaths occurring in hospitals, as well as in French residential establishments for the dependent elderly (EHPAD), for which deaths are aggregated and transmitted only at the regional level [41].

At the global level, the World Health Organization (WHO) has also produced mortality atlases for specific countries and regions. These mortality atlases illustrate and present data on causes of death and mortality rates, as well as temporal trends and the development of these axes in different countries and regions of the world. This enables the WHO to monitor global mortality, assess progress in public health, and develop strategies to reduce these mortality rates [42].

These national and international atlases are important tools for decision makers, whether politicians or specialists involved in health or researchers or professionals at all levels working to improve public health.

**Abbreviations**

**AFNOR:** French national organization for standardization

**ARS:** French regional health agency

**BIM:** building information modeling

**CAD:** computer-aided design

**CNIG:** French National Council for Geolocated Information

**DataAct:** European fair and innovative data

**DGIWG:** defence geospatial information working group

**DINUM:** French interministerial digital department

**ECS:** European Committee for Standardization

**EHPAD:** French residential establishments for the dependent elderly

**EPSG:** European petroleum survey group

**FAIR**: findable, accessible, interoperable, and reusable

**FPH:** France public health

**GeoBIM:** building information modeling with geotechnical or geological modeling

**GIS:** geographic information system

**INSEE:** French National Institute of Statistics and Economic Studies

**INSPIRE**: Infrastructure for spatial information in the European community

**IOGP**: International Oil and Gas Producers’ association

**ISO:** International Organization for Standardization

**OGC:** open geospatial consortium

**OSM:** OpenStreetMap project

**W3C:** World Wide Web consortium

**WHO:** World Health Organization

**Computing: file extensions**

**(DWG):** drawing file format

**(SHP)**: shapefile file format

**References**

1.	Brunet R. Map and sketch: report on Arnould C, Denisart F, Mangin C, 1999, "Geography sketches for the baccalaureate", Paris : Ellipses. In addition, on : Poidevin D, 1999, "The Action Map. A practical guide to map design and production", Paris : Ellipses. Mappemonde [Internet]. 2000; 60(4):[45-6 pp.]. Available from: <https://www.persee.fr/doc/mappe_0764-3470_2000_num_60_4_1955_t16_0045_0000_26>.

2.	Poidevin D. The Map as a Means of Action. A practical guide to card design and production. Paris, France: Ellipses; 1999 15.06.1999. 200 p. ISBN: 9782729868086.

3.	Raveneau J. Bibliographic review of the article by Roger BRUNET (1987). The map: instructions for use. Ed Fayard/Reclus, 270 p. Cahiers de géographie du Québec. 1988;32 (85):99–102. doi: 10.7202/021949ar.

4.	Quesnel-Barbet A, Dufossez F, Souris M, Delerue D, Hansske A. Geomatics in health and applications in the north of France. Techniques de l'ingénieur 2016 06/10;TIB 628 (med4000):5; 1-41. doi: 10.51257/a-v1-med4000.

5.	Open Geospatial Consortium (OGC). The Home of Location Technology Innovation and Collaboration. 2022 [Accessed 2025-05-26]; Available from: <https://www.ogc.org/standards/>.

6.	Wikipedia. FAIR data - FAIR principles, as published by GO FAIR - Findable, Accessible, Interoperable and Reusable (FAIR). Wikipedia; 2018 [Accessed 2025-05-26]; Available from: <https://en.wikipedia.org/wiki/FAIR_data>.

7.	Hayat F. Production of digital commons and cartographic uses [Thesis type: Geography & Geomatics]. France: Paris Cité, France; 2019.

8.	wiki.openstreetmap.org. Welcome to the OpenStreetMap wiki. University College of London: Bytemark; 2022 [Accessed 2025-05-23]; Available from: <https://wiki.openstreetmap.org/wiki/Main_Page>.

9.	Becquet JC. Open data: the opening of public data - Free software issues, open standards, and interoperability. APITUX; 2004 [Accessed 2025-05-26]; Free software and open data serving local communities:[Available from: <http://www.apitux.org/index.php?2010/11/19/235-opendata-l-ouverture-des-donnees-publiques>.

10.	Becquet JC, editor. Open GIS, software, and data conference: technical training for students, professionals, and jobseekers. The world of free software: free software and open data serving local communities; 2006; XSALTO, Digne-les-Bains, France.: APITUX.

11.	Wikipedia. Participatory production (crowdsourcing). 2020 [Accessed 2025-05-26]; Available from: <https://fr.wikipedia.org/wiki/Production_participative>.

12.	Wikipedia. Crowdmapping. Wikipedia.org; 2017 [updated page: 2024-09-12 - Accessed 2025-05-26]; Available from: <https://en.wikipedia.org/wiki/Crowdmapping>.

13.	Noucher M, Gautreau P. Is open access reshuffling the deck? New perspectives for geographical data. Les Cahiers du numérique. 2013;9(1):57-83.

14.	Wikipedia. Web 2.0 (also known as participative web and social web). 2009 [Accessed 2025-05-23]; Available from: <https://en.wikipedia.org/wiki/Web_2.0>.

15.	Becquet JC. Open formats and interoperability. Open formats and interoperability [Internet]. 2004; (Accessed 2025-05-26). Available from: <http://www.apitux.org/index.php?2008/07/25/84-formats-ouverts-et-interoperabilite>.

16.	Talend Corp. What is a data pipeline and how does it work? Definition of a data pipeline. 2022 [Accessed 2025-05-26]; Available from: <https://www.talend.com/fr/resources/what-is-a-data-pipeline/#:~:text=Un%20pipeline%20de%20donn%C3%A9es%20englobe,donn%C3%A9es%20pr%C3%AAtes%20%C3%A0%20%C3%AAtre%20exploit%C3%A9es>.

17.	Bernard M, Brione M. GIS Observatory: Norms and standards, quo vadis? Paris, France: SIGMAG TV.fr; 2022 [updated Accessed 2025-05-26; cited Reprint]; 1-10]. Available from: <http://cnig.gouv.fr/IMG/pdf/_c_vpw_sigmag_sigtv.fr_n34_obervatoire_du_sig_enquete_normes_standards_-_octobre_2022.pdf>.

18.	International Organization for Standardization (ISO). French association for standardization (AFNOR) in cooperation with the International organization for standardization (ISO). La Plaine Saint-Denis, France: Afnor.org; 2022 [Accessed 2025-05-26]; Available from: <https://www.iso.org/organization/1738.html>.

19.	International organization for standardization (ISO). Open geospatial consortium (OGC), Inc. MA, USA: iso.org/organization/9825.html; 2022 [Accessed 2025-05-26]; Available from: <https://www.iso.org/organization/9825.html>.

20.	Wikipedia. Etalab : a French public administration to improve public service and public action through data. Fontenoy-Ségur, France: fr.wikipedia.org/wiki/Etalab; 2011 [Accessed 2025-05-26]; Available from: <https://fr.wikipedia.org/wiki/Etalab>.

21.	EU digital-strategy. Data act: proposal for a regulation on harmonized rules on fair access to and use of data. digital-strategy.ec.europa.eu; 2022 [Accessed 2025-05-26]; Available from: <https://digital-strategy.ec.europa.eu/en/library/data-act-proposal-regulation-harmonised-rules-fair-access-and-use-data>.

22.	ISO. ISO 19111:2019 Geographic information — Coordinate reference system. iso.org/standard/74039; 2019 [Accessed 2025-05-26]; Available from: <https://www.iso.org/standard/74039.html>.

23.	EPSG.io, MapTiler. EPSG:4326 -- WGS84 - World Geodetic System 1984, used in GPS. 2020 [Accessed 2025-05-26]; Available from: <https://epsg.io/4326>; <https://epsg.org/home.html>.

24.	ISO. 35.240.70; IT applications in science; Including digital geographic information. iso.org/ics/35.240.70/x/; 2022 [Accessed 2025-05-26]; Available from: <https://www.iso.org/ics/35.240.70/x/>.

25.	Roger J. Developing With Web Standards – Recommendations and best practices. 456bereastreet.com/lab/developing_with_web_standards; 2004 [Accessed 2025-05-26]; Available from: <https://www.456bereastreet.com/lab/developing_with_web_standards/>.

26.	Sarafinof D, Mistre A, Picinbono G, Vallet B, Heydel L. The GeoBIM approach: from land management to building management. Annales des Mines - Responsibility and the environment. 2019;94(2):42-6. doi: 10.3917/re1.094.0042.

27.	citygml wiki. The GeoBIM standard citygmlwiki.org; 2009.

28.	dgiwg.org. The Defence geospatial information working group (DGIWG) to collectively advance the state of geospatial interoperability. dgiwg.org; 1983 [Accessed 2025-05-26]; Available from: <https://dgiwg.org/about>.

29.	Boulos MN, Roudsari AV, Carson ER. Health geomatics: an enabling suite of technologies in health and healthcare. J Biomed Inform. 2001 Jun;34(3):195-219. PMID: 11723701. doi: 10.1006/jbin.2001.1015.

30.	Biot V. Scancovid. atih.sante.fr; 2021 [Accessed 2025-05-26]; Available from: <https://scancovid.scansante.fr/scancovid/>; <https://scancovid.scansante.fr/scancovid/_w_d5f596fd/?code=open_data>.

31.	Scansante.fr, ANAP, CSIS, DGOS, EHESP. Care and territories. scansante.fr.; 2023 [Accessed 2025-05-26]; Available from: <https://www.scansante.fr/actualites/soins-et-territoires>.

32.	Fry R, Hollinghurst J, Stagg HR, Thompson DA, Fronterre C, Orton C, et al. Real-time spatial health surveillance: Mapping the UK COVID-19 epidemic. Int J Med Inform. 2021 May;149(104400):1-8. PMID: 33667930. doi: 10.1016/j.ijmedinf.2021.104400.

33.	Cromley EK, McLafferty SL. GIS and Public Health: Guilford Press; 2011 Sept 2011. ISBN: 9781609187507.

34.	Jones K, Duncan C, Moon G. Context, scale, and the geography of health: understanding the relevance of ecological studies. American Journal of Public Health. 1998;88(2):216-22.

35.	Kwan MP. The uncertain geographic context problem. Annals of the Association of American Geographers. 2013;103(2):221-44. doi: 10.1080/00045608.2012.687349.

36.	Naghavi M, Mohsen N, Contributors. Global, regional, and national age-sex specific all-cause and cause-specific mortality for 240 causes of death, 1990-2013: a systematic analysis for the Global Burden of Disease Study 2013. Lancet. 2015 Jan 10;385(9963):117-71. PMID: 25530442. doi: 10.1016/s0140-6736(14)61682-2.

37.	cancer.fr. Cancers in France 2015 edition. [Public Health]: cancer.fr; 2015 [Accessed 2025-05-26]; Available from: <https://www.e-cancer.fr/content/download/148692/1867381/file/Les-cancers-en-France-edition-2015.pdf>.

38.	France. SP. Geodes: Geo Data in Public Health, the cartographic observatory of epidemiological indicators. geodes.santepubliquefrance; 2016 [Accessed 2025-05-26]; Available from: <https://geodes.santepubliquefrance.fr/#view=map2&c=indicator>.

39.	HAS. The French health monitoring institute (INVS). France: has-sante.fr; 1998-2016 [Accessed 2025-05-26]; Available from: <https://www.has-sante.fr/jcms/c_436647/fr/institut-de-veille-sanitaire#:~:text=L'Institut%20de%20veille%20sanitaire,la%20population%20et%20son%20%C3%A9volution>.

40.	INSERM, Sorbonne Université, Pierre Louis Institute for Epidemiology and Public Health (IPLESP). Sentinelles. 1984 [Accessed 2025-05-26]; Available from: <https://www.sentiweb.fr/>.

41.	France3-regions, Francetvinfo.fr, Fossurier Y. Survey: Coronavirus: Which municipalities in the French departments (administrative divisions) of Nord and Pas-de-Calais have recorded the highest mortality rates since March 1st? : France 3 TV; 2020 [Accessed 2025-05-26]; Available from: <https://france3-regions.francetvinfo.fr/hauts-de-france/enquete-coronavirus-quelles-communes-du-nord-du-pas-calais-enregistrent-surmortalite-1er-mars-1825674.html?fbclid=IwAR0ue9PUte3GmDvyRKI44WHl2An4YTjrbHUl8pFTiSEOmg7gz3BvrkTuQ-M#La%20surmortalit%C3%A9%20dans%20l'arrondissement%20de%20Lille>.

42.	WHO, editor. World health statistics 2014: Apps.who.int/iris/handle/10665/112738; 2014.
