# Supplementary material for: Relevance of the uMap Collaborative Platform as Support for Choropleth Mapping of a Traffic-Light Statistical Signal Atlas of All-Cause Mortality During the First French Lockdown: Geospatial Analysis
Source: JMIR Med Inform. 2026 Aug 27;14:e82855. doi: 10.2196/82855 (PMC13543287; doi:10.2196/82855)
Supplement: Supplementary file 4 [file medinform-v14-e82855-s004.zip › MA-SF_ready-to-useMortalityDataForR/atlas6_utf8_insee-annee2020_arrdts_loop_output.html]

atlas6\_utf8\_insee-annee2020\_arrdts\_loop\_output.R


# atlas6\_utf8\_insee-annee2020\_arrdts\_loop\_output.R

#### anne.barbet

#### 2024-08-27

```
##script -- atlas6_utf8_insee-annee2020_arrdts_loop_output-----
## An example to export 12 district files----
## 
##updated August 27, 2024----

#rm(list=ls())
getwd()
```

```
## [1] "C:/Users/anne.barbet/Desktop/_Decembre_21/R_data_ttt_2020/_src"
```

```
#[1] "C:/Users/anne.barbet/Desktop/_Decembre_21/R_data_ttt_2020"

options(max.print=30)
#packages
#* install library(openxlsx)----
library(openxlsx)
##use this package for V search or lookup - excel equivalent
#install.packages(“plyr”, dependencies = TRUE)
library(plyr)
#??mapvalues()

########"*********************************
#*****************************************
##New insee extraction dated July 23, 2021
##** The second file to be processed is dated December 12, 2021 ----
#**file with "arrondissements"----
#** The third file is dated December 14, 2021 ----
## 1- Import file france mortalite 2020----
##*recuperate variables from insee 2021 database:  COM, DEP and REG ----
##variable ID_DEP and DEP_LABEL 
##variable ID_region and REG_LABEL 


##2- Import file (December 12, 2021) ----
annee_2020 <- read.csv("C:/Users/anne.barbet/Desktop/_Decembre_21/R_data_ttt_2020/_data/from_thipages_12dec21/mortalite_france_2020vs2018-19_4periodes.csv", header = TRUE, sep = ",", 
                       dec = ".", stringsAsFactors = FALSE, encoding="UTF-8")


#*#https://delladata.fr/exporter-donnees-sorties-r/
#**-to create the folder data_output----
#dir.create("output_csv") 
#dir.create("./_data/from_thipages_14dec21_Arrdts")


##4- Import of the third version of Dec. 14, 21, databases of Paris, Lyon, Marseille detailed districts----
annee_2020_arrdts <- read.csv("C:/Users/anne.barbet/Desktop/_Decembre_21/R_data_ttt_2020/_data/from_thipages_14dec21_Arrdts/mortalite_france_2020vs2018-19_4periodes_arrondisements.csv", header = TRUE, sep = ",", 
                      dec = ".", stringsAsFactors = FALSE, encoding="UTF-8")

#head (annee_2020_arrdts, 180)

##5-Import official insee2020 geographic code: https://www.insee.fr/fr/information/4316069----
inseecom_2020 <- read.csv("C:/Users/anne.barbet/Desktop/_Decembre_21/R_data_ttt_2020/_data/insee_2020/communes2020-csv/communes2020.csv", header = TRUE, sep = ",", 
                           dec = ".", stringsAsFactors = FALSE, encoding="UTF-8")

#*#*****************************************
##Official geographic code as of January 1, 2021 ----
##https://www.insee.fr/fr/information/5057840----
##commune2021.csv downloaded December 02, 2021----
#*****************************************
##6-Import departement2020.csv----
DEP_20 <-read.csv("C:/Users/anne.barbet/Desktop/_Decembre_21/R_data_ttt_2020/_data/insee_2020/departement2020-csv/departement2020.csv", header = TRUE, sep = ",", 
                  dec = ",", stringsAsFactors = FALSE, encoding="UTF-8")
head(DEP_20 , 2)
```

```
##   dep reg cheflieu tncc   ncc nccenr libelle
## 1  01  84    01053    5   AIN    Ain     Ain
## 2  02  32    02408    5 AISNE  Aisne   Aisne
```

```
##7-Import region2020.csv----
REG_20 <-read.csv("C:/Users/anne.barbet/Desktop/_Decembre_21/R_data_ttt_2020/_data/insee_2020/region2020-csv/region2020.csv", header = TRUE, sep = ",", 
                  dec = ",", stringsAsFactors = FALSE, encoding="UTF-8")
head(REG_20 , 2)
```

```
##   reg cheflieu tncc        ncc     nccenr    libelle
## 1   1    97105    3 GUADELOUPE Guadeloupe Guadeloupe
## 2   2    97209    3 MARTINIQUE Martinique Martinique
```

```
##verif tableau d?but 10 lignes
##** correct ID_DEP, it is not ID Dept but and ID communes----
##verification table start 10 lines
head(REG_20, 18)
```

```
##   reg cheflieu tncc        ncc     nccenr    libelle
## 1   1    97105    3 GUADELOUPE Guadeloupe Guadeloupe
## 2   2    97209    3 MARTINIQUE Martinique Martinique
## 3   3    97302    3     GUYANE     Guyane     Guyane
## 4   4    97411    0 LA REUNION La Réunion La Réunion
## 5   6    97608    0    MAYOTTE    Mayotte    Mayotte
##  [ reached 'max' / getOption("max.print") -- omitted 13 rows ]
```

```
##SEPARATION#############
# rename variable
#names(df)[names(df) == 'old.var.name'] <- 'new.var.name'

 

#*##8-Verify unique values of variable ID_COMMUNE----
#*verify number year_2020$Iden unique : 35013 via function : unique()----
compte_2020 <- unique(annee_2020$Iden)
annee_2020$Iden
```

```
##  [1] "01001" "01002" "01004" "01005" "01006" "01007" "01008" "01009" "01010"
## [10] "01011" "01012" "01013" "01014" "01016" "01015" "01017" "01019" "01021"
## [19] "01022" "01453" "01023" "01024" "01025" "01026" "01027" "01028" "01170"
## [28] "01029" "01030" "01032"
##  [ reached getOption("max.print") -- omitted 140022 entries ]
```

```
##verification number inseecom_2020$com unique : 37235 via function : unique()
compteinsee_2020 <- unique(inseecom_2020$com)


##*rename var header SMM_10000 AS SMM_10000_R_ for better management ----
## of variables in philcarto
##(_R_ for a ratio variable, _N_ for a nominal variable, and _Q_ for a ##quantity variable).
 ##quantity variable)
names(annee_2020)[names(annee_2020) == 'SMM_10000'] <- 'SMM_10000_R_'


#*??paste()----
## compare two tables
##d2[!paste(d2$x1, d2$x2)%in%paste(d1$x1, d1$x2),]
comp1VAR_insee_2020 <- data.frame(inseecom_2020[!paste(inseecom_2020$com)%in%paste(annee_2020$Iden),])


## 9-Comparing two tables----
#d2[!paste(d2$x1, d2$x2)%in%paste(d1$x1, d1$x2),]
comp2VAR_annee_2020 <- data.frame(annee_2020[!paste(annee_2020$Iden)%in%paste(inseecom_2020$com),])


##*##10-Verify, compare and delete both df----
##*delete a dataframe or other object----
rm(comp1VAR_insee_2020)
rm(comp2VAR_annee_2020)


########## SEPARATION ###########
# CREATE VAR SVG_
# CONCATENER SVG_ & ID_COM
# function paste(), paste0
#https://stt4230.rbind.io/manipulation_donnees/structures_r/#fonctions-paste-et-paste0


#11-CREATE A VARIABLE WITH SVG----
#color <- c(“Yellow”, “Yellow”, “Red”, “Green”)
annee_2020$SVG <- c("SVG")

head (annee_2020$SVG,10)
```

```
##  [1] "SVG" "SVG" "SVG" "SVG" "SVG" "SVG" "SVG" "SVG" "SVG" "SVG"
```

```
#*#12- Concatener SVG variable and ID_COM variable with separator _ underscore ----
#*paste(LETTERS, letters, sep = “_”)----
annee_2020$Iden1 <- paste(annee_2020$SVG, annee_2020$ID_COMMUNE, sep = "_")
#*deletes bad variable Iden----
annee_2020$SVG <- NULL


##13- Reorganize dataframe move column----
#https://stackoverflow.com/questions/3369959/moving-columns-within-a-data-frame-without-retyping/18540144#18540144
#for example: ordered_columns_leftside=c('var10','var34','var8')
#df=df[c(ordered_columns_leftside, setdiff(names(df),ordered_columns_leftside))]


#* file reordered, to obtain column “Iden” in first position, Iden1 in 2nd position, the ID is adapted to the IDs of the map fund----
#WGS84 for March 2021, allowing output in .svg and .kml formats from philcarto
#setdiff()
names(annee_2020)
```

```
##  [1] "Iden"           "ID_DEP"         "ID_COMMUNE"     "LIB_COMMUNE"   
##  [5] "POPULATION"     "POP_MUNICIPALE" "DC_2018"        "DC_2019"       
##  [9] "DC_2018_19"     "DC_2020"        "SURMORTALITE"   "AGE_MOYEN"     
## [13] "SM_10000"       "SMM_10000_R_"   "statut"         "PERIOD"        
## [17] "Iden1"
```

```
ordered_columns_leftside=c('Iden','Iden1','ID_DEP','ID_COMMUNE','LIB_COMMUNE')
annee_2020=annee_2020[c(ordered_columns_leftside, setdiff(names(annee_2020),ordered_columns_leftside))]


#*#14- After comparison (line 58) output comp1VAR_annee, 1 value is different----
#*between the two tables compared the ID 27676 commune: “Les Trois Lacs”----
annee_2020$ID_COMMUNE == '01001'
```

```
##  [1]  TRUE FALSE FALSE FALSE FALSE FALSE FALSE FALSE FALSE FALSE FALSE FALSE
## [13] FALSE FALSE FALSE FALSE FALSE FALSE FALSE FALSE FALSE FALSE FALSE FALSE
## [25] FALSE FALSE FALSE FALSE FALSE FALSE
##  [ reached getOption("max.print") -- omitted 140022 entries ]
```

```
annee_2020$ID_COMMUNE == '14666'
```

```
##  [1] FALSE FALSE FALSE FALSE FALSE FALSE FALSE FALSE FALSE FALSE FALSE FALSE
## [13] FALSE FALSE FALSE FALSE FALSE FALSE FALSE FALSE FALSE FALSE FALSE FALSE
## [25] FALSE FALSE FALSE FALSE FALSE FALSE
##  [ reached getOption("max.print") -- omitted 140022 entries ]
```

```
annee_2020[34999:35013, 1]
```

```
##  [1] "97607" "97608" "97609" "97610" "97611" "97612" "97613" "97614" "97615"
## [10] "97616" "97617" "14666" "69123" "75056" "13055"
```

```
#??dim()
# AT THE END OF PRINT, not in chronological order for the four Identities
# “14666” “69123” “75056” “13055”
#14666 = commune of “Sannerville”, region, 28 Normandy, department 14 of “Calvados”
#commune restored on 31/12/2019

# #[14986] "97417" "97416" "97418" "97419" "97420" "97421" "97422" "97423" "97601"
# [14995] "97602" "97603" "97604" "97605" "97606" "97607" "97608" "97609" "97610"
# [15004] "97611" "97612" "97613" "97614" "97615" "97616" "97617" "14666" "69123"
# [15013] "75056" "13055"
#


##https://stackoverflow.com/questions/15303283/how-to-do-vlookup-and-fill-down-like-in-excel-in-r
##15- Vertical search for a method among 4----

##* ID STEP0, we start by creating the “ID” dataframe from annee_2020$ID_COMMUNE----
##*Initial data:----
##dat <- data.frame(HouseType = c("Semi", "Single", "Row", "Single", "Apartment", "Apartment", "Row"))
ID <-data.frame(annee_2020$Iden)
head (annee_2020$ID_COMMUNE,10)
```

```
##  [1] "01001" "01002" "01004" "01005" "01006" "01007" "01008" "01009" "01010"
## [10] "01011"
```

```
#LABEL <-data.frame(annee_2020$insee_DEP, annee_2020$insee_REG)

##* ID STEP1 Lookup / crosswalk table TABLE DES CORRESPONDANCES MAILLE COMMUNE 3 variables INSEE "COM", "REG", "DEP":----
##Correspondence table, communal grid, 3 INSEE variables: “COM”, “REG”, “DEP”:----
#lookup <- data.frame(type_text = c("Semi", "Single", "Row", "Apartment"), type_num = c(1, 2, 3, 4))
lookup_com <- data.frame(inseecom_2020$com, inseecom_2020$reg, inseecom_2020$dep)
head (lookup_com,10)
```

```
##    inseecom_2020.com inseecom_2020.reg inseecom_2020.dep
## 1              01001                84                01
## 2              01002                84                01
## 3              01004                84                01
## 4              01005                84                01
## 5              01006                84                01
## 6              01007                84                01
## 7              01008                84                01
## 8              01009                84                01
## 9              01010                84                01
## 10             01011                84                01
```

```
#lookup_dep <- data.frame(DEP_21$DEP, DEP_21$LIBELLE)
#lookup_reg <- data.frame(REG_21$REG, REG_21$LIBELLE)

##* ID STEP2 Create the new variableS insee_REG & insee_DEP in DATAFRAME "ID":----
##dat$house_type_num <- plyr::mapvalues(dat$HouseType, from = lookup$type_text, to = lookup$type_num)
ID$insee2020_reg <- plyr::mapvalues(ID$annee_2020.Iden, from = lookup_com$inseecom_2020.com, to = lookup_com$inseecom_2020.reg)
```

```
## The following `from` values were not present in `x`: 01015, 01025, 01033, 01036, 01059, 01080, 01091, 01095, 01097, 01098, 01119, 01120, 01122, 01130, 01137, 01144, 01154, 01172, 01176, 01182, 01185, 01186, 01187, 01204, 01205, 01215, 01218, 01221, 01227, 01271, 01286, 01292, 01300, 01312, 01316, 01324, 01338, 01340, 01341, 01409, 01413, 01414, 01417, 01426, 01442, 01453, 02018, 02026, 02053, 02147, 02153, 02161, 02285, 02300, 02301, 02325, 02348, 02360, 02434, 02439, 02458, 02475, 02479, 02597, 02630, 02646, 02669, 02733, 02771, 02811, 02825, 03123, 03153, 03158, 03168, 03318, 04003, 04010, 04011, 04014, 04029, 04033, 04052, 04082, 04100, 04105, 04120, 04131, 04153, 04165, 04198, 05001, 05002, 05005, 05020, 05024, 05034, 05042, 05053, 05067, 05069, 05088, 05101, 05118, 05120, 05125, 05132, 05138, 05139, 05143, 05150, 05175, 07011, 07016, 07043, 07046, 07057, 07135, 07164, 07165, 07246, 07256, 07262, 07271, 08007, 08009, 08012, 08042, 08050, 08051, 08053, 08072, 08079, 08112, 08114, 08115, 08116, 08145, 08152, 08173, 08177, 08198, 08213, 08258, 08261, 08265, 08266, 08267, 08285, 08311, 08337, 08345, 08359, 08371, 08441, 08443, 08475, 08490, 08491, 08493, 08495, 09028, 09036, 09062, 09135, 09286, 09296, 09302, 09317, 09334, 10003, 10036, 10277, 10311, 10415, 11050, 11097, 11131, 11171, 11251, 11298, 11323, 11329, 11349, 12005, 12014, 12020, 12021, 12040, 12076, 12081, 12087, 12090, 12112, 12114, 12117, 12120, 12123, 12126, 12132, 12173, 12177, 12196, 12218, 12223, 12224, 12245, 12270, 12271, 12279, 12285, 12304, 14002, 14004, 14005, 14008, 14011, 14013, 14017, 14027, 14028, 14029, 14031, 14035, 14037, 14051, 14052, 14056, 14058, 14061, 14065, 14073, 14074, 14075, 14081, 14098, 14099, 14105, 14109, 14112, 14113, 14114, 14115, 14128, 14129, 14139, 14142, 14143, 14144, 14148, 14150, 14151, 14152, 14153, 14154, 14155, 14156, 14157, 14158, 14164, 14170, 14174, 14176, 14178, 14186, 14187, 14188, 14189, 14192, 14200, 14201, 14208, 14210, 14212, 14213, 14217, 14219, 14222, 14235, 14247, 14253, 14255, 14259, 14264, 14265, 14268, 14279, 14281, 14292, 14294, 14296, 14313, 14317, 14321, 14324, 14330, 14331, 14339, 14340, 14342, 14347, 14349, 14350, 14355, 14356, 14357, 14359, 14361, 14371, 14372, 14373, 14376, 14382, 14386, 14387, 14388, 14395, 14406, 14410, 14413, 14414, 14415, 14416, 14417, 14418, 14420, 14422, 14423, 14429, 14431, 14432, 14433, 14440, 14441, 14442, 14443, 14444, 14450, 14456, 14459, 14462, 14463, 14471, 14472, 14474, 14475, 14477, 14481, 14489, 14493, 14503, 14505, 14508, 14517, 14518, 14521, 14523, 14525, 14527, 14532, 14537, 14538, 14539, 14543, 14544, 14545, 14548, 14549, 14551, 14553, 14554, 14560, 14564, 14568, 14570, 14573, 14576, 14577, 14579, 14580, 14581, 14583, 14584, 14585, 14591, 14596, 14597, 14599, 14600, 14608, 14611, 14615, 14616, 14618, 14628, 14629, 14632, 14633, 14634, 14636, 14638, 14642, 14647, 14653, 14654, 14655, 14658, 14662, 14670, 14671, 14672, 14683, 14686, 14688, 14689, 14690, 14691, 14693, 14695, 14696, 14702, 14703, 14704, 14717, 14718, 14722, 14725, 14726, 14727, 14729, 14730, 14740, 14746, 14749, 14750, 14757, 14762, 14763, 15027, 15031, 15035, 15044, 15047, 15062, 15068, 15071, 15099, 15108, 15109, 15115, 15136, 15138, 15141, 15142, 15145, 15150, 15171, 15177, 15181, 15195, 15197, 15210, 15227, 15239, 15268, 16004, 16005, 16017, 16021, 16023, 16033, 16043, 16046, 16051, 16082, 16092, 16094, 16106, 16110, 16115, 16126, 16129, 16148, 16149, 16153, 16156, 16172, 16175, 16179, 16192, 16201, 16202, 16204, 16214, 16224, 16228, 16230, 16247, 16257, 16259, 16262, 16274, 16278, 16281, 16286, 16294, 16313, 16314, 16322, 16327, 16328, 16332, 16339, 16344, 16371, 16376, 16386, 16391, 16406, 16410, 16411, 16417, 17001, 17040, 17090, 17103, 17123, 17144, 17169, 17189, 17219, 17272, 17277, 17340, 17344, 17352, 17456, 17457, 18023, 18123, 18239, 19010, 19019, 19032, 19098, 19101, 19123, 19127, 19183, 19185, 19197, 19218, 19252, 19267, 19282, 21073, 21178, 21183, 21213, 21272, 21318, 21327, 21352, 21443, 21452, 21453, 21486, 21507, 21513, 21621, 21623, 21697, 22007, 22038, 22046, 22051, 22055, 22058, 22066, 22078, 22080, 22084, 22093, 22100, 22102, 22107, 22123, 22151, 22154, 22158, 22167, 22173, 22191, 22192, 22203, 22206, 22209, 22219, 22247, 22251, 22253, 22264, 22290, 22292, 22297, 22298, 22303, 22357, 22367, 23094, 23109, 23121, 23126, 23149, 23153, 23161, 23163, 23189, 23192, 23231, 24013, 24026, 24028, 24033, 24035, 24044, 24047, 24053, 24064, 24065, 24072, 24079, 24092, 24093, 24099, 24103, 24112, 24117, 24118, 24127, 24147, 24166, 24170, 24172, 24178, 24198, 24201, 24203, 24204, 24216, 24219, 24233, 24235, 24239, 24249, 24253, 24258, 24259, 24270, 24283, 24310, 24312, 24315, 24316, 24333, 24343, 24344, 24362, 24363, 24364, 24368, 24369, 24376, 24387, 24389, 24391, 24402, 24423, 24427, 24431, 24433, 24435, 24439, 24447, 24467, 24469, 24475, 24490, 24497, 24503, 24530, 24540, 24554, 24561, 24579, 25010, 25028, 25034, 25035, 25037, 25064, 25076, 25128, 25140, 25144, 25146, 25165, 25167, 25168, 25205, 25272, 25291, 25302, 25337, 25353, 25358, 25363, 25396, 25407, 25409, 25424, 25434, 25438, 25480, 25484, 25509, 25543, 25568, 25581, 25585, 25587, 25606, 26001, 26086, 26184, 26187, 26210, 26297, 26354, 26366, 27007, 27011, 27022, 27032, 27041, 27049, 27058, 27060, 27062, 27070, 27084, 27085, 27088, 27089, 27090, 27092, 27093, 27105, 27107, 27112, 27121, 27122, 27128, 27131, 27143, 27145, 27150, 27157, 27159, 27160, 27172, 27175, 27191, 27195, 27197, 27198, 27211, 27213, 27221, 27223, 27225, 27244, 27250, 27253, 27255, 27257, 27262, 27263, 27264, 27265, 27268, 27270, 27274, 27277, 27283, 27290, 27292, 27294, 27296, 27297, 27302, 27303, 27305, 27308, 27344, 27356, 27362, 27402, 27412, 27416, 27425, 27447, 27448, 27449, 27452, 27467, 27471, 27479, 27484, 27491, 27499, 27506, 27510, 27513, 27515, 27516, 27519, 27523, 27526, 27532, 27541, 27549, 27551, 27554, 27565, 27566, 27573, 27574, 27578, 27581, 27588, 27596, 27600, 27607, 27626, 27628, 27634, 27636, 27637, 27638, 27639, 27642, 27647, 27648, 27651, 27653, 27657, 27676, 27679, 27685, 27687, 28002, 28011, 28012, 28015, 28017, 28018, 28020, 28043, 28044, 28063, 28083, 28093, 28097, 28101, 28103, 28112, 28115, 28125, 28133, 28138, 28145, 28150, 28165, 28174, 28179, 28181, 28199, 28204, 28205, 28224, 28228, 28236, 28238, 28241, 28258, 28262, 28288, 28295, 28307, 28311, 28318, 28320, 28328, 28330, 28331, 28334, 28338, 28340, 28356, 28361, 28376, 28381, 28384, 28399, 28406, 28412, 28413, 28416, 28420, 28422, 29003, 29021, 29052, 29076, 29092, 29127, 29129, 29149, 29199, 29203, 29219, 29227, 29266, 30052, 30157, 31016, 31154, 31298, 31307, 31412, 32011, 32074, 32168, 32179, 32344, 33018, 33055, 33106, 33107, 33267, 33371, 33380, 33495, 34246, 34330, 35004, 35011, 35036, 35048, 35053, 35060, 35069, 35074, 35083, 35100, 35113, 35129, 35147, 35158, 35163, 35168, 35176, 35184, 35191, 35209, 35220, 35254, 35257, 35267, 35269, 35282, 35292, 35293, 35301, 35303, 35308, 35323, 35341, 35344, 35348, 36039, 36072, 36093, 36151, 36183, 36201, 36206, 36229, 36244, 36245, 37021, 37102, 37120, 37135, 37209, 37227, 37232, 38001, 38014, 38016, 38021, 38022, 38025, 38028, 38073, 38121, 38125, 38145, 38163, 38165, 38225, 38227, 38253, 38262, 38274, 38284, 38292, 38293, 38297, 38302, 38305, 38306, 38367, 38395, 38435, 38456, 38479, 38534, 38541, 38560, 39005, 39012, 39018, 39023, 39036, 39043, 39044, 39064, 39069, 39075, 39113, 39115, 39135, 39158, 39161, 39177, 39186, 39190, 39195, 39209, 39213, 39215, 39226, 39243, 39256, 39258, 39260, 39264, 39273, 39286, 39294, 39303, 39309, 39329, 39332, 39339, 39340, 39341, 39347, 39358, 39368, 39369, 39371, 39378, 39382, 39383, 39395, 39414, 39417, 39438, 39440, 39442, 39482, 39484, 39485, 39488, 39491, 39509, 39510, 39530, 39537, 39542, 39544, 39549, 39562, 39564, 39566, 39576, 39578, 40009, 40048, 40107, 40243, 40302, 41005, 41011, 41015, 41021, 41023, 41033, 41055, 41056, 41059, 41064, 41070, 41076, 41082, 41092, 41133, 41142, 41151, 41165, 41167, 41169, 41170, 41171, 41173, 41183, 41197, 41202, 41210, 41227, 41240, 41244, 41248, 41257, 41263, 41264, 41270, 41272, 42004, 42039, 42080, 42082, 42114, 42190, 42245, 42250, 42268, 42291, 43176, 43255, 44004, 44005, 44008, 44011, 44017, 44021, 44029, 44034, 44040, 44059, 44087, 44093, 44147, 44163, 44180, 44181, 44191, 44213, 44219, 44225, 45003, 45007, 45048, 45051, 45057, 45090, 45106, 45129, 45140, 45163, 45172, 45190, 45191, 45192, 45211, 45221, 45236, 45267, 45287, 45295, 45304, 45318, 45319, 45349, 46014, 46019, 46025, 46033, 46048, 46063, 46067, 46071, 46077, 46083, 46099, 46103, 46110, 46138, 46141, 46150, 46156, 46158, 46166, 46201, 46232, 46248, 46252, 46261, 46262, 46263, 46268, 46274, 46275, 46278, 46285, 46287, 46291, 46298, 46300, 46311, 46325, 46326, 46327, 46331, 47116, 47149, 47166, 47268, 47322, 48009, 48011, 48014, 48022, 48023, 48027, 48035, 48038, 48040, 48047, 48049, 48050, 48057, 48060, 48061, 48062, 48066, 48076, 48078, 48084, 48087, 48093, 48094, 48099, 48101, 48102, 48105, 48116, 48120, 48122, 48125, 48126, 48127, 48133, 48134, 48139, 48142, 48146, 48152, 48154, 48162, 48164, 48166, 48172, 48180, 48183, 48184, 48186, 48189, 48195, 48196, 48197, 49001, 49003, 49004, 49005, 49006, 49013, 49014, 49016, 49018, 49019, 49021, 49023, 49024, 49025, 49029, 49031, 49032, 49033, 49034, 49037, 49039, 49040, 49042, 49043, 49044, 49046, 49047, 49049, 49050, 49051, 49052, 49059, 49060, 49062, 49065, 49066, 49067, 49069, 49071, 49072, 49073, 49074, 49075, 49077, 49078, 49079, 49080, 49081, 49083, 49084, 49085, 49086, 49087, 49088, 49091, 49092, 49093, 49094, 49095, 49096, 49097, 49098, 49101, 49103, 49104, 49105, 49106, 49108, 49111, 49115, 49116, 49117, 49118, 49119, 49122, 49125, 49126, 49128, 49133, 49134, 49136, 49137, 49138, 49139, 49141, 49142, 49143, 49145, 49147, 49148, 49149, 49150, 49151, 49153, 49154, 49156, 49157, 49158, 49159, 49162, 49163, 49165, 49167, 49168, 49169, 49172, 49173, 49174, 49175, 49177, 49179, 49181, 49183, 49184, 49185, 49186, 49187, 49189, 49190, 49191, 49194, 49196, 49197, 49198, 49199, 49200, 49202, 49204, 49206, 49207, 49208, 49212, 49213, 49218, 49225, 49226, 49227, 49228, 49229, 49230, 49232, 49233, 49234, 49238, 49239, 49242, 49243, 49244, 49245, 49248, 49249, 49250, 49251, 49252, 49254, 49256, 49258, 49261, 49263, 49264, 49265, 49268, 49270, 49273, 49274, 49276, 49277, 49279, 49280, 49281, 49282, 49285, 49286, 49287, 49289, 49290, 49292, 49293, 49295, 49296, 49297, 49298, 49300, 49301, 49303, 49304, 49305, 49307, 49309, 49312, 49313, 49314, 49315, 49316, 49317, 49318, 49319, 49320, 49322, 49323, 49324, 49325, 49327, 49331, 49335, 49337, 49342, 49345, 49346, 49348, 49349, 49350, 49351, 49354, 49356, 49360, 49363, 49365, 49366, 49367, 49372, 49373, 49375, 49376, 49377, 49379, 49380, 49382, 50001, 50005, 50007, 50009, 50010, 50012, 50014, 50018, 50020, 50035, 50037, 50041, 50043, 50047, 50051, 50053, 50056, 50057, 50061, 50063, 50067, 50073, 50075, 50080, 50082, 50089, 50090, 50095, 50099, 50100, 50103, 50107, 50113, 50116, 50119, 50123, 50125, 50127, 50128, 50129, 50131, 50132, 50133, 50134, 50136, 50139, 50140, 50142, 50153, 50154, 50160, 50163, 50168, 50170, 50171, 50173, 50179, 50180, 50187, 50189, 50191, 50197, 50201, 50202, 50203, 50204, 50209, 50211, 50212, 50213, 50215, 50220, 50223, 50224, 50236, 50239, 50242, 50244, 50245, 50248, 50249, 50250, 50254, 50255, 50257, 50260, 50267, 50273, 50280, 50284, 50286, 50287, 50292, 50293, 50301, 50306, 50308, 50309, 50313, 50316, 50318, 50319, 50320, 50322, 50323, 50325, 50329, 50330, 50333, 50337, 50339, 50343, 50344, 50346, 50348, 50354, 50355, 50358, 50359, 50363, 50367, 50375, 50377, 50380, 50381, 50385, 50386, 50388, 50391, 50393, 50396, 50400, 50404, 50409, 50410, 50412, 50414, 50415, 50416, 50417, 50418, 50419, 50424, 50427, 50431, 50432, 50434, 50436, 50438, 50439, 50440, 50441, 50444, 50449, 50458, 50460, 50465, 50470, 50477, 50484, 50485, 50487, 50492, 50494, 50497, 50501, 50503, 50508, 50515, 50516, 50520, 50523, 50524, 50526, 50534, 50544, 50545, 50546, 50550, 50557, 50558, 50561, 50564, 50565, 50573, 50581, 50582, 50583, 50586, 50591, 50592, 50595, 50600, 50601, 50602, 50605, 50608, 50611, 50614, 50620, 50622, 50623, 50625, 50627, 50630, 50631, 50632, 50635, 50636, 50638, 50639, 50640, 50642, 50644, 50646, 50649, 51021, 51024, 51030, 51064, 51075, 51171, 51261, 51271, 51331, 51347, 51383, 51405, 51411, 51494, 51564, 51575, 51612, 51651, 51664, 52010, 52018, 52020, 52032, 52036, 52041, 52049, 52052, 52054, 52064, 52068, 52071, 52073, 52077, 52078, 52080, 52081, 52100, 52111, 52112, 52117, 52139, 52140, 52143, 52144, 52150, 52153, 52154, 52166, 52176, 52180, 52186, 52187, 52191, 52192, 52202, 52209, 52210, 52215, 52224, 52225, 52226, 52236, 52238, 52239, 52252, 52259, 52261, 52262, 52263, 52268, 52270, 52279, 52281, 52283, 52293, 52296, 52299, 52309, 52314, 52317, 52324, 52329, 52331, 52333, 52339, 52340, 52345, 52351, 52361, 52368, 52379, 52381, 52382, 52389, 52404, 52405, 52408, 52409, 52410, 52411, 52412, 52417, 52418, 52427, 52434, 52435, 52441, 52449, 52451, 52460, 52462, 52466, 52477, 52478, 52481, 52496, 52498, 52501, 52508, 52509, 52521, 52527, 52529, 52530, 52531, 52532, 52533, 52537, 53004, 53006, 53014, 53017, 53029, 53032, 53044, 53050, 53060, 53062, 53065, 53092, 53095, 53097, 53104, 53124, 53136, 53137, 53138, 53159, 53161, 53185, 53194, 53205, 53207, 53215, 53228, 53231, 53241, 53244, 53252, 53254, 53255, 54099, 54341, 54342, 54506, 54557, 55003, 55006, 55016, 55019, 55020, 55030, 55052, 55056, 55074, 55086, 55090, 55092, 55112, 55126, 55131, 55136, 55151, 55152, 55161, 55164, 55176, 55190, 55203, 55205, 55213, 55223, 55230, 55231, 55233, 55234, 55235, 55238, 55240, 55249, 55259, 55273, 55283, 55287, 55294, 55305, 55308, 55309, 55314, 55318, 55319, 55342, 55390, 55392, 55393, 55402, 55413, 55417, 55418, 55432, 55440, 55441, 55451, 55455, 55478, 55480, 55483, 55486, 55491, 55499, 55513, 55529, 55537, 55538, 55539, 55542, 55548, 55550, 55558, 55585, 56016, 56037, 56038, 56059, 56061, 56064, 56102, 56138, 56142, 56144, 56150, 56183, 56187, 56192, 56197, 57021, 57023, 57135, 57148, 57157, 57184, 57279, 57285, 57378, 57420, 57432, 57439, 57482, 57523, 57578, 57585, 57608, 57722, 58022, 58026, 58091, 58100, 58167, 58204, 59154, 59248, 59260, 59298, 59355, 59380, 59404, 59540, 59561, 59588, 60018, 60029, 60038, 60054, 60080, 60096, 60196, 60209, 60245, 60246, 60256, 60300, 60417, 60453, 60455, 60475, 60532, 60570, 60644, 60649, 60682, 60690, 60694, 61003, 61004, 61007, 61009, 61016, 61019, 61025, 61027, 61031, 61033, 61045, 61047, 61057, 61058, 61059, 61065, 61073, 61081, 61083, 61090, 61096, 61106, 61109, 61110, 61112, 61115, 61116, 61125, 61127, 61131, 61135, 61136, 61144, 61145, 61153, 61154, 61155, 61157, 61161, 61167, 61172, 61173, 61174, 61175, 61177, 61179, 61184, 61185, 61186, 61191, 61194, 61196, 61200, 61201, 61204, 61205, 61211, 61220, 61223, 61226, 61228, 61230, 61231, 61235, 61236, 61239, 61246, 61247, 61249, 61250, 61253, 61270, 61280, 61282, 61285, 61292, 61294, 61296, 61299, 61305, 61306, 61309, 61311, 61313, 61315, 61318, 61320, 61324, 61325, 61335, 61337, 61338, 61339, 61340, 61341, 61343, 61353, 61354, 61355, 61356, 61359, 61364, 61368, 61375, 61377, 61378, 61380, 61383, 61403, 61409, 61410, 61417, 61428, 61429, 61431, 61434, 61437, 61441, 61449, 61455, 61458, 61460, 61463, 61465, 61468, 61469, 61470, 61471, 61474, 61477, 61478, 61483, 61484, 61486, 61489, 61491, 61496, 61504, 61506, 61509, 61511, 61513, 62110, 62226, 62294, 62295, 62431, 62471, 62482, 62575, 62691, 62757, 62807, 63018, 63068, 63078, 63133, 63160, 63226, 63244, 63255, 63266, 63316, 63448, 64020, 64030, 64055, 64076, 64163, 64164, 64169, 64225, 64248, 64278, 64333, 64384, 64402, 64497, 64553, 65008, 65027, 65092, 65122, 65188, 65192, 65227, 65246, 65282, 65365, 67004, 67007, 67014, 67015, 67041, 67042, 67064, 67114, 67153, 67157, 67158, 67170, 67171, 67193, 67207, 67211, 67219, 67224, 67243, 67251, 67262, 67284, 67294, 67297, 67316, 67332, 67372, 67374, 67376, 67390, 67393, 67399, 67402, 67418, 67419, 67431, 67439, 67457, 67469, 67495, 67496, 67512, 67533, 67539, 67549, 67560, 67561, 67562, 68006, 68012, 68031, 68056, 68070, 68108, 68133, 68143, 68162, 68164, 68201, 68206, 68219, 68233, 68240, 68272, 68310, 68314, 68319, 68320, 69015, 69019, 69024, 69025, 69041, 69048, 69066, 69073, 69101, 69114, 69128, 69129, 69135, 69144, 69146, 69147, 69150, 69157, 69158, 69159, 69179, 69185, 69195, 69208, 69210, 69211, 69213, 69221, 69222, 69223, 69224, 69228, 69237, 69247, 69248, 69251, 69255, 70033, 70034, 70068, 70108, 70123, 70131, 70139, 70173, 70191, 70209, 70223, 70241, 70245, 70246, 70281, 70300, 70333, 70345, 70346, 70354, 70370, 70382, 70391, 70418, 70434, 70458, 70465, 70475, 70489, 70497, 70551, 70570, 71055, 71134, 71180, 71260, 71279, 71288, 71304, 71375, 71432, 71511, 71582, 71587, 72023, 72025, 72063, 72069, 72071, 72080, 72081, 72082, 72097, 72108, 72116, 72117, 72128, 72137, 72138, 72159, 72162, 72176, 72189, 72203, 72207, 72240, 72258, 72262, 72284, 72288, 72301, 72304, 72308, 72318, 72363, 72384, 73002, 73003, 73006, 73010, 73035, 73038, 73045, 73046, 73056, 73060, 73062, 73080, 73093, 73102, 73108, 73111, 73112, 73115, 73118, 73126, 73127, 73135, 73143, 73144, 73150, 73151, 73158, 73163, 73167, 73169, 73174, 73185, 73187, 73198, 73199, 73203, 73209, 73212, 73215, 73227, 73235, 73236, 73238, 73239, 73244, 73257, 73260, 73263, 73264, 73266, 73284, 73287, 73290, 73295, 73305, 73321, 74010, 74011, 74022, 74084, 74093, 74113, 74115, 74120, 74123, 74167, 74182, 74187, 74204, 74217, 74245, 74268, 74270, 74275, 74282, 76003, 76027, 76031, 76034, 76037, 76041, 76044, 76073, 76078, 76080, 76081, 76089, 76098, 76127, 76137, 76145, 76146, 76164, 76191, 76215, 76248, 76258, 76267, 76276, 76277, 76289, 76301, 76310, 76326, 76337, 76376, 76401, 76444, 76466, 76476, 76496, 76525, 76539, 76607, 76618, 76625, 76639, 76643, 76659, 76674, 76696, 76701, 76704, 76713, 76729, 76742, 77017, 77028, 77166, 77170, 77189, 77213, 77299, 77316, 77324, 77334, 77346, 77351, 77375, 77399, 77433, 77491, 77497, 77499, 77502, 77504, 78158, 78251, 78320, 78503, 78524, 78551, 79004, 79005, 79006, 79013, 79014, 79021, 79026, 79027, 79028, 79030, 79033, 79035, 79039, 79041, 79043, 79044, 79051, 79052, 79053, 79054, 79061, 79063, 79064, 79065, 79068, 79072, 79073, 79075, 79077, 79083, 79093, 79098, 79099, 79105, 79107, 79113, 79143, 79155, 79168, 79171, 79173, 79174, 79178, 79179, 79181, 79182, 79185, 79186, 79187, 79188, 79192, 79193, 79196, 79199, 79217, 79219, 79222, 79224, 79232, 79233, 79237, 79240, 79247, 79260, 79261, 79264, 79272, 79275, 79279, 79280, 79282, 79285, 79288, 79292, 79296, 79314, 79317, 79318, 79321, 79323, 79324, 79325, 79327, 79328, 79330, 79333, 79334, 79344, 79353, 80007, 80111, 80175, 80209, 80257, 80289, 80295, 80363, 80382, 80394, 80398, 80419, 80441, 80447, 80454, 80457, 80483, 80485, 80492, 80505, 80509, 80510, 80532, 80551, 80564, 80581, 80594, 80608, 80610, 80612, 80621, 80625, 80689, 80712, 80731, 80739, 80745, 80761, 80768, 80816, 80817, 81057, 81062, 81091, 81113, 81153, 81226, 81233, 81241, 81260, 81296, 81301, 85008, 85009, 85019, 85027, 85030, 85043, 85044, 85048, 85052, 85063, 85068, 85069, 85079, 85080, 85084, 85090, 85091, 85107, 85122, 85146, 85150, 85152, 85154, 85162, 85165, 85168, 85177, 85180, 85197, 85203, 85212, 85213, 85217, 85219, 85224, 85230, 85257, 85263, 85270, 85272, 85275, 85279, 85299, 85302, 86019, 86021, 86030, 86043, 86053, 86056, 86060, 86067, 86071, 86082, 86101, 86115, 86123, 86146, 86155, 86166, 86179, 86185, 86188, 86199, 86208, 86212, 86216, 86219, 86240, 86245, 86259, 86267, 86277, 86278, 86281, 86283, 87026, 87028, 87055, 87097, 87098, 87128, 87136, 87171, 87173, 87175, 87184, 87196, 88018, 88029, 88067, 88112, 88204, 88218, 88234, 88235, 88337, 88361, 88420, 88465, 89001, 89003, 89047, 89052, 89070, 89078, 89086, 89090, 89097, 89103, 89107, 89109, 89114, 89130, 89135, 89138, 89140, 89157, 89166, 89174, 89178, 89192, 89196, 89197, 89213, 89231, 89241, 89243, 89260, 89275, 89293, 89294, 89317, 89322, 89330, 89334, 89340, 89343, 89356, 89357, 89358, 89366, 89372, 89381, 89388, 89389, 89401, 89405, 89411, 89420, 89421, 89429, 89435, 89448, 89454, 89457, 89473, 89476, 89484, 90068, 90073, 91182, 91222, 91228, 91390
```

```
ID$insee2020_dep <- plyr::mapvalues(ID$annee_2020.Iden, from = lookup_com$inseecom_2020.com, to = lookup_com$inseecom_2020.dep)
```

```
## The following `from` values were not present in `x`: 01015, 01025, 01033, 01036, 01059, 01080, 01091, 01095, 01097, 01098, 01119, 01120, 01122, 01130, 01137, 01144, 01154, 01172, 01176, 01182, 01185, 01186, 01187, 01204, 01205, 01215, 01218, 01221, 01227, 01271, 01286, 01292, 01300, 01312, 01316, 01324, 01338, 01340, 01341, 01409, 01413, 01414, 01417, 01426, 01442, 01453, 02018, 02026, 02053, 02147, 02153, 02161, 02285, 02300, 02301, 02325, 02348, 02360, 02434, 02439, 02458, 02475, 02479, 02597, 02630, 02646, 02669, 02733, 02771, 02811, 02825, 03123, 03153, 03158, 03168, 03318, 04003, 04010, 04011, 04014, 04029, 04033, 04052, 04082, 04100, 04105, 04120, 04131, 04153, 04165, 04198, 05001, 05002, 05005, 05020, 05024, 05034, 05042, 05053, 05067, 05069, 05088, 05101, 05118, 05120, 05125, 05132, 05138, 05139, 05143, 05150, 05175, 07011, 07016, 07043, 07046, 07057, 07135, 07164, 07165, 07246, 07256, 07262, 07271, 08007, 08009, 08012, 08042, 08050, 08051, 08053, 08072, 08079, 08112, 08114, 08115, 08116, 08145, 08152, 08173, 08177, 08198, 08213, 08258, 08261, 08265, 08266, 08267, 08285, 08311, 08337, 08345, 08359, 08371, 08441, 08443, 08475, 08490, 08491, 08493, 08495, 09028, 09036, 09062, 09135, 09286, 09296, 09302, 09317, 09334, 10003, 10036, 10277, 10311, 10415, 11050, 11097, 11131, 11171, 11251, 11298, 11323, 11329, 11349, 12005, 12014, 12020, 12021, 12040, 12076, 12081, 12087, 12090, 12112, 12114, 12117, 12120, 12123, 12126, 12132, 12173, 12177, 12196, 12218, 12223, 12224, 12245, 12270, 12271, 12279, 12285, 12304, 14002, 14004, 14005, 14008, 14011, 14013, 14017, 14027, 14028, 14029, 14031, 14035, 14037, 14051, 14052, 14056, 14058, 14061, 14065, 14073, 14074, 14075, 14081, 14098, 14099, 14105, 14109, 14112, 14113, 14114, 14115, 14128, 14129, 14139, 14142, 14143, 14144, 14148, 14150, 14151, 14152, 14153, 14154, 14155, 14156, 14157, 14158, 14164, 14170, 14174, 14176, 14178, 14186, 14187, 14188, 14189, 14192, 14200, 14201, 14208, 14210, 14212, 14213, 14217, 14219, 14222, 14235, 14247, 14253, 14255, 14259, 14264, 14265, 14268, 14279, 14281, 14292, 14294, 14296, 14313, 14317, 14321, 14324, 14330, 14331, 14339, 14340, 14342, 14347, 14349, 14350, 14355, 14356, 14357, 14359, 14361, 14371, 14372, 14373, 14376, 14382, 14386, 14387, 14388, 14395, 14406, 14410, 14413, 14414, 14415, 14416, 14417, 14418, 14420, 14422, 14423, 14429, 14431, 14432, 14433, 14440, 14441, 14442, 14443, 14444, 14450, 14456, 14459, 14462, 14463, 14471, 14472, 14474, 14475, 14477, 14481, 14489, 14493, 14503, 14505, 14508, 14517, 14518, 14521, 14523, 14525, 14527, 14532, 14537, 14538, 14539, 14543, 14544, 14545, 14548, 14549, 14551, 14553, 14554, 14560, 14564, 14568, 14570, 14573, 14576, 14577, 14579, 14580, 14581, 14583, 14584, 14585, 14591, 14596, 14597, 14599, 14600, 14608, 14611, 14615, 14616, 14618, 14628, 14629, 14632, 14633, 14634, 14636, 14638, 14642, 14647, 14653, 14654, 14655, 14658, 14662, 14670, 14671, 14672, 14683, 14686, 14688, 14689, 14690, 14691, 14693, 14695, 14696, 14702, 14703, 14704, 14717, 14718, 14722, 14725, 14726, 14727, 14729, 14730, 14740, 14746, 14749, 14750, 14757, 14762, 14763, 15027, 15031, 15035, 15044, 15047, 15062, 15068, 15071, 15099, 15108, 15109, 15115, 15136, 15138, 15141, 15142, 15145, 15150, 15171, 15177, 15181, 15195, 15197, 15210, 15227, 15239, 15268, 16004, 16005, 16017, 16021, 16023, 16033, 16043, 16046, 16051, 16082, 16092, 16094, 16106, 16110, 16115, 16126, 16129, 16148, 16149, 16153, 16156, 16172, 16175, 16179, 16192, 16201, 16202, 16204, 16214, 16224, 16228, 16230, 16247, 16257, 16259, 16262, 16274, 16278, 16281, 16286, 16294, 16313, 16314, 16322, 16327, 16328, 16332, 16339, 16344, 16371, 16376, 16386, 16391, 16406, 16410, 16411, 16417, 17001, 17040, 17090, 17103, 17123, 17144, 17169, 17189, 17219, 17272, 17277, 17340, 17344, 17352, 17456, 17457, 18023, 18123, 18239, 19010, 19019, 19032, 19098, 19101, 19123, 19127, 19183, 19185, 19197, 19218, 19252, 19267, 19282, 21073, 21178, 21183, 21213, 21272, 21318, 21327, 21352, 21443, 21452, 21453, 21486, 21507, 21513, 21621, 21623, 21697, 22007, 22038, 22046, 22051, 22055, 22058, 22066, 22078, 22080, 22084, 22093, 22100, 22102, 22107, 22123, 22151, 22154, 22158, 22167, 22173, 22191, 22192, 22203, 22206, 22209, 22219, 22247, 22251, 22253, 22264, 22290, 22292, 22297, 22298, 22303, 22357, 22367, 23094, 23109, 23121, 23126, 23149, 23153, 23161, 23163, 23189, 23192, 23231, 24013, 24026, 24028, 24033, 24035, 24044, 24047, 24053, 24064, 24065, 24072, 24079, 24092, 24093, 24099, 24103, 24112, 24117, 24118, 24127, 24147, 24166, 24170, 24172, 24178, 24198, 24201, 24203, 24204, 24216, 24219, 24233, 24235, 24239, 24249, 24253, 24258, 24259, 24270, 24283, 24310, 24312, 24315, 24316, 24333, 24343, 24344, 24362, 24363, 24364, 24368, 24369, 24376, 24387, 24389, 24391, 24402, 24423, 24427, 24431, 24433, 24435, 24439, 24447, 24467, 24469, 24475, 24490, 24497, 24503, 24530, 24540, 24554, 24561, 24579, 25010, 25028, 25034, 25035, 25037, 25064, 25076, 25128, 25140, 25144, 25146, 25165, 25167, 25168, 25205, 25272, 25291, 25302, 25337, 25353, 25358, 25363, 25396, 25407, 25409, 25424, 25434, 25438, 25480, 25484, 25509, 25543, 25568, 25581, 25585, 25587, 25606, 26001, 26086, 26184, 26187, 26210, 26297, 26354, 26366, 27007, 27011, 27022, 27032, 27041, 27049, 27058, 27060, 27062, 27070, 27084, 27085, 27088, 27089, 27090, 27092, 27093, 27105, 27107, 27112, 27121, 27122, 27128, 27131, 27143, 27145, 27150, 27157, 27159, 27160, 27172, 27175, 27191, 27195, 27197, 27198, 27211, 27213, 27221, 27223, 27225, 27244, 27250, 27253, 27255, 27257, 27262, 27263, 27264, 27265, 27268, 27270, 27274, 27277, 27283, 27290, 27292, 27294, 27296, 27297, 27302, 27303, 27305, 27308, 27344, 27356, 27362, 27402, 27412, 27416, 27425, 27447, 27448, 27449, 27452, 27467, 27471, 27479, 27484, 27491, 27499, 27506, 27510, 27513, 27515, 27516, 27519, 27523, 27526, 27532, 27541, 27549, 27551, 27554, 27565, 27566, 27573, 27574, 27578, 27581, 27588, 27596, 27600, 27607, 27626, 27628, 27634, 27636, 27637, 27638, 27639, 27642, 27647, 27648, 27651, 27653, 27657, 27676, 27679, 27685, 27687, 28002, 28011, 28012, 28015, 28017, 28018, 28020, 28043, 28044, 28063, 28083, 28093, 28097, 28101, 28103, 28112, 28115, 28125, 28133, 28138, 28145, 28150, 28165, 28174, 28179, 28181, 28199, 28204, 28205, 28224, 28228, 28236, 28238, 28241, 28258, 28262, 28288, 28295, 28307, 28311, 28318, 28320, 28328, 28330, 28331, 28334, 28338, 28340, 28356, 28361, 28376, 28381, 28384, 28399, 28406, 28412, 28413, 28416, 28420, 28422, 29003, 29021, 29052, 29076, 29092, 29127, 29129, 29149, 29199, 29203, 29219, 29227, 29266, 30052, 30157, 31016, 31154, 31298, 31307, 31412, 32011, 32074, 32168, 32179, 32344, 33018, 33055, 33106, 33107, 33267, 33371, 33380, 33495, 34246, 34330, 35004, 35011, 35036, 35048, 35053, 35060, 35069, 35074, 35083, 35100, 35113, 35129, 35147, 35158, 35163, 35168, 35176, 35184, 35191, 35209, 35220, 35254, 35257, 35267, 35269, 35282, 35292, 35293, 35301, 35303, 35308, 35323, 35341, 35344, 35348, 36039, 36072, 36093, 36151, 36183, 36201, 36206, 36229, 36244, 36245, 37021, 37102, 37120, 37135, 37209, 37227, 37232, 38001, 38014, 38016, 38021, 38022, 38025, 38028, 38073, 38121, 38125, 38145, 38163, 38165, 38225, 38227, 38253, 38262, 38274, 38284, 38292, 38293, 38297, 38302, 38305, 38306, 38367, 38395, 38435, 38456, 38479, 38534, 38541, 38560, 39005, 39012, 39018, 39023, 39036, 39043, 39044, 39064, 39069, 39075, 39113, 39115, 39135, 39158, 39161, 39177, 39186, 39190, 39195, 39209, 39213, 39215, 39226, 39243, 39256, 39258, 39260, 39264, 39273, 39286, 39294, 39303, 39309, 39329, 39332, 39339, 39340, 39341, 39347, 39358, 39368, 39369, 39371, 39378, 39382, 39383, 39395, 39414, 39417, 39438, 39440, 39442, 39482, 39484, 39485, 39488, 39491, 39509, 39510, 39530, 39537, 39542, 39544, 39549, 39562, 39564, 39566, 39576, 39578, 40009, 40048, 40107, 40243, 40302, 41005, 41011, 41015, 41021, 41023, 41033, 41055, 41056, 41059, 41064, 41070, 41076, 41082, 41092, 41133, 41142, 41151, 41165, 41167, 41169, 41170, 41171, 41173, 41183, 41197, 41202, 41210, 41227, 41240, 41244, 41248, 41257, 41263, 41264, 41270, 41272, 42004, 42039, 42080, 42082, 42114, 42190, 42245, 42250, 42268, 42291, 43176, 43255, 44004, 44005, 44008, 44011, 44017, 44021, 44029, 44034, 44040, 44059, 44087, 44093, 44147, 44163, 44180, 44181, 44191, 44213, 44219, 44225, 45003, 45007, 45048, 45051, 45057, 45090, 45106, 45129, 45140, 45163, 45172, 45190, 45191, 45192, 45211, 45221, 45236, 45267, 45287, 45295, 45304, 45318, 45319, 45349, 46014, 46019, 46025, 46033, 46048, 46063, 46067, 46071, 46077, 46083, 46099, 46103, 46110, 46138, 46141, 46150, 46156, 46158, 46166, 46201, 46232, 46248, 46252, 46261, 46262, 46263, 46268, 46274, 46275, 46278, 46285, 46287, 46291, 46298, 46300, 46311, 46325, 46326, 46327, 46331, 47116, 47149, 47166, 47268, 47322, 48009, 48011, 48014, 48022, 48023, 48027, 48035, 48038, 48040, 48047, 48049, 48050, 48057, 48060, 48061, 48062, 48066, 48076, 48078, 48084, 48087, 48093, 48094, 48099, 48101, 48102, 48105, 48116, 48120, 48122, 48125, 48126, 48127, 48133, 48134, 48139, 48142, 48146, 48152, 48154, 48162, 48164, 48166, 48172, 48180, 48183, 48184, 48186, 48189, 48195, 48196, 48197, 49001, 49003, 49004, 49005, 49006, 49013, 49014, 49016, 49018, 49019, 49021, 49023, 49024, 49025, 49029, 49031, 49032, 49033, 49034, 49037, 49039, 49040, 49042, 49043, 49044, 49046, 49047, 49049, 49050, 49051, 49052, 49059, 49060, 49062, 49065, 49066, 49067, 49069, 49071, 49072, 49073, 49074, 49075, 49077, 49078, 49079, 49080, 49081, 49083, 49084, 49085, 49086, 49087, 49088, 49091, 49092, 49093, 49094, 49095, 49096, 49097, 49098, 49101, 49103, 49104, 49105, 49106, 49108, 49111, 49115, 49116, 49117, 49118, 49119, 49122, 49125, 49126, 49128, 49133, 49134, 49136, 49137, 49138, 49139, 49141, 49142, 49143, 49145, 49147, 49148, 49149, 49150, 49151, 49153, 49154, 49156, 49157, 49158, 49159, 49162, 49163, 49165, 49167, 49168, 49169, 49172, 49173, 49174, 49175, 49177, 49179, 49181, 49183, 49184, 49185, 49186, 49187, 49189, 49190, 49191, 49194, 49196, 49197, 49198, 49199, 49200, 49202, 49204, 49206, 49207, 49208, 49212, 49213, 49218, 49225, 49226, 49227, 49228, 49229, 49230, 49232, 49233, 49234, 49238, 49239, 49242, 49243, 49244, 49245, 49248, 49249, 49250, 49251, 49252, 49254, 49256, 49258, 49261, 49263, 49264, 49265, 49268, 49270, 49273, 49274, 49276, 49277, 49279, 49280, 49281, 49282, 49285, 49286, 49287, 49289, 49290, 49292, 49293, 49295, 49296, 49297, 49298, 49300, 49301, 49303, 49304, 49305, 49307, 49309, 49312, 49313, 49314, 49315, 49316, 49317, 49318, 49319, 49320, 49322, 49323, 49324, 49325, 49327, 49331, 49335, 49337, 49342, 49345, 49346, 49348, 49349, 49350, 49351, 49354, 49356, 49360, 49363, 49365, 49366, 49367, 49372, 49373, 49375, 49376, 49377, 49379, 49380, 49382, 50001, 50005, 50007, 50009, 50010, 50012, 50014, 50018, 50020, 50035, 50037, 50041, 50043, 50047, 50051, 50053, 50056, 50057, 50061, 50063, 50067, 50073, 50075, 50080, 50082, 50089, 50090, 50095, 50099, 50100, 50103, 50107, 50113, 50116, 50119, 50123, 50125, 50127, 50128, 50129, 50131, 50132, 50133, 50134, 50136, 50139, 50140, 50142, 50153, 50154, 50160, 50163, 50168, 50170, 50171, 50173, 50179, 50180, 50187, 50189, 50191, 50197, 50201, 50202, 50203, 50204, 50209, 50211, 50212, 50213, 50215, 50220, 50223, 50224, 50236, 50239, 50242, 50244, 50245, 50248, 50249, 50250, 50254, 50255, 50257, 50260, 50267, 50273, 50280, 50284, 50286, 50287, 50292, 50293, 50301, 50306, 50308, 50309, 50313, 50316, 50318, 50319, 50320, 50322, 50323, 50325, 50329, 50330, 50333, 50337, 50339, 50343, 50344, 50346, 50348, 50354, 50355, 50358, 50359, 50363, 50367, 50375, 50377, 50380, 50381, 50385, 50386, 50388, 50391, 50393, 50396, 50400, 50404, 50409, 50410, 50412, 50414, 50415, 50416, 50417, 50418, 50419, 50424, 50427, 50431, 50432, 50434, 50436, 50438, 50439, 50440, 50441, 50444, 50449, 50458, 50460, 50465, 50470, 50477, 50484, 50485, 50487, 50492, 50494, 50497, 50501, 50503, 50508, 50515, 50516, 50520, 50523, 50524, 50526, 50534, 50544, 50545, 50546, 50550, 50557, 50558, 50561, 50564, 50565, 50573, 50581, 50582, 50583, 50586, 50591, 50592, 50595, 50600, 50601, 50602, 50605, 50608, 50611, 50614, 50620, 50622, 50623, 50625, 50627, 50630, 50631, 50632, 50635, 50636, 50638, 50639, 50640, 50642, 50644, 50646, 50649, 51021, 51024, 51030, 51064, 51075, 51171, 51261, 51271, 51331, 51347, 51383, 51405, 51411, 51494, 51564, 51575, 51612, 51651, 51664, 52010, 52018, 52020, 52032, 52036, 52041, 52049, 52052, 52054, 52064, 52068, 52071, 52073, 52077, 52078, 52080, 52081, 52100, 52111, 52112, 52117, 52139, 52140, 52143, 52144, 52150, 52153, 52154, 52166, 52176, 52180, 52186, 52187, 52191, 52192, 52202, 52209, 52210, 52215, 52224, 52225, 52226, 52236, 52238, 52239, 52252, 52259, 52261, 52262, 52263, 52268, 52270, 52279, 52281, 52283, 52293, 52296, 52299, 52309, 52314, 52317, 52324, 52329, 52331, 52333, 52339, 52340, 52345, 52351, 52361, 52368, 52379, 52381, 52382, 52389, 52404, 52405, 52408, 52409, 52410, 52411, 52412, 52417, 52418, 52427, 52434, 52435, 52441, 52449, 52451, 52460, 52462, 52466, 52477, 52478, 52481, 52496, 52498, 52501, 52508, 52509, 52521, 52527, 52529, 52530, 52531, 52532, 52533, 52537, 53004, 53006, 53014, 53017, 53029, 53032, 53044, 53050, 53060, 53062, 53065, 53092, 53095, 53097, 53104, 53124, 53136, 53137, 53138, 53159, 53161, 53185, 53194, 53205, 53207, 53215, 53228, 53231, 53241, 53244, 53252, 53254, 53255, 54099, 54341, 54342, 54506, 54557, 55003, 55006, 55016, 55019, 55020, 55030, 55052, 55056, 55074, 55086, 55090, 55092, 55112, 55126, 55131, 55136, 55151, 55152, 55161, 55164, 55176, 55190, 55203, 55205, 55213, 55223, 55230, 55231, 55233, 55234, 55235, 55238, 55240, 55249, 55259, 55273, 55283, 55287, 55294, 55305, 55308, 55309, 55314, 55318, 55319, 55342, 55390, 55392, 55393, 55402, 55413, 55417, 55418, 55432, 55440, 55441, 55451, 55455, 55478, 55480, 55483, 55486, 55491, 55499, 55513, 55529, 55537, 55538, 55539, 55542, 55548, 55550, 55558, 55585, 56016, 56037, 56038, 56059, 56061, 56064, 56102, 56138, 56142, 56144, 56150, 56183, 56187, 56192, 56197, 57021, 57023, 57135, 57148, 57157, 57184, 57279, 57285, 57378, 57420, 57432, 57439, 57482, 57523, 57578, 57585, 57608, 57722, 58022, 58026, 58091, 58100, 58167, 58204, 59154, 59248, 59260, 59298, 59355, 59380, 59404, 59540, 59561, 59588, 60018, 60029, 60038, 60054, 60080, 60096, 60196, 60209, 60245, 60246, 60256, 60300, 60417, 60453, 60455, 60475, 60532, 60570, 60644, 60649, 60682, 60690, 60694, 61003, 61004, 61007, 61009, 61016, 61019, 61025, 61027, 61031, 61033, 61045, 61047, 61057, 61058, 61059, 61065, 61073, 61081, 61083, 61090, 61096, 61106, 61109, 61110, 61112, 61115, 61116, 61125, 61127, 61131, 61135, 61136, 61144, 61145, 61153, 61154, 61155, 61157, 61161, 61167, 61172, 61173, 61174, 61175, 61177, 61179, 61184, 61185, 61186, 61191, 61194, 61196, 61200, 61201, 61204, 61205, 61211, 61220, 61223, 61226, 61228, 61230, 61231, 61235, 61236, 61239, 61246, 61247, 61249, 61250, 61253, 61270, 61280, 61282, 61285, 61292, 61294, 61296, 61299, 61305, 61306, 61309, 61311, 61313, 61315, 61318, 61320, 61324, 61325, 61335, 61337, 61338, 61339, 61340, 61341, 61343, 61353, 61354, 61355, 61356, 61359, 61364, 61368, 61375, 61377, 61378, 61380, 61383, 61403, 61409, 61410, 61417, 61428, 61429, 61431, 61434, 61437, 61441, 61449, 61455, 61458, 61460, 61463, 61465, 61468, 61469, 61470, 61471, 61474, 61477, 61478, 61483, 61484, 61486, 61489, 61491, 61496, 61504, 61506, 61509, 61511, 61513, 62110, 62226, 62294, 62295, 62431, 62471, 62482, 62575, 62691, 62757, 62807, 63018, 63068, 63078, 63133, 63160, 63226, 63244, 63255, 63266, 63316, 63448, 64020, 64030, 64055, 64076, 64163, 64164, 64169, 64225, 64248, 64278, 64333, 64384, 64402, 64497, 64553, 65008, 65027, 65092, 65122, 65188, 65192, 65227, 65246, 65282, 65365, 67004, 67007, 67014, 67015, 67041, 67042, 67064, 67114, 67153, 67157, 67158, 67170, 67171, 67193, 67207, 67211, 67219, 67224, 67243, 67251, 67262, 67284, 67294, 67297, 67316, 67332, 67372, 67374, 67376, 67390, 67393, 67399, 67402, 67418, 67419, 67431, 67439, 67457, 67469, 67495, 67496, 67512, 67533, 67539, 67549, 67560, 67561, 67562, 68006, 68012, 68031, 68056, 68070, 68108, 68133, 68143, 68162, 68164, 68201, 68206, 68219, 68233, 68240, 68272, 68310, 68314, 68319, 68320, 69015, 69019, 69024, 69025, 69041, 69048, 69066, 69073, 69101, 69114, 69128, 69129, 69135, 69144, 69146, 69147, 69150, 69157, 69158, 69159, 69179, 69185, 69195, 69208, 69210, 69211, 69213, 69221, 69222, 69223, 69224, 69228, 69237, 69247, 69248, 69251, 69255, 70033, 70034, 70068, 70108, 70123, 70131, 70139, 70173, 70191, 70209, 70223, 70241, 70245, 70246, 70281, 70300, 70333, 70345, 70346, 70354, 70370, 70382, 70391, 70418, 70434, 70458, 70465, 70475, 70489, 70497, 70551, 70570, 71055, 71134, 71180, 71260, 71279, 71288, 71304, 71375, 71432, 71511, 71582, 71587, 72023, 72025, 72063, 72069, 72071, 72080, 72081, 72082, 72097, 72108, 72116, 72117, 72128, 72137, 72138, 72159, 72162, 72176, 72189, 72203, 72207, 72240, 72258, 72262, 72284, 72288, 72301, 72304, 72308, 72318, 72363, 72384, 73002, 73003, 73006, 73010, 73035, 73038, 73045, 73046, 73056, 73060, 73062, 73080, 73093, 73102, 73108, 73111, 73112, 73115, 73118, 73126, 73127, 73135, 73143, 73144, 73150, 73151, 73158, 73163, 73167, 73169, 73174, 73185, 73187, 73198, 73199, 73203, 73209, 73212, 73215, 73227, 73235, 73236, 73238, 73239, 73244, 73257, 73260, 73263, 73264, 73266, 73284, 73287, 73290, 73295, 73305, 73321, 74010, 74011, 74022, 74084, 74093, 74113, 74115, 74120, 74123, 74167, 74182, 74187, 74204, 74217, 74245, 74268, 74270, 74275, 74282, 76003, 76027, 76031, 76034, 76037, 76041, 76044, 76073, 76078, 76080, 76081, 76089, 76098, 76127, 76137, 76145, 76146, 76164, 76191, 76215, 76248, 76258, 76267, 76276, 76277, 76289, 76301, 76310, 76326, 76337, 76376, 76401, 76444, 76466, 76476, 76496, 76525, 76539, 76607, 76618, 76625, 76639, 76643, 76659, 76674, 76696, 76701, 76704, 76713, 76729, 76742, 77017, 77028, 77166, 77170, 77189, 77213, 77299, 77316, 77324, 77334, 77346, 77351, 77375, 77399, 77433, 77491, 77497, 77499, 77502, 77504, 78158, 78251, 78320, 78503, 78524, 78551, 79004, 79005, 79006, 79013, 79014, 79021, 79026, 79027, 79028, 79030, 79033, 79035, 79039, 79041, 79043, 79044, 79051, 79052, 79053, 79054, 79061, 79063, 79064, 79065, 79068, 79072, 79073, 79075, 79077, 79083, 79093, 79098, 79099, 79105, 79107, 79113, 79143, 79155, 79168, 79171, 79173, 79174, 79178, 79179, 79181, 79182, 79185, 79186, 79187, 79188, 79192, 79193, 79196, 79199, 79217, 79219, 79222, 79224, 79232, 79233, 79237, 79240, 79247, 79260, 79261, 79264, 79272, 79275, 79279, 79280, 79282, 79285, 79288, 79292, 79296, 79314, 79317, 79318, 79321, 79323, 79324, 79325, 79327, 79328, 79330, 79333, 79334, 79344, 79353, 80007, 80111, 80175, 80209, 80257, 80289, 80295, 80363, 80382, 80394, 80398, 80419, 80441, 80447, 80454, 80457, 80483, 80485, 80492, 80505, 80509, 80510, 80532, 80551, 80564, 80581, 80594, 80608, 80610, 80612, 80621, 80625, 80689, 80712, 80731, 80739, 80745, 80761, 80768, 80816, 80817, 81057, 81062, 81091, 81113, 81153, 81226, 81233, 81241, 81260, 81296, 81301, 85008, 85009, 85019, 85027, 85030, 85043, 85044, 85048, 85052, 85063, 85068, 85069, 85079, 85080, 85084, 85090, 85091, 85107, 85122, 85146, 85150, 85152, 85154, 85162, 85165, 85168, 85177, 85180, 85197, 85203, 85212, 85213, 85217, 85219, 85224, 85230, 85257, 85263, 85270, 85272, 85275, 85279, 85299, 85302, 86019, 86021, 86030, 86043, 86053, 86056, 86060, 86067, 86071, 86082, 86101, 86115, 86123, 86146, 86155, 86166, 86179, 86185, 86188, 86199, 86208, 86212, 86216, 86219, 86240, 86245, 86259, 86267, 86277, 86278, 86281, 86283, 87026, 87028, 87055, 87097, 87098, 87128, 87136, 87171, 87173, 87175, 87184, 87196, 88018, 88029, 88067, 88112, 88204, 88218, 88234, 88235, 88337, 88361, 88420, 88465, 89001, 89003, 89047, 89052, 89070, 89078, 89086, 89090, 89097, 89103, 89107, 89109, 89114, 89130, 89135, 89138, 89140, 89157, 89166, 89174, 89178, 89192, 89196, 89197, 89213, 89231, 89241, 89243, 89260, 89275, 89293, 89294, 89317, 89322, 89330, 89334, 89340, 89343, 89356, 89357, 89358, 89366, 89372, 89381, 89388, 89389, 89401, 89405, 89411, 89420, 89421, 89429, 89435, 89448, 89454, 89457, 89473, 89476, 89484, 90068, 90073, 91182, 91222, 91228, 91390
```

```
head (ID,10)
```

```
##    annee_2020.Iden insee2020_reg insee2020_dep
## 1            01001            84            01
## 2            01002            84            01
## 3            01004            84            01
## 4            01005            84            01
## 5            01006            84            01
## 6            01007            84            01
## 7            01008            84            01
## 8            01009            84            01
## 9            01010            84            01
## 10           01011            84            01
```

```
##* ID STEP3: assign new variable from “lookup __match tables”----
##15- Fin assigns the “insee_REG” and “insee_DEP” variables to the “annee_2020” table, at the commune level ----
annee_2020$insee2020_reg <-ID$insee2020_reg
annee_2020$insee2020_dep <-ID$insee2020_dep


##*************************************************************
  ##16- LABEL STEP0, WE CREATE a DATAFRAME “LABEL” from “$insee_DEP” and “$insee_REG” of the table “annee_2020” ----
##Initial data:
##dat <- data.frame(HouseType = c("Semi", "Single", "Row", "Single", "Apartment", "Apartment", "Row"))
LABEL <-data.frame(annee_2020$insee2020_dep, annee_2020$insee2020_reg)
head (LABEL,10)
```

```
##    annee_2020.insee2020_dep annee_2020.insee2020_reg
## 1                        01                       84
## 2                        01                       84
## 3                        01                       84
## 4                        01                       84
## 5                        01                       84
## 6                        01                       84
## 7                        01                       84
## 8                        01                       84
## 9                        01                       84
## 10                       01                       84
```

```
##* LABEL STEP 1 _ Lookup / crosswalk table: Crosswalk table from dataframe “DEP_21” and “REG_21” from insee ----
#lookup <- data.frame(type_text = c("Semi", "Single", "Row", "Apartment"), type_num = c(1, 2, 3, 4))
#lookup <- data.frame(inseecom_2021$COM, inseecom_2021$REG, inseecom_2021$DEP)
lookup_dep <- data.frame(DEP_20$dep, DEP_20$libelle)
lookup_reg <- data.frame(REG_20$reg, REG_20$libelle)
head (lookup_dep,10)
```

```
##    DEP_20.dep          DEP_20.libelle
## 1          01                     Ain
## 2          02                   Aisne
## 3          03                  Allier
## 4          04 Alpes-de-Haute-Provence
## 5          05            Hautes-Alpes
## 6          06         Alpes-Maritimes
## 7          07                 Ardèche
## 8          08                Ardennes
## 9          09                  Ariège
## 10         10                    Aube
```

```
head (lookup_reg,10)
```

```
##    REG_20.reg          REG_20.libelle
## 1           1              Guadeloupe
## 2           2              Martinique
## 3           3                  Guyane
## 4           4              La Réunion
## 5           6                 Mayotte
## 6          11           Île-de-France
## 7          24     Centre-Val de Loire
## 8          27 Bourgogne-Franche-Comté
## 9          28               Normandie
## 10         32         Hauts-de-France
```

```
##* LABEL STEP2 Create a new variable in LABEL table with plyr::mapvalues:----
##dat$house_type_num <- plyr::mapvalues(dat$HouseType, from = lookup$type_text, to = lookup$type_num)
LABEL$insee2020_label_dep <- plyr::mapvalues(LABEL$annee_2020.insee2020_dep, from = lookup_dep$DEP_20.dep, to = lookup_dep$DEP_20.libelle)
LABEL$insee2020_label_reg <- plyr::mapvalues(LABEL$annee_2020.insee2020_reg, from = lookup_reg$REG_20.reg, to = lookup_reg$REG_20.libelle)
head (LABEL$insee2020_label_dep,10)
```

```
##  [1] "Ain" "Ain" "Ain" "Ain" "Ain" "Ain" "Ain" "Ain" "Ain" "Ain"
```

```
head (LABEL$insee2020_label_reg,10)
```

```
##  [1] "Auvergne-Rhône-Alpes" "Auvergne-Rhône-Alpes" "Auvergne-Rhône-Alpes"
##  [4] "Auvergne-Rhône-Alpes" "Auvergne-Rhône-Alpes" "Auvergne-Rhône-Alpes"
##  [7] "Auvergne-Rhône-Alpes" "Auvergne-Rhône-Alpes" "Auvergne-Rhône-Alpes"
## [10] "Auvergne-Rhône-Alpes"
```

```
##*  LABEL STEP3: assign a new variable from lookup __match tables----
annee_2020$insee2020_label_dep <- LABEL$insee2020_label_dep
annee_2020$insee2020_label_reg <- LABEL$insee2020_label_reg
head (annee_2020$insee2020_label_dep,10)
```

```
##  [1] "Ain" "Ain" "Ain" "Ain" "Ain" "Ain" "Ain" "Ain" "Ain" "Ain"
```

```
head (annee_2020$insee2020_label_reg,10)
```

```
##  [1] "Auvergne-Rhône-Alpes" "Auvergne-Rhône-Alpes" "Auvergne-Rhône-Alpes"
##  [4] "Auvergne-Rhône-Alpes" "Auvergne-Rhône-Alpes" "Auvergne-Rhône-Alpes"
##  [7] "Auvergne-Rhône-Alpes" "Auvergne-Rhône-Alpes" "Auvergne-Rhône-Alpes"
## [10] "Auvergne-Rhône-Alpes"
```

```
#17- Checking for missing values - for insee 2020 IDs: There are no missing values----
apply(annee_2020, 2, anyNA)
```

```
##                Iden               Iden1              ID_DEP          ID_COMMUNE 
##               FALSE               FALSE               FALSE               FALSE 
##         LIB_COMMUNE          POPULATION      POP_MUNICIPALE             DC_2018 
##               FALSE               FALSE               FALSE                TRUE 
##             DC_2019          DC_2018_19             DC_2020        SURMORTALITE 
##                TRUE               FALSE                TRUE               FALSE 
##           AGE_MOYEN            SM_10000        SMM_10000_R_              statut 
##               FALSE               FALSE               FALSE               FALSE 
##              PERIOD       insee2020_reg       insee2020_dep insee2020_label_dep 
##               FALSE               FALSE               FALSE               FALSE 
## insee2020_label_reg 
##               FALSE
```

```
# Missing values for the 3 variables “DC_2018”, “DC_2019”, “DC_2020” TRUE
val_manquantes_DC_2018_19_es <- which(annee_2020$DC_2018_19 %in% "")
val_manquantes_DC_2018_19_na <- which(annee_2020$DC_2018_19 %in% "NA")
# val_manquantes_DC_2019_bis <- which(annee_2020$DC_2019 %in% NA)
# val_manquantes_DC_2020_bis <- which(annee_2020$DC_2020 %in% NA)
val_manquantes_DC_2018_19_es <- NULL
val_manquantes_DC_2018_19_na <- NULL

############SEPARATION################################################################
#18- Check for missing values-- Create copy of “annee_2020” table ----
#* Missing value? replace type “” and NA----
mortalite_2020_x <- annee_2020 #rename file ? recode
#*search cell with empty values “”----

#*if empty cell replace with 'x' for philcarto----
mortalite_2020_x[mortalite_2020_x == "" ] <- 'x' #replace empty cell

############SEPARATION######################################################
##18- Faster verification, missing data NA----
#* Search for cells with NA values with the base::apply()---- package.
apply(mortalite_2020_x, 2, anyNA)
```

```
##                Iden               Iden1              ID_DEP          ID_COMMUNE 
##               FALSE               FALSE               FALSE               FALSE 
##         LIB_COMMUNE          POPULATION      POP_MUNICIPALE             DC_2018 
##               FALSE               FALSE               FALSE                TRUE 
##             DC_2019          DC_2018_19             DC_2020        SURMORTALITE 
##                TRUE               FALSE                TRUE               FALSE 
##           AGE_MOYEN            SM_10000        SMM_10000_R_              statut 
##               FALSE               FALSE               FALSE               FALSE 
##              PERIOD       insee2020_reg       insee2020_dep insee2020_label_dep 
##               FALSE               FALSE               FALSE               FALSE 
## insee2020_label_reg 
##               FALSE
```

```
#* if cell with NA, replace with 'x' for philcarto----
head (mortalite_2020_x, 10)
```

```
##    Iden     Iden1 ID_DEP ID_COMMUNE              LIB_COMMUNE POPULATION
## 1 01001 SVG_01001     01      01001 L' Abergement-Clémenciat        794
##   POP_MUNICIPALE DC_2018 DC_2019 DC_2018_19 DC_2020 SURMORTALITE
## 1            776       1       0        0,5       1          0,5
##          AGE_MOYEN SM_10000 SMM_10000_R_         statut PERIOD insee2020_reg
## 1 76,3936042054539      6,3         6,44 Commune simple      0            84
##   insee2020_dep insee2020_label_dep  insee2020_label_reg
## 1            01                 Ain Auvergne-Rhône-Alpes
##  [ reached 'max' / getOption("max.print") -- omitted 9 rows ]
```

```
mortalite_2020_x[is.na(mortalite_2020_x)] <- 'x'   #replace cell with NA

# ##############################################################"
#*package base - fonction apply()----
base::apply(mortalite_2020_x, 2, anyNA)
```

```
##                Iden               Iden1              ID_DEP          ID_COMMUNE 
##               FALSE               FALSE               FALSE               FALSE 
##         LIB_COMMUNE          POPULATION      POP_MUNICIPALE             DC_2018 
##               FALSE               FALSE               FALSE               FALSE 
##             DC_2019          DC_2018_19             DC_2020        SURMORTALITE 
##               FALSE               FALSE               FALSE               FALSE 
##           AGE_MOYEN            SM_10000        SMM_10000_R_              statut 
##               FALSE               FALSE               FALSE               FALSE 
##              PERIOD       insee2020_reg       insee2020_dep insee2020_label_dep 
##               FALSE               FALSE               FALSE               FALSE 
## insee2020_label_reg 
##               FALSE
```

```
??apply()
```

```
## démarrage du serveur d'aide httpd ...
```

```
##  fini
```

```
#############SEPARATION##################################################
##################3??????????????????????????????????????????????
#19- Check variable type, then format variable names----
str(mortalite_2020_x)
```

```
## 'data.frame':    140052 obs. of  21 variables:
##  $ Iden               : chr  "01001" "01002" "01004" "01005" ...
##  $ Iden1              : chr  "SVG_01001" "SVG_01002" "SVG_01004" "SVG_01005" ...
##  $ ID_DEP             : chr  "01" "01" "01" "01" ...
##  $ ID_COMMUNE         : chr  "01001" "01002" "01004" "01005" ...
##  $ LIB_COMMUNE        : chr  "L' Abergement-Clémenciat" "L' Abergement-de-Varey" "Ambérieu-en-Bugey" "Ambérieux-en-Dombes" ...
##  $ POPULATION         : int  794 249 14428 1723 117 2841 767 339 1132 387 ...
##  $ POP_MUNICIPALE     : int  776 248 14035 1689 111 2726 752 330 1115 376 ...
##  $ DC_2018            : chr  "1" "x" "34" "1" ...
##  $ DC_2019            : chr  "0" "x" "43" "0" ...
##  $ DC_2018_19         : chr  "0,5" "x" "38,5" "0,5" ...
##  $ DC_2020            : chr  "1" "x" "36" "1" ...
##  $ SURMORTALITE       : chr  "0,5" "x" "-2,5" "0,5" ...
##  $ AGE_MOYEN          : chr  "76,3936042054539" "x" "83,7593089475413" "51,092432373234" ...
##  $ SM_10000           : chr  "6,3" "x" "-1,73" "2,9" ...
##  $ SMM_10000_R_       : chr  "6,44" "x" "-1,78" "2,96" ...
##  $ statut             : chr  "Commune simple" "Commune simple" "Commune simple" "Commune simple" ...
##  $ PERIOD             : int  0 0 0 0 0 0 0 0 0 0 ...
##  $ insee2020_reg      : chr  "84" "84" "84" "84" ...
##  $ insee2020_dep      : chr  "01" "01" "01" "01" ...
##  $ insee2020_label_dep: chr  "Ain" "Ain" "Ain" "Ain" ...
##  $ insee2020_label_reg: chr  "Auvergne-Rhône-Alpes" "Auvergne-Rhône-Alpes" "Auvergne-Rhône-Alpes" "Auvergne-Rhône-Alpes" ...
```

```
head(mortalite_2020_x, 20)
```

```
##    Iden     Iden1 ID_DEP ID_COMMUNE              LIB_COMMUNE POPULATION
## 1 01001 SVG_01001     01      01001 L' Abergement-Clémenciat        794
##   POP_MUNICIPALE DC_2018 DC_2019 DC_2018_19 DC_2020 SURMORTALITE
## 1            776       1       0        0,5       1          0,5
##          AGE_MOYEN SM_10000 SMM_10000_R_         statut PERIOD insee2020_reg
## 1 76,3936042054539      6,3         6,44 Commune simple      0            84
##   insee2020_dep insee2020_label_dep  insee2020_label_reg
## 1            01                 Ain Auvergne-Rhône-Alpes
##  [ reached 'max' / getOption("max.print") -- omitted 19 rows ]
```

```
#*- Copy, paste “PERIOD”.... variables INTO “PERIOD_N_” ...----
annee_2020$PERIOD_N_ <- annee_2020$PERIOD

#*- Copy, paste “PERIOD”.... variables INTO “PERIOD_N_” ...----
#*#*- Copy, paste “statut”.... variables INTO “statut_N_” ...----
mortalite_2020_x$PERIOD_N_ <- mortalite_2020_x$PERIOD
mortalite_2020_x$statut_N_ <- mortalite_2020_x$statut

#* Copy and paste the “SURMORTALITY” variable into “SURMORTALITY_R_” ----
mortalite_2020_x$SURMORTALITE_R_ <- mortalite_2020_x$SURMORTALITE


#******************
##20- Change variable type “PERIOD_N_” to FACTOR (qualitative variable)----
#*See Philcarto: quick start guide.pdf, New: suffix after variable name----
#*_R_ for ratio, _Q_ for quantitative and _N_ for categorical-factor-qualitative----
mortalite_2020_x$PERIOD_N_ <- as.factor(mortalite_2020_x$PERIOD_N_)


###################SEPARATION##########################################################################
##21-recall of departments “97” corresponds to the 5 departments of the French West Indies: “971”, “972”, “973”, “974” and “975” ----
##* create the “ID_REG” variable ----
##22- Mortality France 2020 with 4 periods ----
#* P0  0=>[01/01,01/03[,----
#* P1  1=>[01/03,16/05[,----
#* P2  2=>[16/05,29/10[,----
#* P3  3=>[29/10,31/12]----

##23- 12 files (3*4) of Mortality France 2020 ARRONDISSEMENTS with 4 periods----
###**An extraction of sub-files by “PERIOD and ARRONDISSEMENT” ----
#* arrondissement of Marseille - Marseille district----
mortalite_2020_x_P0_arrdts <- NULL
mortalite_2020_x_P0_A_Marseille<-subset(mortalite_2020_x, PERIOD == 0 & Iden >= 13201 & Iden < 13217)
mortalite_2020_x_P1_A_Marseille<-subset(mortalite_2020_x, PERIOD == 1 & Iden >= 13201 & Iden < 13217)
mortalite_2020_x_P2_A_Marseille<-subset(mortalite_2020_x, PERIOD == 2 & Iden >= 13201 & Iden < 13217)
mortalite_2020_x_P3_A_Marseille<-subset(mortalite_2020_x, PERIOD == 3 & Iden >= 13201 & Iden < 13217)
#* arrondissement of Paris - Paris district--------
mortalite_2020_x_P0_A_Paris    <-subset(mortalite_2020_x, PERIOD == 0 & Iden >= 75101 & Iden < 75121)
mortalite_2020_x_P1_A_Paris    <-subset(mortalite_2020_x, PERIOD == 1 & Iden >= 75101 & Iden < 75121)
mortalite_2020_x_P2_A_Paris    <-subset(mortalite_2020_x, PERIOD == 2 & Iden >= 75101 & Iden < 75121)
mortalite_2020_x_P3_A_Paris    <-subset(mortalite_2020_x, PERIOD == 3 & Iden >= 75101 & Iden < 75121)
#* arrondissement of Lyon - Lyon district--------
mortalite_2020_x_P0_A_Lyon    <-subset(mortalite_2020_x, PERIOD == 0 & Iden >= 69381 & Iden < 69390)
mortalite_2020_x_P1_A_Lyon    <-subset(mortalite_2020_x, PERIOD == 1 & Iden >= 69381 & Iden < 69390)
mortalite_2020_x_P2_A_Lyon    <-subset(mortalite_2020_x, PERIOD == 2 & Iden >= 69381 & Iden < 69390)
mortalite_2020_x_P3_A_Lyon    <-subset(mortalite_2020_x, PERIOD == 3 & Iden >= 69381 & Iden < 69390)


#*#* Checking extractions ----
head(mortalite_2020_x_P0_A_Lyon)
```

```
##        Iden     Iden1 ID_DEP ID_COMMUNE             LIB_COMMUNE POPULATION
## 27136 69381 SVG_69381     69      69381 Lyon 1er Arrondissement      30142
##       POP_MUNICIPALE DC_2018 DC_2019 DC_2018_19 DC_2020 SURMORTALITE
## 27136          29352      17      19       18,0      16         -2,0
##              AGE_MOYEN SM_10000 SMM_10000_R_                   statut PERIOD
## 27136 84,3546366772533    -0,66        -0,68 Arrondissement municipal      0
##       insee2020_reg insee2020_dep insee2020_label_dep  insee2020_label_reg
## 27136            84            69               Rhône Auvergne-Rhône-Alpes
##       PERIOD_N_                statut_N_ SURMORTALITE_R_
## 27136         0 Arrondissement municipal            -2,0
##  [ reached 'max' / getOption("max.print") -- omitted 5 rows ]
```

```
head(mortalite_2020_x_P0_A_Paris)
```

```
##        Iden     Iden1 ID_DEP ID_COMMUNE              LIB_COMMUNE POPULATION
## 29295 75101 SVG_75101     75      75101 Paris 1er Arrondissement      16395
##       POP_MUNICIPALE DC_2018 DC_2019 DC_2018_19 DC_2020 SURMORTALITE
## 29295          16266       8       4        6,0       7          1,0
##              AGE_MOYEN SM_10000 SMM_10000_R_                   statut PERIOD
## 29295 58,2345072515919     0,61         0,61 Arrondissement municipal      0
##       insee2020_reg insee2020_dep insee2020_label_dep insee2020_label_reg
## 29295            11            75               Paris       Île-de-France
##       PERIOD_N_                statut_N_ SURMORTALITE_R_
## 29295         0 Arrondissement municipal             1,0
##  [ reached 'max' / getOption("max.print") -- omitted 5 rows ]
```

```
head(mortalite_2020_x_P0_A_Marseille)
```

```
##       Iden     Iden1 ID_DEP ID_COMMUNE                  LIB_COMMUNE POPULATION
## 4350 13201 SVG_13201     13      13201 Marseille 1er Arrondissement      39967
##      POP_MUNICIPALE DC_2018 DC_2019 DC_2018_19 DC_2020 SURMORTALITE
## 4350          39786      12      13       12,5      18          5,5
##             AGE_MOYEN SM_10000 SMM_10000_R_                   statut PERIOD
## 4350 77,3335683386266     1,38         1,38 Arrondissement municipal      0
##      insee2020_reg insee2020_dep insee2020_label_dep        insee2020_label_reg
## 4350            93            13    Bouches-du-Rhône Provence-Alpes-Côte d'Azur
##      PERIOD_N_                statut_N_ SURMORTALITE_R_
## 4350         0 Arrondissement municipal             5,5
##  [ reached 'max' / getOption("max.print") -- omitted 5 rows ]
```

```
### End of sub-file extractions by “PERIOD and AREA” ----
#2- Create the function encompassing the actions ----
#**Action1: loop ### extraction of 4 periods 0, 1, 2 and 3, for 18 regions: 4*18 = 72 files----


#** Action 2: create or update list with extracted files ----
dfs_4P_arrdt <- list(mortalite_2020_x_P0_A_Marseille, mortalite_2020_x_P1_A_Marseille, mortalite_2020_x_P2_A_Marseille, mortalite_2020_x_P3_A_Marseille,
                     mortalite_2020_x_P0_A_Lyon ,    mortalite_2020_x_P1_A_Lyon ,mortalite_2020_x_P2_A_Lyon ,           mortalite_2020_x_P3_A_Lyon ,
                     mortalite_2020_x_P0_A_Paris,    mortalite_2020_x_P1_A_Paris, mortalite_2020_x_P2_A_Paris,            mortalite_2020_x_P3_A_Paris)

paths_4P_arrdt <- c("mortalite_2020_x_P0_A_Marseille.xlsx"
                    ,"mortalite_2020_x_P1_A_Marseille.xlsx"
                    ,"mortalite_2020_x_P2_A_Marseille.xlsx"
                    ," mortalite_2020_x_P3_A_Marseille.xlsx"
                    ,"mortalite_2020_x_P0_A_Lyon.xlsx"
                    , "mortalite_2020_x_P1_A_Lyon.xlsx"
                    ,"mortalite_2020_x_P2_A_Lyon.xlsx"
                    ,"mortalite_2020_x_P3_A_Lyon.xlsx"
                    , "mortalite_2020_x_P0_A_Paris.xlsx"
                    , "mortalite_2020_x_P1_A_Paris.xlsx"
                    , "mortalite_2020_x_P2_A_Paris.xlsx"
                    , "mortalite_2020_x_P3_A_Paris.xlsx")


a<- data.frame("P0_A_13","P1_A_13", "P2_A_13",  "P3_A_13", 
               "P0_A_69",     "P1_A_69",      "P2_A_69",       "P3_A_69",
               "P0_A_75",    "P1_A_75",     "P2_A_75",      "P3_A_75")

b <- data.frame("_Marseille","_Marseille", "_Marseille",  "_Marseille", 
                "_Lyon",     "_Lyon",      "_Lyon",       "_Lyon",
                "_Paris",    "_Paris",     "_Paris",      "_Paris")

etiquette<-data.frame(labelarrdt=c(paste(a,b,sep="")))

str(etiquette)
```

```
## 'data.frame':    12 obs. of  1 variable:
##  $ labelarrdt: chr  "P0_A_13_Marseille" "P1_A_13_Marseille" "P2_A_13_Marseille" "P3_A_13_Marseille" ...
```

```
#feuilles_P0 <- data.frame(feuille_carto=c("mortalite_2020_x_P0_R1", "mortalite_2020_x_P0_R2", "mortalite_2020_x_P0_R3","mortalite_2020_x_P0_R4", 
#                                         "mortalite_2020_x_P0_R6", "mortalite_2020_x_P0_R11","mortalite_2020_x_P0_R24","mortalite_2020_x_P0_R27",
#                                        "mortalite_2020_x_P0_R28","mortalite_2020_x_P0_R32","mortalite_2020_x_P0_R44","mortalite_2020_x_P0_R52",
#                                       "mortalite_2020_x_P0_R53", "mortalite_2020_x_P0_R75","mortalite_2020_x_P0_R76","mortalite_2020_x_P0_R84",
#                                      "mortalite_2020_x_P0_R93","mortalite_2020_x_P0_R94")) 


#3- Action 3: loop to export the 72 files extracted and put in a list by period named 'dfs_P0', dfs_P0 and dfs_P0 ----
#https://stackoverflow.com/questions/62270061/r-for-loop-write-csv-with-list-of-dataframes-and-path-names

#* install library(openxlsx)----
library(openxlsx)


for (i in 1:length(dfs_4P_arrdt)) {
  if(length(dfs_4P_arrdt) != length(paths_4P_arrdt)){stop("mismatch in number of data frames and paths")}
  openxlsx::write.xlsx(dfs_4P_arrdt[[i]], file = paths_4P_arrdt[i], rowNames = FALSE, colNames = TRUE, sheetName = etiquette$labelarrdt[i],fileEncoding = "UTF-8")
}


#######################################################

# #summary(warnings())
# The code example for the loop ------
#https://stackoverflow.com/questions/62270061/r-for-loop-write-csv-with-list-of-dataframes-and-path-names
# df1 <- data.frame(num = sample(1:10, 6, replace = TRUE), letter = sample(LETTERS[1:2], 6, replace = 
#                                                                            TRUE))
# df2 <- data.frame(num = sample(150:160, 6, replace = TRUE), letter = sample(LETTERS[1:2], 6, replace 
#                                                                             = TRUE))
# 
# list_dfs <- list(df1,df2)
# 
# paths <- c("C:\\Export\\file1.csv","C:\\Export\\file2.csv")
# 
# for (i in 1:length(list_dfs)) {
#   for (f in 1:length(paths)) {
#     write.csv(list_dfs[i], file = paths[f], row.names = FALSE)
#   }
# }

#*With this writing - it doesn't come out P3_A_Marseille.xlsx ----
# paths_4P_arrdt <- c(" C:/Users/aques/Desktop/_Decembre_21/R_data_ttt_2020/mortalite_2020_x_P0_A_Marseille.xlsx"
#                     ,"C:/Users/aques/Desktop/_Decembre_21/R_data_ttt_2020/mortalite_2020_x_P1_A_Marseille.xlsx"
#                     ,"C:/Users/aques/Desktop/_Decembre_21/R_data_ttt_2020/mortalite_2020_x_P2_A_Marseille.xlsx"
#                     ,"C:/Users/aques/Desktop/_Decembre_21/R_data_ttt_2020/mortalite_2020_x_P3_A_Marseille.xlsx"
#                     ,"C:/Users/aques/Desktop/_Decembre_21/R_data_ttt_2020/mortalite_2020_x_P0_A_Lyon.xlsx"
#                     ,"C:/Users/aques/Desktop/_Decembre_21/R_data_ttt_2020/mortalite_2020_x_P1_A_Lyon.xlsx"
#                     ,"C:/Users/aques/Desktop/_Decembre_21/R_data_ttt_2020/mortalite_2020_x_P2_A_Lyon.xlsx"
#                     ,"C:/Users/aques/Desktop/_Decembre_21/R_data_ttt_2020/mortalite_2020_x_P3_A_Lyon.xlsx"
#                     ,"C:/Users/aques/Desktop/_Decembre_21/R_data_ttt_2020/mortalite_2020_x_P0_A_Paris.xlsx"
#                     ,"C:/Users/aques/Desktop/_Decembre_21/R_data_ttt_2020/mortalite_2020_x_P1_A_Paris.xlsx"
#                     ,"C:/Users/aques/Desktop/_Decembre_21/R_data_ttt_2020/mortalite_2020_x_P2_A_Paris.xlsx"
#                     ,"C:/Users/aques/Desktop/_Decembre_21/R_data_ttt_2020/mortalite_2020_x_P3_A_Paris.xlsx"
#                     )
```
