# Supplementary material for: Relevance of the uMap Collaborative Platform as Support for Choropleth Mapping of a Traffic-Light Statistical Signal Atlas of All-Cause Mortality During the First French Lockdown: Geospatial Analysis
Source: JMIR Med Inform. 2026 Aug 27;14:e82855. doi: 10.2196/82855 (PMC13543287; doi:10.2196/82855)
Supplement: Supplementary file 4 [file medinform-v14-e82855-s004.zip › MA-SF_ready-to-useMortalityDataForR/SF1_supplementary_file_1.pdf]

# atlas6_utf8_insee-annee2020_arrdts_loop_output.R

Anne Barbet

27/08/2024

```
##script -- atlas6_utf8_insee-annee2020_arrdts_loop_output-----
## An example to export 12 district files----
##
##updated August 27, 2024----

#rm(list=ls())
getwd()
```

```
## [1] "C:/Users/anne.barbet/Desktop/_Decembre_21/R_data_ttt_2020/_src"
```

```
#[1] "C:/Users/anne.barbet/Desktop/_Decembre_21/R_data_ttt_2020"

options(max.print=30)
#packages
#* install library(openxlsx)----
library(openxlsx)
##use this package for V search or lookup - excel equivalent
#install.packages("plyr", dependencies = TRUE)
library(plyr)
#??mapvalues()

#######"*********************************
#*****************************************
##New insee extraction dated July 23, 2021
##** The second file to be processed is dated December 12, 2021 ----
#**file with "arrondissements"----
#** The third file is dated December 14, 2021 ----
## 1- Import file france mortalite 2020----
##*recuperate variables from insee 2021 database:  COM, DEP and REG ----
##variable ID_DEP and DEP_LABEL
##variable ID_region and REG_LABEL


##2- Import file (December 12, 2021) ----
annee_2020 <- read.csv("C:/Users/anne.barbet/Desktop/_Decembre_21/R_data_ttt_2020/_data/from_
thipages_12dec21/mortalite_france_2020vs2018-19_4periodes.csv", header = TRUE, sep = ",",
                       dec = ".", stringsAsFactors = FALSE, encoding="UTF-8")


#*#https://delladata.fr/exporter-donnees-sorties-r/
#**-to create the folder data_output----
#dir.create("output_csv")
#dir.create("./_data/from_thipages_14dec21_Arrdts")


##4- Import of the third version of Dec. 14, 21, databases of Paris, Lyon, Marseille detailed
districts----
annee_2020_arrdts <- read.csv("C:/Users/anne.barbet/Desktop/_Decembre_21/R_data_ttt_2020/_dat
a/from_thipages_14dec21_Arrdts/mortalite_france_2020vs2018-19_4periodes_arrondisements.csv",
header = TRUE, sep = ",",
                      dec = ".", stringsAsFactors = FALSE, encoding="UTF-8")

#head (annee_2020_arrdts, 180)

##5-Import official insee2020 geographic code: https://www.insee.fr/fr/information/4316069---
-
inseecom_2020 <- read.csv("C:/Users/anne.barbet/Desktop/_Decembre_21/R_data_ttt_2020/_data/in
see_2020/communes2020-csv/communes2020.csv", header = TRUE, sep = ",",
                           dec = ".", stringsAsFactors = FALSE, encoding="UTF-8")

#*#*****************************************
##Official geographic code as of January 1, 2021 ----
##https://www.insee.fr/fr/information/5057840----
##commune2021.csv downloaded December 02, 2021----
#*****************************************
```

```
##6-Import departement2020.csv----
DEP_20 <-read.csv("C:/Users/anne.barbet/Desktop/_Decembre_21/R_data_ttt_2020/_data/insee_202
0/departement2020-csv/departement2020.csv", header = TRUE, sep = ",",
                  dec = ",", stringsAsFactors = FALSE, encoding="UTF-8")
head(DEP_20 , 2)
```

```
##   dep reg cheflieu tncc   ncc nccenr libelle
## 1  01  84    01053    5   AIN    Ain     Ain
## 2  02  32    02408    5 AISNE  Aisne   Aisne
```

```
##7-Import region2020.csv----
REG_20 <-read.csv("C:/Users/anne.barbet/Desktop/_Decembre_21/R_data_ttt_2020/_data/insee_202
0/region2020-csv/region2020.csv", header = TRUE, sep = ",",
                  dec = ",", stringsAsFactors = FALSE, encoding="UTF-8")
head(REG_20 , 2)
```

```
##   reg cheflieu tncc        ncc     nccenr    libelle
## 1   1    97105    3 GUADELOUPE Guadeloupe Guadeloupe
## 2   2    97209    3 MARTINIQUE Martinique Martinique
```

```
##verif tableau d?but 10 lignes
##** correct ID_DEP, it is not ID Dept but and ID communes----
##verification table start 10 lines
head(REG_20, 18)
```

```
##   reg cheflieu tncc        ncc     nccenr    libelle
## 1   1    97105    3 GUADELOUPE Guadeloupe Guadeloupe
## 2   2    97209    3 MARTINIQUE Martinique Martinique
## 3   3    97302    3     GUYANE     Guyane     Guyane
## 4   4    97411    0 LA REUNION La Réunion La Réunion
## 5   6    97608    0    MAYOTTE    Mayotte    Mayotte
##  [ reached 'max' / getOption("max.print") -- omitted 13 rows ]
```

```
##SEPARATION#############
# rename variable
#names(df)[names(df) == 'old.var.name'] <- 'new.var.name'



#*##8-Verify unique values of variable ID_COMMUNE----
#*verify number year_2020$Iden unique : 35013 via function : unique()----
compte_2020 <- unique(annee_2020$Iden)
annee_2020$Iden
```

```
##  [1] "01001" "01002" "01004" "01005" "01006" "01007" "01008" "01009" "01010"
## [10] "01011" "01012" "01013" "01014" "01016" "01015" "01017" "01019" "01021"
## [19] "01022" "01453" "01023" "01024" "01025" "01026" "01027" "01028" "01170"
## [28] "01029" "01030" "01032"
##  [ reached getOption("max.print") -- omitted 140022 entries ]
```

```
##verification number inseecom_2020$com unique : 37235 via function : unique()
compteinsee_2020 <- unique(inseecom_2020$com)


##*rename var header SMM_10000 AS SMM_10000_R_ for better management ----
## of variables in philcarto
##(_R_ for a ratio variable, _N_ for a nominal variable, and _Q_ for a ##quantity variable).
 ##quantity variable)
names(annee_2020)[names(annee_2020) == 'SMM_10000'] <- 'SMM_10000_R_'


#*??paste()----
## compare two tables
##d2[!paste(d2$x1, d2$x2)%in%paste(d1$x1, d1$x2),]
comp1VAR_insee_2020 <- data.frame(inseecom_2020[!paste(inseecom_2020$com)%in%paste(annee_2020
$Iden),])


## 9-Comparing two tables----
#d2[!paste(d2$x1, d2$x2)%in%paste(d1$x1, d1$x2),]
comp2VAR_annee_2020 <- data.frame(annee_2020[!paste(annee_2020$Iden)%in%paste(inseecom_2020$c
om),])


##*##10-Verify, compare and delete both df----
##*delete a dataframe or other object----
rm(comp1VAR_insee_2020)
rm(comp2VAR_annee_2020)


########## SEPARATION ###########
# CREATE VAR SVG_
# CONCATENER SVG_ & ID_COM
# function paste(), paste0
#https://stt4230.rbind.io/manipulation_donnees/structures_r/#fonctions-paste-et-paste0


#11-CREATE A VARIABLE WITH SVG----
#color <- c(“Yellow”, “Yellow”, “Red”, “Green”)
annee_2020$SVG <- c("SVG")

head (annee_2020$SVG,10)
```

```
##  [1] "SVG" "SVG" "SVG" "SVG" "SVG" "SVG" "SVG" "SVG" "SVG" "SVG"
```

```
#*#12- Concatener SVG variable and ID_COM variable with separator _ underscore ----
#*paste(LETTERS, letters, sep = "_")----
annee_2020$Iden1 <- paste(annee_2020$SVG, annee_2020$ID_COMMUNE, sep = "_")
#*deletes bad variable Iden----
annee_2020$SVG <- NULL



##13- Reorganize dataframe move column----
#https://stackoverflow.com/questions/3369959/moving-columns-within-a-data-frame-without-retyp
ing/18540144#18540144
#for example: ordered_columns_leftside=c('var10','var34','var8')
#df=df[c(ordered_columns_leftside, setdiff(names(df),ordered_columns_leftside))]


#* file reordered, to obtain column "Iden" in first position, Iden1 in 2nd position, the ID i
s adapted to the IDs of the map fund----
#WGS84 for March 2021, allowing output in .svg and .kml formats from philcarto
#setdiff()
names(annee_2020)
```

```
##  [1] "Iden"           "ID_DEP"         "ID_COMMUNE"     "LIB_COMMUNE"
##  [5] "POPULATION"     "POP_MUNICIPALE" "DC_2018"        "DC_2019"
##  [9] "DC_2018_19"     "DC_2020"        "SURMORTALITE"   "AGE_MOYEN"
## [13] "SM_10000"       "SMM_10000_R_"   "statut"         "PERIOD"
## [17] "Iden1"
```

```
ordered_columns_leftside=c('Iden','Iden1','ID_DEP','ID_COMMUNE','LIB_COMMUNE')
annee_2020=annee_2020[c(ordered_columns_leftside, setdiff(names(annee_2020),ordered_columns_l
eftside))]


#*#14- After comparison (line 58) output comp1VAR_annee, 1 value is different----
#*between the two tables compared the ID 27676 commune: "Les Trois Lacs"----
annee_2020$ID_COMMUNE == '01001'
```

```
##  [1]  TRUE FALSE FALSE FALSE FALSE FALSE FALSE FALSE FALSE FALSE FALSE FALSE
## [13] FALSE FALSE FALSE FALSE FALSE FALSE FALSE FALSE FALSE FALSE FALSE FALSE
## [25] FALSE FALSE FALSE FALSE FALSE FALSE
##  [ reached getOption("max.print") -- omitted 140022 entries ]
```

```
annee_2020$ID_COMMUNE == '14666'
```

```
##  [1] FALSE FALSE FALSE FALSE FALSE FALSE FALSE FALSE FALSE FALSE FALSE FALSE
## [13] FALSE FALSE FALSE FALSE FALSE FALSE FALSE FALSE FALSE FALSE FALSE FALSE
## [25] FALSE FALSE FALSE FALSE FALSE FALSE
##  [ reached getOption("max.print") -- omitted 140022 entries ]
```

```
annee_2020[34999:35013, 1]
```

```
##  [1] "97607" "97608" "97609" "97610" "97611" "97612" "97613" "97614" "97615"
## [10] "97616" "97617" "14666" "69123" "75056" "13055"
```

```
#??dim()
# AT THE END OF PRINT, not in chronological order for the four Identities
# “14666” “69123” “75056” “13055”
#14666 = commune of “Sannerville”, region, 28 Normandy, department 14 of “Calvados”
#commune restored on 31/12/2019

# #[14986] "97417" "97416" "97418" "97419" "97420" "97421" "97422" "97423" "97601"
# [14995] "97602" "97603" "97604" "97605" "97606" "97607" "97608" "97609" "97610"
# [15004] "97611" "97612" "97613" "97614" "97615" "97616" "97617" "14666" "69123"
# [15013] "75056" "13055"
#


##https://stackoverflow.com/questions/15303283/how-to-do-vlookup-and-fill-down-like-in-excel-
in-r
##15- Vertical search for a method among 4----

##* ID STEP0, we start by creating the “ID” dataframe from annee_2020$ID_COMMUNE----
##*Initial data:----
##dat <- data.frame(HouseType = c("Semi", "Single", "Row", "Single", "Apartment", "Apartmen
t", "Row"))
ID <-data.frame(annee_2020$Iden)
head (annee_2020$ID_COMMUNE,10)
```

```
##  [1] "01001" "01002" "01004" "01005" "01006" "01007" "01008" "01009" "01010"
## [10] "01011"
```

```
#LABEL <-data.frame(annee_2020$insee_DEP, annee_2020$insee_REG)

##* ID STEP1 Lookup / crosswalk table TABLE DES CORRESPONDANCES MAILLE COMMUNE 3 variables IN
SEE "COM", "REG", "DEP":----
##Correspondence table, communal grid, 3 INSEE variables: “COM”, “REG”, “DEP”:----
#lookup <- data.frame(type_text = c("Semi", "Single", "Row", "Apartment"), type_num = c(1, 2,
3, 4))
lookup_com <- data.frame(inseecom_2020$com, inseecom_2020$reg, inseecom_2020$dep)
head (lookup_com,10)
```

```
##    inseecom_2020.com inseecom_2020.reg inseecom_2020.dep
## 1              01001                84                01
## 2              01002                84                01
## 3              01004                84                01
## 4              01005                84                01
## 5              01006                84                01
## 6              01007                84                01
## 7              01008                84                01
## 8              01009                84                01
## 9              01010                84                01
## 10             01011                84                01
```

```
#lookup_dep <- data.frame(DEP_21$DEP, DEP_21$LIBELLE)
#lookup_reg <- data.frame(REG_21$REG, REG_21$LIBELLE)

##* ID STEP2 Create the new variableS insee_REG & insee_DEP in DATAFRAME "ID":----
##dat$house_type_num <- plyr::mapvalues(dat$HouseType, from = lookup$type_text, to = lookup$t
ype_num)
ID$insee2020_reg <- plyr::mapvalues(ID$annee_2020.Iden, from = lookup_com$inseecom_2020.com,
to = lookup_com$inseecom_2020.reg)
```

```
## The following `from` values were not present in `x`: 01015, 01025, 01033, 01036, 01059, 01
080, 01091, 01095, 01097, 01098, 01119, 01120, 01122, 01130, 01137, 01144, 01154, 01172, 0117
6, 01182, 01185, 01186, 01187, 01204, 01205, 01215, 01218, 01221, 01227, 01271, 01286, 01292,
01300, 01312, 01316, 01324, 01338, 01340, 01341, 01409, 01413, 01414, 01417, 01426, 01442, 01
453, 02018, 02026, 02053, 02147, 02153, 02161, 02285, 02300, 02301, 02325, 02348, 02360, 0243
4, 02439, 02458, 02475, 02479, 02597, 02630, 02646, 02669, 02733, 02771, 02811, 02825, 03123,
03153, 03158, 03168, 03318, 04003, 04010, 04011, 04014, 04029, 04033, 04052, 04082, 04100, 04
105, 04120, 04131, 04153, 04165, 04198, 05001, 05002, 05005, 05020, 05024, 05034, 05042, 0505
3, 05067, 05069, 05088, 05101, 05118, 05120, 05125, 05132, 05138, 05139, 05143, 05150, 05175,
07011, 07016, 07043, 07046, 07057, 07135, 07164, 07165, 07246, 07256, 07262, 07271, 08007, 08
009, 08012, 08042, 08050, 08051, 08053, 08072, 08079, 08112, 08114, 08115, 08116, 08145, 0815
2, 08173, 08177, 08198, 08213, 08258, 08261, 08265, 08266, 08267, 08285, 08311, 08337, 08345,
08359, 08371, 08441, 08443, 08475, 08490, 08491, 08493, 08495, 09028, 09036, 09062, 09135, 09
286, 09296, 09302, 09317, 09334, 10003, 10036, 10277, 10311, 10415, 11050, 11097, 11131, 1117
1, 11251, 11298, 11323, 11329, 11349, 12005, 12014, 12020, 12021, 12040, 12076, 12081, 12087,
12090, 12112, 12114, 12117, 12120, 12123, 12126, 12132, 12173, 12177, 12196, 12218, 12223, 12
224, 12245, 12270, 12271, 12279, 12285, 12304, 14002, 14004, 14005, 14008, 14011, 14013, 1401
7, 14027, 14028, 14029, 14031, 14035, 14037, 14051, 14052, 14056, 14058, 14061, 14065, 14073,
14074, 14075, 14081, 14098, 14099, 14105, 14109, 14112, 14113, 14114, 14115, 14128, 14129, 14
139, 14142, 14143, 14144, 14148, 14150, 14151, 14152, 14153, 14154, 14155, 14156, 14157, 1415
8, 14164, 14170, 14174, 14176, 14178, 14186, 14187, 14188, 14189, 14192, 14200, 14201, 14208,
14210, 14212, 14213, 14217, 14219, 14222, 14235, 14247, 14253, 14255, 14259, 14264, 14265, 14
268, 14279, 14281, 14292, 14294, 14296, 14313, 14317, 14321, 14324, 14330, 14331, 14339, 1434
0, 14342, 14347, 14349, 14350, 14355, 14356, 14357, 14359, 14361, 14371, 14372, 14373, 14376,
14382, 14386, 14387, 14388, 14395, 14406, 14410, 14413, 14414, 14415, 14416, 14417, 14418, 14
420, 14422, 14423, 14429, 14431, 14432, 14433, 14440, 14441, 14442, 14443, 14444, 14450, 1445
6, 14459, 14462, 14463, 14471, 14472, 14474, 14475, 14477, 14481, 14489, 14493, 14503, 14505,
14508, 14517, 14518, 14521, 14523, 14525, 14527, 14532, 14537, 14538, 14539, 14543, 14544, 14
545, 14548, 14549, 14551, 14553, 14554, 14560, 14564, 14568, 14570, 14573, 14576, 14577, 1457
9, 14580, 14581, 14583, 14584, 14585, 14591, 14596, 14597, 14599, 14600, 14608, 14611, 14615,
14616, 14618, 14628, 14629, 14632, 14633, 14634, 14636, 14638, 14642, 14647, 14653, 14654, 14
655, 14658, 14662, 14670, 14671, 14672, 14683, 14686, 14688, 14689, 14690, 14691, 14693, 1469
5, 14696, 14702, 14703, 14704, 14717, 14718, 14722, 14725, 14726, 14727, 14729, 14730, 14740,
14746, 14749, 14750, 14757, 14762, 14763, 15027, 15031, 15035, 15044, 15047, 15062, 15068, 15
071, 15099, 15108, 15109, 15115, 15136, 15138, 15141, 15142, 15145, 15150, 15171, 15177, 1518
1, 15195, 15197, 15210, 15227, 15239, 15268, 16004, 16005, 16017, 16021, 16023, 16033, 16043,
16046, 16051, 16082, 16092, 16094, 16106, 16110, 16115, 16126, 16129, 16148, 16149, 16153, 16
156, 16172, 16175, 16179, 16192, 16201, 16202, 16204, 16214, 16224, 16228, 16230, 16247, 1625
7, 16259, 16262, 16274, 16278, 16281, 16286, 16294, 16313, 16314, 16322, 16327, 16328, 16332,
16339, 16344, 16371, 16376, 16386, 16391, 16406, 16410, 16411, 16417, 17001, 17040, 17090, 17
103, 17123, 17144, 17169, 17189, 17219, 17272, 17277, 17340, 17344, 17352, 17456, 17457, 1802
3, 18123, 18239, 19010, 19019, 19032, 19098, 19101, 19123, 19127, 19183, 19185, 19197, 19218,
19252, 19267, 19282, 21073, 21178, 21183, 21213, 21272, 21318, 21327, 21352, 21443, 21452, 21
453, 21486, 21507, 21513, 21621, 21623, 21697, 22007, 22038, 22046, 22051, 22055, 22058, 2206
6, 22078, 22080, 22084, 22093, 22100, 22102, 22107, 22123, 22151, 22154, 22158, 22167, 22173,
22191, 22192, 22203, 22206, 22209, 22219, 22247, 22251, 22253, 22264, 22290, 22292, 22297, 22
298, 22303, 22357, 22367, 23094, 23109, 23121, 23126, 23149, 23153, 23161, 23163, 23189, 2319
2, 23231, 24013, 24026, 24028, 24033, 24035, 24044, 24047, 24053, 24064, 24065, 24072, 24079,
24092, 24093, 24099, 24103, 24112, 24117, 24118, 24127, 24147, 24166, 24170, 24172, 24178, 24
198, 24201, 24203, 24204, 24216, 24219, 24233, 24235, 24239, 24249, 24253, 24258, 24259, 2427
0, 24283, 24310, 24312, 24315, 24316, 24333, 24343, 24344, 24362, 24363, 24364, 24368, 24369,
24376, 24387, 24389, 24391, 24402, 24423, 24427, 24431, 24433, 24435, 24439, 24447, 24467, 24
469, 24475, 24490, 24497, 24503, 24530, 24540, 24554, 24561, 24579, 25010, 25028, 25034, 2503
5, 25037, 25064, 25076, 25128, 25140, 25144, 25146, 25165, 25167, 25168, 25205, 25272, 25291,
25302, 25337, 25353, 25358, 25363, 25396, 25407, 25409, 25424, 25434, 25438, 25480, 25484, 25
```

```
509, 25543, 25568, 25581, 25585, 25587, 25606, 26001, 26086, 26184, 26187, 26210, 26297, 2635
4, 26366, 27007, 27011, 27022, 27032, 27041, 27049, 27058, 27060, 27062, 27070, 27084, 27085,
27088, 27089, 27090, 27092, 27093, 27105, 27107, 27112, 27121, 27122, 27128, 27131, 27143, 27
145, 27150, 27157, 27159, 27160, 27172, 27175, 27191, 27195, 27197, 27198, 27211, 27213, 2722
1, 27223, 27225, 27244, 27250, 27253, 27255, 27257, 27262, 27263, 27264, 27265, 27268, 27270,
27274, 27277, 27283, 27290, 27292, 27294, 27296, 27297, 27302, 27303, 27305, 27308, 27344, 27
356, 27362, 27402, 27412, 27416, 27425, 27447, 27448, 27449, 27452, 27467, 27471, 27479, 2748
4, 27491, 27499, 27506, 27510, 27513, 27515, 27516, 27519, 27523, 27526, 27532, 27541, 27549,
27551, 27554, 27565, 27566, 27573, 27574, 27578, 27581, 27588, 27596, 27600, 27607, 27626, 27
628, 27634, 27636, 27637, 27638, 27639, 27642, 27647, 27648, 27651, 27653, 27657, 27676, 2767
9, 27685, 27687, 28002, 28011, 28012, 28015, 28017, 28018, 28020, 28043, 28044, 28063, 28083,
28093, 28097, 28101, 28103, 28112, 28115, 28125, 28133, 28138, 28145, 28150, 28165, 28174, 28
179, 28181, 28199, 28204, 28205, 28224, 28228, 28236, 28238, 28241, 28258, 28262, 28288, 2829
5, 28307, 28311, 28318, 28320, 28328, 28330, 28331, 28334, 28338, 28340, 28356, 28361, 28376,
28381, 28384, 28399, 28406, 28412, 28413, 28416, 28420, 28422, 29003, 29021, 29052, 29076, 29
092, 29127, 29129, 29149, 29199, 29203, 29219, 29227, 29266, 30052, 30157, 31016, 31154, 3129
8, 31307, 31412, 32011, 32074, 32168, 32179, 32344, 33018, 33055, 33106, 33107, 33267, 33371,
33380, 33495, 34246, 34330, 35004, 35011, 35036, 35048, 35053, 35060, 35069, 35074, 35083, 35
100, 35113, 35129, 35147, 35158, 35163, 35168, 35176, 35184, 35191, 35209, 35220, 35254, 3525
7, 35267, 35269, 35282, 35292, 35293, 35301, 35303, 35308, 35323, 35341, 35344, 35348, 36039,
36072, 36093, 36151, 36183, 36201, 36206, 36229, 36244, 36245, 37021, 37102, 37120, 37135, 37
209, 37227, 37232, 38001, 38014, 38016, 38021, 38022, 38025, 38028, 38073, 38121, 38125, 3814
5, 38163, 38165, 38225, 38227, 38253, 38262, 38274, 38284, 38292, 38293, 38297, 38302, 38305,
38306, 38367, 38395, 38435, 38456, 38479, 38534, 38541, 38560, 39005, 39012, 39018, 39023, 39
036, 39043, 39044, 39064, 39069, 39075, 39113, 39115, 39135, 39158, 39161, 39177, 39186, 3919
0, 39195, 39209, 39213, 39215, 39226, 39243, 39256, 39258, 39260, 39264, 39273, 39286, 39294,
39303, 39309, 39329, 39332, 39339, 39340, 39341, 39347, 39358, 39368, 39369, 39371, 39378, 39
382, 39383, 39395, 39414, 39417, 39438, 39440, 39442, 39482, 39484, 39485, 39488, 39491, 3950
9, 39510, 39530, 39537, 39542, 39544, 39549, 39562, 39564, 39566, 39576, 39578, 40009, 40048,
40107, 40243, 40302, 41005, 41011, 41015, 41021, 41023, 41033, 41055, 41056, 41059, 41064, 41
070, 41076, 41082, 41092, 41133, 41142, 41151, 41165, 41167, 41169, 41170, 41171, 41173, 4118
3, 41197, 41202, 41210, 41227, 41240, 41244, 41248, 41257, 41263, 41264, 41270, 41272, 42004,
42039, 42080, 42082, 42114, 42190, 42245, 42250, 42268, 42291, 43176, 43255, 44004, 44005, 44
008, 44011, 44017, 44021, 44029, 44034, 44040, 44059, 44087, 44093, 44147, 44163, 44180, 4418
1, 44191, 44213, 44219, 44225, 45003, 45007, 45048, 45051, 45057, 45090, 45106, 45129, 45140,
45163, 45172, 45190, 45191, 45192, 45211, 45221, 45236, 45267, 45287, 45295, 45304, 45318, 45
319, 45349, 46014, 46019, 46025, 46033, 46048, 46063, 46067, 46071, 46077, 46083, 46099, 4610
3, 46110, 46138, 46141, 46150, 46156, 46158, 46166, 46201, 46232, 46248, 46252, 46261, 46262,
46263, 46268, 46274, 46275, 46278, 46285, 46287, 46291, 46298, 46300, 46311, 46325, 46326, 46
327, 46331, 47116, 47149, 47166, 47268, 47322, 48009, 48011, 48014, 48022, 48023, 48027, 4803
5, 48038, 48040, 48047, 48049, 48050, 48057, 48060, 48061, 48062, 48066, 48076, 48078, 48084,
48087, 48093, 48094, 48099, 48101, 48102, 48105, 48116, 48120, 48122, 48125, 48126, 48127, 48
133, 48134, 48139, 48142, 48146, 48152, 48154, 48162, 48164, 48166, 48172, 48180, 48183, 4818
4, 48186, 48189, 48195, 48196, 48197, 49001, 49003, 49004, 49005, 49006, 49013, 49014, 49016,
49018, 49019, 49021, 49023, 49024, 49025, 49029, 49031, 49032, 49033, 49034, 49037, 49039, 49
040, 49042, 49043, 49044, 49046, 49047, 49049, 49050, 49051, 49052, 49059, 49060, 49062, 4906
5, 49066, 49067, 49069, 49071, 49072, 49073, 49074, 49075, 49077, 49078, 49079, 49080, 49081,
49083, 49084, 49085, 49086, 49087, 49088, 49091, 49092, 49093, 49094, 49095, 49096, 49097, 49
098, 49101, 49103, 49104, 49105, 49106, 49108, 49111, 49115, 49116, 49117, 49118, 49119, 4912
2, 49125, 49126, 49128, 49133, 49134, 49136, 49137, 49138, 49139, 49141, 49142, 49143, 49145,
49147, 49148, 49149, 49150, 49151, 49153, 49154, 49156, 49157, 49158, 49159, 49162, 49163, 49
165, 49167, 49168, 49169, 49172, 49173, 49174, 49175, 49177, 49179, 49181, 49183, 49184, 4918
5, 49186, 49187, 49189, 49190, 49191, 49194, 49196, 49197, 49198, 49199, 49200, 49202, 49204,
49206, 49207, 49208, 49212, 49213, 49218, 49225, 49226, 49227, 49228, 49229, 49230, 49232, 49
233, 49234, 49238, 49239, 49242, 49243, 49244, 49245, 49248, 49249, 49250, 49251, 49252, 4925
4, 49256, 49258, 49261, 49263, 49264, 49265, 49268, 49270, 49273, 49274, 49276, 49277, 49279,
```

49280, 49281, 49282, 49285, 49286, 49287, 49289, 49290, 49292, 49293, 49295, 49296, 49297, 49
298, 49300, 49301, 49303, 49304, 49305, 49307, 49309, 49312, 49313, 49314, 49315, 49316, 4931
7, 49318, 49319, 49320, 49322, 49323, 49324, 49325, 49327, 49331, 49335, 49337, 49342, 49345,
49346, 49348, 49349, 49350, 49351, 49354, 49356, 49360, 49363, 49365, 49366, 49367, 49372, 49
373, 49375, 49376, 49377, 49379, 49380, 49382, 50001, 50005, 50007, 50009, 50010, 50012, 5001
4, 50018, 50020, 50035, 50037, 50041, 50043, 50047, 50051, 50053, 50056, 50057, 50061, 50063,
50067, 50073, 50075, 50080, 50082, 50089, 50090, 50095, 50099, 50100, 50103, 50107, 50113, 50
116, 50119, 50123, 50125, 50127, 50128, 50129, 50131, 50132, 50133, 50134, 50136, 50139, 5014
0, 50142, 50153, 50154, 50160, 50163, 50168, 50170, 50171, 50173, 50179, 50180, 50187, 50189,
50191, 50197, 50201, 50202, 50203, 50204, 50209, 50211, 50212, 50213, 50215, 50220, 50223, 50
224, 50236, 50239, 50242, 50244, 50245, 50248, 50249, 50250, 50254, 50255, 50257, 50260, 5026
7, 50273, 50280, 50284, 50286, 50287, 50292, 50293, 50301, 50306, 50308, 50309, 50313, 50316,
50318, 50319, 50320, 50322, 50323, 50325, 50329, 50330, 50333, 50337, 50339, 50343, 50344, 50
346, 50348, 50354, 50355, 50358, 50359, 50363, 50367, 50375, 50377, 50380, 50381, 50385, 5038
6, 50388, 50391, 50393, 50396, 50400, 50404, 50409, 50410, 50412, 50414, 50415, 50416, 50417,
50418, 50419, 50424, 50427, 50431, 50432, 50434, 50436, 50438, 50439, 50440, 50441, 50444, 50
449, 50458, 50460, 50465, 50470, 50477, 50484, 50485, 50487, 50492, 50494, 50497, 50501, 5050
3, 50508, 50515, 50516, 50520, 50523, 50524, 50526, 50534, 50544, 50545, 50546, 50550, 50557,
50558, 50561, 50564, 50565, 50573, 50581, 50582, 50583, 50586, 50591, 50592, 50595, 50600, 50
601, 50602, 50605, 50608, 50611, 50614, 50620, 50622, 50623, 50625, 50627, 50630, 50631, 5063
2, 50635, 50636, 50638, 50639, 50640, 50642, 50644, 50646, 50649, 51021, 51024, 51030, 51064,
51075, 51171, 51261, 51271, 51331, 51347, 51383, 51405, 51411, 51494, 51564, 51575, 51612, 51
651, 51664, 52010, 52018, 52020, 52032, 52036, 52041, 52049, 52052, 52054, 52064, 52068, 5207
1, 52073, 52077, 52078, 52080, 52081, 52100, 52111, 52112, 52117, 52139, 52140, 52143, 52144,
52150, 52153, 52154, 52166, 52176, 52180, 52186, 52187, 52191, 52192, 52202, 52209, 52210, 52
215, 52224, 52225, 52226, 52236, 52238, 52239, 52252, 52259, 52261, 52262, 52263, 52268, 5227
0, 52279, 52281, 52283, 52293, 52296, 52299, 52309, 52314, 52317, 52324, 52329, 52331, 52333,
52339, 52340, 52345, 52351, 52361, 52368, 52379, 52381, 52382, 52389, 52404, 52405, 52408, 52
409, 52410, 52411, 52412, 52417, 52418, 52427, 52434, 52435, 52441, 52449, 52451, 52460, 5246
2, 52466, 52477, 52478, 52481, 52496, 52498, 52501, 52508, 52509, 52521, 52527, 52529, 52530,
52531, 52532, 52533, 52537, 53004, 53006, 53014, 53017, 53029, 53032, 53044, 53050, 53060, 53
062, 53065, 53092, 53095, 53097, 53104, 53124, 53136, 53137, 53138, 53159, 53161, 53185, 5319
4, 53205, 53207, 53215, 53228, 53231, 53241, 53244, 53252, 53254, 53255, 54099, 54341, 54342,
54506, 54557, 55003, 55006, 55016, 55019, 55020, 55030, 55052, 55056, 55074, 55086, 55090, 55
092, 55112, 55126, 55131, 55136, 55151, 55152, 55161, 55164, 55176, 55190, 55203, 55205, 5521
3, 55223, 55230, 55231, 55233, 55234, 55235, 55238, 55240, 55249, 55259, 55273, 55283, 55287,
55294, 55305, 55308, 55309, 55314, 55318, 55319, 55342, 55390, 55392, 55393, 55402, 55413, 55
417, 55418, 55432, 55440, 55441, 55451, 55455, 55478, 55480, 55483, 55486, 55491, 55499, 5551
3, 55529, 55537, 55538, 55539, 55542, 55548, 55550, 55558, 55585, 56016, 56037, 56038, 56059,
56061, 56064, 56102, 56138, 56142, 56144, 56150, 56183, 56187, 56192, 56197, 57021, 57023, 57
135, 57148, 57157, 57184, 57279, 57285, 57378, 57420, 57432, 57439, 57482, 57523, 57578, 5758
5, 57608, 57722, 58022, 58026, 58091, 58100, 58167, 58204, 59154, 59248, 59260, 59298, 59355,
59380, 59404, 59540, 59561, 59588, 60018, 60029, 60038, 60054, 60080, 60096, 60196, 60209, 60
245, 60246, 60256, 60300, 60417, 60453, 60455, 60475, 60532, 60570, 60644, 60649, 60682, 6069
0, 60694, 61003, 61004, 61007, 61009, 61016, 61019, 61025, 61027, 61031, 61033, 61045, 61047,
61057, 61058, 61059, 61065, 61073, 61081, 61083, 61090, 61096, 61106, 61109, 61110, 61112, 61
115, 61116, 61125, 61127, 61131, 61135, 61136, 61144, 61145, 61153, 61154, 61155, 61157, 6116
1, 61167, 61172, 61173, 61174, 61175, 61177, 61179, 61184, 61185, 61186, 61191, 61194, 61196,
61200, 61201, 61204, 61205, 61211, 61220, 61223, 61226, 61228, 61230, 61231, 61235, 61236, 61
239, 61246, 61247, 61249, 61250, 61253, 61270, 61280, 61282, 61285, 61292, 61294, 61296, 6129
9, 61305, 61306, 61309, 61311, 61313, 61315, 61318, 61320, 61324, 61325, 61335, 61337, 61338,
61339, 61340, 61341, 61343, 61353, 61354, 61355, 61356, 61359, 61364, 61368, 61375, 61377, 61
378, 61380, 61383, 61403, 61409, 61410, 61417, 61428, 61429, 61431, 61434, 61437, 61441, 6144
9, 61455, 61458, 61460, 61463, 61465, 61468, 61469, 61470, 61471, 61474, 61477, 61478, 61483,
61484, 61486, 61489, 61491, 61496, 61504, 61506, 61509, 61511, 61513, 62110, 62226, 62294, 62
295, 62431, 62471, 62482, 62575, 62691, 62757, 62807, 63018, 63068, 63078, 63133, 63160, 6322

```
6, 63244, 63255, 63266, 63316, 63448, 64020, 64030, 64055, 64076, 64163, 64164, 64169, 64225,
64248, 64278, 64333, 64384, 64402, 64497, 64553, 65008, 65027, 65092, 65122, 65188, 65192, 65
227, 65246, 65282, 65365, 67004, 67007, 67014, 67015, 67041, 67042, 67064, 67114, 67153, 6715
7, 67158, 67170, 67171, 67193, 67207, 67211, 67219, 67224, 67243, 67251, 67262, 67284, 67294,
67297, 67316, 67332, 67372, 67374, 67376, 67390, 67393, 67399, 67402, 67418, 67419, 67431, 67
439, 67457, 67469, 67495, 67496, 67512, 67533, 67539, 67549, 67560, 67561, 67562, 68006, 6801
2, 68031, 68056, 68070, 68108, 68133, 68143, 68162, 68164, 68201, 68206, 68219, 68233, 68240,
68272, 68310, 68314, 68319, 68320, 69015, 69019, 69024, 69025, 69041, 69048, 69066, 69073, 69
101, 69114, 69128, 69129, 69135, 69144, 69146, 69147, 69150, 69157, 69158, 69159, 69179, 6918
5, 69195, 69208, 69210, 69211, 69213, 69221, 69222, 69223, 69224, 69228, 69237, 69247, 69248,
69251, 69255, 70033, 70034, 70068, 70108, 70123, 70131, 70139, 70173, 70191, 70209, 70223, 70
241, 70245, 70246, 70281, 70300, 70333, 70345, 70346, 70354, 70370, 70382, 70391, 70418, 7043
4, 70458, 70465, 70475, 70489, 70497, 70551, 70570, 71055, 71134, 71180, 71260, 71279, 71288,
71304, 71375, 71432, 71511, 71582, 71587, 72023, 72025, 72063, 72069, 72071, 72080, 72081, 72
082, 72097, 72108, 72116, 72117, 72128, 72137, 72138, 72159, 72162, 72176, 72189, 72203, 7220
7, 72240, 72258, 72262, 72284, 72288, 72301, 72304, 72308, 72318, 72363, 72384, 73002, 73003,
73006, 73010, 73035, 73038, 73045, 73046, 73056, 73060, 73062, 73080, 73093, 73102, 73108, 73
111, 73112, 73115, 73118, 73126, 73127, 73135, 73143, 73144, 73150, 73151, 73158, 73163, 7316
7, 73169, 73174, 73185, 73187, 73198, 73199, 73203, 73209, 73212, 73215, 73227, 73235, 73236,
73238, 73239, 73244, 73257, 73260, 73263, 73264, 73266, 73284, 73287, 73290, 73295, 73305, 73
321, 74010, 74011, 74022, 74084, 74093, 74113, 74115, 74120, 74123, 74167, 74182, 74187, 7420
4, 74217, 74245, 74268, 74270, 74275, 74282, 76003, 76027, 76031, 76034, 76037, 76041, 76044,
76073, 76078, 76080, 76081, 76089, 76098, 76127, 76137, 76145, 76146, 76164, 76191, 76215, 76
248, 76258, 76267, 76276, 76277, 76289, 76301, 76310, 76326, 76337, 76376, 76401, 76444, 7646
6, 76476, 76496, 76525, 76539, 76607, 76618, 76625, 76639, 76643, 76659, 76674, 76696, 76701,
76704, 76713, 76729, 76742, 77017, 77028, 77166, 77170, 77189, 77213, 77299, 77316, 77324, 77
334, 77346, 77351, 77375, 77399, 77433, 77491, 77497, 77499, 77502, 77504, 78158, 78251, 7832
0, 78503, 78524, 78551, 79004, 79005, 79006, 79013, 79014, 79021, 79026, 79027, 79028, 79030,
79033, 79035, 79039, 79041, 79043, 79044, 79051, 79052, 79053, 79054, 79061, 79063, 79064, 79
065, 79068, 79072, 79073, 79075, 79077, 79083, 79093, 79098, 79099, 79105, 79107, 79113, 7914
3, 79155, 79168, 79171, 79173, 79174, 79178, 79179, 79181, 79182, 79185, 79186, 79187, 79188,
79192, 79193, 79196, 79199, 79217, 79219, 79222, 79224, 79232, 79233, 79237, 79240, 79247, 79
260, 79261, 79264, 79272, 79275, 79279, 79280, 79282, 79285, 79288, 79292, 79296, 79314, 7931
7, 79318, 79321, 79323, 79324, 79325, 79327, 79328, 79330, 79333, 79334, 79344, 79353, 80007,
80111, 80175, 80209, 80257, 80289, 80295, 80363, 80382, 80394, 80398, 80419, 80441, 80447, 80
454, 80457, 80483, 80485, 80492, 80505, 80509, 80510, 80532, 80551, 80564, 80581, 80594, 8060
8, 80610, 80612, 80621, 80625, 80689, 80712, 80731, 80739, 80745, 80761, 80768, 80816, 80817,
81057, 81062, 81091, 81113, 81153, 81226, 81233, 81241, 81260, 81296, 81301, 85008, 85009, 85
019, 85027, 85030, 85043, 85044, 85048, 85052, 85063, 85068, 85069, 85079, 85080, 85084, 8509
0, 85091, 85107, 85122, 85146, 85150, 85152, 85154, 85162, 85165, 85168, 85177, 85180, 85197,
85203, 85212, 85213, 85217, 85219, 85224, 85230, 85257, 85263, 85270, 85272, 85275, 85279, 85
299, 85302, 86019, 86021, 86030, 86043, 86053, 86056, 86060, 86067, 86071, 86082, 86101, 8611
5, 86123, 86146, 86155, 86166, 86179, 86185, 86188, 86199, 86208, 86212, 86216, 86219, 86240,
86245, 86259, 86267, 86277, 86278, 86281, 86283, 87026, 87028, 87055, 87097, 87098, 87128, 87
136, 87171, 87173, 87175, 87184, 87196, 88018, 88029, 88067, 88112, 88204, 88218, 88234, 8823
5, 88337, 88361, 88420, 88465, 89001, 89003, 89047, 89052, 89070, 89078, 89086, 89090, 89097,
89103, 89107, 89109, 89114, 89130, 89135, 89138, 89140, 89157, 89166, 89174, 89178, 89192, 89
196, 89197, 89213, 89231, 89241, 89243, 89260, 89275, 89293, 89294, 89317, 89322, 89330, 8933
4, 89340, 89343, 89356, 89357, 89358, 89366, 89372, 89381, 89388, 89389, 89401, 89405, 89411,
89420, 89421, 89429, 89435, 89448, 89454, 89457, 89473, 89476, 89484, 90068, 90073, 91182, 91
222, 91228, 91390
```

```
ID$insee2020_dep <- plyr::mapvalues(ID$annee_2020.Iden, from = lookup_com$inseecom_2020.com,
to = lookup_com$inseecom_2020.dep)
```

```
## The following `from` values were not present in `x`: 01015, 01025, 01033, 01036, 01059, 01
080, 01091, 01095, 01097, 01098, 01119, 01120, 01122, 01130, 01137, 01144, 01154, 01172, 0117
6, 01182, 01185, 01186, 01187, 01204, 01205, 01215, 01218, 01221, 01227, 01271, 01286, 01292,
01300, 01312, 01316, 01324, 01338, 01340, 01341, 01409, 01413, 01414, 01417, 01426, 01442, 01
453, 02018, 02026, 02053, 02147, 02153, 02161, 02285, 02300, 02301, 02325, 02348, 02360, 0243
4, 02439, 02458, 02475, 02479, 02597, 02630, 02646, 02669, 02733, 02771, 02811, 02825, 03123,
03153, 03158, 03168, 03318, 04003, 04010, 04011, 04014, 04029, 04033, 04052, 04082, 04100, 04
105, 04120, 04131, 04153, 04165, 04198, 05001, 05002, 05005, 05020, 05024, 05034, 05042, 0505
3, 05067, 05069, 05088, 05101, 05118, 05120, 05125, 05132, 05138, 05139, 05143, 05150, 05175,
07011, 07016, 07043, 07046, 07057, 07135, 07164, 07165, 07246, 07256, 07262, 07271, 08007, 08
009, 08012, 08042, 08050, 08051, 08053, 08072, 08079, 08112, 08114, 08115, 08116, 08145, 0815
2, 08173, 08177, 08198, 08213, 08258, 08261, 08265, 08266, 08267, 08285, 08311, 08337, 08345,
08359, 08371, 08441, 08443, 08475, 08490, 08491, 08493, 08495, 09028, 09036, 09062, 09135, 09
286, 09296, 09302, 09317, 09334, 10003, 10036, 10277, 10311, 10415, 11050, 11097, 11131, 1117
1, 11251, 11298, 11323, 11329, 11349, 12005, 12014, 12020, 12021, 12040, 12076, 12081, 12087,
12090, 12112, 12114, 12117, 12120, 12123, 12126, 12132, 12173, 12177, 12196, 12218, 12223, 12
224, 12245, 12270, 12271, 12279, 12285, 12304, 14002, 14004, 14005, 14008, 14011, 14013, 1401
7, 14027, 14028, 14029, 14031, 14035, 14037, 14051, 14052, 14056, 14058, 14061, 14065, 14073,
14074, 14075, 14081, 14098, 14099, 14105, 14109, 14112, 14113, 14114, 14115, 14128, 14129, 14
139, 14142, 14143, 14144, 14148, 14150, 14151, 14152, 14153, 14154, 14155, 14156, 14157, 1415
8, 14164, 14170, 14174, 14176, 14178, 14186, 14187, 14188, 14189, 14192, 14200, 14201, 14208,
14210, 14212, 14213, 14217, 14219, 14222, 14235, 14247, 14253, 14255, 14259, 14264, 14265, 14
268, 14279, 14281, 14292, 14294, 14296, 14313, 14317, 14321, 14324, 14330, 14331, 14339, 1434
0, 14342, 14347, 14349, 14350, 14355, 14356, 14357, 14359, 14361, 14371, 14372, 14373, 14376,
14382, 14386, 14387, 14388, 14395, 14406, 14410, 14413, 14414, 14415, 14416, 14417, 14418, 14
420, 14422, 14423, 14429, 14431, 14432, 14433, 14440, 14441, 14442, 14443, 14444, 14450, 1445
6, 14459, 14462, 14463, 14471, 14472, 14474, 14475, 14477, 14481, 14489, 14493, 14503, 14505,
14508, 14517, 14518, 14521, 14523, 14525, 14527, 14532, 14537, 14538, 14539, 14543, 14544, 14
545, 14548, 14549, 14551, 14553, 14554, 14560, 14564, 14568, 14570, 14573, 14576, 14577, 1457
9, 14580, 14581, 14583, 14584, 14585, 14591, 14596, 14597, 14599, 14600, 14608, 14611, 14615,
14616, 14618, 14628, 14629, 14632, 14633, 14634, 14636, 14638, 14642, 14647, 14653, 14654, 14
655, 14658, 14662, 14670, 14671, 14672, 14683, 14686, 14688, 14689, 14690, 14691, 14693, 1469
5, 14696, 14702, 14703, 14704, 14717, 14718, 14722, 14725, 14726, 14727, 14729, 14730, 14740,
14746, 14749, 14750, 14757, 14762, 14763, 15027, 15031, 15035, 15044, 15047, 15062, 15068, 15
071, 15099, 15108, 15109, 15115, 15136, 15138, 15141, 15142, 15145, 15150, 15171, 15177, 1518
1, 15195, 15197, 15210, 15227, 15239, 15268, 16004, 16005, 16017, 16021, 16023, 16033, 16043,
16046, 16051, 16082, 16092, 16094, 16106, 16110, 16115, 16126, 16129, 16148, 16149, 16153, 16
156, 16172, 16175, 16179, 16192, 16201, 16202, 16204, 16214, 16224, 16228, 16230, 16247, 1625
7, 16259, 16262, 16274, 16278, 16281, 16286, 16294, 16313, 16314, 16322, 16327, 16328, 16332,
16339, 16344, 16371, 16376, 16386, 16391, 16406, 16410, 16411, 16417, 17001, 17040, 17090, 17
103, 17123, 17144, 17169, 17189, 17219, 17272, 17277, 17340, 17344, 17352, 17456, 17457, 1802
3, 18123, 18239, 19010, 19019, 19032, 19098, 19101, 19123, 19127, 19183, 19185, 19197, 19218,
19252, 19267, 19282, 21073, 21178, 21183, 21213, 21272, 21318, 21327, 21352, 21443, 21452, 21
453, 21486, 21507, 21513, 21621, 21623, 21697, 22007, 22038, 22046, 22051, 22055, 22058, 2206
6, 22078, 22080, 22084, 22093, 22100, 22102, 22107, 22123, 22151, 22154, 22158, 22167, 22173,
22191, 22192, 22203, 22206, 22209, 22219, 22247, 22251, 22253, 22264, 22290, 22292, 22297, 22
298, 22303, 22357, 22367, 23094, 23109, 23121, 23126, 23149, 23153, 23161, 23163, 23189, 2319
2, 23231, 24013, 24026, 24028, 24033, 24035, 24044, 24047, 24053, 24064, 24065, 24072, 24079,
24092, 24093, 24099, 24103, 24112, 24117, 24118, 24127, 24147, 24166, 24170, 24172, 24178, 24
198, 24201, 24203, 24204, 24216, 24219, 24233, 24235, 24239, 24249, 24253, 24258, 24259, 2427
0, 24283, 24310, 24312, 24315, 24316, 24333, 24343, 24344, 24362, 24363, 24364, 24368, 24369,
24376, 24387, 24389, 24391, 24402, 24423, 24427, 24431, 24433, 24435, 24439, 24447, 24467, 24
469, 24475, 24490, 24497, 24503, 24530, 24540, 24554, 24561, 24579, 25010, 25028, 25034, 2503
5, 25037, 25064, 25076, 25128, 25140, 25144, 25146, 25165, 25167, 25168, 25205, 25272, 25291,
25302, 25337, 25353, 25358, 25363, 25396, 25407, 25409, 25424, 25434, 25438, 25480, 25484, 25
```

509, 25543, 25568, 25581, 25585, 25587, 25606, 26001, 26086, 26184, 26187, 26210, 26297, 2635
4, 26366, 27007, 27011, 27022, 27032, 27041, 27049, 27058, 27060, 27062, 27070, 27084, 27085,
27088, 27089, 27090, 27092, 27093, 27105, 27107, 27112, 27121, 27122, 27128, 27131, 27143, 27
145, 27150, 27157, 27159, 27160, 27172, 27175, 27191, 27195, 27197, 27198, 27211, 27213, 2722
1, 27223, 27225, 27244, 27250, 27253, 27255, 27257, 27262, 27263, 27264, 27265, 27268, 27270,
27274, 27277, 27283, 27290, 27292, 27294, 27296, 27297, 27302, 27303, 27305, 27308, 27344, 27
356, 27362, 27402, 27412, 27416, 27425, 27447, 27448, 27449, 27452, 27467, 27471, 27479, 2748
4, 27491, 27499, 27506, 27510, 27513, 27515, 27516, 27519, 27523, 27526, 27532, 27541, 27549,
27551, 27554, 27565, 27566, 27573, 27574, 27578, 27581, 27588, 27596, 27600, 27607, 27626, 27
628, 27634, 27636, 27637, 27638, 27639, 27642, 27647, 27648, 27651, 27653, 27657, 27676, 2767
9, 27685, 27687, 28002, 28011, 28012, 28015, 28017, 28018, 28020, 28043, 28044, 28063, 28083,
28093, 28097, 28101, 28103, 28112, 28115, 28125, 28133, 28138, 28145, 28150, 28165, 28174, 28
179, 28181, 28199, 28204, 28205, 28224, 28228, 28236, 28238, 28241, 28258, 28262, 28288, 2829
5, 28307, 28311, 28318, 28320, 28328, 28330, 28331, 28334, 28338, 28340, 28356, 28361, 28376,
28381, 28384, 28399, 28406, 28412, 28413, 28416, 28420, 28422, 29003, 29021, 29052, 29076, 29
092, 29127, 29129, 29149, 29199, 29203, 29219, 29227, 29266, 30052, 30157, 31016, 31154, 3129
8, 31307, 31412, 32011, 32074, 32168, 32179, 32344, 33018, 33055, 33106, 33107, 33267, 33371,
33380, 33495, 34246, 34330, 35004, 35011, 35036, 35048, 35053, 35060, 35069, 35074, 35083, 35
100, 35113, 35129, 35147, 35158, 35163, 35168, 35176, 35184, 35191, 35209, 35220, 35254, 3525
7, 35267, 35269, 35282, 35292, 35293, 35301, 35303, 35308, 35323, 35341, 35344, 35348, 36039,
36072, 36093, 36151, 36183, 36201, 36206, 36229, 36244, 36245, 37021, 37102, 37120, 37135, 37
209, 37227, 37232, 38001, 38014, 38016, 38021, 38022, 38025, 38028, 38073, 38121, 38125, 3814
5, 38163, 38165, 38225, 38227, 38253, 38262, 38274, 38284, 38292, 38293, 38297, 38302, 38305,
38306, 38367, 38395, 38435, 38456, 38479, 38534, 38541, 38560, 39005, 39012, 39018, 39023, 39
036, 39043, 39044, 39064, 39069, 39075, 39113, 39115, 39135, 39158, 39161, 39177, 39186, 3919
0, 39195, 39209, 39213, 39215, 39226, 39243, 39256, 39258, 39260, 39264, 39273, 39286, 39294,
39303, 39309, 39329, 39332, 39339, 39340, 39341, 39347, 39358, 39368, 39369, 39371, 39378, 39
382, 39383, 39395, 39414, 39417, 39438, 39440, 39442, 39482, 39484, 39485, 39488, 39491, 3950
9, 39510, 39530, 39537, 39542, 39544, 39549, 39562, 39564, 39566, 39576, 39578, 40009, 40048,
40107, 40243, 40302, 41005, 41011, 41015, 41021, 41023, 41033, 41055, 41056, 41059, 41064, 41
070, 41076, 41082, 41092, 41133, 41142, 41151, 41165, 41167, 41169, 41170, 41171, 41173, 4118
3, 41197, 41202, 41210, 41227, 41240, 41244, 41248, 41257, 41263, 41264, 41270, 41272, 42004,
42039, 42080, 42082, 42114, 42190, 42245, 42250, 42268, 42291, 43176, 43255, 44004, 44005, 44
008, 44011, 44017, 44021, 44029, 44034, 44040, 44059, 44087, 44093, 44147, 44163, 44180, 4418
1, 44191, 44213, 44219, 44225, 45003, 45007, 45048, 45051, 45057, 45090, 45106, 45129, 45140,
45163, 45172, 45190, 45191, 45192, 45211, 45221, 45236, 45267, 45287, 45295, 45304, 45318, 45
319, 45349, 46014, 46019, 46025, 46033, 46048, 46063, 46067, 46071, 46077, 46083, 46099, 4610
3, 46110, 46138, 46141, 46150, 46156, 46158, 46166, 46201, 46232, 46248, 46252, 46261, 46262,
46263, 46268, 46274, 46275, 46278, 46285, 46287, 46291, 46298, 46300, 46311, 46325, 46326, 46
327, 46331, 47116, 47149, 47166, 47268, 47322, 48009, 48011, 48014, 48022, 48023, 48027, 4803
5, 48038, 48040, 48047, 48049, 48050, 48057, 48060, 48061, 48062, 48066, 48076, 48078, 48084,
48087, 48093, 48094, 48099, 48101, 48102, 48105, 48116, 48120, 48122, 48125, 48126, 48127, 48
133, 48134, 48139, 48142, 48146, 48152, 48154, 48162, 48164, 48166, 48172, 48180, 48183, 4818
4, 48186, 48189, 48195, 48196, 48197, 49001, 49003, 49004, 49005, 49006, 49013, 49014, 49016,
49018, 49019, 49021, 49023, 49024, 49025, 49029, 49031, 49032, 49033, 49034, 49037, 49039, 49
040, 49042, 49043, 49044, 49046, 49047, 49049, 49050, 49051, 49052, 49059, 49060, 49062, 4906
5, 49066, 49067, 49069, 49071, 49072, 49073, 49074, 49075, 49077, 49078, 49079, 49080, 49081,
49083, 49084, 49085, 49086, 49087, 49088, 49091, 49092, 49093, 49094, 49095, 49096, 49097, 49
098, 49101, 49103, 49104, 49105, 49106, 49108, 49111, 49115, 49116, 49117, 49118, 49119, 4912
2, 49125, 49126, 49128, 49133, 49134, 49136, 49137, 49138, 49139, 49141, 49142, 49143, 49145,
49147, 49148, 49149, 49150, 49151, 49153, 49154, 49156, 49157, 49158, 49159, 49162, 49163, 49
165, 49167, 49168, 49169, 49172, 49173, 49174, 49175, 49177, 49179, 49181, 49183, 49184, 4918
5, 49186, 49187, 49189, 49190, 49191, 49194, 49196, 49197, 49198, 49199, 49200, 49202, 49204,
49206, 49207, 49208, 49212, 49213, 49218, 49225, 49226, 49227, 49228, 49229, 49230, 49232, 49
233, 49234, 49238, 49239, 49242, 49243, 49244, 49245, 49248, 49249, 49250, 49251, 49252, 4925
4, 49256, 49258, 49261, 49263, 49264, 49265, 49268, 49270, 49273, 49274, 49276, 49277, 49279,

49280, 49281, 49282, 49285, 49286, 49287, 49289, 49290, 49292, 49293, 49295, 49296, 49297, 49
298, 49300, 49301, 49303, 49304, 49305, 49307, 49309, 49312, 49313, 49314, 49315, 49316, 4931
7, 49318, 49319, 49320, 49322, 49323, 49324, 49325, 49327, 49331, 49335, 49337, 49342, 49345,
49346, 49348, 49349, 49350, 49351, 49354, 49356, 49360, 49363, 49365, 49366, 49367, 49372, 49
373, 49375, 49376, 49377, 49379, 49380, 49382, 50001, 50005, 50007, 50009, 50010, 50012, 5001
4, 50018, 50020, 50035, 50037, 50041, 50043, 50047, 50051, 50053, 50056, 50057, 50061, 50063,
50067, 50073, 50075, 50080, 50082, 50089, 50090, 50095, 50099, 50100, 50103, 50107, 50113, 50
116, 50119, 50123, 50125, 50127, 50128, 50129, 50131, 50132, 50133, 50134, 50136, 50139, 5014
0, 50142, 50153, 50154, 50160, 50163, 50168, 50170, 50171, 50173, 50179, 50180, 50187, 50189,
50191, 50197, 50201, 50202, 50203, 50204, 50209, 50211, 50212, 50213, 50215, 50220, 50223, 50
224, 50236, 50239, 50242, 50244, 50245, 50248, 50249, 50250, 50254, 50255, 50257, 50260, 5026
7, 50273, 50280, 50284, 50286, 50287, 50292, 50293, 50301, 50306, 50308, 50309, 50313, 50316,
50318, 50319, 50320, 50322, 50323, 50325, 50329, 50330, 50333, 50337, 50339, 50343, 50344, 50
346, 50348, 50354, 50355, 50358, 50359, 50363, 50367, 50375, 50377, 50380, 50381, 50385, 5038
6, 50388, 50391, 50393, 50396, 50400, 50404, 50409, 50410, 50412, 50414, 50415, 50416, 50417,
50418, 50419, 50424, 50427, 50431, 50432, 50434, 50436, 50438, 50439, 50440, 50441, 50444, 50
449, 50458, 50460, 50465, 50470, 50477, 50484, 50485, 50487, 50492, 50494, 50497, 50501, 5050
3, 50508, 50515, 50516, 50520, 50523, 50524, 50526, 50534, 50544, 50545, 50546, 50550, 50557,
50558, 50561, 50564, 50565, 50573, 50581, 50582, 50583, 50586, 50591, 50592, 50595, 50600, 50
601, 50602, 50605, 50608, 50611, 50614, 50620, 50622, 50623, 50625, 50627, 50630, 50631, 5063
2, 50635, 50636, 50638, 50639, 50640, 50642, 50644, 50646, 50649, 51021, 51024, 51030, 51064,
51075, 51171, 51261, 51271, 51331, 51347, 51383, 51405, 51411, 51494, 51564, 51575, 51612, 51
651, 51664, 52010, 52018, 52020, 52032, 52036, 52041, 52049, 52052, 52054, 52064, 52068, 5207
1, 52073, 52077, 52078, 52080, 52081, 52100, 52111, 52112, 52117, 52139, 52140, 52143, 52144,
52150, 52153, 52154, 52166, 52176, 52180, 52186, 52187, 52191, 52192, 52202, 52209, 52210, 52
215, 52224, 52225, 52226, 52236, 52238, 52239, 52252, 52259, 52261, 52262, 52263, 52268, 5227
0, 52279, 52281, 52283, 52293, 52296, 52299, 52309, 52314, 52317, 52324, 52329, 52331, 52333,
52339, 52340, 52345, 52351, 52361, 52368, 52379, 52381, 52382, 52389, 52404, 52405, 52408, 52
409, 52410, 52411, 52412, 52417, 52418, 52427, 52434, 52435, 52441, 52449, 52451, 52460, 5246
2, 52466, 52477, 52478, 52481, 52496, 52498, 52501, 52508, 52509, 52521, 52527, 52529, 52530,
52531, 52532, 52533, 52537, 53004, 53006, 53014, 53017, 53029, 53032, 53044, 53050, 53060, 53
062, 53065, 53092, 53095, 53097, 53104, 53124, 53136, 53137, 53138, 53159, 53161, 53185, 5319
4, 53205, 53207, 53215, 53228, 53231, 53241, 53244, 53252, 53254, 53255, 54099, 54341, 54342,
54506, 54557, 55003, 55006, 55016, 55019, 55020, 55030, 55052, 55056, 55074, 55086, 55090, 55
092, 55112, 55126, 55131, 55136, 55151, 55152, 55161, 55164, 55176, 55190, 55203, 55205, 5521
3, 55223, 55230, 55231, 55233, 55234, 55235, 55238, 55240, 55249, 55259, 55273, 55283, 55287,
55294, 55305, 55308, 55309, 55314, 55318, 55319, 55342, 55390, 55392, 55393, 55402, 55413, 55
417, 55418, 55432, 55440, 55441, 55451, 55455, 55478, 55480, 55483, 55486, 55491, 55499, 5551
3, 55529, 55537, 55538, 55539, 55542, 55548, 55550, 55558, 55585, 56016, 56037, 56038, 56059,
56061, 56064, 56102, 56138, 56142, 56144, 56150, 56183, 56187, 56192, 56197, 57021, 57023, 57
135, 57148, 57157, 57184, 57279, 57285, 57378, 57420, 57432, 57439, 57482, 57523, 57578, 5758
5, 57608, 57722, 58022, 58026, 58091, 58100, 58167, 58204, 59154, 59248, 59260, 59298, 59355,
59380, 59404, 59540, 59561, 59588, 60018, 60029, 60038, 60054, 60080, 60096, 60196, 60209, 60
245, 60246, 60256, 60300, 60417, 60453, 60455, 60475, 60532, 60570, 60644, 60649, 60682, 6069
0, 60694, 61003, 61004, 61007, 61009, 61016, 61019, 61025, 61027, 61031, 61033, 61045, 61047,
61057, 61058, 61059, 61065, 61073, 61081, 61083, 61090, 61096, 61106, 61109, 61110, 61112, 61
115, 61116, 61125, 61127, 61131, 61135, 61136, 61144, 61145, 61153, 61154, 61155, 61157, 6116
1, 61167, 61172, 61173, 61174, 61175, 61177, 61179, 61184, 61185, 61186, 61191, 61194, 61196,
61200, 61201, 61204, 61205, 61211, 61220, 61223, 61226, 61228, 61230, 61231, 61235, 61236, 61
239, 61246, 61247, 61249, 61250, 61253, 61270, 61280, 61282, 61285, 61292, 61294, 61296, 6129
9, 61305, 61306, 61309, 61311, 61313, 61315, 61318, 61320, 61324, 61325, 61335, 61337, 61338,
61339, 61340, 61341, 61343, 61353, 61354, 61355, 61356, 61359, 61364, 61368, 61375, 61377, 61
378, 61380, 61383, 61403, 61409, 61410, 61417, 61428, 61429, 61431, 61434, 61437, 61441, 6144
9, 61455, 61458, 61460, 61463, 61465, 61468, 61469, 61470, 61471, 61474, 61477, 61478, 61483,
61484, 61486, 61489, 61491, 61496, 61504, 61506, 61509, 61511, 61513, 62110, 62226, 62294, 62
295, 62431, 62471, 62482, 62575, 62691, 62757, 62807, 63018, 63068, 63078, 63133, 63160, 6322

```
6, 63244, 63255, 63266, 63316, 63448, 64020, 64030, 64055, 64076, 64163, 64164, 64169, 64225,
64248, 64278, 64333, 64384, 64402, 64497, 64553, 65008, 65027, 65092, 65122, 65188, 65192, 65
227, 65246, 65282, 65365, 67004, 67007, 67014, 67015, 67041, 67042, 67064, 67114, 67153, 6715
7, 67158, 67170, 67171, 67193, 67207, 67211, 67219, 67224, 67243, 67251, 67262, 67284, 67294,
67297, 67316, 67332, 67372, 67374, 67376, 67390, 67393, 67399, 67402, 67418, 67419, 67431, 67
439, 67457, 67469, 67495, 67496, 67512, 67533, 67539, 67549, 67560, 67561, 67562, 68006, 6801
2, 68031, 68056, 68070, 68108, 68133, 68143, 68162, 68164, 68201, 68206, 68219, 68233, 68240,
68272, 68310, 68314, 68319, 68320, 69015, 69019, 69024, 69025, 69041, 69048, 69066, 69073, 69
101, 69114, 69128, 69129, 69135, 69144, 69146, 69147, 69150, 69157, 69158, 69159, 69179, 6918
5, 69195, 69208, 69210, 69211, 69213, 69221, 69222, 69223, 69224, 69228, 69237, 69247, 69248,
69251, 69255, 70033, 70034, 70068, 70108, 70123, 70131, 70139, 70173, 70191, 70209, 70223, 70
241, 70245, 70246, 70281, 70300, 70333, 70345, 70346, 70354, 70370, 70382, 70391, 70418, 7043
4, 70458, 70465, 70475, 70489, 70497, 70551, 70570, 71055, 71134, 71180, 71260, 71279, 71288,
71304, 71375, 71432, 71511, 71582, 71587, 72023, 72025, 72063, 72069, 72071, 72080, 72081, 72
082, 72097, 72108, 72116, 72117, 72128, 72137, 72138, 72159, 72162, 72176, 72189, 72203, 7220
7, 72240, 72258, 72262, 72284, 72288, 72301, 72304, 72308, 72318, 72363, 72384, 73002, 73003,
73006, 73010, 73035, 73038, 73045, 73046, 73056, 73060, 73062, 73080, 73093, 73102, 73108, 73
111, 73112, 73115, 73118, 73126, 73127, 73135, 73143, 73144, 73150, 73151, 73158, 73163, 7316
7, 73169, 73174, 73185, 73187, 73198, 73199, 73203, 73209, 73212, 73215, 73227, 73235, 73236,
73238, 73239, 73244, 73257, 73260, 73263, 73264, 73266, 73284, 73287, 73290, 73295, 73305, 73
321, 74010, 74011, 74022, 74084, 74093, 74113, 74115, 74120, 74123, 74167, 74182, 74187, 7420
4, 74217, 74245, 74268, 74270, 74275, 74282, 76003, 76027, 76031, 76034, 76037, 76041, 76044,
76073, 76078, 76080, 76081, 76089, 76098, 76127, 76137, 76145, 76146, 76164, 76191, 76215, 76
248, 76258, 76267, 76276, 76277, 76289, 76301, 76310, 76326, 76337, 76376, 76401, 76444, 7646
6, 76476, 76496, 76525, 76539, 76607, 76618, 76625, 76639, 76643, 76659, 76674, 76696, 76701,
76704, 76713, 76729, 76742, 77017, 77028, 77166, 77170, 77189, 77213, 77299, 77316, 77324, 77
334, 77346, 77351, 77375, 77399, 77433, 77491, 77497, 77499, 77502, 77504, 78158, 78251, 7832
0, 78503, 78524, 78551, 79004, 79005, 79006, 79013, 79014, 79021, 79026, 79027, 79028, 79030,
79033, 79035, 79039, 79041, 79043, 79044, 79051, 79052, 79053, 79054, 79061, 79063, 79064, 79
065, 79068, 79072, 79073, 79075, 79077, 79083, 79093, 79098, 79099, 79105, 79107, 79113, 7914
3, 79155, 79168, 79171, 79173, 79174, 79178, 79179, 79181, 79182, 79185, 79186, 79187, 79188,
79192, 79193, 79196, 79199, 79217, 79219, 79222, 79224, 79232, 79233, 79237, 79240, 79247, 79
260, 79261, 79264, 79272, 79275, 79279, 79280, 79282, 79285, 79288, 79292, 79296, 79314, 7931
7, 79318, 79321, 79323, 79324, 79325, 79327, 79328, 79330, 79333, 79334, 79344, 79353, 80007,
80111, 80175, 80209, 80257, 80289, 80295, 80363, 80382, 80394, 80398, 80419, 80441, 80447, 80
454, 80457, 80483, 80485, 80492, 80505, 80509, 80510, 80532, 80551, 80564, 80581, 80594, 8060
8, 80610, 80612, 80621, 80625, 80689, 80712, 80731, 80739, 80745, 80761, 80768, 80816, 80817,
81057, 81062, 81091, 81113, 81153, 81226, 81233, 81241, 81260, 81296, 81301, 85008, 85009, 85
019, 85027, 85030, 85043, 85044, 85048, 85052, 85063, 85068, 85069, 85079, 85080, 85084, 8509
0, 85091, 85107, 85122, 85146, 85150, 85152, 85154, 85162, 85165, 85168, 85177, 85180, 85197,
85203, 85212, 85213, 85217, 85219, 85224, 85230, 85257, 85263, 85270, 85272, 85275, 85279, 85
299, 85302, 86019, 86021, 86030, 86043, 86053, 86056, 86060, 86067, 86071, 86082, 86101, 8611
5, 86123, 86146, 86155, 86166, 86179, 86185, 86188, 86199, 86208, 86212, 86216, 86219, 86240,
86245, 86259, 86267, 86277, 86278, 86281, 86283, 87026, 87028, 87055, 87097, 87098, 87128, 87
136, 87171, 87173, 87175, 87184, 87196, 88018, 88029, 88067, 88112, 88204, 88218, 88234, 8823
5, 88337, 88361, 88420, 88465, 89001, 89003, 89047, 89052, 89070, 89078, 89086, 89090, 89097,
89103, 89107, 89109, 89114, 89130, 89135, 89138, 89140, 89157, 89166, 89174, 89178, 89192, 89
196, 89197, 89213, 89231, 89241, 89243, 89260, 89275, 89293, 89294, 89317, 89322, 89330, 8933
4, 89340, 89343, 89356, 89357, 89358, 89366, 89372, 89381, 89388, 89389, 89401, 89405, 89411,
89420, 89421, 89429, 89435, 89448, 89454, 89457, 89473, 89476, 89484, 90068, 90073, 91182, 91
222, 91228, 91390
```

```
head (ID,10)
```

```
##    annee_2020.Iden insee2020_reg insee2020_dep
## 1            01001            84            01
## 2            01002            84            01
## 3            01004            84            01
## 4            01005            84            01
## 5            01006            84            01
## 6            01007            84            01
## 7            01008            84            01
## 8            01009            84            01
## 9            01010            84            01
## 10           01011            84            01
```

```
##* ID STEP3: assign new variable from “lookup __match tables”----
##15- Fin assigns the “insee_REG” and “insee_DEP” variables to the “annee_2020” table, at the commune level ----
annee_2020$insee2020_reg <-ID$insee2020_reg
annee_2020$insee2020_dep <-ID$insee2020_dep


##*************************************************************
  ##16- LABEL STEP0, WE CREATE a DATAFRAME “LABEL” from “$insee_DEP” and “$insee_REG” of the table “annee_2020” ----
##Initial data:
##dat <- data.frame(HouseType = c("Semi", "Single", "Row", "Single", "Apartment", "Apartment", "Row"))
LABEL <-data.frame(annee_2020$insee2020_dep, annee_2020$insee2020_reg)
head (LABEL,10)
```

```
##    annee_2020.insee2020_dep annee_2020.insee2020_reg
## 1                        01                       84
## 2                        01                       84
## 3                        01                       84
## 4                        01                       84
## 5                        01                       84
## 6                        01                       84
## 7                        01                       84
## 8                        01                       84
## 9                        01                       84
## 10                       01                       84
```

```
##* LABEL STEP 1 _ Lookup / crosswalk table: Crosswalk table from dataframe “DEP_21” and “REG_21” from insee ----
#lookup <- data.frame(type_text = c("Semi", "Single", "Row", "Apartment"), type_num = c(1, 2, 3, 4))
#lookup <- data.frame(inseecom_2021$COM, inseecom_2021$REG, inseecom_2021$DEP)
lookup_dep <- data.frame(DEP_20$dep, DEP_20$libelle)
lookup_reg <- data.frame(REG_20$reg, REG_20$libelle)
head (lookup_dep,10)
```

```
##    DEP_20.dep          DEP_20.libelle
## 1          01                     Ain
## 2          02                   Aisne
## 3          03                  Allier
## 4          04 Alpes-de-Haute-Provence
## 5          05            Hautes-Alpes
## 6          06         Alpes-Maritimes
## 7          07                 Ardèche
## 8          08                Ardennes
## 9          09                  Ariège
## 10         10                    Aube
```

```
head (lookup_reg,10)
```

```
##    REG_20.reg          REG_20.libelle
## 1           1              Guadeloupe
## 2           2              Martinique
## 3           3                  Guyane
## 4           4              La Réunion
## 5           6                 Mayotte
## 6          11           Île-de-France
## 7          24     Centre-Val de Loire
## 8          27 Bourgogne-Franche-Comté
## 9          28               Normandie
## 10         32         Hauts-de-France
```

```
##* LABEL STEP2 Create a new variable in LABEL table with plyr::mapvalues:----
##dat$house_type_num <- plyr::mapvalues(dat$HouseType, from = lookup$type_text, to = lookup$t
ype_num)
LABEL$insee2020_label_dep <- plyr::mapvalues(LABEL$annee_2020.insee2020_dep, from = lookup_de
p$DEP_20.dep, to = lookup_dep$DEP_20.libelle)
LABEL$insee2020_label_reg <- plyr::mapvalues(LABEL$annee_2020.insee2020_reg, from = lookup_re
g$REG_20.reg, to = lookup_reg$REG_20.libelle)
head (LABEL$insee2020_label_dep,10)
```

```
##  [1] "Ain" "Ain" "Ain" "Ain" "Ain" "Ain" "Ain" "Ain" "Ain" "Ain"
```

```
head (LABEL$insee2020_label_reg,10)
```

```
##  [1] "Auvergne-Rhône-Alpes" "Auvergne-Rhône-Alpes" "Auvergne-Rhône-Alpes"
##  [4] "Auvergne-Rhône-Alpes" "Auvergne-Rhône-Alpes" "Auvergne-Rhône-Alpes"
##  [7] "Auvergne-Rhône-Alpes" "Auvergne-Rhône-Alpes" "Auvergne-Rhône-Alpes"
## [10] "Auvergne-Rhône-Alpes"
```

```
##*  LABEL STEP3: assign a new variable from lookup __match tables----
annee_2020$insee2020_label_dep <- LABEL$insee2020_label_dep
annee_2020$insee2020_label_reg <- LABEL$insee2020_label_reg
head (annee_2020$insee2020_label_dep,10)
```

```
##  [1] "Ain" "Ain" "Ain" "Ain" "Ain" "Ain" "Ain" "Ain" "Ain" "Ain"
```

```
head (annee_2020$insee2020_label_reg,10)
```

```
##  [1] "Auvergne-Rhône-Alpes" "Auvergne-Rhône-Alpes" "Auvergne-Rhône-Alpes"
##  [4] "Auvergne-Rhône-Alpes" "Auvergne-Rhône-Alpes" "Auvergne-Rhône-Alpes"
##  [7] "Auvergne-Rhône-Alpes" "Auvergne-Rhône-Alpes" "Auvergne-Rhône-Alpes"
## [10] "Auvergne-Rhône-Alpes"
```

```
#17- Checking for missing values - for insee 2020 IDs: There are no missing values----
apply(annee_2020, 2, anyNA)
```

```
##                Iden               Iden1              ID_DEP          ID_COMMUNE
##               FALSE               FALSE               FALSE               FALSE
##         LIB_COMMUNE          POPULATION      POP_MUNICIPALE             DC_2018
##               FALSE               FALSE               FALSE                TRUE
##             DC_2019          DC_2018_19             DC_2020        SURMORTALITE
##                TRUE               FALSE                TRUE               FALSE
##           AGE_MOYEN            SM_10000         SMM_10000_R_              statut
##               FALSE               FALSE               FALSE               FALSE
##              PERIOD       insee2020_reg       insee2020_dep insee2020_label_dep
##               FALSE               FALSE               FALSE               FALSE
## insee2020_label_reg
##               FALSE
```

```
# Missing values for the 3 variables “DC_2018”, “DC_2019”, “DC_2020” TRUE
val_manquantes_DC_2018_19_es <- which(annee_2020$DC_2018_19 %in% "")
val_manquantes_DC_2018_19_na <- which(annee_2020$DC_2018_19 %in% "NA")
# val_manquantes_DC_2019_bis <- which(annee_2020$DC_2019 %in% NA)
# val_manquantes_DC_2020_bis <- which(annee_2020$DC_2020 %in% NA)
val_manquantes_DC_2018_19_es <- NULL
val_manquantes_DC_2018_19_na <- NULL

###########SEPARATION######################################################################
#18- Check for missing values-- Create copy of “annee_2020” table ----
#* Missing value? replace type “” and NA----
mortalite_2020_x <- annee_2020 #rename file ? recode
#*search cell with empty values “”----

#*if empty cell replace with 'x' for philcarto----
mortalite_2020_x[mortalite_2020_x == "" ] <- 'x' #replace empty cell

###########SEPARATION############################################################
##18- Faster verification, missing data NA----
#* Search for cells with NA values with the base::apply()---- package.
apply(mortalite_2020_x, 2, anyNA)
```

```
##              Iden              Iden1             ID_DEP         ID_COMMUNE
##             FALSE              FALSE              FALSE              FALSE
##       LIB_COMMUNE         POPULATION     POP_MUNICIPALE            DC_2018
##             FALSE              FALSE              FALSE               TRUE
##           DC_2019         DC_2018_19            DC_2020       SURMORTALITE
##              TRUE              FALSE               TRUE              FALSE
##         AGE_MOYEN           SM_10000        SMM_10000_R_             statut
##             FALSE              FALSE              FALSE              FALSE
##            PERIOD      insee2020_reg      insee2020_dep insee2020_label_dep
##             FALSE              FALSE              FALSE              FALSE
## insee2020_label_reg
##             FALSE
```

```
#* if cell with NA, replace with 'x' for philcarto----
head (mortalite_2020_x, 10)
```

```
##    Iden     Iden1 ID_DEP ID_COMMUNE              LIB_COMMUNE POPULATION
## 1 01001 SVG_01001     01      01001 L' Abergement-Clémenciat        794
##   POP_MUNICIPALE DC_2018 DC_2019 DC_2018_19 DC_2020 SURMORTALITE
## 1            776       1       0        0,5       1          0,5
##          AGE_MOYEN SM_10000 SMM_10000_R_         statut PERIOD insee2020_reg
## 1 76,3936042054539      6,3         6,44 Commune simple      0            84
##   insee2020_dep insee2020_label_dep  insee2020_label_reg
## 1            01                 Ain Auvergne-Rhône-Alpes
##  [ reached 'max' / getOption("max.print") -- omitted 9 rows ]
```

```
mortalite_2020_x[is.na(mortalite_2020_x)] <- 'x'   #replace cell with NA

# #################################################################"
#*package base - fonction apply()----
base::apply(mortalite_2020_x, 2, anyNA)
```

```
##              Iden              Iden1             ID_DEP         ID_COMMUNE
##             FALSE              FALSE              FALSE              FALSE
##       LIB_COMMUNE         POPULATION     POP_MUNICIPALE            DC_2018
##             FALSE              FALSE              FALSE              FALSE
##           DC_2019         DC_2018_19            DC_2020       SURMORTALITE
##             FALSE              FALSE              FALSE              FALSE
##         AGE_MOYEN           SM_10000        SMM_10000_R_             statut
##             FALSE              FALSE              FALSE              FALSE
##            PERIOD      insee2020_reg      insee2020_dep insee2020_label_dep
##             FALSE              FALSE              FALSE              FALSE
## insee2020_label_reg
##             FALSE
```

```
??apply()
```

```
## démarrage du serveur d'aide httpd ...
```

```
##  fini
```

```
############SEPARATION##################################################
#################3???????????????????????????????????????????
#19- Check variable type, then format variable names----
str(mortalite_2020_x)
```

```
## 'data.frame':    140052 obs. of  21 variables:
##  $ Iden              : chr  "01001" "01002" "01004" "01005" ...
##  $ Iden1             : chr  "SVG_01001" "SVG_01002" "SVG_01004" "SVG_01005" ...
##  $ ID_DEP            : chr  "01" "01" "01" "01" ...
##  $ ID_COMMUNE        : chr  "01001" "01002" "01004" "01005" ...
##  $ LIB_COMMUNE       : chr  "L' Abergement-Clémenciat" "L' Abergement-de-Varey" "Ambérieu
-en-Bugey" "Ambérieux-en-Dombes" ...
##  $ POPULATION        : int  794 249 14428 1723 117 2841 767 339 1132 387 ...
##  $ POP_MUNICIPALE    : int  776 248 14035 1689 111 2726 752 330 1115 376 ...
##  $ DC_2018           : chr  "1" "x" "34" "1" ...
##  $ DC_2019           : chr  "0" "x" "43" "0" ...
##  $ DC_2018_19        : chr  "0,5" "x" "38,5" "0,5" ...
##  $ DC_2020           : chr  "1" "x" "36" "1" ...
##  $ SURMORTALITE      : chr  "0,5" "x" "-2,5" "0,5" ...
##  $ AGE_MOYEN         : chr  "76,3936042054539" "x" "83,7593089475413" "51,092432373234"
...
##  $ SM_10000          : chr  "6,3" "x" "-1,73" "2,9" ...
##  $ SMM_10000_R_      : chr  "6,44" "x" "-1,78" "2,96" ...
##  $ statut            : chr  "Commune simple" "Commune simple" "Commune simple" "Commune s
imple" ...
##  $ PERIOD            : int  0 0 0 0 0 0 0 0 0 0 ...
##  $ insee2020_reg     : chr  "84" "84" "84" "84" ...
##  $ insee2020_dep     : chr  "01" "01" "01" "01" ...
##  $ insee2020_label_dep: chr  "Ain" "Ain" "Ain" "Ain" ...
##  $ insee2020_label_reg: chr  "Auvergne-Rhône-Alpes" "Auvergne-Rhône-Alpes" "Auvergne-Rhône
-Alpes" "Auvergne-Rhône-Alpes" ...
```

```
head(mortalite_2020_x, 20)
```

```
##    Iden     Iden1 ID_DEP ID_COMMUNE              LIB_COMMUNE POPULATION
## 1 01001 SVG_01001     01      01001 L' Abergement-Clémenciat        794
##   POP_MUNICIPALE DC_2018 DC_2019 DC_2018_19 DC_2020 SURMORTALITE
## 1            776       1       0        0,5       1          0,5
##          AGE_MOYEN SM_10000 SMM_10000_R_         statut PERIOD insee2020_reg
## 1 76,3936042054539      6,3         6,44 Commune simple      0            84
##   insee2020_dep insee2020_label_dep  insee2020_label_reg
## 1            01                 Ain Auvergne-Rhône-Alpes
##  [ reached 'max' / getOption("max.print") -- omitted 19 rows ]
```

```
#*- Copy, paste “PERIOD”.... variables INTO “PERIOD_N_” ...----
annee_2020$PERIOD_N_ <- annee_2020$PERIOD

#*- Copy, paste “PERIOD”.... variables INTO “PERIOD_N_” ...----
#*#*- Copy, paste “statut”.... variables INTO “statut_N_” ...----
mortalite_2020_x$PERIOD_N_ <- mortalite_2020_x$PERIOD
mortalite_2020_x$statut_N_ <- mortalite_2020_x$statut

#* Copy and paste the “SURMORTALITY” variable into “SURMORTALITY_R_” ----
mortalite_2020_x$SURMORTALITE_R_ <- mortalite_2020_x$SURMORTALITE


#******************
##20- Change variable type “PERIOD_N_” to FACTOR (qualitative variable)----
#*See Philcarto: quick start guide.pdf, New: suffix after variable name----
#*_R_ for ratio, _Q_ for quantitative and _N_ for categorical-factor-qualitative----
mortalite_2020_x$PERIOD_N_ <- as.factor(mortalite_2020_x$PERIOD_N_)


###################SEPARATION###################################################################
##########
##21-recall of departments “97” corresponds to the 5 departments of the French West Indies:
“971”, “972”, “973”, “974” and “975” ----
##* create the “ID_REG” variable ----
##22- Mortality France 2020 with 4 periods ----
#* P0  0=>[01/01,01/03[,----
#* P1  1=>[01/03,16/05[,----
#* P2  2=>[16/05,29/10[,----
#* P3  3=>[29/10,31/12]----

##23- 12 files (3*4) of Mortality France 2020 ARRONDISSEMENTS with 4 periods----
###**An extraction of sub-files by “PERIOD and ARRONDISSEMENT” ----
#* arrondissement of Marseille - Marseille district----
mortalite_2020_x_P0_arrdts <- NULL
mortalite_2020_x_P0_A_Marseille<-subset(mortalite_2020_x, PERIOD == 0 & Iden >= 13201 & Iden
< 13217)
mortalite_2020_x_P1_A_Marseille<-subset(mortalite_2020_x, PERIOD == 1 & Iden >= 13201 & Iden
< 13217)
mortalite_2020_x_P2_A_Marseille<-subset(mortalite_2020_x, PERIOD == 2 & Iden >= 13201 & Iden
< 13217)
mortalite_2020_x_P3_A_Marseille<-subset(mortalite_2020_x, PERIOD == 3 & Iden >= 13201 & Iden
< 13217)
#* arrondissement of Paris - Paris district--------
mortalite_2020_x_P0_A_Paris    <-subset(mortalite_2020_x, PERIOD == 0 & Iden >= 75101 & Iden
< 75121)
mortalite_2020_x_P1_A_Paris    <-subset(mortalite_2020_x, PERIOD == 1 & Iden >= 75101 & Iden
< 75121)
mortalite_2020_x_P2_A_Paris    <-subset(mortalite_2020_x, PERIOD == 2 & Iden >= 75101 & Iden
< 75121)
mortalite_2020_x_P3_A_Paris    <-subset(mortalite_2020_x, PERIOD == 3 & Iden >= 75101 & Iden
< 75121)
#* arrondissement of Lyon - Lyon district--------
mortalite_2020_x_P0_A_Lyon    <-subset(mortalite_2020_x, PERIOD == 0 & Iden >= 69381 & Iden <
69390)
mortalite_2020_x_P1_A_Lyon    <-subset(mortalite_2020_x, PERIOD == 1 & Iden >= 69381 & Iden <
```

```
69390)
mortalite_2020_x_P2_A_Lyon    <-subset(mortalite_2020_x, PERIOD == 2 & Iden >= 69381 & Iden <
69390)
mortalite_2020_x_P3_A_Lyon    <-subset(mortalite_2020_x, PERIOD == 3 & Iden >= 69381 & Iden <
69390)


#*#* Checking extractions ----
head(mortalite_2020_x_P0_A_Lyon)
```

```
##        Iden     Iden1 ID_DEP ID_COMMUNE             LIB_COMMUNE POPULATION
## 27136 69381 SVG_69381     69      69381 Lyon 1er Arrondissement      30142
##       POP_MUNICIPALE DC_2018 DC_2019 DC_2018_19 DC_2020 SURMORTALITE
## 27136          29352      17      19       18,0      16         -2,0
##              AGE_MOYEN SM_10000 SMM_10000_R_                  statut PERIOD
## 27136 84,3546366772533    -0,66        -0,68 Arrondissement municipal      0
##       insee2020_reg insee2020_dep insee2020_label_dep  insee2020_label_reg
## 27136            84            69               Rhône Auvergne-Rhône-Alpes
##       PERIOD_N_                statut_N_ SURMORTALITE_R_
## 27136         0 Arrondissement municipal            -2,0
##  [ reached 'max' / getOption("max.print") -- omitted 5 rows ]
```

```
head(mortalite_2020_x_P0_A_Paris)
```

```
##        Iden     Iden1 ID_DEP ID_COMMUNE              LIB_COMMUNE POPULATION
## 29295 75101 SVG_75101     75      75101 Paris 1er Arrondissement      16395
##       POP_MUNICIPALE DC_2018 DC_2019 DC_2018_19 DC_2020 SURMORTALITE
## 29295          16266       8       4        6,0       7          1,0
##              AGE_MOYEN SM_10000 SMM_10000_R_                  statut PERIOD
## 29295 58,2345072515919     0,61         0,61 Arrondissement municipal      0
##       insee2020_reg insee2020_dep insee2020_label_dep insee2020_label_reg
## 29295            11            75               Paris       Île-de-France
##       PERIOD_N_                statut_N_ SURMORTALITE_R_
## 29295         0 Arrondissement municipal             1,0
##  [ reached 'max' / getOption("max.print") -- omitted 5 rows ]
```

```
head(mortalite_2020_x_P0_A_Marseille)
```

```
##       Iden     Iden1 ID_DEP ID_COMMUNE                  LIB_COMMUNE POPULATION
## 4350 13201 SVG_13201     13      13201 Marseille 1er Arrondissement      39967
##      POP_MUNICIPALE DC_2018 DC_2019 DC_2018_19 DC_2020 SURMORTALITE
## 4350          39786      12      13       12,5      18          5,5
##             AGE_MOYEN SM_10000 SMM_10000_R_                  statut PERIOD
## 4350 77,3335683386266     1,38         1,38 Arrondissement municipal      0
##      insee2020_reg insee2020_dep insee2020_label_dep        insee2020_label_reg
## 4350            93            13    Bouches-du-Rhône Provence-Alpes-Côte d'Azur
##      PERIOD_N_                statut_N_ SURMORTALITE_R_
## 4350         0 Arrondissement municipal             5,5
##  [ reached 'max' / getOption("max.print") -- omitted 5 rows ]
```

```
### End of sub-file extractions by "PERIOD and AREA" ----
#2- Create the function encompassing the actions ----
#**Action1: loop ### extraction of 4 periods 0, 1, 2 and 3, for 18 regions: 4*18 = 72 files--
--



#** Action 2: create or update list with extracted files ----
dfs_4P_arrdt <- list(mortalite_2020_x_P0_A_Marseille, mortalite_2020_x_P1_A_Marseille, mortal
ite_2020_x_P2_A_Marseille, mortalite_2020_x_P3_A_Marseille,
                     mortalite_2020_x_P0_A_Lyon ,    mortalite_2020_x_P1_A_Lyon ,mortalite_20
20_x_P2_A_Lyon ,           mortalite_2020_x_P3_A_Lyon ,
                     mortalite_2020_x_P0_A_Paris,    mortalite_2020_x_P1_A_Paris, mortalite_2
020_x_P2_A_Paris,            mortalite_2020_x_P3_A_Paris)

paths_4P_arrdt <- c("mortalite_2020_x_P0_A_Marseille.xlsx"
                    ,"mortalite_2020_x_P1_A_Marseille.xlsx"
                    ,"mortalite_2020_x_P2_A_Marseille.xlsx"
                    ," mortalite_2020_x_P3_A_Marseille.xlsx"
                    ,"mortalite_2020_x_P0_A_Lyon.xlsx"
                    , "mortalite_2020_x_P1_A_Lyon.xlsx"
                    ,"mortalite_2020_x_P2_A_Lyon.xlsx"
                    ,"mortalite_2020_x_P3_A_Lyon.xlsx"
                    , "mortalite_2020_x_P0_A_Paris.xlsx"
                    , "mortalite_2020_x_P1_A_Paris.xlsx"
                    , "mortalite_2020_x_P2_A_Paris.xlsx"
                    , "mortalite_2020_x_P3_A_Paris.xlsx")





a<- data.frame("P0_A_13","P1_A_13", "P2_A_13",  "P3_A_13",
               "P0_A_69",     "P1_A_69",      "P2_A_69",       "P3_A_69",
               "P0_A_75",    "P1_A_75",     "P2_A_75",      "P3_A_75")

b <- data.frame("_Marseille","_Marseille", "_Marseille",  "_Marseille",
                "_Lyon",     "_Lyon",      "_Lyon",       "_Lyon",
                "_Paris",    "_Paris",     "_Paris",      "_Paris")

etiquette<-data.frame(labelarrdt=c(paste(a,b,sep="")))

str(etiquette)
```

```
## 'data.frame':    12 obs. of  1 variable:
##  $ labelarrdt: chr  "P0_A_13_Marseille" "P1_A_13_Marseille" "P2_A_13_Marseille" "P3_A_13_M
arseille" ...
```

```
#feuilles_P0 <- data.frame(feuille_carto=c("mortalite_2020_x_P0_R1", "mortalite_2020_x_P0_R
2", "mortalite_2020_x_P0_R3","mortalite_2020_x_P0_R4",
#                                         "mortalite_2020_x_P0_R6", "mortalite_2020_x_P0_R1
1","mortalite_2020_x_P0_R24","mortalite_2020_x_P0_R27",
#                                        "mortalite_2020_x_P0_R28","mortalite_2020_x_P0_R3
2","mortalite_2020_x_P0_R44","mortalite_2020_x_P0_R52",
#                                       "mortalite_2020_x_P0_R53", "mortalite_2020_x_P0_R7
5","mortalite_2020_x_P0_R76","mortalite_2020_x_P0_R84",
#                                      "mortalite_2020_x_P0_R93","mortalite_2020_x_P0_R94"))



#3- Action 3: loop to export the 72 files extracted and put in a list by period named 'dfs_P
0', dfs_P0 and dfs_P0 ----
#https://stackoverflow.com/questions/62270061/r-for-loop-write-csv-with-list-of-dataframes-an
d-path-names

#* install library(openxlsx)----
library(openxlsx)




for (i in 1:length(dfs_4P_arrdt)) {
  if(length(dfs_4P_arrdt) != length(paths_4P_arrdt)){stop("mismatch in number of data frames
and paths")}
  openxlsx::write.xlsx(dfs_4P_arrdt[[i]], file = paths_4P_arrdt[i], rowNames = FALSE, colName
s = TRUE, sheetName = etiquette$labelarrdt[i],fileEncoding = "UTF-8")
}






#######################################################

# #summary(warnings())
# The code example for the loop ------
#https://stackoverflow.com/questions/62270061/r-for-loop-write-csv-with-list-of-dataframes-an
d-path-names
# df1 <- data.frame(num = sample(1:10, 6, replace = TRUE), letter = sample(LETTERS[1:2], 6, r
eplace =
#                                                                                   TRUE))
# df2 <- data.frame(num = sample(150:160, 6, replace = TRUE), letter = sample(LETTERS[1:2],
6, replace
#                                                                                    = TRUE))
#
# list_dfs <- list(df1,df2)
#
# paths <- c("C:\\Export\\file1.csv","C:\\Export\\file2.csv")
#
# for (i in 1:length(list_dfs)) {
#   for (f in 1:length(paths)) {
#     write.csv(list_dfs[i], file = paths[f], row.names = FALSE)
#   }
# }
```

```
#*With this writing - it doesn't come out P3_A_Marseille.xlsx ----
# paths_4P_arrdt <- c(" C:/Users/aques/Desktop/_Decembre_21/R_data_ttt_2020/mortalite_2020_x_
P0_A_Marseille.xlsx"
#                      ,"C:/Users/aques/Desktop/_Decembre_21/R_data_ttt_2020/mortalite_2020_x_
P1_A_Marseille.xlsx"
#                      ,"C:/Users/aques/Desktop/_Decembre_21/R_data_ttt_2020/mortalite_2020_x_
P2_A_Marseille.xlsx"
#                      ,"C:/Users/aques/Desktop/_Decembre_21/R_data_ttt_2020/mortalite_2020_x_
P3_A_Marseille.xlsx"
#                      ,"C:/Users/aques/Desktop/_Decembre_21/R_data_ttt_2020/mortalite_2020_x_
P0_A_Lyon.xlsx"
#                      ,"C:/Users/aques/Desktop/_Decembre_21/R_data_ttt_2020/mortalite_2020_x_
P1_A_Lyon.xlsx"
#                      ,"C:/Users/aques/Desktop/_Decembre_21/R_data_ttt_2020/mortalite_2020_x_
P2_A_Lyon.xlsx"
#                      ,"C:/Users/aques/Desktop/_Decembre_21/R_data_ttt_2020/mortalite_2020_x_
P3_A_Lyon.xlsx"
#                      ,"C:/Users/aques/Desktop/_Decembre_21/R_data_ttt_2020/mortalite_2020_x_
P0_A_Paris.xlsx"
#                      ,"C:/Users/aques/Desktop/_Decembre_21/R_data_ttt_2020/mortalite_2020_x_
P1_A_Paris.xlsx"
#                      ,"C:/Users/aques/Desktop/_Decembre_21/R_data_ttt_2020/mortalite_2020_x_
P2_A_Paris.xlsx"
#                      ,"C:/Users/aques/Desktop/_Decembre_21/R_data_ttt_2020/mortalite_2020_x_
P3_A_Paris.xlsx"
#                      )
```
