# Supplementary material for: Relevance of the uMap Collaborative Platform as Support for Choropleth Mapping of a Traffic-Light Statistical Signal Atlas of All-Cause Mortality During the First French Lockdown: Geospatial Analysis
Source: JMIR Med Inform. 2026 Aug 27;14:e82855. doi: 10.2196/82855 (PMC13543287; doi:10.2196/82855)
Supplement: Supplementary file 5 [file medinform-v14-e82855-s005.zip › MA-SF_DetailedSectionOnMaterials/MA_SF_DetailedSectionOnMaterials.docx]

##### Supplementary_File – Detailed Section on Materials

The following list details the data sources, software (open, free, or proprietary), formats, and standards used, in addition to a concise list of software associated with the monitoring of the initial projects.

**Open Statistical Data Used from the French National Institute for Statistics and Economic Studies (INSEE)**

The National institute of statistics and economic studies (INSEE) disseminates data in spreadsheet format (CSV or XLSX). Three sets of raw data were extracted from May 2020, covering the period from 1 March to 15 May 2020 (including the first French lockdown):

- The data pertaining to the municipality of residence are indicative of the all-cause mortality rate of the municipal population (2017 census) for the three-year period from 2018--2020 [1, 2].
- The nomenclature of submunicipal districts (also referred to as "arrondissements" in France), municipalities (also referred to as "communes" in France), administrative departmental and regional units, and the official French geographical code (COG) (2020 vintage) are maintained by INSEE [3].
- The 2017 population census data was composed of two files. The first file, in the CSV format "municipalities.csv", lists the population of mainland France and its OSDRs, excluding Mayotte. The second file, in the XLS format "Mayotte-2017.xls", contained the data for Mayotte only [1, 2, 4]. Please refer to the supplementary files entitled 'Gross death dataset' [4] and 'Ready-to-use mortality data for the R-project program' [5].

**Open Geolocalized Data Used from the French National Institute of Geographic and Forest Information (IGN)**

The raw basemaps for 2020 from the French National Institute of Geographic and Forest Information (IGN) are in the form of shapefiles (Esri's proprietary SHP file format), which have been adapted and reworked for use in Philcarto mapping software with the help of the 'Eclat' utility [6]. There are two existing databases on French municipalities. One of these uses a less precise method to define municipal boundaries. This results in smaller files.

- The present study utilized the Admin-Express 2020 (ADE) database at the local level for the entirety of France and by territory [3], which was updated on 17 December 2019. The files dated 25 March 2020 were under the ADE Etalab 2.0 license.
- The Admin-Express 2020 (ADE) reference system was updated on 18 May 2020 and encompasses the geographical area of overseas departments and regions (OSDR) (still referred to as DOM_TOM in 2020). The update incorporated a European Petroleum Survey Group (EPSG_5490) standard, in addition to the two universal formats, ©MapInfo formats for the data file (MID) and exchange file (MIF).

**Other Open Geolocalized Data Used and Published on the French Government Website**

- The geolocated data layers for uMap were obtained from the OpenStreetMap database, which is distributed under the terms of the Open Database License (ODbL). This database is made available via the French government website dedicated to open data [7-11].

**The Data Processing Software Used Was Open: Free, Open-Source, Public Domain**

- For the analysis of the data, the structured query language (SQLite) database file format version 3.39.4 was utilized for the processing of all the data from INSEE [12].
- The R-project constitutes a language and software environment dedicated to statistics and graphics [13]. The software under discussion is a multiplatform package that is licensed under the GNU not unix a general public license, version 3 (GNU GPL). The R-project boasts a substantial ecosystem of extensions (packages), an integrated development environment (IDE), and an enterprise-grade version control system-management system (with GIT and SVN software).
- The ©RStudio environment utilized encompasses the R-Project program version v4.2.2, libraries (plyr; openxlsx), and the RMarkdown library, which furnishes a report of the processing executed by the R-Project (Figures 1, 2) [14 , 15 , 16 , 17].

**Computer-Aided Design (CAD) –Software (Open or Free)**

- The quantum geographic information system (QGIS), version 3.18.1-Zürich [18], was utilized for the purposes of our modeling and workflow processing. The tool integrates the following code versions 202f1bf7e5: It is further noted that the following libraries, APIs and projection systems are implicated: The software environment consists of Qt version 5.11.2, GDAL version 3.1.4 [19], GEOS version 3.8.1, CAPI version 1.13.3 and Projection: Rel. 6.3.2; the world geodetic system 1984 (WGS-84), Project of coordinate reference system (CRS) CRS-ellipsoid_EPSG_7030, CRS and Project-CRS EPSG_4326, as of May 1st, 2020.
- The cartographic software utilized was Philcarto version v2021.d [20], which is supported by a macro language with the file extension (PMC). This helps the preservation of a map's characteristics and the modification of its various parameters. Other export formats include Adobe Illustrator (AI), Enhanced Windows Metafile Format (EMF), and the main georeferenced format, Keyhole Markup Language (KML).
- The "Eclat" utility, developed by Philcarto [6], is employed for the purpose of preprocessing shapefiles (SHP) or universal Mapinfo data files (MID) and exchange files (MIF).
- The MapShaper software (JavaScript) was utilized to optimize the data in GeoJSON format [21].
- The Geojson.io software functions as an online Editing Tool designed for the modification of files utilizing the GeoJSON data interchange format [22].
- The execution of batch image processing operations was achieved through the utilization of the XnView and XnConvert software, which incorporated scripting languages such as SQL [23 , 24].
- The IrfanView software was utilized to perform image cropping, leveraging its capabilities for batch processing [25].

**Private Image Processing or Computer-Aided Designg Software (CAD)**

- Photoshop© version v7.0 (including ATN action script files) was utilized for the automation of the processing of the "double captions" created for the atlases in uMap [26].
- The present study employed the geographical information system (GIS) version 7.0 of Mapinfo, a system that has since been superseded by more recent developments in the field. The software was used for the management and retrieval of basemaps.
- We also used Paint© software [27] and Paint. Net© software combines the Visual Basic for Applications (VBA) language to automate drawing tasks [28 , 29].
- The SpreadsheetLight© function library was employed in conjunction with the "Eclat" utility [30].

**Free and Open-Source Collaborative Platform**

- The uMap interoperable software package (v1.2.1) [31] is a webmapping environment that is used to represent our France-wide atlases by variable. The functionality of uMap encompasses the creation of maps with OpenStreetMap (OSM) layers. The software under discussion is a collaborative mapping software platform under a permissive free software license (WTFPL), [32] (a redistributable and modifiable free software; and a free and open-source software (FOSS)) [33, 34]. uMap is hosted on the French OSM server at “openstreetmap.fr” [34] with the domain name “umap.openstreetmap.fr” [35, 36]. The project is supported by a community of developers working under the Django framework.

**Globe**

- Google Earth Pro© was used to view the Direct KML file and export it in Indirect KML format [37].

**Code Management Software (Free, GitHub Repository)**

- Visual Studio Code© (VSCode) is subject to the provisions of the Microsoft-MIT License [30].
- The view map extension for VSCode was published by Random Fractals, Inc. [38].
- The Git Bash for Windows (script command-line interpreter) provides a Bourne-again shell (BASH) emulation facility for executing the Git software (a distributed version control system) from the command line [39].

**Open Geospatial Consortium (OGC) Standards**

- The KML is a proprietary format developed by Google©. It is now recognized as the open geospatial consortium (OGC) standard. Notably, the KML specification is associated exclusively with a single projection, namely, EPSG_4326^[1]^ [40-43].
- The global positioning system (GPS) eXchange file format (GPX) is a data eXchange format that facilitates the export of data from the uMap environment and Open Geospatial Consortium (OGC) standards [44, 45].

**International Oil and Gas Producers’ Association Standards (IOGP)**

- It is important to note that the EPSG_4326 projection is associated with the KML standard [46].

**Proprietary, Open, Standardized and Brand-Specific Formats**

- The shapefile (SHP) (Esri©) is a geographic data file that conforms to the Esri shapefile specification [47].

**Proprietary, Nonstandardized, Brand-Specific Formats**

- To comprehend the intricacies of the GeoJSON format (RFC 7946 – OpenStreetMap -OSM), see the comprehensive guides and editors that are available for this format [22, 42, 43 , 48].
- It is noteworthy that the MODEL3 format is produced by the modeler® plugin of QGIS [49].
- Photoshop© action script files are saved in ATN format; they facilitate the modeling and saving of macros from Adobe Photoshop© PS7 [26, 50].
- PCM represents a format produced by Philcarto for the purpose of recording macros (script modeling) [20]. [20].

**Computer-Aided Drawing** **Software Watch (Free, Open), Collaborative Platforms—High Learning Curve**

The implementation of a monitoring period is essential for the successful management of any project, and this has led to the testing of the following software [51]:

- GIMP-V2.1 is characterized by its combination of multilingual support (including Python and Scheme) with image manipulation. It is an alternative to the proprietary Adobe Illustrator© [52].
- The Inkscape scripting extension combines the Inkscape application with the Python programming language to automate repetitive drawing tasks [53, 54].
- The ability of Mapbox© to geolocalize and convert SVG data (vector points) to GeoJSON data (geocoordinate points) was tested for import into uMap [55].

**Abbreviations**

**ADE**: Admin Express

**BASH:** bourne-again shell

**CAD:** computer-aided design

**COG:** official French geographical code

**CRS:** coordinate reference system

**EPSG:** European petroleum survey group

**FOSS**: free and open-source software

**GIS**: geographic information system

**GIT**: global information tracker

**GNU GPL:** GNU's not unix, general public license

**GPS**: global positioning system

**IDE:** integrated development environment

**IGN:** French national institute of geographic and forest information

**INSEE:** French national institute of statistics and economic studies

**IOGP:** International oil and gas producers’ association

**ODbL**: open database license

**OGC:** open geospatial consortium

**OSDRs:** overseas departments and regions (officials administrative divisions in France)

**OSM:** OpenStreetMap project

**QGIS**: quantum geographic information system

**SVN**: Apache subversion

**VBA:** visual basic for applications

**VSCode**: visual studio code

**WGS-84:** World geodetic system 1984

**WTFPL:** a permissive free software license

**Computing: file extensions**

**(AI):** Adobe Illustrator file format

**(ATN):** Adobe Photoshop actions file format

**(CSV):** comma-separated values file format

**(DWG):** drawing file format based on computer-aided design (CAD) AutoCAD

**(EMF):** enhanced Windows metafile file format

**(GeoJSON):** GeoJSON file format

**(GPX):** GPS eXchange file format

**(KML):** keyhole markup language file format

**(MID):** MapInfo data file format

**(MIF):** MapInfo interchange file format

**(MODEL 3):** QGIS model file format

**(PMC)**: Philcarto Macro Language file format

**(R):** R script file format

**(SHP):** shapefile file format

**(SQLite):** structured query language database file format

**(SVG):** scalable vector graphics file format

**(UMAP):** umap file format

**(XLS):** Microsoft Excel spreadsheet format

**(XLSX):** Microsoft Excel open XML Spreadsheet file format

**R-project and QGIS software environment packages and APIs**

\#**API (CAPI v1.13.3):** a cluster API for infrastructure provisioning in cloud

\#**API (Qt: v5.11.2):** pronounced "cute", a cross-platform application development framework for creating [graphical user interfaces](https://en.wikipedia.org/wiki/Graphical_user_interfaces)

\#**library (GDAL: v3.1.4):** The GDAL project distributes GDAL as source code

\#**library (GEOS: v3.8.1):** a C/C++ library for [computational geometry](https://en.wikipedia.org/wiki/Computational_geometry)

**#library(openxlsx):** read, write, and edit XLSX files

**#library(plyr):** tools for splitting, applying, and combining data

\#**library (RMarkdown):** dynamic documents for R

**References**

1.	INSEE. Download the file containing the total legal population for 2017 (excluding Mayotte); Population census. insee.fr; 2020 [Accessed 2025-05-26]; Available from: <https://www.insee.fr/fr/statistiques/4265429>.

2.	INSEE. Legal population of Mayotte in 2017, Population census. insee.fr; 2020 [Accessed 2025-06-12]; Available from: <https://www.insee.fr/fr/statistiques/5392668?sommaire=2120838>.

3.	IGN. ADMIN-EXPRESS-COG 2020 edition by territory: the administrative division of the French territory (municipality, departmental district, department, region...). 2020 [Accessed 2025-05-27]; Available from: <https://geoservices.ign.fr/adminexpress>.

4.	INSEE. [SupplementaryFile] Gross death file from 2017 to 2020: daily death files by sex, age and place. Insee.fr; 2017-2020 [Accessed 2025-05-27]; extracted dataset on May 22, 2020:[Available from: <https://thymine.univ-lille.fr/PoleSat_mortality_atlas/MultimediaAppendices/MA-SF_GrossDeathFile_InseeExtraction_20200522.zip;https://www.data.gouv.fr/fr/datasets/fichier-des-personnes-decedees/#/resources>; <https://www.insee.fr/>.

5.	INSEE., Quesnel-Barbet A, Pages T, Soula J, Maignant G, Hansske A. [SupplementaryFile] The ready-to-use mortality data for the R program: an example based on the 2nd version of atlas - covering four periods. Lille, France: CERIM laboratory, univ-lille.fr; 2020 [Accessed 2025-05-27]; INSEE data was extracted on 23 July 2021:[Available from: <https://thymine.univ-lille.fr/PoleSat_mortality_atlas/MultimediaAppendices/MA-SF_ready-to-useMortalityDataForR.zip>.

6.	Waniez P, Contributors. Philcarto: Eclat utility. v 2.10.h ed. Bordeaux, France: Philcarto.free.fr; 2020. p. Produit logiciel - freeware.

7.	data.gouv.fr, Lexman A. Map of French administrative departments including Overseas departments and regions (OSDRs) under ODbL license. Paris, France: data.gouv.fr; 2019 [updated 2 décembre 20192025-05-27]; 3 octobre 2021:[Available from: <https://www.data.gouv.fr/fr/datasets/carte-des-departements-2-1/>; <https://github.com/gregoiredavid/france-geojson/blob/master/departements.geojson>; <https://www.data.gouv.fr/pages/about/a-propos_data-gouv/>.

8.	data.gouv.fr, Lexman A. Map of the new French regions (created in 2016). In: Lexman A, editor. 7 février 2020 ed. Paris, France.: data.gouv.fr; 2020.

9.	data.gouv.fr, OSM-Contributors. Location of hospital intensive care units - "resuscitation-services.geojson", under ODbL licenses. Paris, France: data.gouv.fr; 2020; Available from: <https://www.data.gouv.fr/fr/datasets/localisation-des-services-hospitaliers-de-reanimation/>;

<https://www.data.gouv.fr/pages/about/a-propos_data-gouv/>.

10.	OSM-Contributors, data.gouv.fr. Location of emergency reception services: data - "emergency-reception-services.geojson", under ODbL licenses. Paris, France: data.gouv.fr; 2020; Available from: <https://www.data.gouv.fr/fr/datasets/localisation-des-services-daccueil-des-urgences/#/resources>.

11.	geo.data.gouv.fr. Switching off geo.data.gouv.fr datasets- France - Emergency medicine and authorizations. Paris, France: geo.data.gouv.fr; 2020; Available from: <https://www.data.gouv.fr/fr/posts/extinction-de-geo-data-gouv-fr/>.

12.	Hipp D-R. SQLite: Most Widely Deployed and Used Database Engine. v3.42.0 ed. North Carolina, USA: sqlite.org; 2000. p. Open-Source, not Open-Contribution.

13.	R Core Team, Chambers J, Dalgaard P, Gentleman R, Hornik K, Ihaka R, et al. The R Project. The Comprehensive R Archive Network : R for Windows. v 4.3.1 ed. Global community: cran.r-project; 1997. p. GNU GPL v2.

14.	Allaire JJ, Gutman J, Mcphers J, Cheng J, De-Vries A, Chen D, et al. RStudio IDE. In: github.com/rstudio/rstudio, editor. v 2023.06.1+524 ed. Global community: posit.co/products/open-source/rstudio/; 2011. p. Open Source (AGPL v3).

15.	Wickham HA. Plyr library: Tools for Splitting, Applying and Combining Data. In: plyr.had.co.nz, editor. v1.8.7 ed. Houston, TX, USA: cran.r-project; 2014. p. Copyright Holder: Wickham, Hadley-Alexander, MIT license.

16.	Allaire J, Xie Y, Dervieux C, McPherson J, Luraschi J, Ushey K, et al. Rmarkdown: Dynamic Documents for R. R package. In: github.com/rstudio/rmarkdown, editor. v 2.21 ed. Global community: cran.r-project.org; 2007. p. GPL-3.

17.	Walker A, Schauberger P, Braglia L, Sturm J, Garbuszus J-M, Barbone J-M, et al. Openxlsx library: Read, Write and Edit xlsx Files. In: ycphs.github.io, editor. v 4.2.5 ed. Sidney, Australia; Vienne, Austria: github.com/ycphs/openxlsx; 2014. p. MIT-license.

18.	QGIS-Development-Team. QGIS is A Free, Open Source, Cross Platform (lin/win/mac) Geographic Information System (GIS) In: github.com/qgis/QGIS, editor. v 3.18.1-Zürich ed. Global community: Qgis.org; 2021. p. CC BY-SA 3.0; GPL-2.0.

19.	Open Source Geospatial Foundation (OSGeo). GDAL: an MIT-licensed open source translator library for raster and vector geospatial data formats. © 2023 GitHub, Inc.; 2023 [Accessed 2025-05-27]; GDAL/OGR 3.7.0 May 2023:[Available from: <https://github.com/OSGeo/>.

20.	WANIEZ P. Philcarto. v2021.d ed. Bordeaux, France: Philcarto.free.fr; 2010. p. Produit logiciel - freeware.

21.	Bloch M. Mapshaper: Tools for editing Shapefile, GeoJSON, TopoJSON and CSV files. In: github.com/mbloch/mapshaper/wiki, editor. v0.6.33 ed. NY, USA: mapshaper.org; 2014. p. MPL 2.0 license.

22.	MacWright T, Whong C, Contributors. GEOJSON file editor: geojson.io. In: github.com/mapbox/geojson.io, editor. OSM-mapboxcom - Maxarcom. rolling (2014-08-11) ed. NY, USA; Global community: geojson.io; 2022. p. ISC License.

23.	Gougelet PE, Mezich T, Budz45. XnView is a general-purpose image organizer and file manager V 2.51.2 ed. Reims, France: xnview.com/fr/about/; 1998.

24.	Gougelet PE. XnConvert v 1.98 - Windows x64; v 7.99 Libformat ed. Reims, France: newsgroup.xnview.com/viewforum.php?f=79; Dec 2021. p. Proprietary license.

25.	Irfan S. Irfan Supports Windows XP, Vista, 7, 8, 10 and 11. v 4.5.9 ed. Austria: irfanview.com; 1996-2023. p. Freeware for noncommercial use.

26.	Adobe. Photoshop. v 7.0 ed. Mountain View, California, USA: Adobe.com; 1990-2002. p. Proprietary license.

27.	Microsoft. Paint v 11.2301.22.0 ed. USA: support.microsoft.com; 2000. p. Proprietary license.

28.	dotPDN-LLC., Brewster R, Contributors. Paint.NET image and photo editing software for Windows PCs. v 5.0.7 ed. Washington, USA: Getpaint.net; 2019. p. Classic, free-of-charge.

29.	Wikipedia. Visual Basic for Applications. en.wikipedia.org/wiki/Visual_Basic_for_Applications; 1993 [Accessed 2025-05-27]; Available from: <https://en.wikipedia.org/wiki/Visual_Basic_for_Applications>.

30.	Tan V. Open source developer-friendly spreadsheet library compatible with Microsoft Excel. v3.5.0 (Sept 2020) ed. Singapore: spreadsheetlight.com; 2011. p. MIT License.

31.	Boniface Y, Larlet D, Métaireau A, dependabot, Contributors. uMap-project and source code (58 contributors, 98052 user accounts). In: <https://umap-project.org/fr/>, editor. v 1.4.0 - (v 3.0.6 - 2025-05-14) ed. France: github.com/umap-project; <https://forum.openstreetmap.fr/c/utiliser/umap/29>; <https://hub.docker.com/r/umap/umap>;

<https://peertube.openstreetmap.fr/w/wfi9b4BE1ztiNM5GUAbqRc>; 2013. p. WTFPL license.

32.	Wikipedia. WTFPL license. Wikipedia.org; 2006 [updated page: 2025-04-19 - Accessed 2025-05-27]; Available from: <https://en.wikipedia.org/wiki/WTFPL>.

33.	Boniface Y. Github: umap-project - issue 1115-error 403 forbidden to our mortality atlases - four periods in 2020 In: Github.com/umap-project/umap, editor. v 1.4.0 ed. France: uMap.openstreetmap.fr; 2013. p. WTFPL license.

34.	Shahamati S, Baumann Y, Caquard S, Denieul-Pinsky L, Shaw E. uMap: A Free, Open-Source Alternative to Google My Maps. CartographicPerspectivesorg [Internet]. 2022; (99):[6–18 pp.]. Available from: <https://cartographicperspectives.org/index.php/journal/article/view/1729/2073>.

35.	Hostinger Corp. What's the Difference Between Domain Names and Web Hosting? 2020 [updated page 2025-03-03 - Accessed 2025-05-27]; Available from: <https://www.hostinger.fr/tutoriels/nom-de-domaine-et-hebergement#Quelle_est_la_difference_entre_nom_de_domaine_et_hebergement_web>.

36.	OpenStreetMap. Review 2018 OSM France services. 2018 [Accessed 2025-05-26]; Available from: <https://www.openstreetmap.fr/revue-2018-les-services-osm-france/>.

37.	Google. Google Earth For 3D representation of Earth. v 7.3.6.9345 ed. Menlo Park, California, USA: Google.com; 2001. p. Freeware.

38.	Novak T. Geo Data Viewer - ViewMap GDV-4: geographic data viewer In: github.com/RandomFractals/geo-data-viewer, editor. v 2.6.0 ed. Chicago, Illinois, USA: marketplace.visualstudio.com/items?itemName=RandomFractalsInc.geo-data-viewer; 2020. p. Apache License, V 2.0.

39.	Schindelin J, Xhmikos R, Oliff C, Contributors. Git Bash For Bash Emulation and Connect In The Secure Shell Protocol (SSH). In: github.com/git-for-windows/git/wiki/FAQ/_history?page=3, editor. v 2.40.0 ed. Global community: gitforwindows.org; 2014. p. GNU- GP License v2.

40.	Wikipedia. Keyhole Markup Language (KML): standard of the Open Geospatial Consortium (OGC) Wikipedia.org; 2005 [updated page: 2024-12-26 - Accessed 2025-05-27]; Available from: <https://en.wikipedia.org/wiki/Keyhole_Markup_Language>.

41.	W3C.org. Scalable vector graphics (SVG) version 2 features and syntax specification. Standard: Github.com/w3c/svgwg/; 2018.

42.	Butler H, Daly M, Doyle A, Gillies S, Hagen S, Schaub T. RFC 7946 - The GeoJSON Format, August 2016 - proposed standard. rfc-editor.org; 2016.

43.	geojson.org. The GeoJSON specification (RFC 7946) - a format for encoding a variety of geographic data structures. 2008 [updated August 2016 - Accessed 2025-05-27]; Available from: <https://geojson.org/>.

44.	International organization for standardization (ISO). Open geospatial consortium (OGC), Inc. MA, USA: iso.org/organization/9825.html; 2022 [Accessed 2025-05-26]; Available from: <https://www.iso.org/organization/9825.html>.

45.	Open Geospatial Consortium (OGC). The Home of Location Technology Innovation and Collaboration. 2022 [Accessed 2025-05-26]; Available from: <https://www.ogc.org/standards/>.

46.	EPSG.io, MapTiler. EPSG:4326 -- WGS84 - World Geodetic System 1984, used in GPS. 2020 [Accessed 2025-05-26]; Available from: <https://epsg.io/4326>; <https://epsg.org/home.html>.

47.	Amiar Meftah D, Geoma-sig.com. Shapefile or Geopackage: which format to choose? : © 2025 GEOMA-SIG; 2020 [updated page: 2025 - Accessed 2025-05-27]; Available from: <https://geoma-sig.com/?s=shapefile>.

48.	Dougherty J, Ilyankou I. Geospatial Data and GeoJSON and Draw and Edit with geojson.io. handsondataviz.org; 2022 [Accessed 20 August 2024]; Available from: <https://handsondataviz.org/geojson.html>; <https://handsondataviz.org/geojsonio.html>.

49.	QGIS-Development-Team. Getting Started with the QGIS' Graphical Modeler - QGIS-documentation-v3.28. docs.qgis.org/latest; 2023 [Accessed 2025-05-27]; Available from: <https://docs.qgis.org/3.28/fr/docs/training_manual/processing/modeler_twi.html>.

50.	Lifewire.com. Convert an ATN file. 2011 [Accessed 2025-05-27]; Available from: <https://www.lifewire.com/atn-file-2619722>.

51.	Grafikart.fr, Youtube.com. How do I monitor my computer? France: Grafikart.fr; 2023 [Accessed 2025-05-27]; Available from: <https://grafikart.fr/blog/veille-grafikart-organique>.

52.	Kimball S, Mattis P, Mena F, Singh M, Neumann S, Natterer M, et al. GIMP: The Free & Open Source Image Editor. In: Gitlab.gnome.org, editor. v 2.10.34 ed. Berkeley, California, USA: Gimp.org; 1995. p. CC BY-SA 4.0 License.

53.	Andler JA, Bintz J, Marquardt C, Böck H, Urošević A, Engelen J, et al. Inkscape. In: github.com/inkscape/inkscape, editor. v 1.2.2 ed. Global community: Inkscape.org; 2003. p. GPLv2 License.

54.	Pakin S. Simple Inkscape Scripting For Inkscape. © 2023 GitHub, Inc. © 2021–2025 Scott Pakin; 2021 [Accessed 2025-05-27]; Available from: <https://github.com/spakin/SimpInkScr> ; <https://inkscape.org/~pakin/%E2%98%85simple-inkscape-scripting>.

55.	Bogle B, Gundersen E. Mapbox: a collaborative platform. In: github.com/mapbox, editor. v2.15.0 ed. San Francisco, Californie, USA: Mapbox.com; 2010. p. Proprietary license.

1. EPSG:4326, also known as the WGS84 projection (because it is based on WGS84's ellipsoid), is a coordinate system used in Google Earth and GSP systems. It represents Earth as a three-dimensional ellipsoid.
