# Supplementary material for: Relevance of the uMap Collaborative Platform as Support for Choropleth Mapping of a Traffic-Light Statistical Signal Atlas of All-Cause Mortality During the First French Lockdown: Geospatial Analysis
Source: JMIR Med Inform. 2026 Aug 27;14:e82855. doi: 10.2196/82855 (PMC13543287; doi:10.2196/82855)
Supplement: Supplementary file 6 [file medinform-v14-e82855-s006.zip › BG_question2_geojson_Fig3a/3_Processings_Fig3a/1_video_VSCode_GeoDataViewer_processing/randomfractalsinc.geo-data-viewer-2.6.0/web/map.view.html]

Map View


📥 Save
⚙️ config

{ }  data

{ }  map
<> html

?
☕️

{fileName}


📤

⌸

Loading Map View...

© kepler.gl
© Mapbox
© OpenStreetMap
