# Supplementary material for: Relevance of the uMap Collaborative Platform as Support for Choropleth Mapping of a Traffic-Light Statistical Signal Atlas of All-Cause Mortality During the First French Lockdown: Geospatial Analysis
Source: JMIR Med Inform. 2026 Aug 27;14:e82855. doi: 10.2196/82855 (PMC13543287; doi:10.2196/82855)
Supplement: Supplementary file 7 [file medinform-v14-e82855-s007.zip › BG_question2_geojson_Fig3b/2_Processing_Fig3b_SMM10000_QGis/tuto/BuildingSpatialWorkflowsToIdentifyPotReddIntervQGIS_en.pdf]

USING GEOGRAPHIC INFORMATION TO SUPPORT DECISIONS ON
REDD+ GUARANTEES AND BENEFITS
MULTIPLES OF REDD+

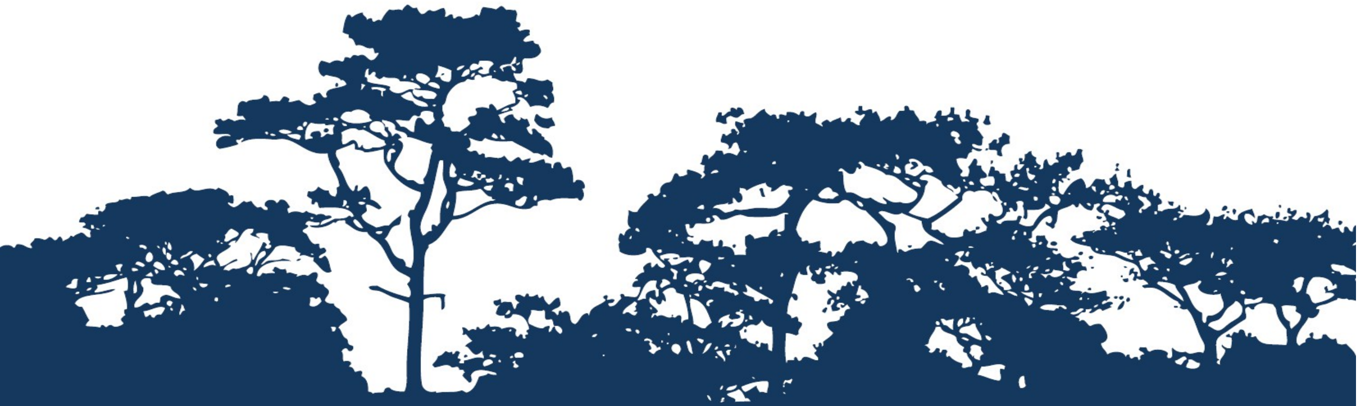

STEP-BY-STEP TUTORIAL VERSION 1.0:
BUILDING SPATIAL OPERATION FLOWS TO HELP IDENTIFY POTENTIAL AREAS FOR A REDD+ INTERVENTION USING THE GRAPHICAL MODELER IN QGIS 2.14.X

AND ADDITIONAL APPENDIX PRESENTING ADVICE ON TOOLS AND TECHNIQUES FOR PROCESSING GEOGRAPHIC DATA (GEOPROCESSING) WITH QGIS BY COMPARING RASTER AND VECTOR ANALYSIS DATA.

UN-REDD PROGRAMME
Food and Agriculture Organization of the United Nations
UNDP Empowered lives. Resilient nations.
UNEP

The UN-REDD Program is a United Nations initiative on Reducing Emissions from Deforestation and Forest Degradation (REDD) in developing countries. The Program was launched in September 2008 to help these developing countries prepare and implement national REDD+ strategies. This program draws on the mobilizing power and expertise of the Food and Agriculture Organization of the United Nations (FAO), the United Nations Development Program (UNDP), and the United Nations Environment Program (UNEP).

The United Nations Environment Programme World Conservation Monitoring Center (UNEP-WCMC) is the biodiversity assessment center of the United Nations Environment Programme, the world's leading intergovernmental organization on the environment. The Center has been operating for over 35 years and combines scientific research with practical policy advice.

Prepared by Corinna Ravilious, Charlotte Hicks, and Simon Blyth. **Copyright:** 2016

United Nations Environment Programme

Copyright permission: Provided the source is acknowledged, this publication may
be reproduced for educational or non-profit purposes without specific permission. Reuse of any figures is subject to permission from the original copyright holders. This publication may not be resold or otherwise commercially exploited without written permission from the United Nations Environment Programme. Requests for permission, stating the purpose and scope of reproduction, should be sent to the Director at the following address: UNEP-WCMC, 219 Huntingdon Road, Cambridge, CB3 0DL, UK.

Disclaimer: The contents of this report do not necessarily reflect the views or policies of the United Nations Environment Programme, the contributing organizations, or the authors. The designations employed and the presentation of material in this report do not imply the expression of any opinion whatsoever on the part of the United Nations Environment Programme, contributing organizations, editors, or publishers concerning the legal status of any country, territory, urban areas or their authorities, or the delimitation of their frontiers or boundaries, or even the designation of their names, frontiers or boundaries. The mention of any entity or commercial product in this publication does not imply endorsement by the United Nations Environment Programme.

Comments on possible errors or issues are welcome. Readers who wish to comment on this document are encouraged to do so via the following link:ccb@unep-wcmc.org .

Citation: Ravilious, C., Hicks, C., and Blyth, S. (2017) Using spatial information to support decisions on safeguards and multiple benefits for REDD+. Step-by-step tutorial Version
1.0: Building spatial operation flows to help identify potential areas for REDD+ intervention using the Graphical Modeler in QGIS 2.14.x Prepared on behalf of the UN-REDD Program. UNEP World Conservation Monitoring Center, Cambridge, UK.

Acknowledgements: These training materials were developed using elements developed as part of a joint operation with Phase II of Vietnam's UN-REDD Program to supplement spatial analyses, thereby supporting provincial REDD+ action planning in Vietnam.

Summary

1. Introduction....7

2. Mapping drivers of deforestation and forest degradation and barriers....9

3. Mapping REDD+ interventions to prevent a driver or constraint....12

4. Define a spatial logic and create a processing flow (using a REDD+ intervention example)....12

5. Use the QGIS Graphical Modeler to set up processing flows....15

5.1. Overview of the Graphical Modeler....15

5.2. Transferring a processing flow to QGIS (using the REDD+ intervention example) .16

5.3. Create a new Model in the Processing toolbox....17

5.4. Add the processing flow to the model....19

5.4.1. Add the first step to the tool - Select Natural forest in the factor extent....19

5.4.2. Edit the first step....23

5.4.3. Document the first step....28

5.4.1. Add the remaining steps in the treatment flow....31

5.4.1.1. Distance from Community Forestry sites....31

5.4.1.2. Distance to villages....34

5.4.1.3. Select natural forest within the factor range and within specific distances.... 36

5.4.1.4. Exclude strictly protected areas....39

5.4.1.5. Identify priority areas based on poverty....42

5.5. Set symbology for model outputs....46

5.6. Run the entire model and verify the results....47

5.7. Share a QGIS model with other users....53

A Appendix 1: Advisory notes on raster analysis and geoprocessing tools in ArcGIS and QGIS.... 54

A.1. Vector vs. raster....54

A.1.1. Difference between vector and raster datasets....54

A.1.2. Vector vs. raster analysis....55

A.2. Raster analysis in QGIS....65

A.2.1. Raster analysis and environment settings....66

A.2.2. Workspaces....66

A.2.3. Output coordinates....67

A.2.4. Cell size....67

A.3. An introduction to some raster analysis techniques in QGIS....68

A.3.1. Standardizing raster data....68

A.3.2. Reclassification functions....71

A.3.3. Raster Calculator: Overview....72

A.3.4. Raster calculator: Boolean intersection and No Data conversion....73

A.3.5. Zonal statistics....78

Appendix 2: Additional Resources for QGIS Graphic Modelers 79

1. Introduction

REDD+ is a voluntary approach to climate change mitigation developed by the Parties to the UNFCCC. It aims to encourage developing countries to reduce emissions from deforestation and forest degradation, conserve carbon stocks, sustainably manage forests, and enhance forest carbon stocks. This involves changes in forest use and management and may require a variety of different actions, such as protecting forests from fire or illegal logging, or rehabilitating degraded forest areas.

REDD+, beyond the issue of carbon, can bring a number of benefits. For example, it can promote biodiversity conservation and secure forest ecosystem services such as water regulation, erosion control, and non-timber forest products (NTFPs). Some of the potential benefits of REDD+, such as biodiversity conservation, can be amplified by using spatial analysis and other approaches to identify areas where REDD+ actions would have the greatest impact.

The purpose of this series of tutorials is to guide participants who are already proficient in GIS in conducting analyses related to REDD+ during technical working sessions. The tutorials have been used in many countries to build capacity to produce datasets and maps relevant to their spatial planning for REDD+ and to develop other similar types of maps. The maps obtained using these approaches appear in numerous publications that aim to support the planning of strategic options that improve biodiversity and ecosystem services and reduce climate change (see http://bit.ly/mbs-redd for resources for the country concerned). Of course, countries are not obliged to use the approaches described in these tutorials.

Spatial analysis can be used to support decision-making, particularly with a view to identifying areas potentially suitable for REDD+ interventions (REDD+ actions), but maps alone are not sufficient for making such decisions. This does not mean that it is useful to map all potential interventions; however, if done in conjunction with methods such as stakeholder consultation and participatory mapping approaches, map analysis can be useful in selecting REDD+ approaches and where to apply them.

This tutorial focuses on how to use spatial analysis workflows to identify potential areas where a REDD+ intervention (often referred to as a REDD+ action or REDD+ measure) could be implemented to address a specific constraint or driver. Certain interventions will be more suitable for specific drivers and sets of constraints.

This tutorial uses land surface imagery to explain how to create and run an analysis using workflows in the QGIS Graphical Modeler. It covers: mapping drivers of deforestation and forest degradation and barriers to "+" activities; mapping REDD+ interventions; defining spatial logic and creating workflows; sharing the resulting model; and repairing a

malfunctioning model. The appendices provide more information on raster data formats and processing for those who are accustomed to vector GIS data. They will also guide users in exploring the various raster analysis tools and methods available in the QGIS geoprocessing toolbox.

Appendix 1 provides a brief summary of the differences between raster and vector data and an introduction to getting started with raster data analysis. Raster analysis provides users with access to many of the tools and functions that are available for vector analysis. Many of these tools are particularly suited to multi-criteria analysis. Raster functions can also be used to generate new data (e.g., hydrology and slope data from digital terrain models). It also includes a comparison table showing the equivalent ArcGIS tools.

Appendix 2 provides links to additional resources and help related to the QGIS Graphical Modeler.

A similar tutorial using ArcGIS as an alternative platform is available at http://bit.ly/GIStools-redd:
Ravilious, C., Hicks, C., and Blyth, S. (2016) Using spatial information to support decisions on safeguards and multiple benefits for REDD+. Step-by-step tutorial Version
1.0: Building spatial workflows to help identify potential areas for REDD+ intervention using a pattern generator in ArcGIS 10.x. Prepared on behalf of the UN-REDD Programme. UNEP World Conservation Monitoring Center, Cambridge, UK.

2. Mapping drivers of deforestation and forest degradation and barriers

When we think about the factors contributing to deforestation and forest degradation and the obstacles to "plus" activities (conservation, improvement, sustainable management), we often refer to the current situation (e.g., what are the current factors and where are changes in shrub cover already being observed). But it is also important to think about future obstacles and future pressures and threats to the forest and how they could lead to deforestation or forest degradation.

To identify locations for REDD+ interventions, it is important to consider both the location of current obstacles and factors and the existence of pressures and threats to the forest in order to identify locations where factors and obstacles may be encountered in the future.

Factors and barriers can be direct or indirect. Examples of **direct factors** include infrastructure development, agricultural development, fires, mining activities, and plantation development (e.g., rubber, palm oil). A direct barrier may be a physical feature (steep slopes, rocky terrain, distance from roads) that complicates reforestation. Examples **of indirect factors and barriers** include: changes in population size (possibly indicating increased demand for land and natural resources), poverty levels (possibly reflecting direct dependence on natural resources), financial incentives (e.g., commodity prices and subsidies that could make certain land uses more attractive), cultural preferences (perhaps defining the use of natural resources), and political decisions (determining the distribution and control of land use).

Factors driving change in shrub cover/forest quality could lead to changes in forest functions, biodiversity levels, ecosystem service provision, and support for forest livelihoods.

To identify and map areas affected or at risk of being affected by factors and barriers, we can:

- Use a participatory approach, involving multiple stakeholders from different sectors, organizations, and locally relevant communities to identify factors, barriers, and affected or at-risk areas.
- Map direct and indirect pressures on the forest, examine their relationship with
  shrub cover and changes in this shrub cover, and analyze their potential for change in the future.
- Map areas that are likely to be affected by specific factors or obstacles in the future (e.g., planned deforestation due to infrastructure development proposals or changes in land use).
  obstacles in the future (e.g., planned deforestation due to proposed infrastructure development or changes in land use).
- Map current areas where conservation, enhancement, and sustainable management activities
  sustainable management are already being carried out and examine the relationship between factors that either hinder or promote their success.

- Map areas with physical, environmental, and socioeconomic potential for promoting "more" activities.

Maps showing individual layers of future pressures/threats and maps using simple data overlays can be created to show where factors coincide. This does not "select" particular areas, but these maps can make an important contribution to stakeholder workshops where participants identify areas suitable for REDD+ interventions based on their advanced local knowledge.

Maps showing areas that meet certain criteria can also be created. For example, a map showing areas at risk of small-scale agricultural development could highlight areas based on the presence of existing agriculture, areas designated for future agriculture through land use plans, and economic factors such as poverty and population.

This approach may be more subjective in terms of the criteria and thresholds used and needs to be validated by experts with local knowledge. If the knowledge needed to develop the map processing flow is poor or if the processing flow is not properly validated, it can lead to the use of erroneous data. Assumptions must be presented transparently so that planners and stakeholders understand the data used to create the map and how it influences the areas selected by the spatial analysis process. This can be done by indicating the input layers or assumptions in a series of maps in the appendix; this makes the method of selecting areas, the input layers used, and the criteria applied clearer. Alternatively, the input maps can be presented alongside the final product, as in the map example below, where the input layers are shown to the right of the final map.

This map shows areas where forests are particularly important for limiting soil erosion that might cause sedimentation problems for dams in Tanzania. The methodology is based on four parameters: slope, precipitation, locations of dams and water bodies and their catchments, and forest.

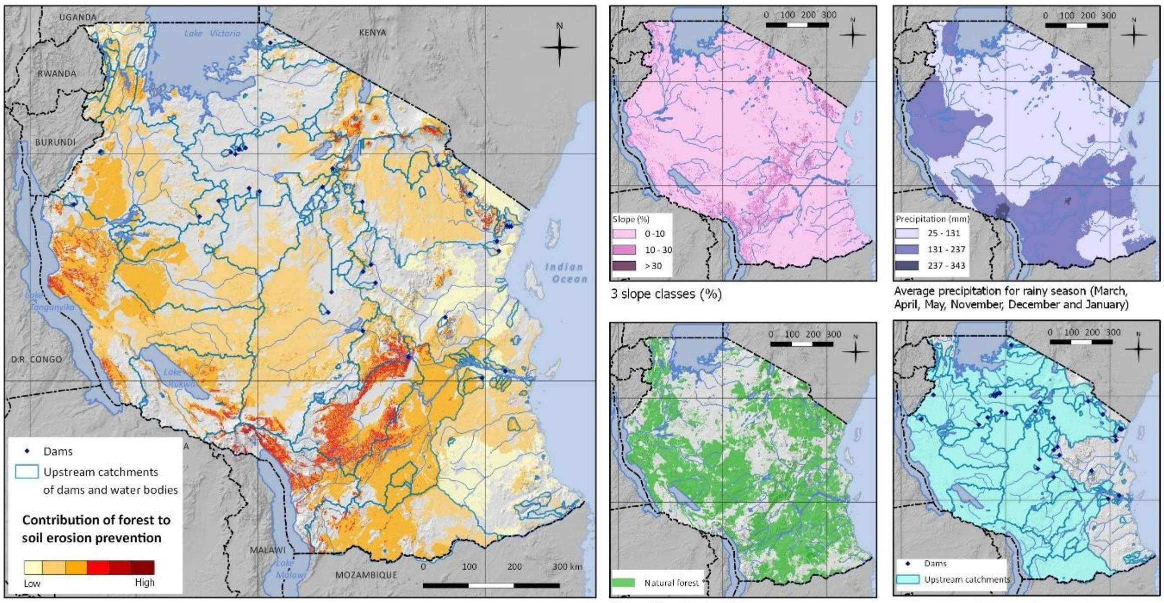

3 slope classes (%)

Average precipitation for rainy season (March, April, May, November, December and January)

Natural forest extent*

* Defined according to the national forest definition to include the following land cover classes: montane and lowland forest, open and closed woodland, mangroves, thickets and bushlands.

Upstream catchments of dams and water bodies

Map projection: WGS84 / UTM Zone 36S. Map prepared by Tanzanian Forest Service (TFS), UNEP-WCMC, FAO, Sokoine University of Agriculture (SUA) and Forestry Training Institute (FTI). Date: May 2013.

Data Sources:
Natural forest and water bodies: NAFORMA. 2013. NAFORMA land-use / land-cover Map 2010.
Dams: Dr. Mark Mulligan, Department of Geography, Kings College, London.
Slope: generated from Lehner, B., Verdin, K., Jarvis, A. 2008: New global hydrography derived from spaceborne elevation data. Eos, Transactions American Geophysical Union, 89 (10) (2008) 93-94. Journal Article.
Precipitation: Hijmans, R.J., S.E. Cameron, J.L. Parra, P.G. Jones and A. 2005. Jarvis. Very high resolution interpolated climate surfaces for global land areas. International Journal of Climatology 25 (2005): 1965-1978. Journal Article.

3. Mapping REDD+ interventions to prevent a factor or obstacle

Both participatory and spatial analysis approaches could play a role in mapping priority areas for REDD+ interventions. During the development of the Provincial REDD+ Action Plan (PRAP) in Vietnam, for example, both approaches were used, with workshop participants first identifying specific intervention areas and spatial analysis teams conducting more in-depth GIS analyses.

For the spatial analysis approach, two main steps are necessary:

1. **Define the spatial logic or processing flow to answer a specific question:** The processing flow will specify which datasets and geoprocessing tools are needed to produce the desired final map. This processing flow must also take into account a) the area affected by/at risk of being affected by the factor/obstacle and b) the results of participatory mapping of interventions.
2. **Execute the processing flow in the QGIS Graphical Modeler.** By executing the geoprocessing steps in a single tool, the analysis can be performed in fewer steps, or even in a single step.

4. Define a spatial logic and create a processing flow (using a REDD+ intervention example)

Defining **processing flows** allows you to think about **how you will perform an analysis**: the spatial logic, the technical GIS processes, and the sequence of steps.

This section of the tutorial focuses on the thinking and preparation required before building a workflow in the QGIS Graphical Modeler. To help identify potential locations for a particular REDD+ intervention, a workflow would combine factors in a multi-criteria analysis to **include** areas **suitable** for the intervention and **exclude** those that are **not**.

Considering the questions below can help clarify the input layers, other data, and processes you will need in your processing pipeline:

- Where are the areas affected/at risk of being affected by the factors or barriers? The location of interventions should be chosen based on the location of the factors/barriers; it can be based on previous maps developed to show factors/barriers or on other knowledge that can be used to determine affected areas. This could be a large part of the land area; for example, the entire forest in the province could be affected by a barrier such as a lack of monitoring resources.
- What physical aspects will affect the implementation of the intervention? For example, slope, soil type, forest type, climate.

- What other aspects could affect its feasibility? For example, site accessibility, carbon stocks, forest condition, or risks that could hinder the success of the intervention.

  the intervention (e.g., risk of fires).
- What is the potential for increasing the benefits of the intervention? For example, can it contribute to poverty reduction, biodiversity conservation, or the provision of

  ecosystem services?
- What social and environmental risks are associated with the intervention? What is the potential for risk reduction and conservation support: for example, preventing

  conversion of natural forests or reducing the risk of displacement (i.e., reducing the risk of displacement of deforestation or degradation to other areas)?

In the example of Vietnam, much more relevant information should have been collected during the PRAP process. For example, previous analyses and participatory mapping of drivers and constraints can help identify the area with the driver/constraint. At the same time, social and environmental risks and benefits assessments should facilitate the identification of the risks and benefits potentially associated with each intervention.

Identifying areas suitable for specific interventions can provide additional information and, with stakeholder engagement, be used in conjunction with other participatory methods. For example, if a participatory mapping approach has been used to define potential areas, is it necessary to carry out further analysis? This may vary from one intervention to another. Participatory maps can provide an overview, for example by identifying areas at an administrative level (e.g., the municipality) and thus providing some criteria that could, if necessary, offer a more detailed spatial analysis to complement the participatory results.

When defining the spatial logic to be used for the processing flow, it is often useful to first consider the areas where **REDD+ intervention cannot be carried out,** in order to **exclude these areas** from the possible scope of REDD+ intervention. This approach makes it easier to identify the areas within the remaining areas where REDD+ intervention **can be carried out**. It is often easier to do this before identifying **the locations where REDD+ interventions can be implemented** in the remaining areas**.**

When considering the spatial analysis to be carried out, you need to identify the criteria and data you will use to make these exclusions/inclusions, the geoprocessing tools you can use in QGIS, and the availability of data (particularly at the right scale).

It is important to document the reasons why you are excluding or including certain areas and to specify which factors were used to determine these areas. This will ensure a clear and transparent presentation of the analysis to decision-makers so that they can understand how a map was created and the assumptions that guided its creation.

In this tutorial, an example of a processing flow will be presented for the development of a REDD+ intervention layer: this will be intended for **sustainable community forestry to counteract the factor of "small-scale conversion of forest to cassava,"** based on the fact that forests that are valuable to the community are less likely to be converted to agricultural land.

To define a processing flow that addresses this issue, we can consider the following questions:

- Where are the areas at risk of small-scale cassava development (now and in the future)?
- Where can sustainable community forestry be practiced? In our examples, areas close to villages are considered more suitable.
- What types of forest area designations should be included? In our example, strictly protected areas are not available for community forestry.
- Should the intervention take place in natural forests and/or planted forests? In our example, natural forest is preferable.
- Should the intervention be limited to community forestry areas or can we look further afield? In our example, areas close to existing or planned community forests are preferred.
- What benefits and risks are associated with the intervention? Can they be mapped? In our example, poverty reduction was the only additional benefit considered.
  considered.

The GIS analysis **would** then **exclude** areas not exposed to the factor and areas where it is not possible to carry out a particular action, and **would include** areas considered suitable where benefits could be increased and risks reduced. The table shows the contribution layers for an example treatment flow:

**Type of data/contribution layers**	**How to use them?**	**Name of the dataset in the Mythical folder _Database**
Shrub cover	Available natural forest area for actions	Natural_forest_mythical
Village locations	Areas near villages with likely demand for cassava production and/or community forestry activities (SC).	Village_mythical
Existing and potential community forests	Forest category; areas already identified as current or potential SC sites.	Community_forestry_mythical
Protected areas	Excludes strictly protected areas	PA_mythical
Extent of factor	Area affected by/at risk of being affected by a factor (conversion to cassava) *(in this example, a future Land Use Plan was used to identify large areas intended for agriculture)*	future_LUP_agriculture_mythical
Poverty reduction potential (as benefit of the intervention)	Areas with medium-high poverty rates	Communes_mythical (using the pov_hml field, values: (1) low (2) medium and (3) high

The processing flow can be done manually in a flowchart before being executed in QGIS. In this example, we will use a processing flow designed for ArcGIS to show how equivalent tools and analyses can be found and to adapt and implement the processing flow in QGIS. The example related to the above intervention is presented below.

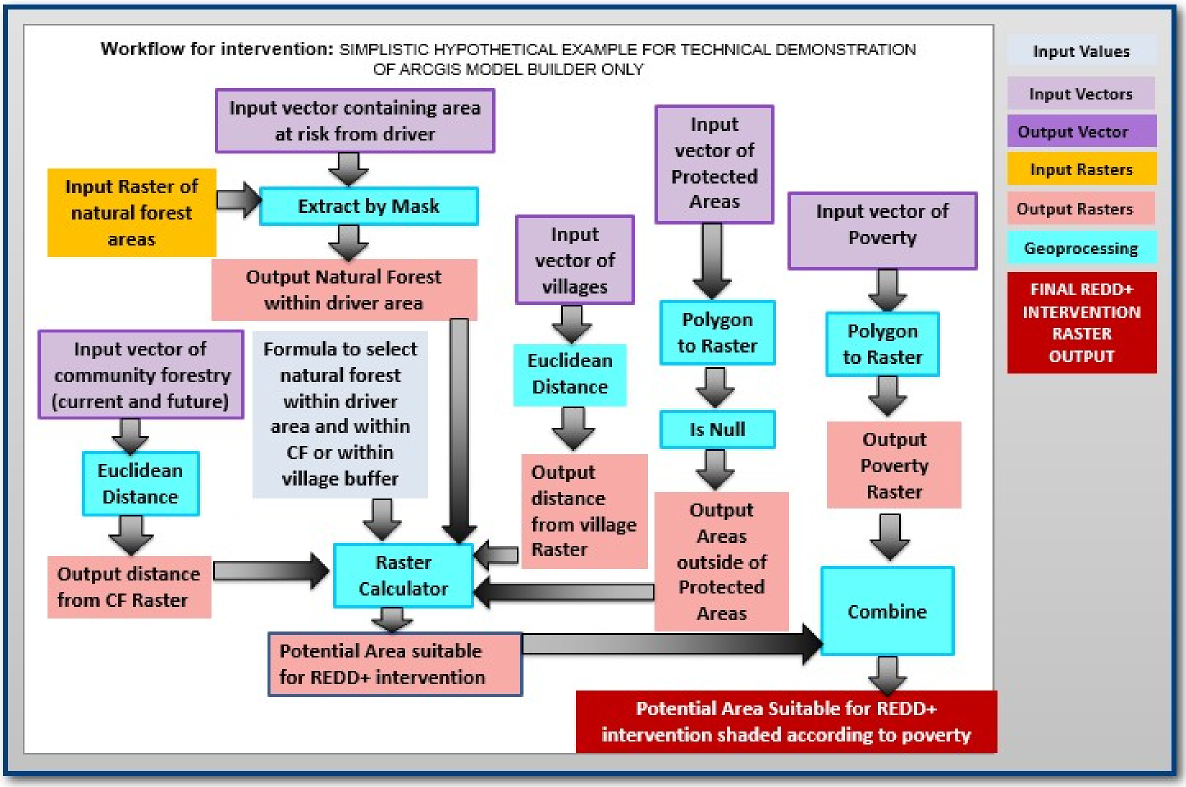

5. Use the QGIS Graphic Modeler to set up processing flows

5.1. Overview of the Graph Modeler

The Graphical Modeler is an application that you can use to **create, publish**, and **manage models**. It is equivalent to the ArcGIS Model Builder. Models in QGIS are processing flows that bring together a series of geoprocessing tools, transferring the result of one tool to the next. The benefits of using the Graphical Modeler lie in better organization, which improves data flow and speeds up analysis. By standardizing processes in this way, any repetition of an analysis is greatly facilitated, allowing for direct replication and comparison of results.

A simple model would only take into account one or two steps or one or two QGIS geoprocessing tools. For example, the model below classifies the forest according to its distance from roads:

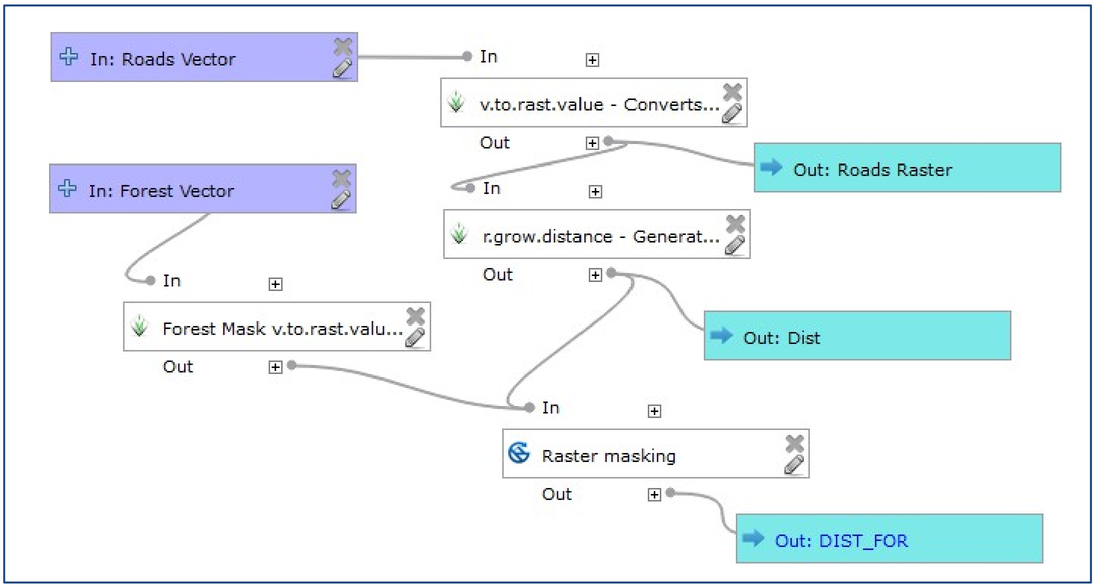

5.2. Transferring a processing flow to QGIS (using the REDD+ intervention example)

a. Open **QGIS**

b. From the main menu, click on **Processing >> Options >> General**

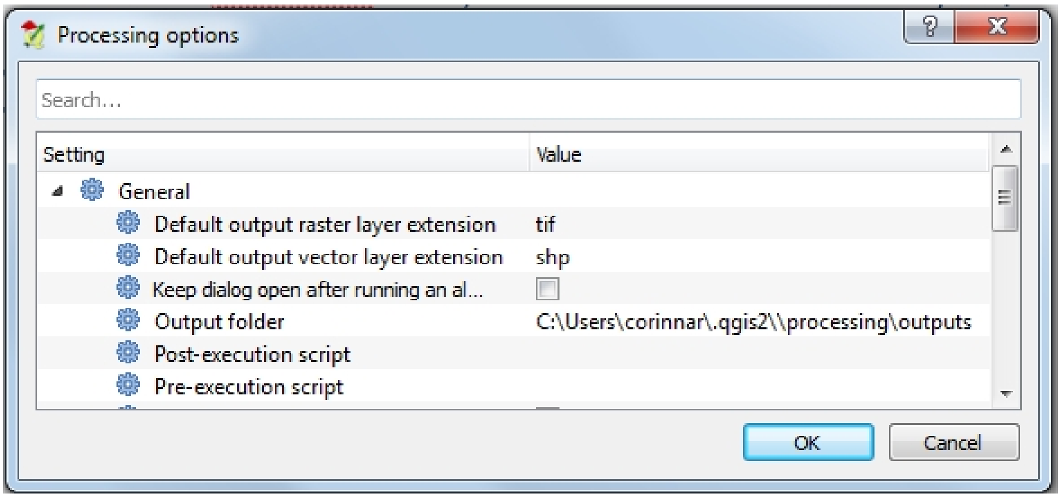

c. The default output location for saving models is the **qgis2** folder. You can change this if you want to save them elsewhere, but **make a note of the location**. All your QGIS project models will be stored in this location.

d. Click **OK** to close the window.

e. Copy **MODEL_BUILDER_DEMO** to your computer.

f. From the **MODEL_BUILDER_DEMO** folder**,** open the **Mythical_Model_Builder.qgs** project**.**

g. Familiarize yourself with the data in the **Table of Contents** (on the left side of the panel) in QGIS.

The data used as examples in this tutorial is stored in the **Mythical_Database** folder. The table below lists the data layers contained in the folder:

communes_mythical – Administrative Boundaries (Municipalities)	community_forestry_mythical – Current and Proposed Community Forestry Sites communautaires actuels et proposés
DEM_mythical – Digital Elevation Model	communes_mythical – Administrative Boundaries (Regions)
future_LUP_agriculture_mythical – Land Use Plan future of land in terms of agriculture	HILL_mythical – Hill shading generated from the Digital Terrain Model
hydrobasins_mythical – Hydrological basins	hydropower_mythical – Planned and existing hydroelectric sites planned and existing
lakes_rivers_mythical – Water bodies and rivers	land_concess_mythical – Land concessions for economic purposes
landuse_mythical – Current land use	market_mythical – Market locations
natural_forest_mythical – A subset of the current land use layer (which contains only the classification of natural forests)	PA_mythical – Protected areas
mining_concessions_mythical – Mining concessions mining	roads_mythical – Existing and planned roads
prod_for_mythical – Production forest	Poverty rate_mythical – Poverty rate by municipality
village_mythical – Village locations	

Note: Not all of these datasets will be used in the demonstration analysis, but they can be used as test data when developing other processing flows.

5.3. Create a new Model in the Processing toolbox

In QGIS, models are stored in user-defined groups. The group is specified when creating a new model.

a. To create a new model, from the main menu click on **Processing>>Graphical Modeler**

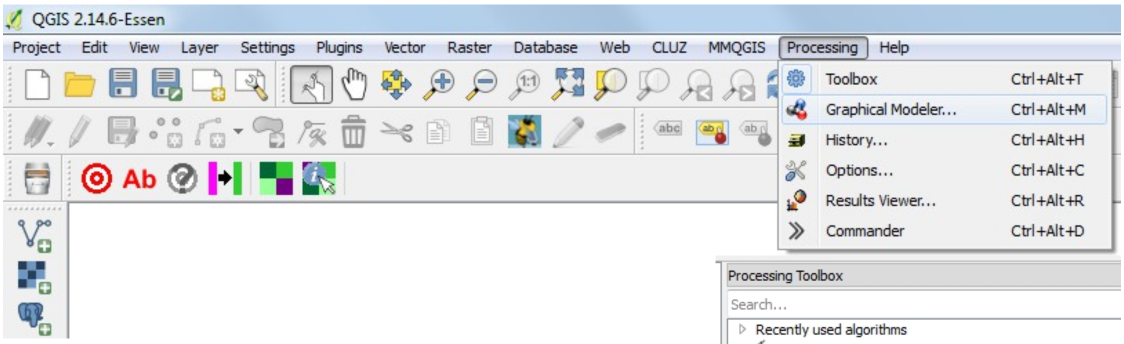

*Otherwise, do it from the **Geoprocessing Toolbox**, expand **Templates**, expand **Tools**, and then click **Create New Template**.*

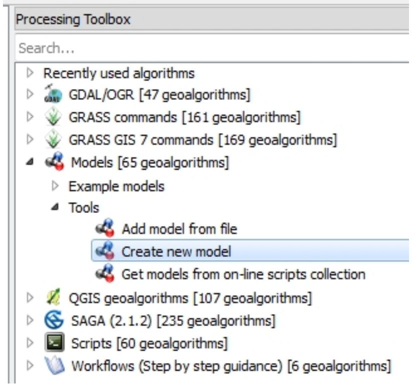

The processing modeler window appears.

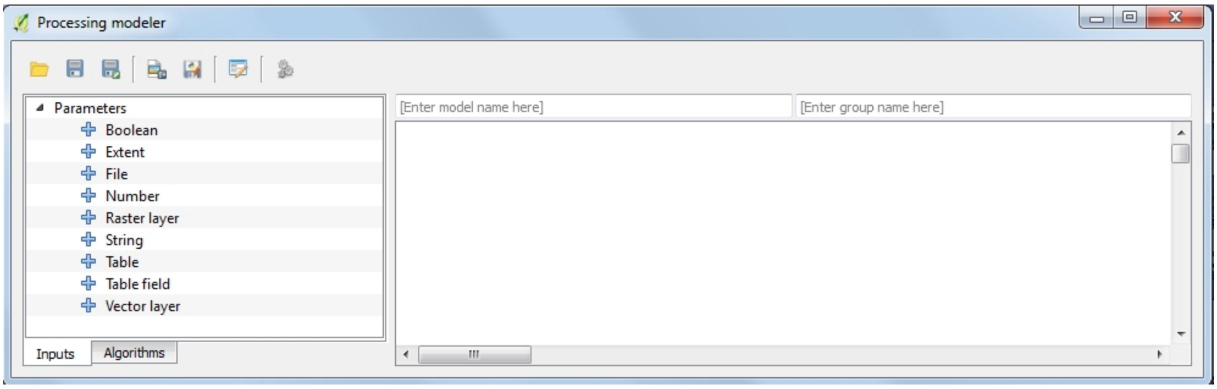

Text boxes are available at the top to specify the name of the template and the group to which it belongs. The group could contain templates related to a particular theme or developed for a particular project, for example.

a. Give the model a name, e.g., **SC Intervention Demo to Counter the Cassava Factor.**

b. Give the group a name, e.g.**, REDD+ intervention processing flow.**

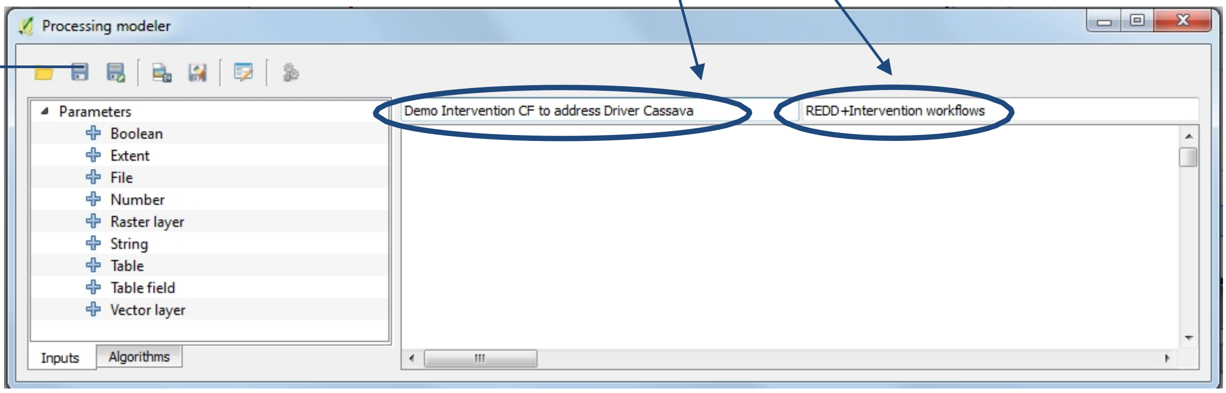

c. Click the **Save As** button and save the template. It will be saved in the default location unless you chose a different one in step 5.2b, e.g. **C:\Users\xxxxxxxxxxxx\.qgis2\processing\models**

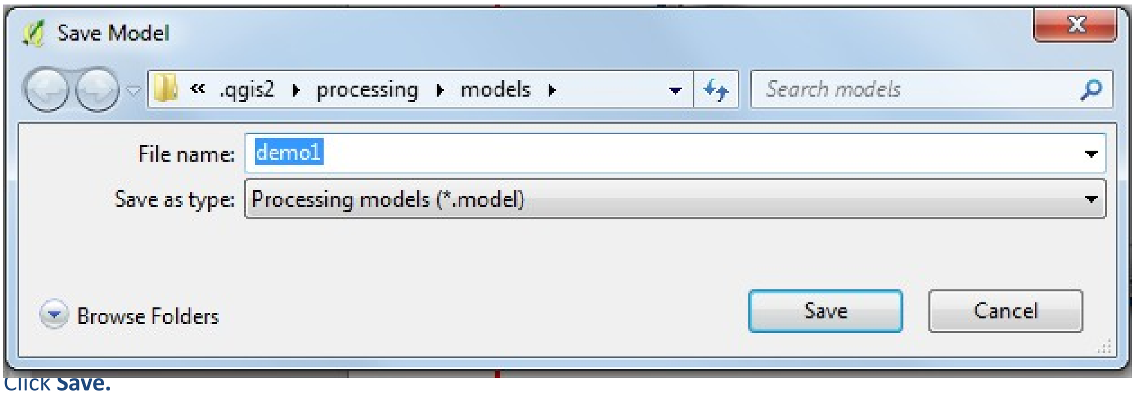

d. Click **Save.**

e**.** Close the model by clicking on the **cross** at the top right of the **processing modeler** window.

In the **Processing Toolbox,** check that your new model group (**REDD+ intervention processing flows**) has appeared as a subset of **Models** and that your new model is located in this set.

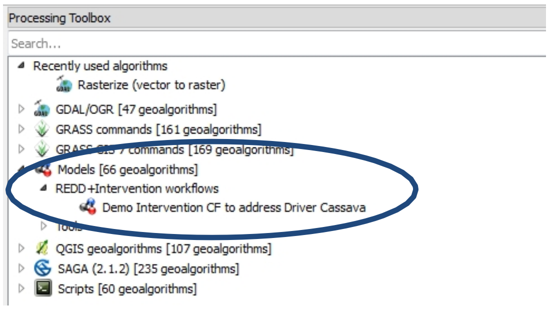

5.4. Add the processing flow to the model

We will use the example processing flow developed and described in **Section 4** to perform this demonstration.

5.4.1. Add the first step to the tool - Select the Natural forest in the factor range

The first step is to select areas likely to be affected by the factor*
"conversion to cassava" and, within these areas, ensure that the "Community Forestry" intervention can only be carried out in natural forest areas.

*(*In this example, a shape file already exists for the factor area).*

a. In the **geoprocessing toolbox,** right-click on the model you created in the previous steps and click

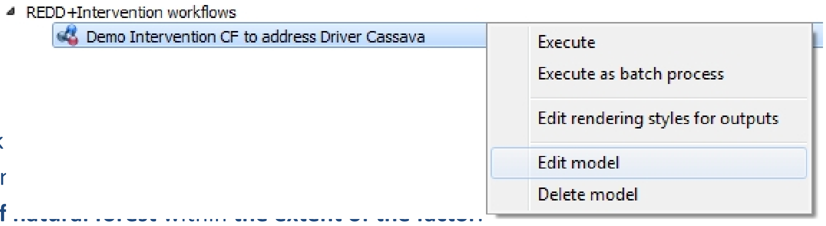

Take a look at your planned processir [illegible]
"Mask extraction" to cut out **areas of** [illegible]

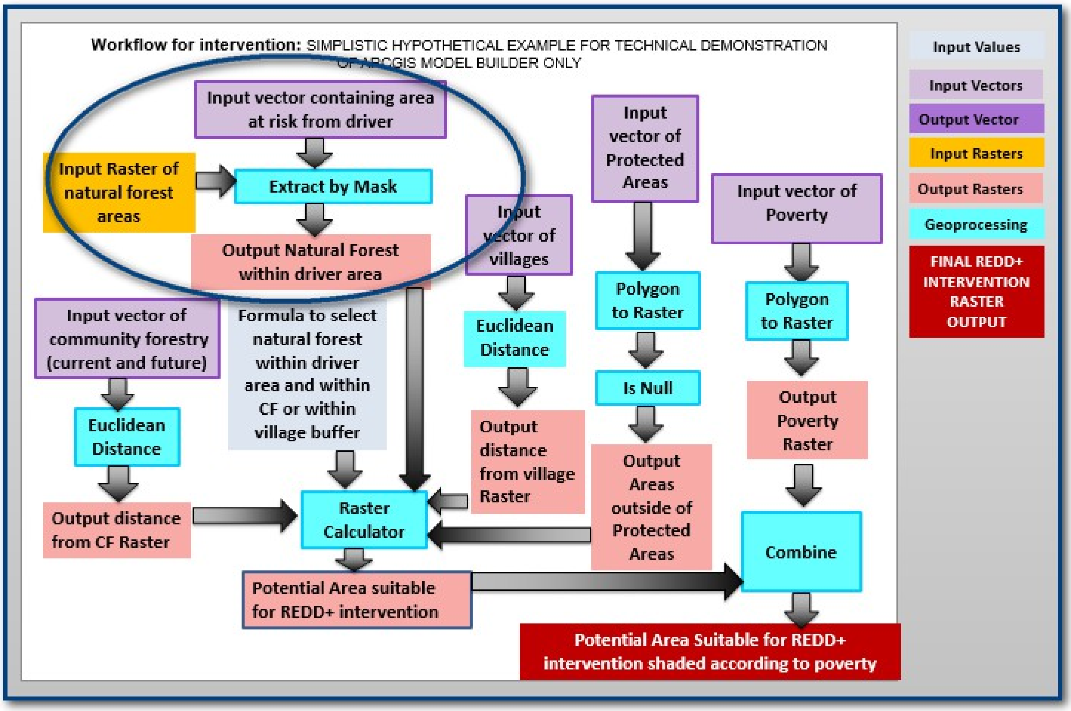

b. Remember that the processing flow was designed for ArcGIS, so the names of the tools may be slightly different. In QGIS, if we click on the **Algorithms** tab and search for an **Extract** function, we will not find any geoprocessing tools named **Extract by mask**, but if we search for a **cut** function, we can find two tools that can work in a similar way: **Clip raster with mask layer (GDAL)** and **Clip raster with polygons (SAGA)**.

c. Both of these tools should allow us to extract an area of interest, but for this example, we will use the **SAGA** tool. Double-click on the **Clip raster with polygon** tool**.**

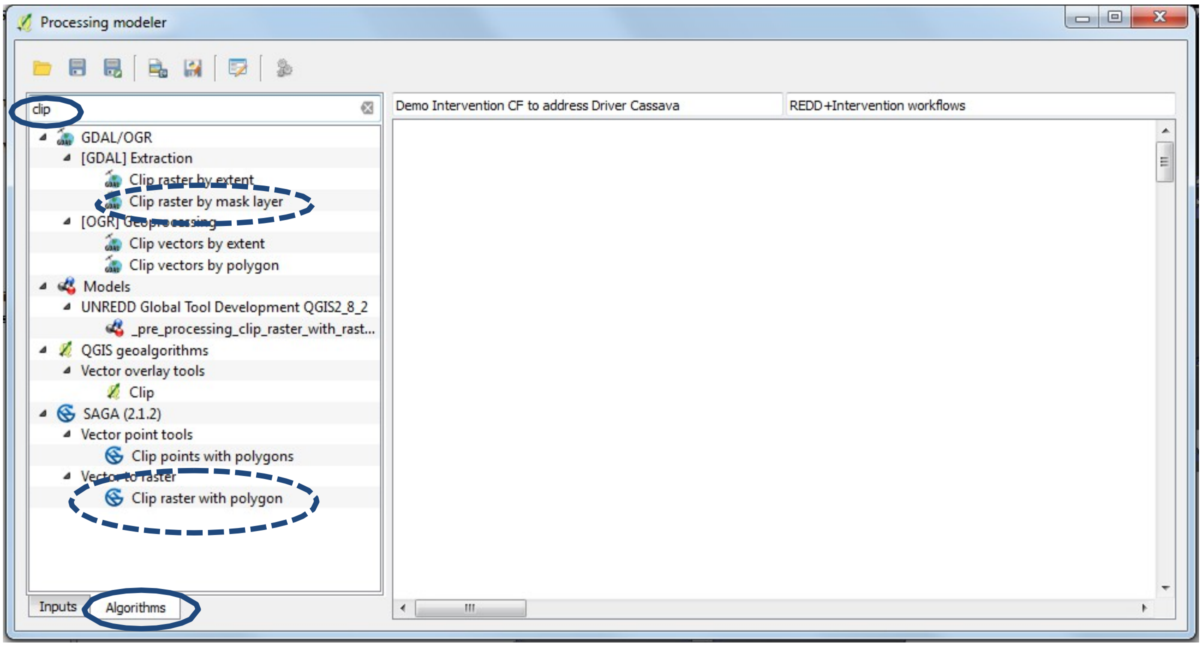

A window appears; it is similar to the dialog box that opens when you use the tool directly from the ***processing toolbox.***

d. Try selecting an **Input** (raster) and a **Polygons** layer (layer for clipping). You cannot select this data because you must insert it into your model as **Input** in the **Clip raster with polygon** tool.

e. Click **Cancel** to return to the **graphic modeler** window.

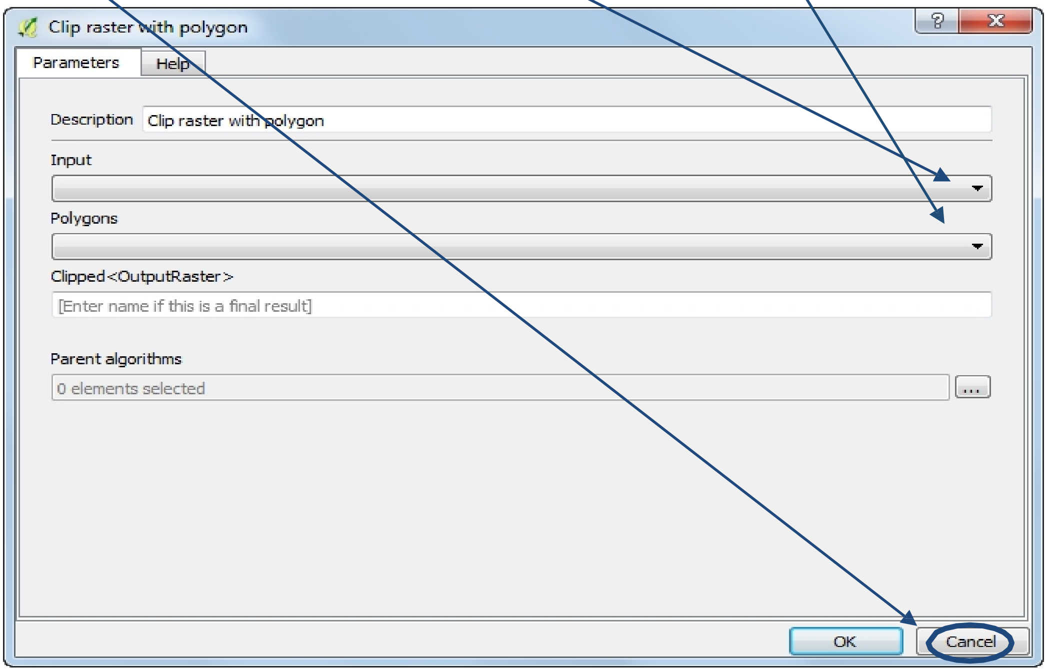

f. Click on the **Inputs** tab.

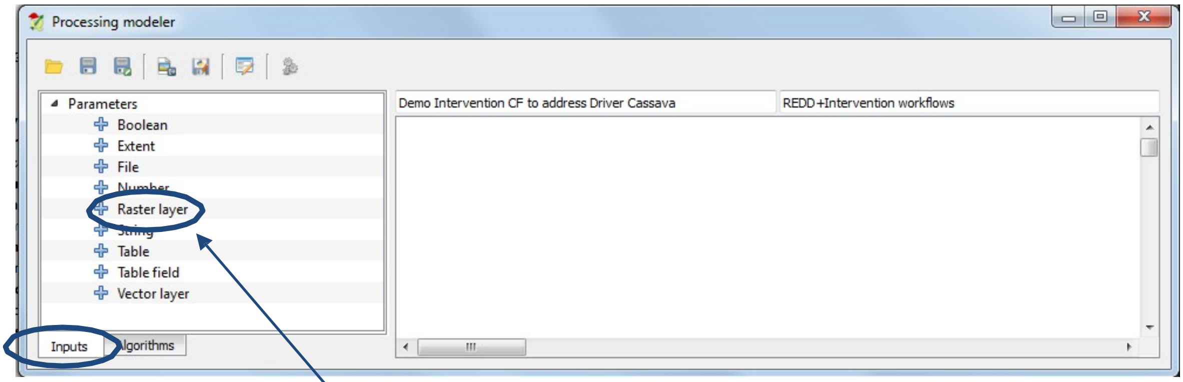

g. Double-click on **Raster layer** in the list of parameters.

h. Give the parameter a name (this is the name that will appear in the template dialog box when you run the tool). In this example, enter **Natural Forest Raster to Cut**. The dataset is required for the tool, so leave **Required** by default set to **Yes**.

i. Click **OK** and the Raster Parameter is added to the template canvas.

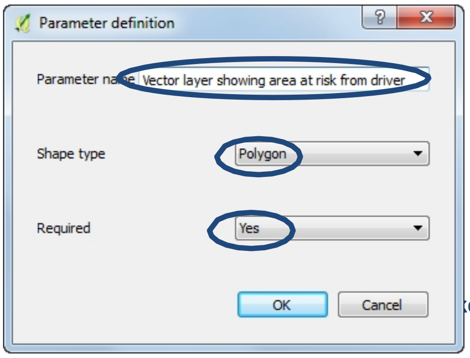

j. Double-click **Vector layer** to add another parameter. (i.e., the polygon layer to be used as a clipping mask).

k. Give a name to the [illegible] **[illegible]ely to be affected by** [illegible]

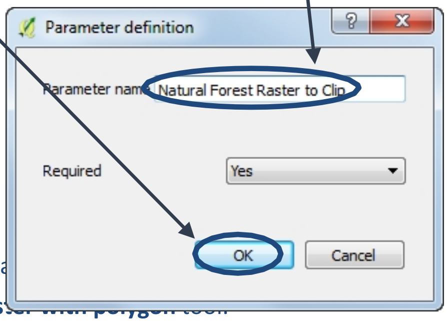

l. Change the shape type to **Polygon.**

m. Leave **Required** set to **Yes.**

n. Click **OK** and the Vector Parameter is added to the template canva[illegible]

o. Click on the **Algorithms** tab again and double-click on the **Clip ras[illegible]** tool.

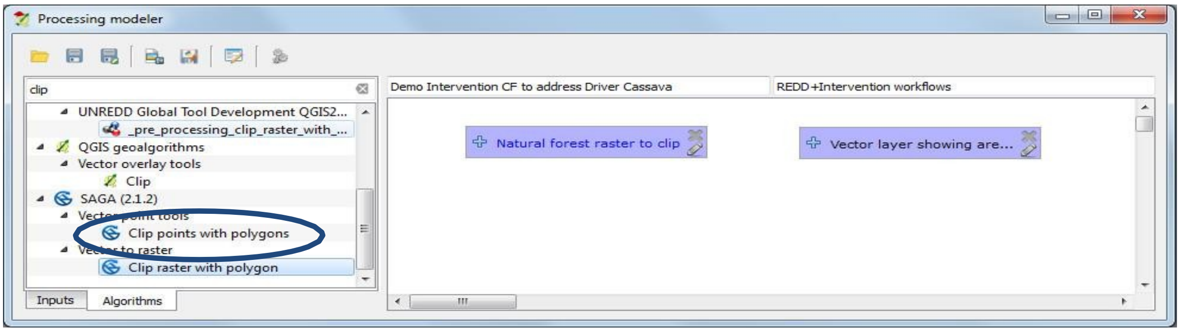

p. Change the description to, for example, **Step 1: Cut out the natural forest according to the extent of the factor.**

q. Enter a name for the output parameter value, e.g. **OutputRaster: natural_forest_in_driver_area**

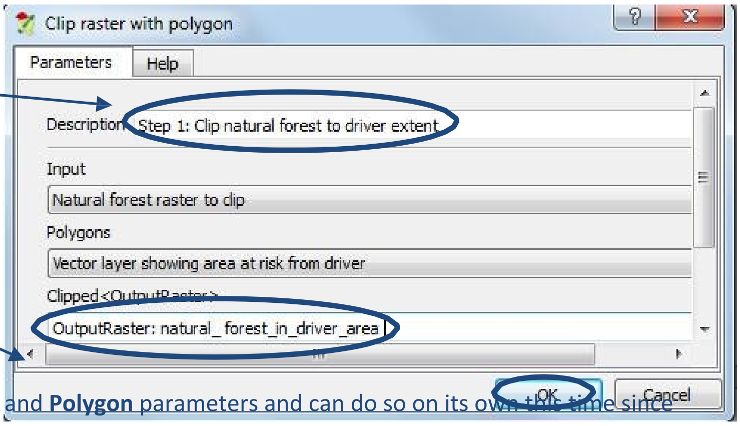

r. **QGIS** automatically retrieves the **Input** and **Polygon** parameters and can do so on its own this time since there is only one vector and one raster parameter in the model. **Click OK.**

s. The first step is now complete. Click the **Save button** to save the model.

t. Click the **Run Model** button to test the model as it is. We immediately find a problem with our planned processing flow—our natural forest layer is a vector dataset, and the model requires a **raster**. Therefore, we need to add a new step at the beginning of our model to convert the natural forest vector to a raster.

u. [illegible] **Modeler.**

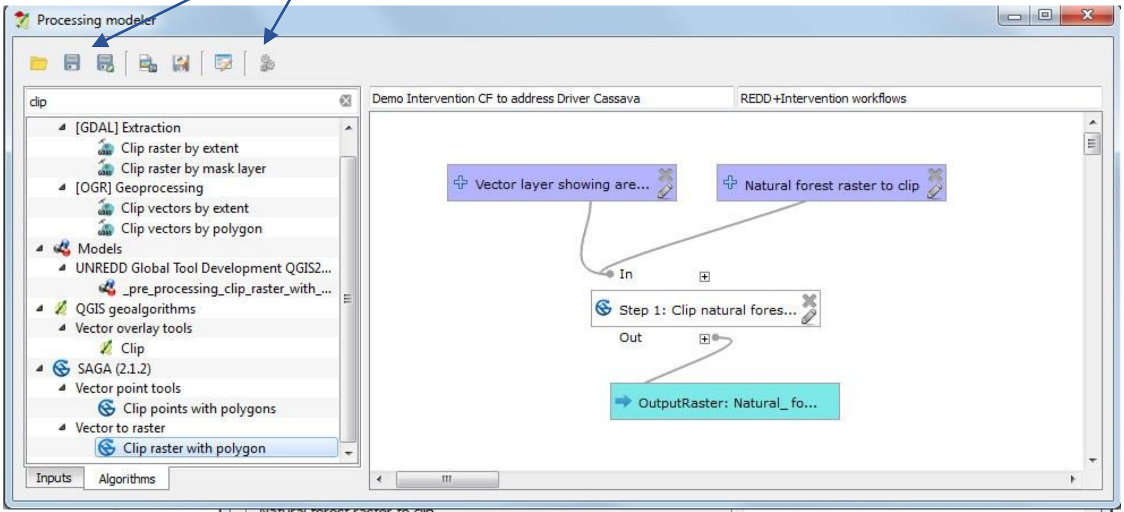

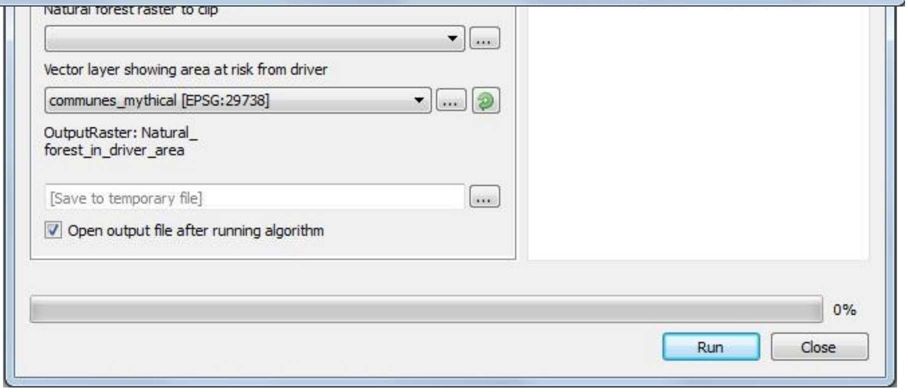

5.4.2. Modify the first step

a. We need to add a vector layer to the **Processing Model** canvas; this will enable conversion to raster. Click on **Inputs**, select **Vector layer**, and drag it to the model canvas.

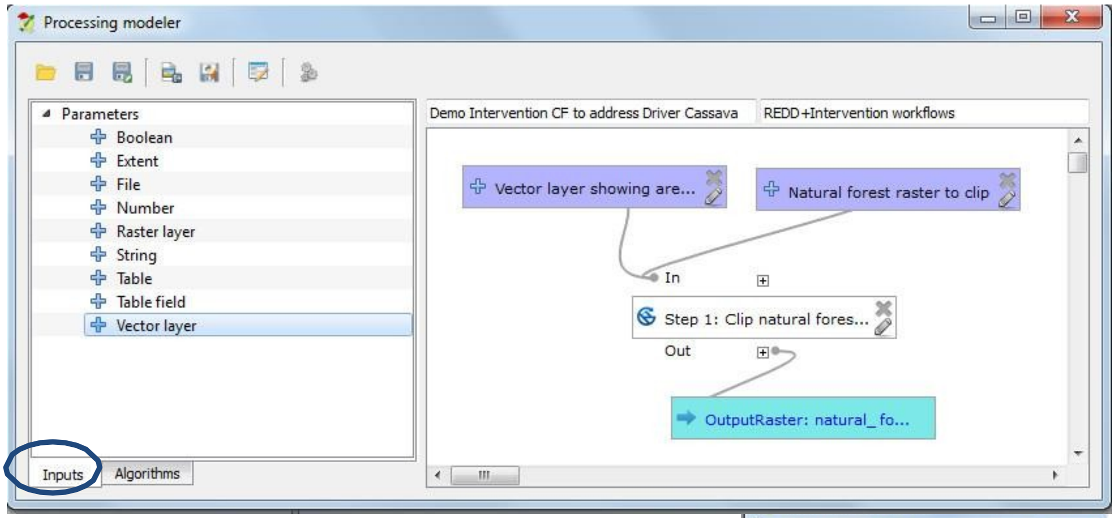

b.

c. Enter **natural forest vector** in the **name parameter box**.

d. Set the **shape type** to **Polygon.**

e. Click **OK.**

f. In the **Processing Modeler**, click on the **Algorithms tab** and search for **v.to.rast.**
Select **the v.to.rast.value tool** and drag it into the model canvas.

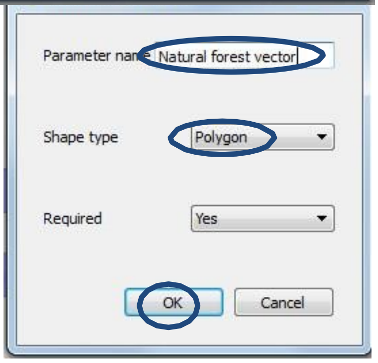

g. Change the **description** to **Step 1: Convert the natural forest vector to raster.**

h. For the **input vector layer,** select the **natural forest vector** parameter**.**

i. Set the **cell size** to the size you want to use for analysis, for example **1000.**

j. Specify a name for the output parameter value **Rasterized <OutputRaster> parameter value, e.g., OutputRaster: natural_forest.**

k. Click **OK.**

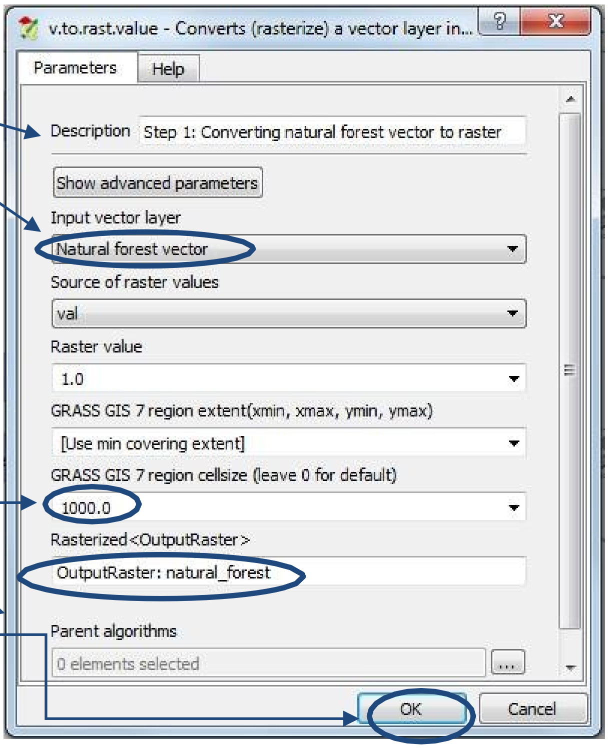

You can move the elements on the template canvas to see them clearly and logically (as in the excerpt below). Once you have moved them, you will see that there are now two separate parts that are not yet linked to each other. To link them, the output of the first step will replace the Natural Forest input raster of the second step. Note that you will also need to change the name of the second step to

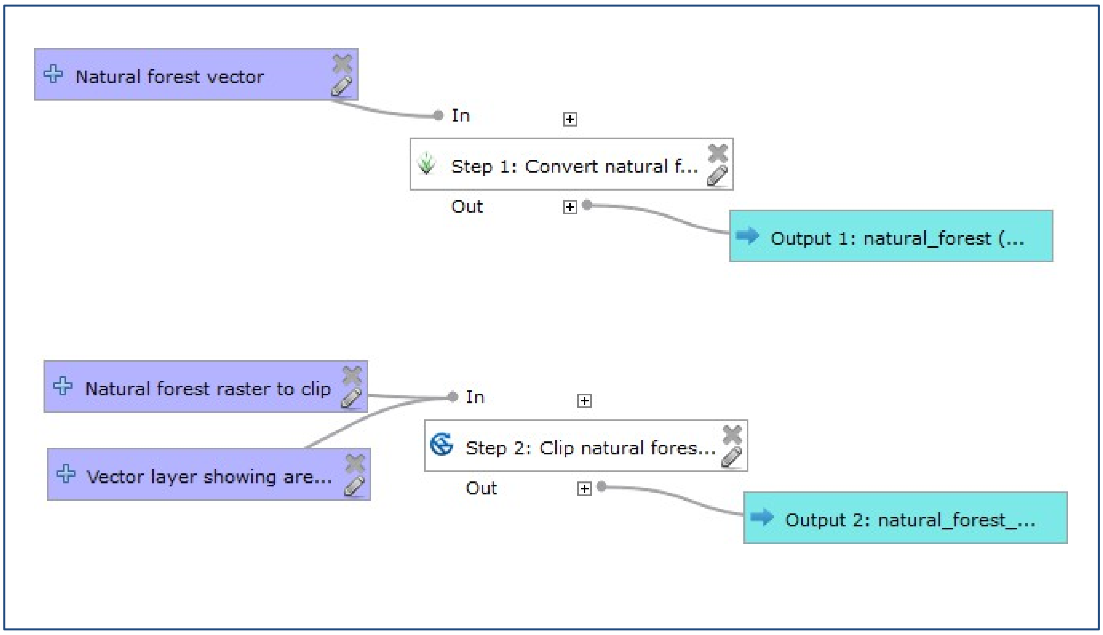

Step 2.

l. Right-click on the **Step 1: Cut the natural forest according to the extent of the factor** tool and click on **edit.**

m. Change the **description** to read **Step 2: Cut the natural forest according to the extent of the factor.**

n. Change the Input to "**Rasterized" created by the algorithm "Step 1: Convert the natural forest vector to raster"** to retrieve the output from the first step.

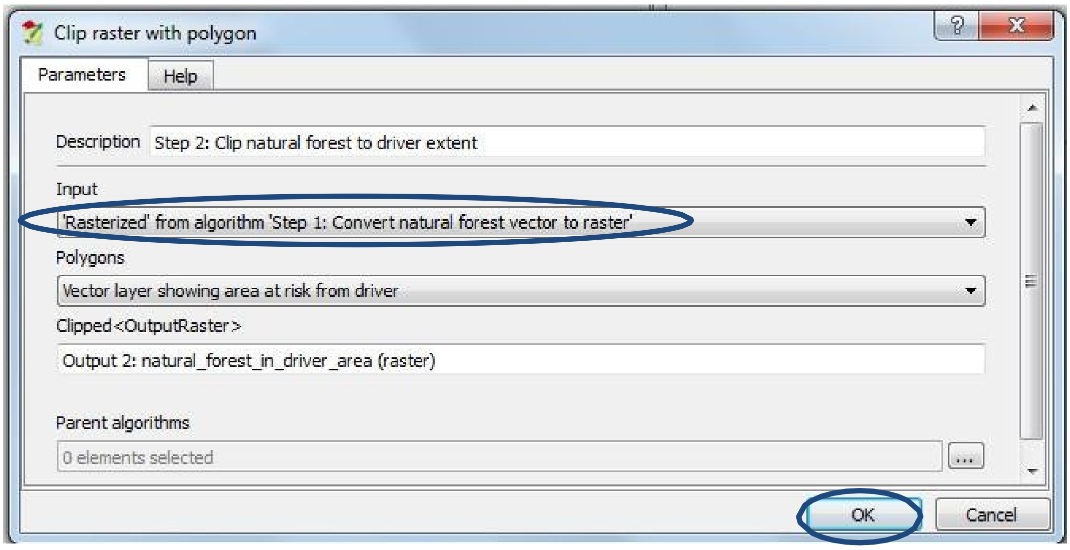

o. **Click OK.**

The old Natural Forest Raster has been disconnected from the rest of the model as it is no longer needed. The natural forest raster produced by step 1 is now the input for step 2.

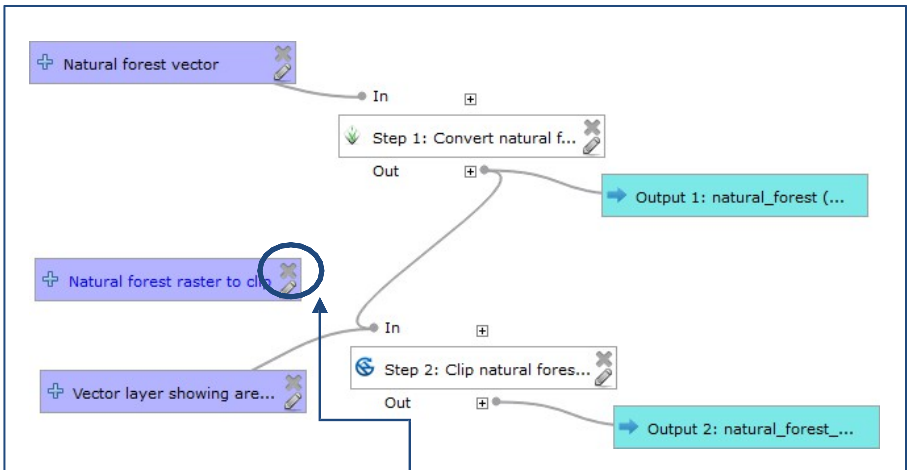

p. To remove the redundant parameter, click on the **X** in the upper right corner.

q. Click the **Save** button to save the model.

r. The tool should now look like the example below**.** To try out the template, click the **Run Template** button.

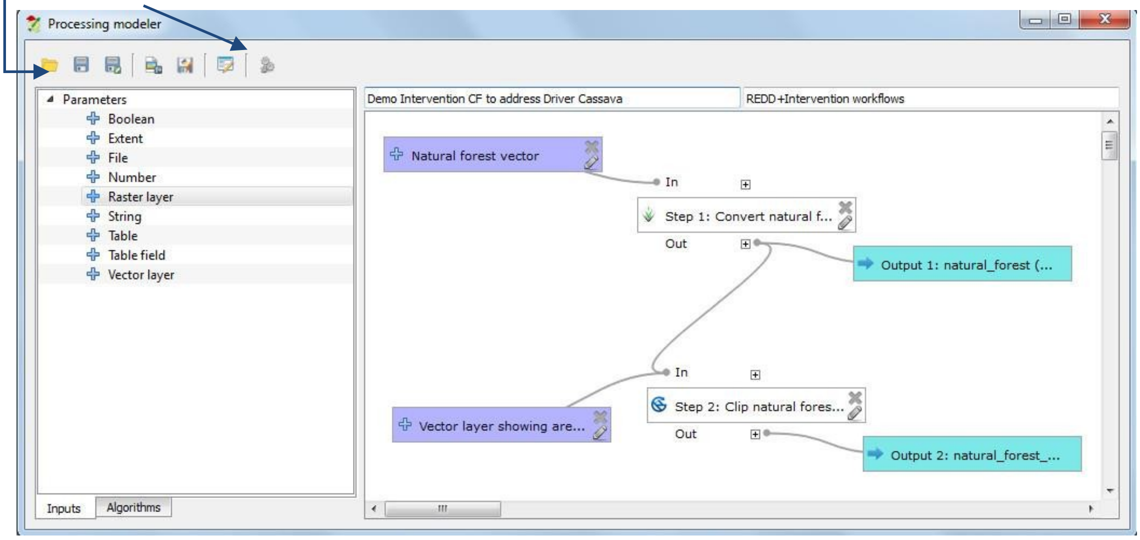

*Please note that testing the model in the **Process Modeler window** has sometimes been known to cause an error, but the model works fine if you do it from the **Process Toolbox**.*

*If your model opens, skip steps **s** and **t** below.*

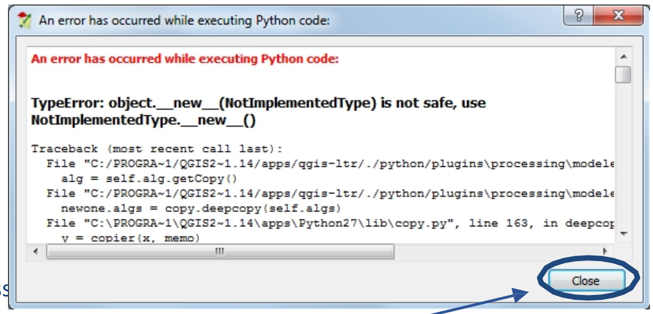

s. If an error occurs, click **Close** on the error mess[illegible] close the **Process Modeler** window.

t. Then, from the **Process Toolbox,** expand your **REDD+ intervention process flow** group and double-click on your **SC Intervention Demo** model **to counter the Cassava Factor.**

When it opens, your dialog box should look like this:

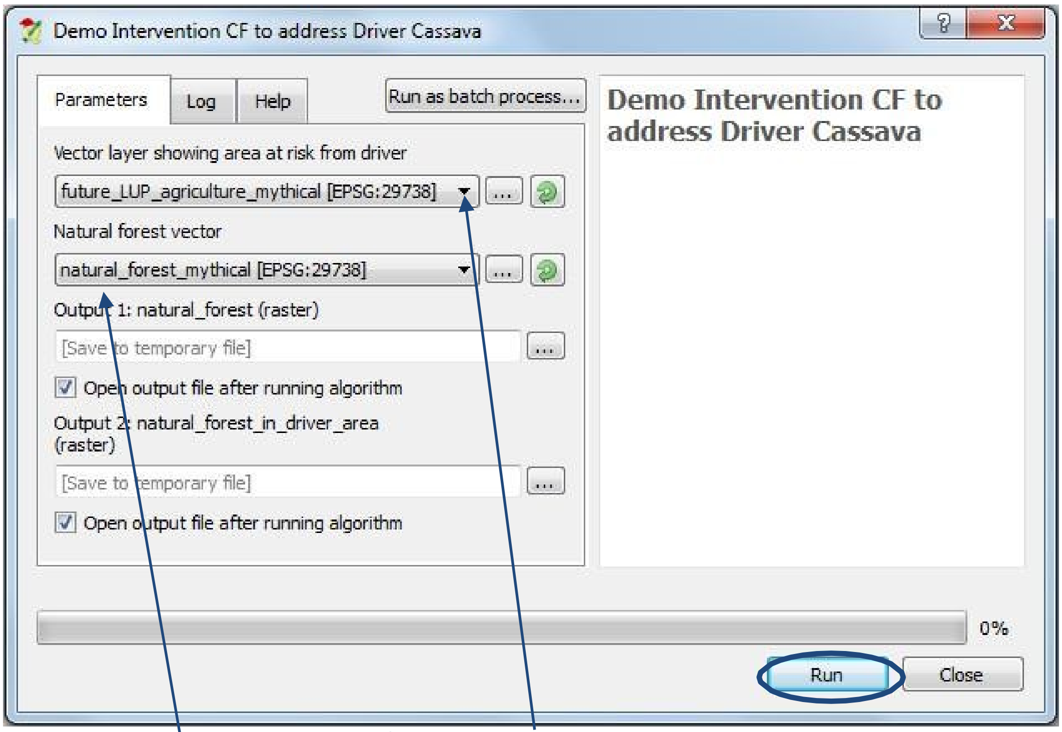

u. For our example model, we have chosen **future_LUP_agriculture_mythical** as **the vector layer showing the areas likely to be affected by the factor.**

v. Choose **natural_forest_mythical** as the **Natural Forest vector.**

w. While we are in the model testing phase, leave both model outputs as **[Save to temporary file].** Click **Run** to execute the model.

The model should run smoothly and close automatically when finished. You can see the progress. The excerpt in the example below shows that step 1 has been executed and that the model is correctly performing step 2. The descriptions you entered provide

valuable information about the process in progress.

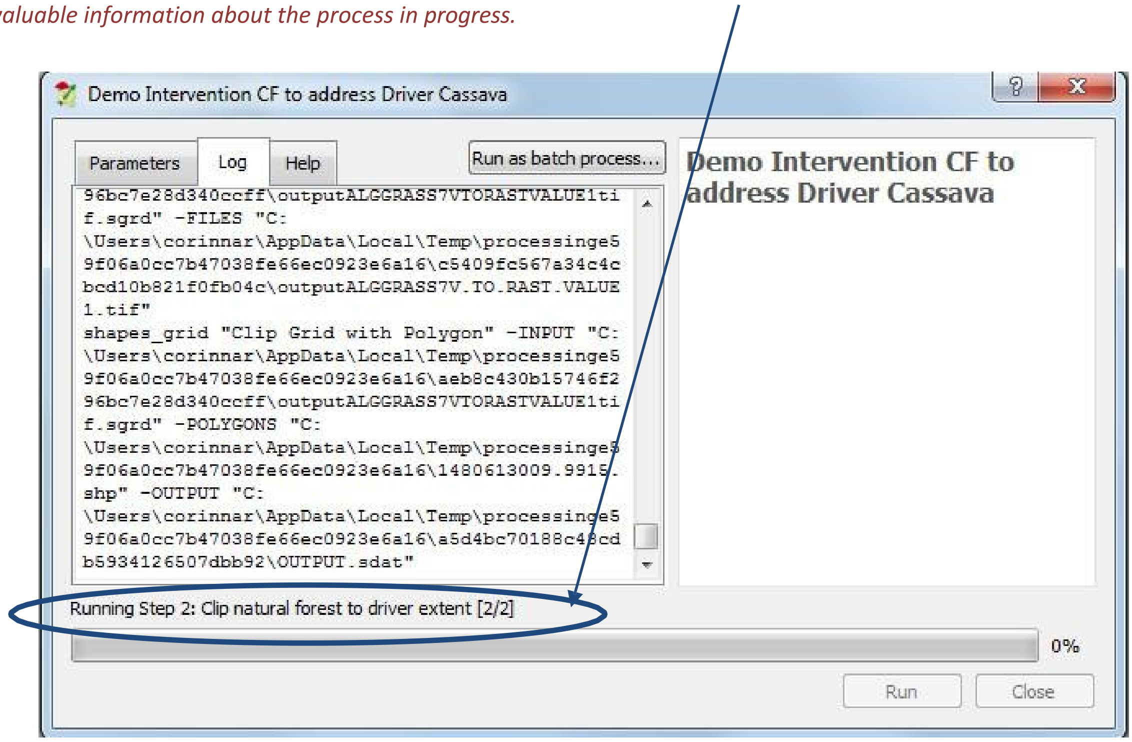

Once processing is complete, the outputs are added to the table of contents as shown below:

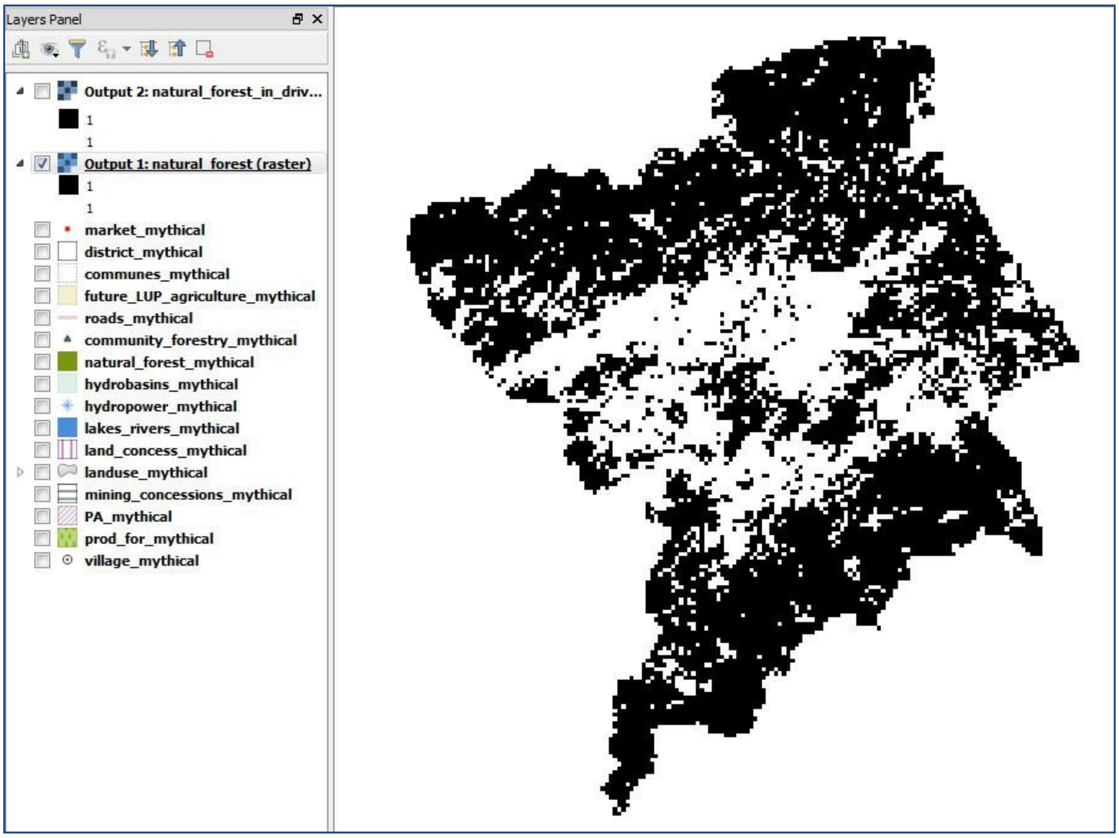

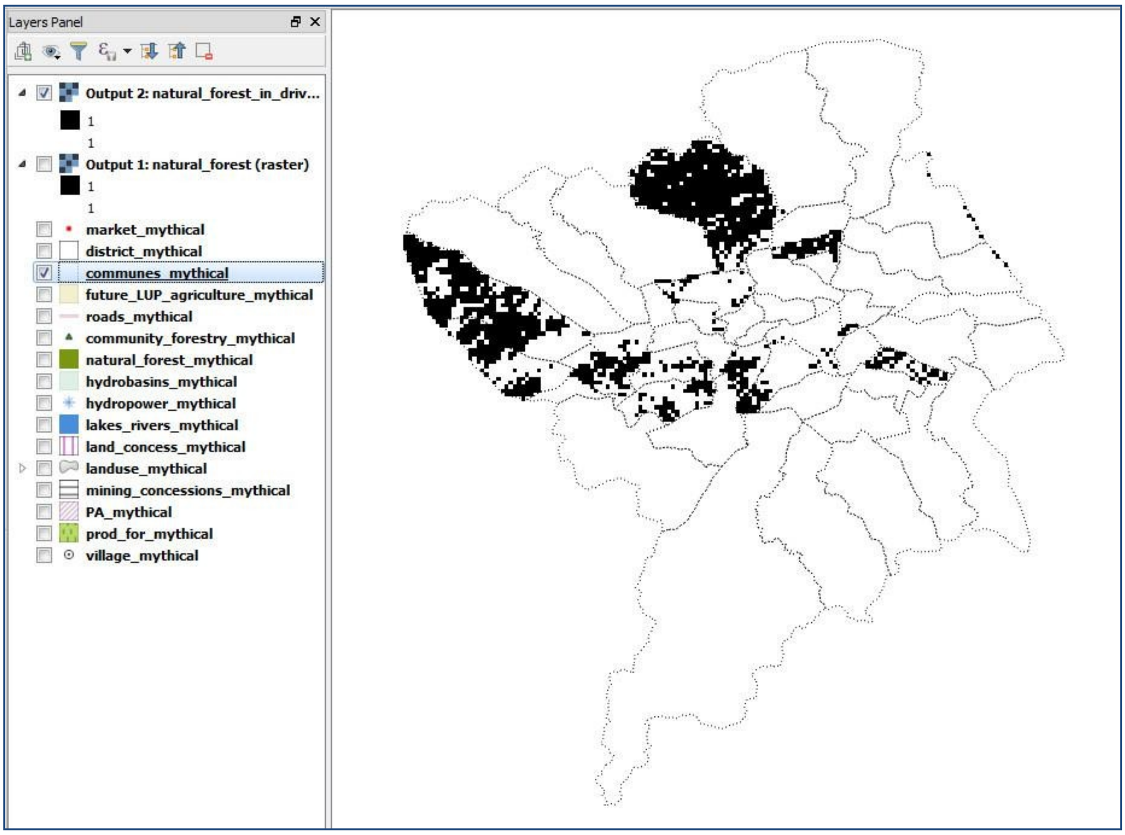

5.4.3. Documenting the first step

At this stage, it is useful to start adding metadata to the model to help you keep track of the different steps, their inputs, and their outputs. The metadata appears in the dialog tool to make the model easier to use.

a. If you have closed your model, right-click on your model in the **Processing Toolbox** and click **Edit Model** to reopen it in the **Graphical Model Editor.**

b. Click the **Model Help Editor** button in the **Processing Modeler** window.

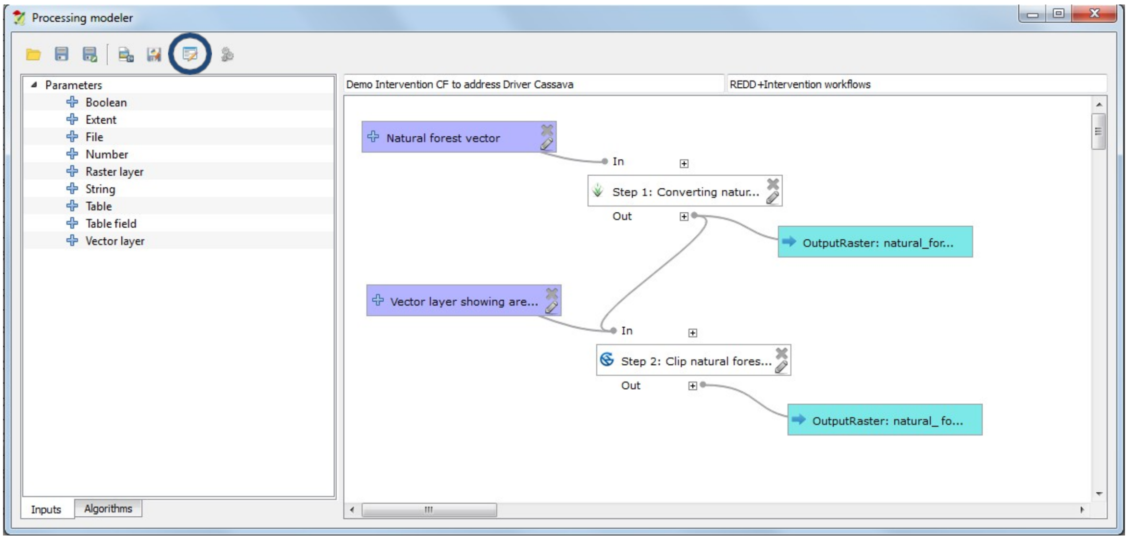

The help editor window appears. At the bottom left of the window, select the item to edit.

c. First, click **Algorithm description.**

d. In the **element description** window, describe the purpose of your model (the entire model, not just the purpose of the first steps you added). For our example, you could write:
An example of a processing flow for the development of a REDD+ intervention layer for sustainable community forestry to counter the factor "small-scale conversion of forest to cassava," based on based on the fact that forests that are va agricultural land.

e. **Click** on the arrow to expand the **input pa**

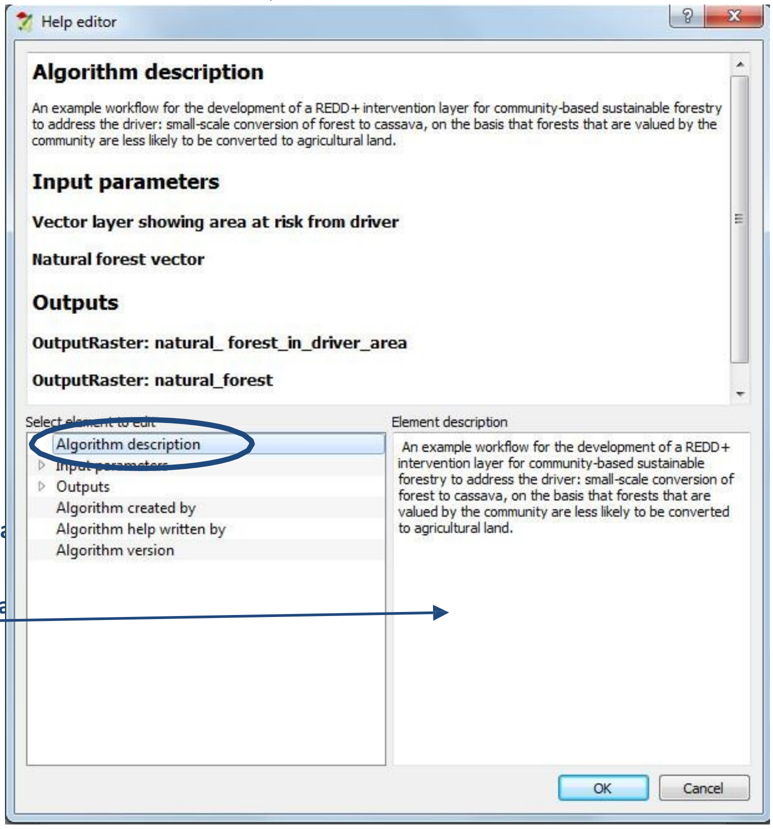

f. Click on **the vector layer showing the areas likely to be affected by the factor** and specify the following description elements: **Choose a vector polygon layer that shows only the areas likely to be affected by the factor (i.e., in this case, small-scale conversion from forest to cassava). You will need to have prepared this layer in advance to run the model; this processing flow does not create this layer for you.**

g. Click on **natural forest vector** and specify the following description element: **Choose a vector polygon layer that shows only these areas of natural forest. You will need to have prepared this layer in advance to run the model; this processing flow does not create this layer for you.**

h. Click the arrow to expand the **outputs.**

i. Click **Output 1: Natural_forest (raster)** and enter the following description: **Output created by the tool showing areas of natural forest in raster format (result of converting a vector dataset to a raster).**

j. Click **Output 2: Natural_forest_in_driver_area (raster)** and enter the following description: **Output created by the tool showing areas of natural forest within the driver area.**

k. **Click** on **Algorithm created by** and enter **your name,** for example**.**

l. **Click** on **Algorithm written by** and enter **your name,** for example**.**

m. **Click Algorithm version** and enter the version number, for example **1.0.**

n. Click **OK** to close the help.

o. Click the **Save** button in the **process modeler** window to save your model and the newly added help.

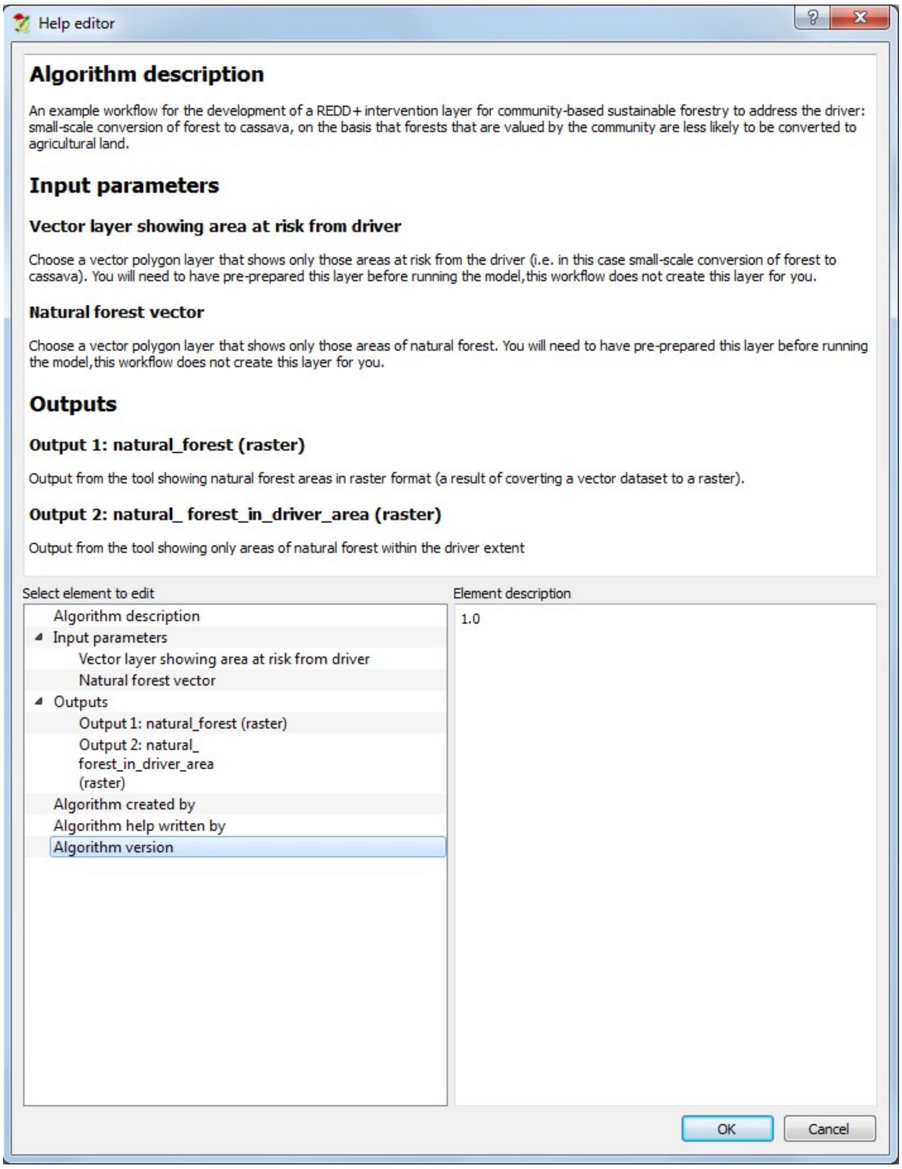

p. Click the **Run Model** button and verify that your **algorithm description** (the model objective - treatment flow) is displayed in the tool dialog box.

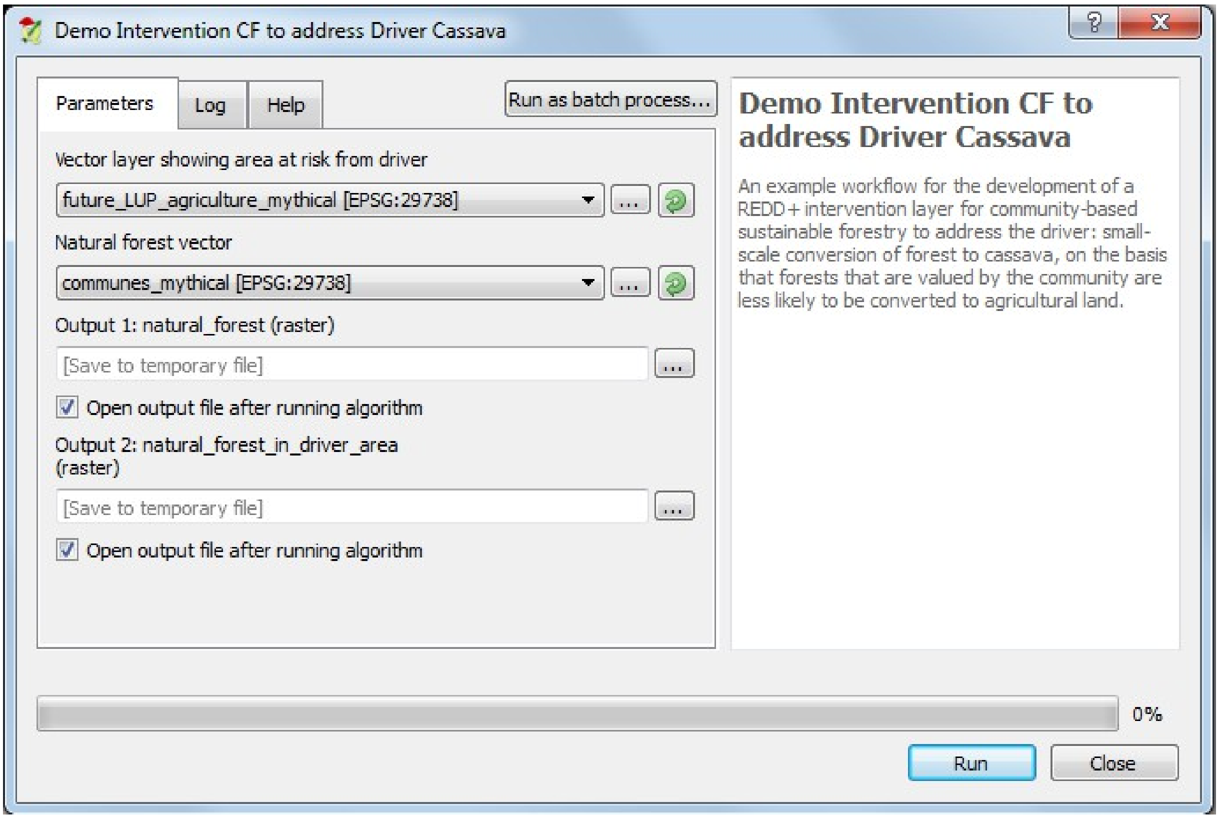

q. Click the **Help** tab and verify that your help text help text is displayed in the tool dialog box.

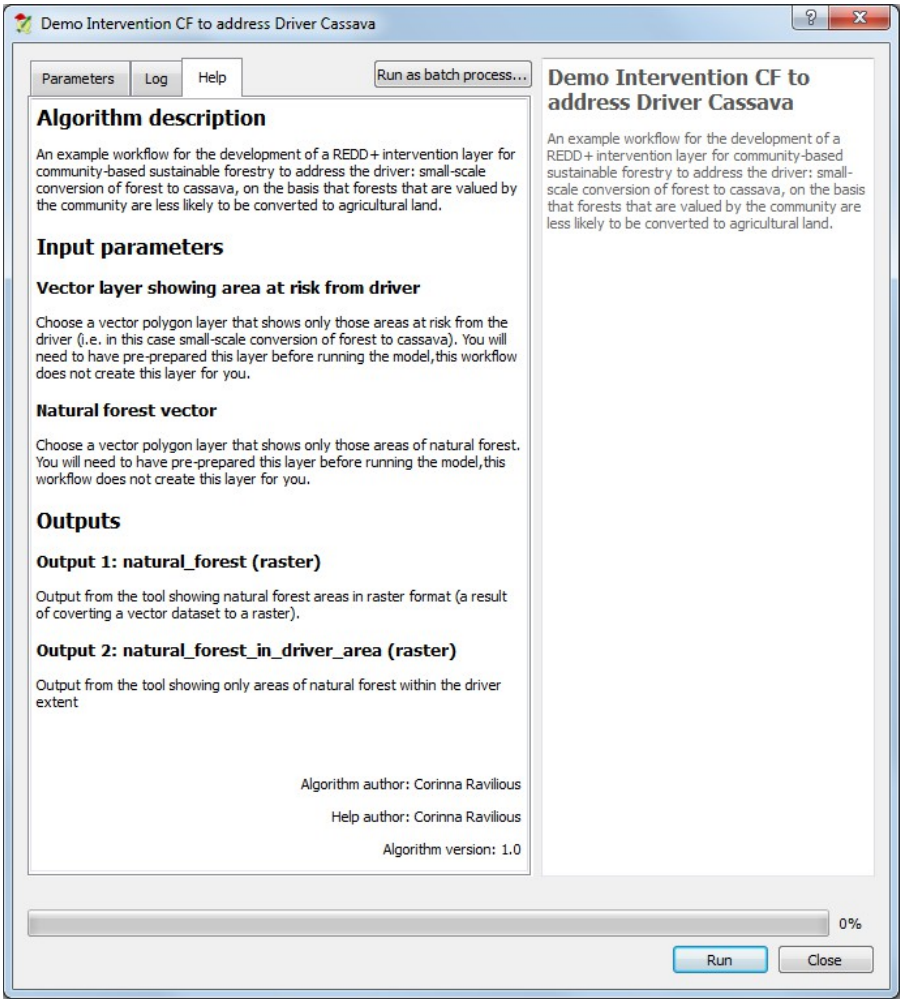

5.4.1. Add the remaining steps in the processing flow

5.4.1.1. Distance from Community Forestry sites

Look again at your processing flow to see the next step to add to **the Graphic Modeler**. There is a **Euclidean Distance** function specified on the left. This will generate an output distance raster from the Community Forestry sites raster.

In QGIS, **Euclidean distance** can be generated using several tools: **Proximity (raster distance) (GDAL), Proximity raster (SAGA),** or **r.grow.distance (GRASS)**. In this example, we have chosen to use **the r.grow.distance tool.**

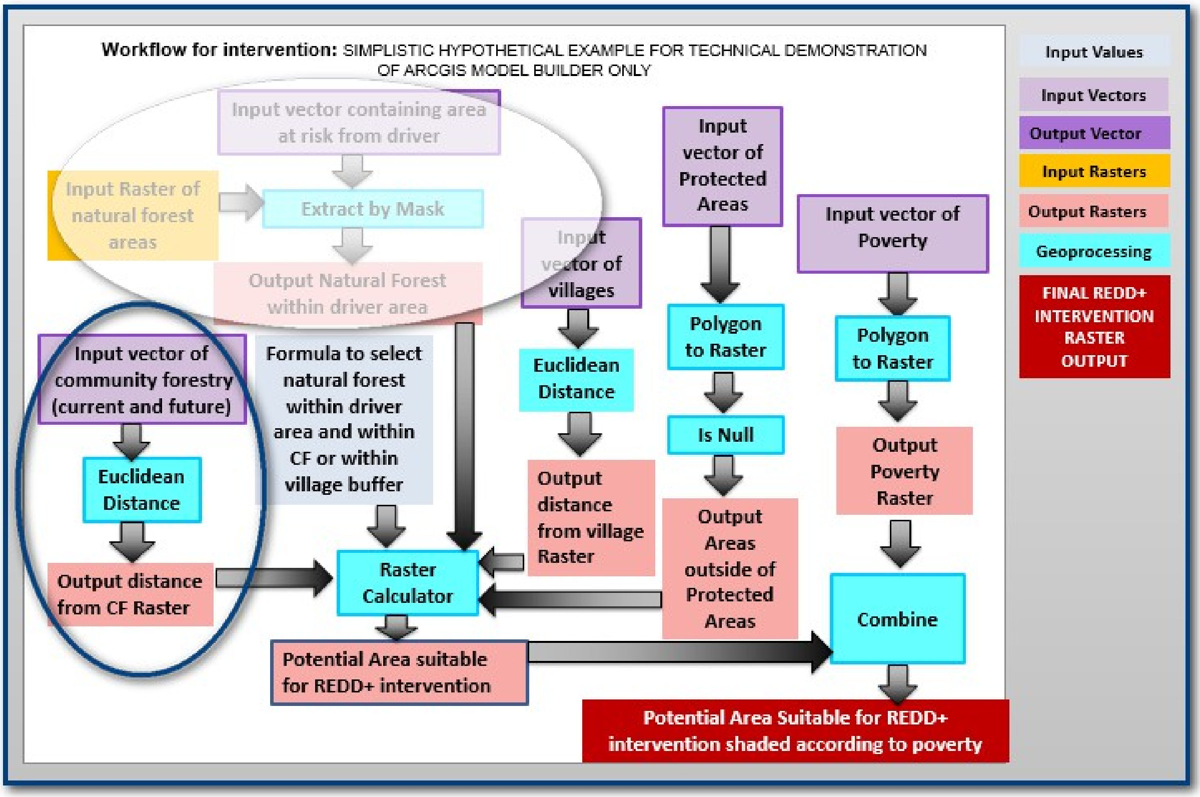

We want to generate an area representing the distance from community forestry areas, which will serve as the input dataset. Note, however, that the **r.grow.region** tool works with a raster layer; therefore, before adding the **r.grow.distance** tool, we must use the **r.to.raster.value** tool to convert the community forestry polygons to raster.

a. Click on **the Inputs tab** to add a **vector layer**.

b. Enter the **name of the Parameter,** e.g., **vector Community Forestry.**

c. Change the **shape type** t[illegible]

d. **Click OK.**

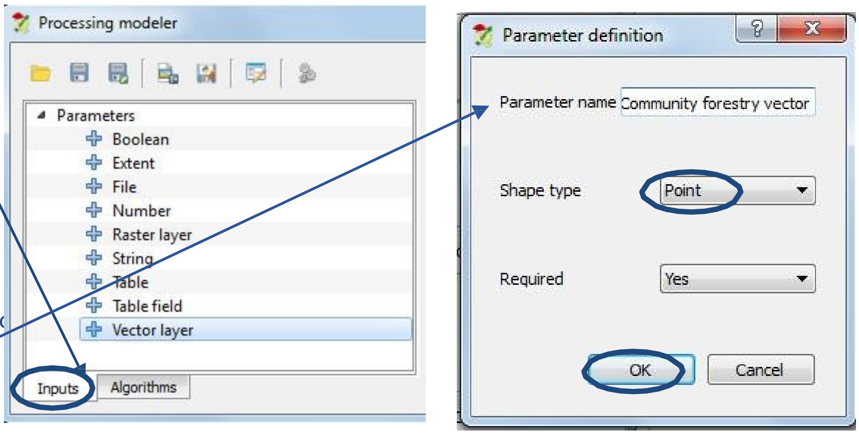

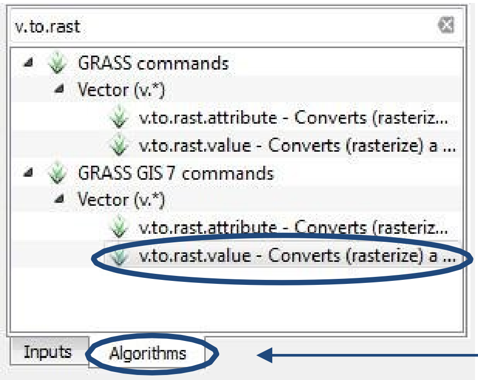

e. Click on the **Algorithms** tab and search for **v.to.rast.a**

f. Double-click to add the **v.rast.value** tool.

g. Change the description in **Step 3: Convert the community forestry vector to a raster**

h. Select the **Community Forestry vector** vector as **the Input vector layer.**

i. Set the **cell size**, for example to **1000.**

j. Click **OK to add the tool.**

k. Click on the Algorithms tab and search for r.grpw.

l. Double-click to add the tool r.grow.distance.

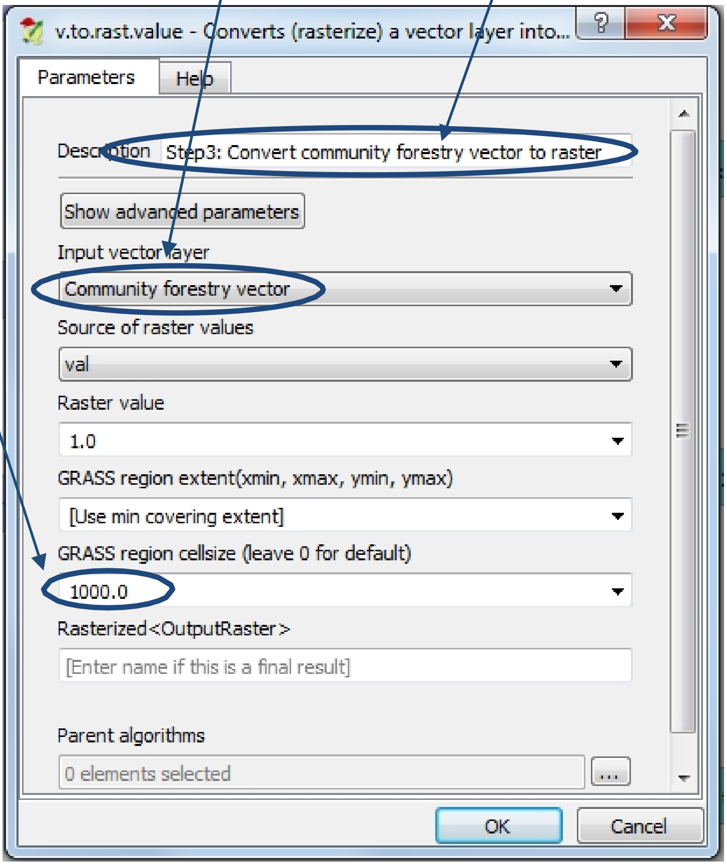

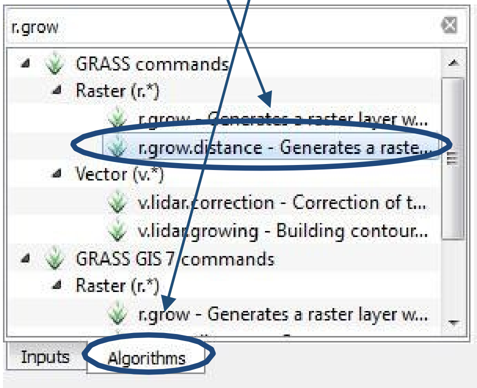

m. Change the description to **Step 4: Create a surface raster linked to the distance to community forestry.**

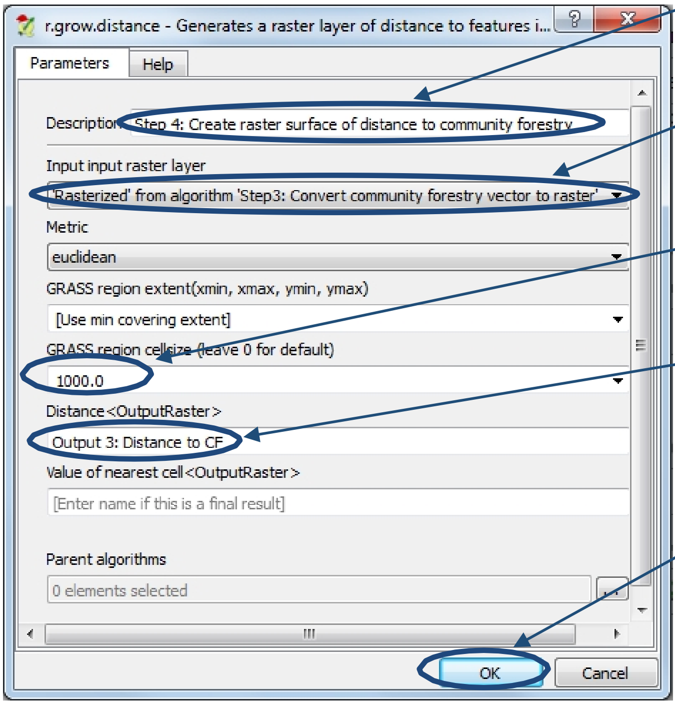

n. Set the Input raster **layer** to **"Rasterized" created by the algorithm "Step 3: Convert the community forestry vector to a raster."**

o. Set the **cell size**, for example to **1000**

p. Give the **Distance<Output>** parameter a name, for example. **Output 3: Distance to CS.**

q. Click **OK to add the tool.**

r. Click the **Save** button to save the template.

s. Click the **Template Help Editor** button to add help documentation to the new steps in the template.

t. Expand the **input settings**, click on **community forestry**, and enter the following description: **Choose a vector point layer that shows only community forestry sites. You will need to have prepared this layer in advance to run the model; this processing flow does not create this layer for you.**

u. Expand the **outputs**, click **Output 3: Distance to CS**, and enter the following description: **Output created from the tool showing a raster surface of distances from community forestry sites.**

v. Click **OK,** then click the **Save** button to save the model and model help.

w. Click **Run Template** to test it.

x. Once processing is complete, the outputs are added to the table of contents.

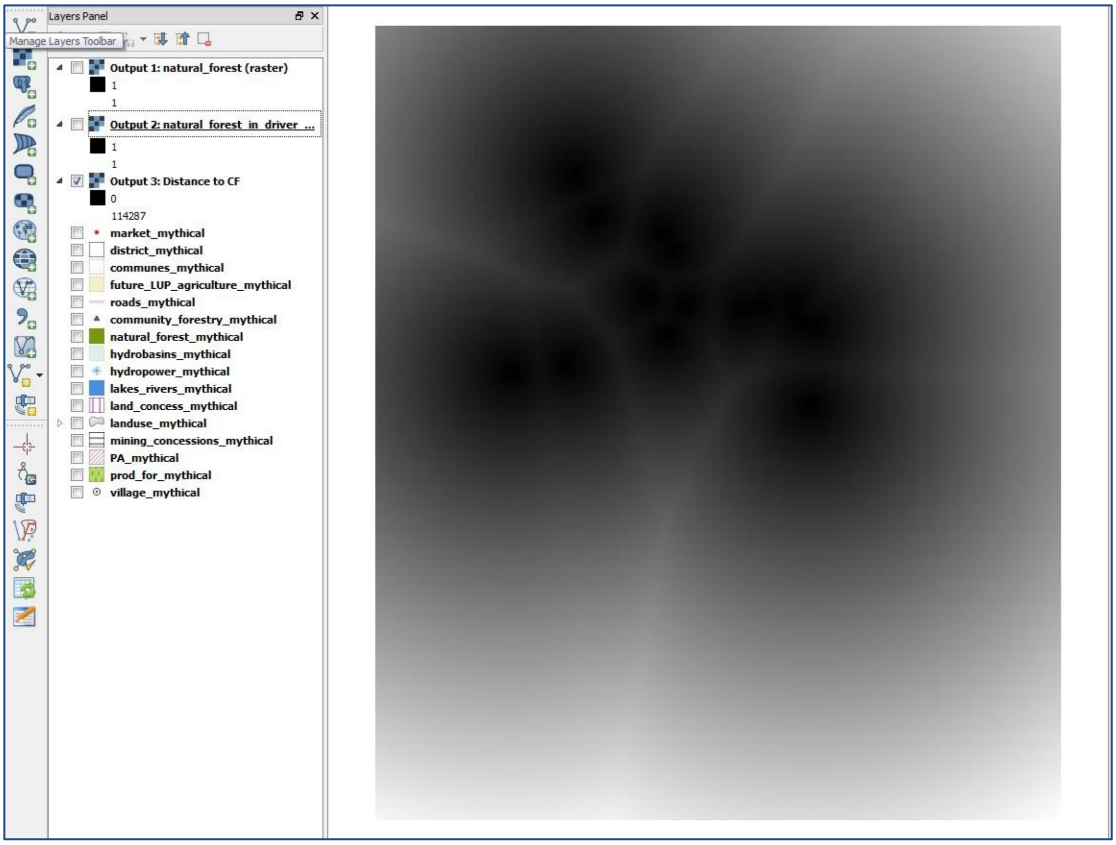

While a workflow is necessary to guide us through the analysis, it is not uncommon to find that either a chosen tool is inappropriate or that a step can be performed more efficiently or more simply. Also, in QGIS there are often many tools available to perform the same or similar functions, so if one tool does not work, try another.

5.4.1.2. Distance to villages

Look at your processing flow again to check the next step to add to the pattern generator. There is another **Euclidean Distance tool** on the right. This will generate a raster output related to the distance from villages.

Repeat **steps a-x** from **section 5.4.1.1**, but this time to generate the distance to villages. The output will be

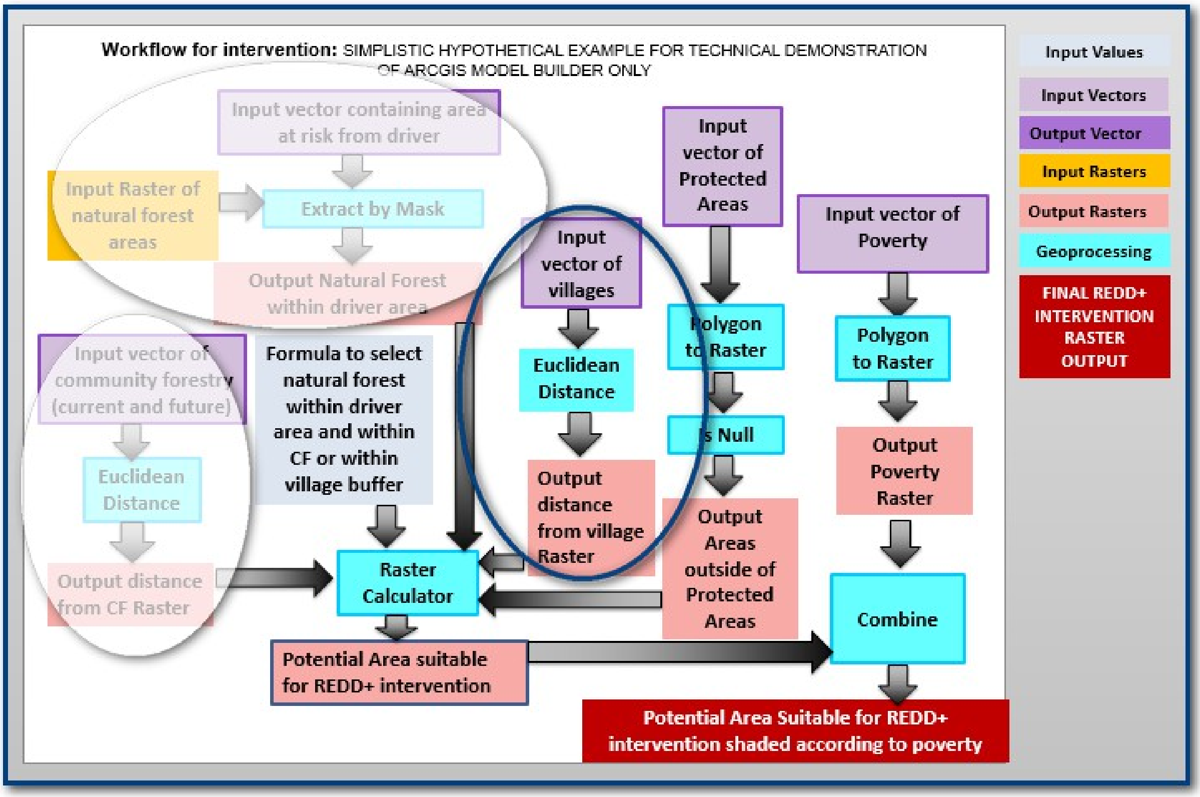

added to the table of contents as shown below.

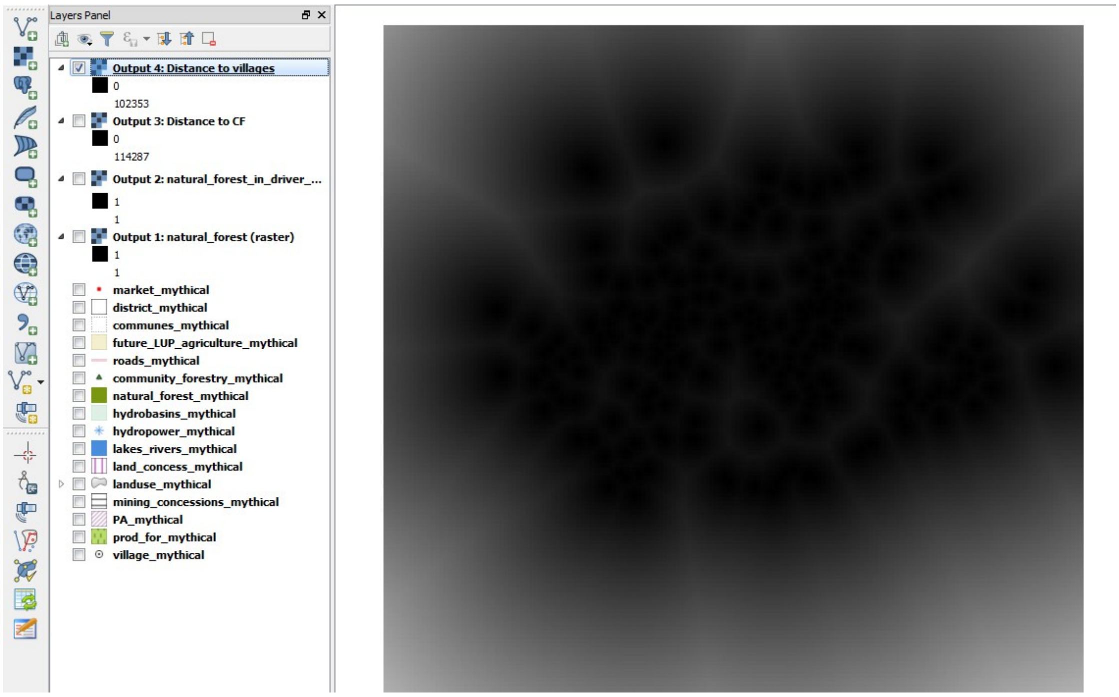

The treatment flow should now resemble the excerpt below. Note that steps 1 and 2 are linked, steps 3 and 4 are linked, and steps 5 and 6 are also linked, but currently the three pairs are not joined.

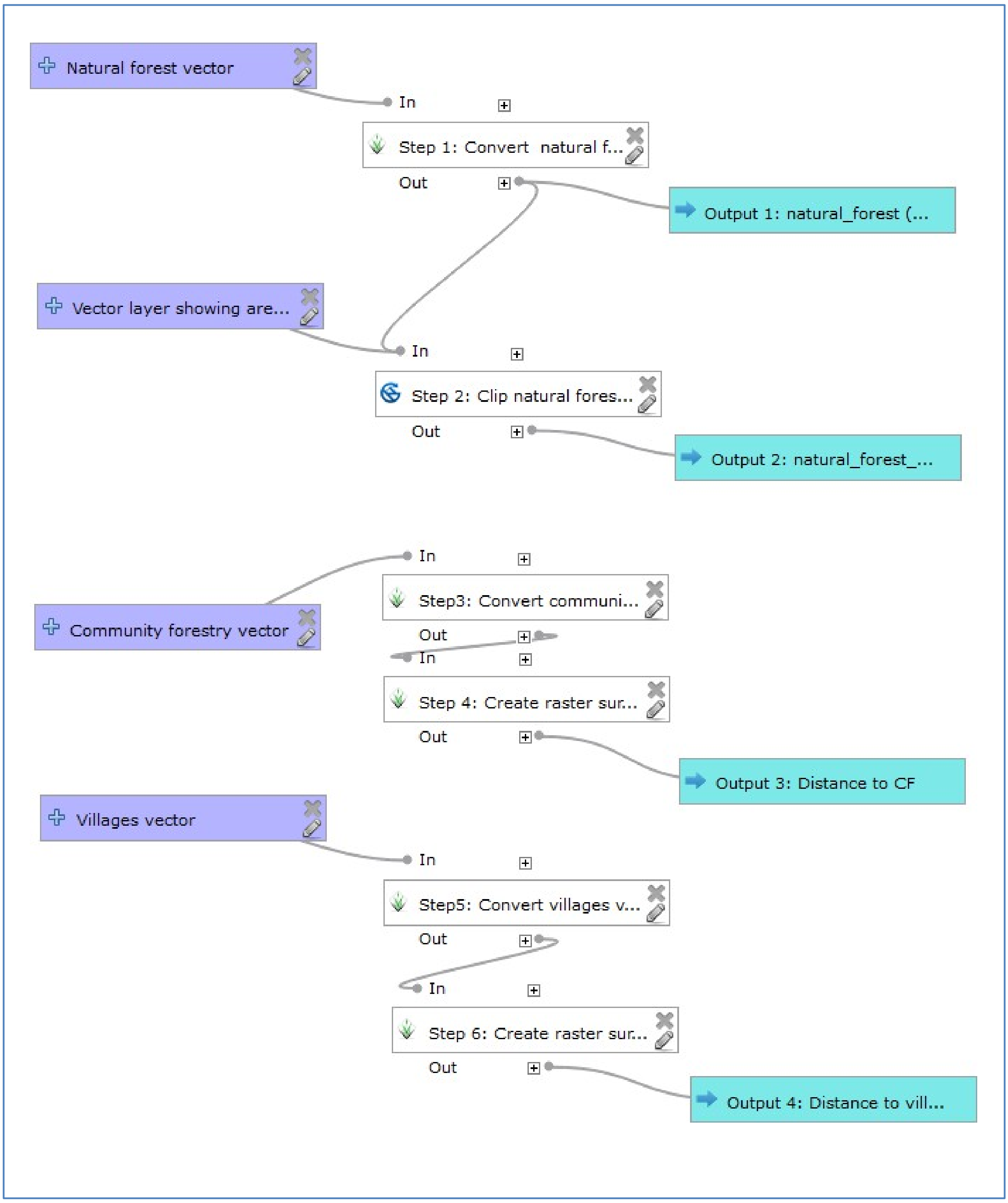

The order in which these three groups are executed does not matter, but they must be executed before the next step. This step requires the outputs of these three groups of steps, which will therefore be linked together in the model.

5.4.1.3. Select the natural forest within the factor range and within specific distances

Look at your processing flow again to check the next step to add to the pattern generator. In the processing flowchart, there is a **processing tool called Rastor Calculator**, which will use all the outputs from the **mask extraction** and **Euclidean distance functions** to select forest areas that are both within the factor extent and within the specified distance from community forestry sites and villages.

In QGIS, there are three **Raster Calculator** options available for use in the graphical modeler: **Raster Calculator (GDAL)**, **r.mapcalculator (GRASS),** and **Raster calculator (SAGA)**.

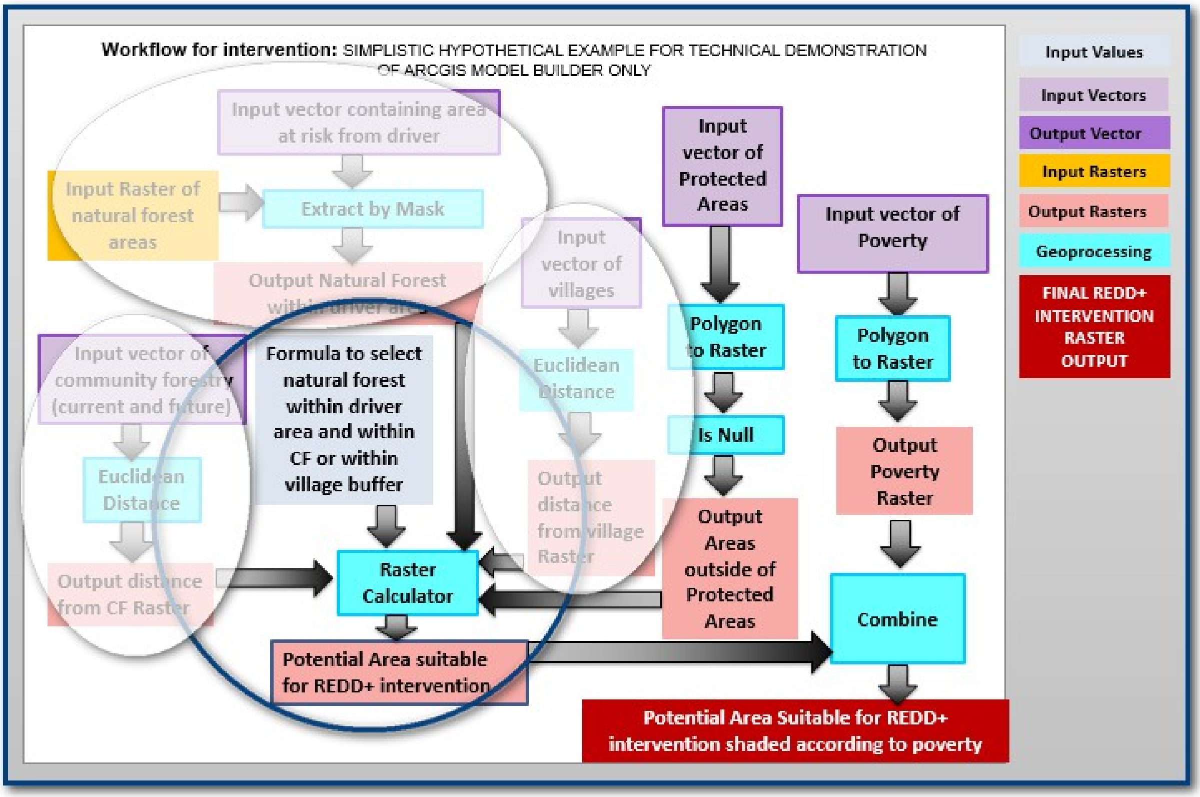

A raster calculator expression is used to select the necessary areas. The syntax varies slightly depending on the calculation tool you choose. (See Appendix 1, section A.3.5 for help with the syntax of **raster calculation tools**). In our example, we will use **the (GRASS) r.mapcalculator tool.**

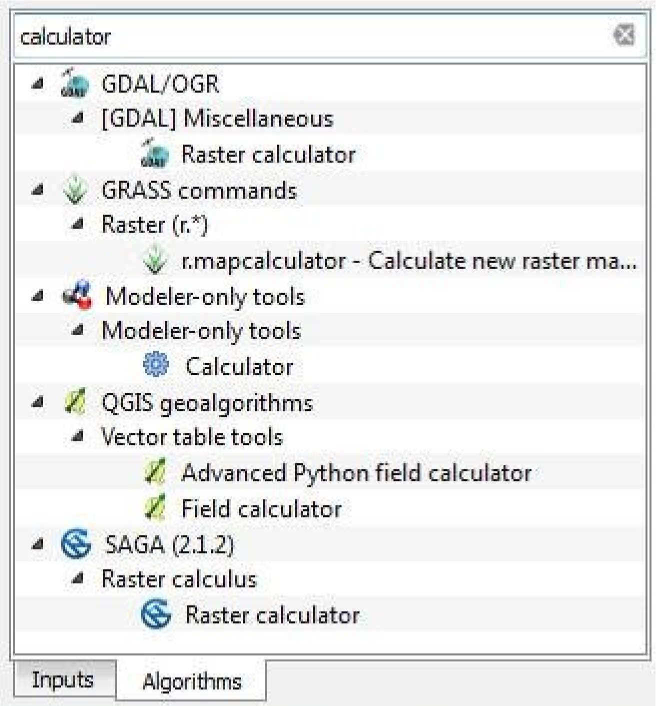

a. Click on the **Algorithms** tab and search for **calculator.**

b. Double-click on **r.mapcalculator** to add the tool to the template canvas.

We will use the map calculator to select forests within the factor extent that are either 10 km or less from existing or proposed community forestry sites or 3 km or less from villages.

c. Change the **description** in **Step 7: Select natural forest within the factor extent and within 10 km or less of a CS site or 3 km or less of villages.**

d. **In Raster Layer A,** select the result of step 2**, "Clipped from algorithm," step 2: Clipping natural forest according to factor extent."**

e. **In** Raster Layer B, select the result of step 4**, "Clipped from algorithm," step 4: Create a raster surface linked to the distance to community forestry."**

f. In Raster Layer C, select the result of step 6**, "Clipped from algorithm." of the algorithm) "Step 6: Create a surface raster linked to the distance to villages."**

g. In the formula window, enter the expression below:

(((A==1)&&(B<=10000))||((A==1)&&(C<=3000)))

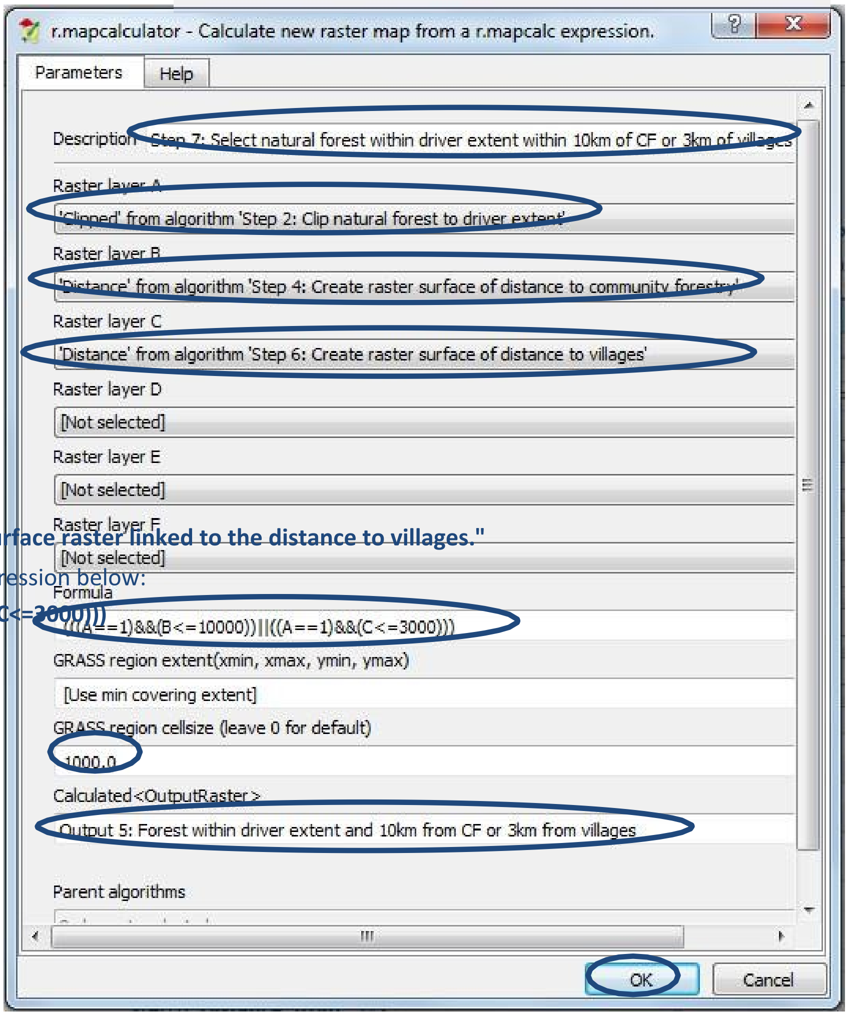

This means: "**select** from raster A (i.e., the forest within the factor's extent) the cells that are equal to 1 and the cells from raster B that are 10 km or less away (i.e., the distance to existing or proposed community forestry sites) **OR** the cells in raster A (i.e., the forest within the factor extent) that are valued at 1 and the cells in raster C are 3 km or less away (i.e., distance to villages). The output corresponds to **forest areas within a 10 km band around proposed and existing community forestry sites, or within a 3 km band around villages.**

h. For **calculated<OutputRaster> (<Outputraster> calculated),** specify **Output 5: Natural forest within the factor extent and within the 10 km band around CS sites or within the 3 km band around villages.**

Once you have added the new step, don't forget to click on the ***model help editor*** *button to add your description for this new step. Don't forget to save your model.*

The three groups of steps are now linked. The model should now look similar to the illustration below:

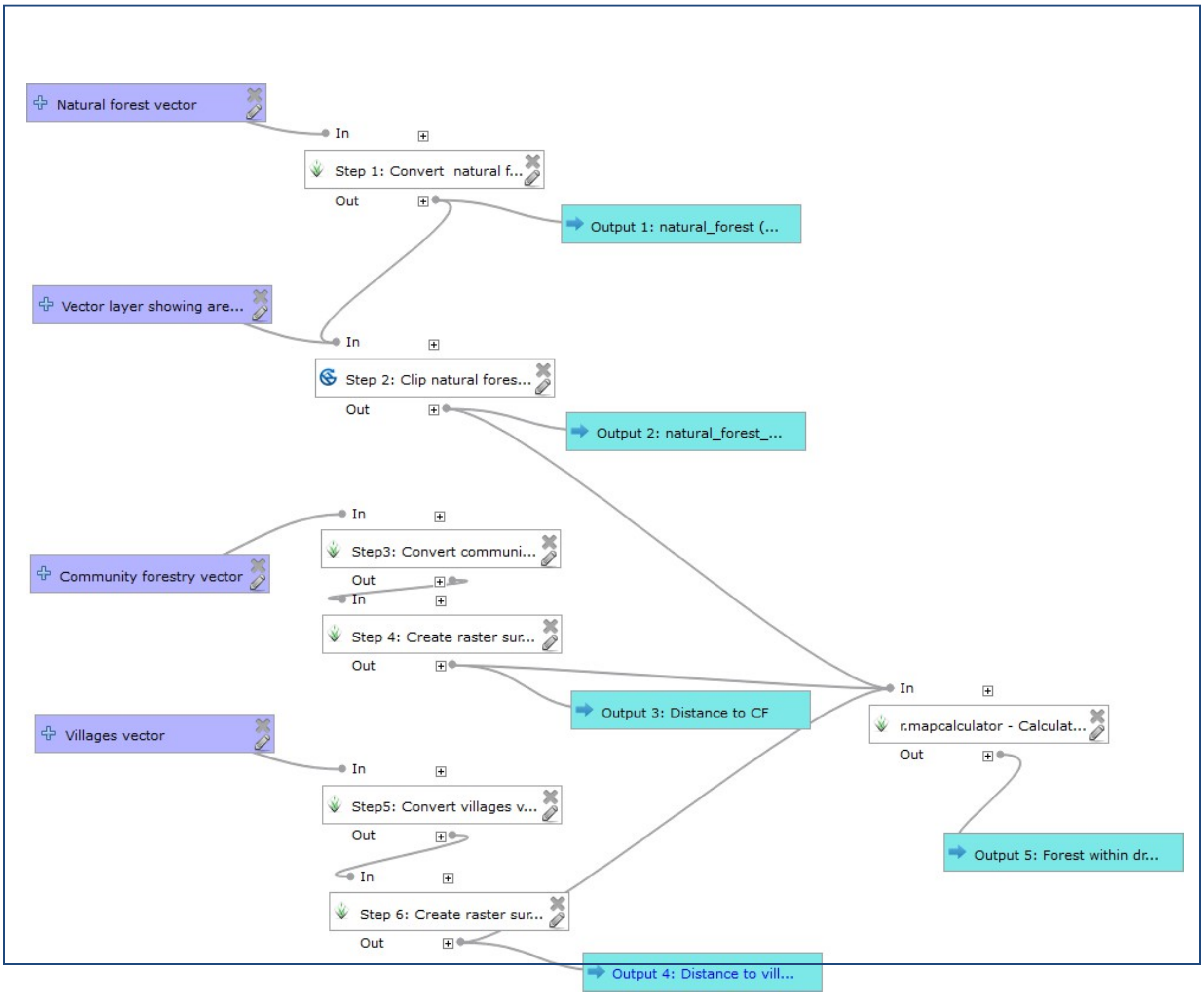

5.4.1.4. Exclude strictly protected areas

Check the model against the list of datasets we identified at the beginning, as well as the processing flow.

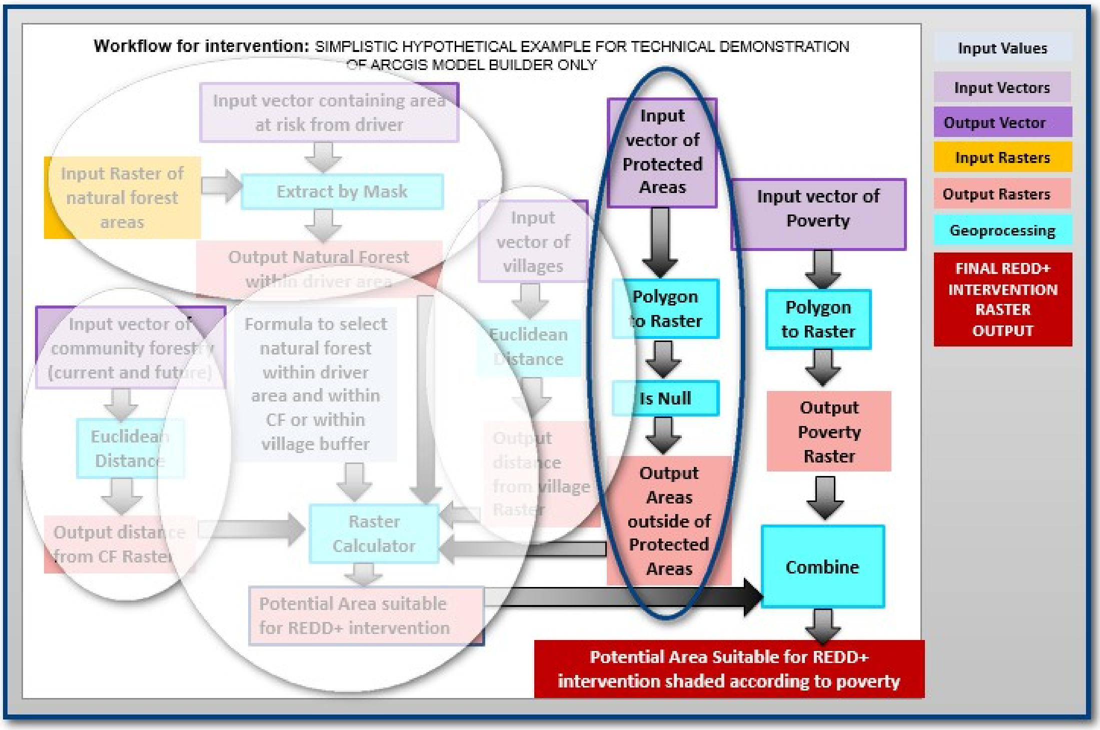

As it stands, the model allows you to complete the first four parts of the processing flow, with two still remaining. The next step to add to your model is to "**exclude strictly protected areas**."

a. Protected areas are a vector layer, so you need to add a **vector layer** and a **v.to.rast.value** to **the processing modeler**.
b. Change the description to **Step 8: Convert the protected areas vector to raster.**
c. **Select** the **Protected Areas** parameter as the **Input Vector** layer.
d. Set the **cell size**, for example to **1000.**
e. **Click OK.**

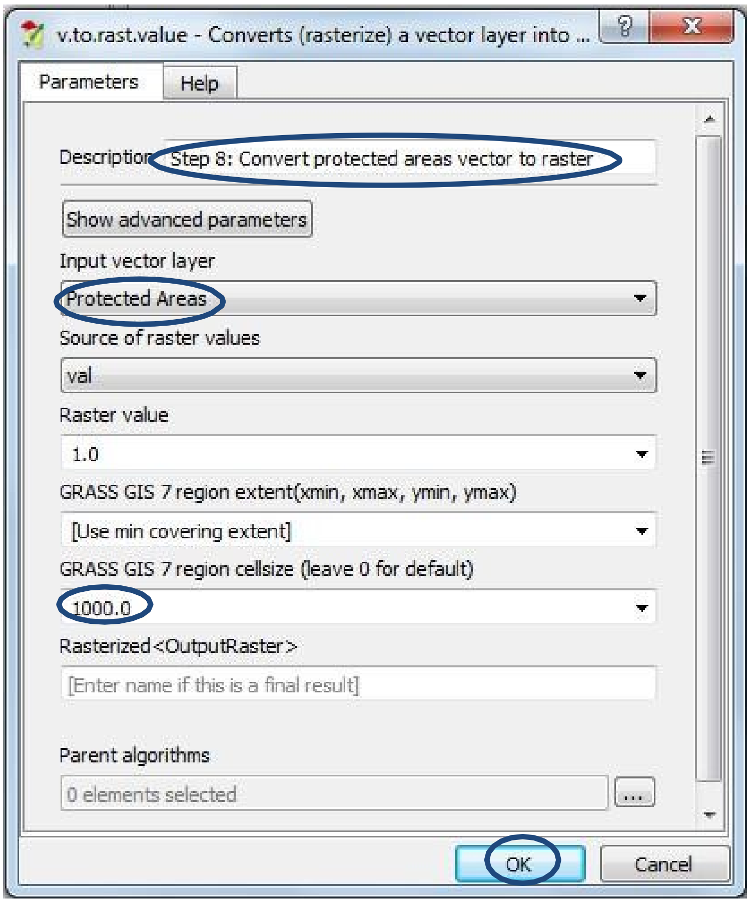

Once you have added the new step, don't forget to click on the ***template help editor*** *button to add your description for this new step. Don't forget to save your template.*

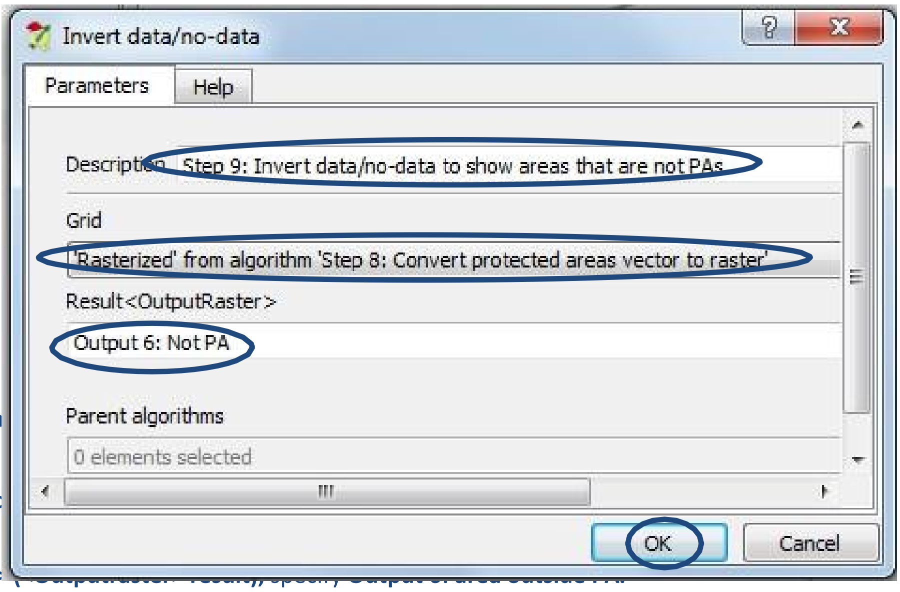

- **f.** To select areas that are not strictly protected, add a **(SAGA) Invert data/nodata** tool in the **processing modeler.**
- **g.** Change the description to **Step 9: Invert data/no data to show areas that are n**[illegible]
- **h.** Select the **"Rasterized" c**[illegible] for the Grid.
- **i.** In **Result<OutputRaster>** [illegible]
- **j.** Click **OK.**

We will then use output 6 in another **r.mapcalculator** expression to select natural forest areas within the extent of the factor and within the 10 km band around SC sites and the 3 km band around villages.

- **k.** Change the description in **Step 10: Select the natural forest within the factor range and within 10 km or less of an SC site or 3 km or less of villages and which is not an AP.**
- **l.** Select **"Calculated" created by the algorithm "Step 7: Select natural forest within the factor extent and within 10 km of an SC site or 3 km of villages"** for raster A.
- **m.** Select the **"Result" created by the algorithm "Step 9: Invert data/no data to show areas that are not PAs"** for raster B.
- **n.** In the formula window, enter **(A==1)&&(B==1)**

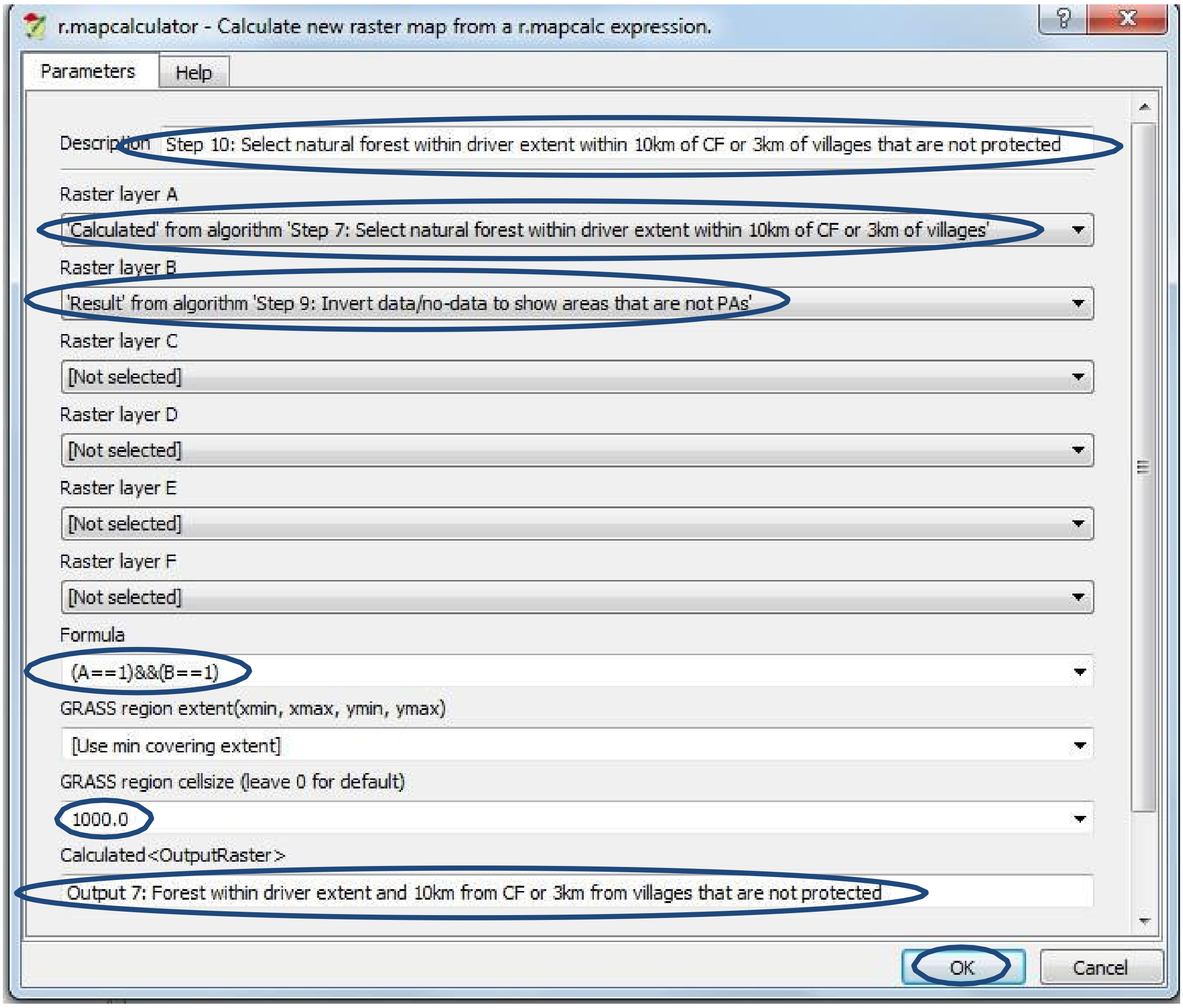

- **o.** Set the **cell size**, for example to **1000.**
- **p.** For **Calculated<OutputRaster> (<Outputraster> calculated),** enter **Output 7: Forest within 10 km or less of an SC site or 3 km or less of villages and which is not an AP.**
- **q.** **Click OK.**

*Once you have added the new step, don't forget to click on the **model help editor** button to add your description for this new step. Don't forget to save your model.*

The legend should look like this:

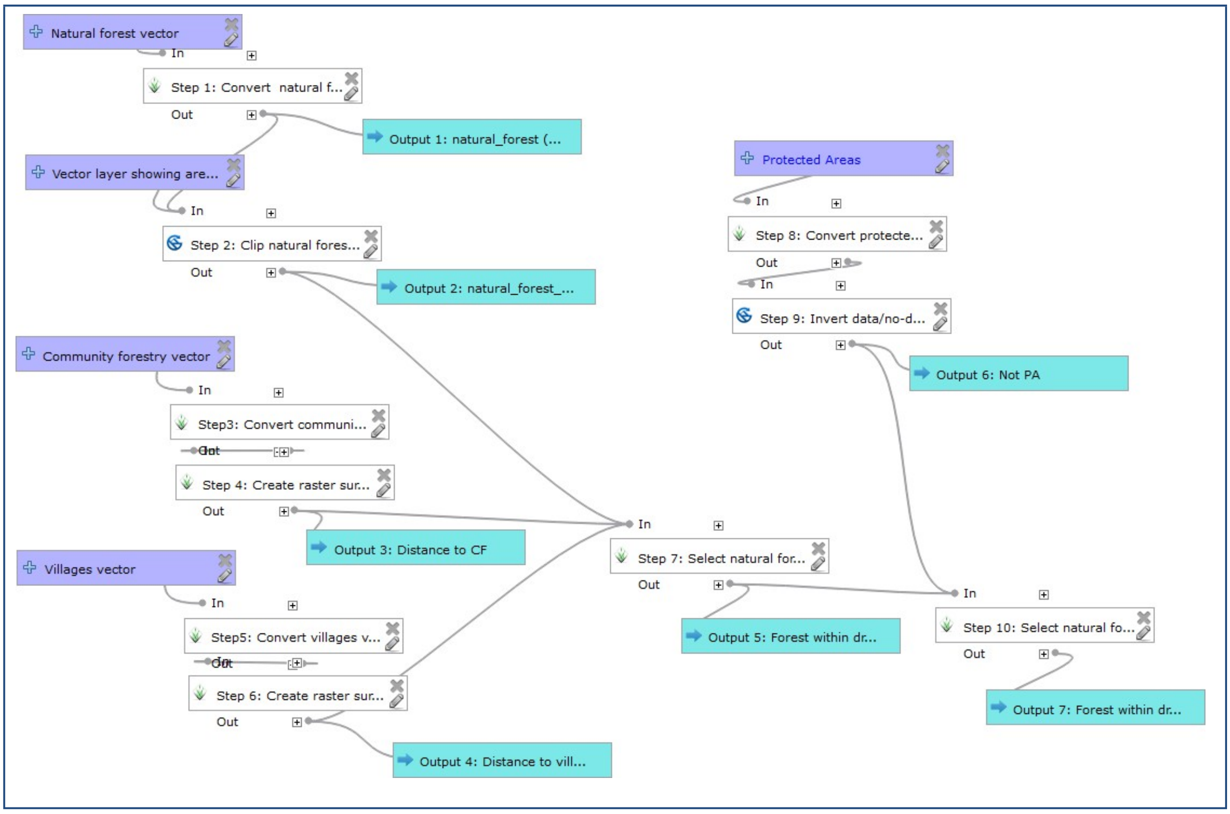

5.4.1.5. Identify priority areas based on poverty

There is one final step in the original processing flow: classifying the selected areas so that they can be prioritized based on poverty. This will clarify one of the additional potential benefits of REDD+:

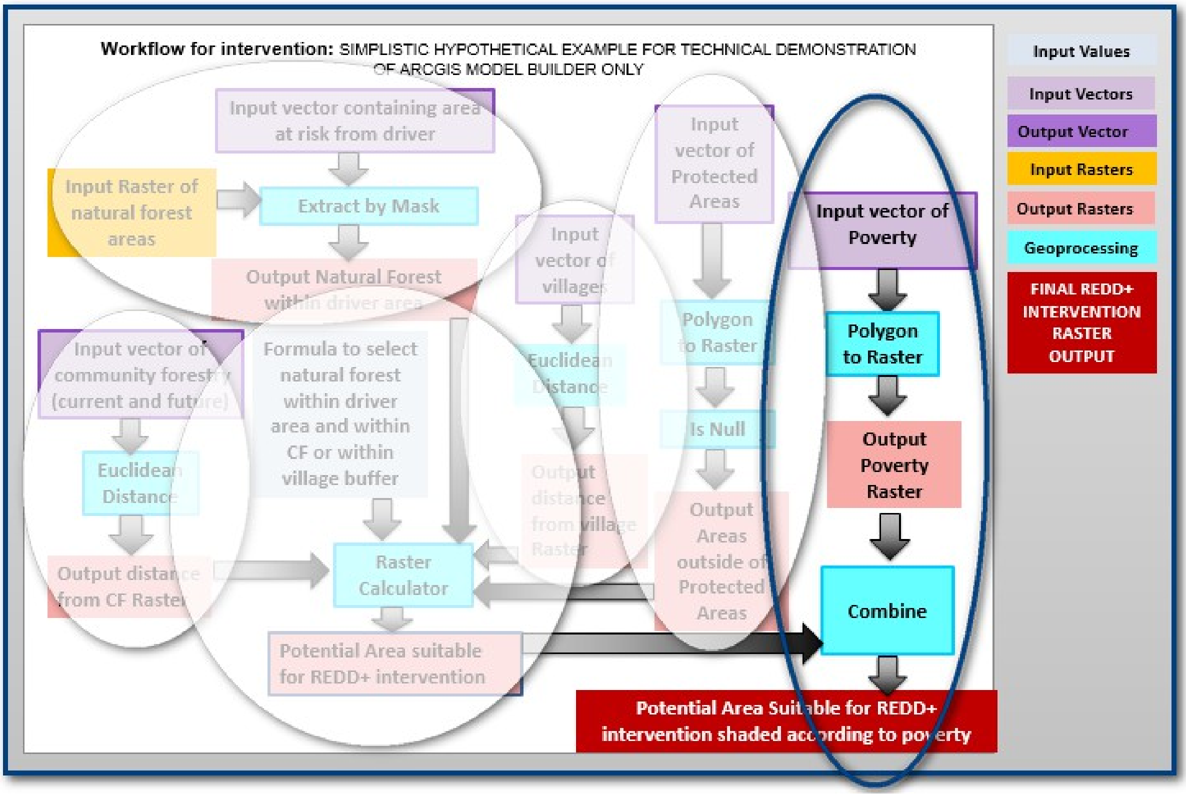

It is important to note that there is no Raster **Combination** tool in QGIS, but once again, we can use the **r.mapcalculator** tool to classify the **natural forest areas** layer **within the extent of the factor in the 10 km band around SC sites or in the 3 km band around villages and areas that are not protected areas** in terms of poverty.

a. The information concerning poverty in our example is an attribute of the vector dataset **communes_mythical**, so we can add a new **vector layer** called, for example, **Poverty vector** to the
b. Next, add a **table field** called, for example, **poverty attr** since this time we want to use the poverty field to creat rather than putting all the values in raster 1.
c. Add a new **v.to.rast.attribute** algorithm to **the processing generator**.
d. Change the description to **Step 11: Convert the poverty vector to raster.**
e. Select the **Poverty vector** parameter as the **Input Vector** layer**.**
f. Select the **poverty attribute** parameter as **Name of colu**
g. Set the **cell size**, for example to **1000.**
h. Click **OK.**

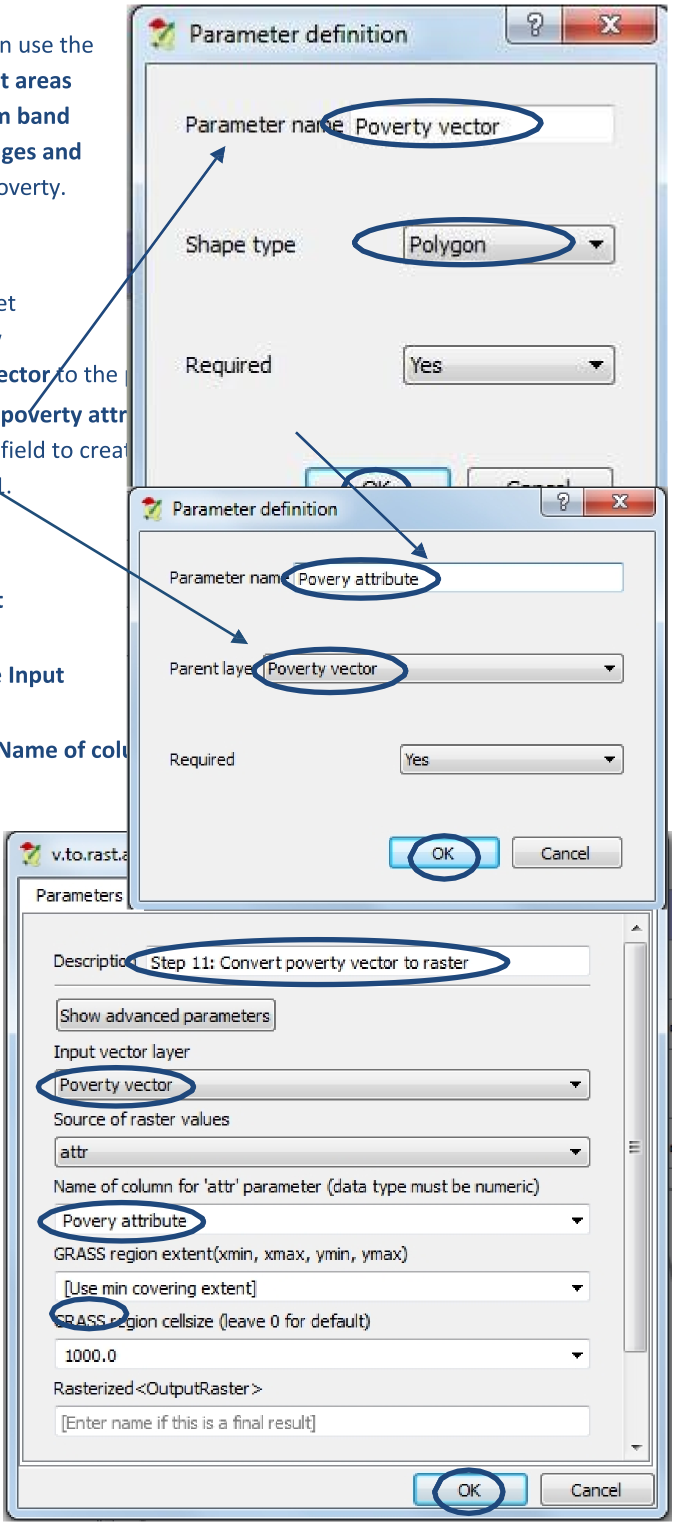

i. Add a new **Algorithm r.mapcalculator** to the **processing generator.**

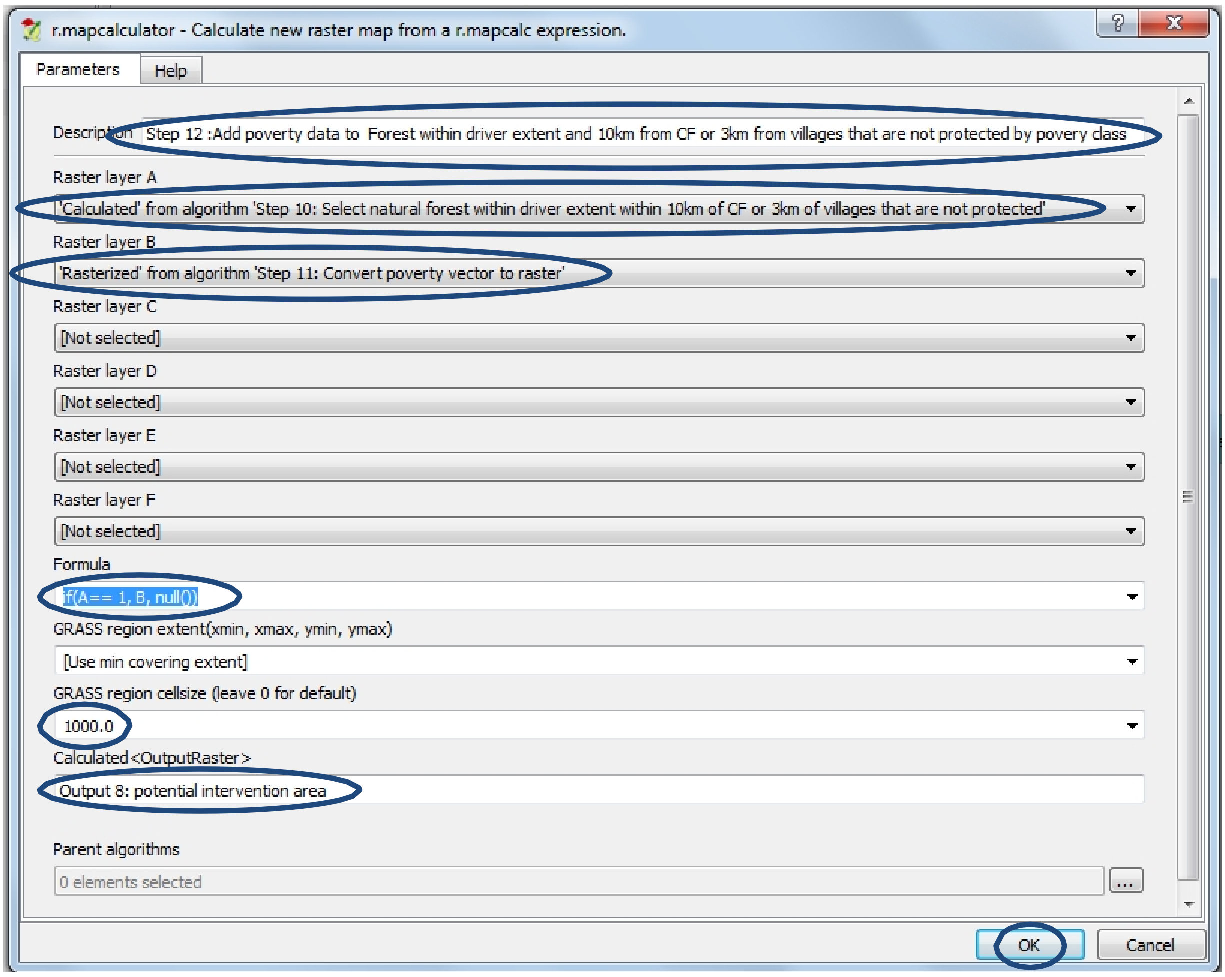

j. Write a **description** for the step**, for example, Step 12: Add poverty data to the forest within the factor extent and within 10 km or less of an SC site or 3 km or less of a village that is not protected by a poverty class.**

k. Select the **result of step 10** for **Raster A.**

l. Select the **result of step 11** for **Raster B.**

m. In the **formula window,** enter **if(A== 1, B, null())**

This means: "Values in A equal to 1 become poverty values in the output; other values in A become null values in the output."

n. **Click OK.**

The model is now complete and the user interface should look similar to this:

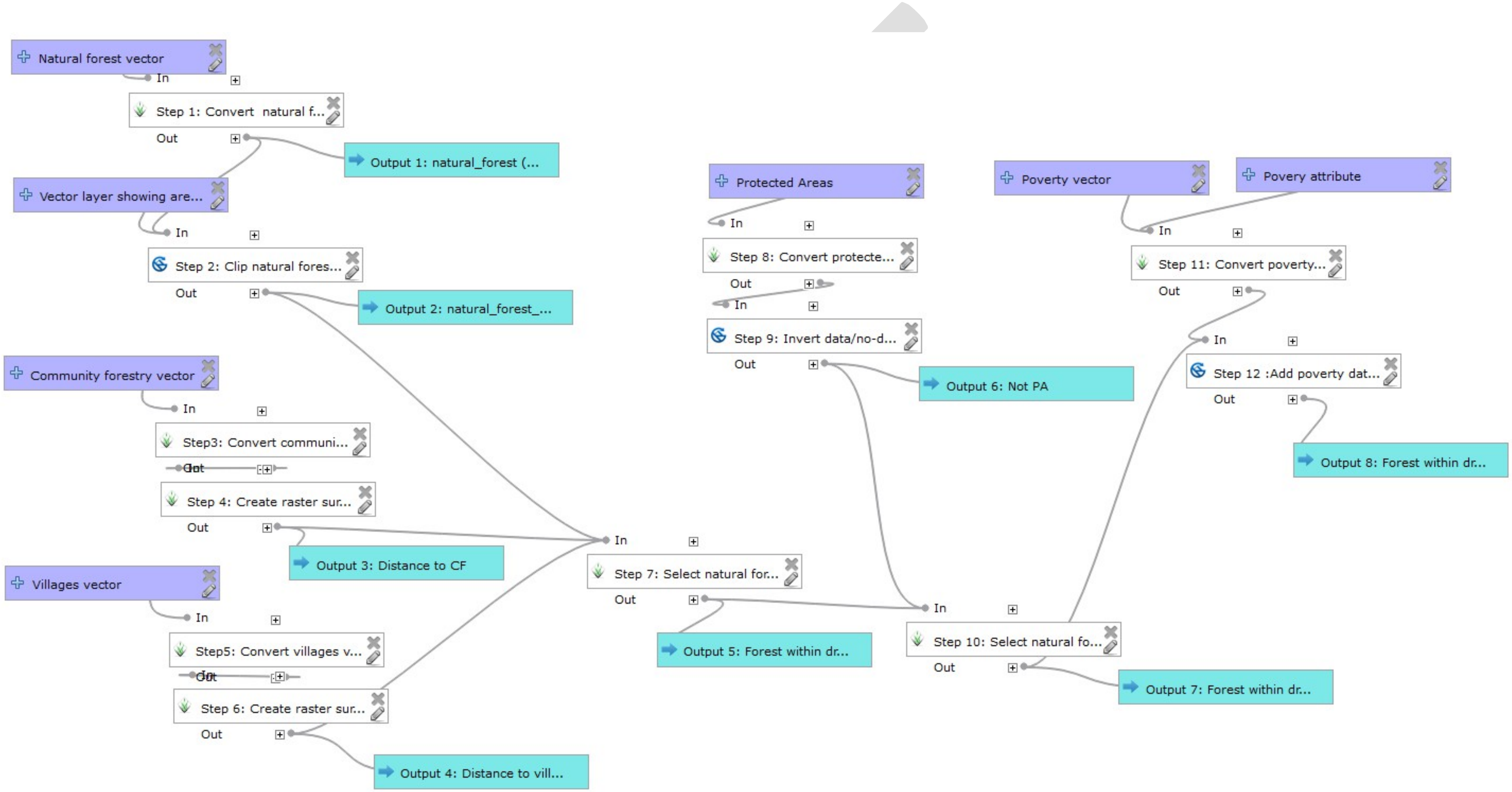

o. Save and **close** the **processing modeler.**

5.5. Set symbology for model outputs

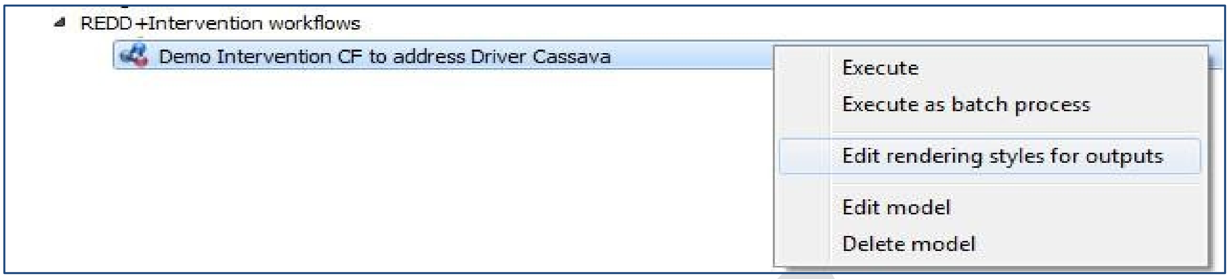

When the output of the tool is added to the table of contents, a default symbology is used. You can pre-configure the symbology for your results. In your model, right-click on the **Output Feature Class** and select **Edit Default Rendering Styles**. You can assign a predefined QGIS style file to determine the symbology as long as you know the range of output values, i.e., as long as you know the minimum and maximum values. For example, for processes that produce Boolean outputs, you know that the output values will be either 1 or 0; thus, the symbology can be created to display 1s and 0s in chosen colors. For some processes, you will not know the minimum and maximum values of the output file until the process is executed; under these conditions, a predefined style cannot be selected.
In this example, predefined style files have been created for all outputs.

a. Output 1 produces an output containing values of 1 or no data values. Click ... to load the style file for Output 1 **dark green raster 1_nodata.qml.**

b. Output 2 provides an output containing values of 1 or no data values. Click ... to load the style file for Output 2 **light green raster 1_nodata.qml.**

c. Output 3 provides an output with a range of distance values. We only want to know if the areas are above or below 3 km so that a style file can be downloaded, and we do not need to know the maximum value in the file. Click on ... to load the style file for Output 3 **cream_to_pink_0 to 3 km_dist.qml.**

d. Output 4 provides an output with a range of distance values; this time we are only interested in areas above or below 10 km. Click on ... to load the style file for Output 4 **cream_to_blue_0 to 10 km_dist.qml.**

e. Output 5 gives an output containing values of 1 or 0. Click ... to load the style file for Output 5 **brown green raster 1_0.qml.**

f. Output 6 gives an output containing values of 1 or no data values. Click ... to load the style file for Output 6 **grey raster 1_nodata.qml.**

g. Output 7 produces an output containing values of 1 or 0. Click ... to load the style file for Output 7 **orange raster 1_0.qml.**

h. Output 8 produces an output containing values of 1, 2, 3, or no data. Click ... to load the style file for Output 8 **yellow_orange_red raster 123_nodata.qml.**

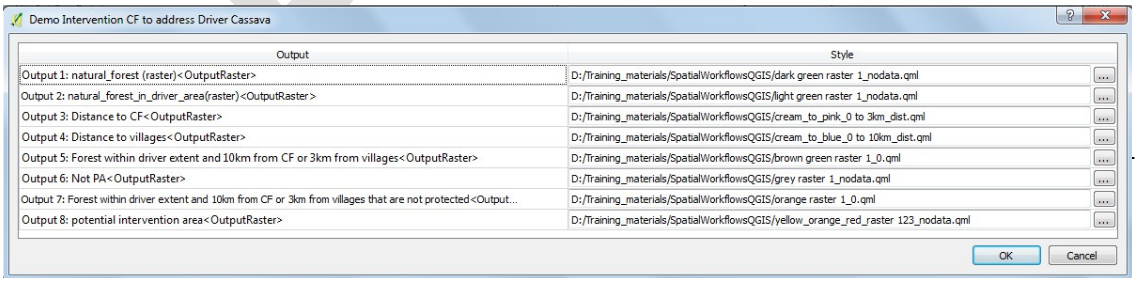

5.6. Run the entire model and check the results

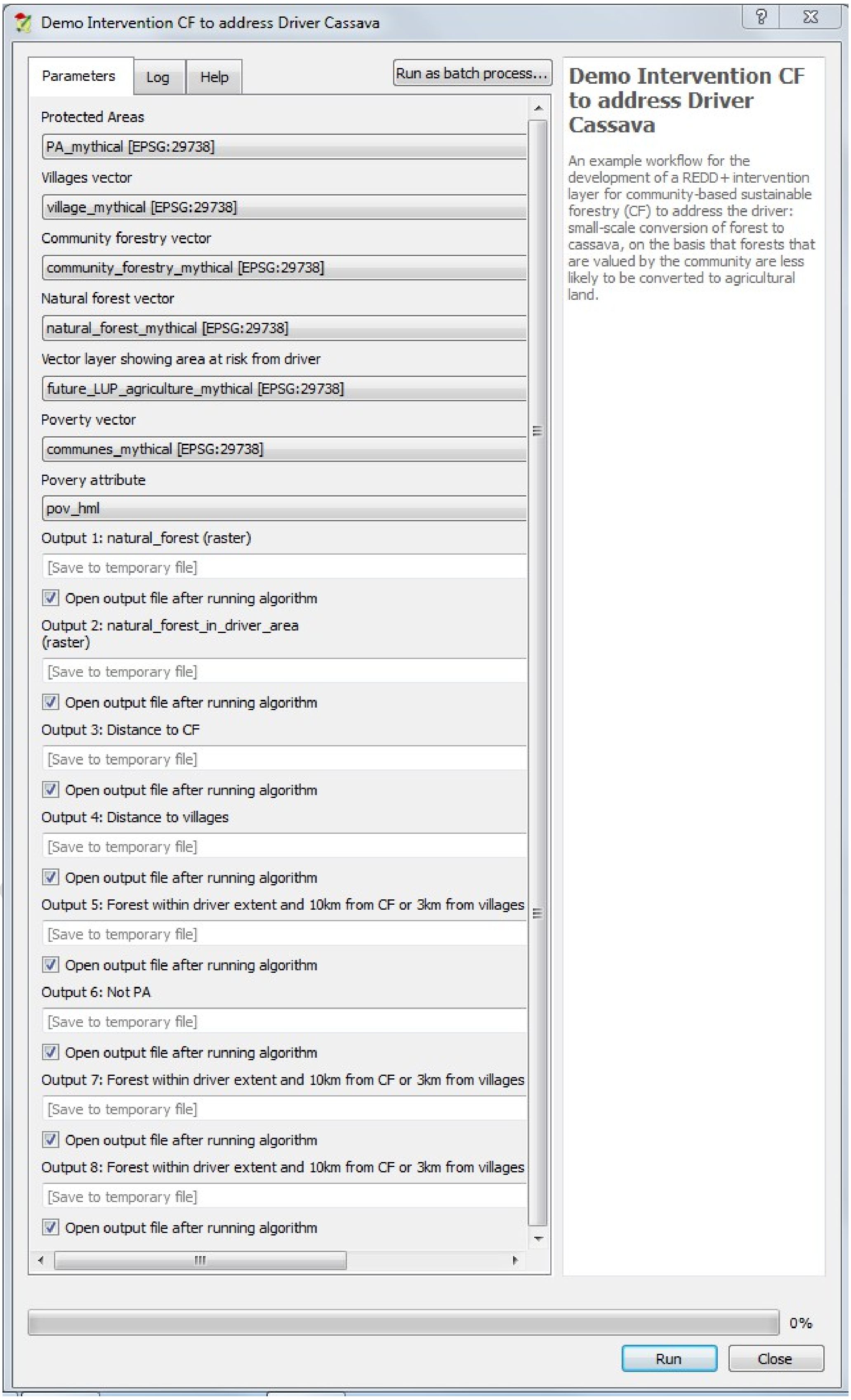

p. Double-click the model in the **Processing Toolbox** and click **Run** to execute the model.

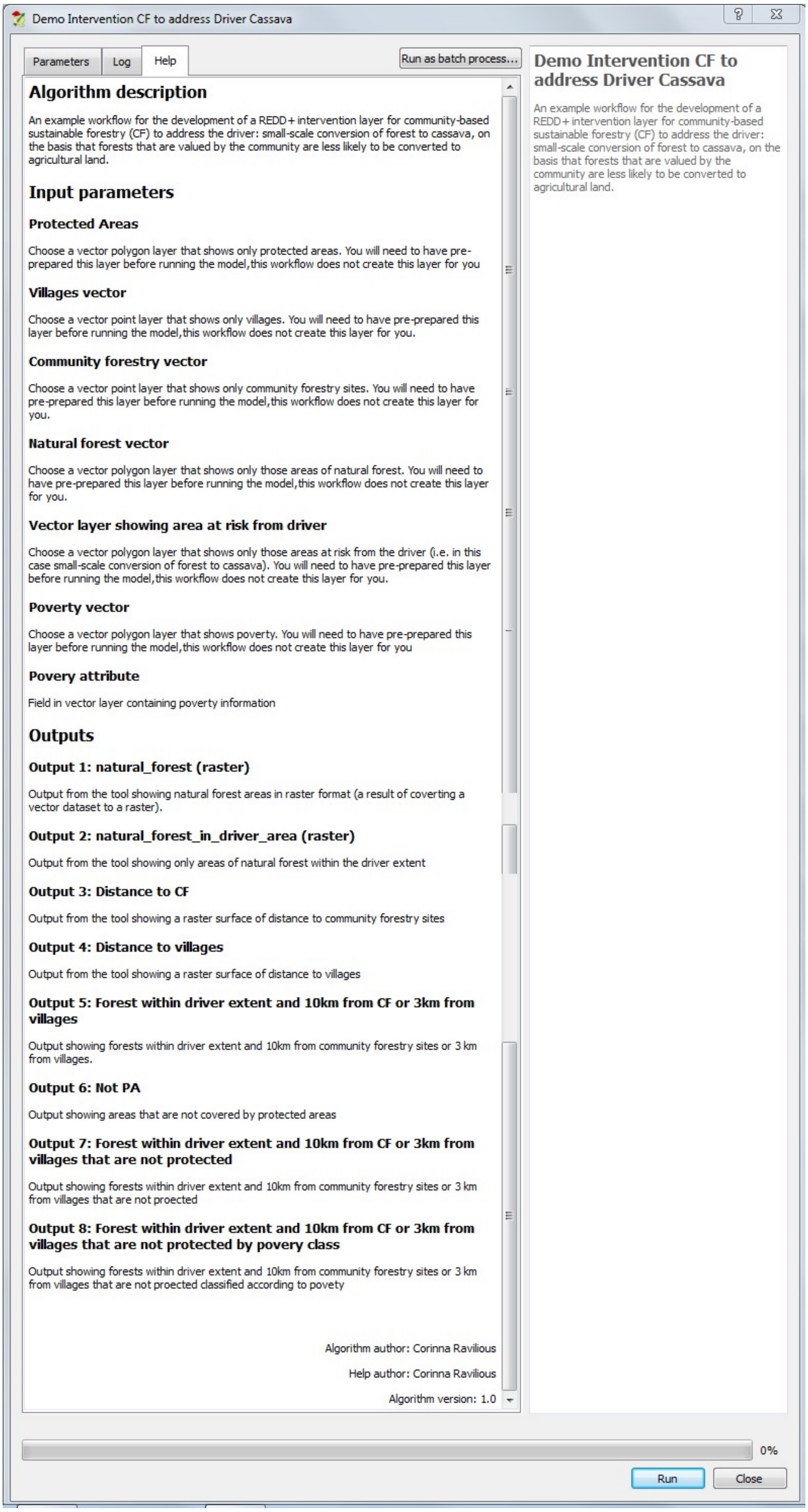

q. Click **Help** to see help for each of the entries.

r. Click on the **Settings** tab to return to the dialog box.

s. Fill in the settings for **inputs and outputs** as shown in the illustration below.

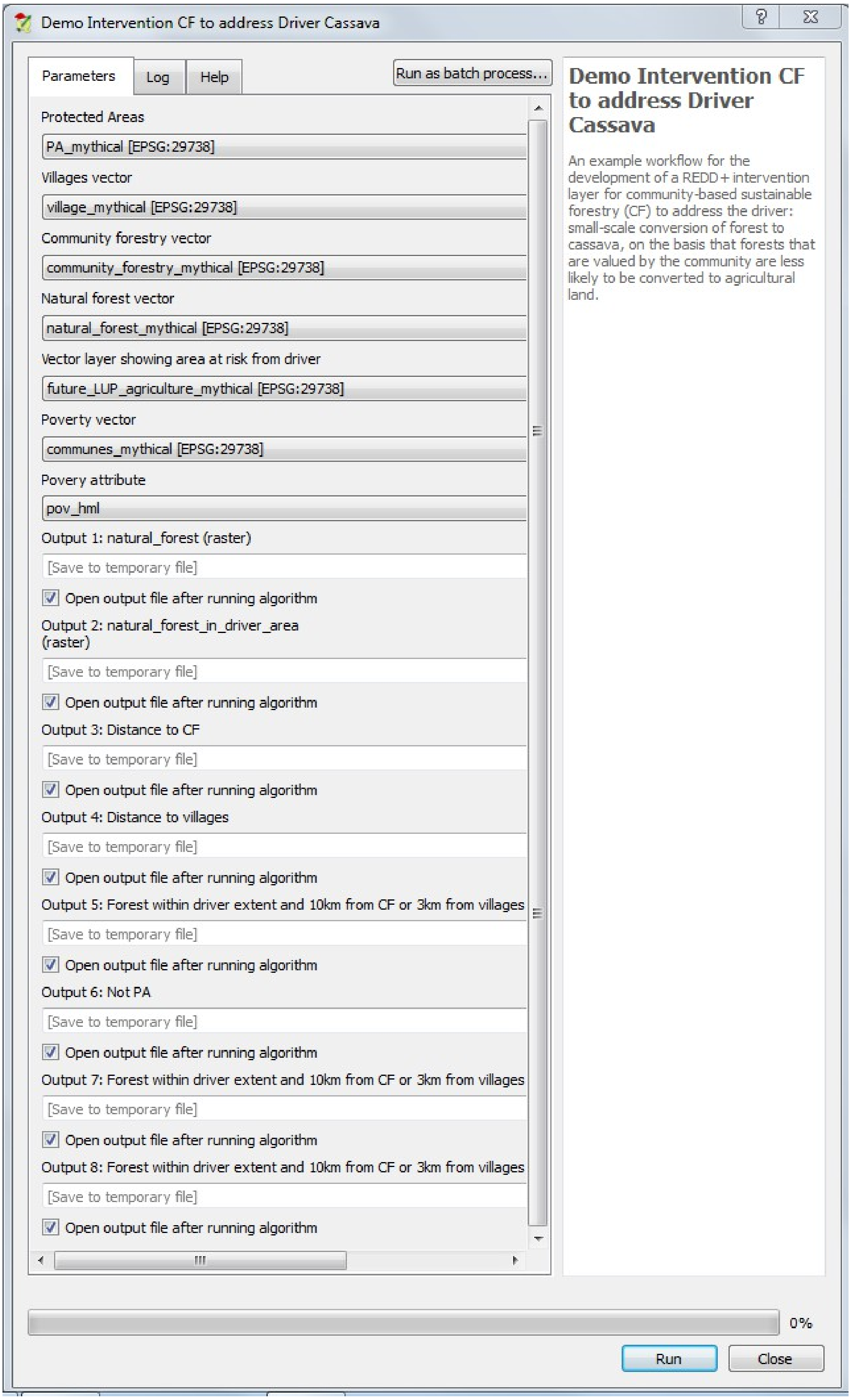

t. Click Run to **launch** the tool.

*****It is IMPORTANT to check the Model's outputs to ensure that there are no errors in the settings. Just because the model worked does not mean that it produced the expected results.*****

For example, in our demonstration model below:

➢ Output 1 corresponds to the natural forest raster layer (green). We can see that it correctly covers the entire district and that there are white **no data** areas where there
there is no natural forest.

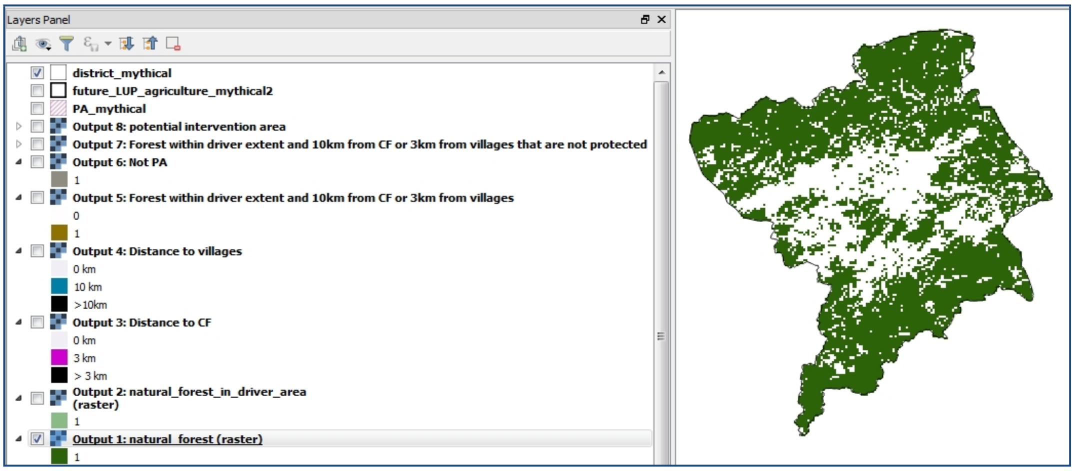

➢ Output 2 corresponds to the natural forest within the factor's extent. We can see that it correctly covers the total extent of the factor's area and that there are white **no data** areas where there is no natural forest.

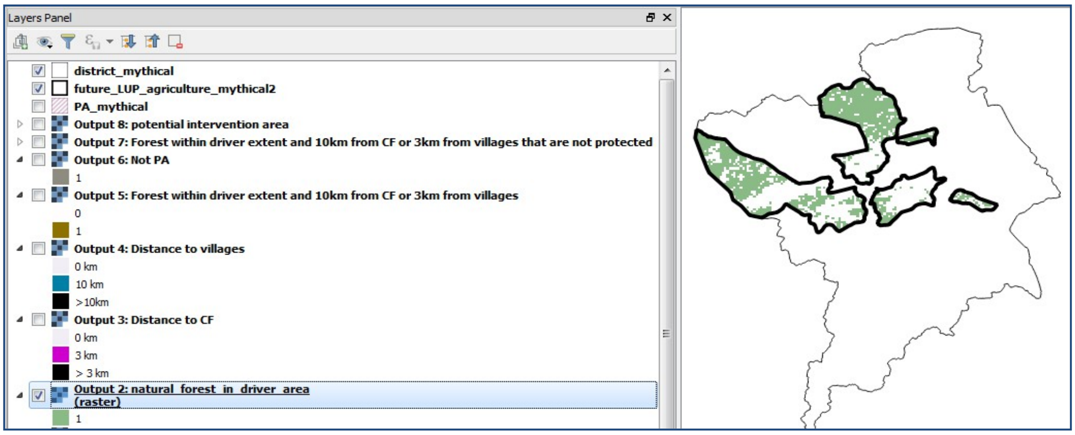

➢ Output 3 corresponds to the Euclidean distance from community forestry sites. We can see that it correctly covers the entire district,
but also extends beyond the district. This is not important for this step, as long as it covers the entire district. The black appears gray because transparency has been set in the output symbology.

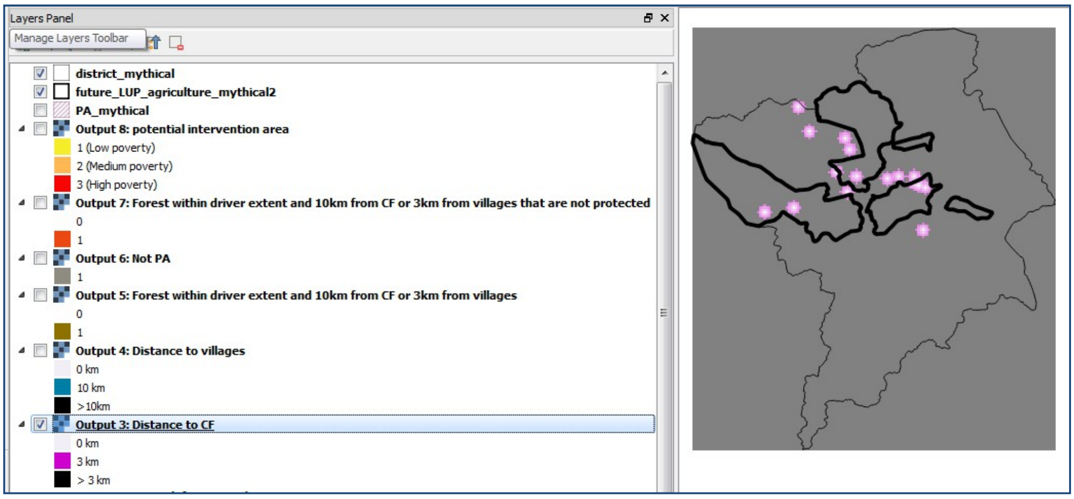

- Output 4 corresponds to the Euclidean distance from villages. Again, we can see that it correctly covers the entire district, but that it also extends beyond the district. This is not important for this step, as long as it covers the entire district. The black appears gray because transparency has been set in the outgoing symbology.

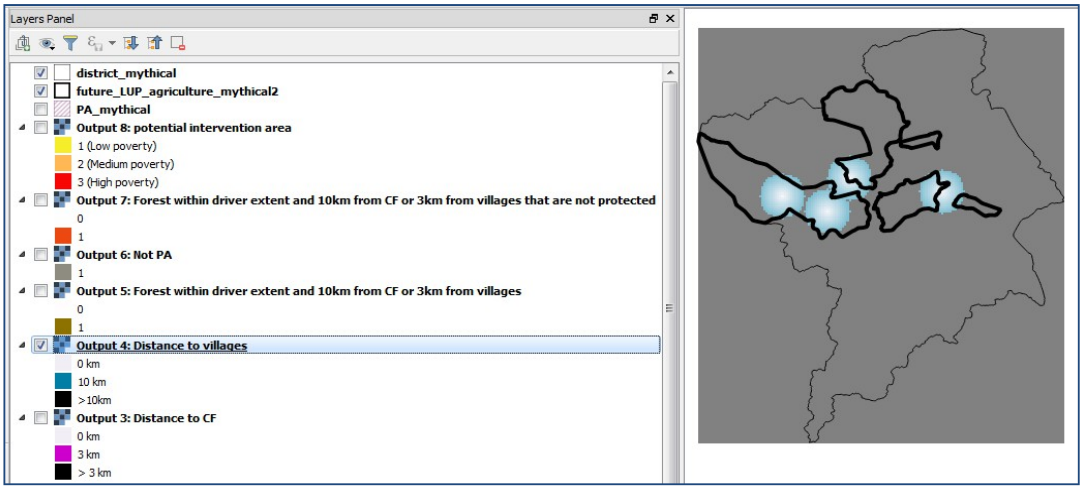

- Figure 5 shows the **brown areas**, which are the areas that have been selected as natural forest areas, located within the 10 km band around existing and proposed forestry sites. The **white areas** are those that were excluded in this step.

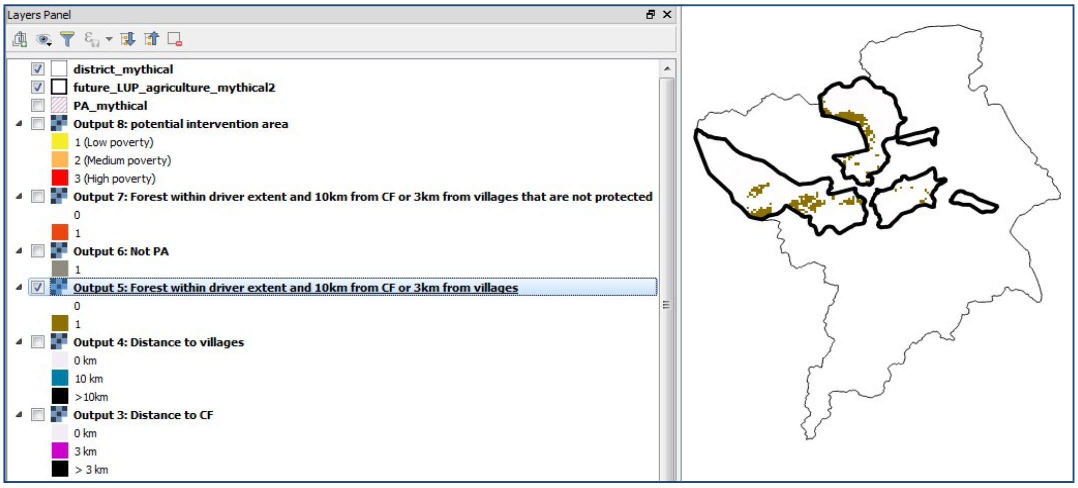

- Output 6 shows **gray areas** that are not protected and **white no data areas** that are protected.
- The outgoing 7 shows the **potential intervention areas in orange**; these areas have been selected as natural

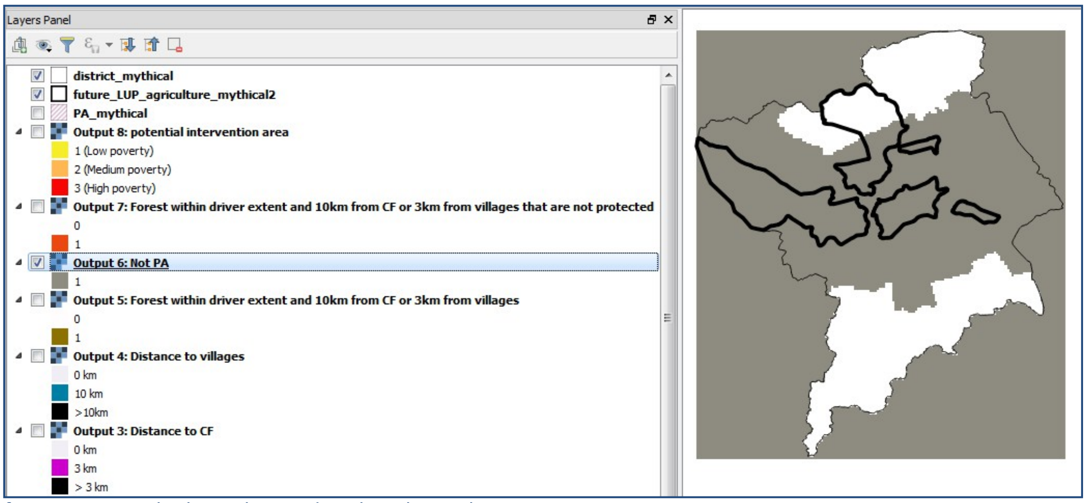

forest areas, which are located within the 10 km strip
around existing and proposed community forestry sites or within the 3 km strip around villages and outside protected areas.

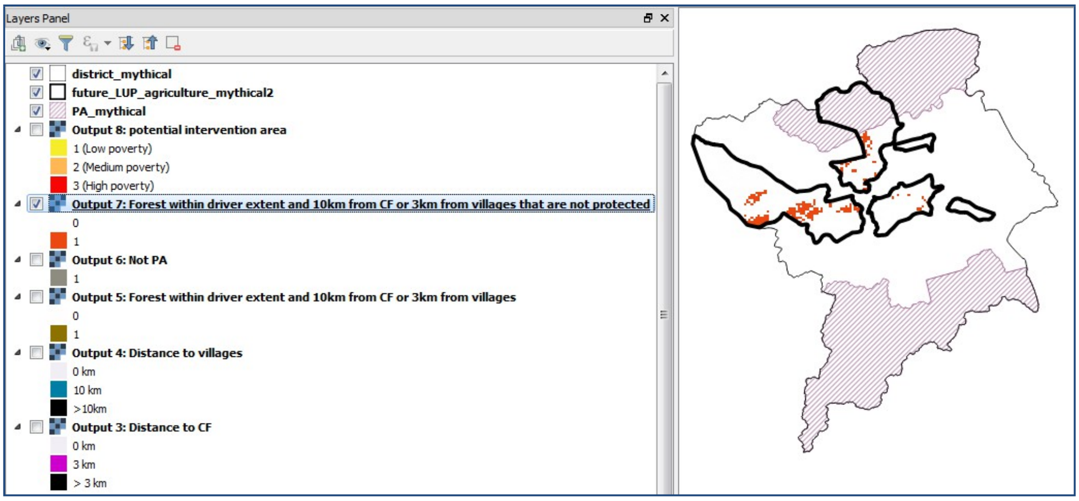

- Finally, if we look at the last layer, Output 8, we can refine **these potential intervention areas by poverty class.** You will see that the poverty class
shows values from 1 to 3 in this example; 1 = low, 2 =
medium, and 3 = high.

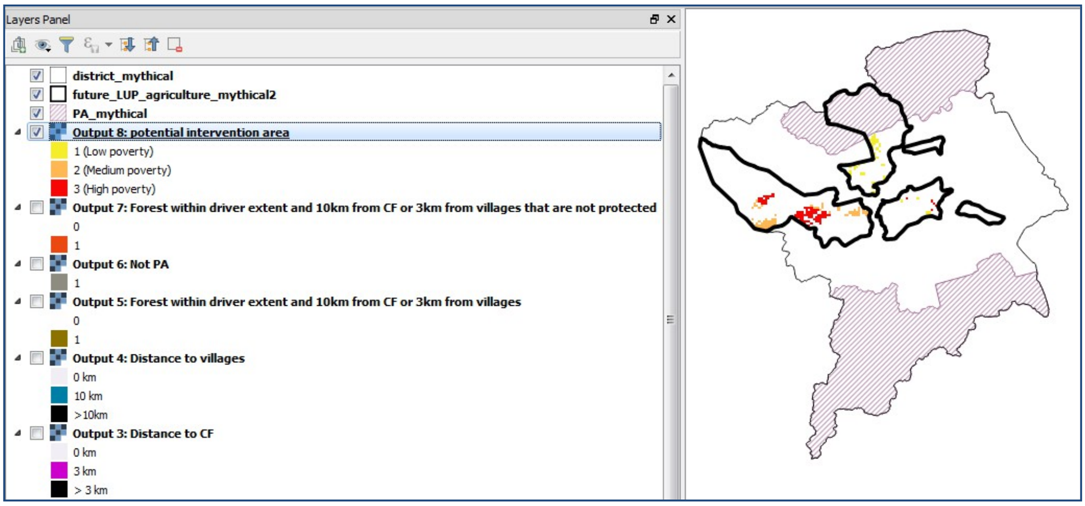

5.7. Sharing a QGIS template with other users

The easiest way to share a tool you have created is to **simply copy the model file using Windows Explorer**, just as you would for any other file. The default location for storing the model is C:\Users\xxxxxxxx\.qgis2\processing\models.

Some custom tools no longer work when changing QGIS versions, often because a geoprocessing tool has changed or works slightly differently from previous versions. In these cases, it may be necessary to select and delete this step in the processing modeler window and drag it into a new version of the tool.

A Appendix 1: Tips on raster analysis and geoprocessing tools in ArcGIS and QGIS

A.1. Vector vs. raster

A.1.1. Difference between vector and raster datasets

Data can be divided into two general types: **discrete data**, which represents objects and can be stored either as **vector entities or as raster**, and **continuous data**, which is generally (and preferably) stored as **raster data**. See Table 1 for more information on the differences between vector and raster data.

With **discrete data**, there is a specific starting point and ending point. It is easy to define the boundaries of the object—a lake, for example, is a discrete object since its boundaries can be perfectly established. Other examples of discrete objects include administrative units (i.e., the boundaries defining a region or country) or the boundaries between different land use classes. The cells belonging to each discrete object are assigned the same values and are stored as integers. **Integer** rasters cannot have decimals. Discrete data is sometimes referred to as categorical data.

With continuous data, values change smoothly across the landscape or surface, and there are no breaks as with discrete data. Cells have individual values, with adjacent cells often having different values from each other, but still showing a relationship. Examples of continuous data include elevation, slope, and aspect, or data such as the Normalized Difference Vegetation Index (NDVI) from a remote sensing image. The numerical values of the cell can be stored either as **integers** or as **floating-point** rasters. **Floating-point** rasters are necessary for storing **continuous** data that has attribute values containing decimals.

Table 1: Summary of some differences between vector and raster data

Vector data	Raster data
Points, lines, polygons	Area with **cells or pixels** of regular
Storage of discrete data, for example, data with discrete boundaries such as country borders, roads, streets, or locations of places.	Raster cells storing either **discrete** data (classified land use data) or **continuous** data (altitude or remote sensing image). Raster data is particularly suitable for continuous data.
Complex spatial relationships may exist within and between vector layers. Topology rules can be set (i.e., relationships between adjacent or surrounding entities) to prevent them from overlapping.	Spatial relationships are based solely on the location, size, and alignment of cells/pixels.
Vector data has attribute tables. For each feature (in single datasets) or group of features (in multiple datasets), fields can be added to store additional information	For entire rasters, cell values can be stored in summary tables called Value Attribute Tables (VAT). Additional information can be stored in fields for

polygon(s), point(s), and line(s).	groups of cells with the same value. No attributes can be added to individual cells/pixels.<br>ArcGIS can create and view VATs, unlike QGIS. Floating-point rasters never have attribute tables.<br>attribute tables.
Queries, selections, and overlays are possible. Overlaying too many datasets can result in small polygon fragments where the boundaries do not match each other. This tends to slow down analyses or, when the problem is significant, can cause analyses to fail, especially when the combined data becomes too heavy for<br>process.	As with queries, selections, and overlay analyses, there are many other analyses that are more suitable or only possible with raster data. In order to perform the analysis, the data is transformed to a homogeneous cell resolution, allowing for fast and efficient analysis of multiple datasets.

A.1.2. Vector vs. raster analysis

Many other analyses are more suitable or only possible with raster data. You can create, query, map, and analyze data with both raster and vector data types. However, vector analysis only allows you to perform a few simple overlay operations, while more complex, multi-criteria analysis options are available with rasters. Raster analysis functions also allow you to perform raster and integer vector analyses, derive new information from existing data, and execute multi-criteria queries using multiple layers of data.

Table 2 provides a brief overview of some of the most common vector and raster functions. This will help you identify and locate raster functions that are equivalent/similar to vector functions. There may also be other functions, not listed here, that perform similar tasks. **It is important to read the help for each tool, as the table only provides a general indication of the function of the various tools listed. The tools may work slightly differently**. The descriptions and names of the tools are mainly based on information from the ArcGIS and QGIS processing toolboxes. In ArcGIS, from the main menu, click **Help >> ArcGIS Desktop Help** or access the "Help" information for the tools in the ArcGIS toolbox. In QGIS, refer to the user guide accessible from the main menu **Help >> Help Contents** or by clicking the **Help** button in the tool dialog box. Note that QGIS help in the tools is not always available and you may see a message saying "Sorry, no help is available for this algorithm."

In QGIS, more information can be obtained by looking at the help page on the web:

GRASShttps://grass.osgeo.org/documentation/manuals/

SAGA http://www.saga-gis.org/en/index.html
GDALhttp://www.gdal.org/gdal_utilities.html

Table 2: Overview of some of the most common vector and raster functions

Extraction tools	Example of vector tools		Example of raster tools	
	ArcGIS	QGIS	ArcGIS	QGIS
**Vector:** Extracts incoming features that are within the boundaries of the clipping features. Features that are partially within the clipping feature layer are clipped along the boundary of the clipping feature layer. **Raster:** Extracts raster cells that match areas defined by a mask, polygon, circle, or bounding box (extent).	Clipping (Clip)	Clipping (QGIS)<br>Clipping vectors according to an extent (OGR)<br>Clipping vectors to a polygon (OGR)<br>Clip points with polygons (SAGA) Cut vector layer (SAGA) v.overlay (GRASS)<br>v.select (GRASS)	Extract by mask Extract by polygon<br>Extract by rectangle<br>Extract by circle	Cut a raster according to an extent (GDAL)<br>Clip raster by mask layer (GDAL)<br>Clip raster with polygon (SAGA)
**Vector:** Extracts entities/tables stored using expressions. ArcGIS uses SQL (Structured Query Language). QGIS uses its own expressions, which are specified in the user guide https://docs.qgis.org/2.14/en/docs/user_manual/working_with_vector/expression.html<br>**Raster:** Extracts cells from a raster	Select Select a table (table select)	Select by attribute (QGIS) Select by sum of attributes (QGIS)<br>Select using an expression (QGIS)<br>v. extract (GRASS)	Extract by attribute	Add raster values to points (SAGA) - add raster values to points Add raster values to features (SAGA) - add raster values to features Raster values to points (SAGA)<br>Raster values to features (SAGA)

DRAFT

based on a logical query. Here again, ArcGIS and QGIS have different syntaxes. In QGIS, the syntax may change depending on whether you are using QGIS, GDAL, SAGA, or GRASS.				
**Vector:** Decomposes the input features to create a subset of multiple output layers from a decomposed layer.<br>**Raster:** Creates a grid output from an input raster dataset.	**Split**	**Split a vector layer (QGIS)**	**Raster split tool**	**Gdal2tiles (GDAL)**
**Vector:** Selects features in one layer based on their relationships with features in another layer.	**Select layer based on location**	**Extract by location (QGIS)**	**N/A**	**N/A**
**Proximity tools**	**Example of vector tools**		**Example of raster tools**	
	**ArcGIS**	**QGIS**	**ArcGIS**	**QGIS**
See summary here:http://resources.arcgis.com/EN/HELP/MAIN/10.1/index.html#//018p0000000000 0<br><br>**Vector:** Straight-line distance tools to create buffer zones at a specific distance.<br>**Raster:** Various distance tools	**Buffer Multiple circle Buffer**	**Fixed distance buffer (QGIS) Fixed distance buffer (SAGA) - fixed distance buffer**<br>**Variable buffer distance (QGIS)**<br>**Variable distance buffer (SAGA) - Variable buffer distance**<br>**v.buffer.distance (GRASS)**	**Euclidean distance**<br>**Travel cost (Cost distance)**	**Proximity (raster distance) (GDAL)**<br>**r.grow.distance (GRASS)**<br>**r.grow (GRASS) r.cost (GRASS) r.cost.full (GRASS)**<br>**r.cost.full.raster (GRASS)**<br>**r.spreadpath (GRASS)**

**Proximity tools**	**Example of vector tools**		**Example of raster tools**	
that calculate distances more complex than just a straight line, taking into account factors such as altitude, etc.		**v.distance (GRASS)**<br>**v.distance.toattr (GRASS)**	**Shortest distance (Path distance)**	**Least cost paths (SAGA)**<br>**Accumulated cost (anisotropic) (SAGA)**<br>**Accumulated cost (isotrophic) (SAGA)**
**Vector:** Each Thiessen polygon contains only one point entity as input. Any location inside a Thiessen polygon is closer to the point associated with that polygon than to any other incoming point feature.<br>**Raster:** divides an area and assigns each cell to the closest input feature that has a value.	**Create Thiessen polygons**	**Voronoi polygons (QGIS) v.voronoi (GRASS)**	**Euclidean attribution**	**r.grow.distance (GRASS)**

**Statistical tools**	**Example of vector tools**		**Example of raster tools**	
	**ArcGIS**	**QGIS**	**ArcGIS**	**QGIS**
**Vector:** Creates a table with statistics based on polygon contiguity (overlaps, coincident edges, or points).<br>**Raster:** Calculates, for each incoming cell location, a statistic of the values in a specified neighboring space.	**Neighboring Polygons**	**NNjoin (QGIS extension)**	**Neighborhood toolbox: Focal statistics tool**	**Raster layer statistics (QGIS)**

Statistical tools	Example of vector tools		Example of raster tools	
Reads a table and a set of fields and creates a new table containing unique field values and the number of occurrences of each unique field value.	**Frequency**	**Frequency Analysis (QGIS)**	For entire rasters, the attribute value table is similar to a frequency table in that it contains unique field values and the count of the number of cells.	**Unique values count (SCRIPT) Extract raster values to CSV (SCRIPT)**
Calculates summary statistics for table field(s)	**Summary statistics**	**Basic statistics for numeric fields (QGIS) Basic statistics for text fields (QGIS)**<br>**Statistics by categories (QGIS)**	N/A	N/A
Calculates statistics on the values of a raster in the areas of another dataset.<br><br>Summarizes the values of a raster in the areas of another dataset and reports the results in a table.	N/A	**N/A**	**Zonal statistics tool, zonal statistics as a table tool**	**r.stats (GRASS) r.univar (GRASS) r.statistics (GRASS)**<br>**Zonal raster statistics (SAGA) Zonal statistics (QGIS)**<br>**Raster statistics for polygons (SAGA)**
Calculates the intersecting areas between two datasets and creates a table.	N/A	N/A	**Cross-Classification and Tabulation (SAGA)**	**Cross-Classification and Tabulation (SAGA)**
Calculates a statistic per cell from multiple rasters.	N/A	N/A	**Statistics for**	**Statistics for rasters (SAGA) r.report (GRASS)**

Statistical tools	Example of vector tools		Example of raster tools	
The available statistics are Majority, Maximum, Mean, Median, Minimum, Minority, Range, Standard deviation, Sum, Variety			**Cell**	

Overlay tools	Example of vector tools		Example of raster tools	
	ArcGIS	QGIS	ArcGIS	QGIS
**Vector: Union tool** - All features and their attributes will be written to the output layer. **Identity tool** - Incoming features or portions that overlap identity features will have the attributes of those identity features. **Intersection tool** - Features and portions of features that overlap in all layers and/or feature classes will be written to the output layer.<br>**Raster:** Combines multiple rasters so that a single output value is assigned to each unique combination of input values.<br>Note: See also the multi-criteria analysis tools, which are listed separately in this table under raster-specific tools.	**Union Identity**<br>**Intersection**	**Union (QGIS)**<br>**Polygon identity (SAGA)**<br>**Intersect (SAGA) Intersection**<br>**(QGIS) v.overlay (GRASS)**<br>**v.patch (GRASS)**	**Combination** (Note: all cells in the input rasters that have a no data value will be excluded from the output raster even if these cells contain data in some of the input rasters).	**r.cross (GRASS)**<br>**Cross-classification and tabulation (SAGA)**

Overlay tools	Example of vector tools		Example of raster tools	
**Vector:** Calculates a geometric intersection of Input Features and Updated Features. Attributes and geometry are updated by the updated features in the output layer.<br>**Raster:** Creates a mosaic from multiple raster datasets into a new raster dataset.	**Update**	**Polygonization (QGIS)**	**Mosaic to a new raster**	**Mosaic raster layers (SAGA) r.patch (GRASS)**

**Specific raster tools**	**Example of raster tools**	
	**ArcGIS**	**QGIS**
**Multi-criteria analysis tools:**<br>Overlay analysis tools allow you to apply weighting to multiple inputs and combine them into a single output.<br>The most common application is for adaptability modeling.	**Fuzzy membership**<br>Transforms the input raster into a scale from 0 to 1, indicating the importance of belonging to a set, based on a specific fuzzy processing (fuzzification) algorithm.<br>**Fuzzy Overlay**<br>Combines fuzzy membership raster data together based on a selected overlay type.<br>**Weighted Overlay**<br>Overlays multiple rasters using a common measurement scale and weights each of them according to their importance. **Weighted sum**<br>Overlays multiple rasters, multiplying each by its own weight and adding them together.<br>For more details, see the Help pages: http://help.arcgis.com/en/arcgisdesktop/10.0/help/index.html#/An_overview_of_the_Overlay_tools/009z000000rm00000/ and this article on fuzzy logic: http://www.esri.com/news/arcuser/0410/fuzzylogic.html	**Fuzzify (SAGA)**<br>Converts grid values into fuzzy membership sets in preparation for fuzzy logic analysis.<br>**Fuzzy union (or) (SAGA)**<br>Calculates the union (max operator) for each cell in the selected grids.<br>**Fuzzy Intersection (and) (SAGA)**<br>Calculates the intersection (min operator) for each cell in the selected grids.<br>**Ordered weighted averaging (SAGA)**<br>Overlays multiple rasters using a common measurement scale and weights each of them according to their importance.<br><br>See the help section for grid calculation tools (Grid Calculus) in SAGA: http://www.saga-gis.org/saga_tool_doc/3.0.0/grid_calculus.html and grid analysis tools: http://www.saga-gis.org/saga_tool_doc/3.0.0/grid_analysis.html
**Conditional evaluation:**<br>performs a conditional evaluation "if/else" evaluation on each of the input cells of an input. For example, if a value is > x, then it performs a certain operation, and if it is not	**Tools con**<br><br>**(this function can also be performed in the raster calculator)**	**This function can also be performed in the raster calculator.**<br>**Seehttp://gis.stackexchange.com/questions/69734/what-is-the-equivalent-of-arcpy-con-in-qgis-and-or-r-raster-package**

Specific raster tools	Example of raster tools	
If it is not, it executes another one.		
**Set Null tool** returns the locations of cells identified as No Data based on a specific criterion. It returns a No Data value if a conditional evaluation is true and returns the value specified by another raster if it is false.	**Set Null**<br><br>**(this function can also be performed in the raster calculator)**	**This function can also be performed in the raster calculator.**
**Math tools** that perform mathematical operations on rasters.<br><br>**Logical Math tools** are useful for multi-criteria analysis. They evaluate inputs and determine output values based on Boolean logic.	**The Math toolset** in ArcGIS contains tools that perform mathematical operations on rasters. **The Logic Math toolset** contains tools for performing logical evaluations on rasters that check whether statements are true or false. The tools are divided into the following categories: **Boolean, Combinatorial, Relational,** and **Conditional.**<br>➢ **Boolean tools** only evaluate the truth or falsity of conditions and<br>return the result of a particular tool as a Boolean value of 1 or 0 (True or False).<br>➢ **Combinational tools** identify unique combinations of input values based on<br>based on the logic of a particular tool and return a different value for each unique combination.<br>➢ **Boolean tools** compare the values of one input to another input and	The **Raster Calculus toolset (SAGA)** in QGIS contains tools that perform mathematical operations on rasters.<br><br>**There is no Math Logic tool in QGIS. These functions can also be performed in the raster calculator.**

Specific raster tools	Example of raster tools	
	return the result of a particular tool as a Boolean value of 1 or 0.<br><br>**(these functions can also be performed in the raster calculator)**	
**Raster calculator**<br>Allows you to perform calculations customized based on the values of raster datasets.	**Raster calculator**<br>Builds and executes a single algebraic expression of map using Python syntax in a calculator-type interface.<br>calculator-like interface.	The syntax varies depending on the calculator you are using:<br>**Raster calculator (QGIS)**<br>https://docs.qgis.org/2.2/en/docs/user_manual/working_with_raster/raster_calculator.html<br><br>**r.mapcalculator (GRASS)**<br>https://grass.osgeo.org/grass73/manuals/r.mapcalc.html<br>**Raster calculator (SAGA)**<br>http://www.saga-gis.org/saga_tool_doc/3.0.0/grid_calculus_1.html

A.2. Raster analysis in QGIS

The **processing toolbox** provides QGIS users with access to a wide range **of raster analysis tools** in QGIS, GDAL, SAGA, GRASS, and more. Some raster tools are also accessible from **the raster tab** in the main menu.

To convert vector data to raster, go to the main menu and select **Raster >> Conversion.**

Note: In QGIS, there are several tools that are very similar. Different tools may have different options. It is important to read the help section and, once the tool has been executed, to carefully check the results to ensure that an appropriate output has been created for your task.

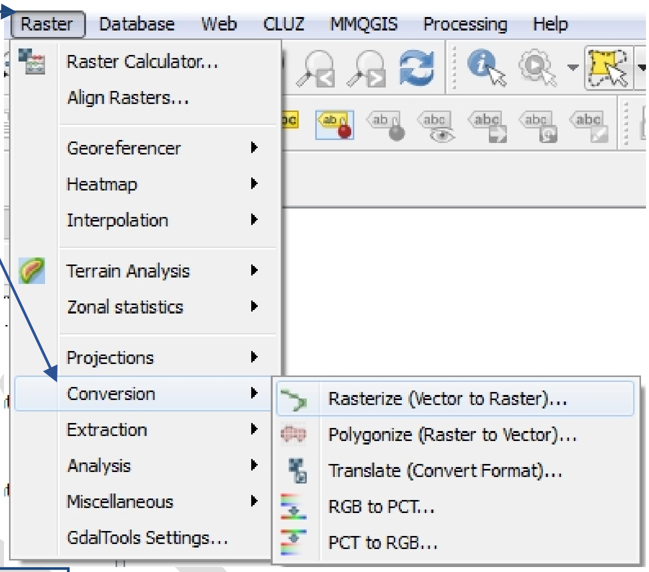

Many raster conversion tools can be found in **the Processing Toolbox.**

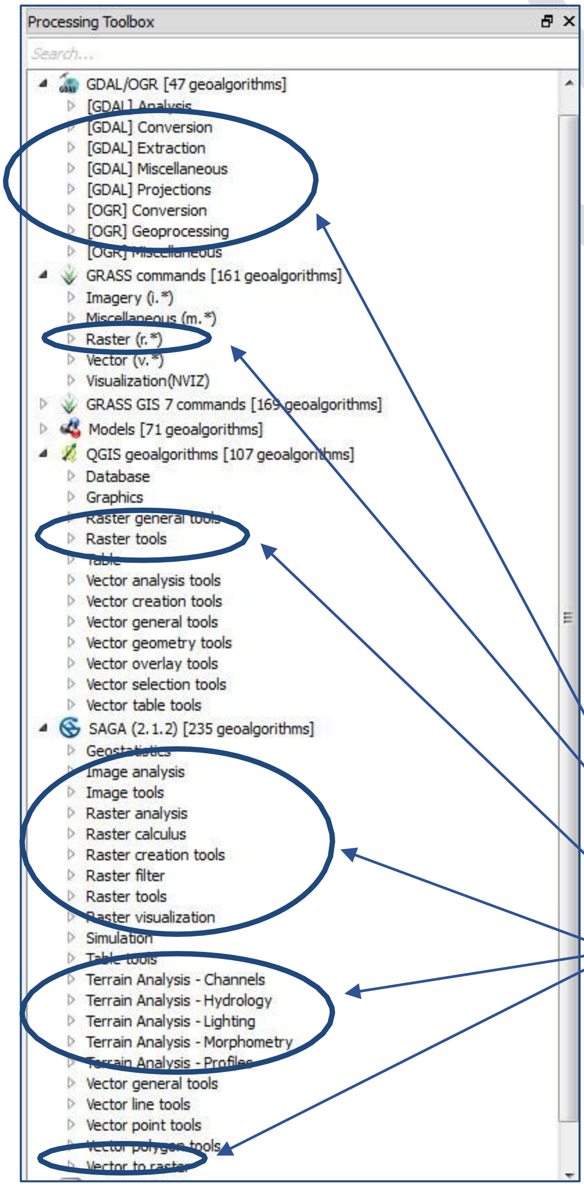

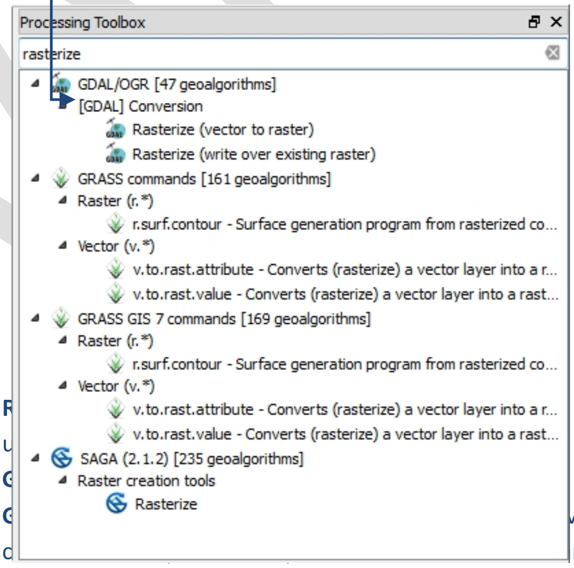

[illegible] vector [illegible] nder two raster subgroups

SAGA – raster tools listed under different raster subgroups

Some of the advantages of raster analysis include the ability to:

- Integrate vector and raster analyses
- Obtain new information from existing data
- Make queries on multiple layers
- Performing complex analyses in a shorter time frame

A.2.1. Raster analysis and environment settings

You may be familiar with adjusting **Environment Settings** for geoprocessing in ArcMap. Settings are additional configurations that can be added to modify the results of a tool. There are fewer hidden settings in QGIS, but there are often settings that are generally optional in individual tools.

When performing raster analysis, it is very important to ensure that cells are not misaligned (causing data slippage) in the output layers.

In **GRASS** tools, there are parameters that can be used to prevent such slippage.

- **Extent of the GRASS region (xmin, xmax, ymin, ymax) that can be configured significantly throughout the analysis.**
- **Size of the GRASS region cell**

Similarly, in **SAGA** tools:

- **output extent (xmin, xmax, ymin, ymax)**
- **Cell size**

In the graphical modeler, there are also some tools reserved for models that allow the user to configure the parameters of **raster layer boundaries** and **vector layer boundaries** that retrieve a geographic bounding rectangle from a vector or raster dataset that will be used as the extent.

Since QGIS accesses algorithms from different providers, it is even more important to verify that the analysis steps produce consistent results.

A.2.2. Workspaces

When performing geoprocessing in QGIS, you can decide whether the output produced by the tool is permanent or temporary. For temporary outputs, the datasets are stored in an output file specified in the processing options from the **Processing** tab of the

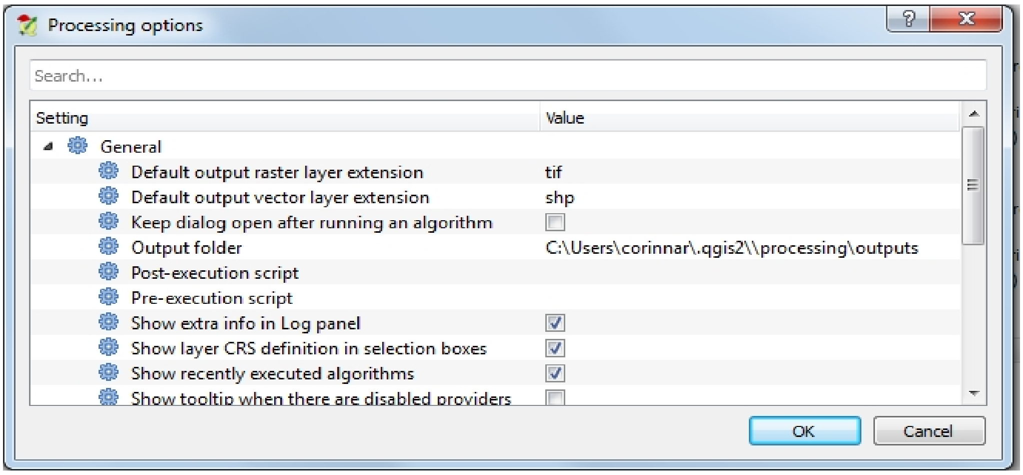

main menu bar.

A.2.3. Output Coordinates

In QGIS, there are several options for configuring the output coordinate system. You can preconfigure the Output Coordinate System for output datasets for the entire project.

a. From the QGIS main menu, click **Preferences>>Options**

b. Click on the **SCR** tab.

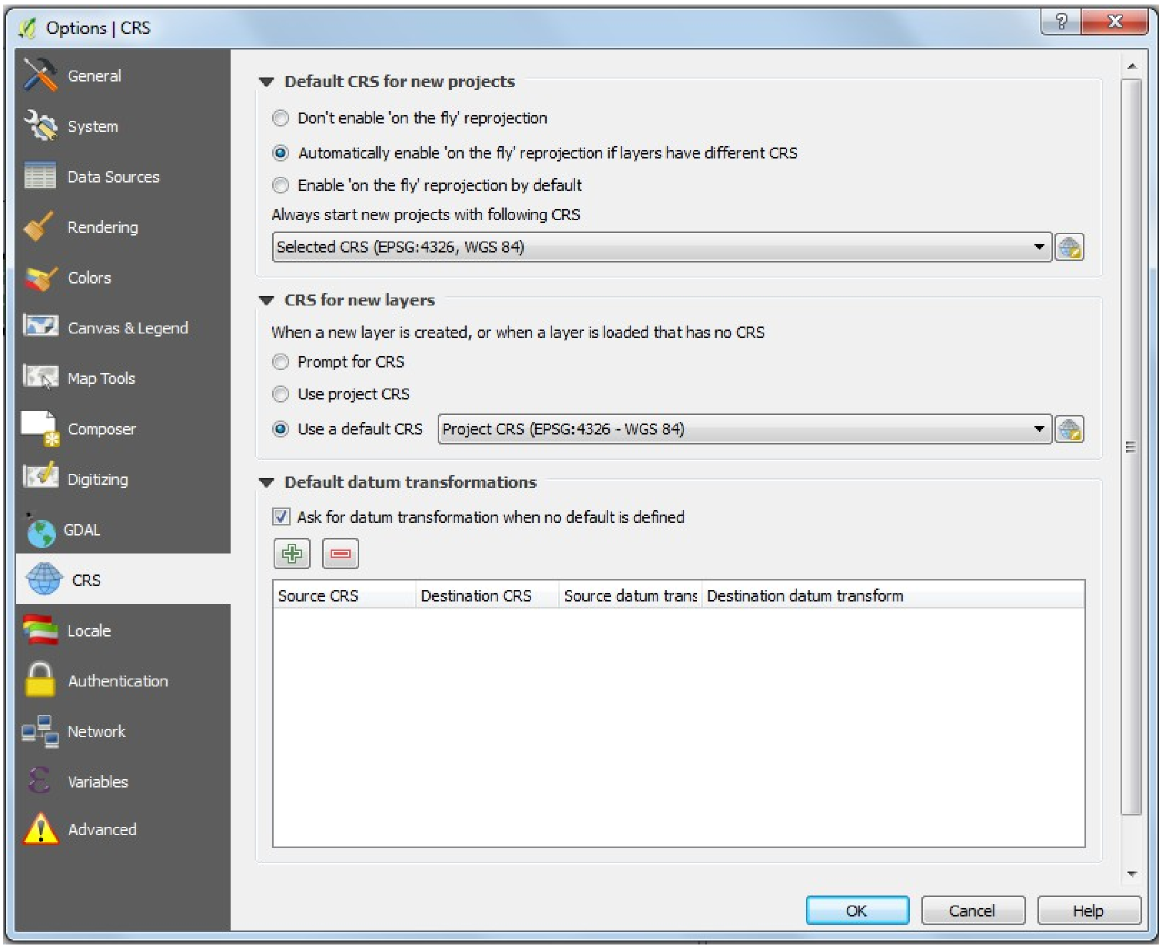

A.2.4. Cell Size

When you configure the cell size in raster analysis, the tools will use this cell size for the cell size of the output rasters, or the resolution, for the operation. By default, the output resolution is determined by the coarsest of the input raster datasets. **

It is important to read the help associated with the tool you are using to ensure that it correctly transforms the data to a different resolution. In some cases, it may be preferable to use ***resampling*** *or* ***aggregation*** *before performing an analysis, since cells are resampled using the nearest neighbor resampling in the environment settings, and this may not necessarily be the most appropriate.*

A raster dataset can always be resampled to have larger or smaller cells, but this will not improve the level of detail in the dataset. Important factors in choosing cell size are:

- The spatial resolution of the input datasets
- The type of analysis
- The size of the output dataset
- The speed of analysis execution

Generally, a **small cell size** should be used for **large-scale data** with **high spatial accuracy of the feature** (small details are retained), but in this case, visualizing this over a large area can slow down creation time. A **large cell size** is generally selected **for small-scale data, as this will shorten creation time.**

A.3. An introduction to some raster analysis techniques in QGIS

The following tips provide a brief introduction to some of the common raster analysis functions and guidelines for using **the raster calculator tool** to construct more complex expressions. The main difference between ArcGIS and QGIS when working with rasters is that QGIS does not have raster attribute tables associated with entire rasters, so some calculation methods are different.

A.3.1. Standardizing raster data

There are two main methods: 1) Resampling and 2) Aggregation

Method 1: Resampling

There are two tools that can be used, allowing the user to change the cell size and choose the resampling method.

a. r.resample.inter (GRASS)

In this tool, the default resampling method is the **nearest neighbor** method. However, users can choose between three options:

- Nearest Neighbor
- Bilinear
- Bicubic

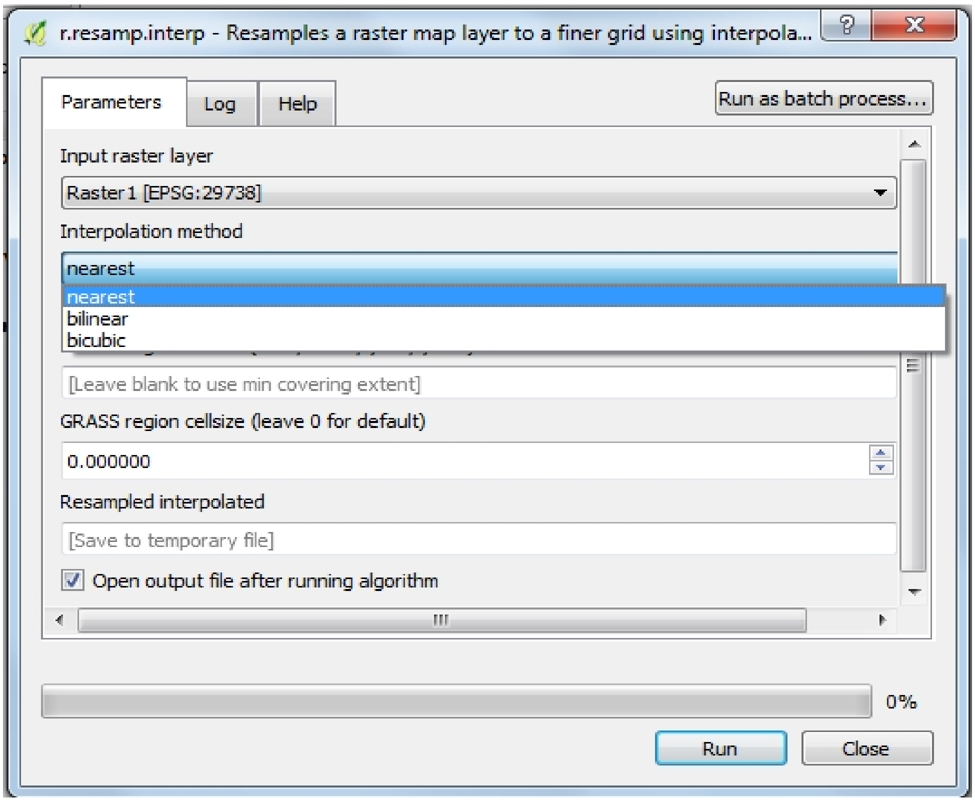

b. Resampling (SAGA)

In this tool, the default resampling method is the **nearest neighbor** method.

However, users can choose from ten options to increase the scale:

- Nearest Neighbor
- Bilinear interpolation
- Inverse distance interpolation
- Bicubic spline interpolation
- B-spline interpolation
- Average value
- Average value (weighted cell area)
- Minimum value
- Maximum value
- Majority

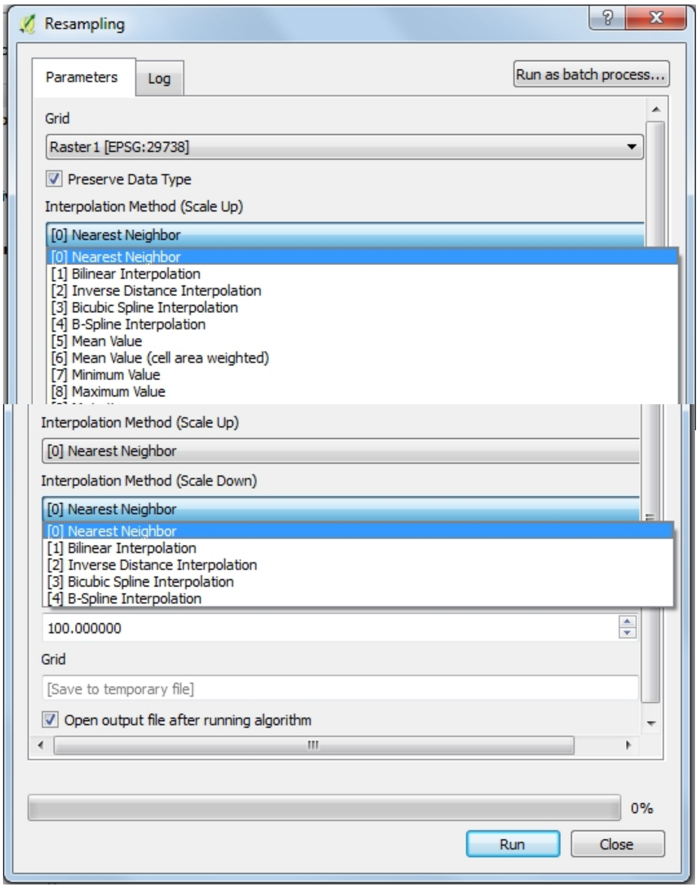

Or five options for reducing the scale:

- Nearest neighbor
- Bilinear interpolation
- Inverse distance interpolation
- Bicubic spline interpolation
- B-spline interpolation

Method 2: Aggregation

Aggregation tools allow the user to choose a cell-related factor to create a version with reduced resolution of a raster dataset. If a user wants to increase the scale of the data from a cell size of 30 m to ~1 km, an **Aggregation Size** of 33 will result in a cell size that is 33 times larger (i.e., 990 m). The cell factor must be an integer greater than 1.

Two tools are available:

c. Aggregate (SAGA)

In this tool, the default aggregation method is the sum method.

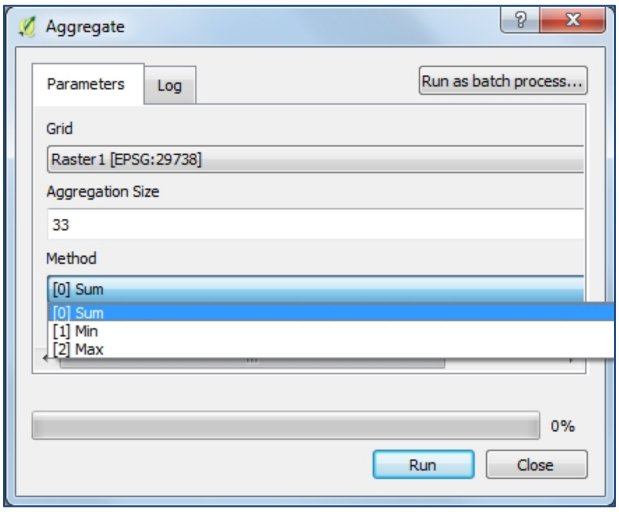

To determine the method for calculating the cell value, the user has three options:

- Sum
- Min
- Max

d. r.resample.stats (GRASS)

The default aggregation method is the **average** method.

To determine the method for calculating the cell value, the user has ten options:

- Average (average)
- Median
- Mode
- Minimum
- Maximum
- Quartile 1
- Quart3
- Perc90
- Sum
- Variance

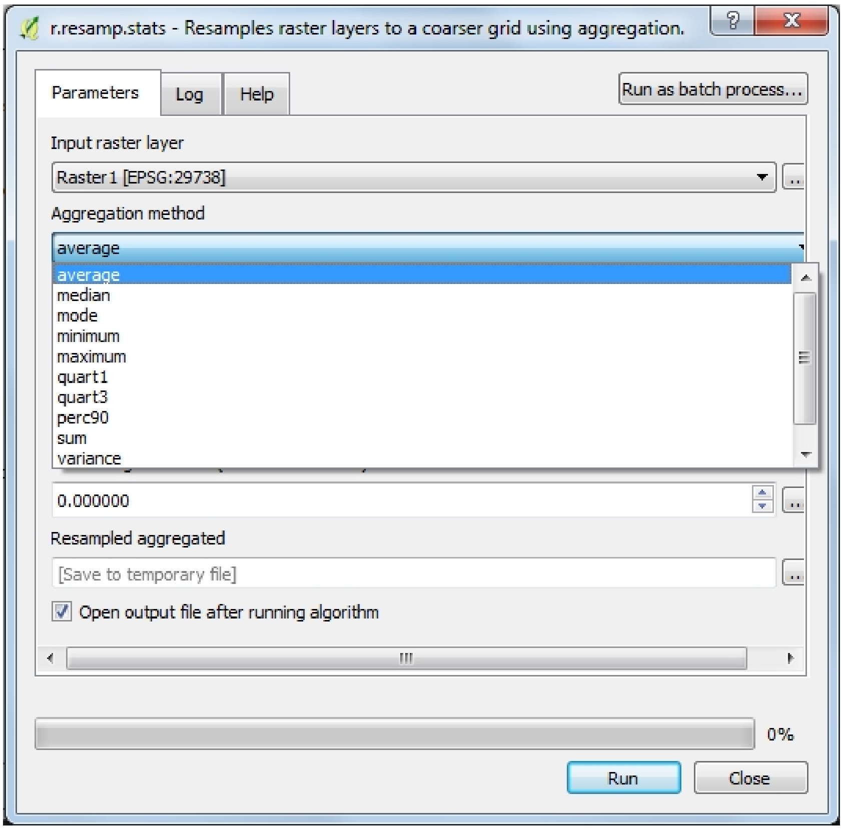

The user specifies an output cell size.

Note that resampling and aggregation offer different methods for determining cell output values. Resampling allows users to decrease or increase the cell size, while aggregation only allows generalization to a larger cell size.

A.3.2. Reclassification functions

Many raster processes use the value field when processing data. If you want to group data or change values in a raster dataset,
of many tools
reclassification are available:

a. r.reclass (GRASS)

The rules for are contained in a txt file (see the tool help for details on how to create a reclassification file).

b. Reclassify values (SAGA)

This tool offers various options for reclassifying from a single value, a range of values, or using a lookup table.
It also provides an option to convert No Data values to another value.

SAGA also offers a simplified version of this tool, which is available in **the Reclassify values (Simple) tool.**

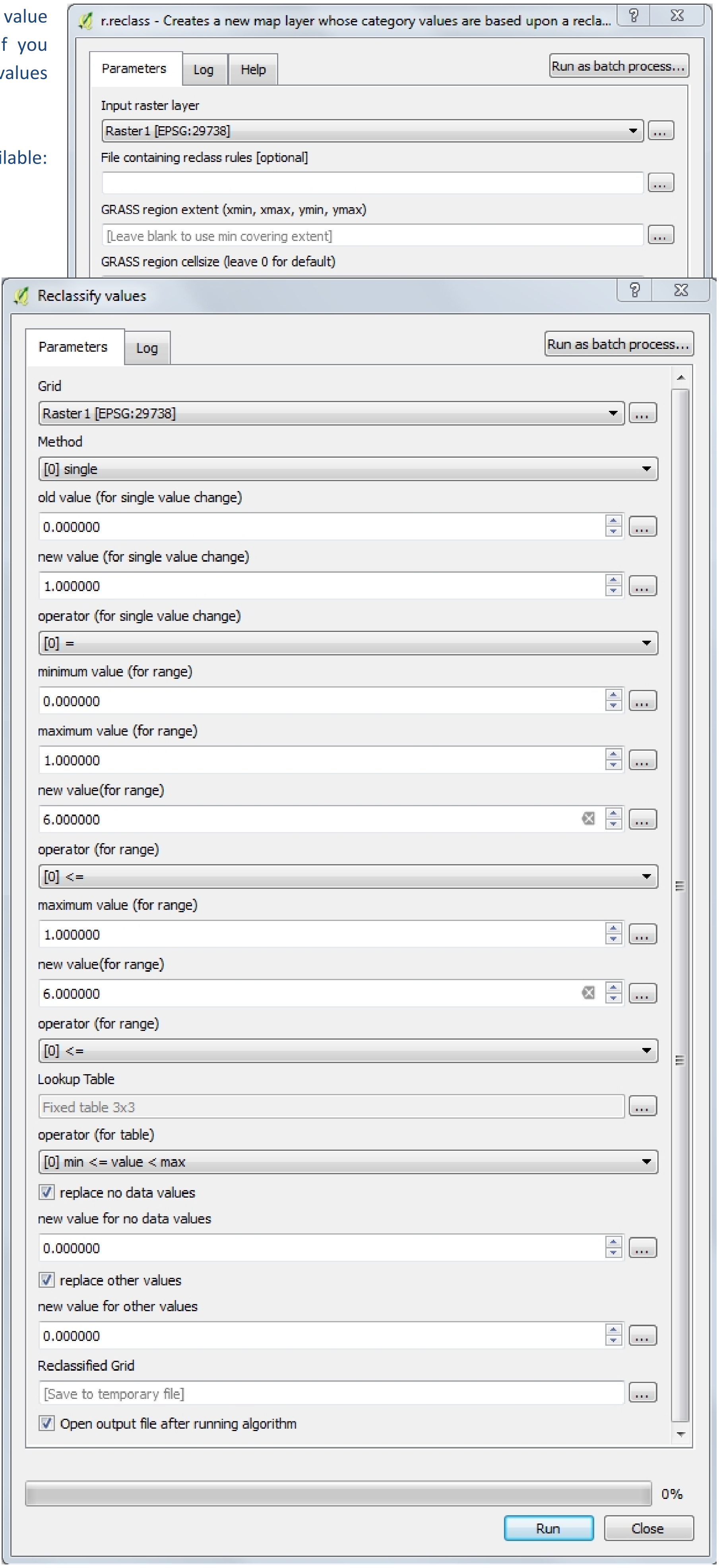

A.3.3. Raster Calculator: Overview

You can also run a tool using **spatial algebra** expressions specified in a **Raster Calculator tool.** Spatial algebra expressions can be used to run a single tool or operators, or to create a more complex chain of tools or expressions. In QGIS, there are three different raster calculators. The main Raster Calculator in QGIS is accessible via the main menu (but it is important to note that it is not accessible via the processing toolbox or the graphical model). The other two are **GRASS r.mapcalculator** and **SAGA Raster Calculator**. We will illustrate the core function of the QGIS raster calculator here. The syntax for the different raster calculators varies; more guidance is available in the QGIS, GRASS, and SAGA documentation.

In the QGIS Raster Calculator, the expression is written at the bottom of the window. In this example, four raster datasets are added together.

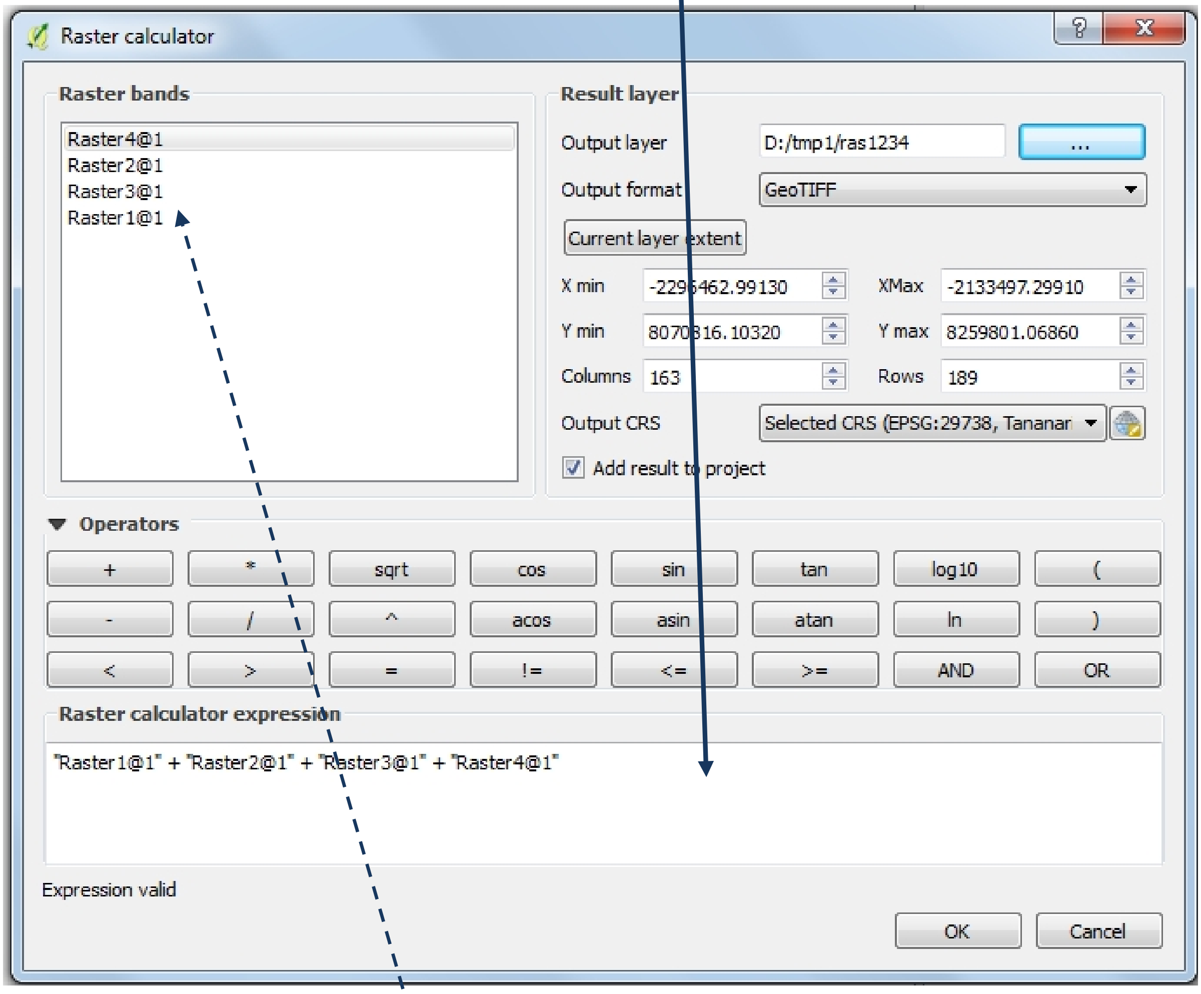

Raster1

2	5	0
8	6	1
5	1	1

Raster2

1	0	1
0	1	0
0	1	1

Raster3

2	1	1
1	1	1
1	3	1

Raster4

2	1	5
0	0	3
3	3	3

=

Outgoing Raster

7	7	7
9	8	5
9	8	6

c. The **left side of the window** shows the **list of layers** that can be used in the expression. These are the raster layers you have added to your ArcMap session. By

Double-clicking on a layer will take you to an expression box enclosed in quotation marks (and an @1 is placed at the end of the name).

d. **The center of the window contains the list of operators. Click once on them to add them to the expression box.** There are simple operators for **Add (+), Subtract (-), Divide (/), or Multiply (*).**
The center of the window also contains operators corresponding to **Boolean logic:**

AND and Boolean	!= Not equal to	>= Equal to or greater than
OR Boolean OR	> Greater than	<= Equal to or less than
= Equal to	< Less than	

A.3.4. Raster calculator: Boolean intersection and No Data conversion

Boolean intersection is the simplest variant of criteria-based processing and is often seen as a thematic mapping tool. Before combining, each input criterion is standardized to a certain scale of adaptability (or reclassified into classes of 1 and 0), i.e., all factors are thus reduced to Boolean raster datasets of suitable and unsuitable areas.

In an example **of Boolean** analysis, if the data for forests contains the value 1, the data for protected areas contains the values 1 and 2, and areas outside protected areas and forests have the value **No data**, the output raster is 1 when forests and protected areas coincide. The remaining areas are classified as **No Data**. The syntax in the raster calculator is:
"nat_for@1" = 1 AND "PA_Raster@1" > 0

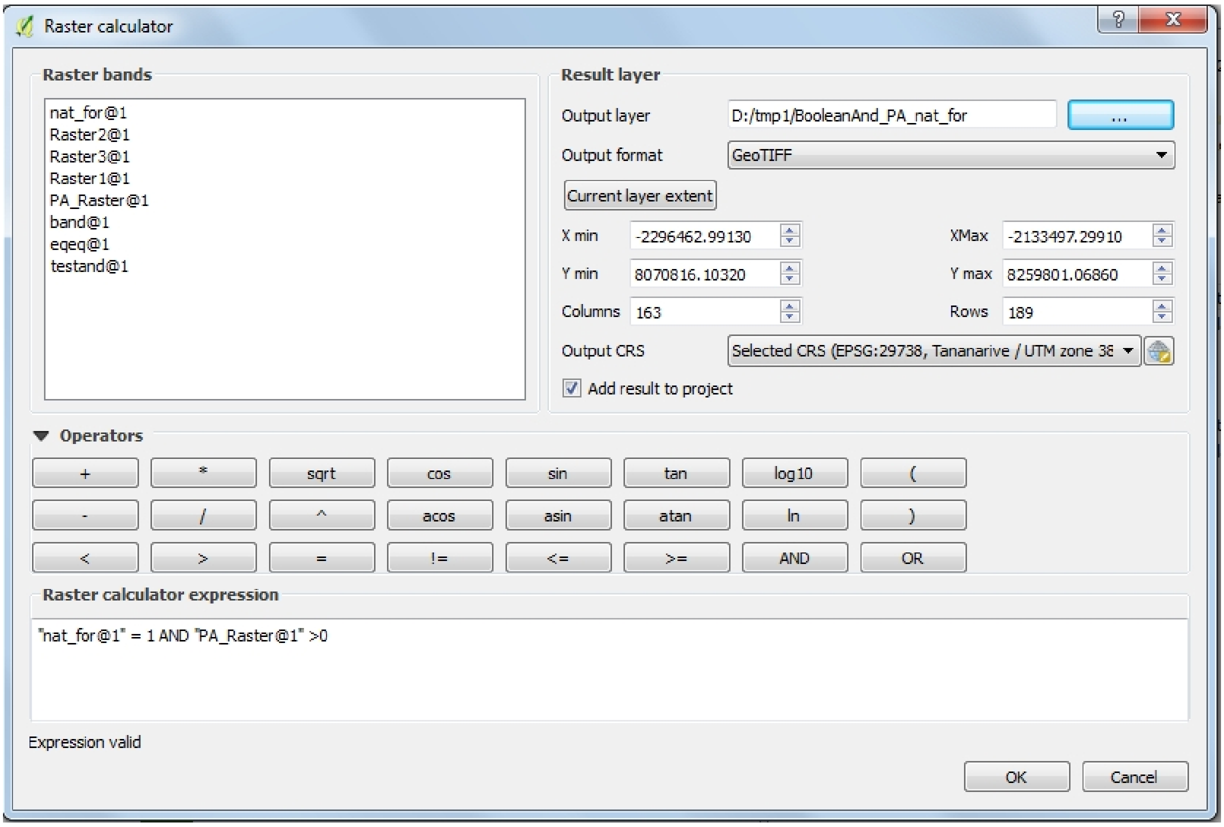

The input datasets are shown on the left and result from the calculation shown on the right:

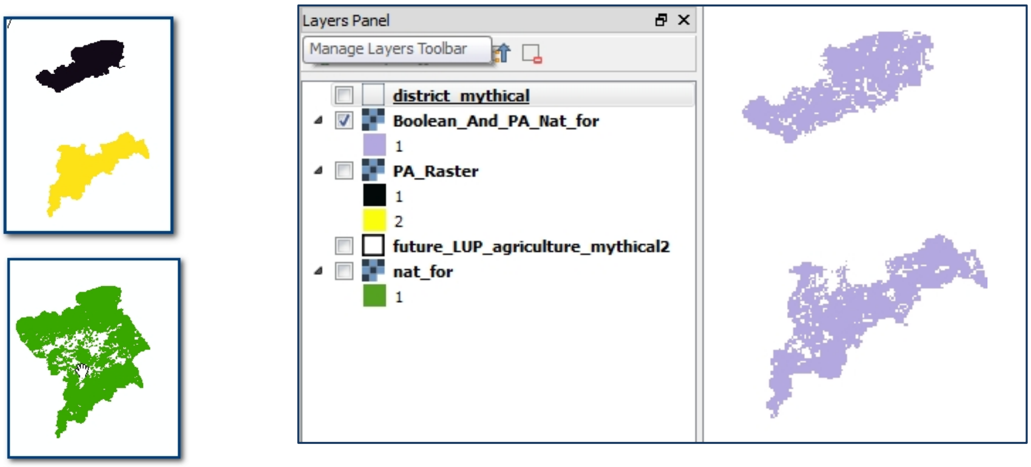

No Data is not a problem with the **Boolean AND** analysis tool since the analysis only selects values that are present in BOTH datasets at the same time. If you use **MOST of the OTHER tools in the logic toolbox,** you will find that **No Data values can cause problems** and that it is necessary to convert them to 0 before using the data (see section 1.4.1 on how to convert No Data values to data).

The **Boolean OR** function selects locations where values are greater than 0 in either of the two datasets. However, if No Data values are present in either dataset, they take precedence over all other values and act as an additional constraint. For example, if a cell has a value of 1 in the forest dataset and a No Data value in the Protected Areas dataset, the output cell will have a No Data value. *** Thus, with **No Data values** present **in the input layers, the output of the Boolean OR** will be **FALSE**!***

The syntax in the raster calculator is: "nat_for@1" = 1 OR "PA_Raster@1" > 0

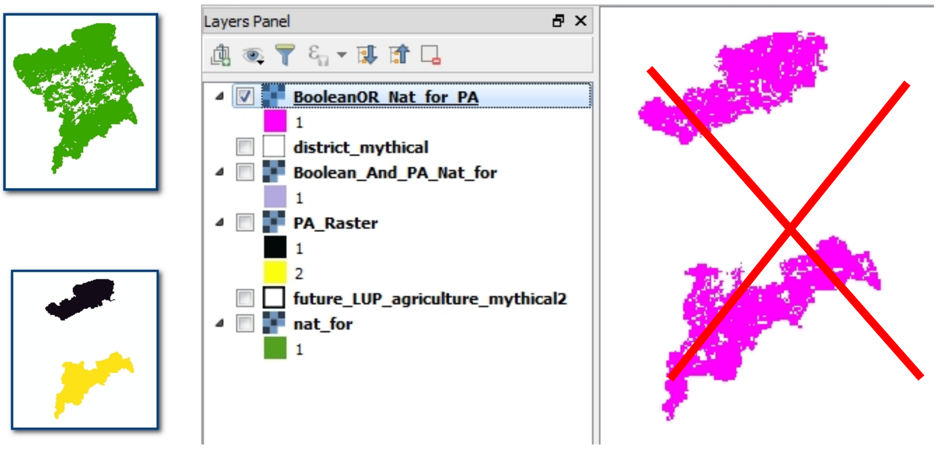

The result should display values of 1 where there is a protected area OR a natural forest. Also, No Data values must be changed to 0.

To convert the No Data values in the Protected Areas raster to 0, we can use the district vector to create a new layer for the district where all values are set to 0 using the **GRASS v.to.rast**

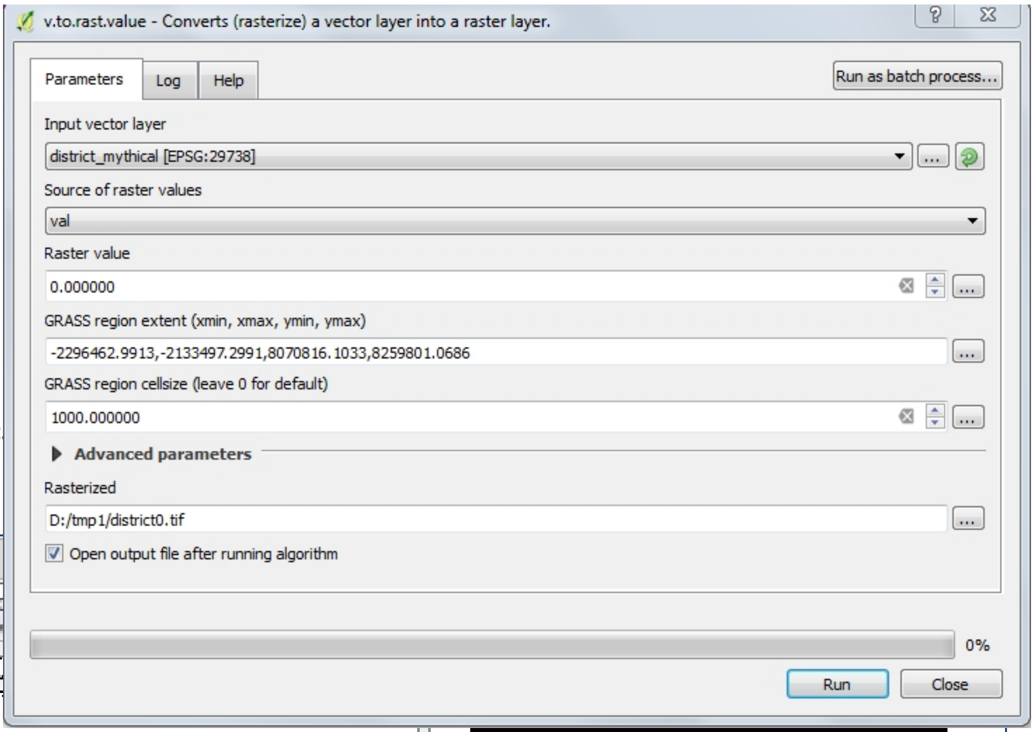

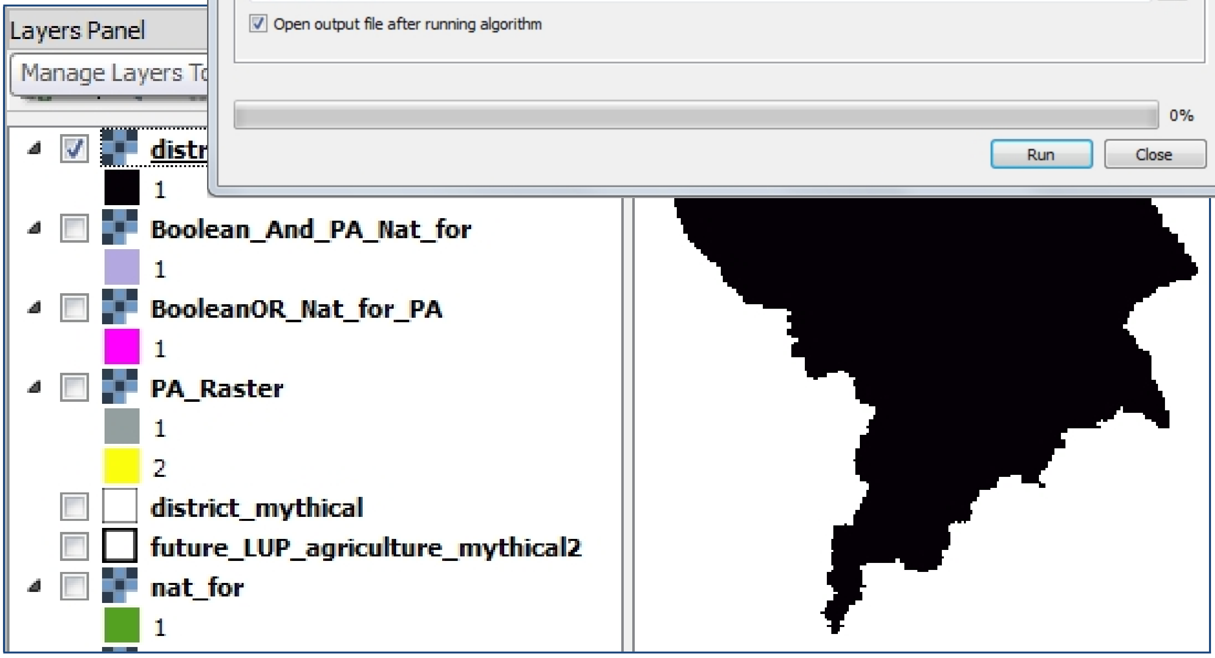

The next step is to merge the districts with the Protected Areas so that areas within the district that are not protected have a value of 0 instead of No Data. To do this, the protected areas layer needs to be moved above the district raster in the table of contents.

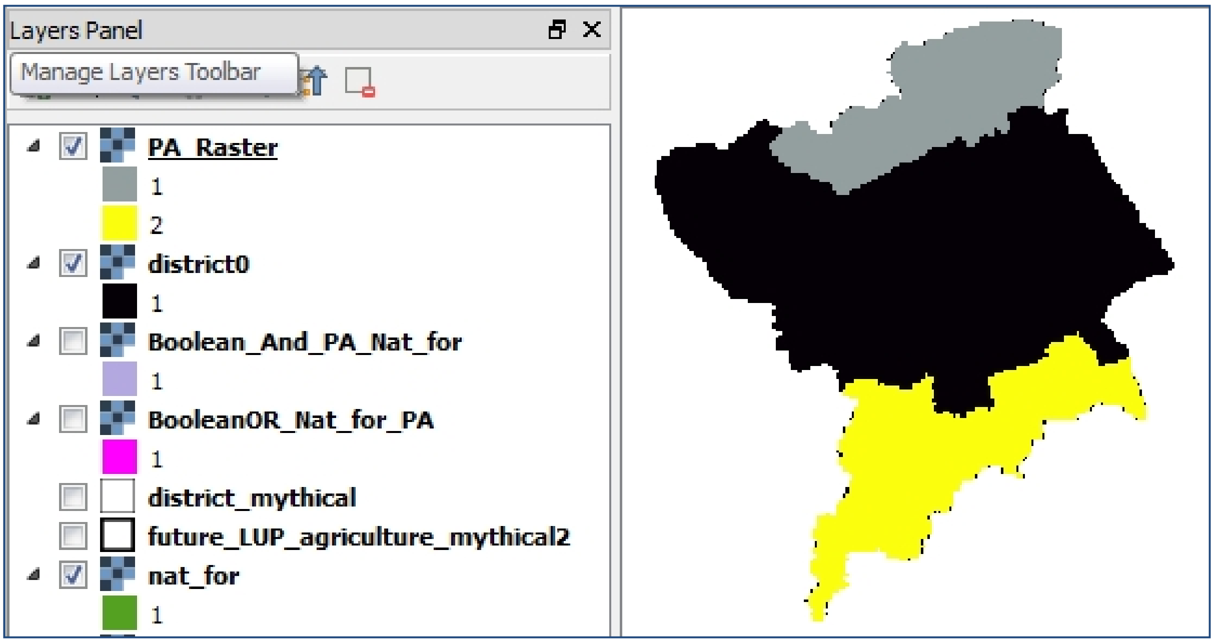

Next, the GRASS **r.patch** tool is used to merge this layer with the protected areas layer. The tool will retrieve the values from the top raster as determining values for the bottom raster.

The raster layers to be merged must be checked and the extent set to the total extent of the area of interest, i.e., in this example, the total extent of the district.

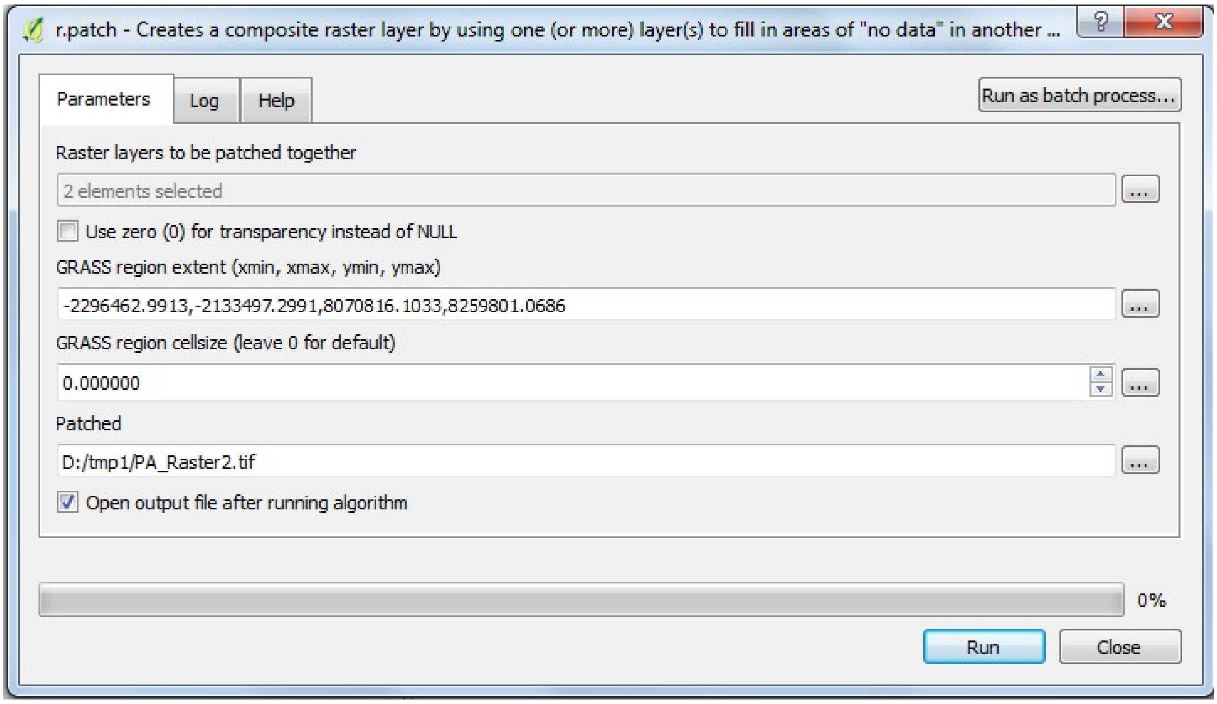

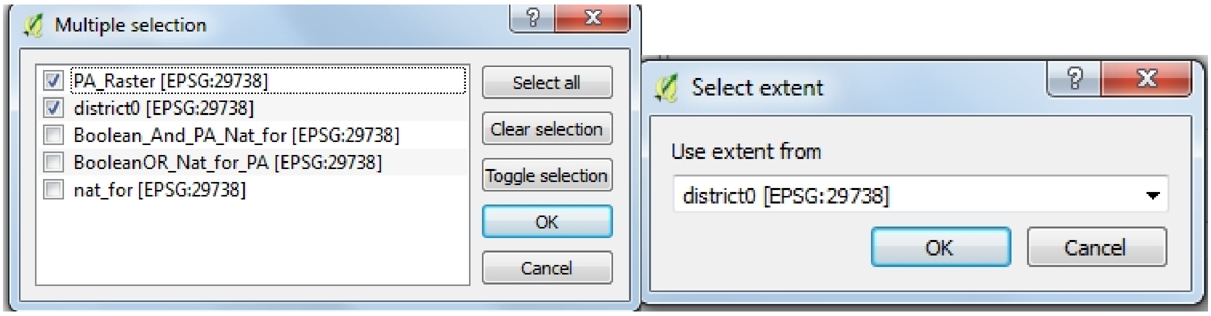

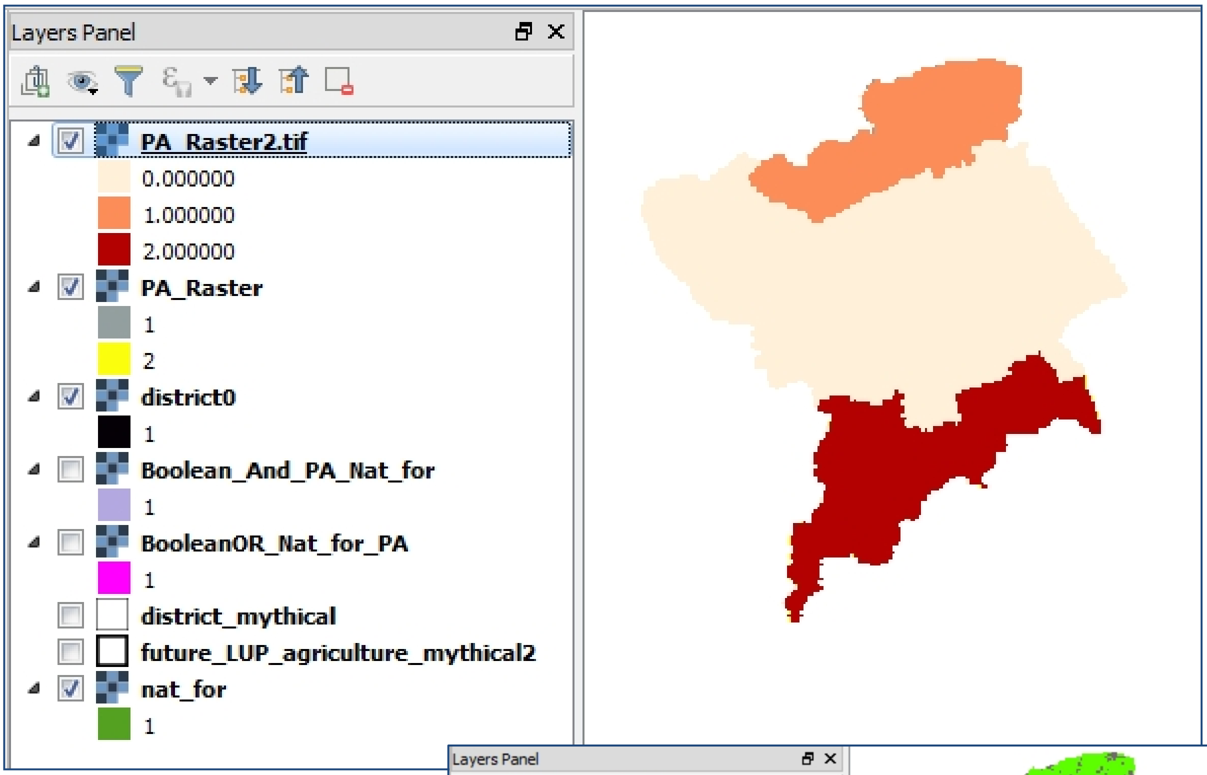

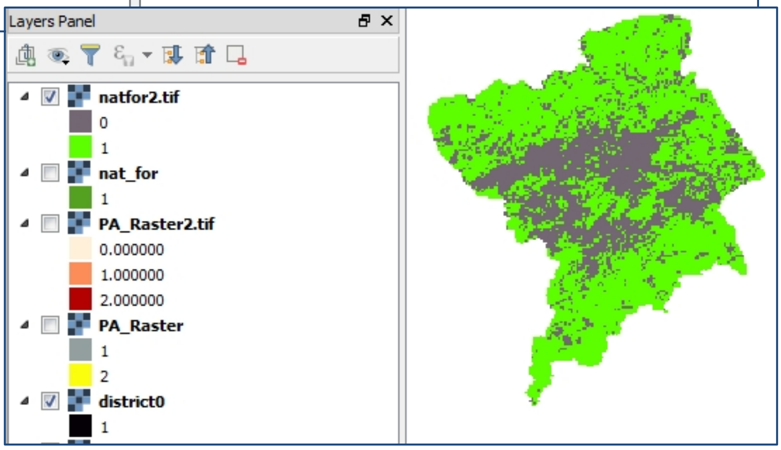

The forest layer must also be moved above the district raster in the table of contents and merged with the district raster using the **r.patch** tool.

Once the layers cover the entire extent and their No Data values have been converted to 0, the Boolean OR expression can be entered into the Raster calculator. The syntax in the raster calculator is:

"nat_for2@1" = 1 OR "PA_Raster2@1" > 0

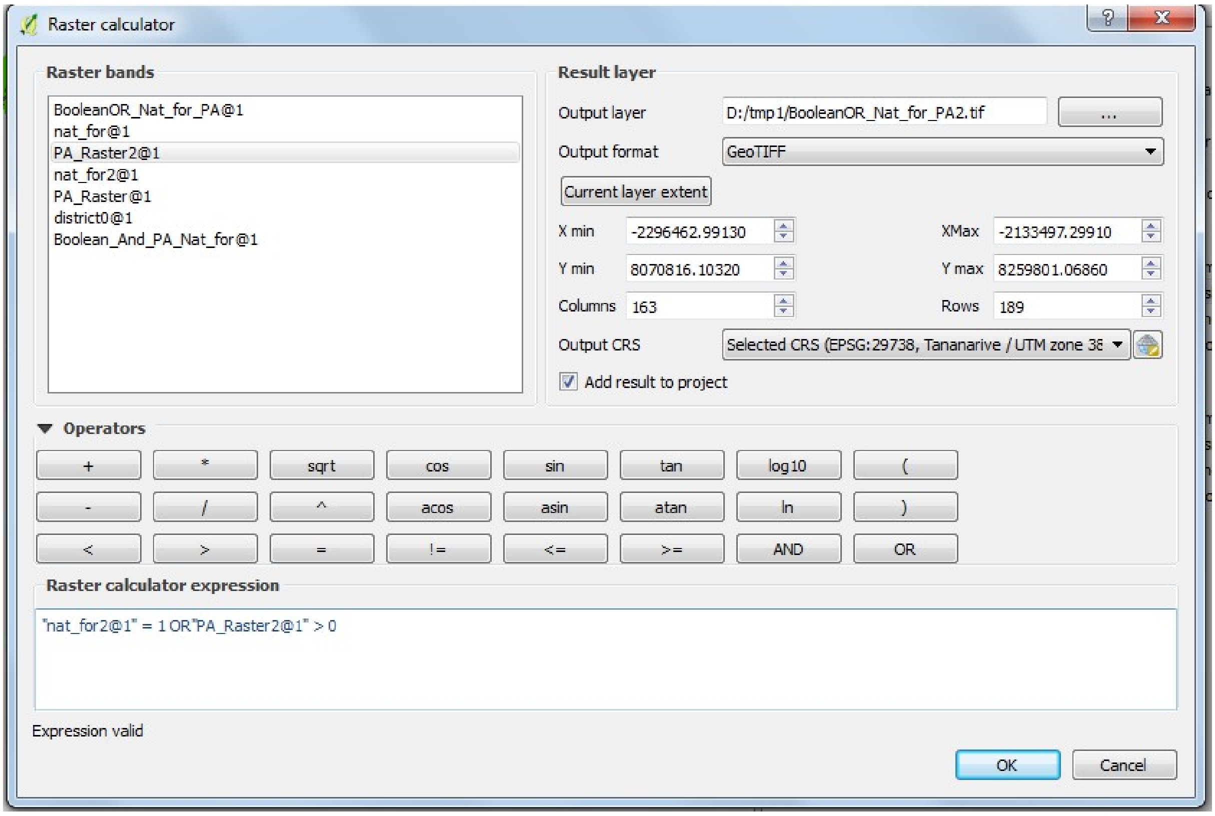

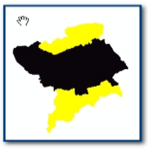

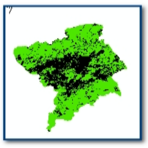

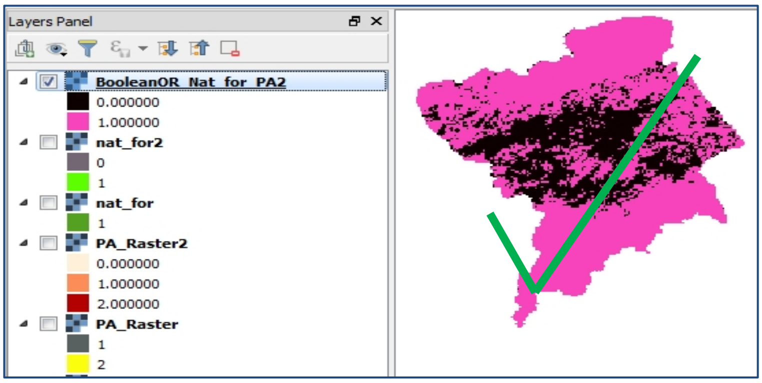

A.3.5. Zonal Statistics

Zonal statistics tools are very useful. They can be used to summarize an input raster data layer into a zone layer. The **Zone** layer can be either vector or raster and can, for example, be used to summarize the number of different forest types in individual protected areas.

This guide only presented a few of the tools in detail in order to help users get started with raster analysis. It should not be considered a comprehensive guide. Users can explore the toolboxes in QGIS themselves to find and identify the relevant tools that will enable them to answer their questions.

Appendix 2: Additional resources for QGIS Graphical Modelers

- Tutorials
- Online examples:
  - http://docs.qgis.org/2.0/ca/docs/user_manual/processing/modeler.html
  - http://www.qgistutorials.com/en/docs/processing_graphical_modeler.html
  - http://gis.stackexchange.com/questions/176599/simplify-repetitive-tasks-in-qgis-graphical-modeler
  - http://www.digital-geography.com/creating-models-qgis/#.WEgN7rKLSM8
  - http://gracilis.carleton.ca/CUOSGwiki/index.php/Automating_Vector_and_Raster_Workflows_using_the_Graphical_Modeler_in_QGIS

DRAFT
