# Supplementary material for: Relevance of the uMap Collaborative Platform as Support for Choropleth Mapping of a Traffic-Light Statistical Signal Atlas of All-Cause Mortality During the First French Lockdown: Geospatial Analysis
Source: JMIR Med Inform. 2026 Aug 27;14:e82855. doi: 10.2196/82855 (PMC13543287; doi:10.2196/82855)
Supplement: Supplementary file 7 [file medinform-v14-e82855-s007.zip › BG_question2_geojson_Fig3b/2_Processing_Fig3b_SMM10000_QGis/tuto/BuildingSpatialWorkflowsToIdentifyPotReddIntervQGIS_fr.pdf]

UTILISER L'INFORMATION GÉOGRAPHIQUE EN SOUTIEN AUX DÉCISIONS EN MATIÈRE DE GARANTIE ET DE BÉNÉFICES MULTIPLES DE LA REDD+

TUTORIEL PAS A PAS VERSION 1.0 : CONSTRUIRE DES FLUX D'OPERATIONS SPATIALES POUR AIDER A IDENTIFIER LES AIRES POTENTIELLES POUR L'ENTREPRISE D'UNE INTERVENTION REDD+ UTILISANT LE MODELEUR GRAPHIQUE DANS QGIS 2.14.X

ET ANNEXE ADDITIONNELLE PRESENTANT DES CONSEILS SUR LES OUTILS ET LES TECHNIQUES DE TRAITEMENT DE DONNEES GEOGRAPHIQUES (GEOPROCESSUS) AVEC QGIS EN COMPARANT LES DONNEES D'ANALYSE RASTER ET VECTORIELLES.

UN-REDD PROGRAMME
Food and Agriculture Organization of the United Nations
UNDP Empowered lives. Resilient nations.
UNEP

Le Programme UN-REDD est une initiative des Nations Unies relative à la Réduction des Émissions dues à la Déforestation et à la Dégradation des forêts (REDD) dans les pays en développement. Le Programme a été lancé en septembre 2008 pour aider ces pays en développement à préparer et mettre en place les stratégies nationales REDD+. Ce programme s'appuie sur le pouvoir de mobilisation et sur l'expertise de l'Organisation des Nations Unies pour l'Alimentation et l'Agriculture (FAO), du Programme des Nations Unies pour le Développement (UNDP) et des Nations Unies pour l'Environnement (UNEP).

Le Centre Mondial de Surveillance de la Conservation des Nations Unies pour l'Environnement (UNEP-WCMC) est le centre spécialiste de l'évaluation de la biodiversité des Nations Unies pour l'Environnement, principale organisation intergouvernementale mondiale en termes d'environnement. Le Centre est opérationnel depuis plus de 35 ans et combine recherches scientifiques et conseils pratiques en matière de politique à adopter.

Élaboré par Corinna Ravilious, Charlotte Hicks et Simon Blyth.

Copyright : 2016 Programme des Nations Unies pour l'Environnement

Autorisation de droits d'auteur : A condition que la source soit mentionnée, cette publication peut être reproduite à des fins éducatives ou non-lucratives sans permission spécifique. La réutilisation de toute figure est sujette à permission des détenteurs originaux des droits d'auteur. Cette publication ne peut pas faire l'objet d'une revente ou de toute autre opération commerciale sans permission écrite des Nations Unies pour l'Environnement. Les demandes de permission, énonçant l'objectif et l'étendue de la reproduction, doivent être envoyées au Directeur à l'adresse suivante : UNEP-WCMC, 219 Huntingdon Road, Cambridge, CB3 0DL, UK.

Clause de non-responsabilité : Le contenu de ce rapport ne reflète pas nécessairement les opinions ou politiques des Nations Unies pour l'Environnement, des organisations et des rédacteurs contributifs. Les désignations employées et les présentations des données dans ce rapport n'impliquent l'expression d'aucune opinion quelle qu'elle soit de la part des Nations Unies pour l'Environnement ou d'organisations, des rédacteurs ou éditeurs contributifs concernant : le statut juridique de tous pays, territoires, zones citadines ou de leurs autorités ou encore la délimitation de leurs frontières, ou limites, ou même de la désignation de leurs noms, frontières ou délimitations. La mention d'entité ou produit commercial dans cette publication n'implique aucun soutien de la part des Nations Unies pour l'Environnement.

Tout commentaire concernant d'éventuelles erreurs ou problématiques est bienvenu. Tout lecteur qui souhaiterait faire un commentaire sur ce document est encouragé à le faire via le lien suivant : ccb@unep-wcmc.org.

Citation : Ravilious, C., Hicks, C. and Blyth, S. (2017) Using spatial information to support decisions on safeguards and multiple benefits for REDD+ (*Utiliser l'information géographique en soutien aux décisions en matière de garantie et de bénéfices multiples pour la REDD+*). Tutoriel pas à pas Version 1.0 : Construire des flux d'opérations spatiales pour aider à identifier les aires potentielles pour l'entreprise d'une intervention REDD+ utilisant le Modeleur Graphique dans QGIS 2.14.x Établi au nom du Programme UN-REDD. Centre Mondial de Surveillance de la Conservation de l'UNEP, Cambridge, UK.

Remerciements : Ces supports de formation ont été réalisés à partir d'éléments développés dans le cadre d'un fonctionnement conjoint avec la Phase II du Programme UN-REDD du Vietnam pour compléter les analyses spatiales, et supportant ainsi la planification de l'action provinciale REDD+ au Vietnam.

UN environment WCMC

Food and Agriculture Organization of the United Nations

UNDP Empowered lives. Resilient nations.

UNEP

Sommaire

1. Introduction ... 7
2. Cartographier des facteurs de déboisement et de dégradation des forêts et des obstacles .. 9
3. Cartographier les interventions REDD+ pour prévenir un facteur ou un obstacle ... 12
4. Définir une logique spatiale et créer un flux de traitements (utilisant un exemple d'intervention REDD+) ... 12
5. Utiliser le Modeleur Graphique QGIS pour mettre en place les flux de traitements ... 15
5.1. Aperçu du Modeleur Graphique ... 15
5.2. Transférer un flux de traitement dans QGIS (en utilisant l'exemple d'intervention REDD+) . 16
5.3. Créer un nouveau Modèle dans la boîte à outils du Traitement ... 17
5.4. Ajouter le flux de traitements au modèle ... 19
5.4.1. Ajouter la première étape à l'outil - Sélectionner la forêt Naturelle dans l'étendue du facteur ... 19
5.4.2. Modifier la première étape ... 23
5.4.3. Documenter la première étape ... 28
5.4.1. Ajouter les étapes restantes du flux de traitements ... 31
5.4.1.1. Distance par rapport aux sites de Sylviculture Communautaire ... 31
5.4.1.2. Distance aux villages ... 34
5.4.1.3. Sélectionner la forêt naturelle dans l'étendue du facteur et au sein de distances spécifiques ... 36
5.4.1.4. Exclure les aires strictement protégées ... 39
5.4.1.5. Identifier les zones prioritaires en fonction de la pauvreté ... 42
5.5. Paramétrer la symbologie pour les sortants du modèle ... 46
5.6. Exécuter le modèle en entier et vérifier les résultats ... 47
5.7. Partager un modèle QGIS avec d'autres utilisateurs ... 53
A Annexe 1 : Notes de conseil sur l'analyse raster et les outils de géotraitement dans ArcGIS et QGIS ... 54
A.1. Vecteur vs raster ... 54
A.1.1. Différence entre les jeux de données vectoriels et raster ... 54
A.1.2. Analyse vectorielle vs raster ... 55
A.2. Analyse raster en QGIS ... 65
A.2.1. Analyse raster et les paramètres d'environnement ... 66
A.2.2. Espaces de travail ... 66
A.2.3. Coordonnées de Sortie ... 67
A.2.4. Taille de la cellule ... 67
A.3. Une introduction à quelques techniques d'analyse raster dans QGIS ... 68
A.3.1. Standardiser des données raster ... 68
A.3.2. Fonctions de reclassification ... 71
A.3.3. Calculatrice raster : Aperçu ... 72

A.3.4. Calculatrice raster : Intersection Booléenne et conversion No Data 73
A.3.5. Statistiques Zonales 78

Annexe 2 : Ressources supplémentaires concernant les Modeleurs graphiques QGIS 79

1. Introduction

REDD+ est une approche volontaire de réduction du changement climatique qui a été développée par les Parties du CCNUCC. Elle vise à inciter les pays en voie de développement à réduire leurs émissions causées par le déboisement et la dégradation des forêts, à conserver les réserves de carbone, à gérer durablement les forêts et à augmenter les réserves de carbone des forêts. Ceci implique des changements dans l'utilisation et la gestion des forêts et pourrait nécessiter diverses et multiples actions différentes comme la protection des forêts contre le feu ou l'abattage illégal ou encore la réhabilitation des zones de forêts dégradées.

La REDD+, au-delà de la question du carbone, peut apporter nombre d'avantages. Par exemple, elle peut promouvoir la conservation de la biodiversité et sécuriser les services écosystémiques forestiers tels que la régulation hydrique, le contrôle de l'érosion et les produits forestiers non-ligneux (PFNL). Quelques-uns des bénéfices potentiels de la REDD+, tels que la conservation de la biodiversité, peuvent être amplifiés en identifiant, grâce à l'utilisation d'analyses spatiales et autres approches, des zones au sein desquelles les actions de REDD+ auraient le meilleur impact.

Le but de cette série de tutoriels est de guider, lors des sessions de travail technique, les participants déjà compétents en SIG dans la réalisation d'analyses qui sont liées à REDD+. Les tutoriels ont été utilisés dans de nombreux pays pour renforcer les capacités à produire des ensembles de données et de cartes pertinentes avec leur planification spatiale pour REDD+ et pour développer d'autres types de cartes similaires. Les cartes obtenues en utilisant ces approches apparaissent dans de nombreuses publications qui visent à soutenir la planification d'options stratégiques améliorant les services concernant biodiversité et les services écosystémiques ainsi qu'à réduire le changement climatique (voir http://bit.ly/mbs-redd pour les ressources du pays concerné). Bien entendu, les pays ne sont pas obligés d'utiliser les approches décrites dans ces tutoriels.

L'analyse spatiale peut être utilisée pour soutenir la prise de décision notamment en vue d'identifier les aires potentiellement appropriées aux interventions REDD+ (actions REDD+), toutefois les cartes seules ne sont pas suffisantes pour prendre ces décisions. Ceci ne signifie pas qu'il soit utile de cartographier toutes les interventions potentielles ; cependant, si elle est réalisée parallèlement à des méthodes telles que la consultation des parties prenantes et des approches de cartographie participative, l'analyse de la carte peut alors être utile pour la sélection des approches REDD+ et des lieux où les appliquer.

Ce tutoriel se focalise sur la façon d'utiliser les flux de traitements d'analyse spatiale dans l'identification d'aires potentielles au niveau desquelles une intervention REDD+ (notamment appelée action REDD+, ou mesure REDD+) pourrait être réalisée pour répondre à un obstacle ou un facteur spécifique. Certaines intervention seront plus adaptées à certains facteurs et ensembles d'obstacles spécifiques.

Ce tutoriel utilise une imagerie de surfaces terrestres pour expliquer comment créer et faire fonctionner une analyse utilisant des flux de traitements (workflows) dans le Modeleur Graphique de QGIS. Il traite : la cartographie des facteurs de déboisement et de dégradation de la forêt et des obstacles aux activités « + » ; la cartographie des interventions REDD+ ; la définition d'une logique spatiale et la création de flux de traitement ; le partage du modèle obtenu ; et la réparation d'un

modèle dysfonctionnant. Les annexes fournissent davantage d'informations sur les formats et le traitement des données raster pour les personnes qui sont habituées aux données SIG vectorielles. Elles guideront également les utilisateurs dans l'exploration des différents outils et méthodes d'analyse raster qui sont disponibles dans la boîte à outils de géotraitement de QGIS.

L'annexe 1 fournit un bref résumé des différences entre les données raster et vectorielles ainsi qu'une introduction pour commencer l'analyse de données raster. L'analyse raster offre aux utilisateurs un accès à de nombreux outils et fonctions qui sont disponibles pour l'analyse vectorielle. Nombre de ces outils sont particulièrement adaptés aux analyses multicritères. Les fonctions raster peuvent également être utilisées pour la génération de nouvelles données (ex. données sur l'hydrologie et les pentes depuis les modèles numériques de terrains). Elle comprend également un tableau de comparaison montrant les outils ArcGIS équivalents.

L'annexe 2 fournit des liens vers des ressources et des aides supplémentaires liées au Modeleur Graphique QGIS.

Un tutoriel similaire qui utilise ArcGIS comme plateforme alternative est disponible depuis http://bit.ly/GIStools-redd :
Ravilious, C., Hicks, C. and Blyth, S. (2016) Using spatial information to support decisions on safeguards and multiple benefits for REDD+ (*Utiliser l'information géographique en soutien aux décisions en matière de garantie et de bénéfices multiples pour la REDD+*). Tutoriel pas à pas Version 1.0 : Construire des flux d'opérations spatiales pour aider à identifier les aires potentielles pour l'entreprise d'une intervention REDD+ utilisant un générateur de modèle dans ArcGIS 10.x. Établi au nom du Programme UN-REDD. Centre Mondial de Surveillance de la Conservation de l'UNEP, Cambridge, UK.

2. Cartographier des facteurs de déboisement et de dégradation des forêts et des obstacles

Lorsqu'on pense aux facteurs de déboisement et de dégradation des forêts et aux obstacles aux activités « plus » (conservation, amélioration, gestion durable), nous nous référons souvent à la situation actuelle (ex. quels sont les facteurs actuels et quelles sont les zones où des changements dans le couvert arbustif sont déjà observés). Mais il est également important de penser aux obstacles futurs ainsi qu'aux futures pressions et menaces pesant sur la forêt et à la façon dont elles pourraient mener au déboisement ou à sa dégradation de la forêt.

Pour identifier les lieux des interventions REDD+, il est important de prendre en compte à la fois la localisation des obstacles et facteurs actuels et l'existence de pressions et menaces pesant sur la forêt en vue d'identifier les lieux où pourraient être rencontrés, à l'avenir, facteurs et obstacles.

Les facteurs et obstacles peuvent être directs ou indirects. Des exemples de **facteurs directs** comprennent le développement des infrastructures, le développement de l'agriculture, les incendies, les activités minières et le développement des plantations (ex. caoutchouc, huile de palme). Un obstacle direct peut être un élément physique (pentes raides, terrain caillouteux, distance aux routes) qui complique le reboisement. Des exemples **de facteurs et d'obstacles indirects** comprennent : les changements dans l'importance de la population (indiquant éventuellement une augmentation de la demande en terre et en ressources naturelles), les niveaux de pauvreté (reflétant éventuellement une dépendance directe aux ressources naturelles), les motivations financières (ex. prix des marchandises et les subventions qui pourraient rendre certaines utilisations des terres plus attractives), les préférences culturelles (définissant peut-être l'utilisation des ressources naturelles) et les décisions politiques (déterminant la distribution et le contrôle de l'utilisation des terres).

Les facteurs de changement dans le couvert arbustif/ la qualité de la forêt pourraient entraîner des modifications des fonctions forestières, des niveaux de biodiversité, des provisions de services écosystémiques et des soutiens pour les moyens de subsistance forestiers.

Pour identifier et cartographier les zones affectées ou risquant d'être affectées par les facteurs et obstacles, nous pouvons :

- Utiliser une approche participative, impliquant multiples parties prenantes de différents secteurs, des organisations et communautés compétentes localement pour identifier les facteurs, les obstacles et les aires affectées ou à risque.
- Cartographier les pressions directes et indirectes sur la forêt, examiner leur relation avec le couvert arbustif et le changement de ce couvert arbustif et analyser leurs possibilités d’évolution dans l’avenir.
- Cartographier les zones qui sont susceptibles d'être affectées par des facteurs ou des obstacles particuliers dans le futur (ex. déboisement planifié en raison des propositions de développement d'infrastructures ou changements de destination pour l'utilisation des terres).
- Cartographier les zones actuelles où les activités de conservation, d'amélioration et de gestion durable sont déjà réalisées et examiner la relation entre les facteurs, qui soit entravent, soit favorisent leur succès.

- Cartographier les zones présentant un potentiel physique, environnemental et socio-économique pour la promotion d'activités « plus ».

Des cartes montrant les couches individuelles de pressions/ menaces futures et des cartes utilisant des recouvrements simples de données peuvent être créées pour présenter les lieux où les facteurs coïncident. Ceci ne « sélectionne » pas des zones particulières, mais ces cartes peuvent apporter une contribution importante pour les ateliers de travail des parties prenantes où les participants identifient les zones adaptées aux interventions REDD+ en se basant sur leur connaissance locale avancée.

Des cartes montrant des zones remplissant certains critères peuvent également être créées. Par exemple, une carte montrant les zones à risque de développement agricole à petite échelle pourrait mettre en lumière les zones basées sur la présence d'agriculture existante, les zones désignées pour l'agriculture future au travers des plans d'utilisation des terres, et les facteurs économiques tels que la pauvreté et la population.

Cette approche peut s'avérer plus subjective en termes de critères et de seuils utilisés et nécessite d'être validée par des experts aux connaissances locales. Si les connaissances nécessaires pour élaborer le flux de traitements de la carte sont pauvres ou si le flux de traitements n'est pas correctement validé, il peut mener à une utilisation de données erronées. Les hypothèses doivent être présentées de manière transparente afin que les planificateurs et les parties prenantes comprennent les données qui sont utilisées pour créer la carte et la façon dont elles influencent les zones qui ont été sélectionnées par le processus d'analyse spatiale. On peut faire ceci en indiquant les couches entrantes ou les hypothèses dans une série de cartes mises en annexe ; la méthode de sélection des zones, les couches entrantes utilisées et les critères appliqués sont ainsi plus clairs. Sinon, les cartes entrantes peuvent être présentées à côté du produit final, comme dans l'exemple de carte ci-dessous où les couches entrantes sont présentées à droite de la carte finale.

This map shows areas where forests are particularly important for limiting soil erosion that might cause sedimentation problems for dams in Tanzania. The methodology is based on four parameters: slope, precipitation, locations of dams and water bodies and their catchments, and forest.

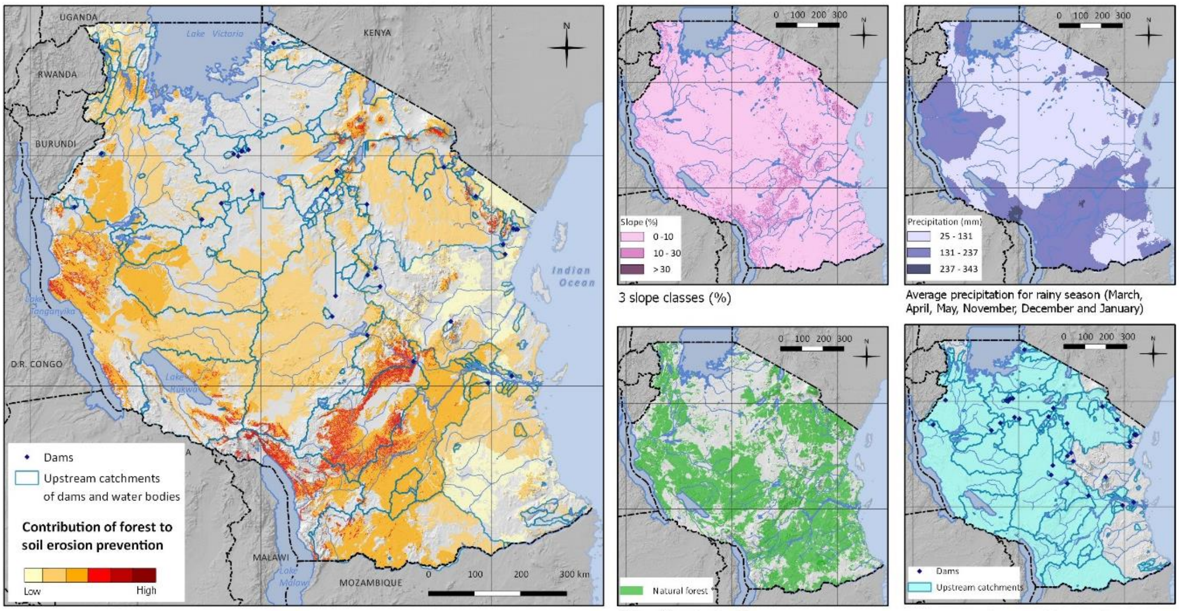

3 slope classes (%)

Average precipitation for rainy season (March, April, May, November, December and January)

Natural forest extent*

* Defined according to the national forest definition to include the following land cover classes: montane and lowland forest, open and closed woodland, mangroves, thickets and bushlands.

Upstream catchments of dams and water bodies

Map projection: WGS84 / UTM Zone 36S. Map prepared by Tanzanian Forest Service (TFS), UNEP-WCMC, FAO, Sokoine University of Agriculture (SUA) and Forestry Training Institute (FTI). Date: May 2013.

Data Sources:
Natural forest and water bodies: NAFORMA. 2013. NAFORMA land-use / land-cover Map 2010.
Dams: Dr. Mark Mulligan, Department of Geography, Kings College, London.
Slope: generated from Lehner, B., Verdin, K., Jarvis, A. 2008: New global hydrography derived from spaceborne elevation data. Eos, Transactions American Geophysical Union, 89 (10) (2008) 93-94. Journal Article.
Precipitation: Hijmans, R.J., S.E. Cameron, J.L. Parra, P.G. Jones and A. 2005. Jarvis. Very high resolution interpolated climate surfaces for global land areas. International Journal of Climatology 25 (2005): 1965-1978. Journal Article.

3. Cartographier les interventions REDD+ pour prévenir un facteur ou un obstacle

Les deux approches d'analyse, participative et spatiale, pourraient jouer un rôle dans la cartographie des zones prioritaires pour la mise en place d'interventions REDD+. Pendant le processus de développement du Plan d'Action Provincial REDD+ (PRAP) au Vietnam, par exemple, les deux approches ont été utilisées, les participants à l'atelier de travail indiquant dans un premier temps les zones d'interventions particulières et les équipes d'analyse spatiale réalisant les analyses SIG plus poussées.

Pour l'approche d'analyse spatiale, deux étapes principales sont nécessaires :

1. **Définir la logique spatiale ou un flux de traitements pour répondre à une question spécifique :** Le flux de traitement précisera quels jeux de données et quels outils de géotraitement sont nécessaires pour produire la carte finale désirée. Ce flux de traitements doit aussi prendre en compte a) la zone affectée par/ risquant d'être affectée par le facteur/ obstacle et b) les résultats de la cartographie participative des interventions.
2. **Exécuter le flux de traitements dans le Modeleur Graphique QGIS.** En exécutant les étapes de géotraitement dans un seul outil, l'analyse peut être réalisée en moins d'étapes, voire une seule étape.

4. Définir une logique spatiale et créer un flux de traitements (utilisant un exemple d'intervention REDD+)

Définir **les flux de traitements** vous permet de réfléchir à **la façon dont vous réaliserez une analyse** : la logique spatiale, les processus techniques SIG et la séquence d'étapes.

Cette section du tutoriel se focalise sur la réflexion et la préparation nécessaires avant la construction d'un flux de traitements dans le Modeleur Graphique QGIS. Pour aider à l'identification des localisations potentielles à une intervention REDD+ particulière, un flux de traitements combinerait les facteurs dans une analyse multicritère, afin **d'inclure** les zones **adaptées** à l'intervention et **d'exclure** celles qui ne le sont **pas**.

Réfléchir aux questions ci-dessous peut aider à clarifier les couches entrantes, les autres données et les processus dont vous aurez besoin dans votre flux de traitements :

- Où sont situées les zones affectées/ risquant d'être affectées par les facteurs ou les obstacles ? La localisation des interventions doit être choisie en fonction de la localisation des facteurs/ obstacles ; elle peut se baser sur des cartes précédentes développées pour montrer les facteurs/ obstacles ou sur d'autres connaissances permettant de déterminer les zones affectées. Ceci pourrait être une large partie de la zone terrestre, par exemple, toute la forêt de la province pourrait être affectée par un obstacle tel qu'un manque de ressources de suivi.
- Quels aspects physiques affecteront la mise en place de l'intervention ? Par exemple, la pente, le type de sol, le type de forêt, le climat.

- Quels autres aspects pourraient affecter sa faisabilité ? Par exemple, l'accessibilité des sites, les réserves de carbone, la condition de la forêt ou les risques pouvant nuire au succès de l'intervention (ex. risque d'incendies).
- Quel est le potentiel d'augmentation des bénéfices de l'intervention ? Par exemple, peut-elle contribuer à la diminution de la pauvreté, à la conservation de la biodiversité ou à l'offre de services écosystémiques ?
- Quels risques sociaux et environnementaux sont associés à l'intervention ? Quel est le potentiel de réduction des risques et de soutien à la sauvegarde : par exemple, prévenir la conversion des forêts naturelles ou réduire le risque de déplacement (c.à.d. réduire le risque de déplacement du déboisement ou de la dégradation vers d'autres zones) ?

Dans l'exemple du Vietnam, des informations bien plus pertinentes auraient dû être collectées pendant le processus PRAP. Ainsi, des analyses précédentes et la cartographie participative des facteurs et obstacles peuvent permettre d'identifier la zone présentant le facteur/ obstacle. Parallèlement, les risques sociaux et environnementaux et les évaluations des bénéfices devraient faciliter l'identification des risques et bénéfices potentiellement associés à chaque intervention.

L'identification des zones adaptées pour les interventions spécifiques peut apporter un complément d’information et, avec l’engagement des parties prenantes, être utilisée avec d’autres méthodes participatives. Par exemple, si une approche de cartographie participative a été réalisée pour définir les zones potentielles, est-il nécessaire de réaliser des analyses plus poussées ? Ceci peut varier d'une intervention à l'autre. Les cartes participatives peuvent donner une idée d'ensemble, elles permettent par exemple d'identifier les zones à un niveau administratif (ex. : la commune) et d'apporter ainsi quelques critères qui pourraient offrir, si nécessaire, une analyse spatiale plus détaillée pour compléter les résultats participatifs.

En définissant la logique spatiale à utiliser pour le flux de traitements, il est souvent utile de penser dans un premier temps aux endroits où **l'intervention REDD+ ne peut pas être réalisée,** afin d'**exclure ces zones** du possible champ d'intervention REDD+. Cette démarche permet d'identifier plus facilement dans les aires restantes les endroits où l'intervention REDD+ **peut être réalisée**. Il est souvent plus facile de faire ceci avant d'identifier **les endroits où l'intervention REDD+ peut être réalisée** dans les aires restantes**.**

Lorsque vous réfléchissez à l'analyse spatiale à réaliser, vous avez besoin d'identifier les critères et les données que vous utiliserez pour faire ces exclusions/inclusions, les outils de géotraitement que vous pouvez utiliser dans QGIS et la disponibilité des données (notamment à la bonne échelle).

Il est important de documenter les raisons pour lesquelles vous excluez ou incluez certaines zones et de préciser quels facteurs ont été utilisés pour déterminer ces zones. Ceci assurera une présentation claire et transparente de l'analyse aux décideurs pour qu'ils puissent comprendre la façon dont une carte a été créée et les hypothèses qui ont guidé sa réalisation.

Dans ce tutoriel, un exemple de flux de traitements sera exposé pour le développement d'une couche d'intervention REDD+ : celle-ci sera destinée à **la sylviculture communautaire durable pour contrer le facteur « conversion à petite échelle de la forêt vers le manioc »**, en se basant sur le fait que les forêts précieuses pour la communauté risquent moins d'être converties en terres agricoles.

Pour définir un flux de traitement répondant à cette question, nous pouvons considérer les questions suivantes :

- Où sont les zones à risque de développement du manioc à petite échelle (maintenant et dans le futur) ?
- Quels sont les endroits où une sylviculture communautaire durable peut se faire ? Dans nos exemples, les zones à proximité des villages sont considérées comme plus adaptées.
- Quels types de désignation des zones forestière devraient être inclus ? Dans notre exemple, les aires strictement protégées ne sont pas disponibles pour la sylviculture communautaire.
- L'intervention devrait-elle se faire dans les forêts naturelles et/ ou les forêts plantées ? Dans notre exemple, la forêt naturelle est préférable.
- L'intervention doit-elle être limitée aux zones sylvicoles communautaires ou peut-on voir plus large ? Dans notre exemple, les zones à proximité des forêts communautaires existantes ou planifiées sont préférées.
- Quels bénéfices et quels risques sont associés à l'intervention ? Peuvent-ils être cartographiés ? Dans notre exemple, la réduction de la pauvreté était le seul bénéfice supplémentaire considéré.

L'analyse SIG **exclurait** par la suite les zones non exposées au facteur et les zones où il n'est pas possible de réaliser une action particulière et **inclurait** les zones considérées adaptées et où les bénéfices pourraient être augmentés et les risques réduits. Le tableau présente les couches de contribution pour un exemple de flux de traitements :

**Type de données/ couches de contribution**	**Comment les utiliser ?**	**Nom du jeu de données dans le dossier Mythical _Database**
Couvert arbustif	Zone de forêt naturelle disponible pour les actions	Natural_forest_mythical
Localisations des villages	Les zones à proximité des villages avec une demande probable en production de manioc et/ ou en activités sylvicoles communautaires (SC).	Village_mythical
Forêts communautaires existantes et potentielles	Catégorie de forêt ; zones déjà identifiées comme des sites SC actuels ou potentiels.	Community_forestry_mythical
Aires protégées	Exclut les aires strictement protégées	PA_mythical
étendue du facteur	Zone affectée par/ risquant d'être affectée par un facteur (conversion vers le manioc) *(dans cet exemple un Plan d'Utilisation des Terres futur a été utilisé pour identifier les larges zones destinées pour l'agriculture)*	future_LUP_agriculture_mythical
Potentiel de réduction de la pauvreté (comme bénéfice de l'intervention)	Zones présentant des taux de pauvreté moyens-élevés	Communes_mythical (utilisant le champ pov_hml, valeurs : (1) faible (2) moyen et (3) élevé

Le flux de traitements peut être fait manuellement dans un organigramme avant d'être exécuté dans QGIS. Dans cet exemple, nous utiliserons un flux de traitement conçu pour ArcGIS pour montrer comment des outils et analyses équivalents peuvent être trouvés et pour adapter et mettre en œuvre le flux de traitements dans QGIS. L'exemple relatif à l'intervention ci-dessus est présenté ci-après.

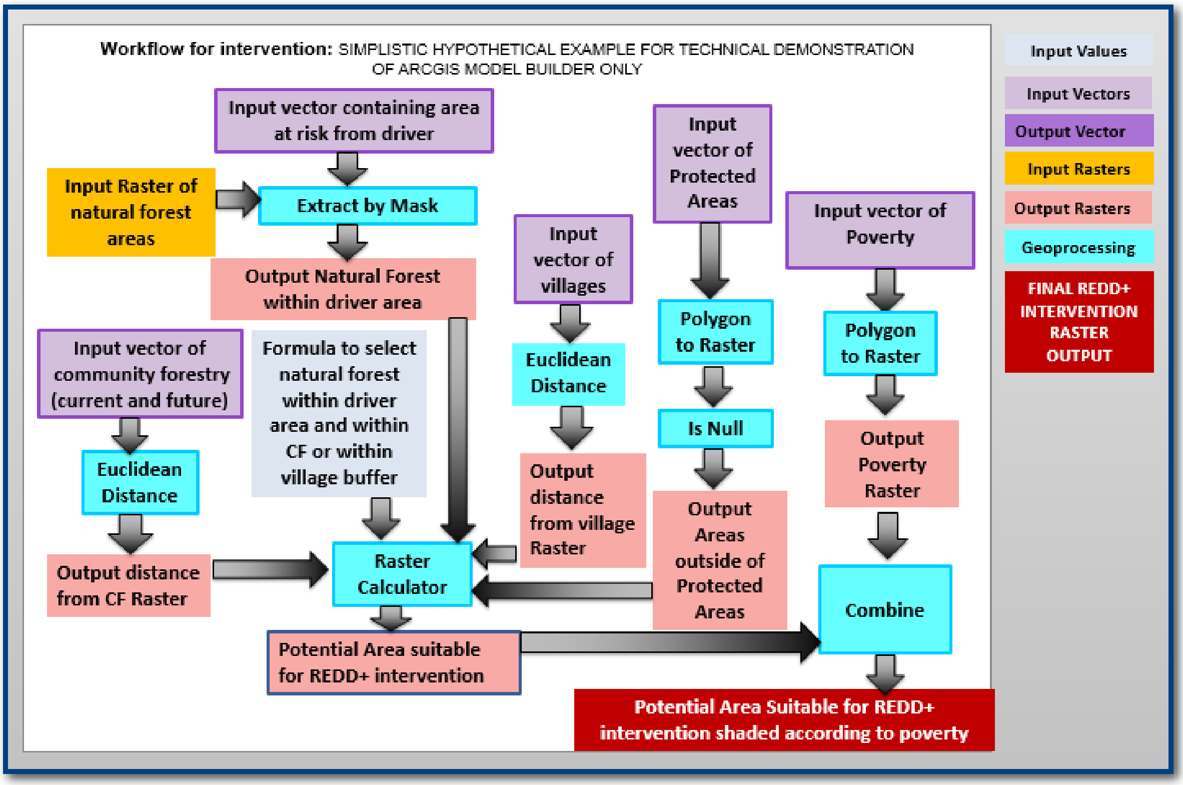

5. Utiliser le Modeleur Graphique QGIS pour mettre en place les flux de traitements

5.1. Aperçu du Modeleur Graphique

Le Modeleur Graphique est une application que vous pouvez utiliser pour **créer, publier** et **gérer les modèles**. C'est l'équivalent du Générateur de Modèle ArcGIS. Les Modèles dans QGIS sont des flux de traitements qui rassemblent des séries d'outils de géotraitement, transférant le résultat d'un outil dans l'outil suivant. Les bénéfices de l'utilisation du Modeleur Graphique résident dans une meilleure organisation permettant à la fois une amélioration du flux de données et une plus grande rapidité d'exécution de l'analyse. En normalisant ainsi les processus, toute répétition d'une analyse sera éminemment facilitée, permettant une réplication et une comparaison directes des résultats.

Un modèle simple ne prendrait en compte qu'une ou deux étapes ou un ou deux outils de géotraitement QGIS. Par exemple, le modèle ci-dessous classifie la forêt en fonction de sa distance par rapport aux routes :

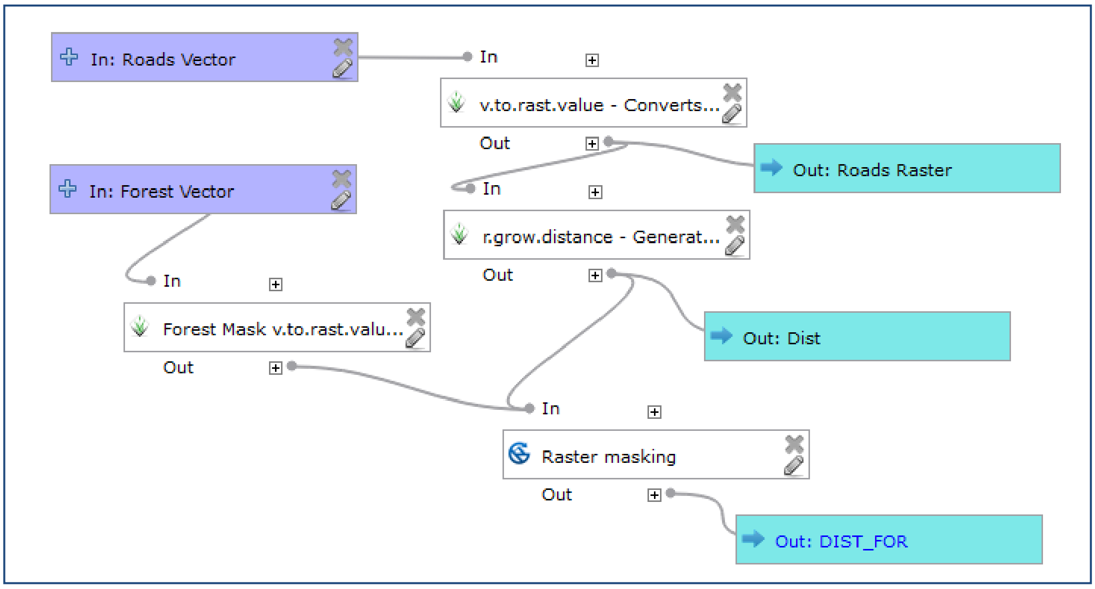

5.2. Transférer un flux de traitement dans QGIS (en utilisant l'exemple d'intervention REDD+)

a. Ouvrez **QGIS**

b. Depuis le menu principal cliquez sur **Traitement >>Options>> Général**

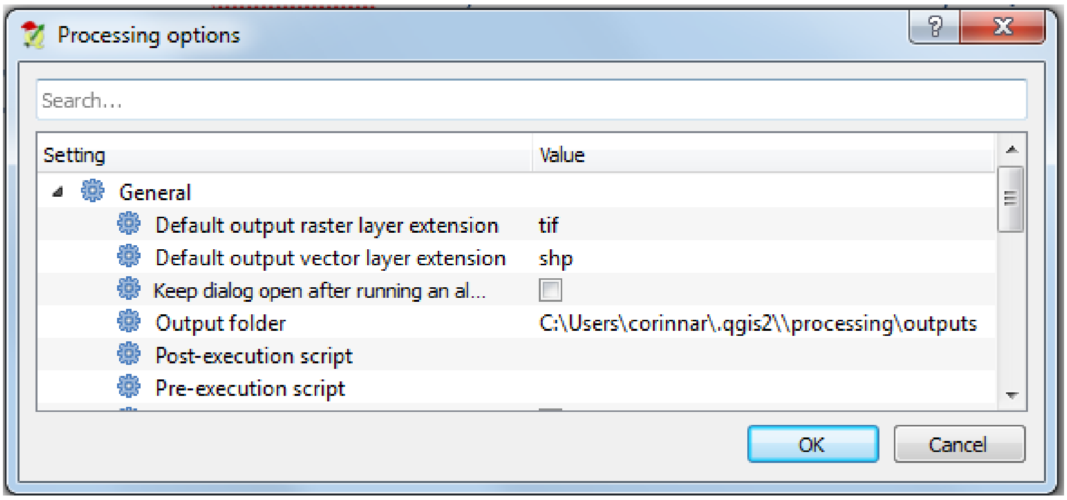

c. L'emplacement de sortie par défaut pour la sauvegarde des modèles est le dossier **qgis2**. Vous pouvez le changer si vous voulez les sauvegarder ailleurs, **notez l'emplacement**. Tous vos modèles de projet QGIS seront stockés à cet endroit.

d. Cliquez sur **OK** pour fermer la fenêtre.

e. Copiez le **MODEL_BUILDER _DEMO** sur votre ordinateur.

f. Depuis le dossier **MODEL_BUILDER _DEMO,** ouvrez le projet **Mythical_Model_Builder.qgs.**

g. Familiarisez-vous avec les données de la **Table des matières** (à gauche du panneau) dans QGIS.

Les données utilisées comme exemples de ce tutoriel sont stockées dans le dossier **Mythical_Database**. Le tableau ci-dessous liste les couches de données qui sont contenues dans le dossier :

communes_mythical – Limites Administratives (Communes)	community_forestry_mythical – Sites sylvicoles communautaires actuels et proposés
DEM_mythical – Modèle Numérique de Terrain	communes_mythical – Limites Administratives (Régions)
future_LUP_agriculture_mythical – Plan d'utilisation future des terres en termes d'Agriculture	HILL_mythical – Ombrage des collines généré depuis le Modèle Numérique de Terrain
hydrobasins_mythical – Bassins hydrologiques	hydropower_mythical – Sites hydroélectriques planifiés et existants
lakes_rivers_mythical – Plan d'eau et rivières	land_concess_mythical – Concessions foncières à fins économiques
landuse_mythical – Utilisation des terres courante	market_mythical – Localisations des marchés
natural_forest_mythical – Un sous-ensemble de la couche actuelle de l'utilisation des sols (qui contient uniquement la classification des forêts naturelles)	PA_mythical – Aires protégées
mining_concessions_mythical – Concessions minières	roads_mythical – Routes existantes et planifiées
prod_for_mythical – Forêt de production	Poverty rate_mythical – Taux de pauvreté par commune
village_mythical – Localisations des villages	

Note : ces jeux de données ne seront pas tous utilisés dans l'analyse de démonstration, mais ils peuvent être utilisés comme données test lors du développement d'autres flux de traitements.

5.3. Créer un nouveau Modèle dans la boîte à outils du Traitement

Dans QGIS, les modèles sont stockés dans des groupes définis par l'utilisateur. Le groupe est spécifié au moment de la création d'un nouveau modèle.

a. Pour créer un nouveau modèle, depuis le menu principal cliquez sur **Traitement>>Modeleur Graphique**

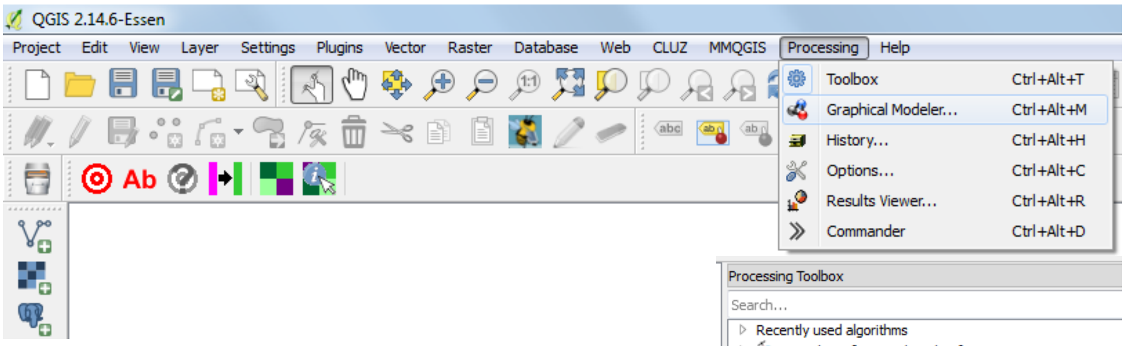

*Sinon, faites-le depuis la **Boîte à Outil de géotraitement**, déroulez les **Modèles**, déroulez les **Outils** puis cliquez sur **Créer un nouveau modèle.***

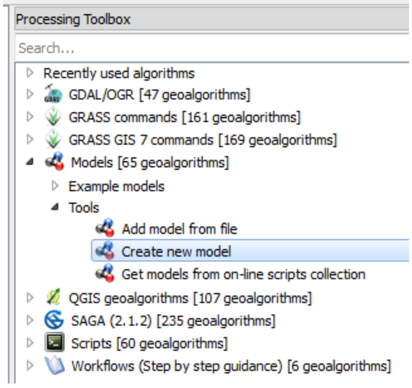

La fenêtre **du modeleur de traitement** apparaît.

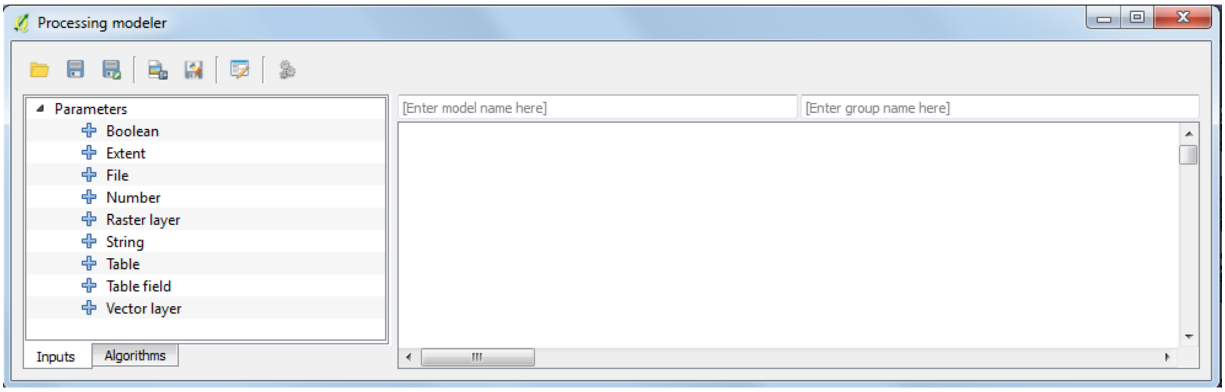

Des zones de texte sont disponibles en haut pour spécifier le nom du modèle et le groupe auquel il appartient. Le groupe pourrait contenir des modèles liés à un thème particulier ou développés pour un projet particulier par exemple.

- **a.** Donnez un nom au modèle, ex. **Démo Intervention SC pour contrer le Facteur Manioc.**
- **b.** Donnez un nom au groupe, ex. **flux de traitements de l'intervention REDD+.**

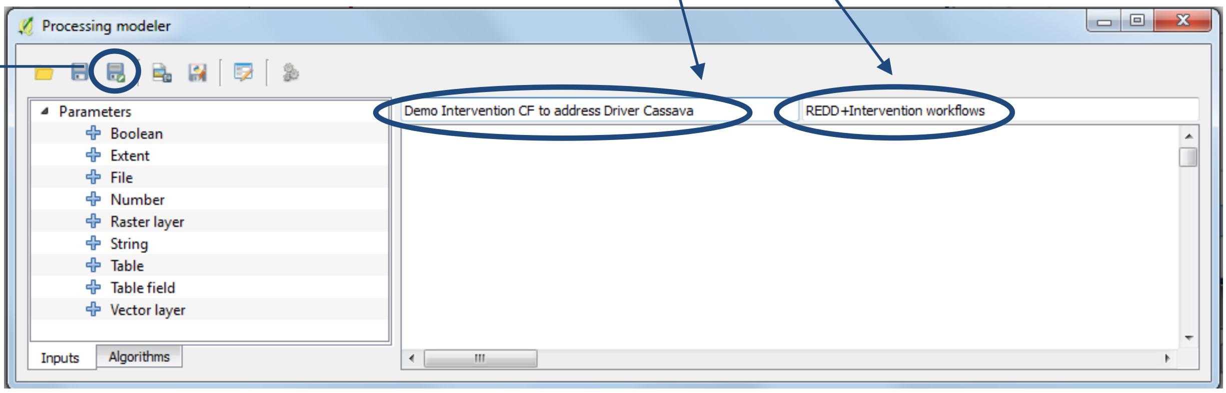

- **c.** Cliquez sur le bouton **Enregistrer sous** et enregistrez le modèle. Il sera sauvegardé dans l'emplacement par défaut à moins que vous en ayez choisi un autre à l'étape 5.2b, ex. **C:\Users\xxxxxxxxxxxx\.qgis2\processing\models**

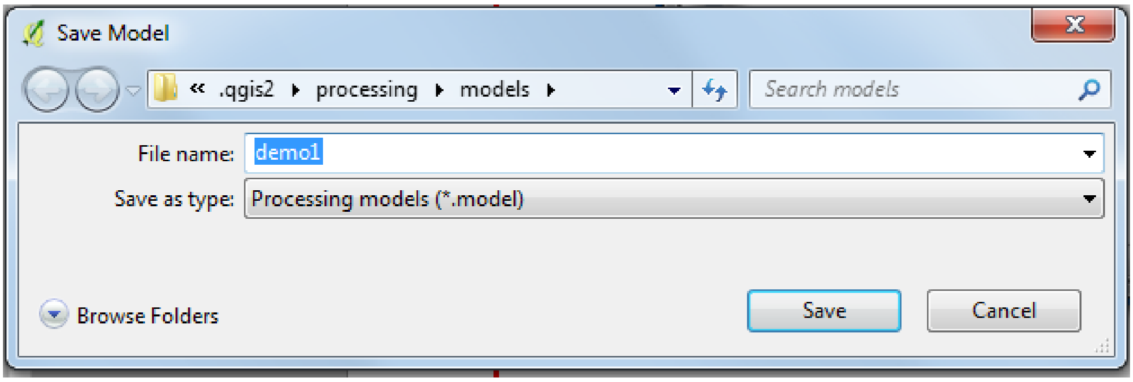

- **d.** Cliquez sur **Enregistrer.**
- **e.** Fermez le modèle en cliquant sur la **croix** en haut à droite de la fenêtre du **modeleur de traitement**.

Dans la **Boîte à Outils de Traitements** vérifiez que votre nouveau groupe modèle (**flux de traitements de l'intervention REDD+**) est apparu comme un sous-ensemble de **Modèles** et que votre nouveau modèle se situe dans cet ensemble.

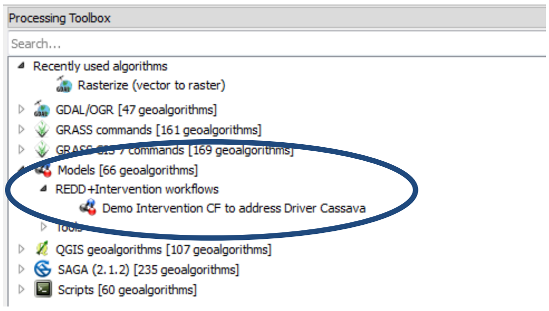

5.4. Ajouter le flux de traitements au modèle

Nous utiliserons l'exemple de flux de traitements développé et décrit dans la **Section 4** pour réaliser cette démonstration.

5.4.1. Ajouter la première étape à l'outil - Sélectionner la forêt Naturelle dans l'étendue du facteur

La première étape consiste à sélectionner les zones risquant d'être affectées par le facteur* « conversion vers le manioc » et au sein de ces zones, assurez-vous que l'intervention « Sylviculture Communautaire » ne peut uniquement se faire que dans les zones de forêt naturelle.

*(*Dans cet exemple un fichier shape existe déjà pour la zone du facteur).*

a. Dans la **boîte à outils de géotraitement,** faites un clic droit sur le modèle que vous avez créé lors des étapes précédentes et cliquez sur **Éditer modèle.**

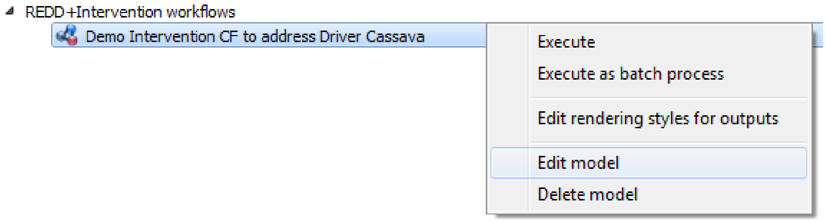

Jetez un œil à votre flux de traitement planifié. Pour l'Étape 1, nous pouvons voir une fonction « Extraction par masque » pour découper **les zones de forêt naturelle** dans **l'étendue du facteur**.

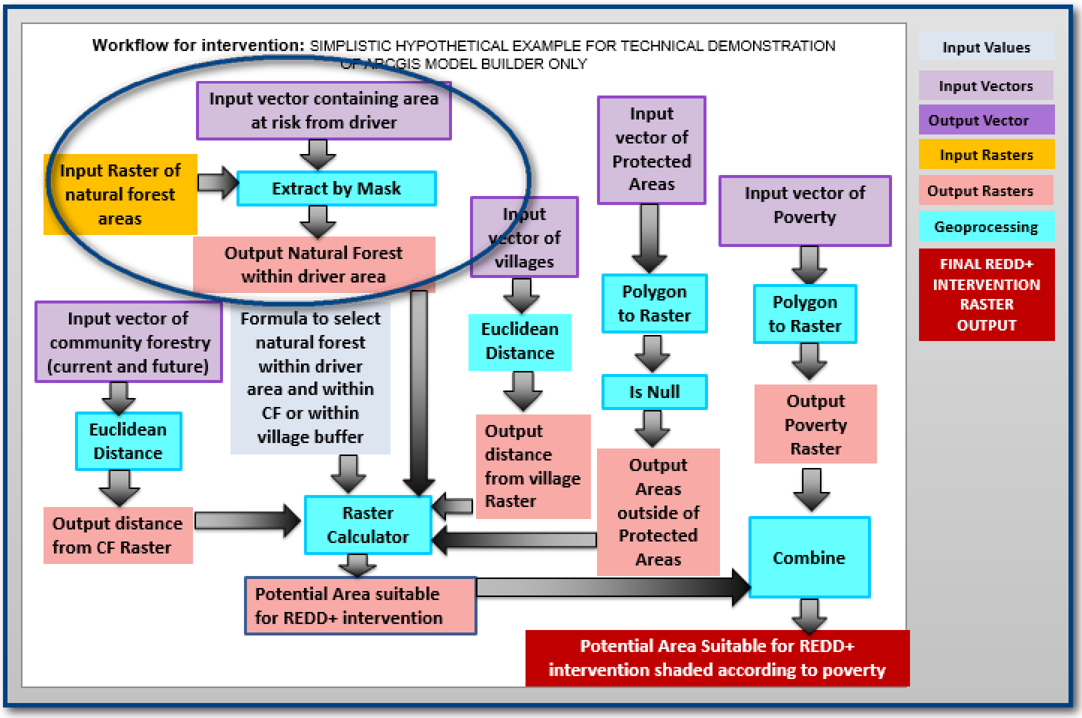

b. Rappelez-vous que le flux de traitements a été conçu pour ArcGIS, les noms des outils peuvent donc être légèrement différents. Dans QGIS, si nous cliquons sur l'onglet **Algorithmes** et cherchons une fonction **Extraire**, nous ne trouverons aucun outil de géotraitement nommé **Extraire par masque** mais si nous cherchons une fonction **découper**, nous pouvons trouver deux outils qui peuvent fonctionner de façon similaire : **Découper un raster selon une couche de masque (GDAL)** et **Clip raster with polygons (SAGA) (Découper un raster avec des polygones)**.

c. Ces deux outils devraient nous permettre d'extraire une zone d'intérêt mais pour cet exemple, nous utiliserons l'outil **SAGA**. Double-cliquez sur l'outil **Clip raster with polygon.**

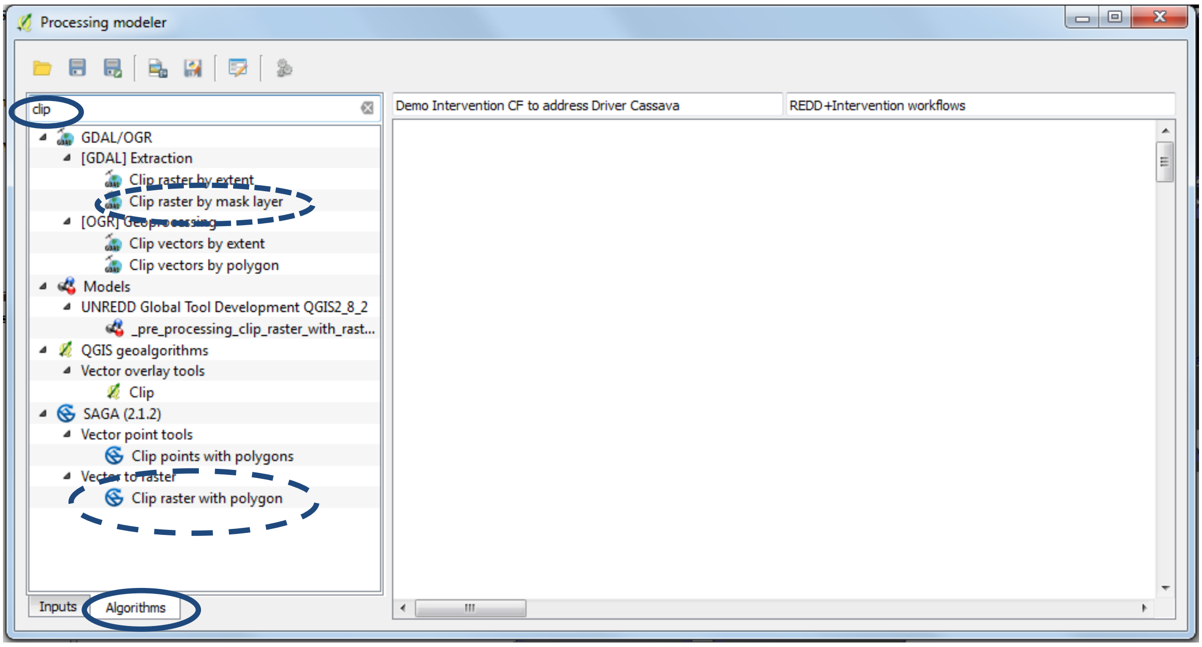

*Une fenêtre apparaît ; elle est semblable à la boîte de dialogue qui s'ouvre lorsque vous utilisez l'outil directement depuis la **boîte à outils de traitements.***

d. Essayez de sélectionner un **Entrant** (raster) et une couche **Polygones** (couche permettant la découpe). Il ne vous est pas possible de sélectionner ces données puisque vous devez les insérer dans votre modèle en tant que **Input** dans l'outil **Clip raster with polygon**.

e. Cliquez sur **Annuler** pour retourner sur la fenêtre du **modeleur graphique**.

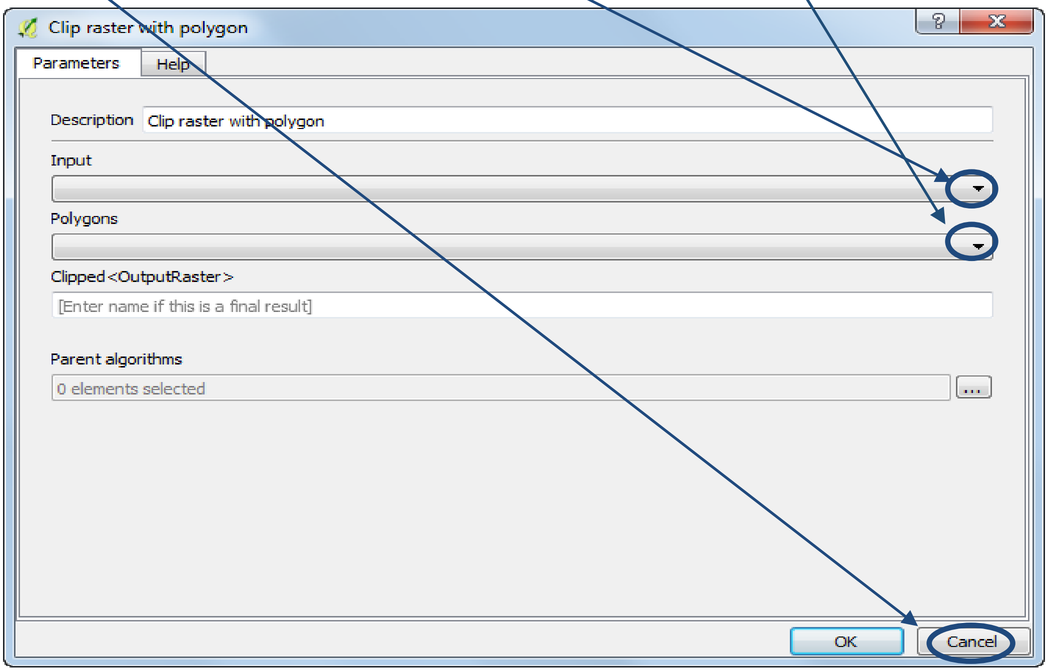

f. Cliquez sur l'onglet **Entrées.**

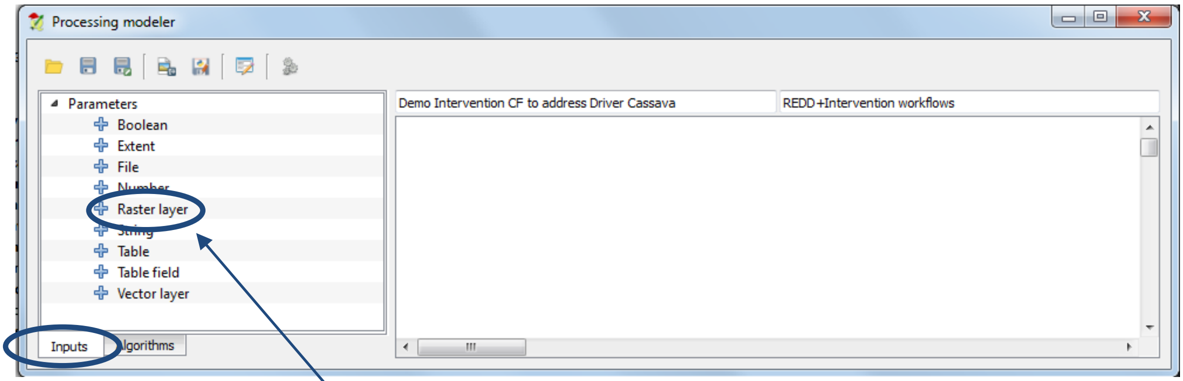

g. Double-cliquez sur **Raster layer** dans la liste des paramètres.

h. Donnez un nom au paramètre (Il s'agit du nom qui apparaîtra dans la boîte de dialogue du modèle lorsque vous exécuterez l'outil). Dans cet exemple, inscrivez **Raster Forêt Naturel à Découper**. Le jeu de données est nécessaire à l'outil, laissez ainsi **Requis** par défaut sur **Oui**.

i. Cliquez sur **OK** et le Paramètre raster est ajouté dans les canevas modèles.

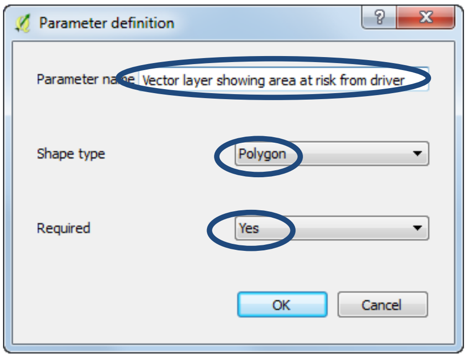

j. Double-cliquez sur **Vector layer** pour ajouter un autre paramètre. (c.à.d.. la couche polygone à utiliser comme masque de découpage).

k. Donnez un nom au paramètre. Dans cet exemple, indiquez **Couche vectorielle montrant les zones risquant d'être affectées par le facteur.**

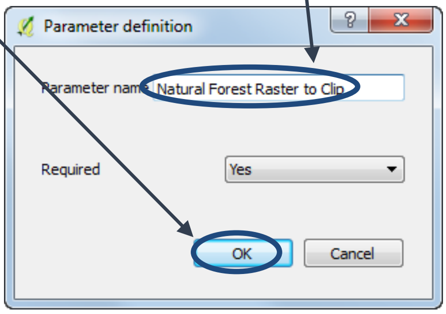

l. Changez le type de forme sur **Polygone.**

m. Laissez **Requis** sur **Oui.**

n. Cliquez sur **OK** et le Paramètre vectoriel est ajouté dans les canevas modèles.

o. Cliquez à nouveau sur l'onglet **Algorithmes** et double cliquez sur l'outil **Clip raster with polygon**.

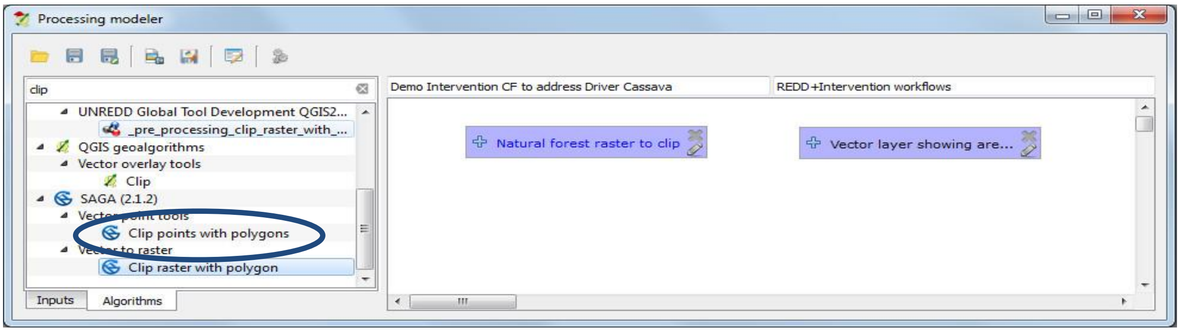

p. Changez la description par exemple en **Étape 1 : Découper la forêt naturelle selon l'étendue du facteur.**

q. Indiquez un nom pour la valeur du paramètre sortant, ex. **RasterSortant: natural_ forest_in_driver_area**

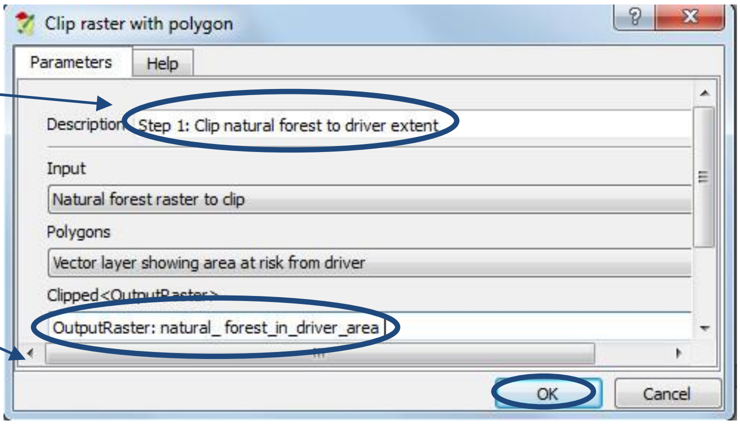

r. **QGIS** récupère automatiquement les paramètres d'**Entrées** et les **Polygones** et il peut le faire seul cette fois puisqu'il n'y a qu'un seul vecteur et un seul paramètre raster dans le modèle. Cliquez **sur OK.**

s. La première étape est maintenant terminée, cliquez sur le **bouton Enregistrer** pour sauvegarder le modèle.

t. Cliquez sur le bouton **Lancer le modèle** pour tester le modèle en l'état. Nous trouvons immédiatement un problème sur notre flux de traitements planifié - notre couche forêt naturelle est un jeu de données vectorielles et le modèle nécessite un **raster**. Ainsi, nous avons besoin d'ajouter une nouvelle étape au début de notre modèle pour convertir levecteur forêt naturelle en raster.

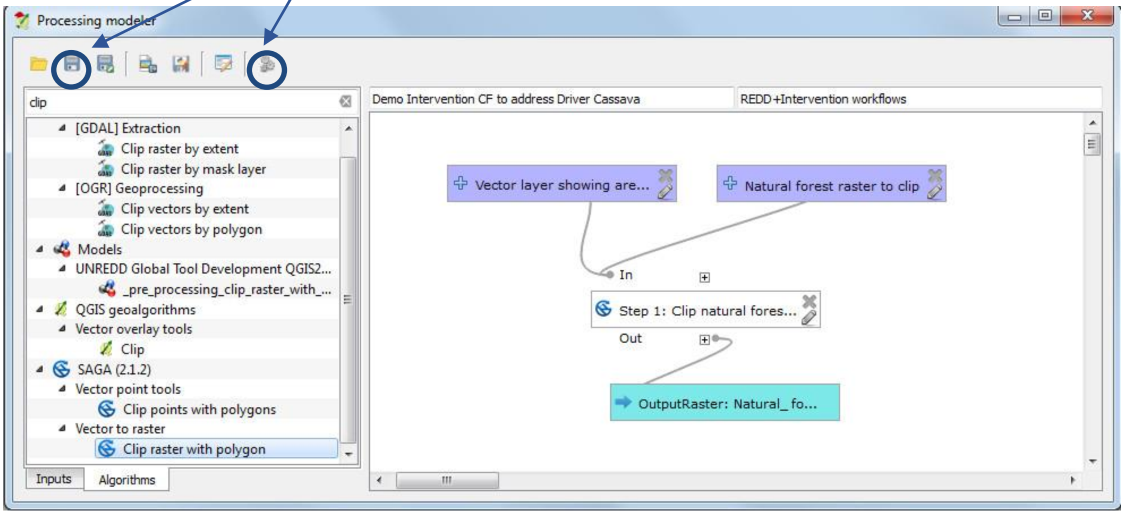

u. Cliquez sur **Fermer** pour fermer votre outil. Vous retournez vers le **Modeleur Graphique.**

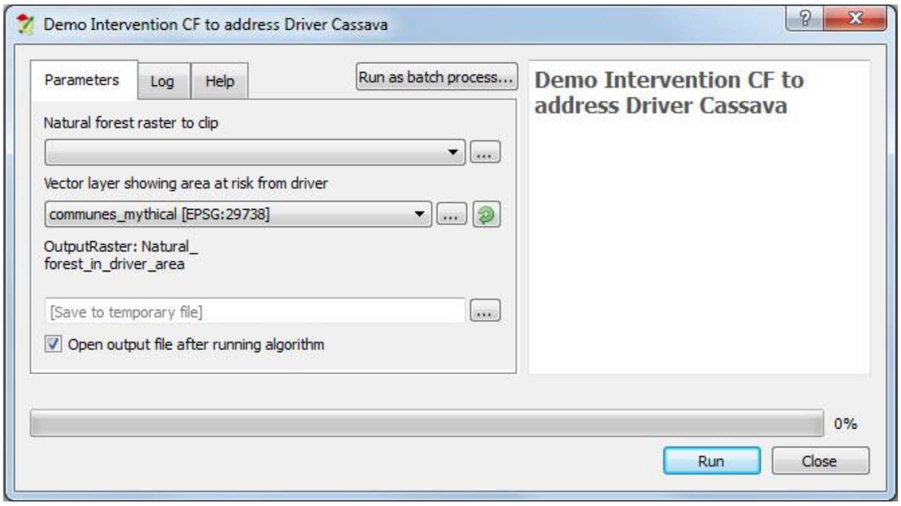

5.4.2. Modifier la première étape

a. Nous avons besoin d'ajouter une couche vecteur au canevas **Modeleur de traitement** ; celle-ci permettra la conversion en raster, cliquez sur **Entrées** et sélectionnez **Vector layer** et faites-le glisser vers le canevas modèle.

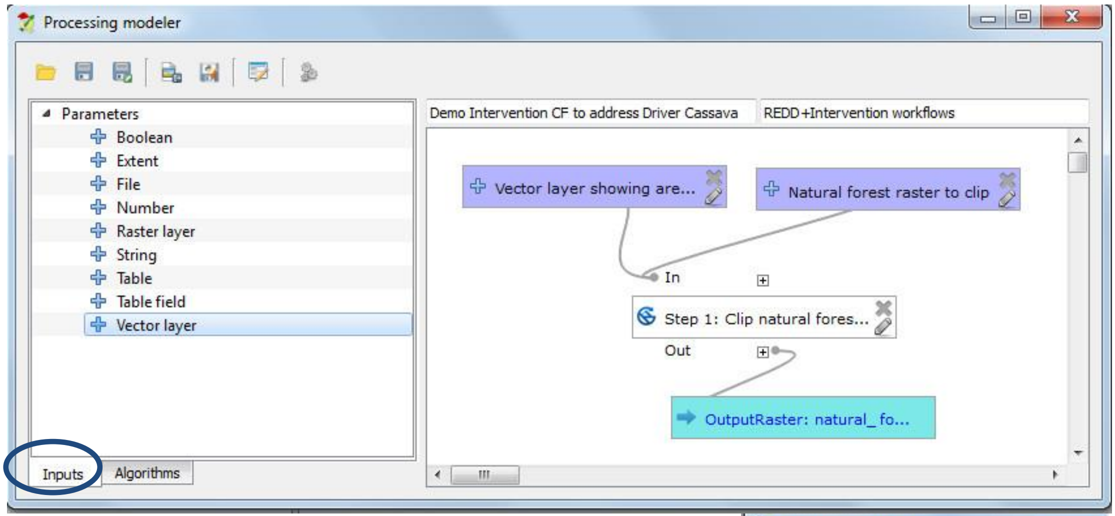

b. Indiquez **vecteur forêt naturelle** dans la boîte **paramètre du nom**.

c. Réglez le **type de forme** sur **Polygone.**

d. Cliquez sur **OK.**

e. Dans le **Modeleur de Traitement**, cliquez sur l'onglet **Algorithmes** et cherchez **v.to.rast.**

f. Sélectionnez l'**outil v.to.rast.value** et faites le glisser dans le canevas modèle.

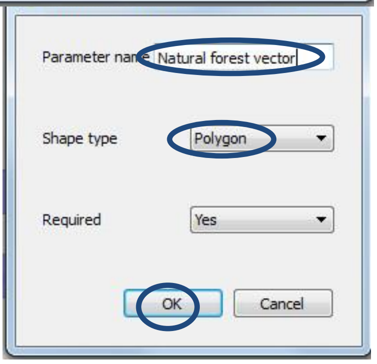

g. Changez la **description** en **Étape 1 : Convertir le vecteur forêt naturelle en raster.**

h. Pour la **couche vectorielle entrante** sélectionnez le paramètre **vecteur forêt naturelle.**

i. Réglez la **taille des cellules** sur la taille que vous voulez utiliser pour l'analyse, par exemple **1000.**

j. Indiquez un nom pour la valeur du paramètre sortant **Rasterized <OutputRaster>, ex. RasterSortant : natural_forest.**

k. Cliquez sur **OK.**

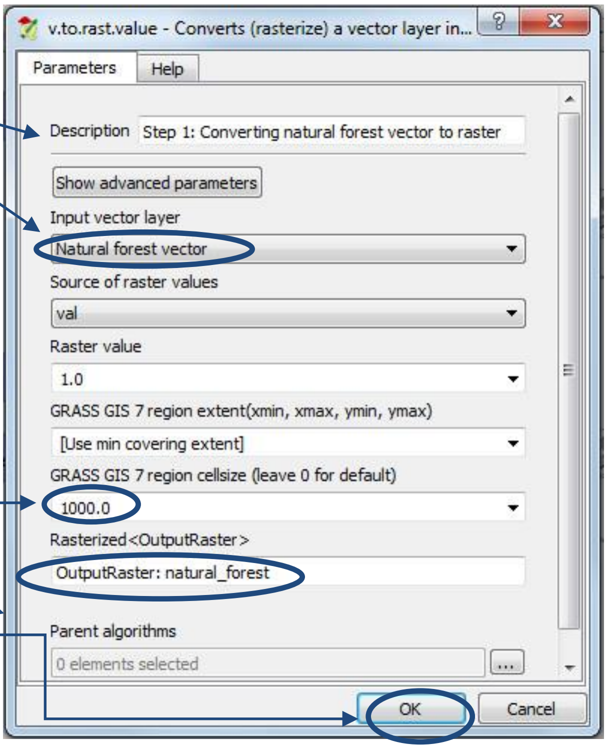

Vous pouvez déplacer les éléments sur le canevas modèle afin de les voir de façon claire et logique (comme dans l'extrait ci-dessous). Une fois que vous les avez déplacés, vous verrez qu'il existe alors deux parties distinctes qui ne sont pas encore liées l'une à l'autre. Pour les lier, le sortant de la première étape va remplacer le raster d'entrée Forêt Naturelle de la seconde étape. Notez que vous aurez aussi besoin de changer le nom de la seconde étape en

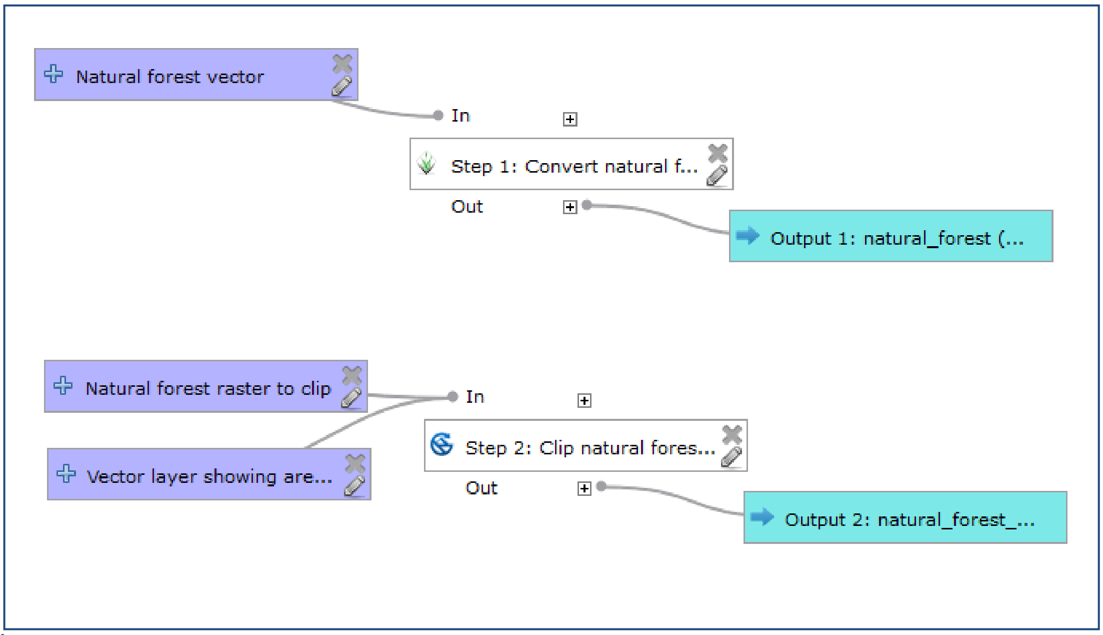

Étape 2.

l. Faites un clic droit sur l'outil **Étape 1 : Découper la forêt naturelle selon l'étendue du facteur** et cliquez sur **éditer.**

m. Changez la **description** pour qu'elle indique **Étape 2 : Découper la forêt naturelle selon l'étendue du facteur.**

n. Changez l'Input en « **Rasterized » créé par l'algorithme « étape1 : Convertir le vecteur forêt naturelle en raster »** pour récupérer le sortant de la première étape.

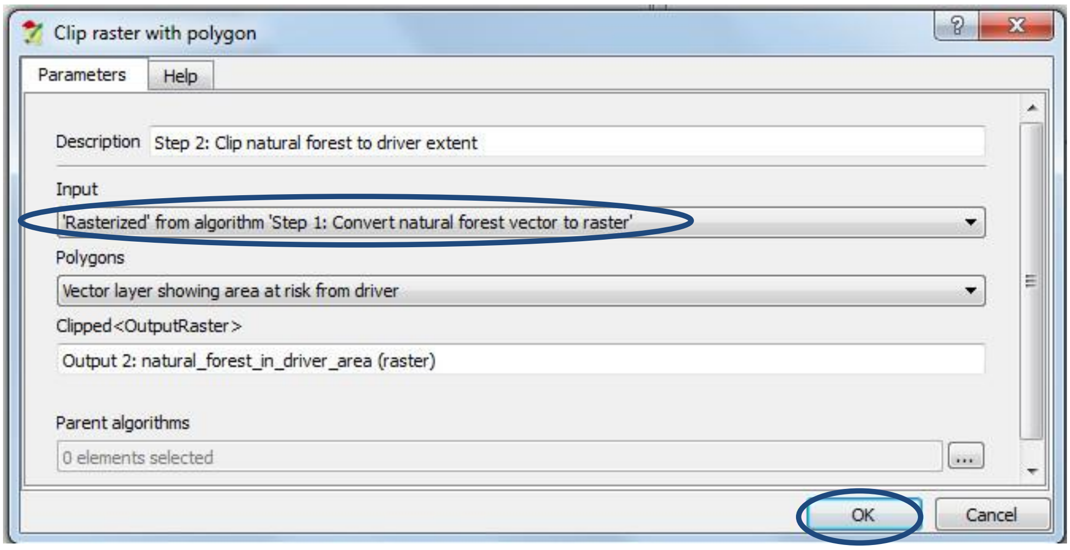

o. Cliquez **sur OK.**

L'ancien Raster Forêt Naturelle a été déconnecté du reste du modèle puisqu'il n'est plus nécessaire. Le raster forêt naturelle produit par l'étape 1 est maintenant l'entrée de l'étape 2.

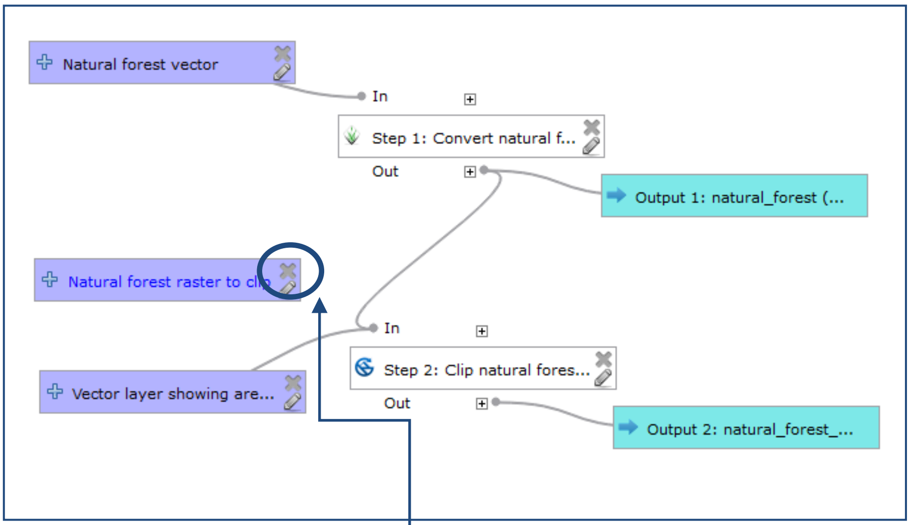

p. Pour enlever le paramètre redondant cliquez sur la **X** dans le coin en haut à droite.

q. Cliquez sur le bouton **Enregistrer** pour enregistrer le modèle.

r. L'outil devrait à présent ressembler à l'extrait ci-dessous**.** Pour essayer de faire fonctionner le modèle vous pouvez appuyer sur le bouton **Lancer le modèle**.

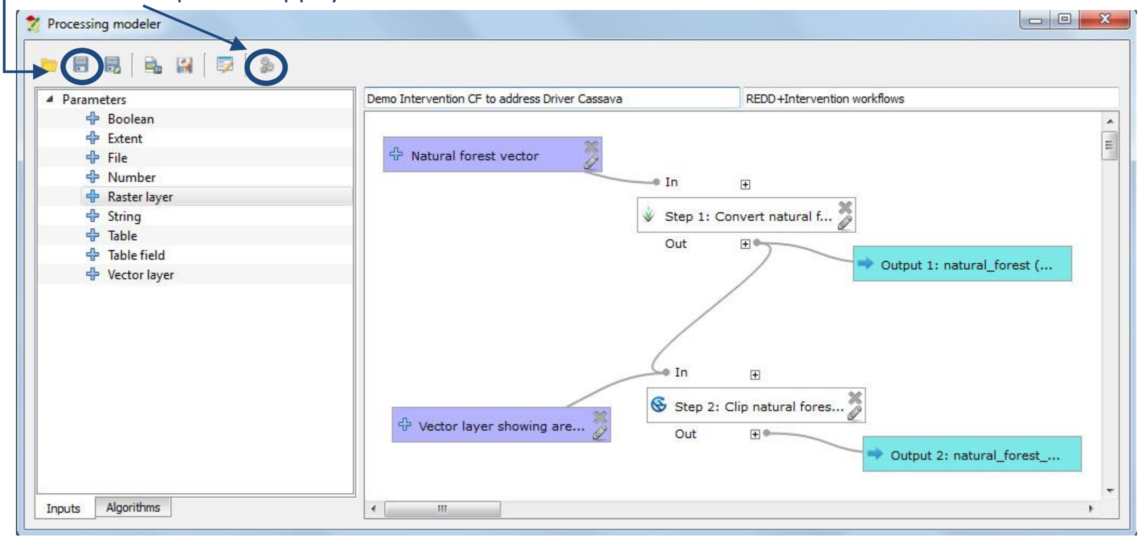

*Veuillez noter qu'il s'est parfois révélé que les tests du modèle dans la **fenêtre Modeleur de traitement** créaient une erreur mais le modèle fonctionne bien si vous le faites depuis la **boîte à outils de traitements**.*

*Si votre modèle s'ouvre alors sautez les étapes **s** et **t** ci-dessous.*

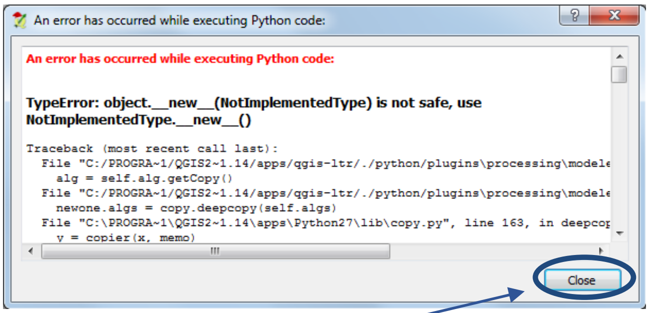

s. Si une erreur se produit cliquez sur **Fermer** sur la fenêtre du message d'erreur puis cliquez sur la **X** dans le coin supérieur droit pour fermer la fenêtre du **Modeleur de Traitements**.

t. Puis depuis le **Boîte à outils de traitements** déroulez votre groupe de **flux de traitements d'intervention REDD+** et double cliquez sur votre modèle **Démo Intervention SC pour contrer le Facteur Manioc.**

Au moment de l'ouverture, votre boîte de dialogue devrait ressembler à ceci :

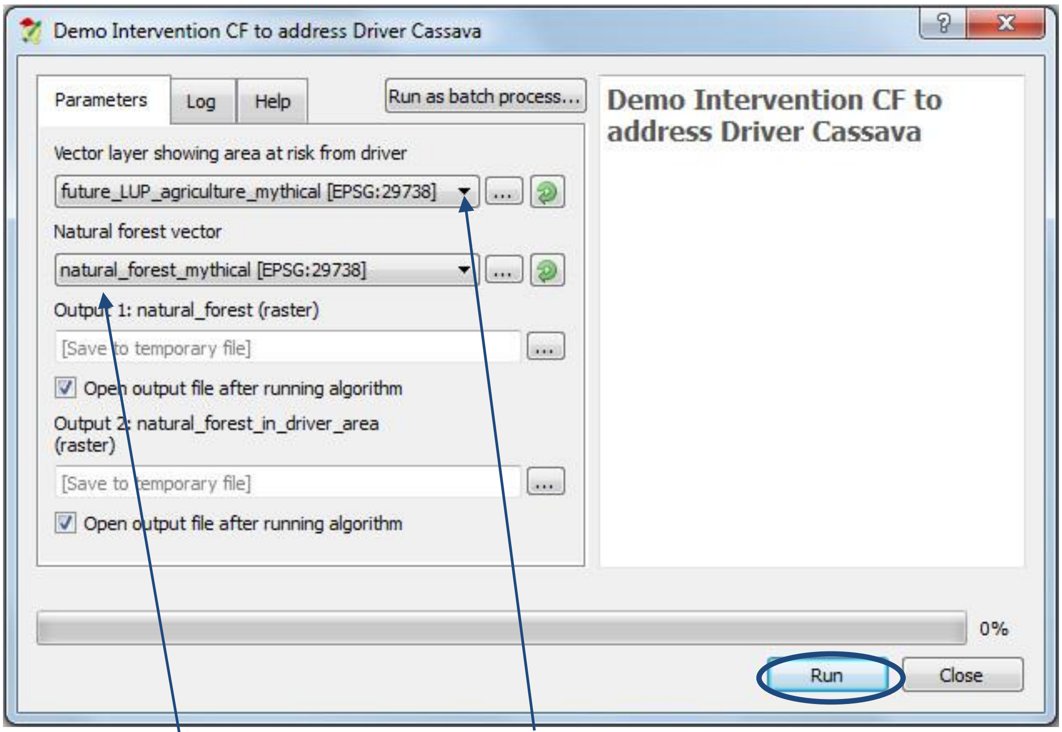

- **u.** Pour notre modèle exemple, nous avons choisi **future_LUP_agriculture_mythical** comme **couche vectorielle montrant les aires risquant d'être affectées par le facteur.**
- **v.** Choisissez **natural_forest_mythical** comme le **vecteur forêt Naturelle.**
- **w.** Tant que nous sommes en phase de test du modèle, laissez les deux sortants du modèle en tant que **[Enregistrer dans un fichier temporaire].**

  Cliquez sur **Run** pour exécuter le modèle.

Le modèle devrait bien fonctionner et se fermer automatiquement lorsqu'il est terminé. Vous pouvez voir la progression. L'extrait dans l'exemple ci-dessous montre que l'étape 1 a été exécutée et que le modèle réalise correctement l'étape 2. Les descriptions que vous avez entrées fournissent des informations précieuses concernant le processus en cours.

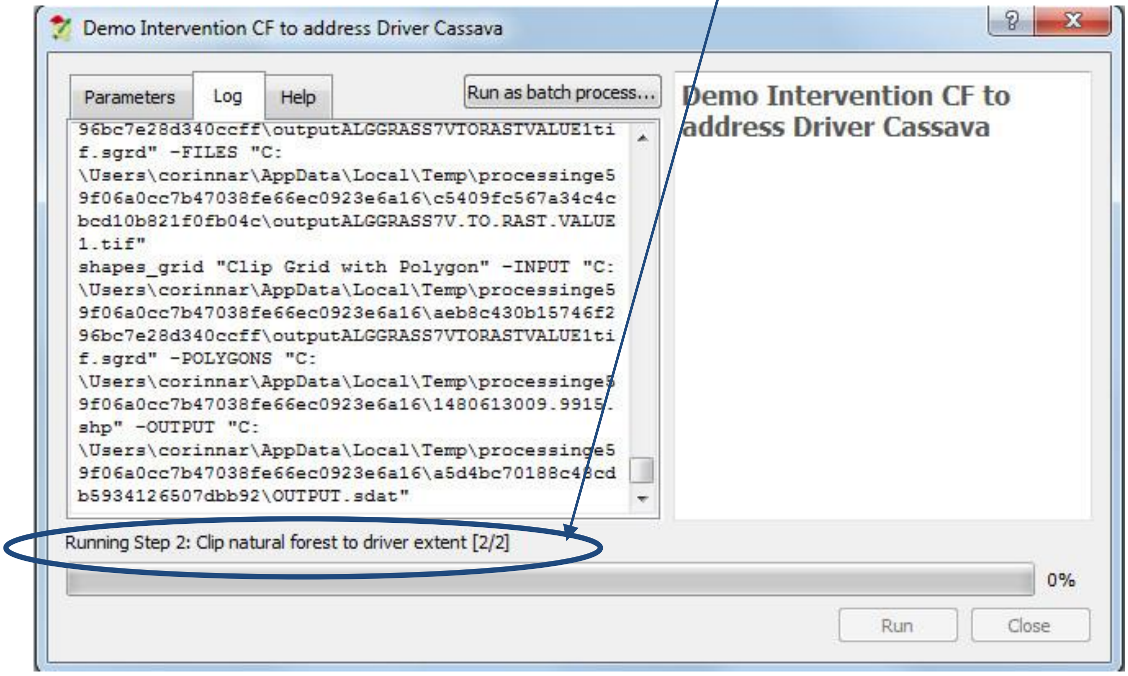

Une fois que le traitement est terminé, les sortants sont ajoutés à la table des matières comme présenté ci-dessous :

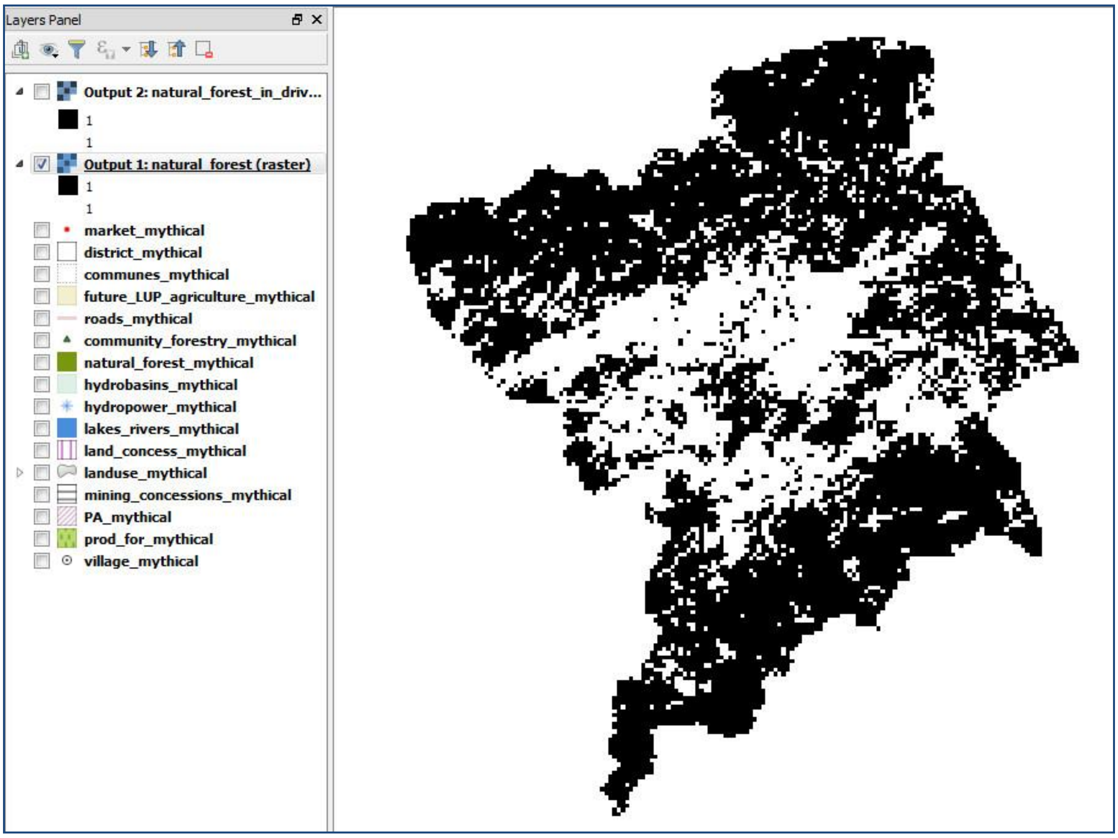

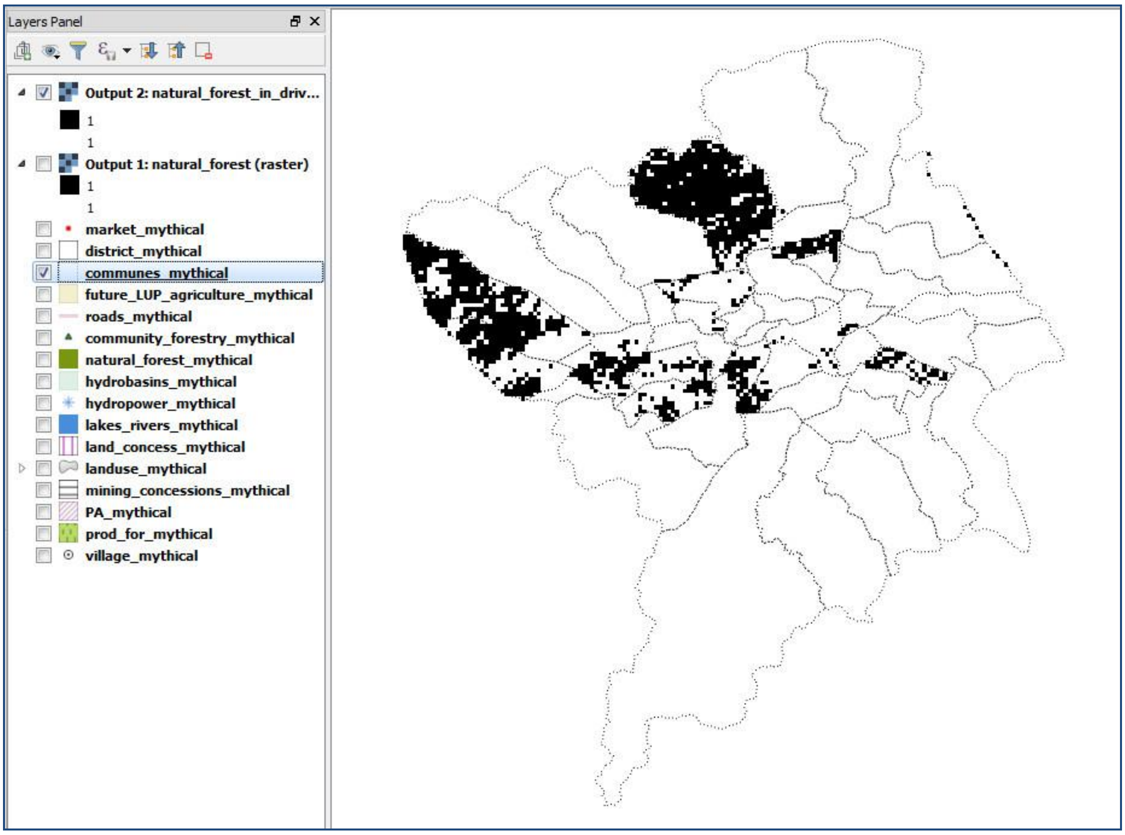

5.4.3. Documenter la première étape

Il est utile, à cette étape, de commencer à ajouter des métadonnées au modèle pour vous aider à garder trace des différentes étapes, de leurs entrants et de leurs sortants. Les métadonnées apparaissent dans l'outil de dialogue afin que le modèle soit plus facile d'utilisation.

a. Si vous avez fermé votre modèle, faites un clic droit sur votre modèle dans la **Boîte à outils de traitement** et cliquez **Éditer modèle** pour le ré-ouvrir dans le **Modeleur Graphique.**

b. Cliquez sur le bouton **Éditeur d'aide du modèle** dans la fenêtre du **Modeleur de traitement**.

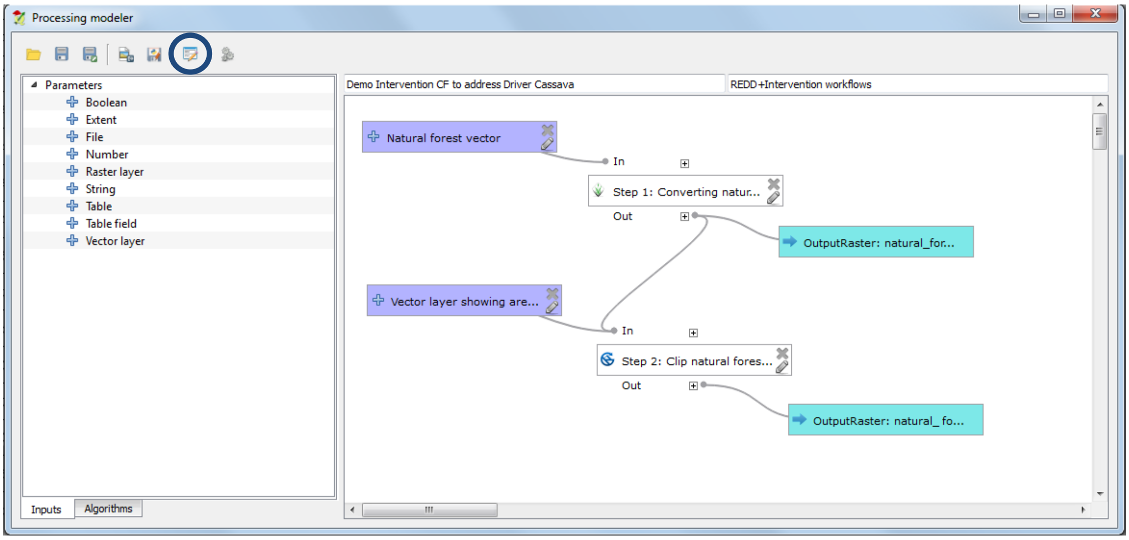

La fenêtre d'éditeur d'aide apparaît. En bas à gauche de la fenêtre, sélectionnez l'élément à éditer.

c. Dans un premier temps, cliquez sur **Algorithm description (Description de l'algorithme).**

d. Dans la fenêtre **element description (description de l'élément)**, décrivez l'objectif de votre modèle (de l'ensemble du modèle et non pas uniquement l'objectif des premières étapes que vous avez ajoutées). Pour notre exemple, vous pourrez écrire :

Un exemple de flux de traitements pour le développement d'une couche d'intervention REDD+ destinée à la sylviculture communautaire durable pour contrer le facteur « conversion à petite échelle de la forêt vers le manioc », en se basant sur le fait que les forêts précieuses pour la communauté risquent moins d'être converties en terres agricoles.

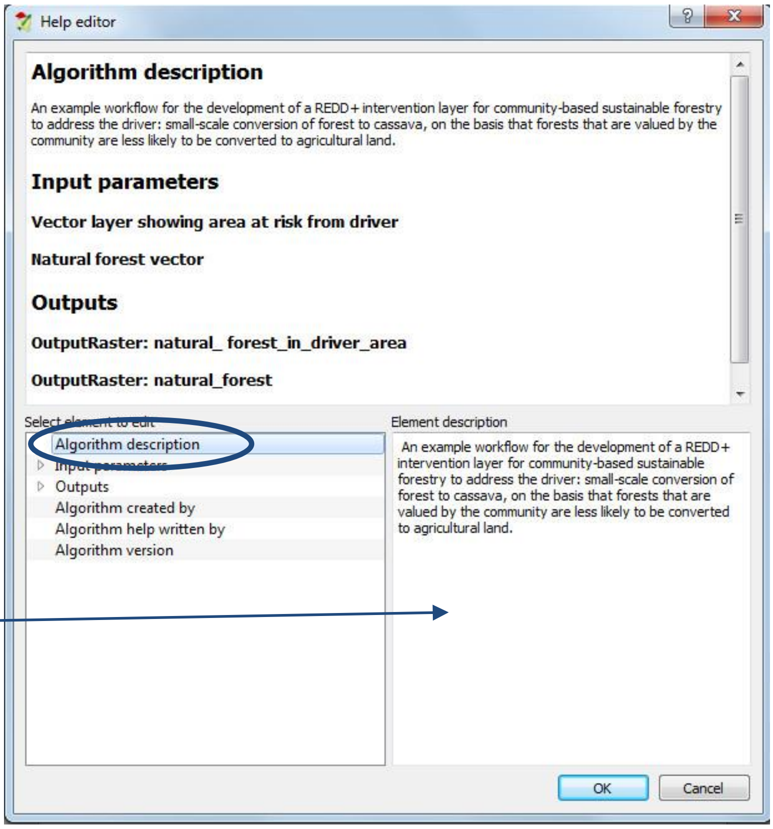

e. Cliquez sur la flèche pour étendre les **input parameters (paramètres entrants).**

f. Cliquez sur **la couche vectorielle montrant les aires risquant d'être affectées par le facteur** et indiquez les éléments de description suivants : **Choisissez une couche de polygones vectorielle qui montre uniquement les aires risquant d'être affectées par le facteur (c.à.d. dans ce cas la conversion à petite échelle de la forêt vers le manioc). Vous aurez besoin d'avoir préparé cette couche à l'avance pour exécuter le modèle ; ce flux de traitement ne crée pas cette couche pour vous.**

g. Cliquez sur **vecteur forêt naturelle** et indiquez l'élément de description suivant : **Choisissez une couche polygones vectorielle qui montre seulement ces zones de forêt naturelle. Vous aurez besoin d'avoir préparé cette couche à l'avance pour exécuter le modèle ; ce flux de traitement ne crée pas cette couche pour vous.**

h. Cliquez sur la flèche pour étendre les **outputs (sortants).**

i. Cliquez sur **Sortant 1 : forêt naturelle (raster)** et indiquez la description suivante : **Sortant créé par l'outil montrant les zones de forêt naturelle au format raster (résultat de la conversion d'un jeu de données vectorielles vers un raster).**

j. Cliquez sur **Sortant 2 : Natural_forest_in_driver_area (raster)** et indiquez la description suivante : **Sortant créé par l'outil montrant les zones de forêt naturelle dans l'étendue du facteur**

k. Cliquez sur **Algorithm created by (Algorithme créé par)** et indiquez par exemple **votre nom.**

l. Cliquez sur **Algorithm written by (Algorithme écrit par)** et indiquez par exemple **votre nom.**

m. Cliquez sur **Algorithm version (version de l'algorithme)** et indiquez le numéro de version, par exemple **1.0.**

n. Cliquez sur **OK** pour fermer l'aide.

o. Cliquez sur le bouton **Enregistrer** dans la fenêtre **du modeleur de traitement** pour sauvegarder votre modèle et l'aide nouvellement ajoutée.

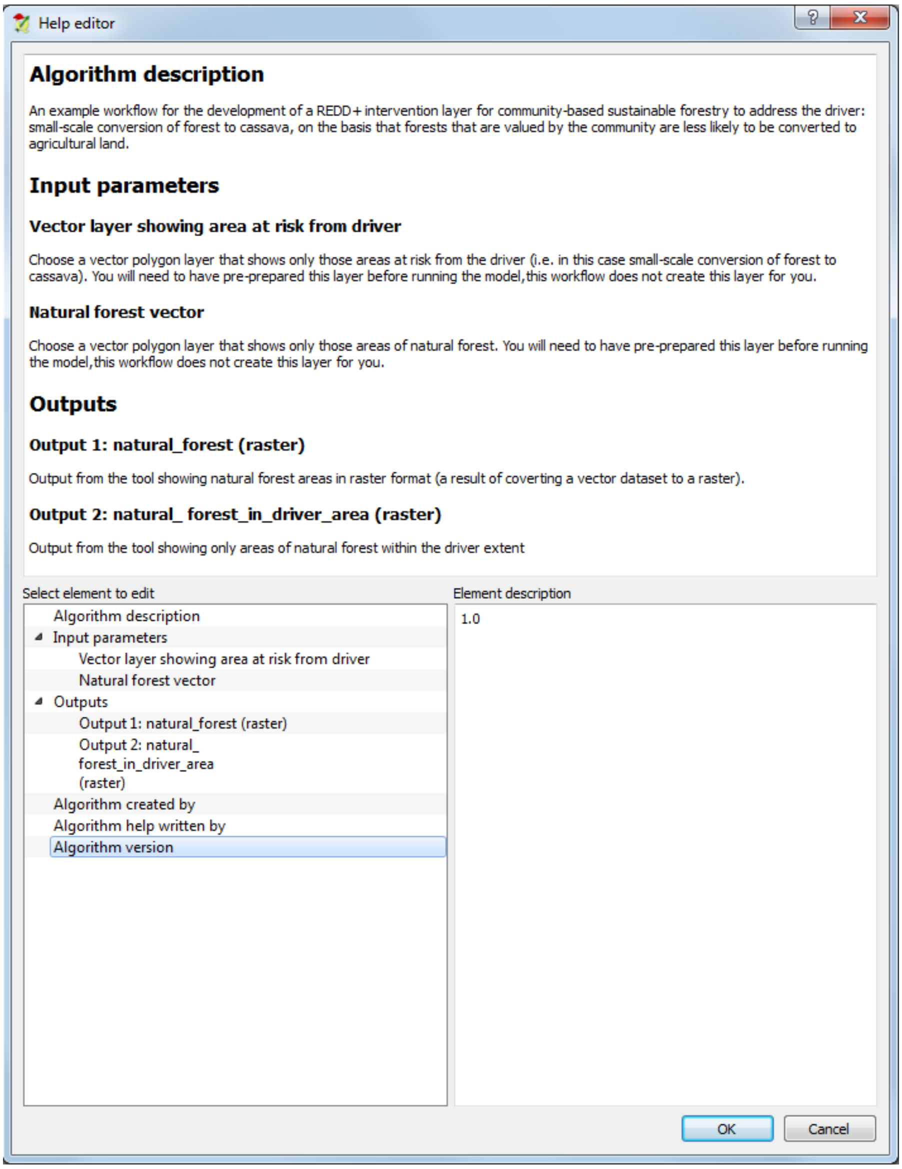

p. Cliquez sur le bouton **Lancer le modèle** et vérifiez que votre **description de l'algorithme** (l'objectif du modèle - flux de traitements) est affichée dans la boîte de dialogue de l'outil.

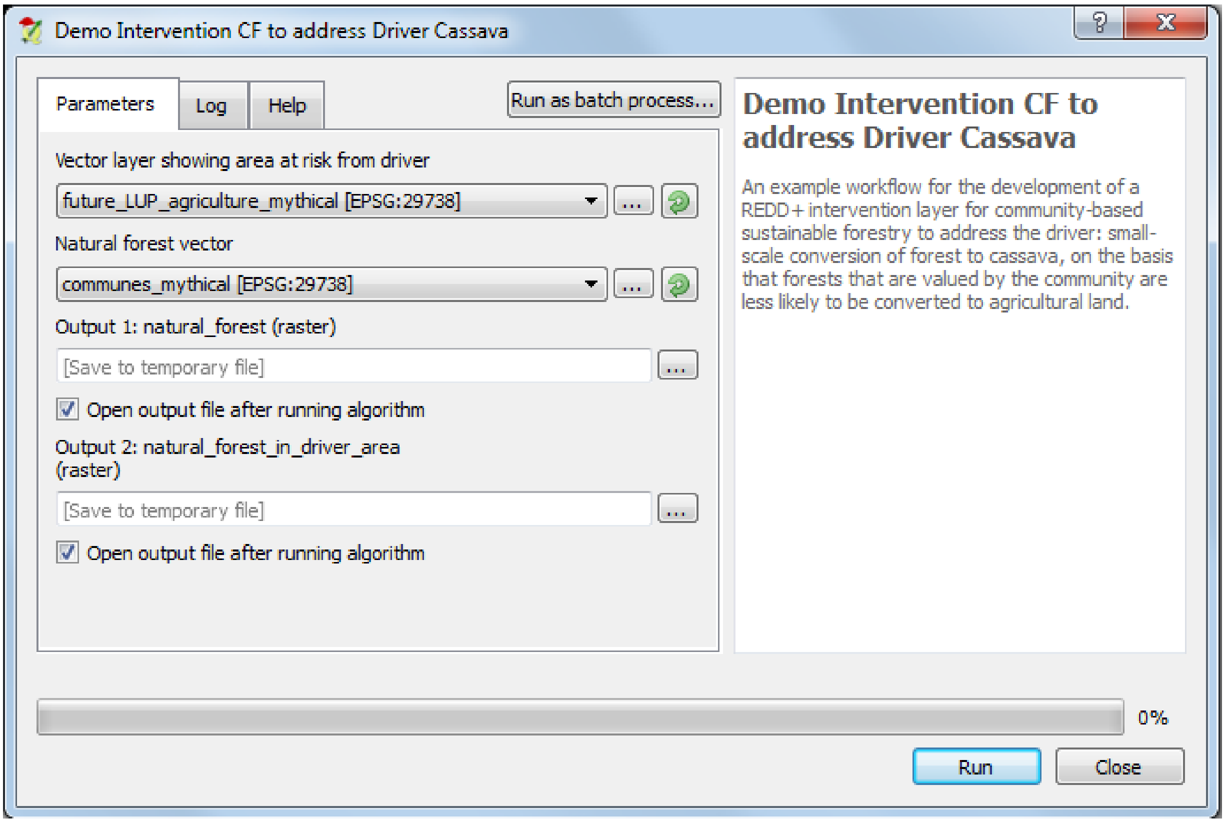

q. Cliquez sur l'onglet **Help (Aide)** et vérifiez que votre texte d'aide s'affiche dans la boîte de dialogue de l'outil.

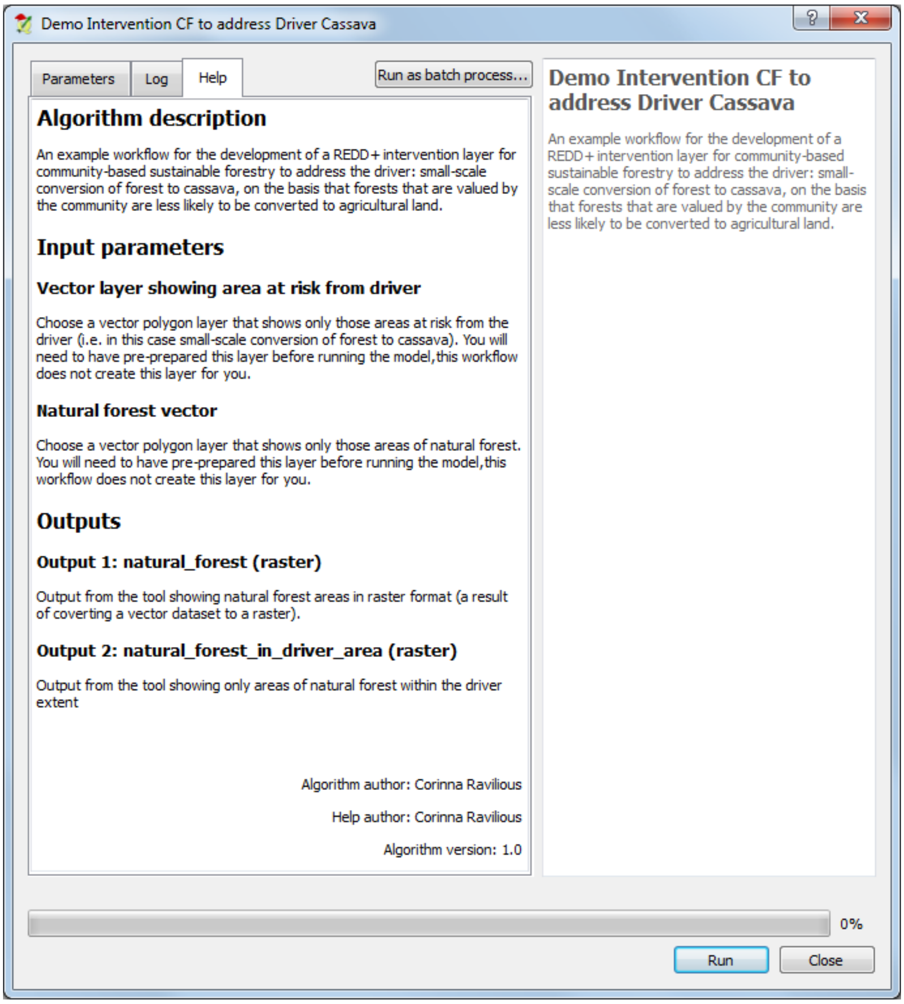

5.4.1. Ajouter les étapes restantes du flux de traitements

5.4.1.1. Distance par rapport aux sites de Sylviculture Communautaire

Regardez à nouveau votre flux de traitements pour voir la prochaine étape à ajouter au **Modeleur Graphique**. Il y a une fonction **Euclidean Distance (Distance Euclidienne)** précisée à gauche. Celle-ci générera un raster distance de sortie à partir du Raster sites de sylviculture communautaire.

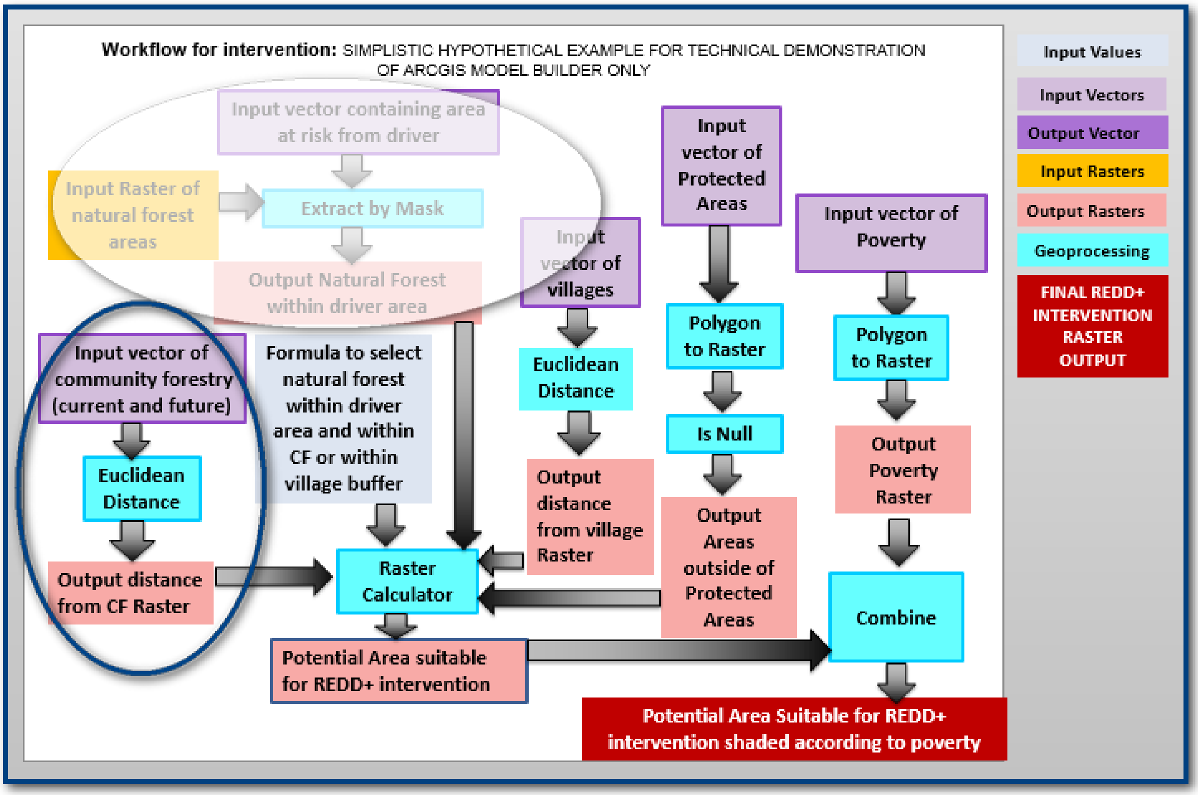

Dans QGIS, la **Distance Euclidienne** peut être générée par plusieurs outils : **Proximité (raster distance) (GDAL), Proximity raster (SAGA)** ou **r.grow.distrance (GRASS)**. Dans cet exemple, nous avons choisi d'utiliser l'**outil r.grow.distance.**

Nous voulons générer une surface représentant la distance par rapport aux zones de sylviculture communautaire, qui servira de jeu de données d'entrées. Notez toutefois, que l'outil **r.grow.region** fonctionne avec une couche raster ; ainsi, avant d'ajouter l'outil **r.grow.distance** nous devons utiliser l'outil **r.to.raster.value** pour convertir les polygones sylviculture communautaire en raster.

a. Cliquez sur l'**onglet Entrées** pour ajouter une **couche vectorielle.**
b. Indiquez le **nom du Paramètre,** ex. **vecteur Sylviculture Communautaire.**
c. Changez le **type de forme** sur **Point.**
d. Cliquez **sur OK.**

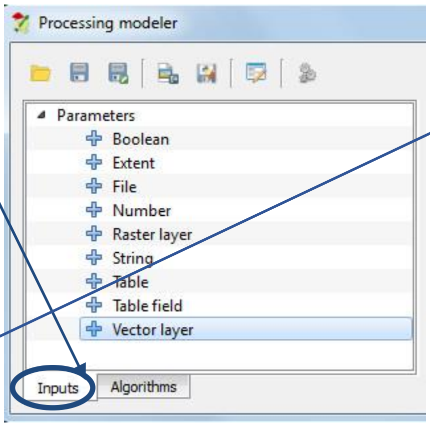

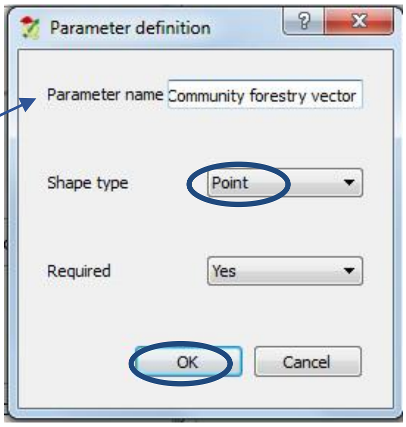

e. Cliquez sur l'onglet **Algorithmes** et cherchez **v.to.rast.a**

f. Double cliquez pour ajouter l'outil **v.rast.value**.

g. Changez la description en **Étape 3 : Convertir le vecteur sylviculture communautaire vers un raster**

h. Sélectionnez le **vecteur Sylviculture Communautaire** comme **Input vector layer (couche vectorielle entrante).**

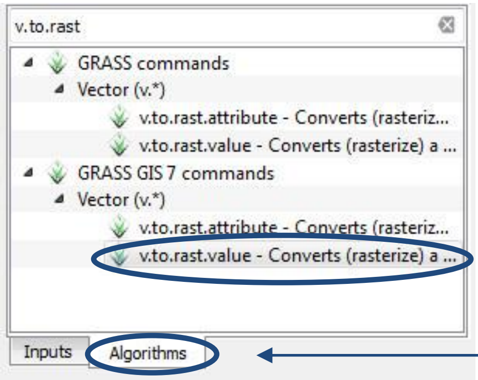

i. Réglez la **taille de la cellule**, par exemple sur **1000.**

j. Cliquez sur **OK pour ajouter l'outil.**

k. Cliquez sur l'onglet Algorithmes et cherchez r.grpw.

l. Double cliquez pour ajouter l'outil r.grow.distance.

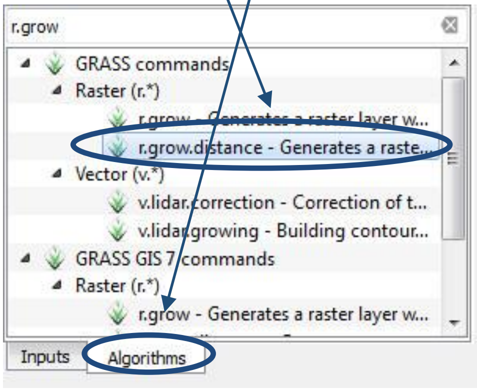

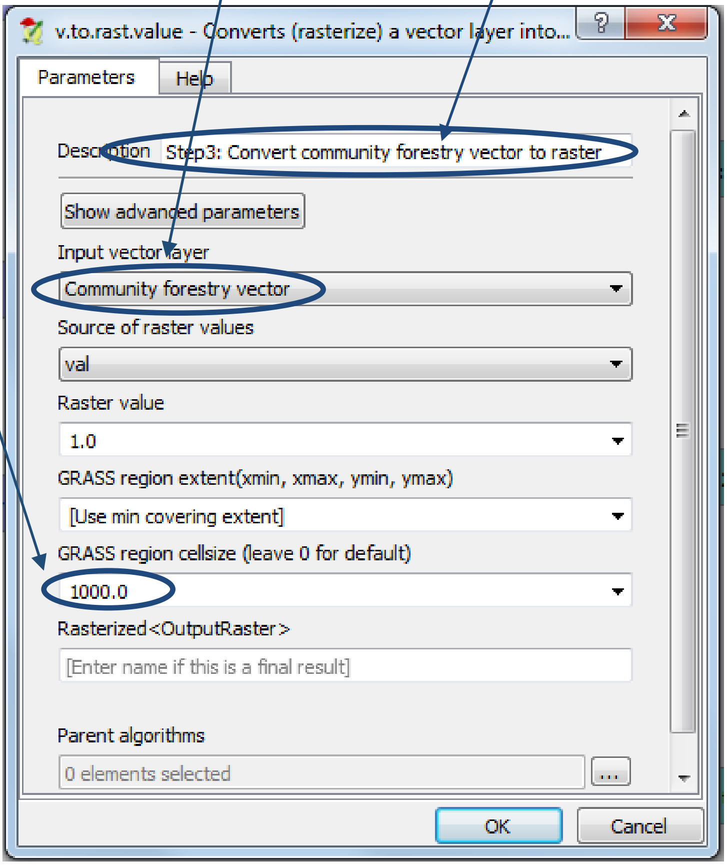

m. Changez la description en **Étape 4 : Créer un raster surface lié à la distance à la sylviculture communautaire.**

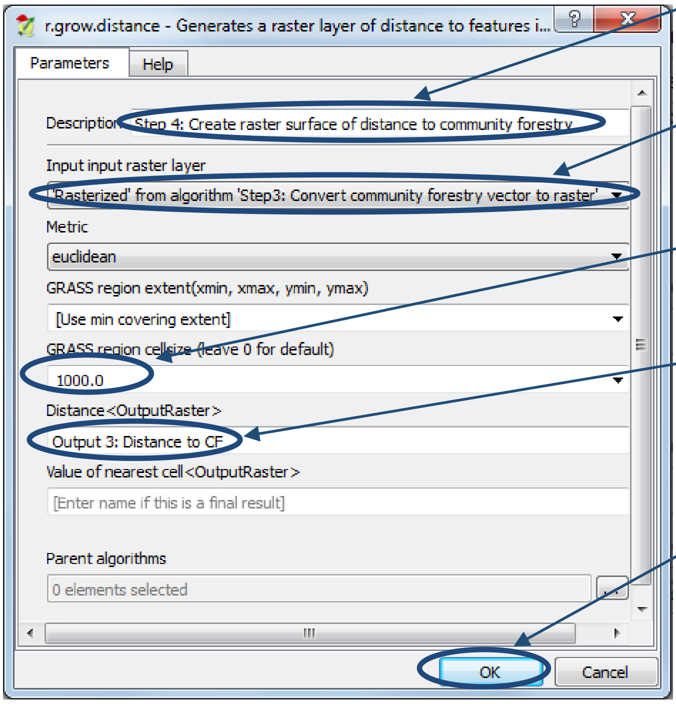

n. Réglez la Input raster **layer (couche raster entrante)** sur **« Rasterized » créée par l'algorithme « Étape 3 : Convertir le vecteur sylviculture communautaire vers un raster ».**

o. Réglez la **taille de la cellule**, par exemple sur **1000**

p. Donnez au paramètre **Distance<Output> (distance<sortante>)** un nom, par exemple. **Sortant 3 : Distance au SC.**

q. Cliquez sur **OK pour ajouter l'outil.**

r. Cliquez sur le bouton **Enregistrer** pour enregistrer le modèle.

s. Cliquez sur le bouton **éditeur d'aide du modèle** pour ajouter une documentation d'aide aux nouvelles étapes du modèle.

t. Déroulez les **paramètres d'entrée**, cliquez sur **sylviculture communautaire** et indiquez la description suivante : **Choisissez une couche de points vectorielle qui montre uniquement les sites de sylviculture communautaire. Vous aurez besoin d'avoir préparé cette couche à l'avance pour exécuter le modèle ; ce flux de traitement ne crée pas cette couche pour vous.**

u. Déroulez les **sortants**, cliquez sur **Sortant 3 : distance au SC** et indiquez la description suivante : **Sortant créé depuis l'outil montrant un raster surface des distances par rapport aux sites de sylviculture communautaire.**

v. Cliquez sur **OK** puis cliquez sur le bouton **Enregistrer** pour sauvegarder le modèle et l'aide du modèle.

w. Cliquez sur **lancer le modèle** pour le tester.

x. Une fois que le traitement est terminé, les sortants sont ajoutés à la table des matières.

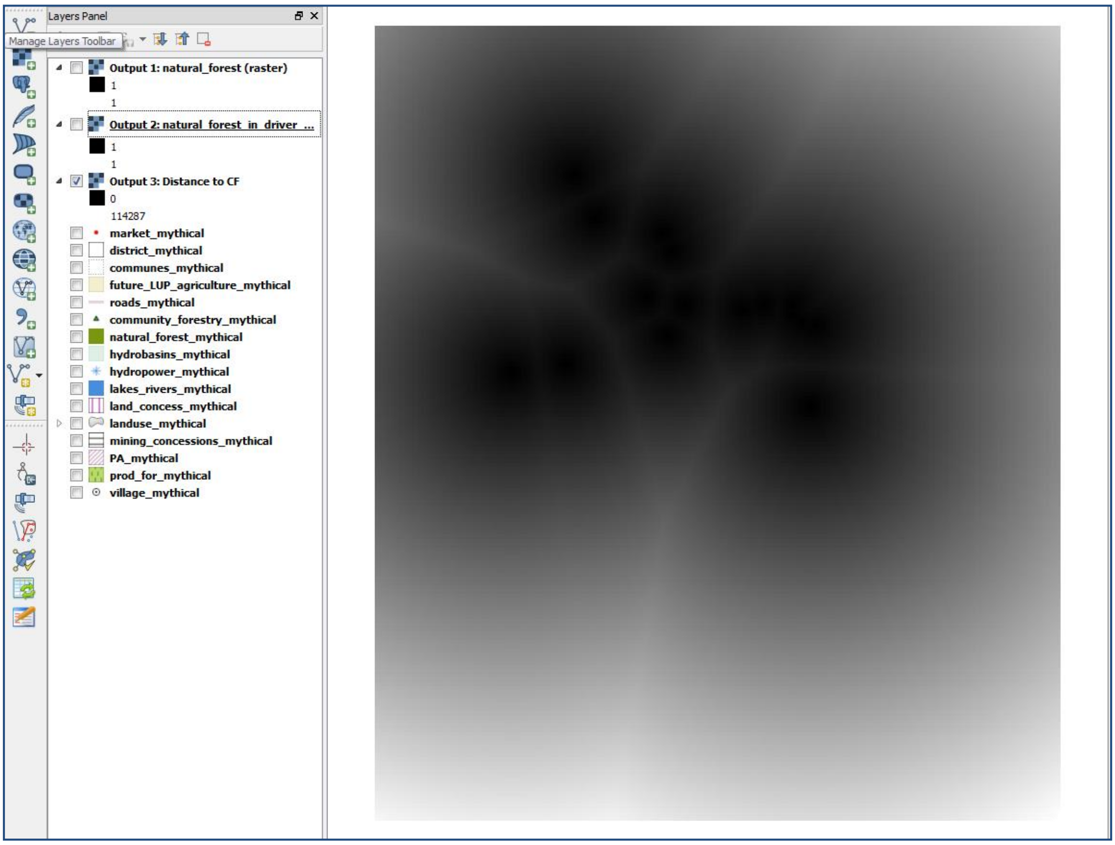

Alors qu'un flux de traitements est nécessaire pour nous guider tout au long de l'analyse, il n'est pas rare de s'apercevoir soit qu'un outil choisi se trouve être inapproprié, soit qu'une étape peut être réalisée de façon plus efficace ou plus simple. Aussi, dans QGIS il existe souvent de nombreux outils permettant de réaliser une fonction identique ou similaire, donc si un outil ne fonctionne pas, essayez-en un autre.

5.4.1.2. Distance aux villages

Regardez à nouveau votre flux de traitements pour vérifier la prochaine étape à ajouter au générateur de modèle. Il existe un autre **outil de Distance Euclidienne (Euclidean Distance)** sur la droite. Ceci générera un sortant raster lié à la distance par rapport aux villages.

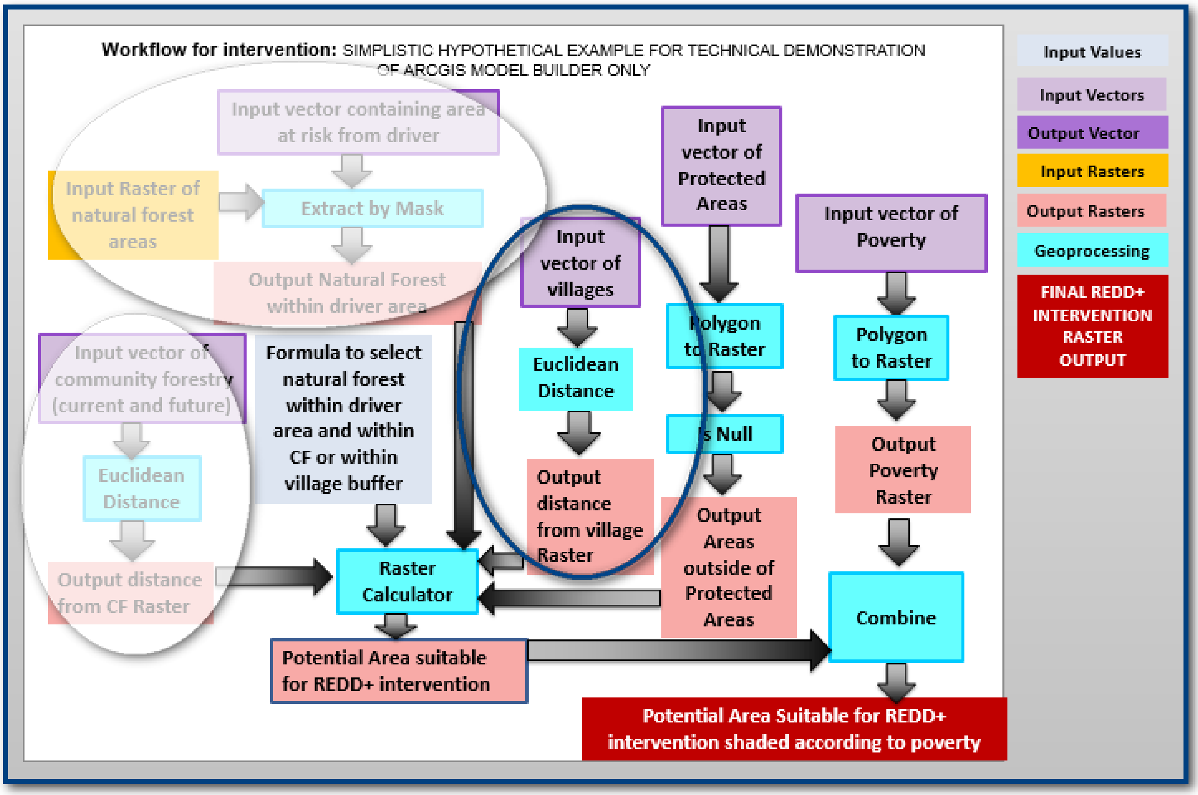

Répétez les **étapes a-x** de la **section 5.4.1.1**, mais cette fois pour générer la distance par rapport aux villages. Le sortant sera ajouté à la table des matières comme présenté ci-dessous.

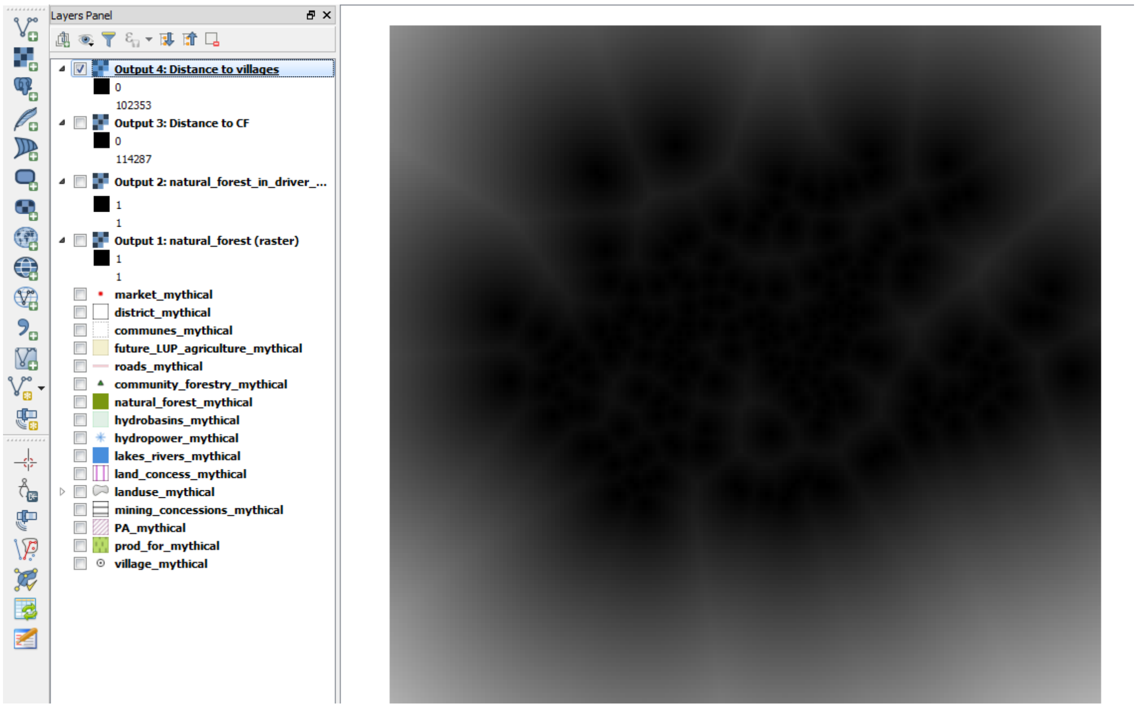

Le flux de traitements devrait à présent ressembler à l'extrait ci-dessous. Notez que les étapes 1 et 2 sont liées, les étapes 3 et 4 sont liées et que les étapes 5 et 6 le sont également mais qu'actuellement, les trois paires ne sont pas jointes.

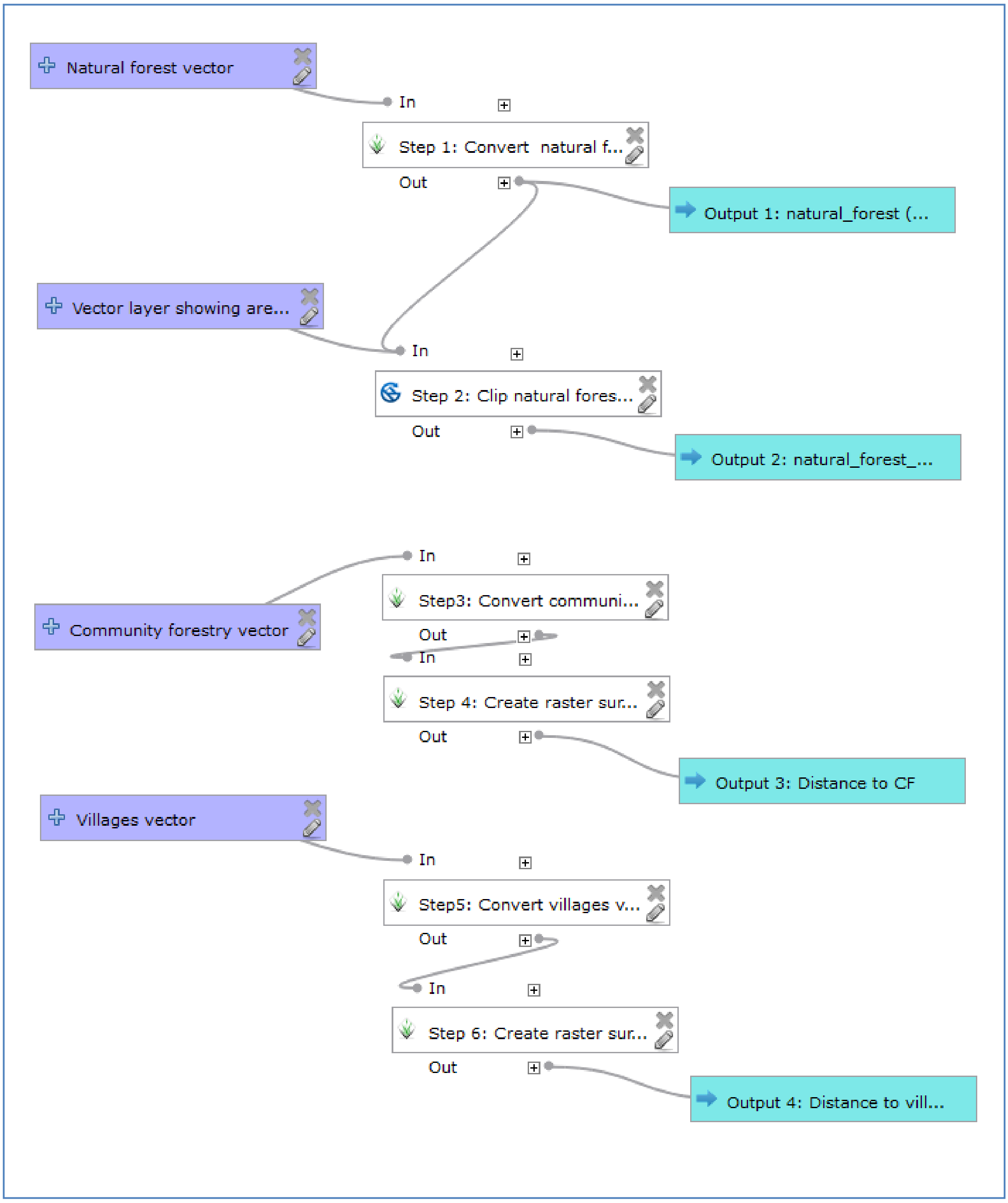

L'ordre dans lequel ces trois groupes sont exécutés importe peu, mais ils doivent être exécutés avant la prochaine étape. Celle-ci nécessite les sortants de ces trois groupes d'étapes qui se lieront donc ensemble au modèle.

5.4.1.3. Sélectionner la forêt naturelle dans l'étendue du facteur et au sein de distances spécifiques

Regardez à nouveau votre flux de traitements pour vérifier la prochaine étape à ajouter au générateur de modèle. Dans l'organigramme du flux de traitements il y a un **outil de traitement Rastor Calculator (calculatrice raster)**, qui utilisera tous les sortants de **l'extraction par masque** et des **fonctions de distance euclidienne** pour sélectionner les zones de forêt situées à la fois dans l'étendue du facteur et au sein de la distance spécifiée par rapport aux sites de sylviculture communautaire et aux villages.

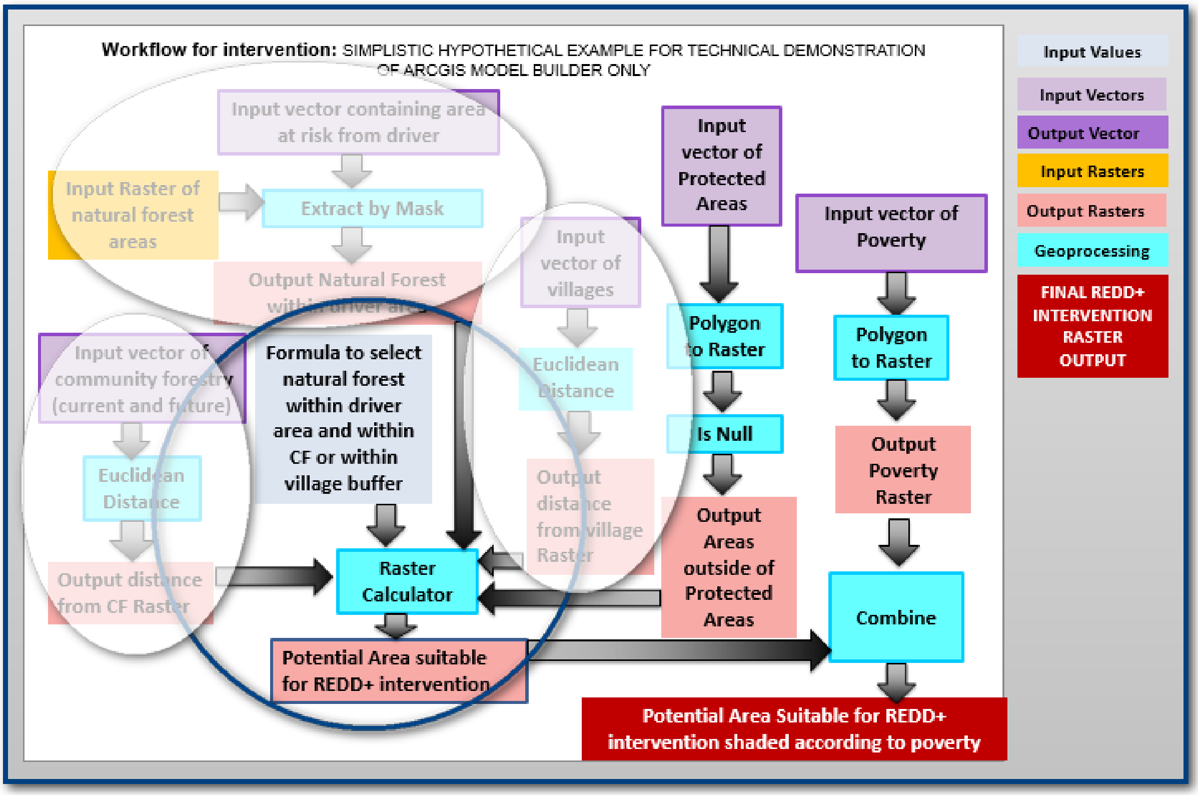

Dans QGIS, il existe trois options disponibles de **Raster Calculator** à utiliser dans le modeleur graphique : **Raster Calculator (GDAL)**, **r.mapcalculator (GRASS)** et **Raster calculator (SAGA)**.

Une expression calculatrice raster est utilisée pour sélectionner les zones nécessaires. La syntaxe varie légèrement en fonction de l'outil de calcul que vous choisissez. (Voir l'annexe 1 section A.3.5 pour une aide sur la syntaxe des **outils de calcul raster**). Dans notre exemple, nous utiliserons l'**outil (GRASS) r.mapcalculator.**

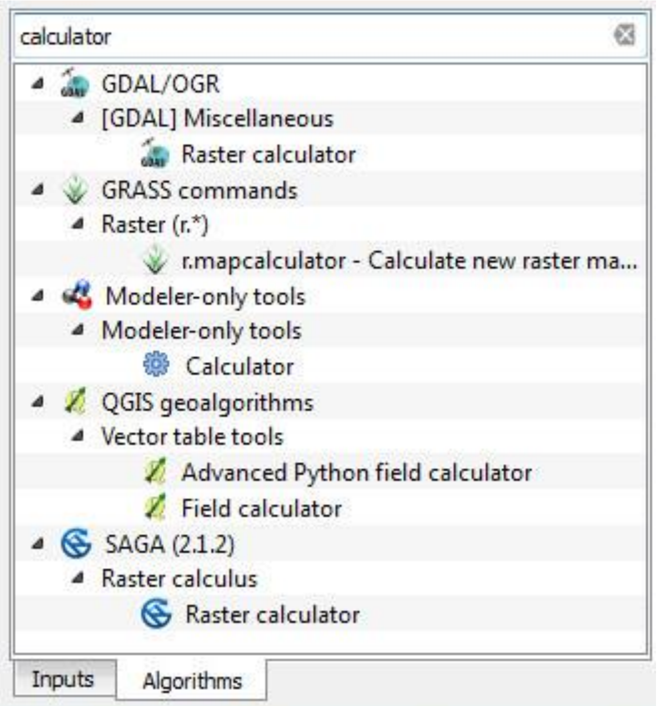

a. Cliquez sur l'onglet **Algorithmes** et cherchez **calculatrice.**

b. Double-cliquez sur **r.mapcalculator** pour ajouter l'outil dans le canevas modèle.

Nous allons utiliser la calculatrice de carte pour sélectionner les forêts au sein de l'étendue du facteur qui sont soit à 10 km ou moins des sites de sylviculture communautaire existants ou proposés ou à 3 km ou moins des villages.

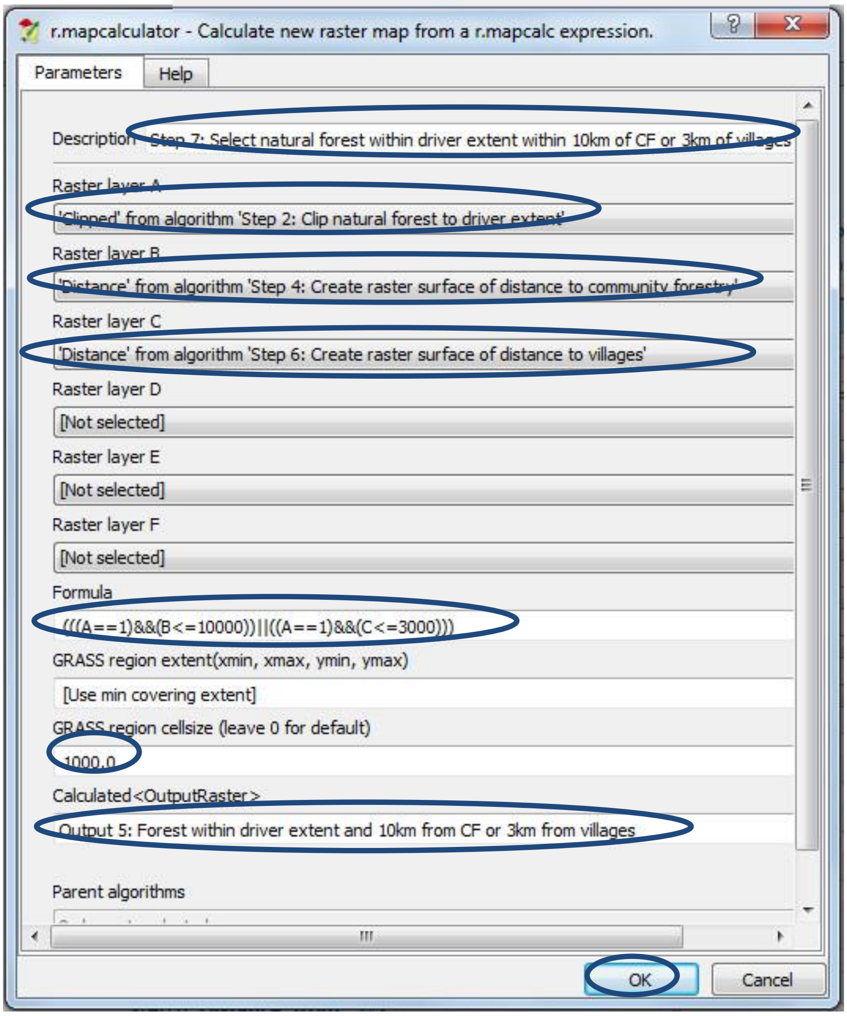

c. Changez la **description** en **Étape 7 : Sélectionner la forêt naturelle dans l'étendue du facteur et à 10 km ou moins d'un site SC ou 3 km ou moins des villages.**

d. En **Couche Raster A** sélectionnez le résultat de l'étape 2 '**Clipped from algorithm (Découpé à partir de l'algorithme) « étape 2 : Découper la forêt naturelle selon l'étendue du facteur ».**

e. En Couche Raster B sélectionnez le résultat de l'étape 4 '**Clipped from algorithm (Découpé à partir de l'algorithme) « étape 4 : Créer un raster surface lié à la distance à la sylviculture communautaire ».**

f. En Couche Raster C sélectionnez le résultat de l'étape 6 '**Clipped from algorithm (Découpé à partir de l'algorithme) « étape 6 : Créer un raster surface lié à la distance aux villages ».**

g. Dans la fenêtre de formule, indiquez l'expression ci-dessous :
(((A==1)&&(B<=10000))||((A==1)&&(C<=3000)))

Ceci signifie : « **sélectionne** dans le raster A (c.à.d. la forêt dans l'étendue du facteur) les cellules qui valent 1 et les cellules du raster B qui sont à 10 km ou moins (c.à.d. la distance aux sites de sylvicultures communautaires existants ou proposés) **OU** les cellules du raster A (c.à.d. la forêt dans l'étendue du facteur) qui valent 1 et les cellules du raster C sont à 3 km ou moins (c.à.d. distance aux villages) ». Le sortant correspond aux **zones de forêt au sein d'une bande de 10 km autour des sites de sylviculture communautaire proposés et existants, ou au sein d'une bande de 3 km autour des villages.**

h. Pour **calculated<OutputRaster> (<Outputraster> calculé)** indiquez **Sortant 5 : Forêt naturelle dans l'étendue du facteur et dans la bande de 10 km autour des sites SC ou dans la bande de 3 km autour des villages.**

*Une fois que vous avez ajouté la nouvelle étape, n'oubliez pas de cliquer sur le bouton **éditeur d'aide du modèle** pour ajouter votre description correspondant à cette nouvelle étape. N'oubliez pas de sauvegarder votre modèle.*

Les trois groupes d'étapes sont maintenant liés. Le modèle devrait maintenant être similaire à l'illustration ci-dessous :

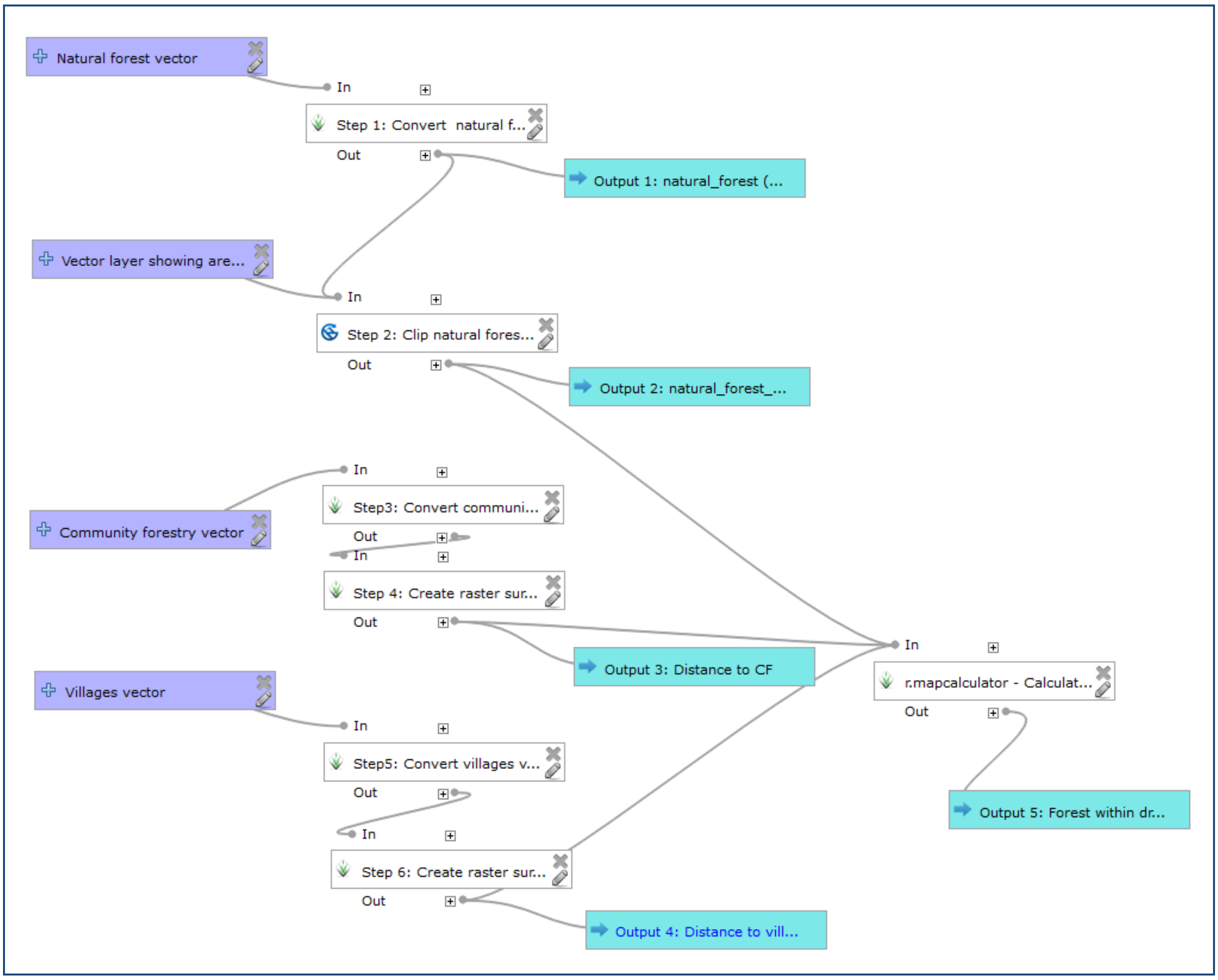

5.4.1.4. Exclure les aires strictement protégées

Vérifiez le modèle au niveau de la liste de jeux de données que nous avons identifiés au début ainsi que le flux de traitement.

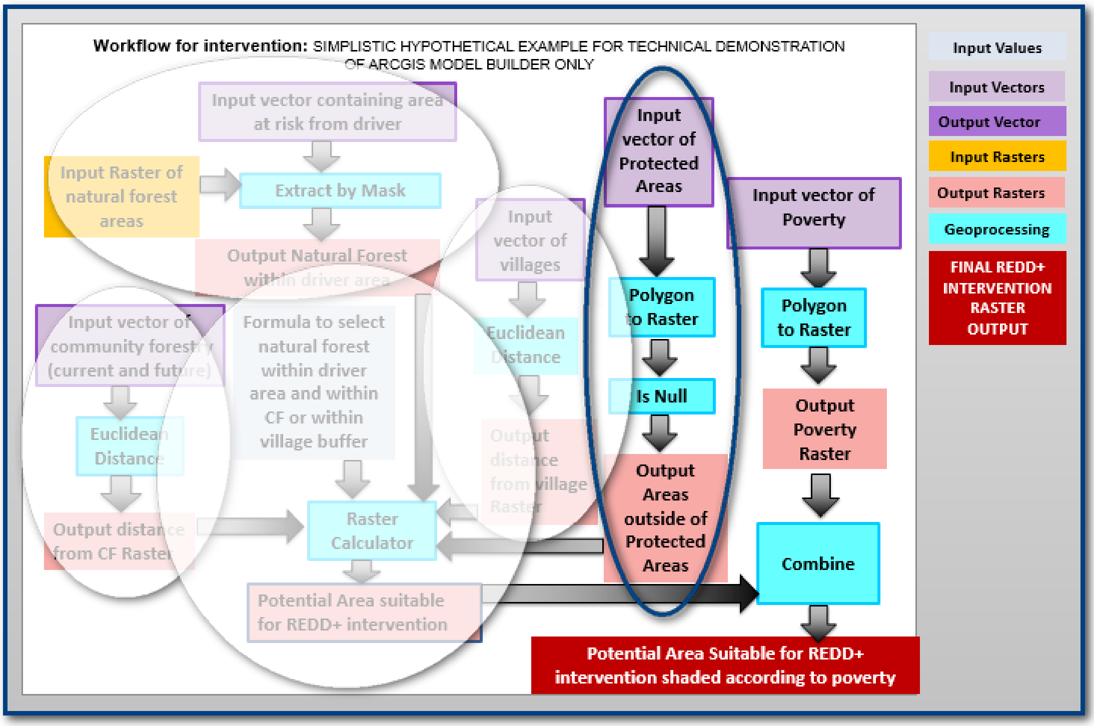

En l'état, le modèle permet de réaliser les quatre premières parties du flux de traitements et il en reste encore deux. La prochaine étape à ajouter à votre modèle consiste à « **exclure les aires strictement protégées** ».

a. Les aires protégées sont une couche vectorielle, il faut donc ajouter une **couche vectorielle (Vector Layer)** et une **v.to.rast.value** au **modeleur de traitement**.
b. Changez la description en **Étape 8 : Convertir le vecteur aires protégées en raster.**
c. Sélectionnez le paramètre **Aires protégées** en tant que couche **Input Vector (vecteur entrant)**.
d. Réglez la **taille de la cellule**, par exemple sur **1000.**
e. Cliquez **sur OK.**

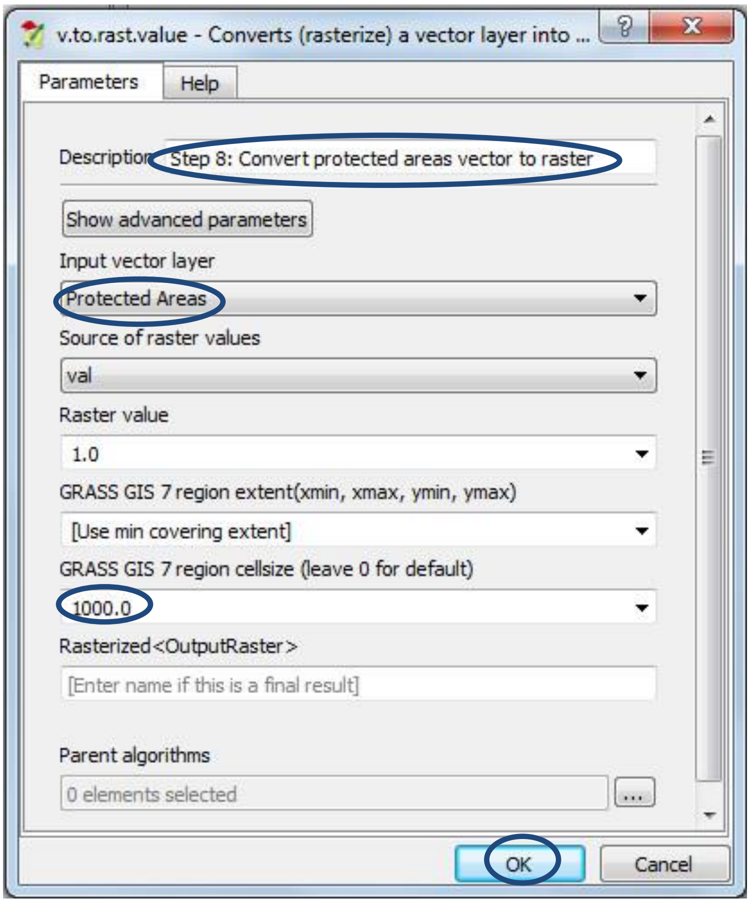

*Une fois que vous avez ajouté la nouvelle étape, n'oubliez pas de cliquer sur le bouton **éditeur d'aide du modèle** pour ajouter votre description correspondant à cette nouvelle étape. N'oubliez pas de sauvegarder votre modèle.*

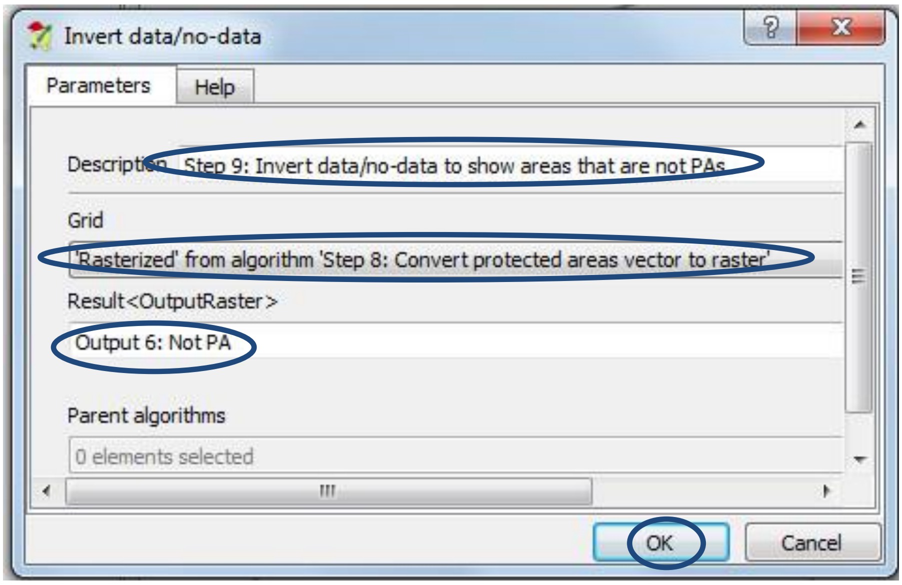

f. Pour sélectionner les zones qui ne sont pas strictement sous protection, ajoutez un outil **Invert data/nodata** de **(SAGA)** dans le **modeleur de traitement.**
g. Changez la description en **Étape 9 : Inverser données/no data pour montrer les zones qui ne sont pas des AP.**

h. Sélectionnez le **« Rasterized » créé par l'algorithme « étape 8 : Convertir le vecteur aires protégées en raster »** pour la Grille.
i. Dans **Result<OutputRaster> (<Outputraster> résultat)** indiquez **Sortant 6 : zone hors AP.**
j. Cliquez sur **OK.**

Par la suite nous utiliserons le sortant 6 dans une autre expression **r.mapcalculator** pour sélectionner les zones de forêt naturelle au sein de l'étendue du facteur et dans la bande de 10 km autour des sites SC et de la bande de 3 km autour des villages.

k. Changez la description en **Étape 10 : Sélectionner la forêt naturelle dans l'étendue du facteur et à 10 km ou moins d'un site SC ou 3 km ou moins de villages et qui n'est pas une AP.**
l. Sélectionnez **« Calculated (calculé) » créé par l'algorithme « étape 7 : Sélectionner la forêt naturelle dans l'étendue du facteur et à 10 km ou moins d'un site SC ou 3 km ou moins de villages »** pour le raster A.
m. Sélectionnez le **« Result » créé par l'algorithme « étape 9 : Inverser données/no data pour montrer les zones qui ne sont pas des AP »** pour le raster B.
n. Dans la fenêtre de formule indiquez **(A==1)&&(B==1)**

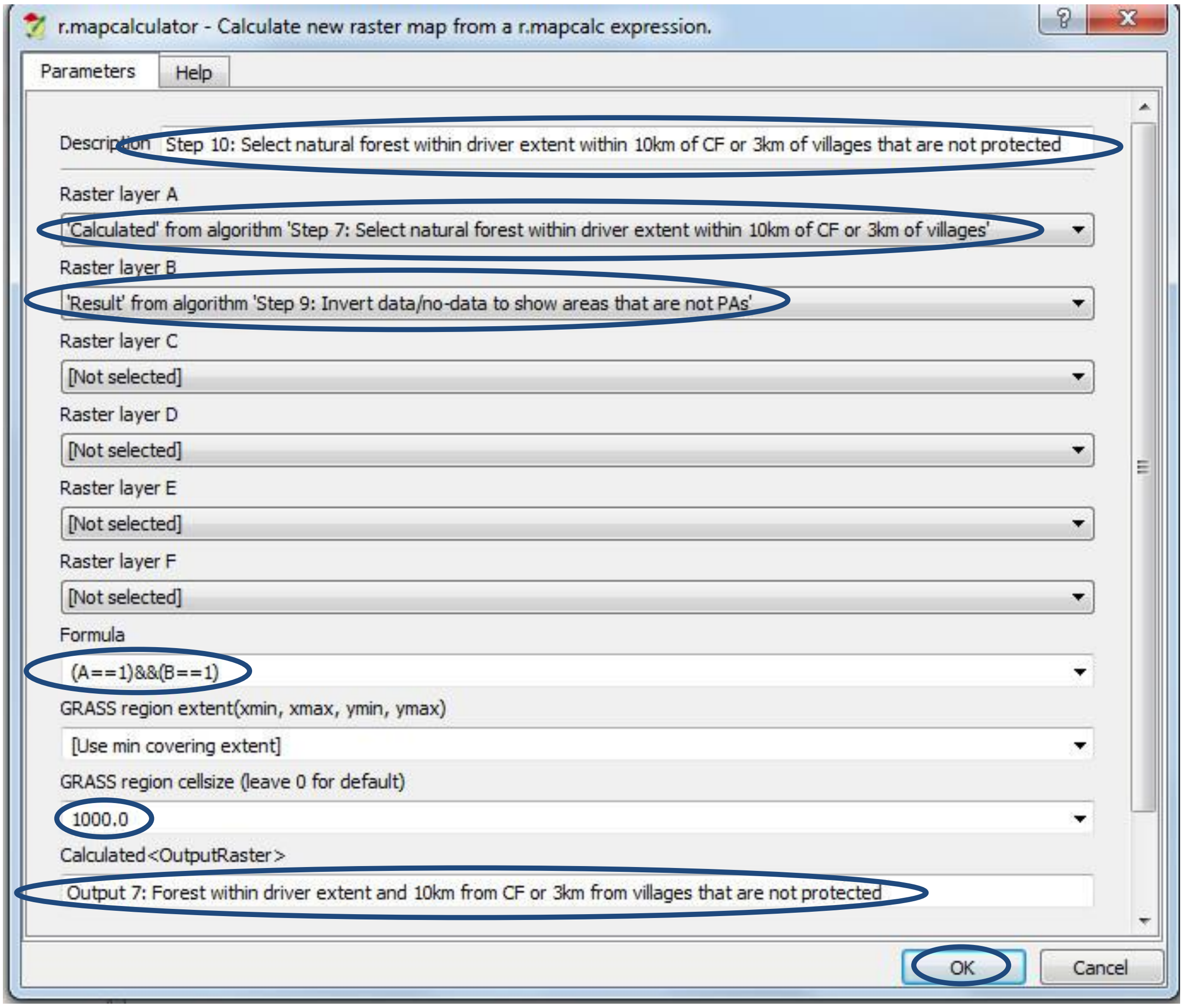

o. Réglez la **taille de la cellule**, par exemple sur **1000.**
p. Pour **Calculated<OutputRaster> (<Outputraster> calculé)** indiquez **Sortant 7 : Forêt dans l'étendue du facteur à 10 km ou moins d'un site SC ou 3 km ou moins de villages et qui n'est pas une AP.**
q. Cliquez **sur OK.**

*Une fois que vous avez ajouté la nouvelle étape, n'oubliez pas de cliquer sur le bouton **éditeur d'aide du modèle** pour ajouter votre description correspondant à cette nouvelle étape. N'oubliez pas de sauvegarder votre modèle.*

La légende devrait ressembler à ceci :

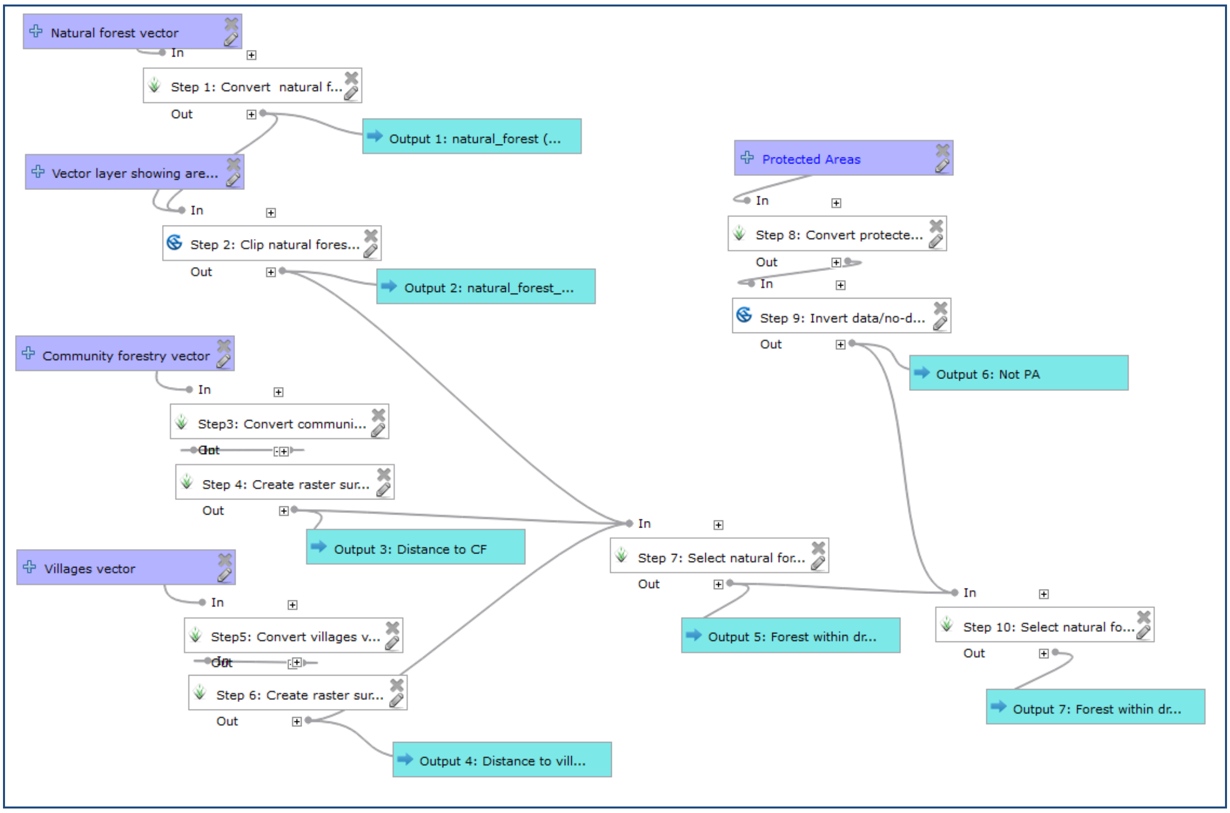

5.4.1.5. Identifier les zones prioritaires en fonction de la pauvreté

Il y a une dernière étape dans le flux de traitement original : il s'agit de classifier les zones sélectionnées afin qu'elles puissent être priorisées en fonction de la pauvreté. Ceci permettra de préciser l'un des bénéfices supplémentaires possibles du REDD+ :

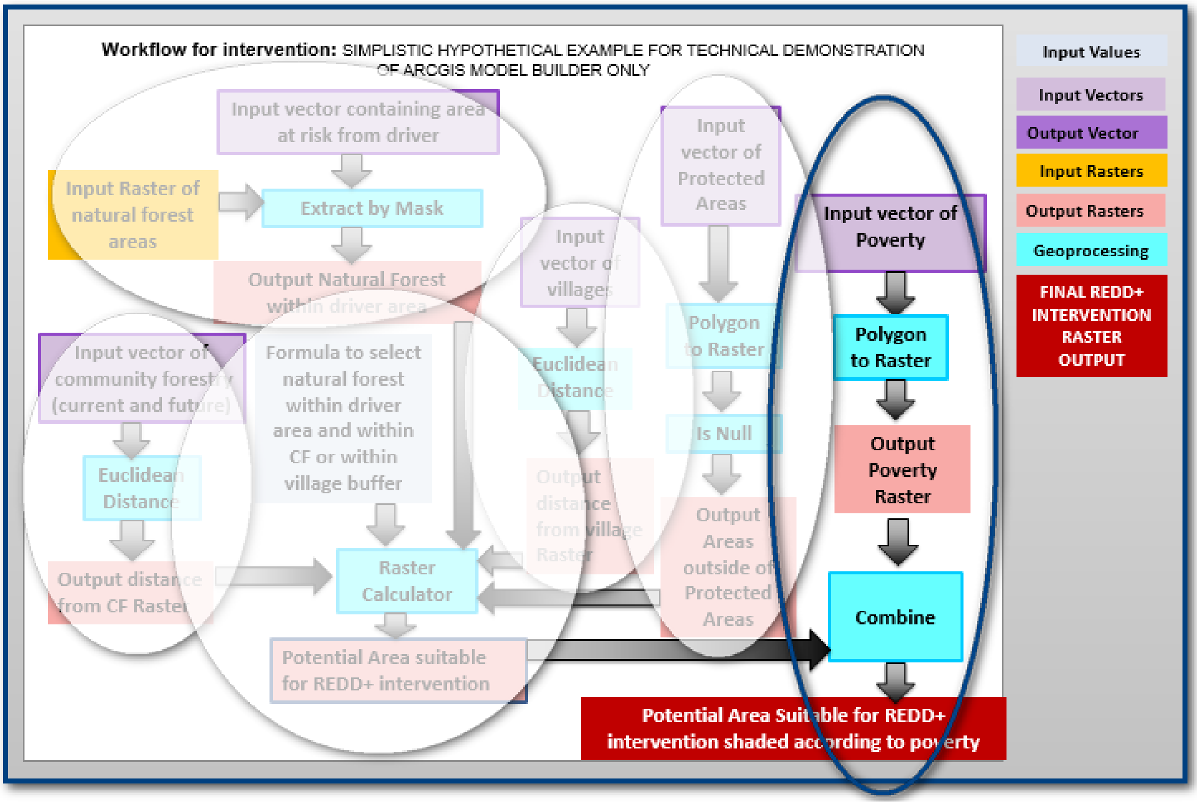

Il est important de noter qu'il n'y a pas d'outil de **Combinaison** de raster dans QGIS mais, à nouveau, nous pouvons utiliser l'outil **r.mapcalculator** pour classifier la couche **zones de forêt naturelle dans l'étendue du facteur dans la bande de 10 km autour des sites SC ou dans la bande de 3 km autour des villages et zones qui ne sont pas des aires protégées** en matières de pauvreté.

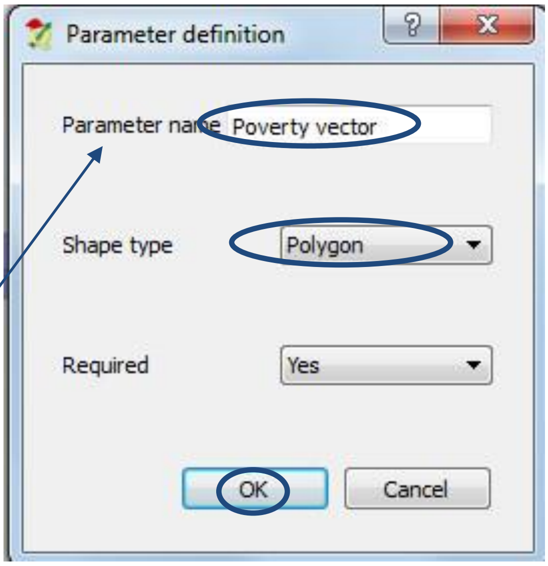

a. L'information concernant la pauvreté dans notre exemple est un attribut du jeu de données vectorielles **communes_mythical**, nous pouvons donc ajouter une nouvelle **couche vectorielle** appelée ex. **vecteur Pauvreté** au générateur de traitement.
b. Par la suite, ajoutez un **champ de table** appelé par exemple, **attribut pauvreté** et liez-le à la **couche parente vecteur pauvreté** puisque cette fois nous voulons utiliser le champ pauvreté pour réaliser notre jeu de données raster plutôt que de mettre toutes les valeurs dans le raster 1.

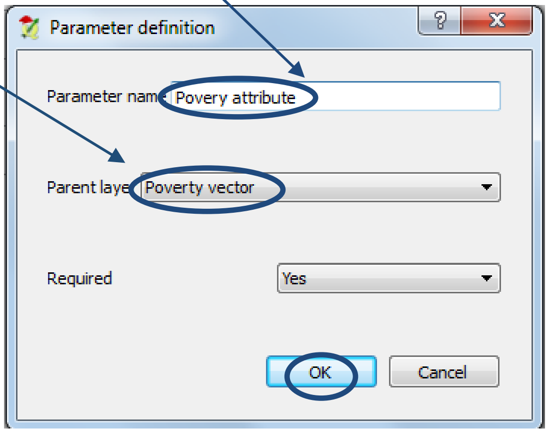

c. Ajoutez un nouvel Algorithme **v.to.rast.attribute** au **générateur de traitement**.
d. Changez la description en **Étape 11 : Convertir le vecteur pauvreté en raster.**
e. Sélectionnez le paramètre **Vecteur pauvreté** en tant que couche **Input Vector (vecteur entrant).**
f. Sélectionnez le paramètre **attribut pauvreté** comme **Name of column for 'attr' parameter (Nom de colonne pour le paramètre « attr »).**
g. Réglez la **taille de la cellule**, par exemple sur **1000.**
h. Cliquez sur **OK.**

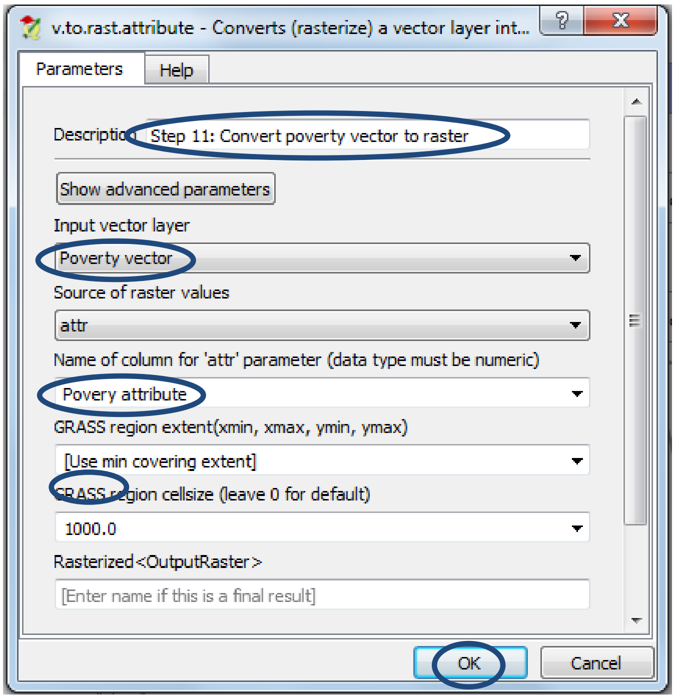

i. Ajoutez un nouvel **Algorithm r.mapcalculator** au **générateur de traitement.**

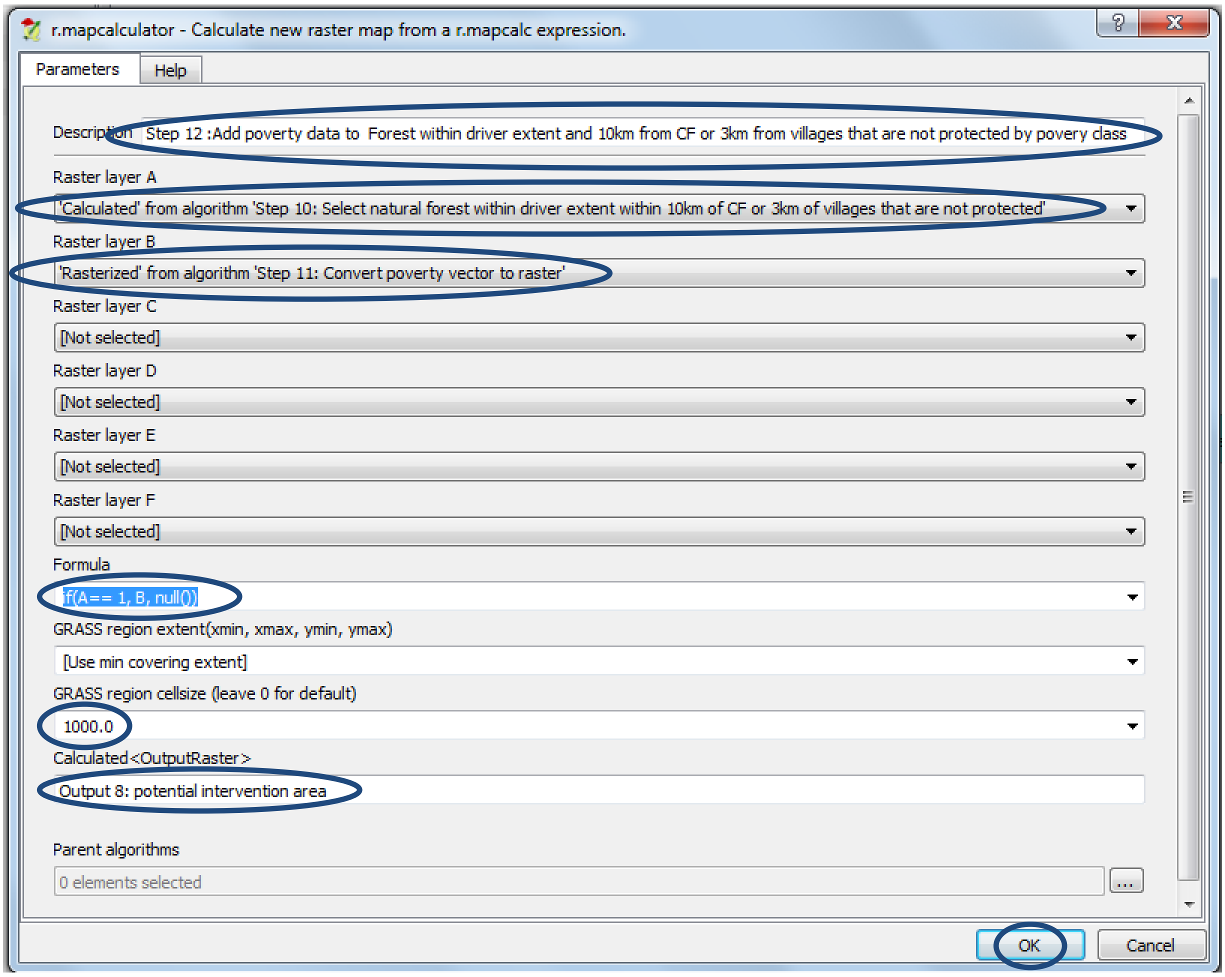

j. Rédigez une **description** pour l'étape**, par exemple étape 12 : Ajouter les données pauvreté à la forêt dans l'étendue du facteur et à 10 km ou moins d'un site SC ou 3 km ou moins d'un village qui n'est pas protégé par une classe de pauvreté.**

k. Choisissez le **résultat de l'étape 10** pour le **Raster A.**

l. Choisissez le **résultat de l'étape 11** pour le **Raster B.**

m. Dans la **fenêtre de formule** indiquez **if(A== 1, B, null())**

Ceci signifie : « Les valeurs dans A égales à 1 deviennent les valeurs de pauvreté dans le sortant ; les autres valeurs dans A deviennent des valeurs Nulles dans le sortant »

n. Cliquez **sur OK.**

Le modèle est maintenant terminé et l'interface d'utilisation devrait être similaire à ceci :

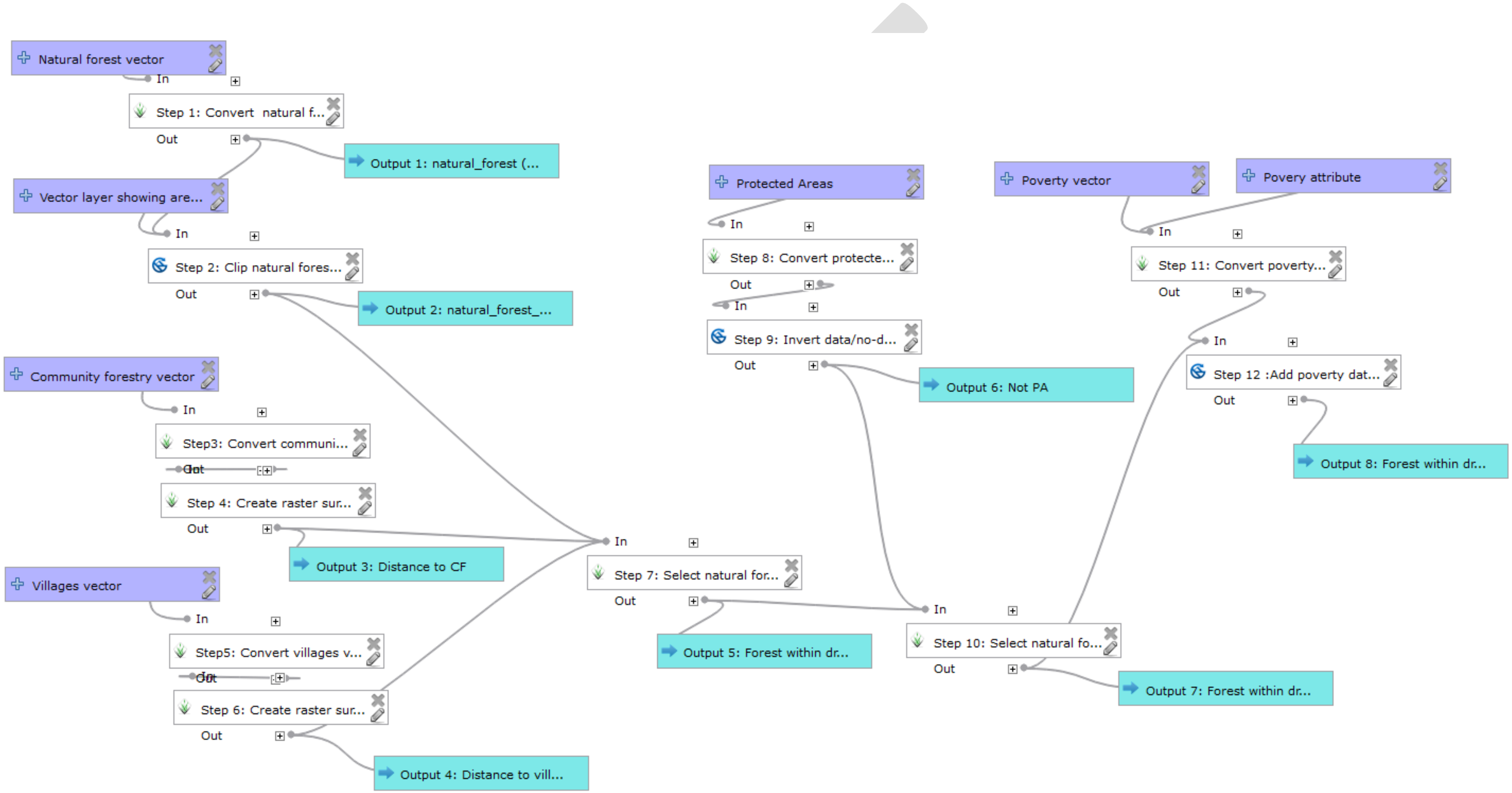

o. **Enregistrez** et **fermez** le **modeleur de traitement.**

5.5. Paramétrer la symbologie pour les sortants du modèle

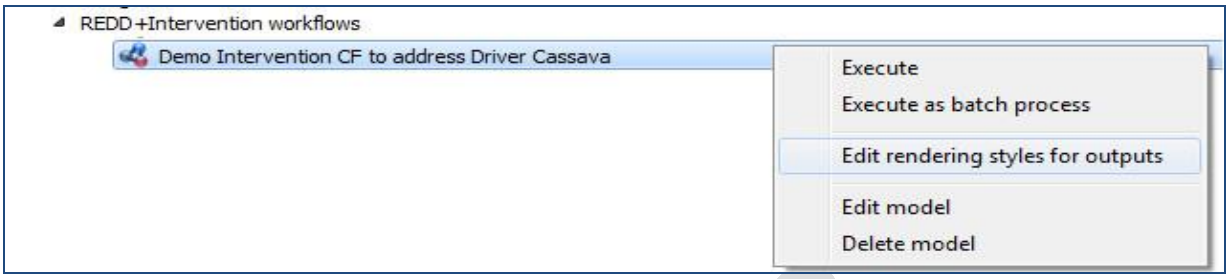

Lorsque le sortant de l'outil est ajouté dans la table des matières, une symbologie par défaut est utilisée. Vous pouvez pré-paramétrer la Symbologie pour vos résultats. Dans votre modèle, faites un clic droit sur la **Classe d'entité en Sortie** et sélectionnez **Éditer les styles de rendu par défaut**. Vous pouvez attribuer un fichier de style prédéfini de QGIS pour déterminer la symbologie tant que vous connaissez la gamme des valeurs sortantes, c.à.d. tant que vous connaissez les valeurs minimum et maximum. Par exemple, pour les traitements qui produisent des sortants booléens, vous savez que les valeurs sortantes seront soit 1 ou 0 ; ainsi, la symbologie peut être créée pour afficher les 1 et 0 dans des couleurs choisies. Pour certains traitements, vous ne connaitrez les valeurs minimum et maximum du fichier sortant que lorsque le traitement sera exécuté ; dans ces conditions, un style prédéfini ne peut pas être sélectionné.
Dans cet exemple, des fichiers style prédéfinis ont été créés pour tous les sortants.

a. Le sortant 1 donne un sortant contenant des valeurs de 1 ou des valeurs no data. Cliquez sur … pour charger le fichier style pour le Sortant 1 **dark green raster 1_nodata.qml.**

b. Le sortant 2 donne un sortant contenant des valeurs de 1 ou des valeurs no data. Cliquez sur … pour charger le fichier style pour le Sortant 2 **light green raster 1_nodata.qml.**

c. Le sortant 3 donne un sortant avec une gamme de valeurs de distance. Nous voulons seulement savoir si les zones sont au-dessus ou en-dessous de 3 km afin qu'un fichier style soit téléchargé et nous n'avons pas besoin de connaître la valeur maximum dans le fichier. Cliquez sur … pour charger le fichier style pour le Sortant 3 **cream_to_pink_0 to 3 km_dist.qml.**

d. Le sortant 4 donne un sortant avec une gamme de valeurs de distance ; cette fois nous nous intéressons seulement au fait que les zones soient au-dessus ou en-dessous de 10 km. Cliquez sur … pour charger le fichier style pour le Sortant 4 **cream_to_blue_0 to 10 km_dist.qml.**

e. Le sortant 5 donne un sortant contenant des valeurs valant 1 ou 0. Cliquez sur … pour charger le fichier style pour le Sortant 5 **brown green raster 1_0.qml.**

f. Le sortant 6 donne un sortant contenant des valeurs de 1 ou des valeurs no data. Cliquez sur … pour charger le fichier style pour le Sortant 6 **grey raster 1_nodata.qml.**

g. Le sortant 7 donne un sortant contenant des valeurs valant 1 ou 0. Cliquez sur … pour charger le fichier style pour le Sortant 7 **orange raster 1_0.qml.**

h. Le sortant 8 donne un sortant contenant des valeurs valant 1, 2, 3 ou des valeurs no data. Cliquez sur … pour charger le fichier style pour le Sortant 8 **yellow_orange_red raster 123_nodata.qml.**

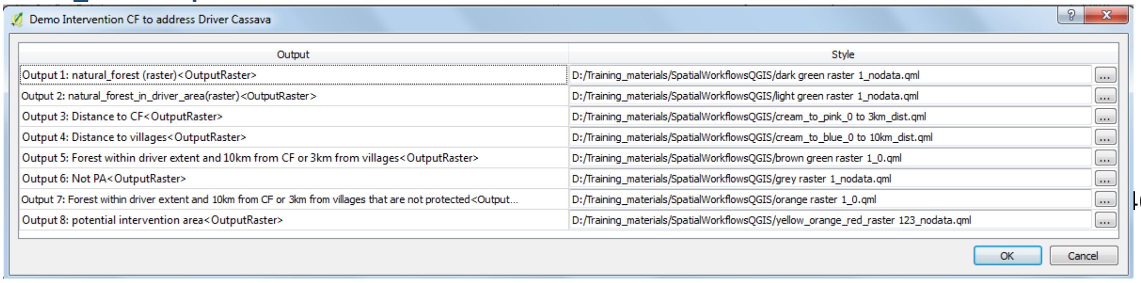

5.6. Exécuter le modèle en entier et vérifier les résultats

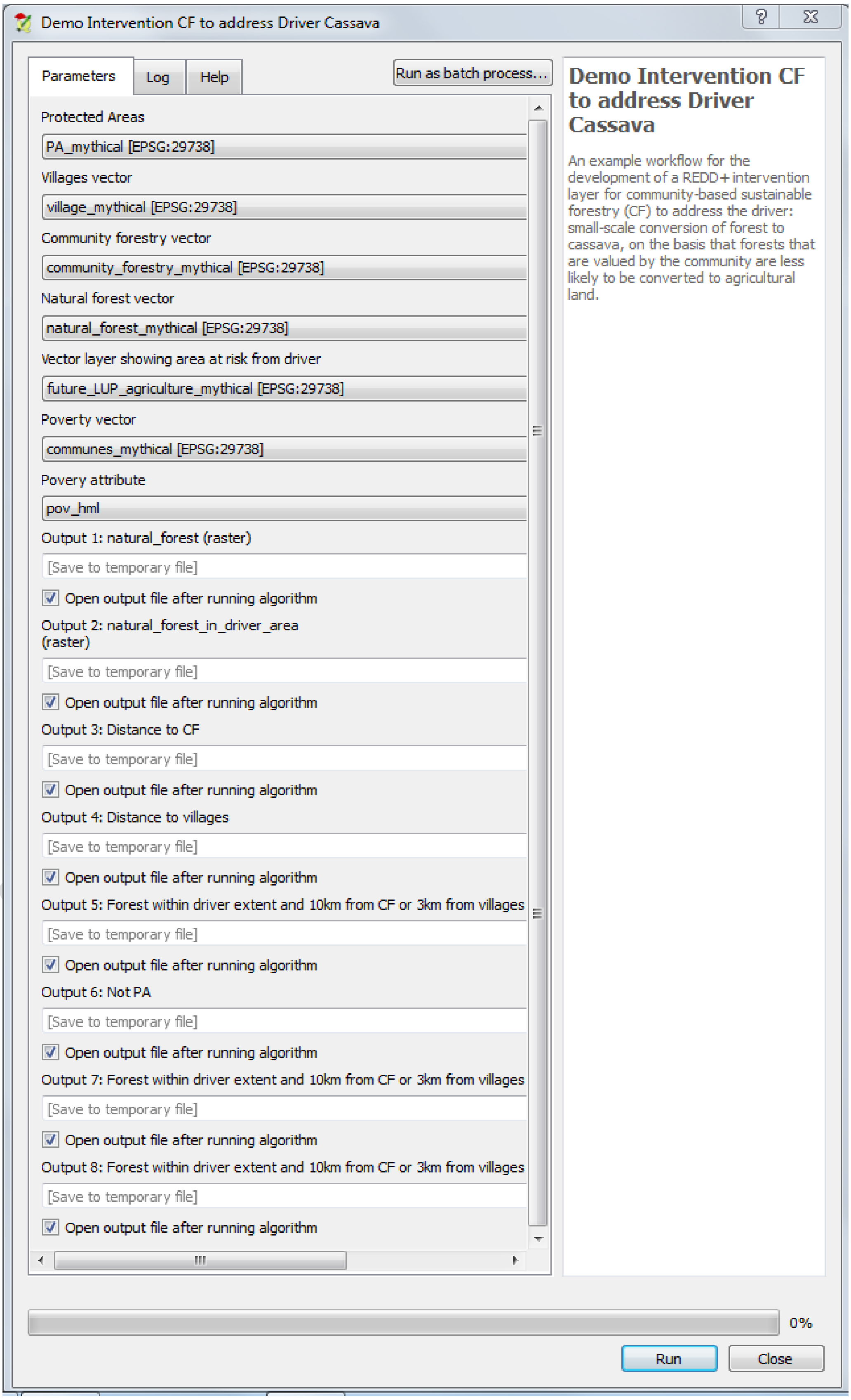

p. **Double-cliquez** sur le modèle dans la **Boîte à outils de traitements** et cliquez sur **Run (Lancer le modèle)** pour exécuter le modèle.

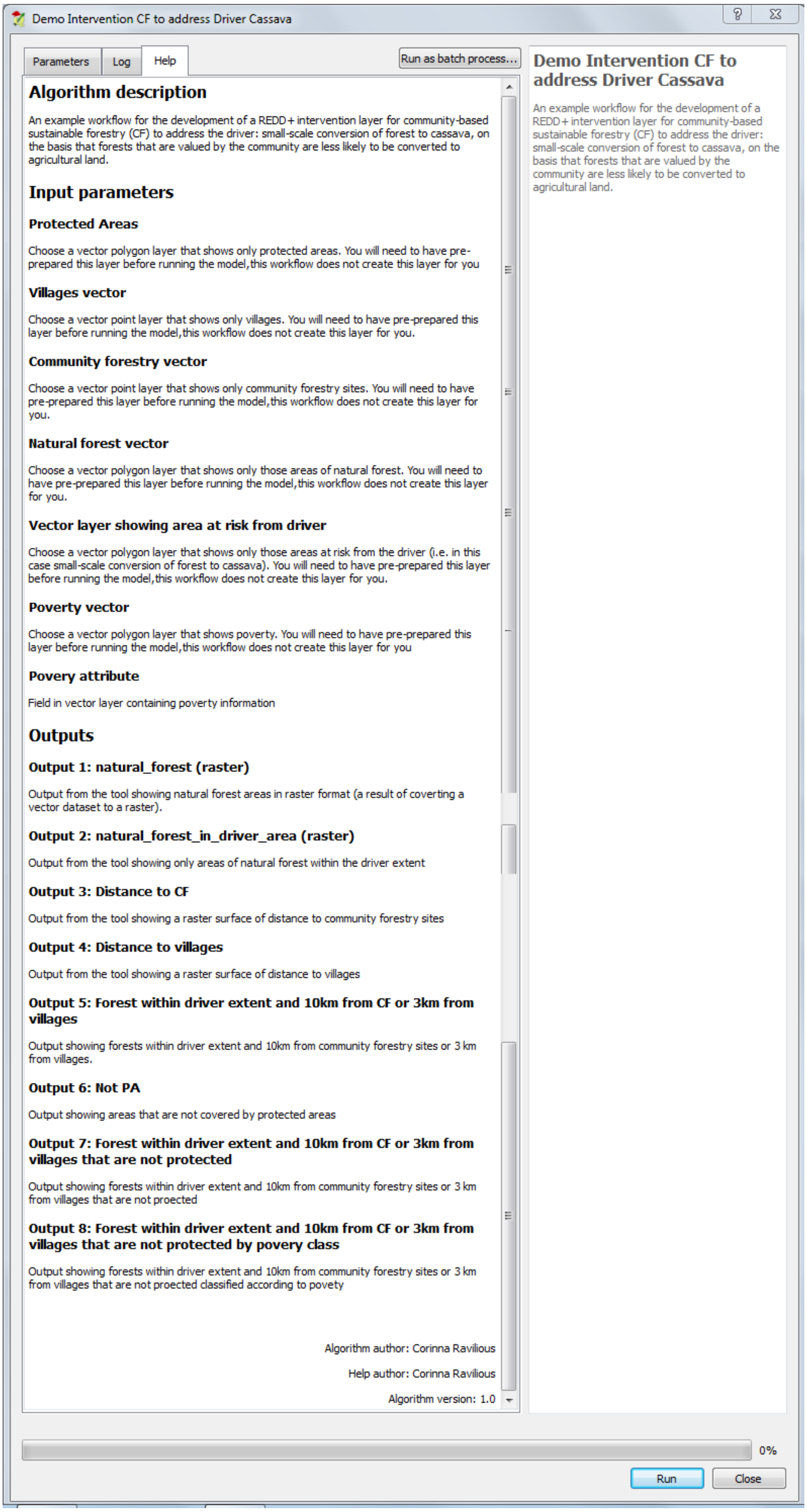

q. **Cliquez** sur **Aide** pour voir les aides de chacun des entrants.

r. Cliquez sur l'onglet **Paramètres** pour retourner à la fenêtre de dialogue.

s. Remplissez les paramètres concernant les **entrants et les sortants** comme dans l'illustration ci-dessous.

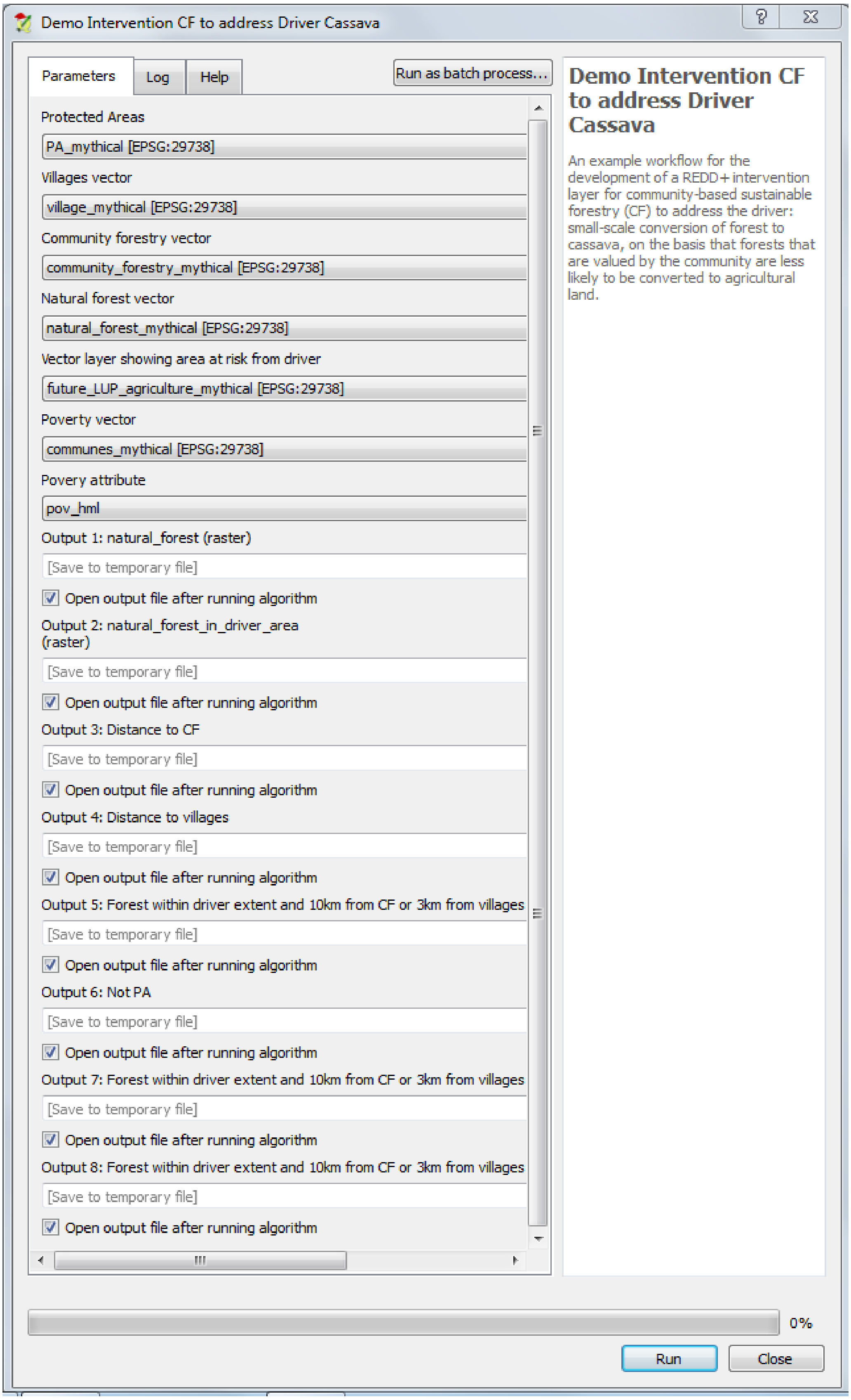

t. Cliquez sur Run pour **Lancer** l'outil.

*****Il est IMPORTANT de vérifier les sortants du Modèle pour vous assurer qu'il n'y a pas d'erreur dans les paramètres. Le fait que le modèle ait fonctionné ne signifie pas qu'il ait donné les résultats attendus.*****

Par exemple, dans notre modèle de démonstration ci-dessous :

- Le sortant 1 correspond à la couche raster forêt naturelle (vert). Nous pouvons voir qu'il couvre correctement l'étendue totale du district et qu'il y a des zones blanches **no data** où il n'y a pas de forêt naturelle.

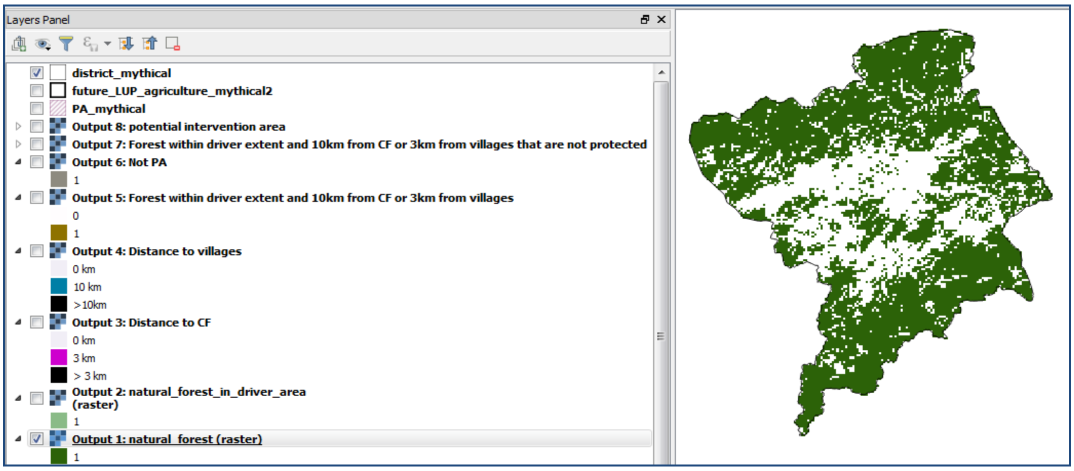

- Le sortant 2 correspond à la forêt naturelle dans l'étendue du facteur. Nous pouvons voir qu'il couvre correctement l'étendue totale de la zone du facteur et qu'il y a des zones blanches **no data** où il n'y a pas de forêt naturelle.

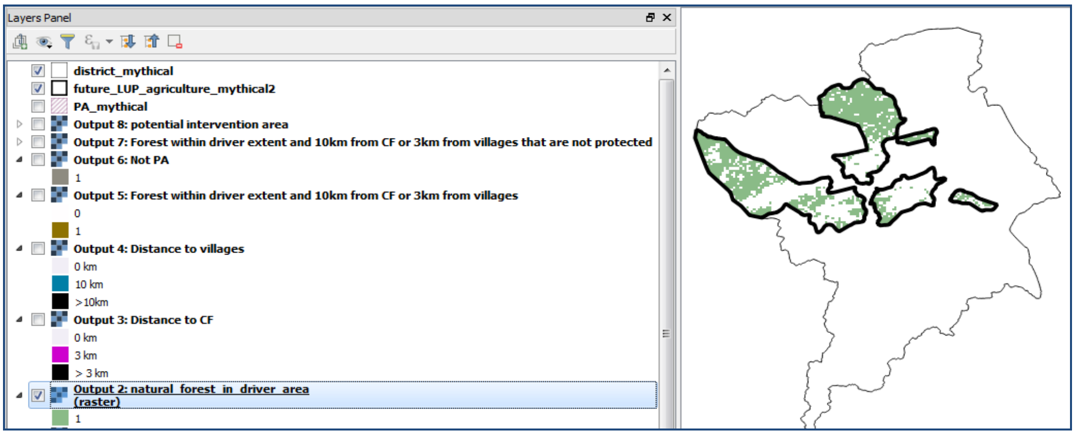

- Le sortant 3 correspond à la distance euclidienne par rapport aux sites de sylviculture communautaire. Nous pouvons voir qu'il couvre correctement l'étendue totale du district, mais qu'il s'étale aussi au-delà du district. Ceci n'est pas important pour cette étape, tant qu'il couvre la totalité du district. Le noir semble gris puisqu'une transparence a été établie dans la symbologie du sortant.

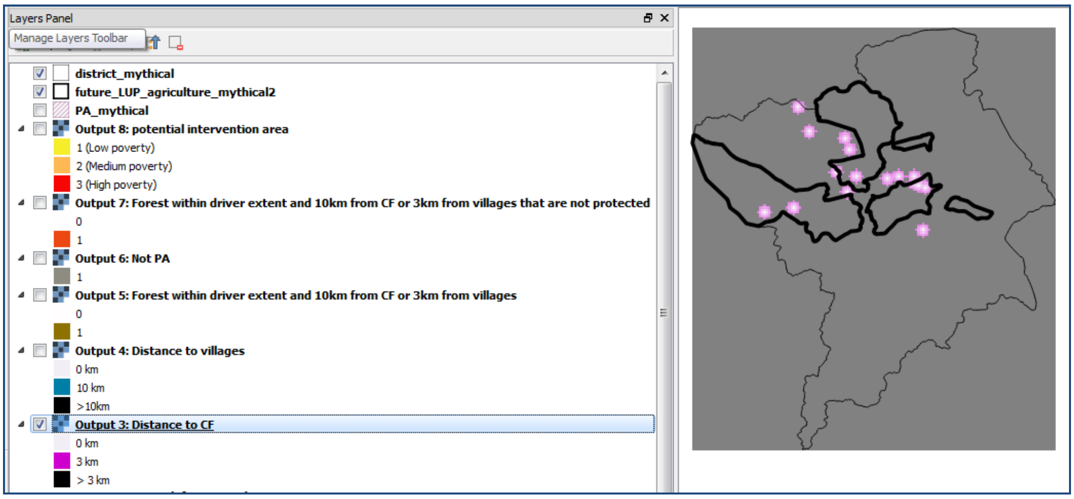

- Le sortant 4 correspond à la distance euclidienne par rapport aux villages. Encore une fois, nous pouvons voir qu'il couvre correctement l'étendue totale du district, mais qu'il s'étale aussi au-delà du district. Ceci n'est pas important pour cette étape, tant qu'il couvre la totalité du district. Le noir semble gris puisqu'une transparence a été établie dans la symbologie du sortant.

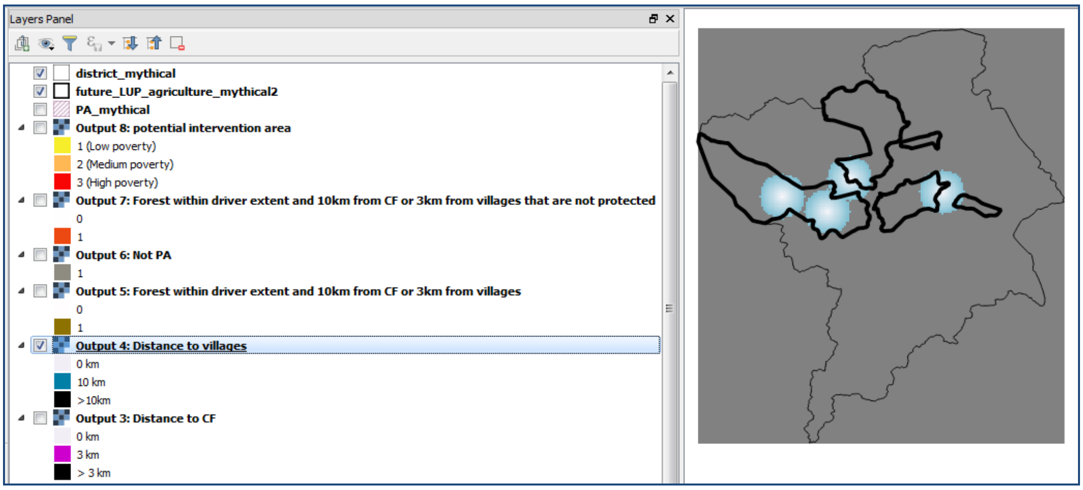

- Le sortant 5 montre les **zones marron** qui sont les zones qui ont été sélectionnées comme zones de forêt naturelle, situées dans la bande de 10 km autour des sites de sylviculture communautaire existants et proposés ou dans la bande de 3 km autour des villages. Les **zones blanches** sont les zones qui ont été exclues dans cette étape.

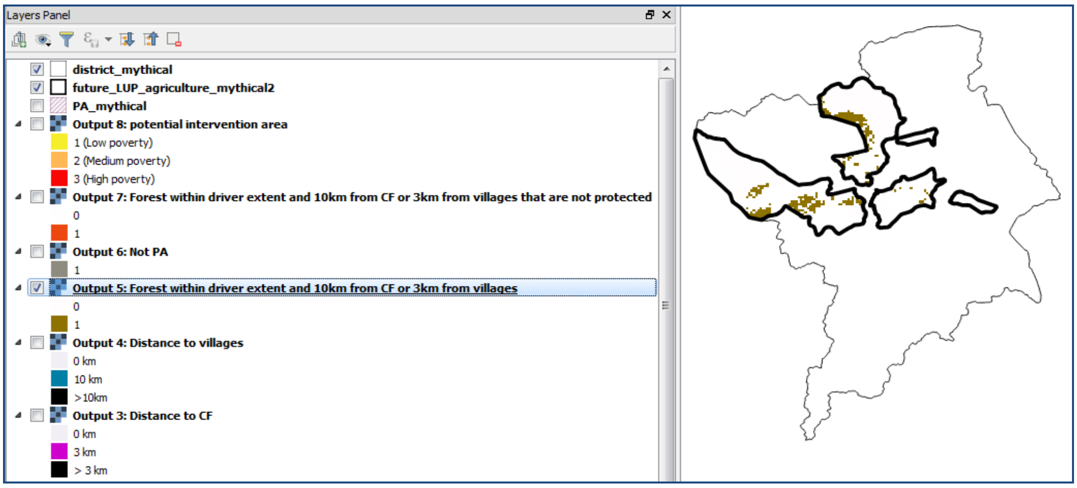

- Le sortant 6 montre des **zones grises** qui ne sont pas protégées et **des zones blanches no data** qui sont protégées.

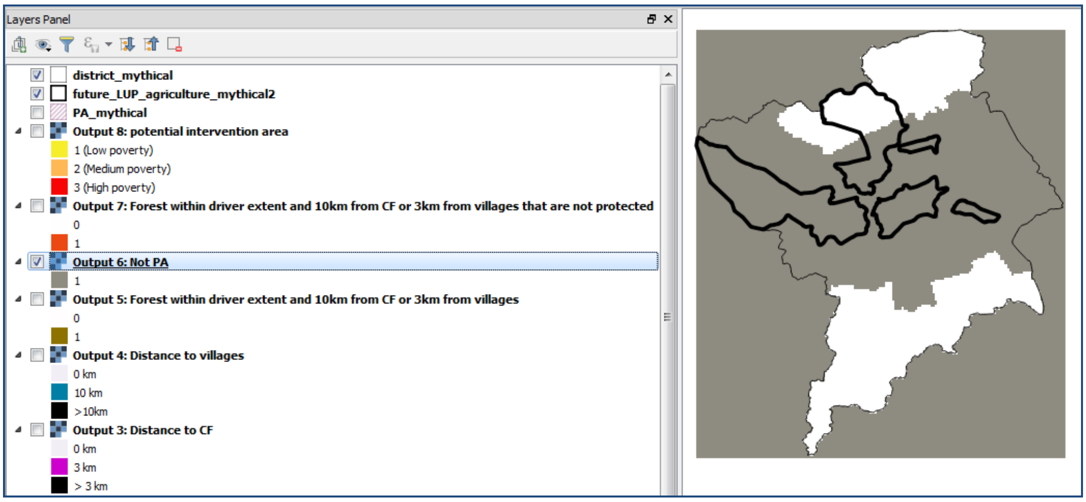

- Le sortant 7 montre les **zones d'intervention potentielles orange**, ces zones ont été sélectionnées comme zones de forêt naturelle, qui sont situées dans la bande de 10 km autour des sites de sylviculture communautaire existants et proposés ou dans la bande de 3 km autour des villages et hors des aires protégées.

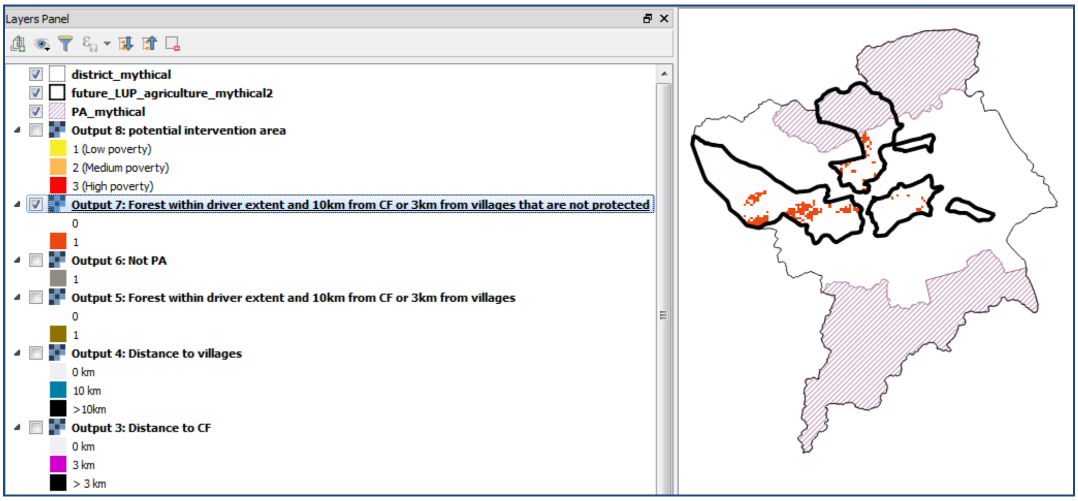

- Enfin, si nous regardons la dernière couche, Sortant 8, nous pouvons nuancer **ces zones d'intervention potentielles par classe de pauvreté.** Vous verrez que la classe de pauvreté montre des valeurs de 1 à 3 dans cet exemple ;
  1 = faible, 2 = moyen et 3 = élevé.

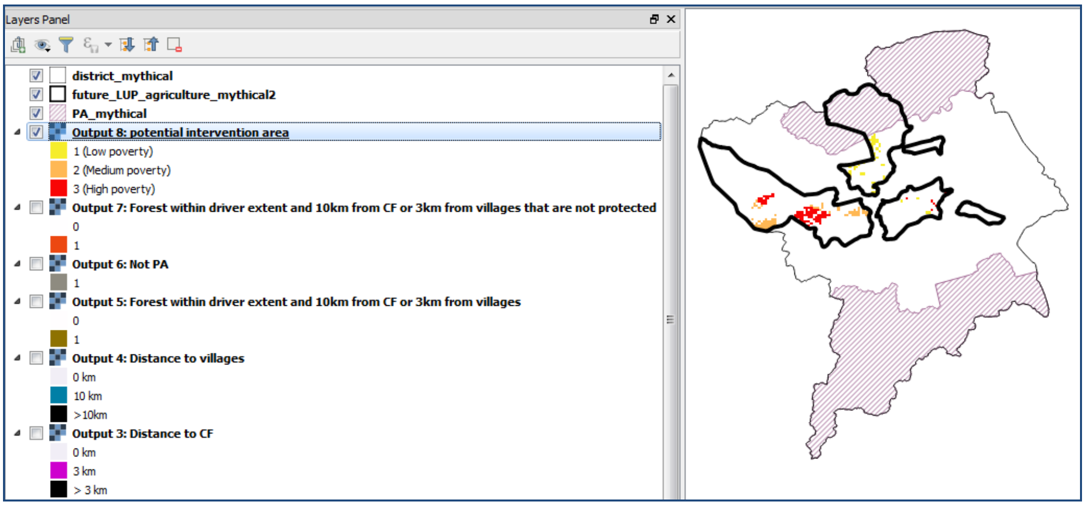

5.7. Partager un modèle QGIS avec d'autres utilisateurs

Le moyen le plus facile de partager un outil que vous avez créé consiste à **simplement copier le fichier modèle en utilisant l'explorateur Windows**, de la même façon que vous le feriez pour un autre fichier. L'emplacement par défaut pour le stockage du modèle est C:\Users\xxxxxxxx\.qgis2\processing\models.

Certains outils personnalisés ne fonctionnent plus lors d'un changement de version de QGIS, souvent parce qu'un outil de géotraitement a changé ou fonctionne de façon légèrement différente par rapport aux versions précédentes. Dans ces cas, il peut être nécessaire de sélectionner et d'effacer cette étape dans la fenêtre du modeleur de traitement et de la faire glisser dans une nouvelle version de l'outil.

A Annexe 1 : Notes de conseil sur l'analyse raster et les outils de géotraitement dans ArcGIS et QGIS

A.1. Vecteur vs raster

A.1.1. Différence entre les jeux de données vectoriels et raster

Les données peuvent être divisées en deux types généraux : **Les données discrètes**, qui représentent des objets et qui peuvent être stockées soit comme **entités vectorielles ou comme raster**, et **les données continues**, qui sont généralement (et préférablement) stockées comme **données au format raster.** Voir le tableau 1 pour plus d'informations sur les différences entre les données vectorielles et raster.

Avec **les données discrètes**, il existe un point de départ et un point de fin précis. Il est facile de définir les limites de l'objet - un lac, par exemple, est un objet discret puisque ses limites peuvent être parfaitement établies. D'autres exemples d'objets discrets comprennent les unités administratives (i.e. les frontières définissant une région ou un pays) ou les limites entre les différentes classes d'utilisation des terres. Les cellules appartenant à chaque objet discret se voient attribuer les mêmes valeurs et sont stockées comme Entiers. Les rasters **Entiers** ne peuvent pas présenter de décimales. Les données discrètes sont parfois appelés données catégoriques.

Avec les données continues, les valeurs changent doucement le long du paysage ou de la surface et il n'y a pas de coupure comme pour les données discrètes. Les cellules ont des valeurs individuelles, avec les cellules juxtaposées présentant souvent des valeurs différentes les unes des autres, mais qui présentent toutefois un lien. Les exemples de données continues comprennent l'altitude, la pente et l'aspect ou des données telles que l'indice de la végétation par différence normalisée (Normalized Difference Vegetation Index - NDVI) à partir d'une image de télédétection. Les valeurs numériques de la cellule peuvent être soit stockées comme **entiers** soit comme raster à **virgule flottante**. Les rasters à **virgule-flottante** sont nécessaires pour le stockage des données **continues** qui ont des valeurs d'attribut contenant des décimales.

Tableau 1 : Résumé de certaines différences entre les données vectorielles et raster

Données vectorielles	Données raster
Points, lignes, polygones	Surface avec des **cellules ou des pixels** de taille régulière
Stockage de données discrètes, par exemple, des données avec des limites discrètes telles que les limites d'un pays, des routes, des rues ou des localisations de places.	Cellules raster stockant soit des données **discrètes** (données d'utilisation classée des terres) ou des données **continues** (altitude ou image de télédétection). Les données raster sont spécialement adaptées pour les données continues.
Des relations spatiales complexes peuvent exister au sein et entre les couches vectorielles. Les règles de topologie peuvent être paramétrées (i.e. les relations entre les entités adjacentes ou environnantes) afin qu'elles ne se superposent pas.	Les relations spatiales sont basées sur la localisation, la taille et l'alignement des cellules/ pixels uniquement.
Les données vectorielles ont des tables d'attributs. Pour chaque entité (dans des jeux de données uniques) ou groupe d'entités (dans des jeux de données multiples), des champs peuvent être ajoutés pour stocker des informations supplémentaires	Pour les rasters entiers, les valeurs de la cellule peuvent être stockées dans des tableaux récapitulatifs appelés tableaux de valeur d'attribut (VAT - Value Attribute Tables). Des informations supplémentaires peuvent être stockées dans des champs pour les

concernant le(s) polygone(s), point(s) et la (les) ligne(s).	groupes de cellules de même valeur. Aucun attribut ne peut être ajouté aux cellules/ pixels individuel(le)s. ArcGIS peut créer et voir des VAT, à l'inverse de QGIS. Les rasters à virgule flottante n'ont jamais de table d'attributs.
Les requêtes, les sélections et les superpositions sont possibles. La superposition de trop nombreux jeux de données peut entraîner l'apparition de petits éclats de polygones où les limites ne correspondent pas les unes aux autres. Ceci a tendance à ralentir les analyses ou, lorsque le problème est important, il peut entraîner l'échec des analyses notamment quand les données combinées deviennent trop lourdes pour le traitement.	Comme pour les requêtes, les sélections et les analyses de superposition, il existe de nombreuses autres analyses qui sont plus adaptées ou uniquement réalisables avec des données raster. Les données, afin de réaliser l'analyse, sont transformées vers une résolution de cellules homogènes permettant une analyse rapide et efficace de multiples jeux de données.

A.1.2. Analyse vectorielle vs raster

De nombreuses autres analyses sont plus adaptées ou uniquement réalisables avec des données raster. Vous pouvez créer, faire des requêtes, cartographier et analyser des données avec les deux types de données, raster et vectorielles. Toutefois, l'analyse vectorielle vous permet uniquement de réaliser quelques opérations de superposition simples alors que des options d'analyse plus complexes, multicritères, sont disponibles avec les rasters. Les fonctions d'analyse raster vous permettent également de faire des analyses de raster et vecteur entier, de dériver de nouvelles informations à partir de données existantes et d'exécuter des requêtes multicritères en utilisant plusieurs couches de données.

Le tableau 2 offre un bref aperçu de certaines des fonctions vectorielles et raster les plus courantes. Ceci permettra d'identifier et de localiser les fonctions raster qui sont équivalentes/ similaires aux fonctions vectorielles. Il peut également exister d'autres fonctions, non listées, qui réalisent des tâches similaires. **Il est important de lire l'aide pour chaque outil puisque la table donne seulement une indication générale de la fonction des différents outils listés. Les outils peuvent fonctionner de façon légèrement différente**. Les descriptions et noms d'outils sont principalement basés sur les informations des boîtes à outils de traitements d'ArcGIS et QGIS. Dans AcrGIS, depuis le menu principal cliquez sur **Aide>>Aide de bureau ArcGIS** ou accédez à l'information « Aide » des outils dans la boîte à outil d'ArcGIS. Dans QGIS, référez-vous au guide d'utilisation accessible depuis le menu principal **Aide>> Table des matières de l'Aide** ou en cliquant sur le bouton **Aide** dans la fenêtre de dialogue des outils. Notez que l'aide QGIS dans les outils n'est pas toujours présente et que vous pourrez voir un message « Sorry, no help is available for this algorithm » (Désolé, l'aide n'est pas disponible pour cet algorithme).

Dans QGIS, davantage d'informations peuvent être obtenues en regardant la page d'aide sur le net :

GRASS https://grass.osgeo.org/documentation/manuals/
SAGA http://www.saga-gis.org/en/index.html
GDAL http://www.gdal.org/gdal_utilities.html

Tableau 2 : Aperçu de certaines des fonctions vectorielles et raster les plus courantes

**Outils d'extraction**	**Exemple d'outils vectoriels**		**Exemple d'outils raster**	
	**ArcGIS**	**QGIS**	**ArcGIS**	**QGIS**
**Vecteur :** Extrait les entités entrantes qui sont à l'intérieur des limites des entités de découpage. Les entités qui sont partiellement dans la couche des/ de l'entité(s) de découpage sont coupées le long de la limite de la couche des/ de l'entité(s) de découpage.<br>**Raster :** Extrait les cellules d'un raster qui correspondent aux zones définies par un masque, polygone, au sein d'un cercle ou d'une boîte de délimitation (étendue)	**Découpe (Clip)**	**Découpe (QGIS)**<br>**Découpe des vecteurs selon une étendue (OGR)**<br>**Découpe des vecteurs selon un polygone (OGR)**<br>**Clip points with polygons (SAGA)**<br>**Cut vector layer (SAGA)**<br>**v.overlay (GRASS)**<br>**v.select (GRASS)**	**Extraire par masque**<br>**Extraire par polygone**<br>**Extraire par rectangle**<br>**Extraire par cercle**	**Découpe un raster selon une étendue (GDAL)**<br>**Découpe un raster selon une couche de masque (GDAL)**<br>**Clip raster with polygon (SAGA)**
**Vecteur :** Extrait les entités/ tableaux enregistrés en utilisant des expressions. ArcGIS utilise le langage SQL (Langage de requête structuré- structured Query Language). QGIS utilise ses propres expressions qui sont précisées dans le guide d'utilisation https://docs.qgis.org/2.14/en/docs/user_manual/working_with_vector/expression.html<br>**Raster :** Extrait les cellules d'un raster	**Sélectionner (Select)**<br>**Sélectionner un tableau (table select)**	**Sélection par attribut (QGIS)**<br>**Sélection par somme d'attributs (QGIS)**<br>**Sélection à l'aide d'une expression (QGIS)**<br>**v. extract (GRASS)**	**Extraire par attribut (Extract by attribute)**	**Add raster values to points (SAGA) - ajouter des valeurs raster aux points**<br>**Add raster values to features (SAGA) - ajouter des valeurs raster aux entités**<br>**Raster values to points (SAGA)**<br>**Raster values to features (SAGA)**

DRAFT

sur la base d'une requête logique. ici encore ArcGIS et QGiS possèdent une syntaxe différente. Dans QGIS, la syntaxe peut changer selon qu'on utilise QGIS, GDAL, SAGA ou GRASS.				
**Vecteur :** Décompose les entités entrantes pour créer un sous ensemble de couches multiples sortantes depuis une couche décomposée.<br>**Raster :** Crée un sortant quadrillé à partir d'un jeu de données entrant raster.	**Décomposer (Split)**	**Séparer une couche vecteur (QGIS)**	**Outil de décomposition raster**	**Gdal2tiles (GDAL)**
**Vecteur :** Sélectionne les entités dans une couche sur la base de leurs relations avec les entités d'une autre couche.	**Sélectionner la couche en fonction de la localisation**	**Extraire par localisation (QGIS)**	**N/A**	**N/A**
**Outils de proximité**	**Exemple d'outils vectoriels**		**Exemple d'outils raster**	
	**ArcGIS**	**QGIS**	**ArcGIS**	**QGIS**
Voir le résumé ici :- http://resources.arcgis.com/EN/HELP/MAIN/10.1/index.html#//018p00000007000000<br><br>**Vecteur :** Outils de distance en ligne droite pour créer des zones tampon à une distance spécifique.<br>**Raster :** Différents outils de distance	**Zone tampon (Buffer)**<br>**Cercle multiple**<br>**Zone tampon (Buffer)**	**Distance tampon fixe (QGIS)**<br>**Fixed distance buffer (SAGA) - distance tampon fixe**<br>**Distance tampon variable (QGIS)**<br>**Variable distance buffer (SAGA) - Distance tampon variable**<br>**v.buffer.distance (GRASS)**	**Distance euclidienne (Euclidean distance)**<br>**Coût de déplacement (Cost distance)**	**Proximité (raster distance) (GDAL)**<br>**r.grow.distance (GRASS)**<br>**r.grow (GRASS)**<br>**r.cost (GRASS)**<br>**r.cost.full (GRASS)**<br>**r.cost.full.raster (GRASS)**<br>**r.spreadpath (GRASS)**

Outils de proximité	Exemple d'outils vectoriels		Exemple d'outils raster	
qui réalisent des distances plus élaborées que la seule ligne droite, prenant par exemple en compte l'altitude, etc.		**v.distance (GRASS)**<br>**v.distance.toattr (GRASS)**	**Distance la plus courte (Path distance)**	**Least cost paths (SAGA)**<br>**Accumulated cost (anisotrophic) (SAGA)**<br>**Accumulated cost (isotrophic) (SAGA)**
**Vecteur :** Chaque polygone de Thiessen contient uniquement une seule entité de type point en entrée. N'importe quelle localisation à l'intérieur d'un polygone de Thiessen est plus proche du point associé à ce polygone que de toute autre entité ponctuelle entrante.<br>**Raster :** divise une zone et attribue chaque cellule à l'entité entrante la plus proche qui a une valeur.	**Créer des polygones Thiessen**	**Polygones de Voronoi (QGIS)**<br>**v.voronoi (GRASS)**	**Attribution euclidienne**	**r.grow.distance (GRASS)**

Outils statistiques	Exemple d'outils vectoriels		Exemple d'outils raster	
	ArcGIS	QGIS	ArcGIS	QGIS
**Vecteur :** Crée un tableau avec des statistiques basées sur la contiguïté du polygone (superpositions, bords coïncidents ou points).<br>**Raster :** Calcule, pour chaque localisation de cellule entrante, une statistique des valeurs dans un espace voisinant spécifié.	**Polygones voisins**	**NNjoin (extension QGIS)**	**Boîte à outils Voisinage : Outil de statistiques focales**	**Statistiques de la couche raster (QGIS)**

Outils statistiques	Exemple d'outils vectoriels		Exemple d'outils raster	
Lit une table et un jeu de champs et crée une nouvelle table contenant des valeurs de champ unique et le nombre d'occurrences de chaque valeur de champ unique.	**Fréquence**	**Frequency Analysis (QGIS)**	Pour les rasters entiers, la table de valeur attributaire est similaire à une table de fréquence dans le sens qu'elle contient des valeurs de champ uniques et le compte du nombre de cellules.	**Unique values count (SCRIPT)**<br>**Extract raster values to CSV (SCRIPT)**
Calcule des statistiques de synthèse pour le(s) champ(s) d'une table	**Résumés Statistiques**	**Statistiques de base pour les champs numériques (QGIS)**<br>**Statistiques de base pour les champs de texte (QGIS)**<br>**Statistiques par catégories (QGIS)**	N/A	N/A
Calcule de statistiques sur les valeurs d'un raster dans les zones d'un autre jeu de données.<br><br>Synthétise les valeurs d'un raster dans les zones d'un autre jeu de données et reporte les résultats dans une table.	N/A	**N/A**	**Outil de statistiques zonales, statistiques zonales comme outil table**	**r.stats (GRASS)**<br>**r.univar (GRASS)**<br>**r.statistics (GRASS)**<br>**Zonal raster statistics (SAGA)**<br>**Statistiques zonales (QGIS)**<br>**Raster statistics for polygons (SAGA)**
Calcule des aires recoupées entre deux jeux de données et crée une table.	N/A	N/A	**Croiser les superficies**	**Cross-Classification and Tabulation (SAGA)**
Calcule une statistique par cellule depuis des raster multiples.	N/A	N/A	**Statistiques de**	**Statistics for rasters (SAGA)**<br>**r.report (GRASS)**

Outils statistiques	Exemple d'outils vectoriels		Exemple d'outils raster	
Les statistiques disponibles sont Majorité, Maximum, Moyenne, Médiane, Minimum, Minorité, Plage, Écart type, Somme, Variété			**cellule**	

Outils superposition	Exemple d'outils vectoriels		Exemple d'outils raster	
	**ArcGIS**	**QGIS**	**ArcGIS**	**QGIS**
**Vecteur : Outil Union** - Toutes les entités et leurs attributs seront écrits dans la couche de sortie. **Outil identité** - les entités ou portions entrantes qui chevauchent les entités d'identité auront les attributs de ces entités d'identité. **Outil d'intersection** - Les entités et les portions d'entités qui se superposent dans toutes les couches et/ ou les classes d'entité seront inscrites dans la couche de sortie.<br>**Raster :** Combine les rasters multiples pour qu'une valeur de sortie unique soit attribuée à chaque combinaison unique de valeurs entrantes.<br>Note : Voir aussi les outils d'analyse multicritères qui sont listés séparément dans cette table sous les outils spécifiques rasters.	**Union**<br>**Identité**<br>**Intersection**	**Union (QGIS)**<br>**Polygon identity (SAGA)**<br>**Intersect (SAGA)**<br>**Intersection (QGIS)**<br>**v.overlay (GRASS)**<br>**v.patch (GRASS)**	**Combinaison** (Note : toutes les cellules dans les rasters d'entrée qui ont une valeur no data seront exclues du raster sortant même si ces cellules contiennent des données dans certains des rasters d'entrée).	**r.cross (GRASS)**<br>**Cross-classification and tabulation (SAGA)**

Outils superposition	Exemple d'outils vectoriels		Exemple d'outils raster	
**Vecteur :** Calcule une intersection géométrique des Entités Entrantes et des Entités Mises à jour. Les attributs et la géométrie sont mis à jour par les entités mises à jour dans la couche sortante.<br>**Raster :** Crée une mosaïque, à partir de multiples jeux de données rasters, dans un nouveau jeu de données raster.	**Mise à jour**	**Mise en polygone (QGIS)**	**Mosaïque vers un nouveau raster**	**Mosaic raster layers (SAGA)**<br>**r.patch (GRASS)**

Outils raster spécifiques	Exemple d'outils raster	
	**ArcGIS**	**QGIS**
**Outils d'analyses multicritères :**<br>Les outils d'analyse de superposition vous permettent d'appliquer une pondération à plusieurs entrants et de les combiner en un seul sortant. L'application la plus commune est destinée à la modélisation d'adaptabilité.	**Appartenance floue**<br>Transforme le raster entrant en une échelle de 0 à 1, indiquant l'importance de l'appartenance à un jeu, en se basant sur un algorithme de traitement flou (fuzzification) spécifique.<br>**Superposition floue**<br>Combine des données raster d'appartenance floue ensemble en se basant sur un type de superposition sélectionné.<br>**Superposition pondérée**<br>Superpose plusieurs rasters utilisant une échelle de mesure commune et pondère chacun d'eux selon leur importance.<br>**Somme pondérée**<br>Superpose plusieurs rasters, multipliant chacun d'entre eux par leur propre pondération et en les additionnant ensemble.<br>Pour plus de détails, vois les pages d'Aide : http://help.arcgis.com/en/arcgisdesktop/10.0/help/index.html#/An_overview_of_the_Overlay_tools/009z000000rm000000/ et cet article sur la logique floue : http://www.esri.com/news/arcuser/0410/fuzzylogic.html	**Fuzzify (SAGA)**<br>Traduit les valeurs de la grille en jeu d'appartenance floue comme préparation pour une analyse logique floue.<br>**Fuzzy union (or) (SAGA)**<br>Calcule l'union (opérateur max) pour chaque cellule des grilles sélectionnées.<br>**Fuzzy Intersection (and) (SAGA)**<br>Calcule l'intersection (opérateur min) pour chaque cellule des grilles sélectionnées.<br>**Ordered weighted averaging (SAGA)**<br>Superpose plusieurs rasters utilisant une échelle de mesure commune et pondère chacun d'eux selon leur importance.<br><br>Voir l'aide pour les outils de calculateur de grille (Grid Calculus) dans SAGA :<br>http://www.saga-gis.org/saga_tool_doc/3.0.0/grid_calculus.html<br>et les outils d'analyse de grille :<br>http://www.saga-gis.org/saga_tool_doc/3.0.0/grid_analysis.html
**Évaluation conditionnelle :**<br>réalise une évaluation conditionnelle « si/ sinon » sur chacune des cellules d'entrée d'un entrant. Par exemple, si une valeur est > x alors elle exécute une certaine opération et si elle ne	**Outils con**<br><br>**(cette fonction peut aussi être réalisée dans la calculatrice raster)**	**Cette fonction peut aussi être réalisée dans la calculatrice raster.**<br>**Voir http://gis.stackexchange.com/questions/69734/what-is-the-equivalent-of-arcpy-con-in-qgis-and-or-r-raster-package**

Outils raster spécifiques	Exemple d'outils raster	
l'est pas, elle en exécute une autre.		
**Outil de réglage Nul (Set Null tool)** renvoie les localisations de cellules identifiées à une valeur No Data en se basant sur un critère spécifique. Il renvoie à une valeur No Data si une évaluation conditionnelle est vraie et renvoie à la valeur spécifiée par un autre raster si elle est fausse.	**Set Null**<br>**(cette fonction peut aussi être réalisée dans la calculatrice raster)**	**Cette fonction peut aussi être réalisée dans la calculatrice raster.**
**Outils Math** qui réalisent des opérations mathématiques sur des rasters.<br><br>Les **Outils Math Logiques (Logical Math)** sont utiles pour les analyses multicritères. Ils évaluent les entrants et déterminent les valeurs sortantes en se basant sur une logique booléenne.	Le **jeu d'outils Math** dans ArcGIS contient des outils qui réalisent des opérations mathématiques sur les rasters. **Le jeu d'outils Math Logique** contient des outils pour réaliser des évaluations logiques sur des rasters qui vérifient que les déclarations sont vraies ou fausses. Les outils sont distribués dans les catégories suivantes : **Booléen, Combinatoire, Relationnel** et **Conditionnel.**<br>➢ Les **outils Booléens** évaluent uniquement la véracité ou l'inexactitude des conditions et renvoient le résultat d'un outil particulier comme valeur booléenne 1 ou 0 (Vrai ou faux).<br>➢ Les **outils Combinatoires** identifient des combinaisons uniques de valeurs entrantes en se basant sur la logique d'un outil particulier et renvoient une valeur différente pour chaque combinaison unique.<br>➢ Les **outils Booléens** comparent les valeurs d'un entrant par rapport à un autre entrant et	Le **jeu d'outils Raster Calculus (SAGA)** dans QGIS contient des outils qui réalisent des opérations mathématiques sur les rasters.<br><br>**Pas d'outil Math Logique dans QGIS. Ces fonctions peuvent aussi être réalisées dans la calculatrice raster.**

Outils raster spécifiques	Exemple d'outils raster	
	renvoient le résultat d'un outil particulier comme valeur Booléenne 1 ou 0.<br><br>**(ces fonctions peuvent aussi être réalisées dans la calculatrice raster)**	
**Calculatrice raster**<br>Permet de réaliser des calculs personnalisés basés sur les valeurs de jeux de données rasters.	**Calculatrice raster**<br>Construit et exécute une unique expression algébrique de carte en utilisant la syntaxe python dans une interface de type calculatrice.	La syntaxe varie selon la calculatrice que vous utilisez :<br>**Calculatrice raster (QGIS)**<br>https://docs.qgis.org/2.2/en/docs/user_manual/working_with_raster/raster_calculator.html<br><br>**r.mapcalculator (GRASS)**<br>https://grass.osgeo.org/grass73/manuals/r.mapcalc.html<br>**Raster calculator (SAGA)**<br>http://www.saga-gis.org/saga_tool_doc/3.0.0/grid_calculus_1.html

A.2. Analyse raster sous QGIS

La **boîte à outils de traitements** offre aux utilisateurs QGIS un accès à un grand nombre d'**outils d'analyse raster** dans QGIS, GDAL, SAGA, GRASS et plus. Quelques outils rasters sont également accessibles depuis **l'onglet raster** du menu principal.

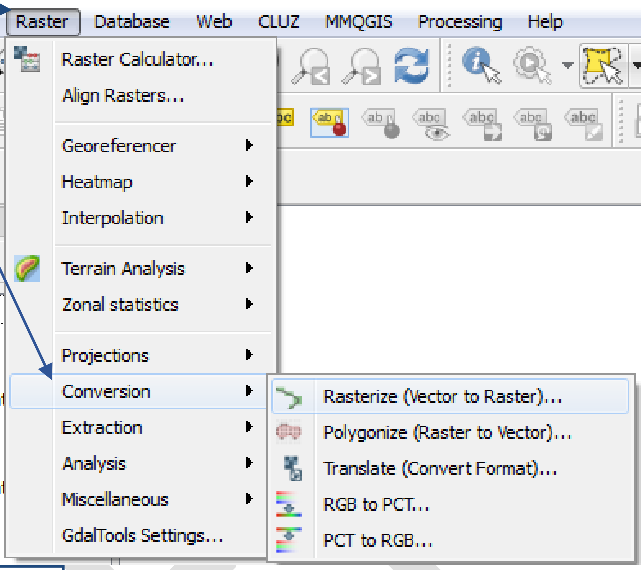

Pour convertir des données vectorielles en raster, depuis le menu principal sur **Raster>> Conversion.**

Note : Dans QGIS il existe différents outils qui sont très proches. Des outils différents peuvent présenter des options différentes. Il est important de lire l'aide et, une fois l'outil exécuté, de bien vérifier les résultats pour vous assurer qu'un sortant approprié a été créé pour votre tâche.

Nombreux outils de conversion raster sont retrouvés dans **la Boîte à Outils de traitements** :

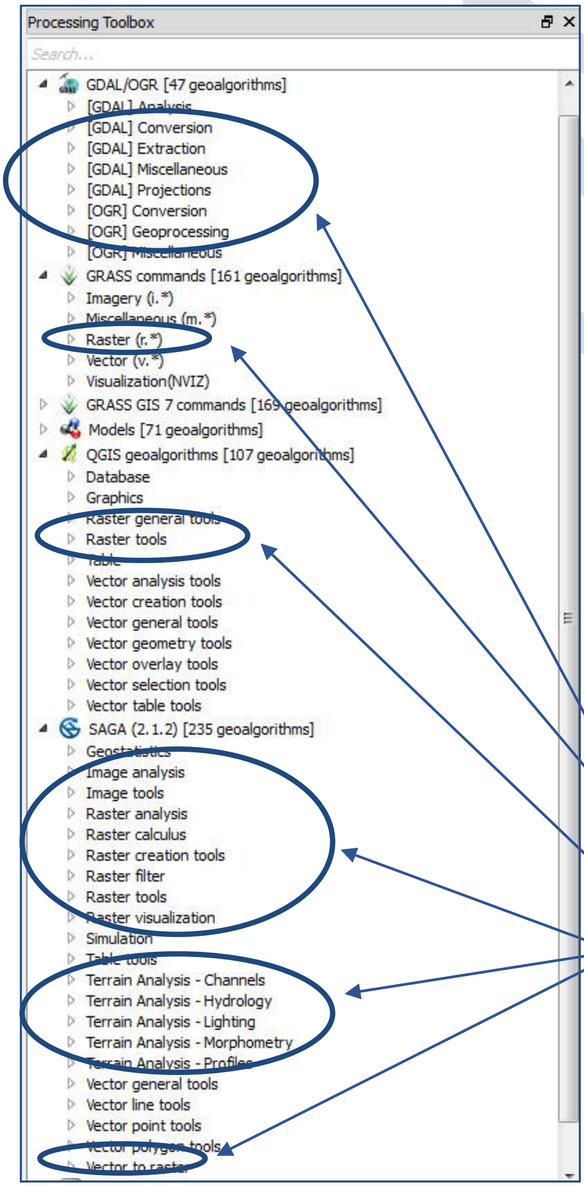

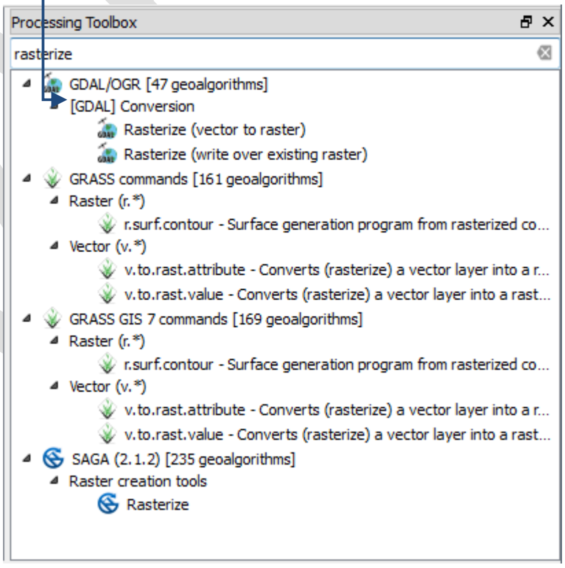

Les outils d'analyse raster sont regroupés dans un sous-ensemble d'outils sous le nom de chaque fournisseur :

GDAL – tous les outils GDAL sont raster

GRASS – la plupart sont sous **Raster (r.*)** bien qu'il existe aussi des outils de conversion vectorielle (v.to.raster...)

QGIS – outils raster listés sous deux sous-groupes raster

SAGA – outils raster listés sous différents sous-groupes raster

Quelques-uns des avantages des analyses raster comprennent la possibilité de :

- Intégrer des analyses vectorielles et raster
- Obtenir de nouvelles informations depuis des données existantes
- Faire des requêtes concernant plusieurs couches
- Réaliser des analyses complexes dans un délai raccourci

A.2.1. Analyse raster et les paramètres d'environnement

Il se peut que vous connaissiez l'ajustement des **Paramètres d'Environnement** pour les géotraitements dans ArcMap. Les paramètres sont des configurations supplémentaires qui peuvent être ajoutées pour modifier les résultats d'un outil. Il y a moins de paramètres cachés dans QGIS mais il y a souvent des paramètres qui sont généralement optionnels dans les outils individuels.

Lors d'une analyse raster, il est très important de s'assurer que les cellules ne sont pas mal alignées (entraînant un glissement des données) dans les couches sortantes.

Dans les outils **GRASS**, il existe des paramètres qui permettent d'éviter de tels glissements.

- **Étendue de la région GRASS (xmin, xmax, ymin, ymax) qui peut être configurée de façon importante tout au long de l'analyse.**
- **Taille de la cellule de la région GRASS**

Et de façon similaire dans les outils **SAGA** :

- **étendue du sortant (xmin, xmax, ymin, ymax)**
- **Taille de la cellule**

Dans le modeleur graphique, il existe également quelques outils réservés aux modèles qui permettent à l'utilisateur de configurer les paramètres des **limites des couches raster** et des **limites des couches vectorielles** qui récupèrent un rectangle de délimitation géographique à partir d'un jeu de données vectorielles ou raster qui sera utilisé comme étendue.

Puisque QGIS accède à des algorithmes à partir de différents fournisseurs, il est encore plus important de vérifier que les étapes de l'analyse produisent un résultat homogène.

A.2.2. Espaces de travail

Lorsque vous réalisez un géotraitement dans QGIS, vous pouvez décider que le sortant produit par l'outil soit permanent ou temporaire. Pour les sortants temporaires, les jeux de données sont stockés dans un fichier de sortie spécifié dans les options de traitement à partir de l'onglet **Traitement** de la

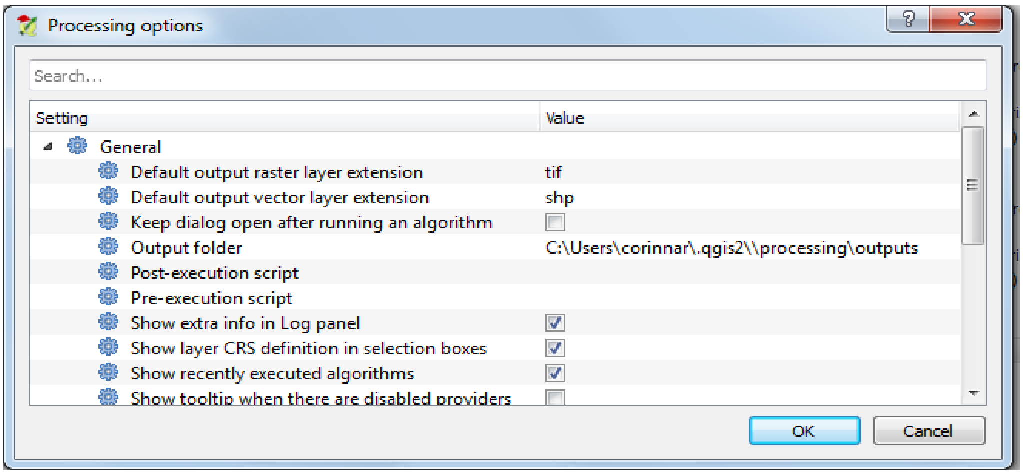

barre de menu principale.

A.2.3. Coordonnées de Sortie

Dans QGIS, il existe plusieurs options pour la configuration du système de coordonnées de sortie. Vous pouvez préconfigurer le Système de Coordonnées de Sortie pour les jeux de données sortants pour l'ensemble du projet.

a. Depuis le menu principal de QGIS, cliquez sur **préférence>>options**
b. Cliquez sur l'onglet **SCR.**

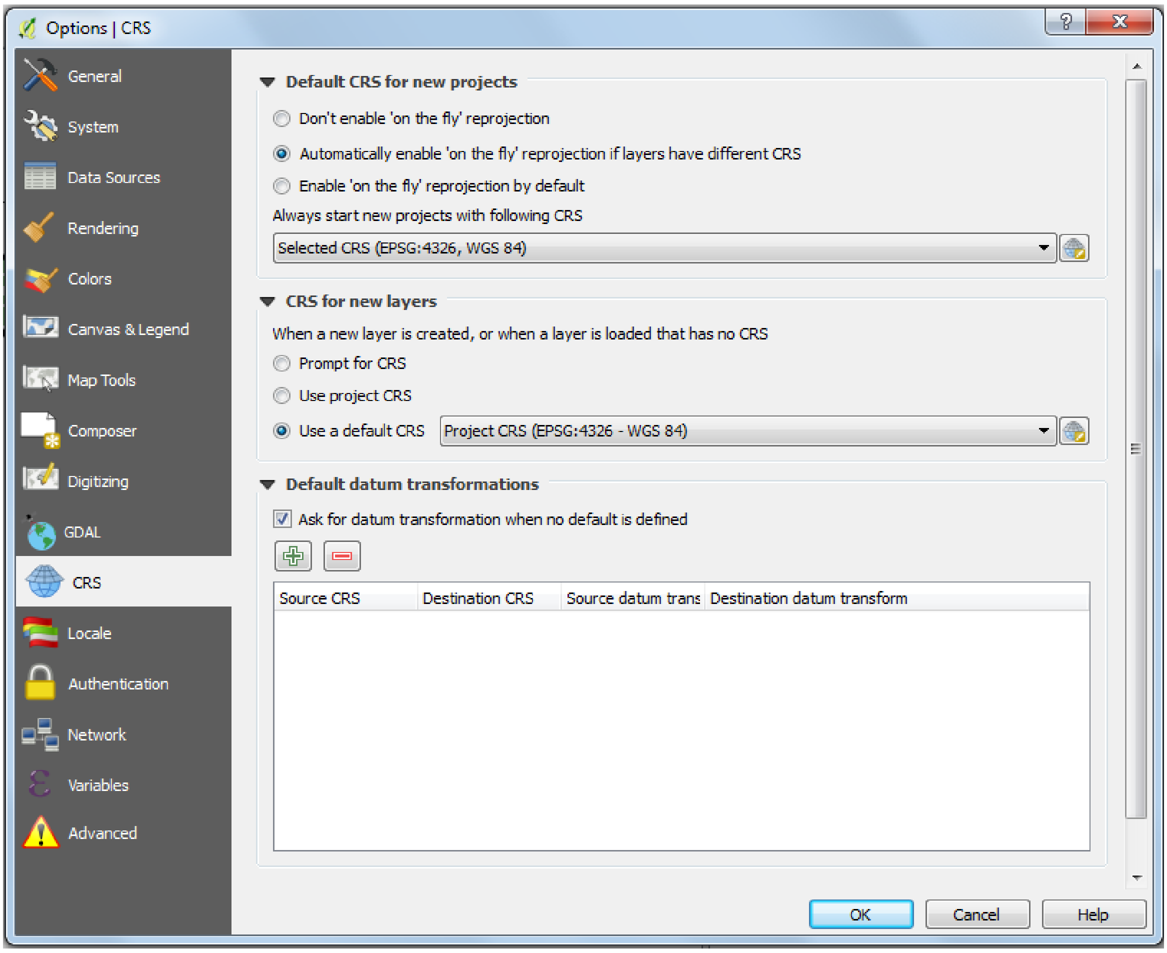

A.2.4. Taille de la cellule

Lorsque vous configurez la taille de la cellule dans l'analyse raster, les outils utiliseront cette taille de cellule pour la taille de cellule des rasters de sortie, ou la résolution, pour l'opération. Par défaut, la résolution du sortant est déterminée par le plus grossier des jeux de données raster entrants. **

*** Il est important de lire l'aide associée à l'outil que vous utilisez pour vous assurer qu'il transforme correctement les données vers une résolution différente. Dans certains cas, il peut être préférable d'utiliser le* ***ré-échantillonnage*** *ou l'****agrégation*** *avant de réaliser une analyse, puisque les cellules sont ré-échantillonnées en utilisant le ré-échantillonnage voisin le plus proche dans les paramètres d'environnement et que ce dernier n'est pas forcément le plus approprié.*

Un jeu de données raster peut toujours être ré-échantillonné pour présenter des cellules plus ou moins grandes, mais ceci n'améliorera pas le niveau de détails du jeu de données. Les facteurs importants dans le choix de la taille des cellules sont :

- La résolution spatiale des jeux de données entrants
- Le type d'analyse
- La taille du jeu de données sortant
- La vitesse d'exécution de l'analyse

Généralement une **petite taille de cellule** doit être utilisée pour des **données à grande échelle** avec **une grande précision spatiale de l'entité** (les petits détails sont retenus), mais dans ce cas, la visualisation de ceci sur une large zone peut entraîner un ralentissement du temps de création. Une **grande taille de cellule** est généralement sélectionnée **pour des données à petite échelle,** puisque **que le temps de création sera ainsi écourté.**

A.3. Une introduction à quelques techniques d'analyse raster dans QGIS

Les conseils suivants offrent d'une part une brève introduction à certaines des fonctions d'analyse raster communes et, d'autre part, des lignes directrices concernant l'utilisation de l'**outil calculatrice raster** pour la construction d'expressions plus complexes. La grande différence entre ArcGIS et QGIS, lorsqu'on travaille avec des rasters, réside dans le fait que QGIS ne présente pas de table attributaire raster associées aux rasters entiers ; ainsi, certaines méthodes de calcul sont différentes.

A.3.1. Standardiser des données raster

Il existe deux méthodes principales : 1) Ré-échantillonner et 2) Agrégation

Méthode 1 : Ré-échantillonner

Vous avez le choix entre deux outils qui peuvent être utilisés et qui permettent à l'utilisateur de changer la taille des cellules et de choisir la méthode de ré-échantillonnage.

a. r.resample.inter (GRASS)

Dans cet outil, la méthode de ré-échantillonnage par défaut est la méthode du **plus proche**. Mais les utilisateurs ont le choix entre trois options :

- Le plus proche
- Bilinéaire
- Bicubique

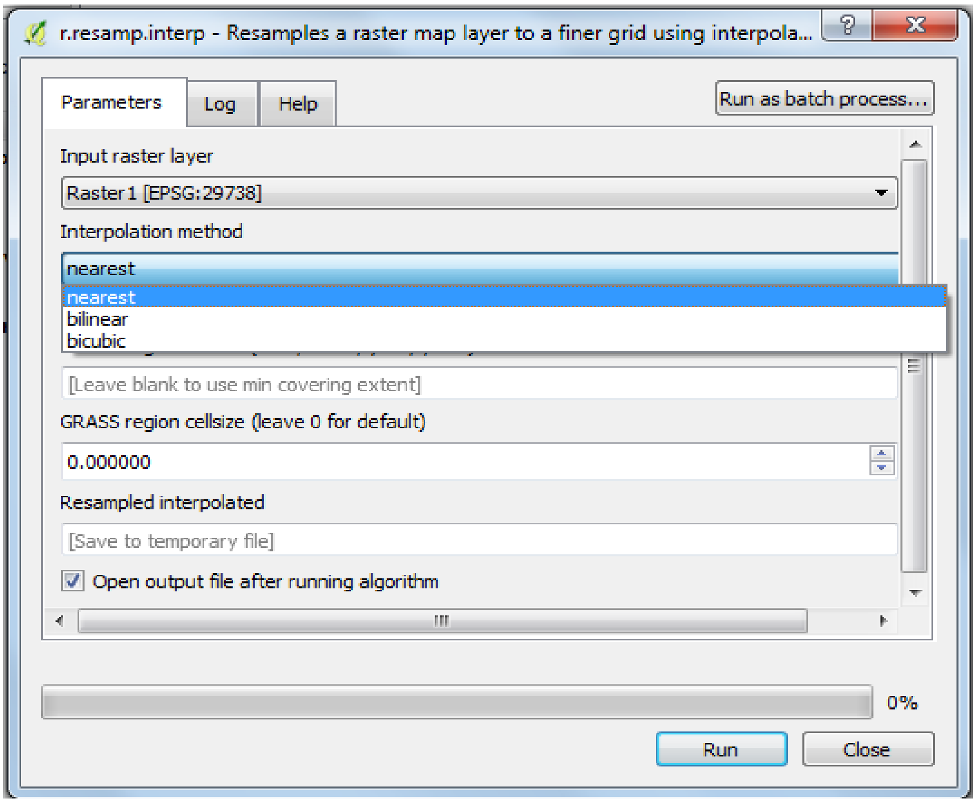

b. Resampling (SAGA)

Dans cet outil, la méthode de ré-échantillonnage par défaut est la méthode du **plus proche voisin**.

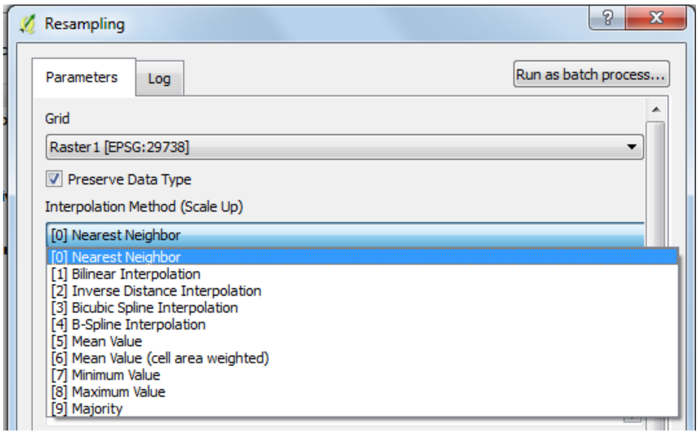

Mais les utilisateurs ont le choix entre dix options pour augmenter l'échelle :

- Voisin le plus proche
- Interpolation bilinéaire
- Interpolation distance inverse
- Interpolation spline bicubique
- Interpolation B-spline
- Valeur moyenne
- Valeur moyenne (zone de cellules pondérées)
- Valeur minimum
- Valeur maximum
- Majorité

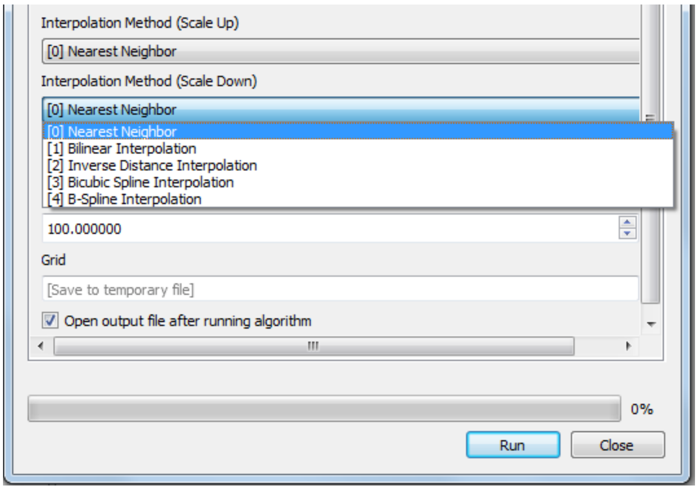

Ou cinq options pour diminuer l'échelle :

- Voisin le plus proche
- Interpolation bilinéaire
- Interpolation distance inverse
- Interpolation spline bicubique
- Interpolation B-spline

Méthode 2 : Agrégation

Les **outils d'Agrégation** permettent à l'utilisateur de choisir un facteur lié à une cellule pour créer une version dont la résolution d'un jeu de données raster sera réduite. Si un utilisateur veut augmenter l'échelle des données à partir d'une taille de cellule de 30 m jusqu'à ~1 km, une **Taille d'Agrégation** de 33 donnera une taille de cellule 33 fois plus grande (i.e. 990 m). Le facteur cellule doit être un nombre entier supérieur à 1.

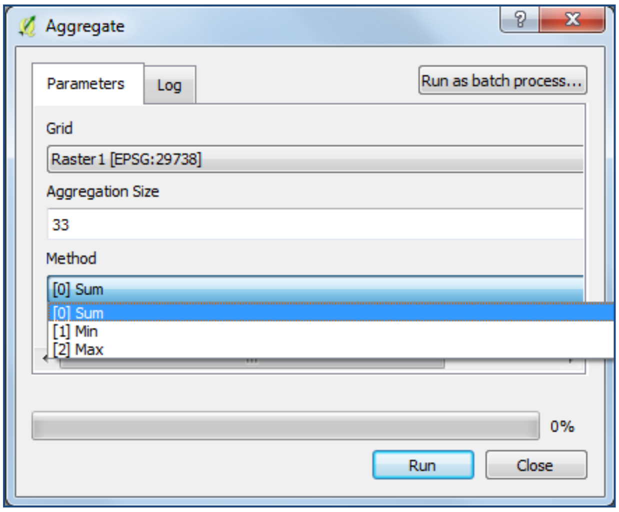

Deux outils au choix peuvent être utilisés :

c. Aggregate (SAGA)

Dans cet outil, la méthode d'agrégation défaut est la méthode somme.

Pour déterminer la méthode de calcul de la valeur de la cellule, l'utilisateur a trois options :

- Sum (Somme)
- Min
- Max

d. r.resample.stats (GRASS)

La méthode d'agrégation par défaut est la méthode **moyenne (average)**.

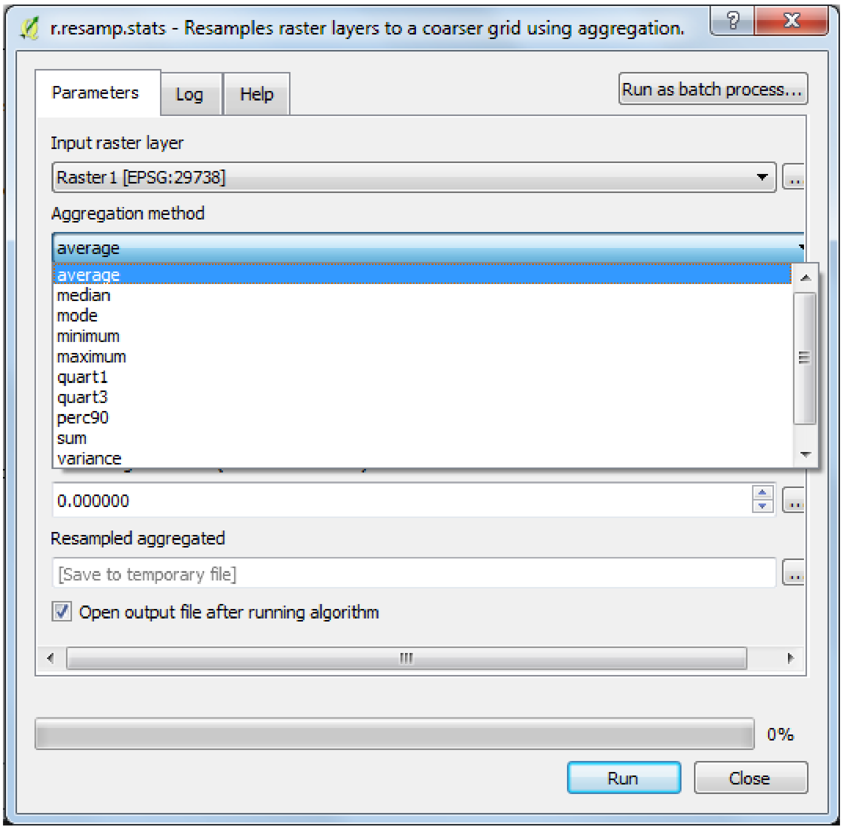

Pour déterminer la méthode de calcul de la valeur de la cellule, l'utilisateur a dix options :

- Moyenne (average)
- Médian
- Mode
- Minimum
- Maximum
- Quart1
- Quart3
- Perc90
- Sum (Somme)
- Variance

L'utilisateur indique une taille de cellule de sortie.

Notez que le ré-échantillonnage et l'agrégation offrent différentes méthodes pour déterminer les valeurs sortantes des cellules. Le ré-échantillonnage permet aux utilisateurs de diminuer ou d'augmenter la taille de la cellule alors que l'agrégation permet uniquement une généralisation vers une taille de cellule plus grande.

A.3.2. Fonctions de reclassification

De nombreux traitements raster utilisent le champ valeur lors du traitement de données. Au cas où vous voudriez regrouper des données ou changer les valeurs dans un jeu de données raster,

de nombreux outils de reclassification sont disponibles :

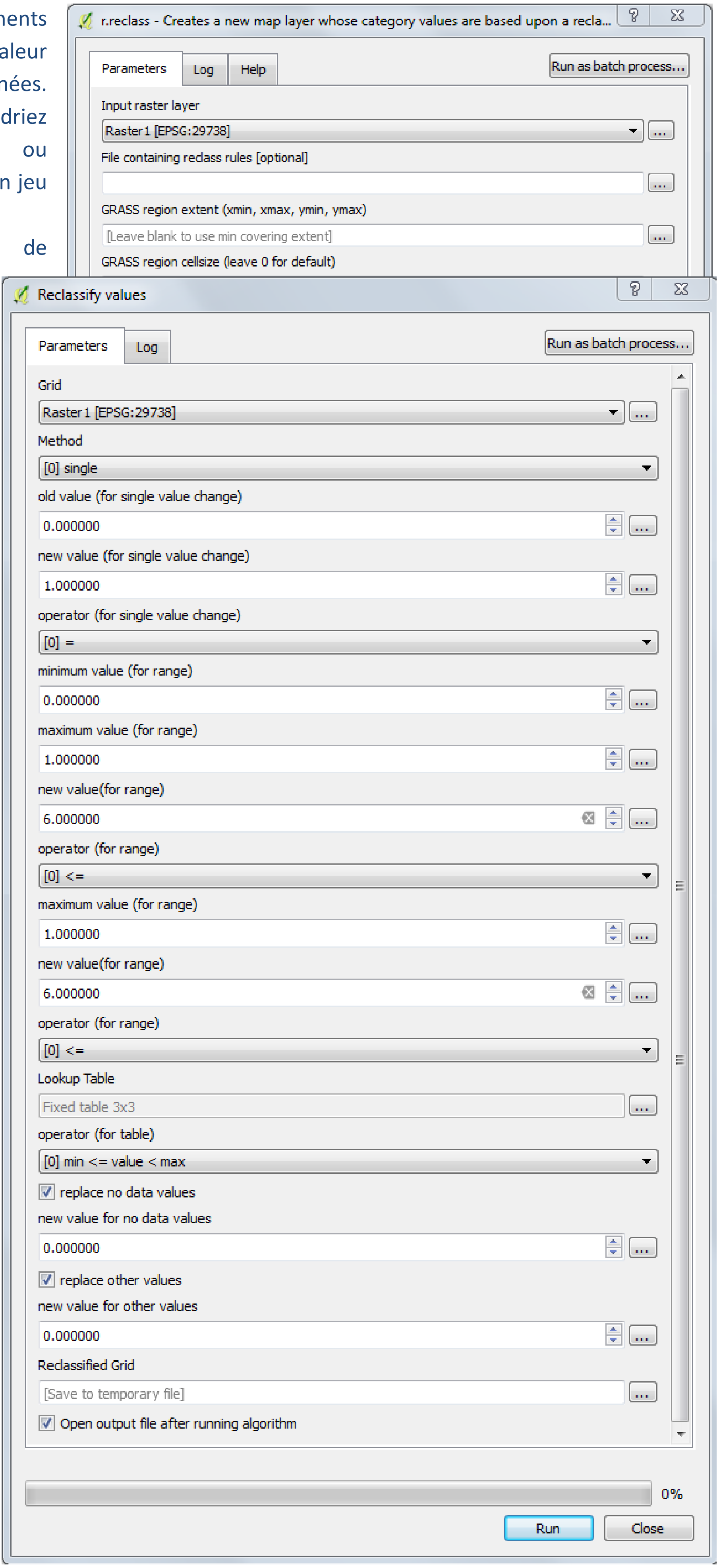

a. r.reclass (GRASS)

Les règles de reclassification sont contenues dans un fichier txt (voir l'aide de l'outil pour plus de détails sur la façon de créer un fichier de reclassification).

b. Reclassify values (SAGA)

Cet outil offre des options variées pour reclassifier depuis une valeur unique, une gamme de valeurs ou en utilisant une table de consultation.

Il fournit également une option pour convertir les valeurs No Data en une autre valeur.

SAGA offre aussi une version simplifiée de cet outil; elle est disponible dans l'**outil Reclassify values (Simple).**

A.3.3. Calculatrice raster : Aperçu

Vous pouvez aussi exécuter un outil utilisant des expressions **d'Algèbre spatial** indiquées dans un **outil Calculatrice Raster.** Les expressions d'algèbre spatiale peuvent être utilisées pour exécuter un outil unique ou des opérateurs, ou pour créer une chaîne d'outils ou d'expressions plus complexe. Dans QGIS, il y a trois différentes calculatrices raster. La calculatrice Raster principale dans QGIS est accessible via le menu principal (mais il est important de noter que celle-ci n'est pas accessible via la boîte à outils de traitements ou le modèle graphique). Les deux autres sont **GRASS r.mapcalculator** et **SAGA Raster Calculator**. Nous allons illustrer la fonction cœur de la calculatrice raster QGIS ici. La syntaxe pour les différentes calculatrice raster varie ; davantage de conseils sont disponibles dans l'aide documentaire de QGIS, GRASS et SAGA.

Dans la calculatrice Raster de QGIS, l'expression est rédigée au bas de la fenêtre. Dans cet exemple, quatre jeux de données raster sont additionnés ensemble.

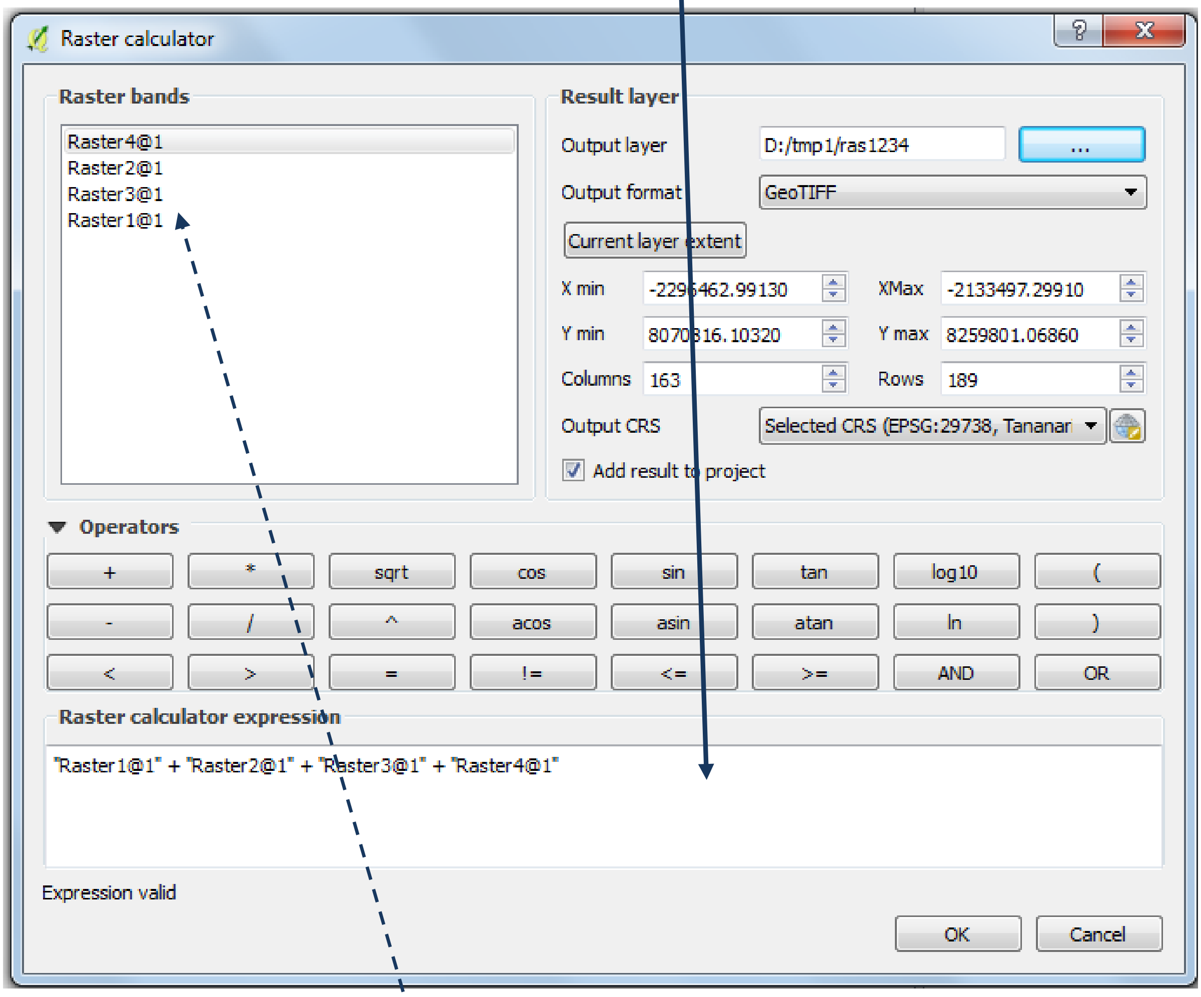

2	5	0
8	6	1
5	1	1

Raster1

1	0	1
0	1	0
0	1	1

Raster2

2	1	1
1	1	1
1	3	1

Raster3

2	1	5
0	0	3
3	3	3

Raster4

=

7	7	7
9	8	5
9	8	6

Sortant Raster

c. La **partie gauche de la fenêtre** montre la **liste des couches** qui peuvent être utilisées dans l'expression. Il s'agit des couches raster que vous avez ajoutées à votre session ArcMap. En

double cliquant sur une couche, vous serez conduit jusque vers une boîte d'expression entre guillemets (et un @1 est placé à la fin du nom).

d. **Le centre de la fenêtre contient la liste des opérateurs. Cliquez une fois sur ceux-ci pour les ajouter à la boîte d'expression.** Il y a des opérateurs simples pour **Ajouter (+), Soustraire (-), Diviser (/) ou multiplier(*).**
Le centre de la fenêtre contient également des opérateurs correspondant à la **logique Booléenne :**

AND et Booléen	!= Inégal à	>= Égale ou supérieur à
OR Ou Booléen	> Supérieur à	<= Égale ou inférieur à
= Égale à	< Inférieur à	

A.3.4. Calculatrice raster : Intersection Booléenne et conversion No Data

L'intersection booléenne est la variante la plus simple de traitement basé sur les critères et est souvent vue comme un outil de cartographie thématique. Avant la combinaison, chaque critère entrant est standardisé à une certaine échelle d'adaptabilité (ou reclassifié en classes de 1 et 0), c.à.d. tous les facteurs sont ainsi réduits à des jeux de données raster Booléens de zones adaptées et inadaptées.

Dans un exemple d'analyse **Et Boolléenne**, si les données pour les forêts contiennent la valeur 1, les données pour les aires protégées contiennent les valeurs 1 et 2 et les zones en dehors des Aires Protégées et de la forêt ont la valeur **No data** ; le raster sortant est 1 lorsque les forêts et les aires protégées coïncident. Les zones restantes sont classifiées en **No Data**. La syntaxe dans la calculatrice raster est :
"nat_for@1" = 1 AND "PA_Raster@1" > 0

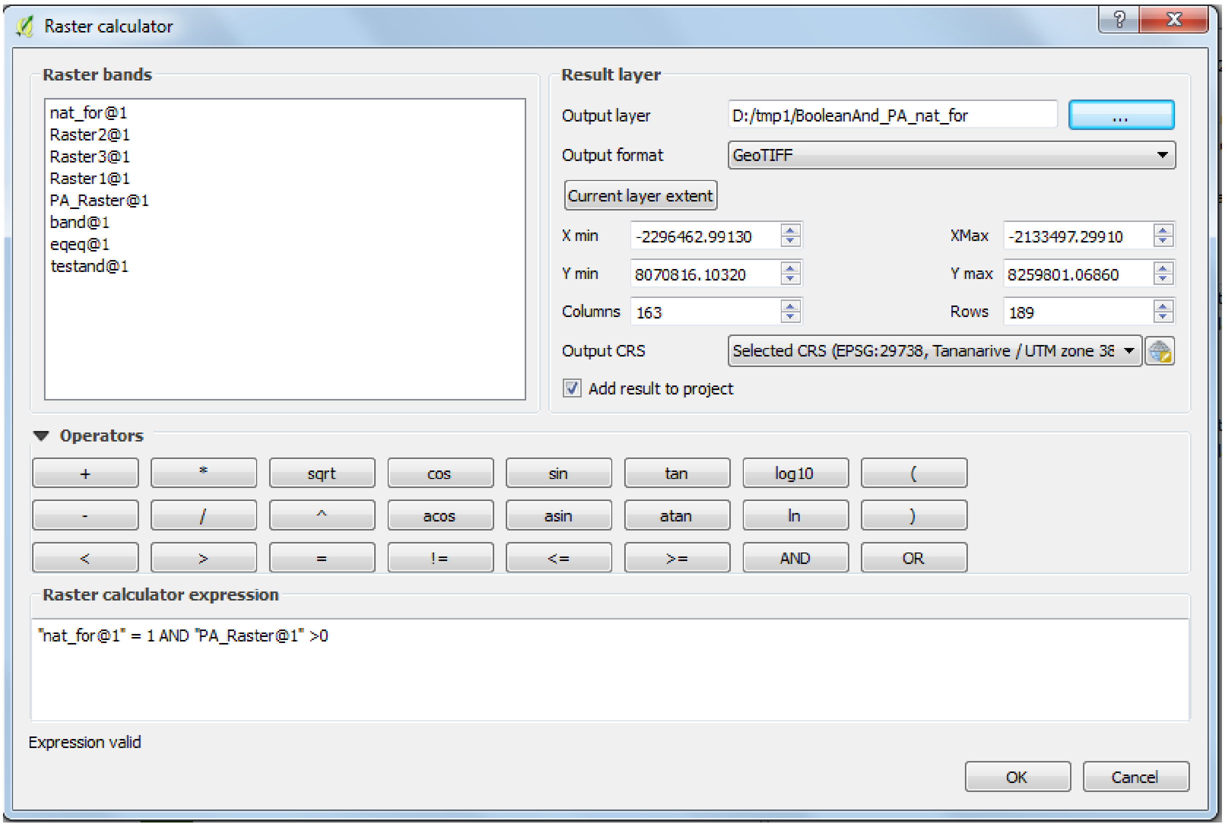

Les jeux de données entrants sont illustrés à gauche et résultent du calcul donné à droite :

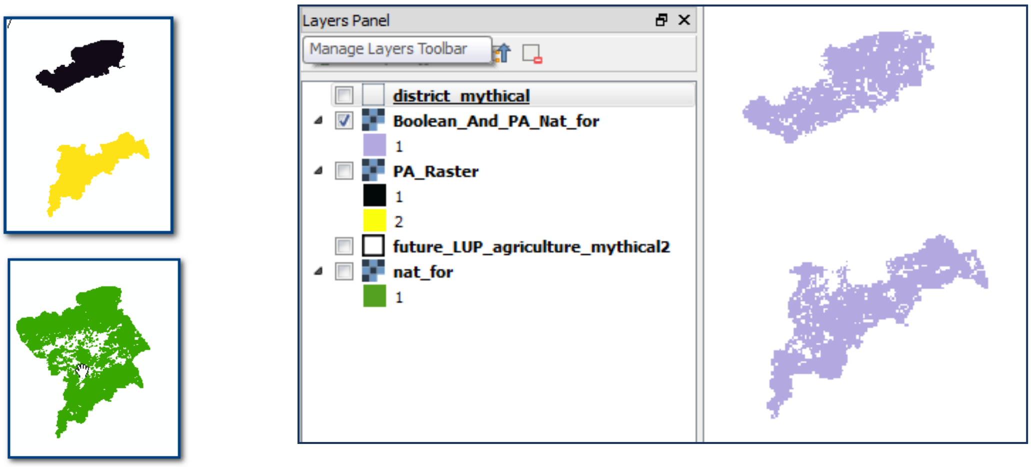

Le No Data n'est pas un problème avec l'outil d'analyse **Et Booléen** puisque l'analyse sélectionne uniquement des valeurs présentes dans les DEUX jeux de données à la fois. Si vous utilisez **LA PLUPART des AUTRES outils dans la boîte à outils logiques,** vous vous apercevrez que **des valeurs No Data peuvent causer des problèmes** et qu'il est nécessaire de les convertir en 0 avant d'utiliser les données (voir section 1.4.1 sur la façon de convertir les valeurs No Data en données).

La fonction **Ou Booléen** sélectionne les endroits où les valeurs sont supérieures à 0 dans l'un des deux jeux de données. Toutefois, si des valeurs No Data sont présentes dans l'un des jeux de données, celles-ci priment sur toute autre valeur et jouent comme une contrainte supplémentaire. Par exemple, si une cellule a une valeur de 1 dans le jeu de données forêt et une valeur No Data dans le jeu de données Aires Protégées, la cellule de sortie aura une valeur No Data. *** Ainsi, avec des **valeurs No Data** présentes **dans les couches entrantes, le sortant du OU Booléen** sera **FAUX** !***

La syntaxe dans la calculatrice raster est : "nat_for@1" = 1 OR "PA_Raster@1" > 0

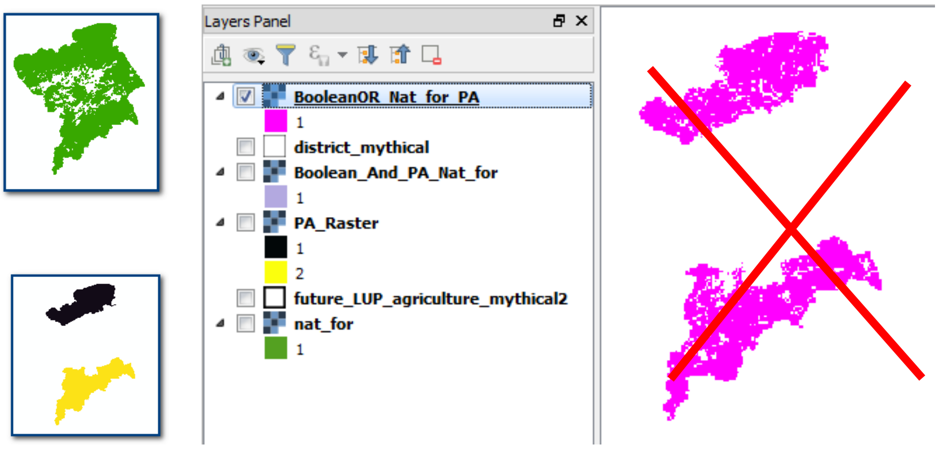

Le résultat devrait afficher des valeurs de 1 là où on retrouve une aire protégée OU une forêt naturelle. Aussi, les valeurs No Data doivent être changées en 0.

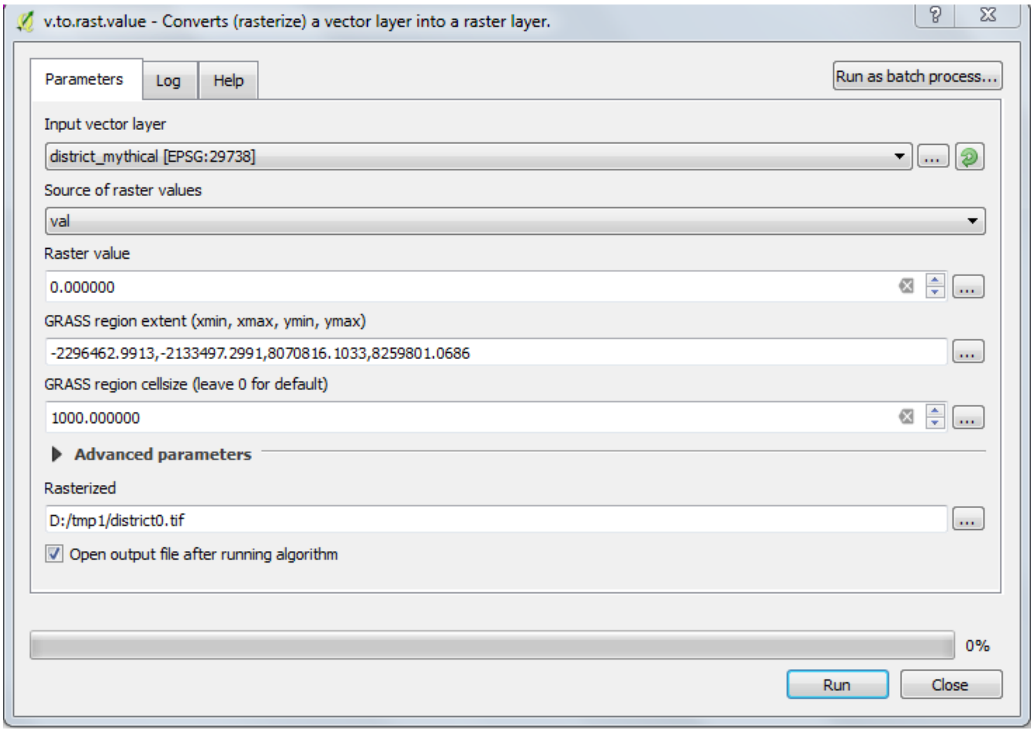

Pour convertir les valeurs No Data du raster Aires Protégées en 0, nous pouvons utiliser le vecteur district afin de créer une nouvelle couche pour le district où toutes les valeurs sont réglées sur 0 en utilisant l'outil **GRASS v.to.rast.value**.

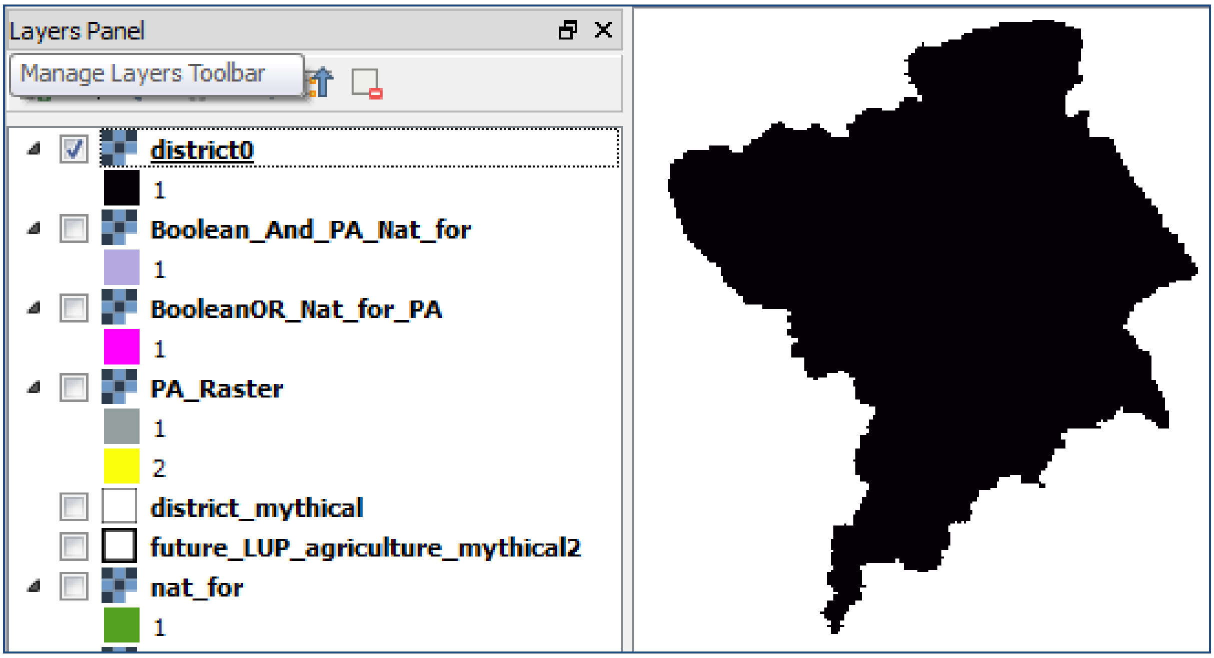

La prochaine étape consiste à fusionner les districts avec les Aires Protégées pour que les zones au sein du district qui ne sont pas protégées aient une valeur 0 au lieu d'être en No Data. Pour réaliser ceci, la couche aires protégées a besoin d'être déplacée au-dessus du raster district dans la table des matières.

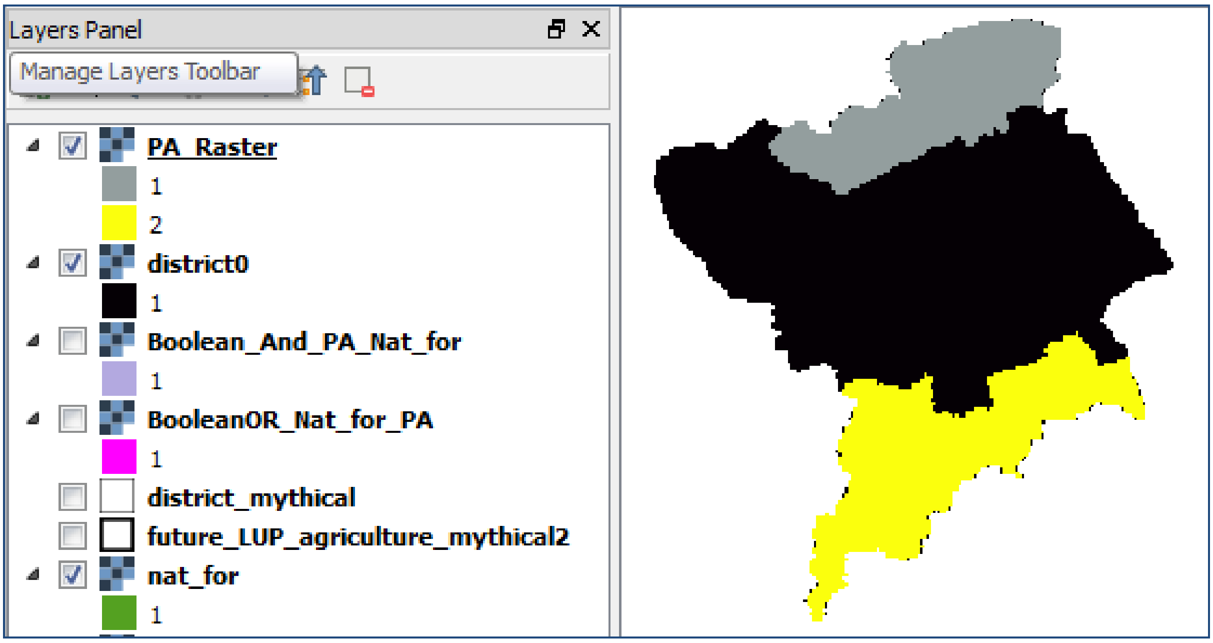

Ensuite, l'outil **r.patch** de GRASS est utilisé pour fusionner cette couche avec la couche aires protégées. L'outil récupérera les valeurs du raster du dessus comme valeurs déterminantes par rapport au raster inférieur.

Les couches raster à rassembler doivent être cochées et l'étendue est réglée sur l'étendue totale de l'aire d'intérêt, c.à.d. dans cet exemple, l'étendue totale du district.

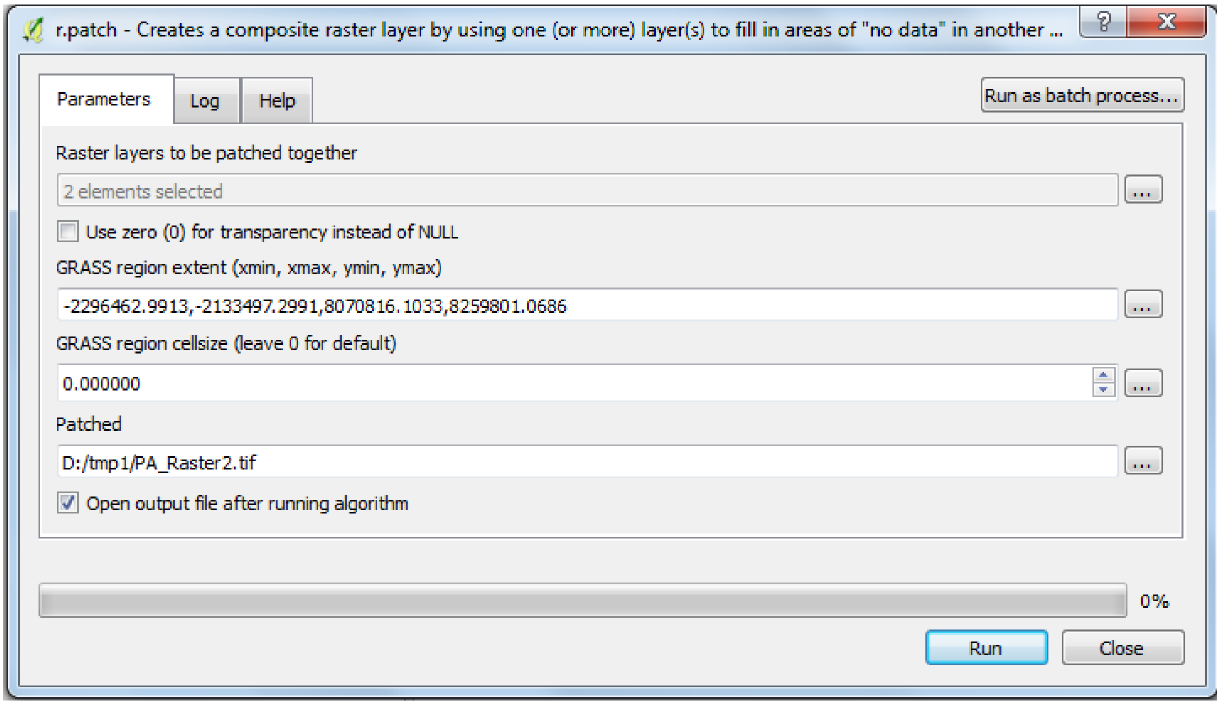

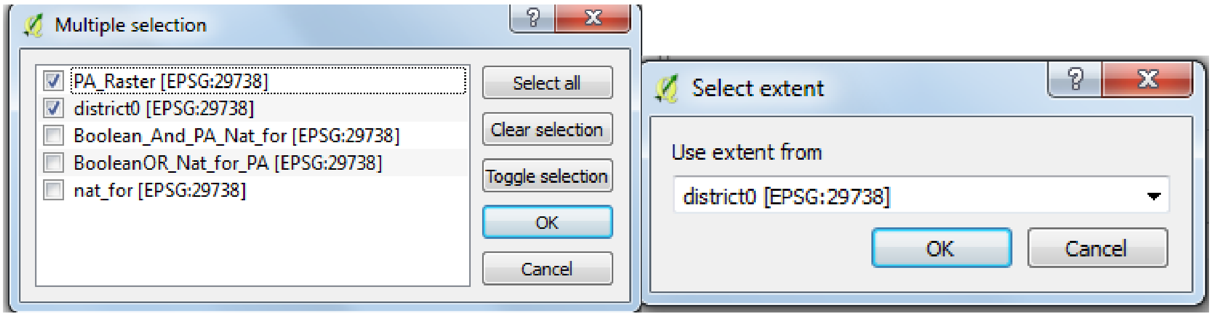

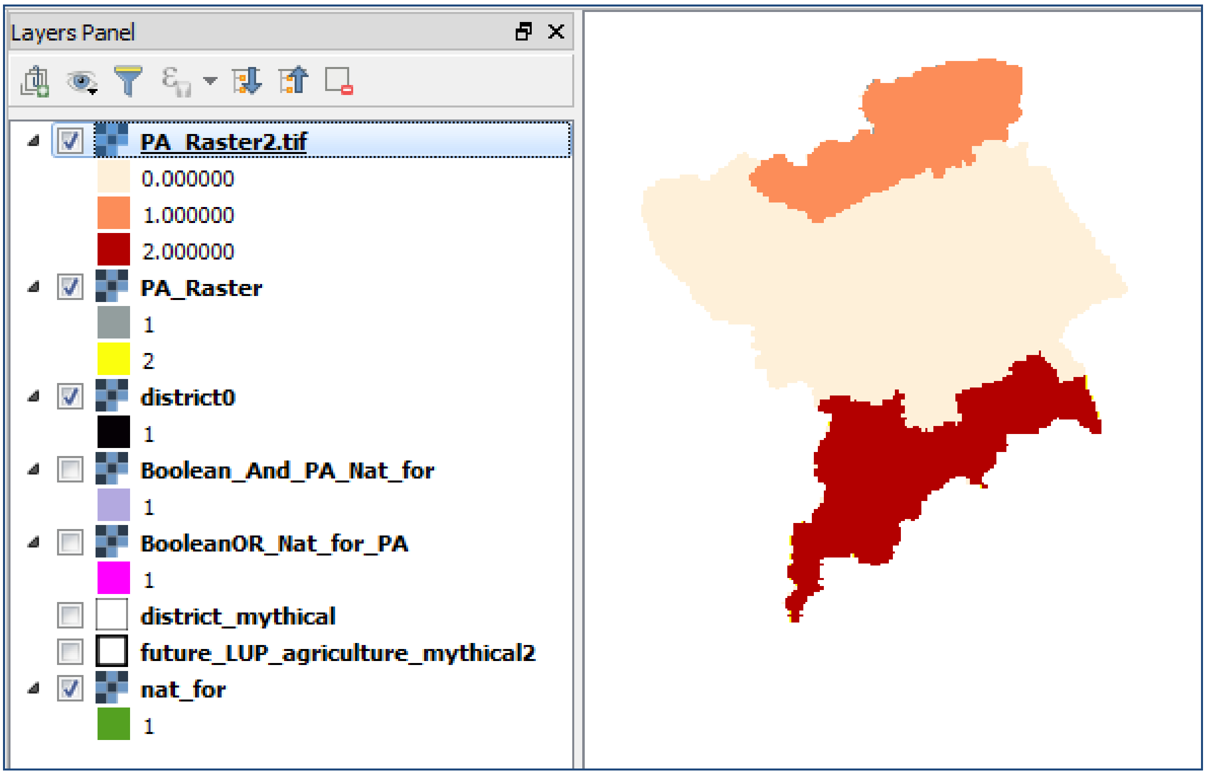

La couche forêt doit également être déplacée au-dessus du raster district dans la table des matières et être fusionnée avec le raster district en utilisant l'outil **r.patch**.

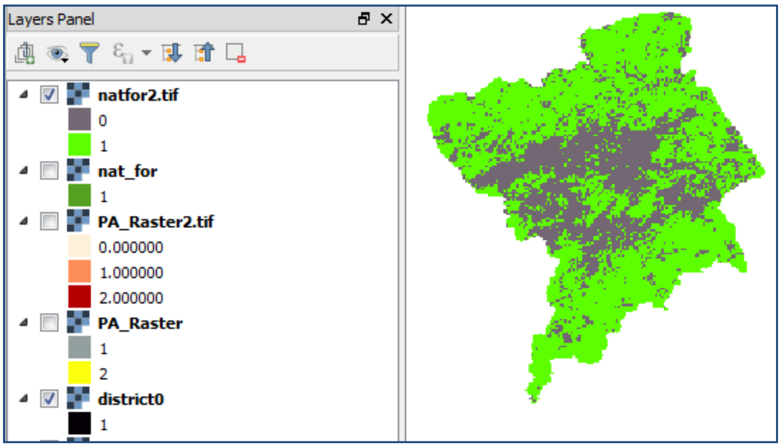

Une fois que les couches couvrent l'ensemble de l'étendue et que leurs valeurs No Data ont été converties en 0, l'expression Booléenne OU peut être entrée dans la calculatrice Raster. La syntaxe dans la calculatrice raster est :

"nat_for2@1" = 1 OR "PA_Raster2@1" > 0

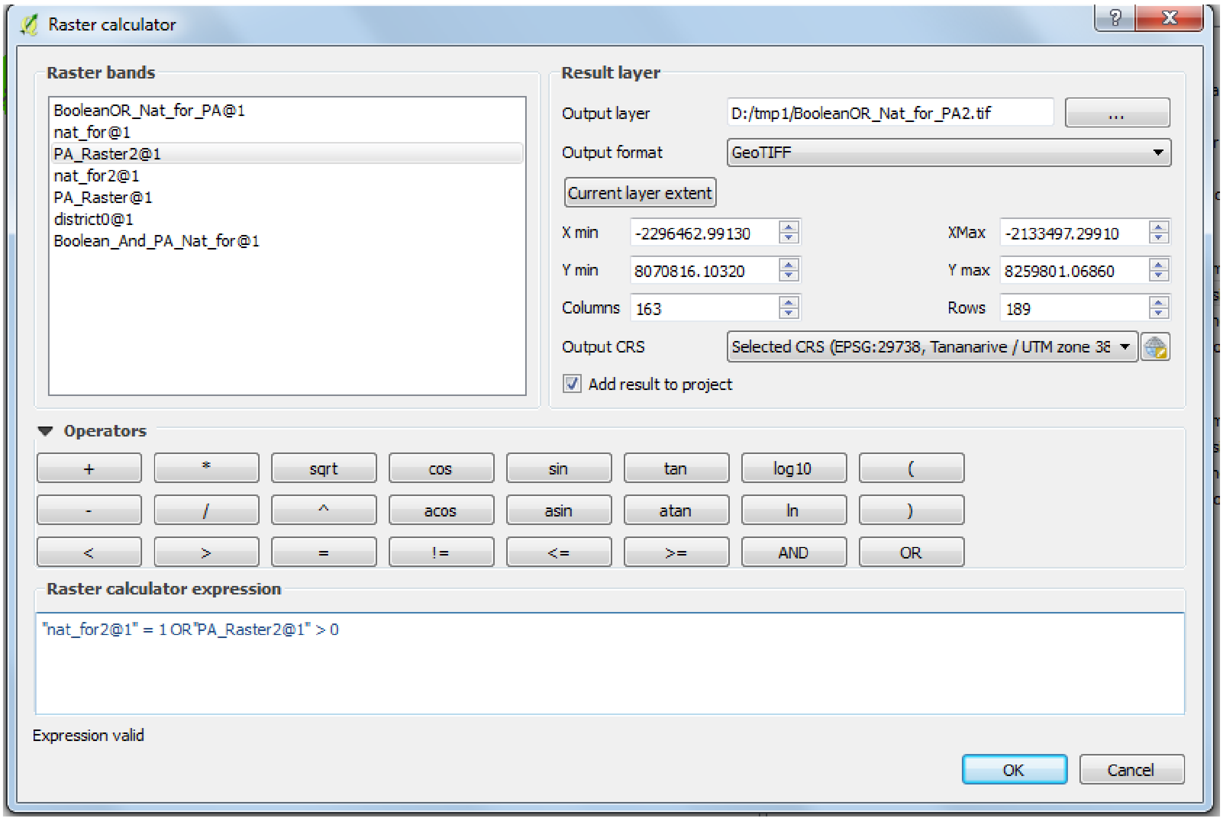

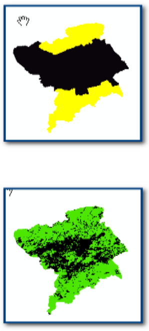

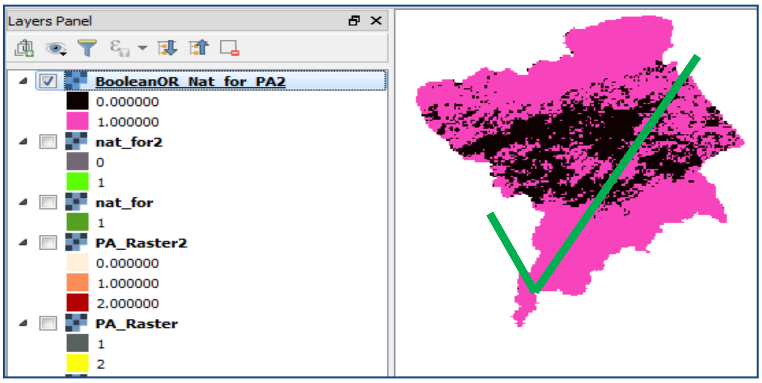

A.3.5. Statistiques Zonales

Les outils de statistiques zonales sont très utiles. Ils peuvent être utilisés pour synthétiser une couche de données raster entrante en une couche zone. La couche **Zone** peut être soit vectorielle soit raster et peut, par exemple, être utilisée pour récapituler le nombre de types de forêts différentes dans des zones protégées individuelles.

Dans ce guide n'étaient présentés en détails que quelques-uns des outils, et ce afin d'aider les utilisateurs à démarrer avec l'analyse raster. Il ne doit pas être considéré comme un guide complet. Les utilisateurs peuvent explorer les boîtes à outils dans QGIS eux-mêmes pour trouver et identifier les outils pertinents qui leur permettront de répondre aux questions qu'ils se posent.

Annexe 2 : Ressources supplémentaires concernant les Modeleurs graphiques QGIS

- Tutoriels
- Exemples en ligne :
  - http://docs.qgis.org/2.0/ca/docs/user_manual/processing/modeler.html
  - http://www.qgistutorials.com/en/docs/processing_graphical_modeler.html
  - http://gis.stackexchange.com/questions/176599/simplify-repetitive-tasks-in-qgis-graphical-modeler
  - http://www.digital-geography.com/creating-models-qgis/#.WEgN7rKLSM8
  - http://gracilis.carleton.ca/CUOSGwiki/index.php/Automating_Vector_and_Raster_Workflows_using_the_Graphical_Modeler_in_QGIS

DRAFT
