# Supplementary material for: Relevance of the uMap Collaborative Platform as Support for Choropleth Mapping of a Traffic-Light Statistical Signal Atlas of All-Cause Mortality During the First French Lockdown: Geospatial Analysis
Source: JMIR Med Inform. 2026 Aug 27;14:e82855. doi: 10.2196/82855 (PMC13543287; doi:10.2196/82855)
Supplement: Supplementary file 8 [file medinform-v14-e82855-s008.zip › BG_question2_geojson_Fig4-Fig5/1_summaryFile_Figs4-5/1_summaryFile_Figs4-5_modeler_qgis.pdf]

```


******************************************************************************************************************** ************
=============================================English========================================================================
*********************************************************************************************************************
======================= Figure 4 and Figure 5==========================

************************* ************************************************************************
===================================================================================================================
Watch videos 1 (Atlas version 1) and 2 (Atlas version 1)

D:\SLIM\__BUREAU_2026\__February_2026\___revision_JMIR_Feb26_c\
BG_response_question2_geojson_Fig4-Fig5\2_video1_modeling_model_creation_processing_Figs4-5

2_core_step4.model3.mp4

D:\SLIM\__BUREAU_2026\__February_2026\___revision_JMIR_Feb26_c\
BG_response_question2_geojson_Fig4-Fig5\3_video2_modeler_python_BatchProcessing

1_run_ttt_histo_and_console_python.mp4



************************* *********************************************************************** *
===================================================================================================================
Use the projects and models provided to reproduce the processes

************************* ****************************************** ****************************
D:\SLIM\__BUREAU_2026\__February_2026\___revision_JMIR_Feb26_c\
BG_response_question2_geojson_Fig4-Fig5\

D:\SLIM\__BUREAU_2026\__February_2026\___revision_JMIR_Feb26_c\
BG_response_question2_geojson_Fig4-Fig5\models
=================================================================================================================== 1_summaryFile_modeler_qgis.txt
The projects are in .qgz format
The models are in .model3 format

NEW_MODELS_DENSI_SMM_KHI2_FEU.qgz
P0_SMM_13Regions_updated_2026-01-22.qgz
‘qgis_processings_22janv2026 - Copy.qgz’
qgis_processings_22janv2026.qgz



```
