# Supplementary material for: Relevance of the uMap Collaborative Platform as Support for Choropleth Mapping of a Traffic-Light Statistical Signal Atlas of All-Cause Mortality During the First French Lockdown: Geospatial Analysis
Source: JMIR Med Inform. 2026 Aug 27;14:e82855. doi: 10.2196/82855 (PMC13543287; doi:10.2196/82855)
Supplement: Supplementary file 13 [file medinform-v14-e82855-s013.zip › MA-SF_FromDensi_iStatisticsToVerbalLabelCategories_12mai2026.docx]

“Multimedia Appendix: [From DENSI_i Statistics to Verbal Label Categories]

In the methodology section of the article, we derived the Khi-square log likelihood ratio (Khi2LnFR) statistics. Mathematically, this statistic represents a log likelihood ratio, which is typically used in spatial epidemiology to compare the observed number of deaths $O_{i}$ with the expected number $E_{i}$ in municipality i, based on the following Poisson test formula (a):

$$\text{Khi2LnFR}_{i}=2\left[ O_{i}\ln\left( \frac{O_{i}}{E_{i}} \right)-(O_{i}-E_{i}) \right]$$

This statistic asymptotically follows a khi-squared distribution with 1 degree of freedom, as confirmed by Wilks’ theorem below [1].

Starting from the defined variable DENSI, for all i (municipalities) and by convention setting $(O/E)_{i}=O_{i}/E_{i}$, we obtain formula (b):

$$\text{DENSI}_{i}=\frac{(O_{i}-E_{i})^{2}}{E_{i}}=((O/E)_{i}-1)^{2}\times E_{i}$$

The resulting formula (b) corresponds to the classical Pearson’s χ² statistic for a Poisson cell.

We set approximation (or “approximate equality”): $e^{\text{Khi2LnFR}_{i}}\approx E_{i}((O/E)_{i}-1)^{2}$, which allows us to isolate the ratio $(O/E)_{i}$, using the following formula (c):

$$(O/E)_{i}\approx1+\sqrt{\frac{e^{\text{Khi2LnFR}_{i}}}{E_{i}}}$$

This mathematical formula (c) thus highlights the dependence of this ratio on the size $E_{i}$; indeed, for a given fixed Khi2LnFR threshold, the implicit $(O/E)_{i}$ ratio decreases as $E_{i}$ increases. For example, if we examine this variation as thresholds are crossed, we obtain the ratio $(O/E)_{i}$ values in Table 1 and Table 2.

Table 1: The ratio $(O/E)_{i}$ values of the “green/orange threshold: “$\text{Khi2LnFR}\text{”}=1.826$, and$e^{1.826}\approx6.21$

| $E_{i}$ | Calculation | $(O/E)_{i}\approx$ |
| --- | --- | --- |
| 5 | $1+\sqrt{6.21/5}=1+1.114$ | 2.11 |
| 10 | $1+\sqrt{6.21/10}=1+0.788$ | 1.79 |

Table 2: The ratio $(O/E)_{i}$ values of the “orange/red threshold: “$\text{Khi2LnFR}\text{”}=3.736$, and$e^{3.736}\approx41.9$

| $E_{i}$ | Calculation | $(O/E)_{i}\approx$ |
| --- | --- | --- |
| 5 | $1+\sqrt{41.9/5}=1+2.895$ | 3.90 |
| 10 | $1+\sqrt{41.9/10}=1+2.047$ | 3.05 |

The verbal labels (used to determine Traffic-Light colors) were ordinal approximations, not fixed thresholds (Figure 1) below. The labels “close to the average to two times above average,” “×2,” “×2–4×,” and “×4–11×” were calibrated for municipalities of typical size (E ≈ 5–20), but the implicit ratio $(O/E)_{i}$ deviates for very small municipalities (discontinuity), creating artifacts rather than actual excess mortality.

Figure 1: Khi2LnFr distribution – 34,833 French municipalities – Traffic-Light classification (Jenks method, 10 classes) – 1^st^ French Lockdown 2020.

# Let us breakdown the calculation of the variable Khi2LnFR

The observed mortality variable ($O_{i}$) follows a Poisson distribution as written in formula (d):

($O_{i}\sim\mathcal{P}\left( E_{i} \right),avec P(O_{i}=k)=\frac{E_{i}^{k} e^{-E_{i}}}{k!}$).

We observe deaths in municipalities. For each municipality i, we know two things:

- **Oᵢ** = the number of observed deaths (what actually occurred)
- **Eᵢ** = the number of expected deaths (which we would have predicted based

The question is as follows: Is the difference between Oᵢ and Eᵢ significant, or is it due to chance? The Khi-Square (LnFr) statistic is the mathematical tool that answers this question. Thus, if mortality is normal in this municipality (no excess or submortality), then Oᵢ is a random draw around the expected value Eᵢ. For example, if Eᵢ = 10 expected deaths, what is the probability of observing exactly 14? We calculate [(10¹⁴ × e⁻¹⁰)/14! ], which is approximately 0.052. This model is standard in epidemiology for counting rare events.

# Let us now compare two classic scenarios in statistics: the null hypothesis H₀ and the alternative hypothesis H₁, defined as follows:

•	H₀ (null hypothesis): the municipality is normal; the true parameter of the Poisson distribution is exactly Eᵢ—there is no excess mortality.

•	H₁ (alternative hypothesis): something is happening; the true parameter is unknown, and we estimate it using the value that maximizes the likelihood—which turns out to be Oᵢ itself.

Likelihood measures how likely the observed data are, given a model. Mathematically, we prefer to use the logarithm (the log likelihood) to linearize the problem. For a Poisson distribution P(λ), the log likelihood of observing Oᵢ is written in formula (e) as follows:

l(λ) = Oᵢ ln(λ) − λ − ln(Oᵢ!)

This formula (e) is obtained simply by taking the logarithm of both sides of Poisson’s formula; the log likelihood is then calculated for both hypotheses H₀ and H₁.

Under H₀, the parameter is constrained to λ = Eᵢ; thus, we substitute lambda with $E_{i}$ as written in formula (f):

l₀ = Oᵢ ln(Eᵢ) − Eᵢ − ln(Oᵢ!)

Under H₁: the parameter is free; we choose it to maximize the likelihood. By differentiating l(λ) with respect to λ and setting it equal to zero as written in formula (g):

dl/dλ = Oᵢ/λ − 1 = 0 ⟹ λ̂ = Oᵢ

The best estimator is therefore Oᵢ itself (which makes sense: the best prediction of a count is the actual count). Substituting λ = Oᵢ as written in formula (h):

l₁ = Oᵢ ln(Oᵢ) − Oᵢ − ln(Oᵢ!)

Using these two values from formulas g and h, we perform the likelihood ratio test (LRT), which compares the two models by calculating twice their difference in log likelihood as written in formula (i):

LRT = 2 × (l₁ − l₀), which gives l₁ − l₀ = Oᵢ ln(Oᵢ/Eᵢ) − (Oᵢ − Eᵢ)

Hence, the final formula below is known as the Khi2LnFR formula (a), written as follows:

Khi2LnFRᵢ = 2 [Oᵢ ln(Oᵢ/Eᵢ) − (Oᵢ − Eᵢ)]

In formula (a), the factor Oᵢ ln(Oᵢ/Eᵢ) captures the relative deviation (the observed-to-expected ratio, in logarithms). The factor −(Oᵢ − Eᵢ) is a correction term that vanishes when Oᵢ = Eᵢ exactly.

When there is no excess mortality (Oᵢ ≈ Eᵢ), the two log likelihoods are nearly equal, their difference is close to 0, and Khi2LnFR ≈ 0. The more abnormal the municipality is, the greater the deviation, and the higher Khi2LnFR is—and Wilks’ theorem guarantees that under H₀, this statistic asymptotically follows a khi-square distribution with 1 degree of freedom, which allows us to set significance thresholds (Traffic-Lights: green/orange/red).

Formula (a) is as follows:

Khi2LnFRᵢ = 2 [Oᵢ ln(Oᵢ/Eᵢ) − (Oᵢ − Eᵢ)]

is of the form of this formula (j):

$\text{Khi2LnFR}_{i}=2E_{i}[(1+u)ln(1+u)-u]$, where$u=(O/E)_{i}-1$

# Let us show that$\text{Khi2LnFR}_{\boldsymbol{i}}\boldsymbol{\approx}\text{DENSI}_{\boldsymbol{i}}$

To do this, we use a limited Taylor–Maclaurin expansion of ln(1+u) into a Taylor series, in other words, into a polynomial of degree n, namely, (formula k):

$$ln(1+u)=u-\frac{u^{2}}{2}+\frac{u^{3}}{3}-\cdots$$

By multiplying by (1+u) and subtracting u, we obtain the following second-order approximation in formula (l):

$$(1+u)ln(1+u)-u\approx\frac{u^{2}}{2}$$

Let $\text{Khi2LnFR}_{i}\approx2E_{i}\cdot\frac{u^{2}}{2}=E_{i} u^{2}=E_{i}((O/E)_{i}-1)^{2}=\text{DENSI}_{i}$; thus, the original formula (c) is as follows:

$$\boxed{(O/E)_{i}\approx1+\sqrt{\frac{e^{\text{Khi2LnFR}_{i}}}{E_{i}}}=1+\frac{e^{\text{Khi2LnFR}_{i}/2}}{\sqrt{E_{i}}}}$$

Thus, the colors (green/orange/red) seem to indicate specific multiples of the average rate, as if the color red meant “always at least ×4.” However, this is not the case because the ratio $(O/E)_{i}$ corresponding to a given color threshold varies depending on $E_{i}$, as shown in Table 3.

Table 3: Correlations between verbal labels and O/E ratios

| Municipality | $E_{i}$ | $(O/E)_{i}$ at the orange/red threshold | Verbal labels |
| --- | --- | --- | --- |
| Very small | 5 | ≈ 3.90 | “×4–11×” |
| Average | 10 | ≈ 3.05 | “×4–11×” |
| Large | 50 | ≈ 1.92 | “×4–11×” |

The same red color may correspond to $(O/E)_{i}\approx2$ for a large municipality and to $(O/E)_{i}\approx4$for a very small one; these are indeed “ordinal approximations.” The order red > orange > green is indeed respected in terms of excess mortality, but the exact intervals in multiples of ($O/E)_{i}$ are not fixed—they depend on $E_{i}$ (the verbal labels are not a precise absolute measure).

## Setting thresholds on Khi2LnFR penalizes small populations:

A municipality with E=2 and $(O/E)_{i}$=4 is far less statistically significant than a municipality with E=50 and $(O/E)_{i}$=4. The verbal label “×4” is an approximate description for municipalities of typical size (E ≈ 5–20) and is not a universal mathematical guarantee.

Setting thresholds on Khi2LnFR rather than directly on O/E is statistically meaningful:

A municipality with E=2 that observes O=8 (OE = 4×) is much less statistically significant than a municipality with E=50 that observes O=200 (OE = 4× as well). The threshold on Khi2LnFR penalizes small sample sizes and avoids overalerting because of random fluctuations in very small numbers. The label “×4” is therefore an approximate description for municipalities of typical size (E ≈ 5–20) and not a universal mathematical guarantee.

References

[1] Wilks, S. S. (1938). The large-sample distribution of the likelihood ratio for testing composite hypotheses. The Annals of Mathematical Statistics, 9(1), 60–62.
