# Supplementary material for: Relevance of the uMap Collaborative Platform as Support for Choropleth Mapping of a Traffic-Light Statistical Signal Atlas of All-Cause Mortality During the First French Lockdown: Geospatial Analysis
Source: JMIR Med Inform. 2026 Aug 27;14:e82855. doi: 10.2196/82855 (PMC13543287; doi:10.2196/82855)
Supplement: Supplementary file 11 [file medinform-v14-e82855-s011.zip › BG_question2_legend_Fig8/_01_xnView_xnConvert_win10-64bits/XnViewMP-win-x64.zip!/XnViewMP/AddOn/exiftool_files/Licenses_Strawberry_Perl.zip!/License.rtf]

Strawberry Perl Distribution

This distribution is an aggregate of several different projects, licensed under various OSI-compatible Open Source licenses, or given to the public domain.

A summary of licenses for the component portions of this distribution is included below.

The full texts of these licenses are included in subdirectories of this directory.

Perl (core)
License

This program is free software; you can redistribute it and/or modify it under the terms of either:

	a)	the GNU General Public License as published by the Free Software Foundation; either version 1, or (at your option) any later version, or

	b)	the "Artistic License" which comes with this Kit.

Source Code

See info at http://www.cpan.org/src/README.html
 
Perl Modules Included in Strawberry Perl

There are many perl modules preinstalled in Strawberry Perl distribution. All of them are licensed under various OSI-compatible Open Source licenses. For getting license information concerning these modules use command: perldoc <ModuleName>

For the most part, modules will state that they are licensed identically to the Perl interpreter, but you may wish to check on the modules that you use.

A few license statements for libraries that are compiled into Perl modules statically are also mentioned below:

MD5 reference implementation (derivative used in Digest::MD5):

This library is free software; you can redistribute it and/or modify it under the same terms as Perl itself.

The MD5 algorithm is defined in RFC 1321. This implementation is derived from the reference C code in RFC 1321 which is covered by the following copyright statement:

Copyright (C) 1991-2, RSA Data Security, Inc. Created 1991. All rights reserved.

License to copy and use this software is granted provided that it is identified as the "RSA Data Security, Inc. MD5 Message-Digest Algorithm" in all material mentioning or referencing this software or this function.

License is also granted to make and use derivative works provided that such works are identified as "derived from the RSA Data Security, Inc. MD5 Message-Digest Algorithm" in all material mentioning or referencing the derived work.

RSA Data Security, Inc. makes no representations concerning either the merchantability of this software or the suitability of this software for any particular purpose. It is provided "as is" without express or implied warranty of any kind.

These notices must be retained in any copies of any part of this documentation and/or software.

This copyright does not prohibit distribution of any version of Perl containing this extension under the terms of the GNU or Artistic licenses.

PARI (included in Math::Pari):

PARI/GP is free software; you can redistribute it and/or modify it under the terms of the GNU General Public License as published by the Free Software Foundation. It is distributed in the hope that it will be useful, but WITHOUT ANY WARRANTY WHATSOEVER.

Check the License for details. You should have received a copy of it, along with the package; see the file 'COPYING'. If not, write to the Free Software Foundation, Inc., 59 Temple Place - Suite 330, Boston, MA 02111-1307, USA.

SQLite (included in DBD::SQLite):

All of the deliverable code in SQLite has been dedicated to the public domain by the authors. All code authors, and representatives of the companies they work for, have signed affidavits dedicating their contributions to the public domain and originals of those signed affidavits are stored in a firesafe at the main offices of Hwaci. Anyone is free to copy, modify, publish, use, compile, sell, or distribute the original SQLite code, either in source code form or as a compiled binary, for any purpose, commercial or non-commercial, and by any means. 
External Tools and Utilities Included in Strawberry Perl

Component			License and Source Code information
Binutils				subdirectory ./binutils
GCC compiler			subdirectory ./gcc
Mingw-w64-crt runtime		subdirectory ./mingw-w64-crt
Dmake				subdirectory ./dmake
GNU make			subdirectory ./make
Patch				subdirectory ./patch
Pexports			subdirectory ./pexports
External Libraries Included in Strawberry Perl

There are many external libraries (binaries + development files) included in Strawberry Perl distribution. For license and source code information concerning these libraries see corresponding subdirectory.
